# Structurally Constrained Cyclic (Diacyloxyiodo)Arenes as an Enabling Platform for Hypervalent Iodine(III) Chemistry

**DOI:** 10.1002/advs.202506041

**Published:** 2025-07-30

**Authors:** Shengyu Zhong, Shaoyan Gan, Xin Zhang, Lijuan Song, Lei Shi

**Affiliations:** ^1^ School of Chemistry and Chemical Engineering School of Science (Shenzhen) Harbin Institute of Technology Harbin 150001 China; ^2^ Fuyao University of Science and Technology Fuzhou 350000 China; ^3^ Shenzhen Bay Laboratory Shenzhen 518055 China

**Keywords:** cyclic (diacyloxyiodo)arenes, hypervalent iodine(III) chemistry, intramolecular secondary bonding, spatial dianion control

## Abstract

Hypervalent iodine(III) chemistry has unlocked remarkable potential in synthetic and catalytic applications over the past decades, yet its development has been constrained by biased thermodynamics between iodine(I) and iodine(III) species, relying on unstable or environmentally unfriendly oxidants and complex electrocatalytic platforms. Here, structurally constrained cyclic (diacyloxyiodo)arenes are presented as a transformative platform to resolve the reactivity/selectivity‐generality paradox, enabling one‐pot synthesis of diverse hypervalent iodine(III) reagents. The synthetic utility and sustainability of the in situ controlled‐release approach are demonstrated through catalytic reactions, asymmetric variations, and biocompatibility studies, including peptide modifications. Central to this success is the discovery of cyclic (diacyloxyiodo)arenes—a previously unexplored chemical entity. DFT calculations reveal that spatial dianion control via intramolecular secondary bonding enhances reactivity and selectivity while delivering superior outcomes.

## Graphical abstract

We developed cyclic (diacyloxyiodo)arenes to overcome the reactivity/selectivity paradox in iodine chemistry. This new platform enables simple, one‐pot synthesis of diverse hypervalent iodine(III) reagents. The method is sustainable, catalytic, works for asymmetric reactions, and is biocompatible (e.g., modifying peptides). DFT shows their structure enhances reactivity/selectivity via intramolecular bonding.

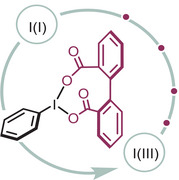

## 1 Introduction

Hypervalent iodine(III) species, termed organic‐λ_3_‐iodanes and organic‐λ_3_‐iodonium salts, have enabled versatile organic transformations including selective oxidations, oxidative rearrangements, and functional group transfer reactions in an umpolung fashion.^[^ ^1^, ^2^, ^3^ ^]^ Three key factors underlie their exceptional reactivity: electron‐deficient nature, ligand exchange capability, and excellent leaving group ability. Despite extensive research since their discovery, inherent limitations remain, including instability under strong bases, in the presence of transition metals, or upon heating, and stoichiometric iodoarene byproduct formation. Furthermore, the relationship between ligands and their dominant iodine speciation (especially the preferred oxidation state and structural features) remains poorly understood. Owing to the high oxidation potentials of aryl iodides (E_1/2, PhI_ = 2.17 V vs SCE),^[^ ^4^ ^]^ hypervalent iodine(III) reagent synthesis typically requires judicious choice of terminal oxidants^[^ ^1^, ^5^ ^]^ or anodic oxidation.^[^ ^6^ ^]^ In this context, inefficient reoxidation of ArI to hypervalent iodine(III) species precludes the establishment of an efficient catalytic cycle, often necessitating (super)stoichiometric amounts of hypervalent iodine(III) reagents. Thus, the current limitations of hypervalent iodine(III) reagents are often linked to safety, atom economy, sustainability, and biocompatibility concerns.

In 2005, Kita and Ochiai independently reported iodoarene‐catalyzed oxidations of phenols^[^ ^7^ ^]^ and carbonyl compounds^[^ ^8^ ^]^ using *meta*‐chloroperbenzoic acid (*m*CPBA) as the terminal oxidant, which minimized potential complications from side reactions of the oxidant with starting materials or products. Since then, research has focused on developing novel iodoarenes as organocatalysts,^[^ ^9^, ^10^ ^]^ including chiral variants. Recently, the use of molecular oxygen (O_2_) has been explored to synthesize some hypervalent iodine(III) reagents and to perform a few iodoarene‐catalyzed transformations, using excess aldehydes^[^ ^11^, ^12^, ^13^ ^]^ or photocatalysis,^[^ ^14^ ^]^ because the single‐electron oxidation potential of iodobenzene (E_1/2, PhI_ = 2.17 V vs SCE)^[^ ^4^ ^]^ is much higher than the reduction potential of oxygen (−0.33 V vs SHE, for single‐electron reduction at pH 7).^[^ ^15^ ^]^ To date, the synthesis of hypervalent iodine(III) reagents from aryl iodides^[^ ^1^, ^5^ ^]^ and attempts at asymmetric hypervalent iodine(III) catalysis^[^ ^9^, ^10^ ^]^ still rely on stoichiometric or excess oxidants, notably *m*CPBA and Selectfluor. Furthermore, the precise structures of the catalytic species — or even the actual catalyst — in hypervalent iodine(III) catalysis remain elusive, owing to complications arising from interactions between aryl iodides and oxidants.^[^ ^9^ ^]^ For example, oxidation of aryl iodides with *m*CPBA reportedly yields mixtures of hypervalent iodine species with varying oxidation states, including iodosylarenes, iodylarenes, (dicarboxyliodo)arenes, and oxo‐*µ*‐aryliodanes.^[^ ^7^, ^8^, ^16^ ^]^

**Scheme 1:**
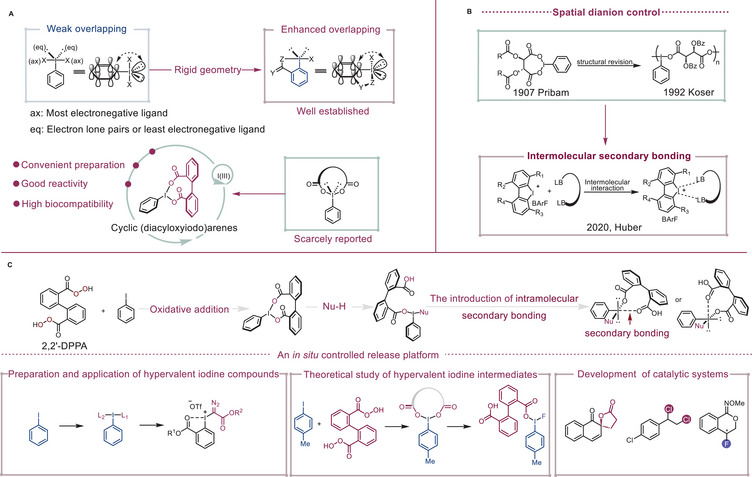
A) General structure of aryl‐substituted hypervalent iodine(III) species. B) Previous exploration of other coordination patterns in hypervalent iodine(III) reagents. C) This work: cyclic (diacyloxyiodo)arenes as an in situ controlled release platform and subsequent generation of secondary‐bonding‐assisted organoiodine(III) species by the additional internal coordination of iodine atom.

Recent advances in enhancing the stability and atom economy of hypervalent iodine(III) reagents focus on bridging equatorial and apical positions via five‐ or six‐membered rings (**Scheme** **1**A).^[^ ^17^, ^18^, ^19^, ^20^ ^]^ Molecules with a central atom capable of diverse coordination numbers or oxidation states exhibit greater versatility in molecular geometry, facilitating access to unconventional electronic structures and reactivity. In 2020, the Čorić group demonstrated that a structurally constrained dicarboxylate ligand around palladium significantly boosted reactivity in arene‐limited C─H arylation by modulating spatial arrangements through a bisanion backbone distortion.^[^ ^21^ ^]^ Recognizing the transition metal‐like properties of organoiodine(III) redox chemistry, we hypothesized that imposing a cyclic coordination structure spanning two apical positions could provide precise redox control to modulate the properties and reactivity of hypervalent iodine(III) species. Additionally, such a ligand was expected to introduce additional intramolecular halogen‐bonding interactions^[^ ^22^ ^]^ to enforce highly directional discrimination during ligand exchange and beyond (Scheme 1B). Notably, organoiodine redox chemistry using tethered dicarboxylate ligands as rigid linkers remains rare and underdeveloped.^[^ ^23^, ^24^, ^25^, ^26^ ^]^ The main challenge might stem from the difficulty in finding a suitable backbone that connects two apical positions at a comparatively remote distance, similar to the previously reported spatial anion control on a palladium center.^[^ ^21^ ^]^ Indeed, earlier cyclic iodine(III) succinate derivatives were reclassified as non‐cyclic polymers following detailed structural analysis by the Koser group.^[^ ^23^, ^25^ ^]^ Thus, our rational design hinges on discovering a geometrically constrained dicarboxylate ligand to enable in situ generation of cyclic (diacyloxyiodo)arene active species. If successful, such a strategy would not only allow deeper understanding of organoiodine(III) speciation but also offer an unrecognized opportunity to further expand the organoiodine(III) chemical space. Here, we report 2,2′‐diperoxyphenic acid (2,2′‐DPPA) as a bench‐stable, efficient alternative to *m*CPBA and Selectfluor, enabling rapid synthesis of diverse hypervalent iodine(III) reagents and establishing a versatile platform with enhanced synthetic utility and biocompatibility (Scheme 1C). Moreover, structural and computational analyses further confirm the in situ formation of cyclic (diacyloxyiodo)arenes, revealing intramolecular halogen bonds that modulate structure and reactivity during ligand exchange.

## 2 Results and Discussion

### 2.1 Optimization of the Reaction Conditions

As noted, oxidant selection critically governs the transformation's efficacy. Thus, we initiated reactivity screening of iodobenzene (**1a**) with bisperoxides and cyclic peroxides, anticipating that in situ formation of cyclic (diacyloxyiodo)arenes during the synthesis of diphenyliodonium salt (**3a**) and α‐phenyliodonio diazoacetate (**8a**) would be feasible with these oxidants. Notably, 2,2′‐DPPA outperformed all other oxidants in both model reactions, yielding the products rapidly and in satisfactory yields (**Tables** **1** and **2**). In contrast, phthaloyl peroxide (PPO) and malonoyl peroxides (MPO 1–5) failed to generate any products even after extended reaction times, indicating that the geometric constraints of the hypervalent iodine(III) intermediate significantly affect its reactivity. After optimizing the conditions (Tables 1 and 2; Tables S1–S6, Supporting Information), the reaction of **1a** (1 equiv), benzene (2 equiv), 2,2′‐DPPA (0.75 equiv), and trifluoromethanesulfonic acid (TfOH, 1.5 equiv) in DCM (0.1 m) for 10 min produced diphenyliodonium salt **3a** in 84% yield. Similarly, reacting **1a** (1 equiv), ethyl diazoacetate (**7a**, 2 equiv), and trimethylsilyl trifluoromethanesulfonate (TMSOTf, 1.1 equiv) with 2,2′‐DPPA (0.7 equiv) in DCM (0.1 m) for 30 min afforded α‐phenyliodonio diazoacetate **8a** in 87% yield. Extending the reaction time to 1 h (Table 1, entry 8; Table 2, entry 8) slightly reduced the yields in both cases. In both categories, gram‐scale syntheses using 2,2′‐DPPA maintained efficiency, confirming the practicality of this method. Notably, 2,2′‐DPPA is easy to handle, exhibiting bench and air stability and insensitivity to shock and friction as a solid at room temperature.^[^ ^27^ ^]^ Specifically, TGA and DSC measurements showed that 2,2′‐DPPA has superior thermal stability (T₀ = 101.9 °C, Q = 173.7 cal g^−1^) compared to *m*CPBA (T₀ = 89 °C, Q = 290 cal g^−1^; Figure S3, Supporting Information),^[^ ^28^, ^29^ ^]^ a shelf‐stable *m*‐chlorobenzoic acid/water mixture. Notably, traditional methods to prepare diphenyliodonium salts using *m*CPBA^[^ ^29^, ^30^ ^]^ required elevated temperatures (40–80 °C) or prolonged reaction times with excess TfOH (2–3 equiv).^[^ ^31^, ^32^ ^]^ Moreover, *m*CPBA failed to generate α‐diazoiodonium triflate **8a** even with 3 equiv TMSOTf, yielding only *α*‐diazoiodonium 3‐chlorobenzoate (**DICB**) without further counterion exchange (Table 2, entry 4). Taken together, these observations led us to hypothesize that the enhanced reaction rates and reduced TfOH or TMSOTf requirements for these two model reactions could be attributed to the high reactivity of the geometrically constrained cyclic iodine(III) species and the excellent anion‐exchange ability of the secondary‐bonding‐assisted organoiodine(III) species. Sensitivity assessment indicated minor water sensitivity (likely from product instability in water), whereas temperature, concentration, light, oxygen, and scale had minimal impact on the yields (Tables S8 and S10, Supporting Information).^[^ ^33^ ^]^

**Table 1:** Optimization of the synthesis of diaryliodonium salts.

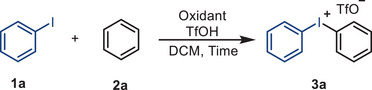				
Entry^a^ ^)^	Oxidant	Time	TfOH	Yield^b^ ^)^
1	2,2′‐DPPA(0.6 equiv)	10 min	1.1 equiv	51%
2	PPO(0.6 equiv)	10 min	1.1 equiv	N.D.
3	MPO 1–5(0.6 equiv)	10 min	1.1 equiv	N.D.
4	2,2′‐DPPA(0.6 equiv)	10 min	1.1 equiv	68%
5	2,2′‐DPPA(0.6 equiv)	10 min	1.1 equiv	70%
6	2,2′‐DPPA(0.6 equiv)	10 min	1.1 equiv	62%
7	2,2′‐DPPA(0.6 equiv)	10 min	1.1 equiv	84%(81%^c^ ^)^)
8	2,2′‐DPPA(0.6 equiv)	1 h	1.1 equiv	78%

^a)^ Reaction conditions: **1a** (0.3 mmol), oxidant, TfOH, and **2a** (0.6 mmol) dissolved in DCM (3 mL) at room temperature;

^b)^ Isolated yields;

^c)^ Substrate **1a** (5 mmol). N.D., not detected.

**Table 2:** Optimization of the synthesis of *α*‐diazoiodonium salts.

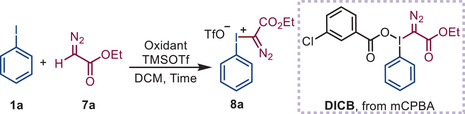				
Entry^a)^	Oxidant	Time	TMSOTf	Yield^b)^
1	2,2′‐DPPA(0.55 equiv)	10 min	1.1 equiv	55%
2	PPO(1.1 equiv)	10 min	1.1 equiv	N.D.
3	MPO 1–5(1.1 equiv)	10 min	1.1 equiv	N.D.
4	*m*CPBA(1.1 equiv)	10 min	1.1 equiv	N.D.
5	2,2′‐DPPA(0.7 equiv)	10 min	1.1 equiv	58%
6	2,2′‐DPPA(0.7 equiv)	10 min	2.0 equiv	65%
7	2,2′‐DPPA(0.7 equiv)	30 min	2.0 equiv	87%(84%^c)^)
8	2,2′‐DPPA(0.7 equiv)	1 h	2.0 equiv	62%
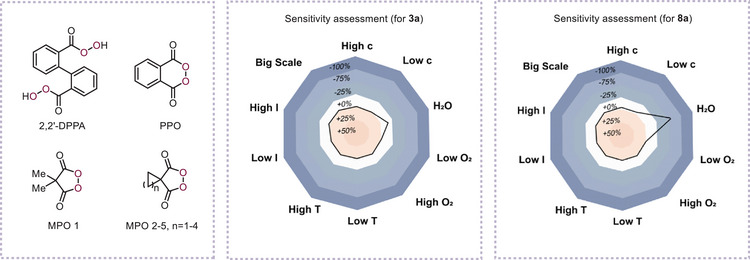				

^a)^ Reaction condition: **1a** (0.3 mmol), oxidant, TMSOTf (0.33 mmol), and **7a** dissolved in DCM (3 mL) at room temperature;

^b)^ Isolated yields;

^c)^ Substrate **1a** (5 mmol). N.D., not detected.

### 2.2 Preparations and Applications of Hypervalent Iodine(III) Reagents

**Scheme 2:**
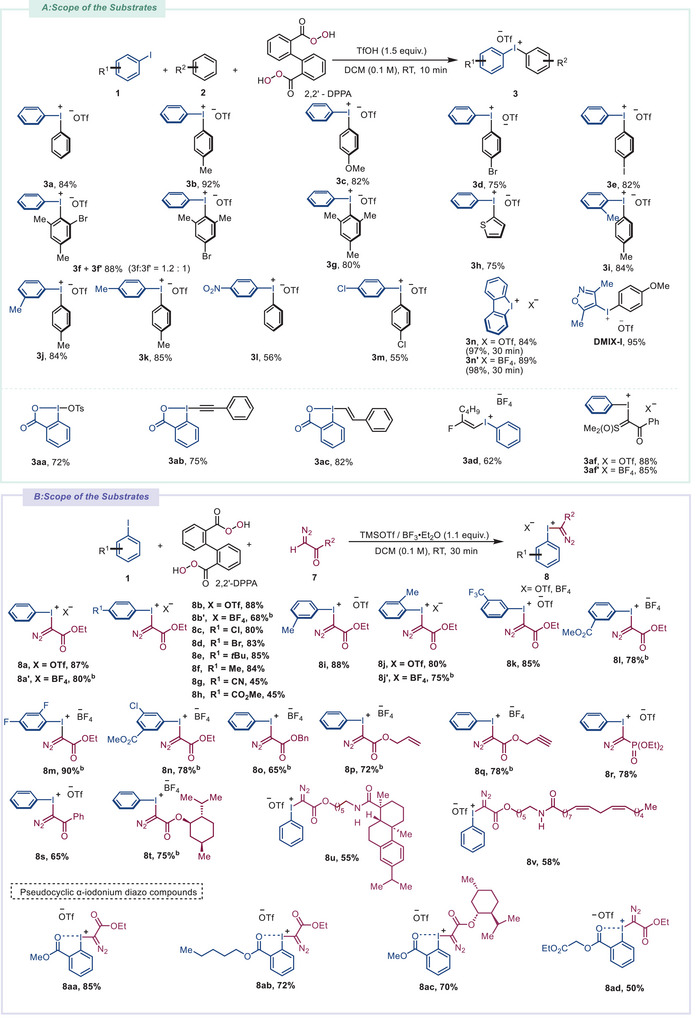
One‐pot preparation of hypervalent iodine(III) reagents. A) One‐pot preparation of hypervalent iodine(III) reagents and efficient preparation of target compounds. B) One‐pot preparation of α‐diazoiodonium salts and efficient preparation of target compounds.

With optimized conditions in hand, we explored the scope of hypervalent iodine(III) reagent synthesis (**Scheme** **2**A). The reaction of **1a** with monosubstituted benzenes (**2a**–**2e**) in the presence of 2,2′‐DPPA and TfOH afforded (4‐substituted phenyl)phenyliodonium triflates (**3a**–**3e**) in 75–92% yields, with strong *para*‐selectivity attributed to the steric hindrance of the cyclic organoiodine(III) intermediate during electrophilic aromatic substitution (EAS). Notably, 4‐iodophenyl(phenyl)iodonium triflate (**3e**) was obtained in the case where iodobenzene served a dual role as both aryl iodide and arene. Treating **1a** with 1‐bromo‐3,5‐dimethylbenzene (**2f**) yielded an inseparable mixture of regioisomers **3f** and **3f′** (combined 88% yield, 1.2:1 regioselectivity). Thiophene, as a π‐electron‐excessive heteroarene, underwent electrophilic aromatic substitution at its α‐position to form **3** **h**. This protocol efficiently introduced electron‐rich groups (e.g., mesityl in **3** **g**, thienyl in **3** **h**) as inert ligands to enable selective arylation.^[^ ^34^ ^]^ Monosubstituted aryl iodides (*ortho*, *meta*, and *para*; electron‐donating or ‐withdrawing) all successfully generated diaryliodonium triflates (**3i**–**3m**). Similarly, cyclic diaryliodonium triflate **3n** and an analog (**3n′**) were prepared by replacing triflic acid with other acids. Beyond diaryliodonium salts, 2,2′‐DPPA enabled the synthesis of diverse iodine(III) reagents, including tosyloxybenziodoxolone (**3aa**),^[^ ^35^ ^]^ alkynyl benziodoxolone (**3ab**),^[^ ^36^ ^]^ alkenyl benziodoxoles (**3ac**),^[^ ^37^ ^]^ *β*‐fluorovinyliodonium salt (**3ad**),^[^ ^38^ ^]^ and mixed iodonium‐sulfoxonium ylides (**3ae** and **3ae′**).^[^ ^39^ ^]^ We developed efficient one‐pot syntheses of high‐value *ortho*‐iodo diaryl ethers **5** and isoquinolines **6**. This was achieved by leveraging the controlled in situ generation of diverse hypervalent iodine(III) species from cyclic (diacyloxyiodo)arenes, enabling direct access without intermediate isolation and affording excellent yields (see Sections S7.4 and S7.5, Supporting Information for details).^[^ ^40^, ^41^ ^]^

*α*‐iodonium diazo compounds that previously required lengthy syntheses. Treating ethyl diazoacetate with substituted iodoarenes in the presence of 2,2′‐DPPA and an acid (TMSOTf or BF_3_·Et_2_O) provided α‐aryliodonio diazoacetates **8a**–**8n** in uniformly high yields. However, the use of 4‐iodoanisole failed to yield the desired product, presumably due to the extreme instability of that α‐iodonium diazo species.^[^ ^3^, ^42^, ^43^ ^]^ Further exploration of diazomethane derivatives, including aliphatic diazoesters with different substituents, an α‐diazo carbonyl compound, and diethyl (diazomethyl)phosphonate, afforded the corresponding α‐iodonium diazo compounds (**8o**–**8s**) in 48–78% yields. The successful late‐stage derivatization of structurally complex bioactive molecules (**8t**–**8v**) demonstrates the practical potential of our in situ controlled‐release technology. Moreover, the method's compatibility with oxidation‐sensitive functional groups (e.g., ketone, alkene, alkyne) under our standard conditions highlights its synthetic utility. Importantly, a range of pseudocyclic α‐iodonium diazo compounds (**8aa**–**8ad**) were also obtained in good yields under the same reaction conditions. Compared to literature methods,^[^ ^44^, ^45^, ^46^, ^47^ ^]^ this approach offers greater simplicity and broader scope, expanding accessible chemical space (Scheme 2B). The reactivity of **8aa** was assessed using multiple classical reactions that often give unsatisfactory yields. Significantly, these reactions proceeded to furnish the corresponding products in higher yields (ethyl 1‐benzoyl‐1H‐diazirine‐3‐carboxylate **10**, ethyl 2‐diazo‐2‐(2‐(dimethylamino)phenyl) acetate **12**, ethyl 2‐diazo‐4‐methoxy‐4‐phenylbutanoate **14**, ethyl 2‐naphthoate **15**, see Sections S7.6–S7.9, Supporting Information for details).^[^ ^43^, ^48^, ^49^, ^50^ ^]^

### 2.3 I(I)/I(III) Catalysis by Leveraging 2,2′‐DPPA as the Terminal Oxidant

**Scheme 3:**
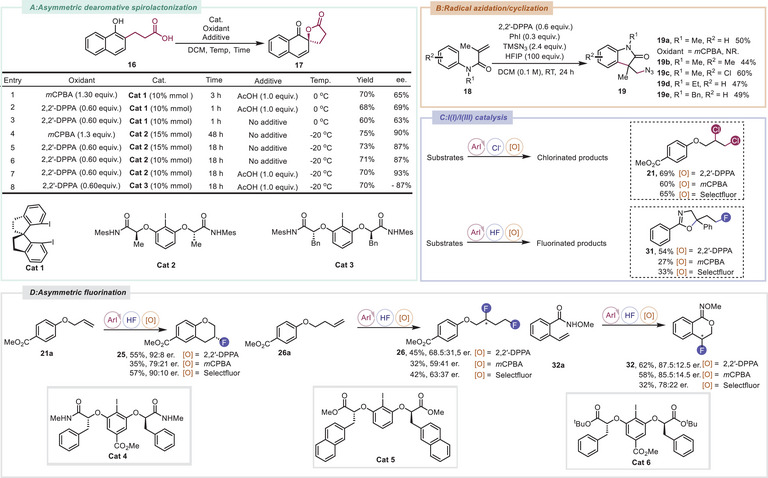
Cyclic (diacyloxyiodo)arenes as an in situ controlled release platform for I(I)/I(III) catalysis. A) Catalytic asymmetric dearomatization of naphthols using chiral iodoarene catalysts. B) Metal‐free azidoarylation of alkenes. C) I(I)/I(III)‐Catalyzed chlorination and fluorination reactions. D) I(I)/I(III)‐Catalyzed asymmetric fluorination reactions.

In recent years, the importance of catalytic systems based on aryl iodide precatalysts has gained significant recognition.^[^ ^5^, ^9^ ^]^ Accordingly, we set out to use 2,2′‐DPPA as the terminal oxidant to establish an efficient I(I)/I(III) catalytic platform. For the asymmetric dearomative spirolactonization of propanoic acid‐substituted 1‐naphthol (**16**), we systematically investigated *C*_2_‐symmetric iodoarene catalysts (**Cat 1**–**3**) combined with 2,2′‐DPPA (**Scheme** **3**A). In contrast to previous asymmetric catalytic variants that required superstoichiometric *m*CPBA as a co‐oxidant,^[^ ^51^, ^52^, ^53^ ^]^ the addition of 0.6 equiv of 2,2′‐DPPA to the reaction mixture of substrate **16** with 10 mol% of the rigid 1,1′‐spirobiindane‐based iodoarene catalyst (**Cat 1**) or the flexible lactate‐derived iodoarene catalyst (**Cat 2**) in dichloromethane afforded product **17** in comparable yield and with slightly improved enantiomeric excess (ee) in a significantly shorter reaction time. The rate acceleration suggested that subtle structural differences in the in situ‐generated hypervalent iodine(III) species (arising from different oxidants) had a strong influence on reactivity. Additionally, the introduction of a polarizable benzylic group at the catalyst's (**Cat 3**) stereogenic center failed to improve the catalytic performance, suggesting a negligible influence of steric factors or aromatic interactions. Furthermore, the combination of an in situ‐formed cyclic (diacyloxyiodo)arene with azide generated an azide‐containing hypervalent iodine(III) species, enabling a tandem radical azidation/cyclization of N‐arylacrylamide (**18**) to produce indolin‐2‐one products in good yields (Scheme 3B).^[^ ^54^ ^]^ Significantly, using 30 mol% PhI as a catalyst yielded 3‐disubstituted indolin‐2‐ones (**19a**–**19e**) in 44–60% yields, providing proof‐of‐principle that PhI can serve as a catalyst. When 2,2′‐DPPA was replaced with *m*CPBA, the reaction failed to proceed. We hypothesized that the limited catalyst turnover arises from the inherent instability of the diazidoiodinane intermediate.^[^ ^55^ ^]^ Control experiments confirmed that no product formed in the absence of iodobenzene.

Building on these findings, we established a versatile iodoarene‐catalyzed platform using 2,2′‐DPPA as the oxidant and ArI as a cost‐effective catalyst under I(I)/I(III) catalytic cycling. This system enabled efficient functionalization of diverse substrates with tunable halogen sources. By modulating the reagent (TMSCl or Olah's reagent), selective incorporation of distinct halogen motifs was achieved, delivering structurally varied products across multiple substrate classes (Scheme 3C, see Supporting Information for details).^[^ ^56^, ^57^, ^58^, ^59^, ^60^, ^61^, ^62^, ^63^ ^]^ Notably, the oxazoline compound **31**, obtained from ring‐opening fluorocyclization of an unactivated cyclopropane, represents a novel fluorinated scaffold that is unprecedented in the literature. It is also important to note that when 2,2′‐DPPA was utilized as the terminal oxidant, the yields obtained were generally higher than those achieved with the frequently used oxidants *m*CPBA and selectfluor. Having established achiral iodoarene‐catalyzed fluorofunctionalization reactions, we focused on developing enantioselective processes to access structural motifs with secondary carbon–fluorine stereocenters. Compounds **21a**, **26a**, and **32a** were selected as model substrates for optimization. Following extensive optimization of reaction conditions, the chiral 3‐fluorochromane **25**, the 1,3‐difluorinated product **26**, and the fluorocyclization product **32** were successfully synthesized. Remarkably, **Cat 4** and **Cat 6** effectively catalyzed aryl allyl ether fluorocyclization and fluoroiminolactonization, delivering products **25** and **32** with good yields and promising enantioselectivity. However, 1,3‐difluorinated product **26** showed limited ee (37%) despite extensive catalyst screening. Despite the modest enantioselectivity (37% ee), this work represents the first catalytic enantioselective 1,3‐difluorination of homoallylic (aryl) ethers via transient oxonium intermediates,^[^ ^61^ ^]^ and it demonstrates significant potential for further optimization (Scheme 3D). Notably, the use of 2,2′‐DPPA as the terminal oxidant demonstrated significant advantages over conventional alternatives, achieving enhanced yields and superior enantiocontrol.

## 3 Structural Evaluation and Analysis

To acquire underlying structural information on the combination of iodoarenes and 2,2′‐DPPA, 4‐iodotoluene (**1k**, 0.5 mmol) and 2,2′‐DPPA (1 mmol) were reacted in DCM (0.1 m, RT, 1 h). Post‐reaction filtration removed insoluble iodosylarenes, and vacuum concentration yielded a white powder. ^1^H/^1^ ^3^C NMR analysis revealed it was a mixture of different hypervalent iodine(III) species, evidenced by strong ipso‐carbon shielding in ^1^ ^3^C NMR^[^ ^64^ ^]^ The ^1^H NMR spectra revealed the aromatic region (6.8–8.2 ppm) integral area was about four times that of the methyl signals (1.0–2.5 ppm), indicating a 1:1 molar ratio of 4‐iodotoluene to 2,2′‐DPPA moieties. LC‐MS and HRMS confirmed the coexistence of cyclic (diacyloxyiodo)arenes and their soluble dimeric, trimeric, and oligomeric organoiodine(III) species, which might resemble main‐group organometallic reagents in Schlenk equilibrium,^[^ ^65^ ^]^ along with a small quantity of µ‐*oxo*‐bridged bisiodine(III) derivative. Strikingly, reacting the crude powder with toluene (**2k**) or ethyl diazoacetate (**7a**) led to rapid formation (within <1 min) of **3k** or **8f** in improved yields, confirming that cyclic (diacyloxyiodo)arenes are the active species.

**Scheme 4:**
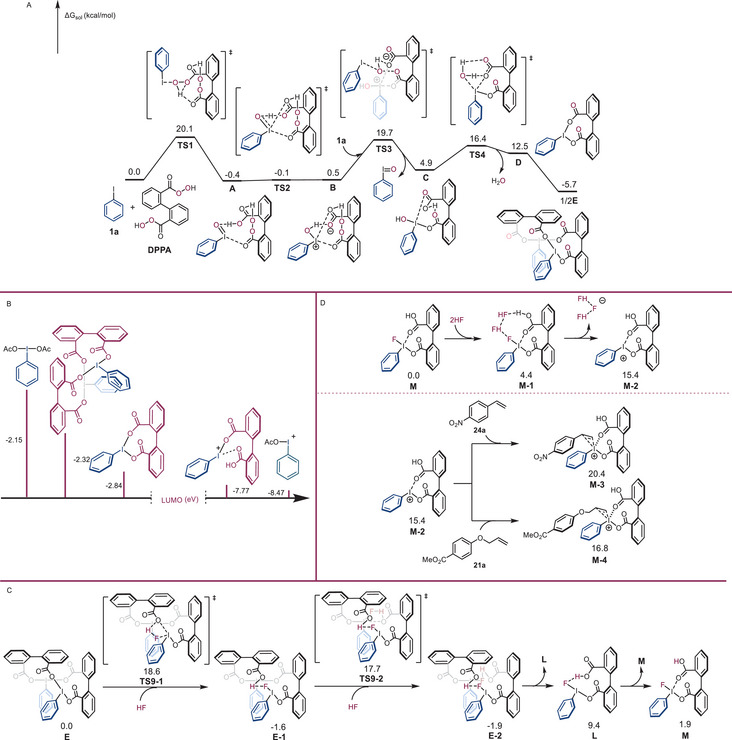
Computational analysis of cyclic (diacyloxyiodo)arenes. A) Calculated potential energy profile for forming cyclic (diacyloxyiodo)arenes and analogs. B) DFT calculated LUMO energies (eV) for cyclic (diacyloxyiodo)arenes and analogs. C) Free energy profile of the pathway to generate key intermediate **M**. D) Computed pathways to generate olefin adducts catalyzed by iodoarene fluoride **M**.

To investigate the reaction of iodoarenes and 2,2′‐DPPA, density functional theory (DFT) computations were performed using iodobenzene **1a** and 2,2′‐DPPA (**Scheme** **4**A). The computational results revealed that the reaction proceeds through the oxidation of two iodobenzene molecules to form intermediate **D**, accompanied by the release of iodosylbenzene and one molecule of H_2_O. Both oxidation steps with energy barriers of 20.1 kcal mol^−1^ were found to be stabilized by intramolecular hydrogen‐bonding networks and halogen bonding (Figure S19, Supporting Information). Meanwhile, the reaction of iodosylbenzene with 2,2′‐biphenyldicarboxylic acid, which usually appears as a byproduct, to regenerate intermediate **D** is thermodynamically favorable. Moreover, intermediate **D** can dimerize into the more stable intermediate **E** due to its favorable T‐shape geometry of the iodane atom. Alternative pathways from intermediate **C** with higher energy barriers can be excluded (see Figures S18–S22, Supporting Information for details). Molecular orbital analysis indicates that the structurally constrained cyclic (diacyloxyiodo)arenes lower the lowest unoccupied molecular orbital (LUMO) energy of hypervalent iodine(III) reagent (−2.84 eV vs −2.15 eV), resulting in species a stronger Lewis acid and oxidant (Scheme 4B).^[^ ^31^ ^]^ These DFT computations are consistent with the experimental observations and offer insights into the structure and reactivity of organoiodine(III) oligomers.

Building upon the DFT results, it was observed that the intermediates generated from the reaction of 2,2′‐DPPA with aryl iodides differ from those produced using oxidants like Selectfluor or *m*CPBA, suggesting that iodoarene (III) difluoride is unlikely to be formed during hypervalent iodine(III)‐catalyzed fluorinations. To probe the active species, cyclic (diacyloxyiodo)arenes were synthesized and in situ treated with an HF source in DCM. ^19^F NMR showed a distinct signal at −133 ppm, which represents a significant shift from the previously reported PhIF_2_ signal observed at around −177 ppm,^[^ ^66^ ^]^ suggesting the formation of an iodine(III) carboxylate **M**. This observation was further confirmed by LC‐MS analysis, which identified a molecular ion at m/z consistent with the molecular weight of **M**.(Figure S11, Supporting Information).

Further DFT computations were conducted to gain mechanistic insights into iodoarene‐catalyzed fluorofunctionalization. The stable dimeric intermediate **E** would react with HF to generate the active catalytic species fluoro‐iodane **L** with low activation barriers of 18.6 and 19.3 kcal mol^−1^ (Scheme 4C). The intermediate **L** could isomerize to a more stable fluorinated intermediate **M**, where the flexible carbonyl oxygen coordinates to the iodine center so that there are six electron pairs surrounding the iodine atom. The formation of iodoarene difluoride **N** is less favorable, which has higher barriers of 18.7 and 21.5 kcal mol^−1^ (Figure S23, Supporting Information). Similar to the mechanism for the aryl iodine‐catalyzed fluorination reactions,^[^ ^60^, ^62^ ^]^ two molecules of HF were employed for iodoarene fluoride **M** (Scheme 4D). Coordination of the two HF molecules leads to the hydrogen‐bonded complexes **M‐1** and **N‐1** (Scheme 4C; Figures S25 and S26, Supporting Information), followed by fluoride loss to generate the cationic active catalytic species. Olefin substrates then coordinate to the cationic species to give iodonium ion intermediates. The pathways catalyzed by iodoarene fluoride **M** exhibit lower energy profiles, probably due to the stabilization from the four‐electron pair coordination (Scheme 4D).

## 4 Biocompatibility Evaluation

**Scheme 5:**
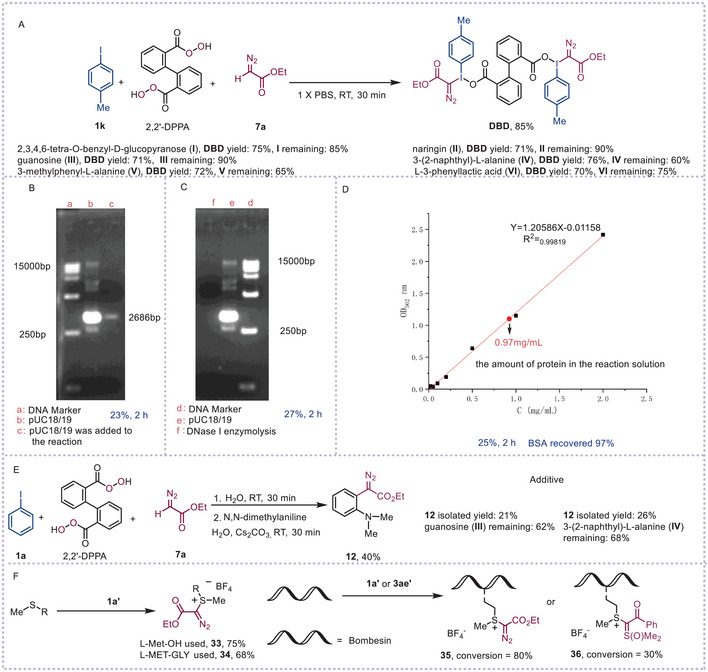
Biocompatible reactions and bio‐additive‐based robustness screen. A) The compatibility of external additives in the preparation of bis(α‐diazoiodonium) 2,2'‐biphenyldicarboxylate. B) Agarose gel electrophoresis to test the activity of pUC 18/19. C) Agarose gel electrophoresis to test the activity of DNase I. (f: the reaction solution containing DNase 1). D) The concentration of BSA was calculated from a standard curve. E) One‐pot transformation for the synthesis of ethyl 2‐diazo‐2‐(2‐(dimethylamino)phenyl) acetate **12** in water. F) Chemoselective labelling at methionine residues.

To enhance bio‐robustness and biocompatibility,^[^ ^67^ ^]^ we synthesized α‐iodonium diazo compounds under biologically ambient conditions (aqueous solvent, pH 6–8, <37 °C), directly usable in subsequent transformations without purification or solvent exchange. Initial attempts to prepare α‐phenyliodonio diazoacetate triflate (**8a**) and tetrafluoroborate (**8a**′) in 1× PBS buffer (pH ≈7) using TMSOTf or BF_3_·Et_2_O failed. However, bis(α‐diazoiodonium) 2,2′‐biphenyldicarboxylate (**DBD**) was efficiently obtained (85% yield) in PBS without anion exchange, offering environmental and yield advantages (**Scheme** **5**A).^[^ ^68^, ^69^, ^70^ ^]^ A robustness screen assessed tolerance to pharmacophores and functional groups. Adding equimolar 2,3,4,6‐tetra‐O‐benzyl‐D‐glucopyranose (**I**), naringin (**II**), guanosine (**III**), 3‐(2‐naphthyl)‐L‐alanine (**IV**), 3‐methylphenyl‐L‐alanine (**V**), or L‐3‐phenyllactic acid (**VI**) minimally impacted the reaction, with 60–90% additive recovery. This fully aqueous system demonstrated compatibility with amines, alcohols, phenols, acetals, carboxylic acids, and the guanine nucleobase. Adding pUC18/19 plasmid DNA or BSA minimally affected the reaction, yielding **DBD** in acceptable yield. A conversion drop occurred, likely due to shaking instead of stirring. Plasmid integrity was assessed via agarose gel electrophoresis (Scheme 5B),^[^ ^71^ ^]^ no new bands appeared, and the original pUC18/19 band remained (lane c), confirming no DNA fragmentation. With DNase I and agitation, **DBD** formed in 27% yield (81% starting material recovered; Scheme 5C). Plasmid degradation (lane f) confirmed active DNase I. Post‐reaction BSA concentration (2 h, RT) measured 0.97 mg mL^−1^ (Scheme 5D), similar to the initial level, confirming minimal protein alteration. Using water‐compatible **DBD**, a one‐pot reaction with *N,N*‐dimethylaniline under aqueous conditions yielded ethyl 2‐diazo‐2‐(2‐(dimethylamino)phenyl)acetate (**12**) moderately, even with bio‐additives (Scheme 5E).

We next explored cyclic (diacyloxyiodo)arenes as a controlled‐release platform for complex peptide systems with diverse side‐chain functionalities. In 2018, Gaunt's group achieved site‐selective methionine bioconjugation under aqueous biological conditions using electrophilic linear α‐aryliodonio diazo compounds.^[^ ^72^ ^]^ However, reactant limitations—poor solubility, instability, or susceptibility to hydrolysis—hindered target product formation. For instance, reacting phenyliodonio diazoacetate triflate (**8a**) with dipeptides in water gave a low yield (27%) of the desired product, owing to **8a**’s instability in aqueous media.^[^ ^72^ ^]^ Since counteranions influence α‐diazoiodonium salt solubility and reactivity, we developed an in situ solvent‐switch method to generate α‐diazoiodonium tetrafluoroborates in water, enabling selective functionalization of amino acids, dipeptides, and complex polypeptides. Specifically, complete solvent evaporation followed by water addition allowed direct functionalization of L‐methionine (L‐Met‐OH) and L‐methionyl‐glycine (L‐Met‐Gly) without isolating intermediate iodonium salts (Scheme 5F). For bombesin, efficient conversion to sulfonium conjugate **35** was achieved using dilute thiourea (100 mm), TEMPO (100 mm), and formic acid (33 mm, pH ≈3).^[^ ^72^ ^]^ Unlike prior methods, this approach eliminated the need for a 2,4‐difluorophenyl group (Scheme 5F). Bombesin also reacted with iodonium‐sulfoxonium ylide **3ae′** in a one‐pot, two‐step process to yield product **36**, albeit with low conversion due to **3ae′**’s poor solubility. These results underscore the platform's potential for site‐selective peptide/protein modifications while maintaining structural and functional integrity.

## 5 Conclusion

Here, we disclose the first utilization of bench‐stable 2,2′‐diperoxyphenic acid to construct structurally constrained cyclic (diacyloxyiodo)arenes via an in situ tethering strategy. This dianionic scaffold controls geometry in subsequent elementary steps and introduces a secondary coordination sphere proximate to the iodine(III) center, enhancing reactivity and selectivity beyond previous systems. The strategy demonstrates remarkable generality and robustness, enabling efficient access to diverse hypervalent iodine(III) reagents—including previously inaccessible examples—with operational simplicity and scalability. Its broad applicability is demonstrated through one‐pot transformations, including asymmetric organocatalysis, peptide modifications, and biocompatible aqueous reactions, offering enhanced performance and sustainability. Given its versatility, this platform holds significant potential for advancing both academic research and industrial applications in hypervalent iodine(III) chemistry.

## Conflict of Interest

The authors declare no conflict of interest.

## Supporting information



Supporting Information

## Data Availability

The data that support the findings of this study are available on request from the corresponding author. The data are not publicly available due to privacy or ethical restrictions.
