# Supplementary material for: Structurally Constrained Cyclic (Diacyloxyiodo)Arenes as an Enabling Platform for Hypervalent Iodine(III) Chemistry
Source: Adv Sci (Weinh). 2025 Jul 30;12(36):e06041. doi: 10.1002/advs.202506041 (PMC12463073; doi:10.1002/advs.202506041)
Supplement: Supplementary file 1 — Supporting Information [file ADVS-12-e06041-s001.pdf]

ADVANCED SCIENCE
Open Access

Supporting Information

for *Adv. Sci.*, DOI 10.1002/advs.202506041

Structurally Constrained Cyclic (Diacyloxyiodo)Arenes as an Enabling Platform for Hypervalent Iodine(III) Chemistry

Shengyu Zhong, Shaoyan Gan, Xin Zhang, Lijuan Song and Lei Shi**

Supporting Information

Structurally Constrained Cyclic (Diacyloxyiodo)arenes as an Enabling Platform for Hypervalent Iodine(III) Chemistry

Shengyu Zhong, Shaoyan Gan, Xin Zhang, Lijuan Song and Lei Shi**

Table of contents

1. General information 1

1.1. Computational methods 1

1.2. Reagents 1

1.3. Characterization 1

2. Experimental section 1

3. General procedure for the preparation of the 2,2'-diperoxyphenic acid 4

4. Reaction optimization 5

5. Procedure and results of sensitivity assessment 7

6. TGA–DSC analysis 9

7. General procedure for synthesis and applications of hypervalent iodine(III) species 10

7.1. General procedure: preparation of diphenyliodonium salts 3 10

7.2. General procedure: preparation of hypervalent iodine(III) reagents 3aa-3ae 11

7.3. Preparation of *ortho*-iodo diaryl ether 5 13

7.4. Preparation of indene 6 13

7.5. General procedure: preparation of α-diazoiodonium salts 8 13

7.6. Preparation of 10 14

7.7. Preparation of 12 14

7.8. Preparation of 14 15

7.9. Preparation of 15 15

8. The asymmetric dearomative spirolactonization of propanoic acid-substituted 1-naphthol 16 15

9. Preparation of 3-disubstituted indolin-2-ones 19 17

10. Preparation of chlorinated compounds 17

11. Preparation of fluorinated compounds 17

12. Structural evaluation and analysis of cyclic [bis(acyloxyl)iodo]arene species 21

13. The synthesis of DBD in aqueous solution 24

14. Biomolecule-compatibility experiments 25

14.1. Compatibility with biomolecules in 1 X PBS 25

14.2. The stability of nucleic acids in the oxidation 25

14.3. The activity of DNase I in the oxidation 26

14.4. The stability of proteins in the oxidation 27

14.5. Preparation of ethyl 2-diazo-2-(2-(dimethylamino)phenyl) acetate 12 27

15. Chemoselective labelling at methionine residues 27

15.1. Preparation of amino acid 33 and 34 27

15.2. Preparation of peptides 35 28

15.3. Preparation of peptides 38 29

16. Computational details 30

17 Supplemental references 73

18. Characterization of compounds 75

19 NMR spectra of compounds 92

1. General information

1.1. Computational methods

All calculations were performed with the Gaussian 16 package.[1] Unrestrained geometry were optimized using dispersion corrections in conjunction with B3LYP-D3[2–4] functional combining a basis set of SDD for I and 6-31G(d) for other atoms. Vibrational frequency analyses were used to ensure the identities of the local minimas (zero imaginary frequencies) and transition structures (one imaginary frequency). Single point calculations were performed at the M06-2X[5] level of theory with the aug-cc-PVTZ(-PP) basis set. Solvation energies were evaluated by a self-consistent reaction field (SCRF) using an SMD[6,7] implicit solvation model with solvent parameters chosen to represent dichloromethane. Intrinsic reaction coordinate (IRC) calculations[8–10] were carried out to confirm that the stationary points were smoothly connected to each other. The Gibbs energies were calculated at 298.15 K and 1 atm. Images of the optimized structures were prepared using CYLview.[11]

1.2. Reagents

Unless otherwise noted, all experiments were carried out under air atmosphere. Commercially available reagents, starting materials and solvents were used without further purification. All the solvents including *N, N*-dimethylformamide (DMF), 1,2-dimethoxyethane (DME), dimethyl sulfoxide (DMSO), 1,2-dichloroethane (DCE), dichloromethane (DCM), acetonitrile (MeCN), toluene (PhMe), tetrahydrofuran (THF), ethyl acetate (EA), petroleum ether (PE) and acetone were used as received without purification. Flash column chromatography was performed using 200-300 mesh silica gel. Schlenk tubes were purchased from Synthware.

1.3. Characterization

All reactions were monitored by TLC and visualized by UV lamp (254 nm). ^{1}H NMR (400 MHz or 600 MHz) and ^{13}C NMR (101MHz or 150 MHz) spectra were obtained on Bruker 400M or 600M or Zhongke-Niujin (Quantum-I Plus 400M) nuclear magnetic resonance spectrometers. For $CDCl_3$ solutions, the chemical shifts are reported as parts per million (ppm) referenced to residual protium or carbon of the solvents; $CHCl_3$ δ H (7.26 ppm) and $CDCl_3$ δ C (77.16 ppm). For DMSO-d_6 solutions, the chemical shifts are reported as parts per million (ppm) referenced to residual protium or carbon of the solvents; DMSO-d_6 δ H (2.5ppm) and DMSO-d_6 δ C(39.6ppm). Coupling constants(*J*) are reported in Hertz (Hz). Data for ^{1}H NMR and ^{13}CNMR spectra are reported as follows: chemical shift (ppm, referenced to protium: s = singlet, d = doublet, t = triplet, q = quartet, p = pentet (quintet), dd = doublet of doublets, td = triplet of doublets, ddd = doublet of doublet of doublets, m = multiplet, coupling constant (Hz), and integration). HRMS (ESI) was recorded using an Agilent 6520 accurate-Mass Q-TOF spectrometer. Yields of kinetic experiments were determined by LC-MS using the Agilent1260 Infinity IIG7129A/ G7115A/ G6125B or GC using Agilent7890B.

2. Experimental section

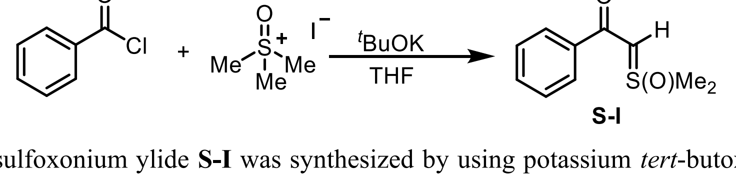

S-I

Compound sulfoxonium ylide **S-I** was synthesized by using potassium *tert*-butoxide (27.2 mmol, 3.0 g, 4.0 equiv.), trimethylsulfoxonium iodide (20.4 mmol, 4.48 g, 3.0 equiv.), and the corresponding

acyl chloride (6.8 mmol, 952.0 mg, 1.0 equiv.) following the standard procedure. The product, **S-I** was obtained as a white solid (1.2 g, 92% yield) through flash chromatography on silica gel using a mixture of methanol (MeOH) and ethyl acetate (EA) in a ratio of 1:15 (MeOH:EA). **^{1}H NMR** (400 MHz, Chloroform-*d*) δ 7.83-7.78 (m, 2H), 7.46-7.36 (m, 3H), 4.98 (s, 1H), 3.51 (s, 6H). The NMR spectra are consistent with the reported literature.[12]

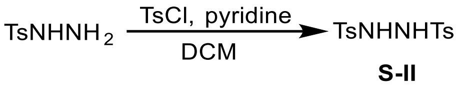

S-II

A flame-dried, 500-mL, round-bottomed flask equipped with a magnetic stir bar was charged with *p*-toluenesulfonyl hydrazide (9.32 g, 50.0 mmol) and *p*-toluenesulfonyl chloride (14.3 g, 75.0 mmol) in 50 mL of anhydrous CH_2Cl_2. The suspension was stirred at room temperature while pyridine (6.0 mL, 75.0 mmol) was added dropwise over 1 minute. During the addition, the reaction mixture became homogeneous and turned yellow. Within 3 minutes, a white precipitate was observed, and the reaction mixture was stirred for 1.5 hours. To the stirred mixture, Et_2O (200 mL) and H_2O (100 mL) were added and stirred at 0 °C for 15 minutes. The resulting white solid precipitate was collected in a Büchner funnel using suction filtration and washed with Et_2O (100 mL). The obtained solid was dissolved in boiling MeOH (100 mL). After cooling to room temperature, a precipitate formed. Approximately 100 mL of MeOH was removed by rotary evaporation and cooled to 0 °C. The resulting precipitate was collected in a Büchner funnel using suction filtration and washed with cold MeOH (20 mL) and Et_2O (100 mL) to obtain **S-II** (14.0 g, 82%). **^{1}H NMR** (DMSO-*d*$_6$, 400 MHz) δ 8.73 (s, 1H), 6.80 (d, *J* = 8.2Hz, 2H), 6.54 (d, *J* = 8.2 Hz, 2H), 1.56 (s, 3H); **^{13}C NMR** (DMSO-*d*$_6$, 100 MHz) δ 143.7, 135.7, 129.7, 128.0, 21.3; The NMR spectras are consistent with the reported literature.[13]

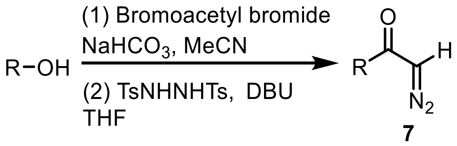

7

The corresponding alcohol (1.0 mmol) and $NaHCO_3$ (252 mg, 3.0 mmol) were dissolved in acetonitrile (5.0 mL), and bromoacetyl bromide (131 µL, 1.5 mmol) was slowly added at 0 °C. After stirring for 10 minutes at 0 °C, the reaction was quenched with water. The solution was extracted three times with CH_2Cl_2. The organic phase was washed with brine and dried over $MgSO_4$. The solvent was evaporated, and the residue was used in the subsequent reaction without purification. The obtained bromoacetate and *N, N'*-ditosylhydrazine (681 mg, 2.0 mmol) were dissolved in THF (5.0 mL) and cooled to 0 °C. DBU (750 mL, 5.0 mmol) was added dropwise and stirred at the same temperature for 10 minutes. After quenching the reaction with saturated $NaHCO_3$ solution, the mixture was extracted three times with Et_2O. The organic phase was washed with brine, dried over $MgSO_4$, and evaporated to yield the crude diazoacetate. The crude diazoacetate was purified using neutral silica gel, resulting in the products as a yellow oil.

Ethyl 2-diazoacetate **7a** was purchased from commercial sources. The diazoacetates **7o**, **7p**, **7q**, **7r**, **7s**, **7t**, **7u**, were known.[14-17]

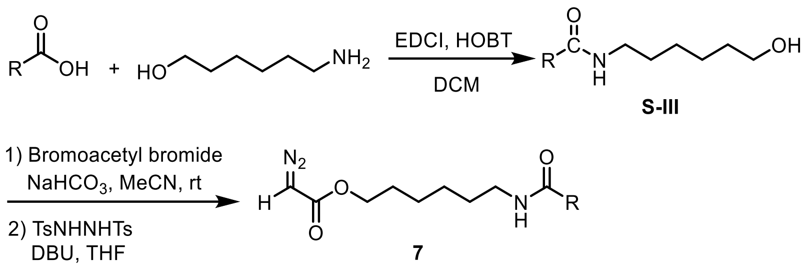

A round-bottomed flask equipped with a magnetic stirrer and a sidearm inlet adapter was charged with the corresponding acid (3.2 mmol) and *N*-hydroxybenzotriazole (379 mg, 3.2 mmol). The flask was evacuated and refilled with N_2. Methylene chloride (5 mL) was added to the flask, and the resulting solution was rapidly stirred and chilled in an ice bath. *N*-ethyl-*N'*-dimethylaminopropyl carbodiimide (680 mg, 3.6 mmol) was added in a single portion, and the mixture was stirred rapidly. After 7 minutes, the ice bath was removed, and the mixture was stirred for another 7 minutes. 6-amino-1-hexanol (529 mg, 3.9 mmol) in methylene chloride (6 mL) was added to the flask, and the resulting mixture was stirred at room temperature for 4 hours. The reaction was quenched with NH_4Cl, and the biphasic solution was transferred to a separatory funnel for phase separation. The aqueous layer was extracted once with methylene chloride. The combined organic layers were washed with 1 M HCl, $NaHCO_3$, and brine. After drying over $MgSO_4$, the mixture was filtered, and the solvent was removed using a rotary evaporator to obtain crude **S-III**. The resulting residue (1 mmol) and $NaHCO_3$ (252 mg, 3.0 mmol) were dissolved in acetonitrile (5.0 mL), and bromoacetyl bromide (131 μL, 1.5 mmol) was added slowly at 0 °C. After stirring for 10 minutes at 0 °C, the reaction was quenched with water. The solution was extracted three times with CH_2Cl_2. The organic phase was washed with brine and dried over $MgSO_4$. The solvent was evaporated, and the residue was used in the next reaction without purification. The obtained bromoacetate and *N, N'*-ditosylhydrazine (681 mg, 2.0 mmol) were dissolved in THF (5.0 mL) and cooled to 0 °C. DBU (750 mL, 5.0 mmol) was added dropwise and stirred at the same temperature for 10 minutes. After quenching the reaction with saturated $NaHCO_3$ solution, the mixture was extracted three times with Et_2O. The organic phase was washed with brine, dried over $MgSO_4$, and evaporated to yield the crude diazoacetate. The crude diazoacetate was purified using column chromatography, resulting in the products as a yellow solid.

The synthesis route refers to the reported literature.[15]

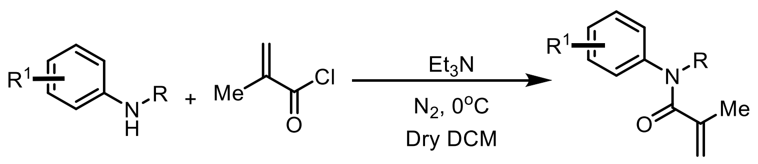

18

A solution of the corresponding aniline (1 equiv.) in DCM (60 mL) and triethylamine (2 equiv.) was added to a 250 mL round-bottom flask equipped with a magnetic stir bar. The mixture was stirred at 0 °C, and methacryloyl chloride (1.5 equiv.) was added under a nitrogen atmosphere. The resulting solution was allowed to warm up to room temperature and stirred for 6 hours. Then, H_2O (150 mL) was added to quench the excess acyl chloride. The mixture was transferred to a separation funnel, and the organic layer was extracted, washed with brine (3 x 100 mL), and dried over Na_2SO_4. The solvent was evaporated under reduced pressure, and the crude product was purified by column chromatography using a gradient of 0 to 6% ethyl acetate in hexanes.

The spectral data of the compounds **18a-e**, are in accordance with previous reports.[18]

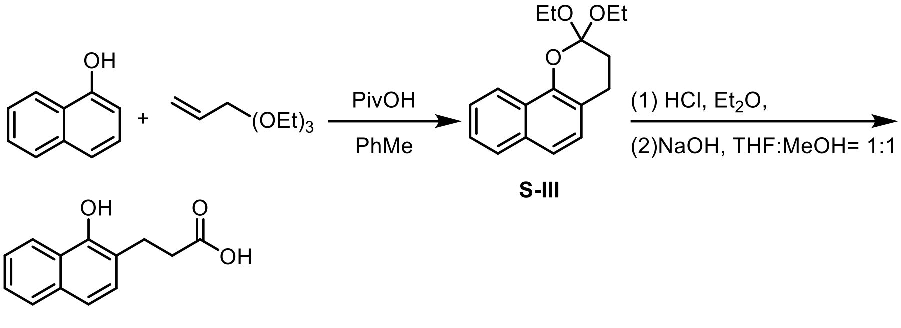

16

A solution of the appropriate 1-naphthol (1.0 equiv.) in dry toluene (0.3 M) was prepared in a flask. To this solution, pivalic acid (0.5 equiv.) and triethyl ortho acrylate (1.5 equiv.) were added, and the resulting mixture was refluxed for 24 hours. After refluxing, the reaction mixture was diluted with EtOAc, and the aqueous phase was extracted with EtOAc (three times) after washing with 1 M Na_2CO_3 (twice). The combined organic layers were washed with brine and dried over Na_2SO_4. The crude product was subjected to purification by passing through a short column packed with silica gel, using a gradient of cyclohexane and EtOAc as the eluent, to obtain the intermediate compound. The intermediate compound was dissolved in Et_2O (0.4 M), and 2 N HCl (1.3 mL/mmol) was added to the solution. The resulting mixture was stirred at room temperature overnight. Water was added, and the aqueous phase was extracted with EtOAc (three times). The combined organic layers were washed with brine, dried over Na_2SO_4, and the solvent was evaporated under reduced pressure. The crude product was dissolved in a 1:1 mixture of THF and MeOH (0.4 M), and 2 N NaOH (1.3 mL/mmol) was added. After stirring overnight at room temperature, the resulting mixture was acidified with 1 N HCl and extracted with EtOAc (three times). The combined organic layers were washed with brine, dried over Na_2SO_4, and the solvent was evaporated under reduced pressure. The crude product was purified by flash chromatography on silica gel, using a gradient of cyclohexane and EtOAc as the eluent. **^{1}H NMR** (400 MHz, Acetone-*d_6*) δ = 8.14- 8.12 (m, 1H), 7.92-7.90 (m, 1H), 7.67 (d, *J* = 8.3 Hz, 1H), 7.60 – 7.52 (m, 2H), 7.39 (d, *J* = 8.4 Hz, 1H), 3.76 (s, 1H), 3.23-3.19 (m, 2H), 2.94-2.91 (m, 2H). **^{13}C NMR** (101 MHz, Methanol-*d_4*) δ = 184.1, 162.6, 136.0, 131.1, 130.0, 128.0, 125.1, 124.7, 123.5, 122.8, 112.7, 40.4, 29.5.
The physical and spectroscopic data was in full accordance with the data reported in literature.[19]

3. General procedure for the preparation of the 2,2'-diperoxyphenic acid

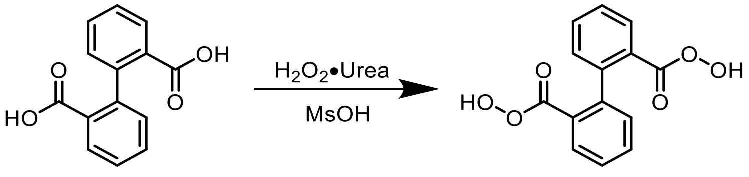

In a round-bottomed flask equipped with a large magnetic stir bar, methane sulfonic acid (30 mL) was placed and the flask was immersed in a water bath at 22 °C. Urea hydrogen peroxide (9.82 g, 104 mmol) was added in a single portion and stirred for 30 seconds. Then, diphenic acid (35 mmol) was added in a single portion, and the reaction was vigorously stirred for 24 hours. After completion of the reaction, the reaction mixture was poured into a mixture of ice (80 g) and ethyl acetate (100 mL), and the layers were separated. The aqueous layer was further extracted with ethyl acetate (2 × 100 mL), and

the combined organic layers were washed with $NaHCO_3$ (2 × 50 mL), brine (20 mL), and dried over Na_2SO_4. The solvent was removed under reduced pressure, resulting in the formation of 2,2'-diperoxyphenic acid. **^{1}H NMR** (400MHz, Chloroform-*d*) δ 11.28 (s, 2H), 8.03 (dd, *J* = 7.9, 1.3 Hz, 2H), 7.70 (td, *J* = 7.6,1.4 Hz, 2H), 7.58 (td, *J* = 7.7, 1.3 Hz, 2H), 7.32 (dd, *J* = 7.7, 1.3 Hz, 2H). **^{13}C NMR** (101 MHz, Chloroform-*d*) δ 168.3, 142.1, 133.3, 130.9, 129.9, 128.39, 124.69.
The physical and spectroscopic data was in full accordance with the data reported in literature.[20]

4. Reaction optimization

Table S1. Screening of oxidants in the synthesis of diaryliodonium salts[a]

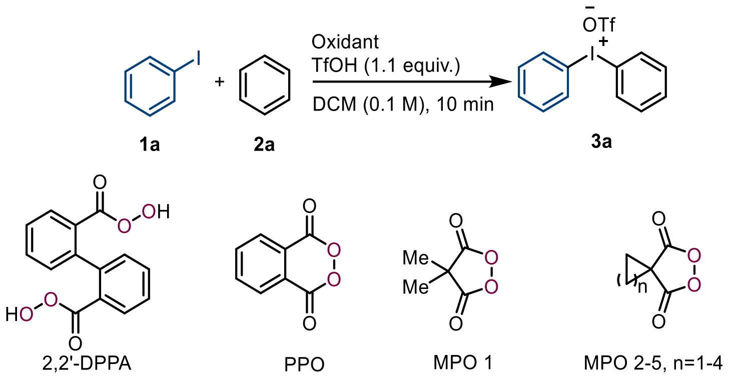

Entry	Oxidant (equiv.)	Yield[b] (%)
1	2,2'-DPPA (0.6)	51
2	PPO (1.2)	N.D.
3	MPO-1 (1.2)	N.D.
4	MPO-2 (1.2)	N.D.
5	MPO-3 (1.2)	N.D.
6	MPO-4 (1.2)	N.D.
7	MPO-5 (1.2)	N.D.
8	2,2'-DPPA (0.75)	**70**

[a]The reactions were performed with **1a** (0.3 mmol, 1.0 equiv.), **2a** (0.6 mmol, 2 equiv.), oxidant and TfOH (0.33 mmol, 1.1 equiv.) in DCM (3 mL) for 10 min. [b]Isolated yields.

.

Table S2. Screening of reaction times[a]

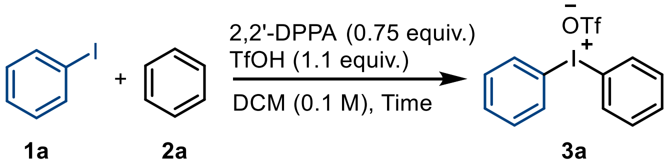

Entry	Time	Yield[b] (%)
1	1 min	< 5
2	5 min	20
3	10 min	**70**
4	30 min	62

5	1 h	60

[a]The reactions were performed with **1a** (0.3 mmol, 1.0 equiv.), **2a** (0.6 mmol, 2 equiv.), 2,2'-DPPA (0.23 mmol, 0.75 equiv.) and TfOH (0.33 mmol, 1.1 equiv.) in DCM (3 mL) at indicated time. [b]Isolated yields.

Table S3. Screening of equivalents of TfOH[a]

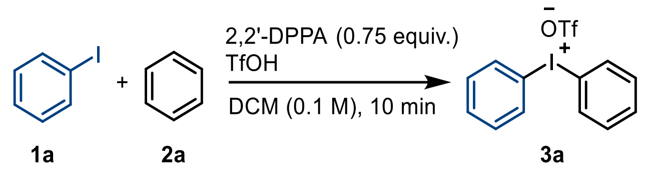

Entry	TfOH (equiv.)	Yield[b] (%)
1	1	65
2	1.1	68
3	1.5	**84**
4	2	80

[a]The reactions were performed with **1a** (0.3 mmol, 1.0 equiv.), **2a** (0.6 mmol, 2 equiv.), 2,2'-DPPA (0.23 mmol, 0.75 equiv.) and TfOH in DCM (3 mL) for 10 min. [b]Isolated yields.

Table S4. Screening of oxidants in the synthesis of α-diazoiodonium salt[a]

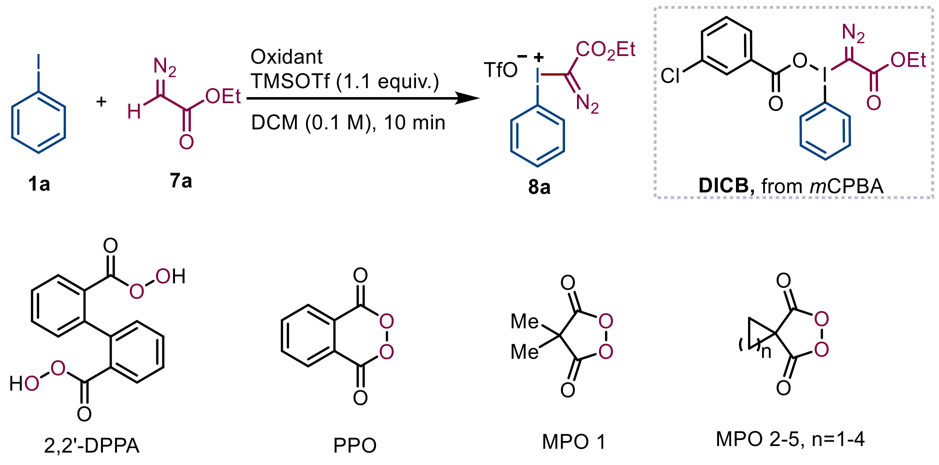

Entry	Oxidant (equiv.)	Yield[b] (%)
1	2,2'-DPPA (0.55)	55
2	PPO (1.1)	N.D.
3	MPO-1 (1.1)	N.D.
4	MPO-2 (1.1)	N.D.
5	MPO-3 (1.1)	N.D.
6	MPO-4 (1.1)	N.D.
7	MPO-5 (1.1)	N.D.
8	*m*CPBA (1.1)	N.D.
9	2,2'-DPPA (0.7)	**58**

[a]The reactions were performed with **1a** (0.3 mmol, 1.0 equiv.), **7a** (0.33 mmol, 1.1 equiv.), oxidants and

TMSOTf (0.33 mmol, 1.1 equiv.) in DCM (3 mL) for 10 min. [b]Isolated yields.

Table S5. Screening of reaction times[a]

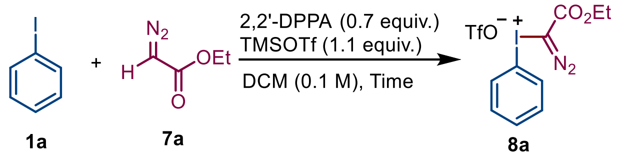

Entry	Time	Yield[b] (%)
1	1 min	< 5
2	5 min	35
3	10 min	58
4	30 min	**65**
5	1 h	62

[a]The reactions were performed with **1a** (0.3 mmol, 1.0 equiv.), **7a** (0.33 mmol, 1.1 equiv.), 2,2'-DPPA (0.21mmol, 0.7 equiv.) and TMSOTf (0.33 mmol, 1.1 equiv.) in DCM (3 mL) at indicated time. [b]Isolated yields.

Table S6. Screening of equivalent of **7a**[a]

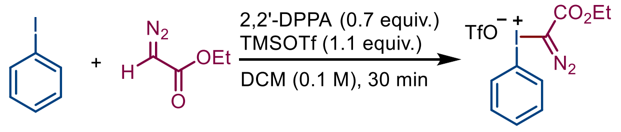

Entry	**7a** (equiv.)	Yield[b] (%)
1	1	58
2	1.1	65
3	1.5	77
4	2	**87**
5	3	80

[a]The reactions were performed with **1a** (0.3 mmol, 1.0 equiv.), **7a**, 2,2'-DPPA (0.21 mmol, 0.7 equiv.) and TMSOTf (0.33 mmol, 1.1 equiv.) in DCM (3 mL) for 30 min. [b]Isolated yields.

5. Procedure and results of sensitivity assessment

Table S7. General description of experiments included in one sensitivity assessment

Entry	Experiment	Description
1	High concentration	2.7 mL of solvent
2	Low concentration	3.3 mL of solvent
3	High H_2O	30 μL of H_2O
4	Low O_2	N_2
5	High O_2	O_2
6	Low temperature	35 °C
7	High temperature	15 °C

8	Low light intensity	dark
9	High light intensity	Blue LED
10	Gram scale	**1a** (6mmol, 0.67 mL)

Table S8. Outcome of sensitivity assessment of diaryliodonium salt

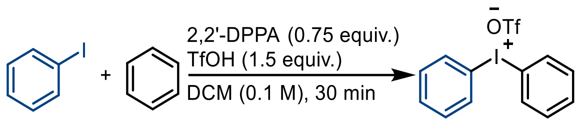

Entry	Parameters	Yield/%	Deviation/%
1	High concentration	82	- 2
2	Low concentration	83	-1
3	High H_2O	66.5	-17.5
4	Low O_2	83	-1
5	High O_2	82	-2
6	Low temperature	83.5	-0.5
7	High temperature	83	-1
8	Low light intensity (dark)	83.5	-0.5
9	Blue LED	78	-6
10	Control	84	
11	Gram scale	79.5	-4.5

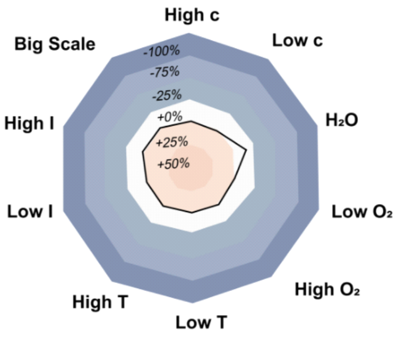

Figure S1. Radar diagram for sensitivity assessment of diaryliodonium salts.

Table S9. General description of experiments included in one sensitivity assessment

Entry	Experiment	Description
1	High concentration	2.7 mL of solvent
2	Low concentration	3.3 mL of solvent
3	High H_2O	30 μL of H_2O
4	Low O_2	N_2
5	High O_2	O_2
6	Low temperature	35 ℃

7	High temperature	15 °C
8	Low light intensity	dark
9	High light intensity	Blue LED
10	Gram scale	**1a** (6mmol, 0.67 mL)

Table S10. Outcome of sensitivity assessment of α-diazoiodonium salt

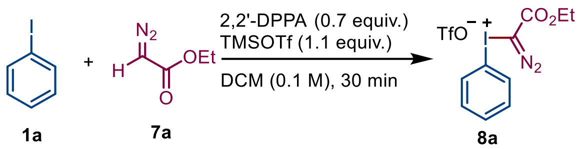

Number	Parameters	Yield/%	Deviation/%
1	High concentration	82	-5
2	Low concentration	81	-6
3	High H_2O	31.5	-55.5
4	Low O_2	85	-2
5	High O_2	82.5	-4.5
6	Low temperature	81.5	-5.5
7	High temperature	84.5	-2.5
8	Low light intensity (dark)	84	-3
9	Blue LED	80.5	-6.5
10	Control	87	
11	Gram scale	81	-6

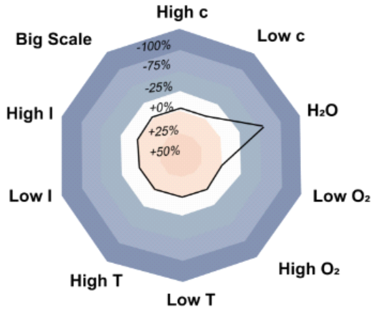

Figure S2. Radar diagram for sensitivity assessment of α-diazoiodonium salt.

6. TGA–DSC analysis

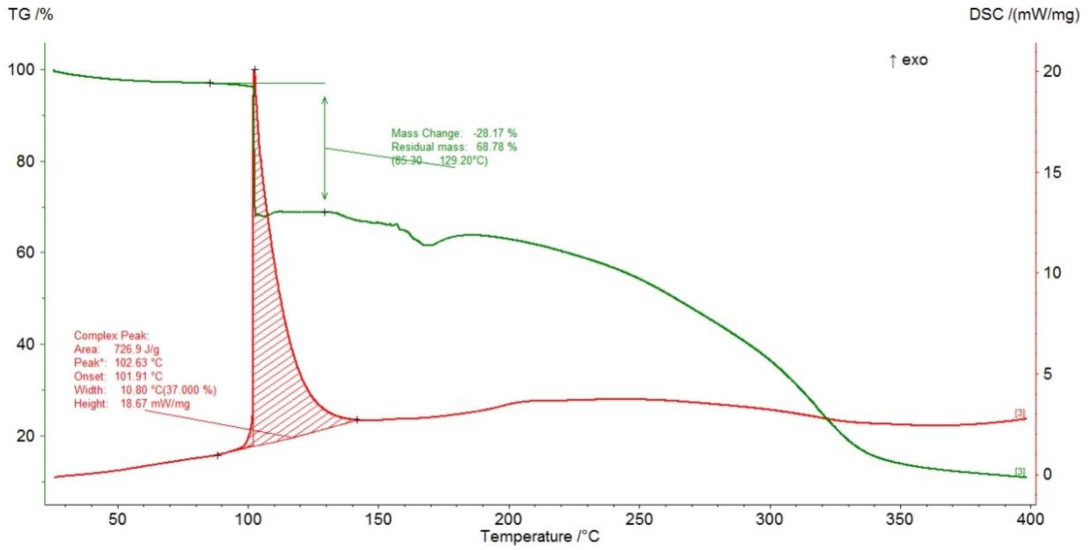

Figure S3. TGA–DSC of 2,2’-DPPA.

7. General procedure for synthesis and applications of hypervalent iodine(III) species

7.1. General procedure: preparation of diphenyliodonium salts 3

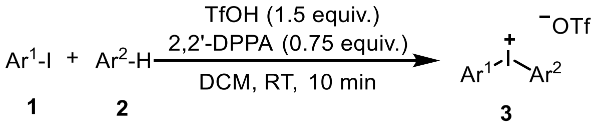

A dry round-bottom flask, flushed with argon and equipped with a magnetic stirrer bar and a septum was prepared. Aryl iodide **1** (0.3 mmol) and DCM (3 mL) were charged into the flask, followed by the addition of 2,2'-DPPA (0.23 mmol). The reaction mixture was allowed to react at room temperature for 5 minutes. Then, arene **2** (0.6 mmol) was added, and the solution was cooled to the specified temperature. TfOH (0.45 mmol) was added dropwise, resulting in a slight increase in temperature and a change in color from a clear solution to a yellow transparent solution. The solution was stirred at the specified temperature and time and then concentrated under vacuum while keeping it cold. Next, Et_2O (1-2 mL) was added, and the mixture was stirred at room temperature for 10 minutes to precipitate an off-white solid. The flask was stored in the freezer for 30 minutes, after which the solid was filtered off, washed with cold Et_2O, and dried under vacuum to obtain diaryliodonium salt **3**.

The physical and spectroscopic data were in full accordance with the data reported in literature.[21,22]

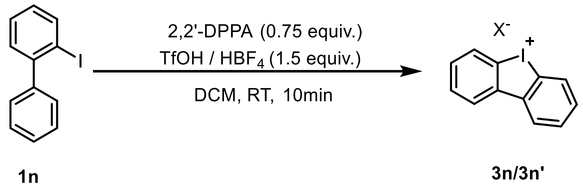

A dry round-bottom flask, flushed with argon and equipped with a magnetic stirrer bar and a septum, was prepared. 2-iodo-1,1'-biphenyl **1n** (0.3 mmol) and 2,2’-DPPA (0.23 mmol) were added to the flask containing DCM (3 mL). TfOH or HBF_4 (0.45 mmol) was slowly added over a period of 20 minutes at 0 °C. The reaction mixture was then brought to room temperature and stirred for 10 min. It was subsequently concentrated using a rotary evaporator. The resulting residue was triturated with Et_2O (1-2

mL) for 30 minutes and filtered. The collected solid was washed with Et_2O (1-2 mL) and dried, yielding **3n** or **3n’** as a yellow solid. When the reaction time is extended to 30 minutes, **3n**/**3n'** can be obtained in a yield of 97%/98%.
The physical and spectroscopic data were in full accordance with the data reported in literature.[23]

7.2. General procedure: preparation of hypervalent iodine(III) reagents 3aa-3ae

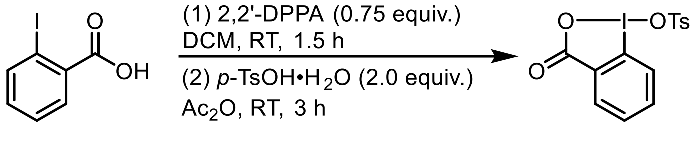

1aa **3aa**

2,2-DPPA (3 mmol, 0.75 equiv.) was added to an oven-dried flask containing a suspension of 2-iodobenzoic acid (**1aa**) (4 mmol, 1.0 equiv.) in dry DCM (12 mL). After 5 minutes, a white solid precipitated. The reaction mixture was stirred at room temperature under N_2 for 1.5 hours. Then, acetic anhydride (4 mL) was added, followed by the gradual addition of *p*-TsOH•H_2O (8 mmol, 2.0 equiv.). After 5 minutes, a slightly exothermic reaction started, and the mixture turned into a clear, slightly yellow solution. The reaction was stirred at room temperature under N_2 for 3 hours. The DCM solvent was removed under vacuum, and dry Et_2O (40 mL) was added. The mixture was cooled to 0 °C for 10 minutes, expecting precipitation of the product. The resulting solid was filtered, washed with dry Et_2O (4 x 20 mL), and dried under vacuum to obtain 1-(*p*-methylbenzenesulfonyloxy)-1,2-benziodoxol-3-(1H)-one (**3aa**) as a white solid.
The physical and spectroscopic data were in full accordance with the data reported in literature.[24]

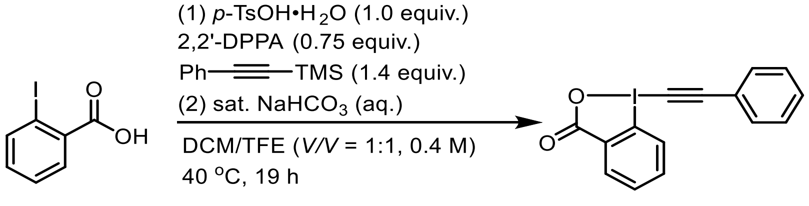

1aa **3ab**

2-iodobenzoic acid **1aa** (4 mmol, 1.0 equiv.), *para*-toluenesulfonic acid monohydrate (1.0 equiv.), and 2,2'-DPPA (0.75 equiv.) were dissolved in a mixture of dichloroethane and 2,2,2-trifluoroethanol (0.4 M, v/v = 1:1). After stirring for 1 hour at 40 °C, the corresponding alkynyl trimethylsilane species (1.4 equiv.) was added in one portion. The reaction mixture was stirred for an additional 18 hours at the same temperature. Then, the resulting suspension was filtered, and the volatile components were removed under reduced pressure. The resulting residue was dissolved in dichloromethane and treated with a solution of saturated aqueous sodium bicarbonate. The mixture was vigorously stirred for 1 hour, and then the two layers were separated. The aqueous layer was further extracted with additional portions of dichloromethane. The organic layers were combined, dried over magnesium sulfate, filtered, and concentrated under reduced pressure. Purification was performed either by column chromatography or by recrystallization in MeCN.
The physical and spectroscopic data was in full accordance with the data reported in literature.[25]

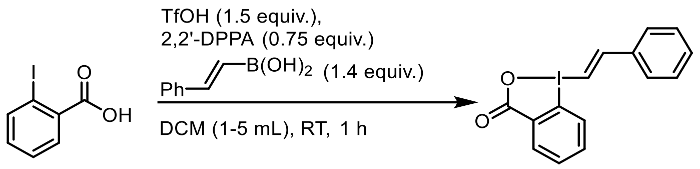

1aa **3ac**

2-iodobenzoic acid **1aa** (4 mmol, 1 equiv.) was added to a round-bottom flask, followed by the addition of DCM. 2,2'-DPPA (0.75 equiv.) was added, and the mixture was cooled to 0 °C. Then, TfOH (1.5 equiv.) was added. The mixture was stirred at room temperature for 15 minutes and then cooled to 0 °C for 5 minutes. The corresponding boronic acid (1.4 equiv.) was added in one portion and rinsed down with DCM (1-5 mL). The mixture was stirred at room temperature for 1 hour. Saturated $NaHCO_3$ solution was added, and the mixture was vigorously stirred at room temperature for 1 hour. The reaction mixture was then transferred to a separation funnel and diluted with DCM and water. Note: Dilution helped to prevent emulsions during separation. The layers were separated, and the aqueous phase was extracted three times with DCM. The combined organic phases were washed with water and brine, and then dried over Na_2SO_4. The drying agent was filtered off, and the solvent was removed under vacuum. Et_2O was added to the resulting white precipitate, and the mixture was vigorously stirred at room temperature for approximately 30 minutes. The solid was filtered off using a glass filter funnel with porosity and washed with Et_2O to obtain **3ac**.
The physical and spectroscopic data was in full accordance with the data reported in literature.[26]

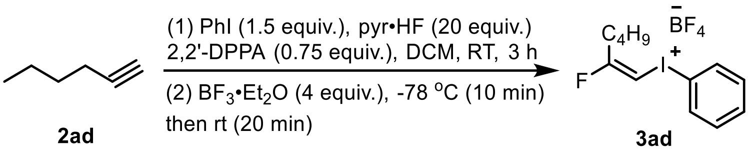

2ad **3ad**

In a teflon tube, PhI (0.75 mmol), Pyr·HF (10 mmol), 2,2'-DPPA (0.38 mmol), and CH_2Cl_2 (2 mL) were placed. The mixture was stirred at room temperature for 3 hours, and then alkyne **2ad** (0.5 mmol) was added at the same temperature. After cooling the mixture to -78 °C, $BF_3{\cdot}Et_2O$ (2.0 mmol) was added, and the mixture was stirred for 10 minutes. The reaction mixture was then warmed to room temperature and stirred for 20 minutes. The mixture was poured into water (15 mL) and extracted with CH_2Cl_2 (10 mL × 3). The combined organic layer was washed with an aqueous solution (15 mL) of $NaBF_4$ (5.0 mmol) and dried over anhydrous Na_2SO_4. After evaporating the solvent, the residue was subjected to column chromatography on silica gel.
The physical and spectroscopic data was in full accordance with the data reported in literature.[27]

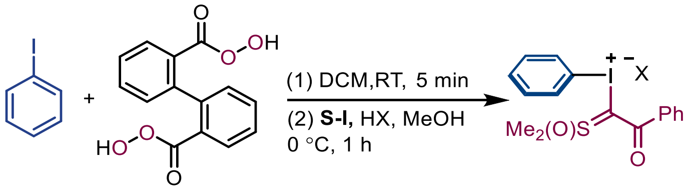

1a 2,2'-DPPA **3ae** and **3ae'**

A dry round-bottom flask, flushed with argon and equipped with a magnetic stirrer bar and a septum, was charged with aryl iodide **1a** (5 mmol) and DCM (10 mL). 2,2'-DPPA (3.8 mmol) was added, and the reaction mixture was stirred at room temperature for 5 minutes. The solvent was then removed under

reduced pressure. The resulting residue was dissolved in MeOH (5.0 mL, 1.0 M) and treated with the corresponding acid HX (5.0 mmol, 1.0 equiv.) at room temperature. The clear solution was added dropwise to an ice bath-cooled solution of sulfoxonium ylides **S-I** (5.0 mmol) in MeOH (5.0 mL, 1.0 M) over 10 minutes with stirring. The resulting reaction mixture was stirred at 0 °C for an additional 1 hour. During this period, a significant amount of white precipitate was formed. The product was collected by filtration, washed successively with MeOH (5 mL × 3) and Et_2O (5 mL × 3), dried under high vacuum, and stored at -30 °C. If the hypervalent iodine reagent failed to precipitate, it was subjected to flash column chromatography, eluting with DCM/Acetone mixtures.
The physical and spectroscopic data were in full accordance with the data reported in literature.[28]

7.3. Preparation of *ortho*-iodo diaryl ether 5

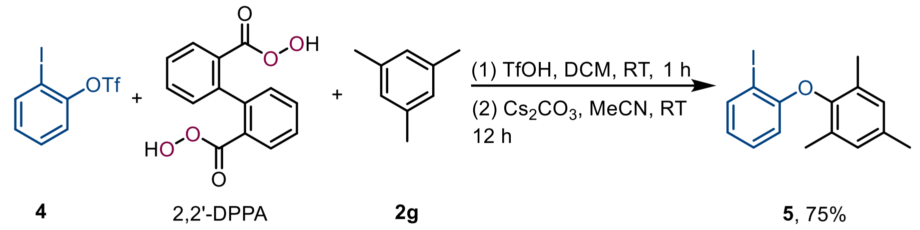

2-iodophenyl trifluoromethanesulfonate **4** (1.0 mmol) and 2,2'-DPPA (0.75 equiv.) were dissolved in CH_2Cl_2 (10 mL) in a round-bottom flask. Then mesitylene **2g** (2 equiv.) was added and the solution was cooled to 0 ºC followed by dropwise addition of TfOH (1.5 equiv.), resulting in a coloured solution. The reaction mixture was stirred at room temperature for 1 h and subsequently concentrated under vacuum. The resultant residue was dissolved in MeCN (0.1 M), Cs_2CO_3 (1.2 equiv.) was added, the tube was degassed with argon for three times, the mixture was stirred at 25 ºC. After TLC indicated that the iodonium salts were completely consumed, the solvent was evaporated under vacuum. The crude products were purified using flash column chromatography on silica gel to afford the desired product **5**. The physical and spectroscopic data was in full accordance with the data reported in literature.[29]

7.4. Preparation of indene 6

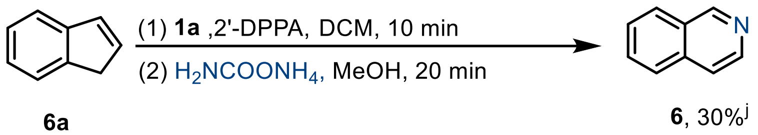

A dry round bottom flask flushed with argon and equipped with a magnetic stirrer bar and a septum was charged with aryl iodide **1a** (0.2 mmol), DCM (3 mL) and 2,2'-DPPA (0.23 mmol) was added. The reaction mixture was reacted at room temperature for 10 minutes and subsequently concentrated under vacuum. The resultant residue was dissolved in MeOH (3 mL), indene (0.2 mmol, 1.0 equiv.) and ammonium carbamate (0.8 mmol, 4.0 equiv.) were added at 0 °C. The reaction mixture was stirred for 20 minutes at 0 °C. The ice bath was removed, and the mixture was warmed to room temperature. The reaction progress was monitored by TLC.
The physical and spectroscopic data was in full accordance with the data reported in literature.[30]

7.5. General procedure: preparation of α-diazoiodonium salts 8

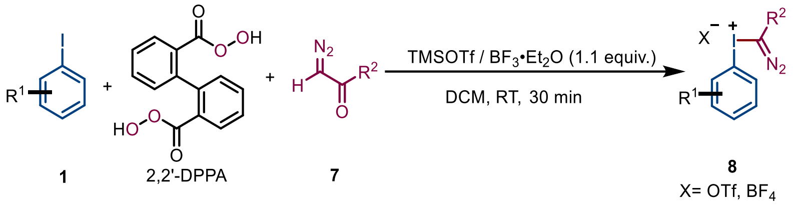

A dry round-bottom flask, flushed with argon and equipped with a magnetic stirrer bar and a septum, was charged with aryl iodide **1** (0.3 mmol), DCM (3 mL), and 2,2'-DPPA (0.21 mmol). The reaction mixture was stirred at room temperature for 5 minutes. The resulting residue was then treated with trimethylsilyl trifluoromethanesulfonate or $BF_3 \cdot Et_2O$ (0.33 mmol) at room temperature. Afterward, the corresponding diazo compound **7** (0.6 mmol) was added dropwise over a period of 10 minutes. Nitrogen evolution was observed, and the resulting yellow reaction mixture was stirred for 30 minutes at room temperature. The solvent was removed under vacuum, and the crude product was recrystallized from a mixture of diethyl ether/dichloromethane (5/1) at -30 °C for 12 hours. The product was collected by filtration, washed with cold diethyl ether, dried under high vacuum, and stored at -30 °C.
The physical and spectroscopic data were in full accordance with the data reported in literature.[15,16]

7.6. Preparation of 10

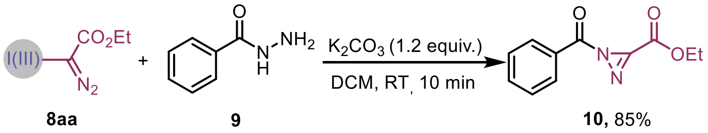

At First a round bottom flask was placed in Emerson cooler and the temperature was maintained at 0°C. Next, the carbohydrazide **9** (0.25 mmol, 1.0 equiv.), hypervalent iodine reagent **8aa** (0.3 mmol, 1.2 equiv.) and K_2CO_3 (0.3 mmol, 1.2 equiv.) were taken in RBF. Thereafter, 3 mL of DCM was added with syringe and the reaction mixture was transferred at room temperature and stirred for 10 minutes in open air. Next, the reaction mixture was passed through a short plug of silica gel and washed with dichloromethane (3 x 10 mL). The solvent was removed under vacuum and the residue was purified by column chromatography to give **10**.
The physical and spectroscopic data was in full accordance with the data reported in literature.[31]

7.7. Preparation of 12

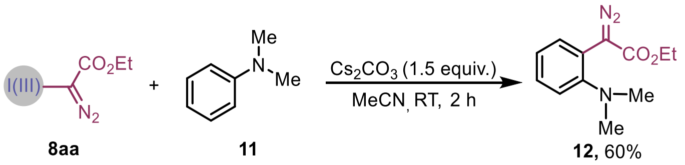

A mixture of **11** (1.0 mmol), Hypervalent iodine(III) reagent **8aa** (2.5 mmol), Cs_2CO_3 (1.5 mmol) were added to a 25-mL Schlenk tube in nitrogen atmosphere condition. Thereafter, acetonitrile (5 mL) was added with syringe and the reaction mixture was stirred at r.t for 2 h. The progress of the reaction was monitored by TLC. The product **12** was isolated by direct flash column chromatography of the crude reaction
The physical and spectroscopic data was in full accordance with the data reported in literature.[32]

7.8. Preparation of 14

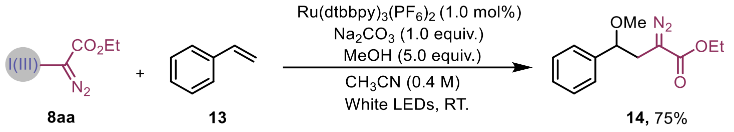

To an oven-dried 10 mL screw-capped vial equipped with a stirring bar was added ruthenium photocatalyst (0.002 mmol, 1 mol%), reagent **8aa** (0.2 mmol, 1.0 equiv) and Na_2CO_3 (21.2 mg, 0.2 mmol, 1.0 equiv). The tube was sealed before being evacuated and backfilled with argon 3 times. After that, the corresponding alkene **13** (0.6 mmol, 3 equiv), degassed MeCN (0.5 mL) and the MeOH (1.0 mmol, 5.0 equiv) were added. The reaction tube was placed in the photoreactor at 0 ºC and after 2 hours, the resulting reaction mixture was passed through a short pad of silica gel and washed with dichloromethane (5.0 mL). The solvent was removed under vacuum and the residue was purified by flash column chromatography on silica gel to give the corresponding β-alkoxydiazo compounds **14**.

The physical and spectroscopic data was in full accordance with the data reported in literature.[33]

7.9. Preparation of 15

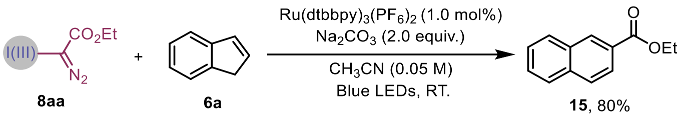

In an oven-dried 10 ml Schlenk tube equipped with a PTFE-coated rare-earth “extra power" oval stirring bar, $Ru(dtbbpy)_3(PF_6)_2$ (1 mol%, 2.4 mg), hypervalent iodine reagents **8aa** (0.24 mmol, 1.2 equiv.) and Na_2CO_3 (0.4 mmol, 2 equiv.) were charged under air, then the vessel was evacuated and re-filled with argon for four times. Dry acetonitrile (4.0 mL, 0.05 M) and indene **6a** (0.2 mmol, 1.0 equiv.) were added under argon counter flow. The reaction tube was placed in the photoreactor at 0 ºC and after 2 hours, the resulting reaction mixture was passed through a short pad of silica gel and washed with dichloromethane (5.0 mL). Purification by flash column chromatography on SiO_2, using pentane/EtOAc mixtures afforded the corresponding product **15**.

The physical and spectroscopic data were in full accordance with the data reported in literature.[17]

8. The asymmetric dearomative spirolactonization of propanoic acid-substituted 1-naphthol 16

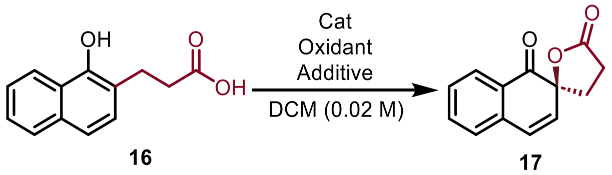

A solution of **16** (10.8 mg, 0.05 mmol), **Cat 2** (0.005 mmol, 10 mol%), AcOH (0.05mmol, 1 eq) and *m*CPBA (14.6 mg, 0.065 mmol, 1.3 eq) or 2,2’-DPPA (8.2 mg, 0.03 mmol, 0.6 eq) in CH_2Cl_2 (2.5 mL) was stirred at -20 °C. After 18h, the resulting mixture poured into aqueous $Na_2S_2O_3$ (5 mL) and aqueous $NaHCO_3$, and extracted with $CHCl_3$ (2 times). The organic layers were dried over anhydrous $MgSO_4$ and solvents were removed in vacuo. The residue was purified by flash column chromatography on silica gel (eluent: hexane-EtOAc = 10:1 to 4:1) to give **17** (7.8 mg, 0.035 mmol) in 70% yield. White solid; TLC, R_f = 0.46 (hexane–EtOAc–$CHCl_3$ = 1:2:1); **^{1}H NMR** (Chloroform-*d*, 400 MHz) 2.18 (ddd,

J = 9.6, 11.0, 13.5 Hz, 1H), 2.49 (ddd, J = 1.8, 9.6, 13.5 Hz, 1H), 2.60 (ddd, J = 1.8, 9.6, 17.6 Hz, 1H), 2.92 (ddd, J = 9.6, 11.0, 17.6 Hz, 1H), 6.21 (d, J = 10.4 Hz, 1H), 6.66 (d, J = 10.4 Hz, 1H), 7.26 (d, J = 8.0 Hz, 1H), 7.41 (t, J = 8.0 Hz, 1H), 7.62 (t, J = 8.0 Hz, 1H) , 8.02 (d, J = 8.0 Hz, 1H); **^{13}C NMR** (Chloroform-*d*, 100 MHz) 26.5, 31.2, 83.4, 127.3, 127.8, 127.9, 127.9, 129.0, 132.3, 135.7, 136.8, 176.5, 196.5; HPLC (OD-H column), Hexane : *i*PrOH = 85:15 as eluent, 1.0 mL/min, t_R = 16.2 min, t_S = 21.3 min. The physical and spectroscopic data was in full accordance with the data reported in literature.[19]

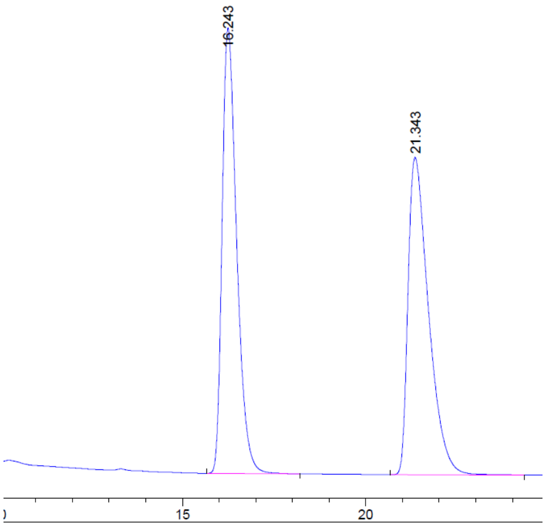

信号 1: VWD1 A, Wavelength=230 nm

峰 #	保留时间 [min]	类型	峰宽 [min]	峰面积 [mAU*s]	峰高 [mAU]	峰面积 %
1	16.243	BB	0.4228	1.73066e4	627.35065	49.9504
2	21.343	BB	0.5888	1.73410e4	446.81311	50.0496

Figure S4. Chiral HPLC spectrum of (rac)-**17**.

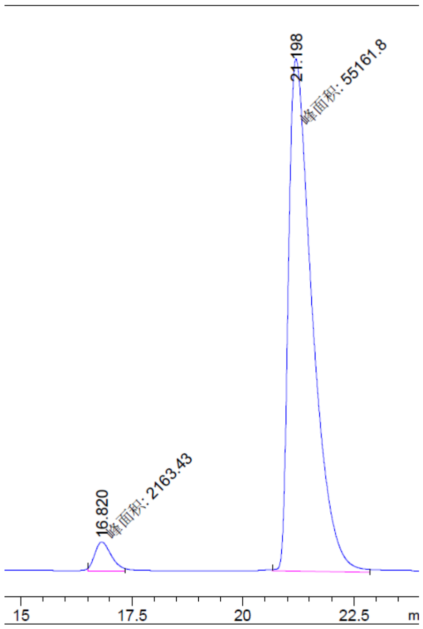

信号 1: VWD1 A, Wavelength=230 nm

峰 #	保留时间 [min]	类型	峰宽 [min]	峰面积 [mAU*s]	峰高 [mAU]	峰面积 %
1	16.820	MM	0.4315	2163.42920	83.56776	3.7740
2	21.198	MM	0.6203	5.51618e4	1482.13940	96.2260

Figure S5. Chiral HPLC spectrum of **17** when 2,2'-DPPA as oxidant.

9. Preparation of 3-disubstituted indolin-2-ones 19

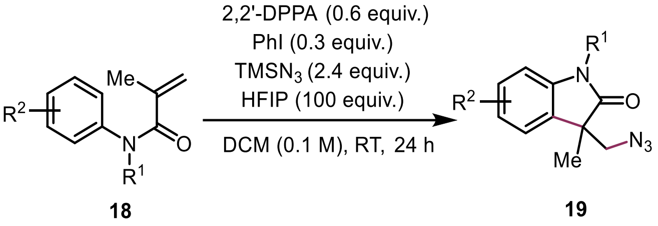

A dry round bottom flask flushed with argon and equipped with a magnetic stirrer bar and a septum was charged with aryl iodide **1a** (0.06 mmol), DCM (2 mL), *N*-arylacrylamide **18** (0.2 mmol), HFIP (20 mmol), and $TMSN_3$ (0.48 mmol). The 2,2'-DPPA (0.12 mmol) was added portion-wise at room temperature. After stirring for 24 h at room temperature, the reaction mixture was diluted with diethyl ether (10 mL) and washed with water (1 mL). The organic layer was dried on $MgSO_4$ and solvents were removed under reduced pressure. Column chromatography of the crude mass provided pure product **19**. The physical and spectroscopic data were in full accordance with the data reported in literature.[18]

10. Preparation of chlorinated compounds

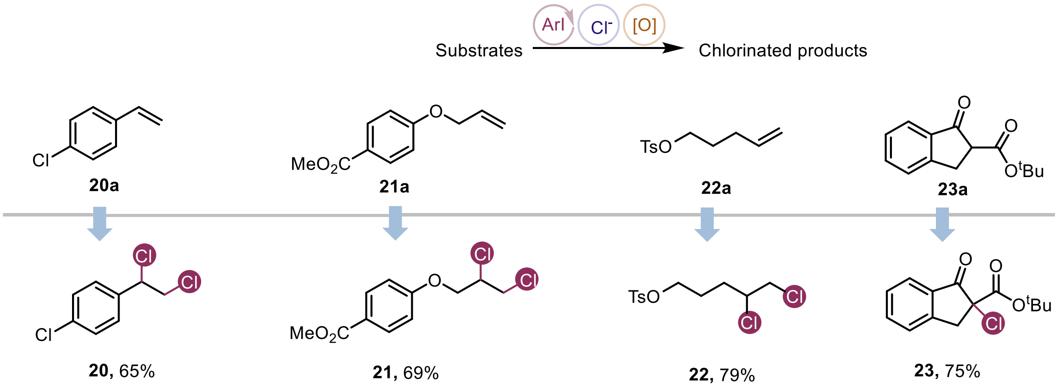

The corresponding substrates (0.20 mmol, 1.0 equiv.), *p*-iodotoluene (8.7 mg, 0.04 mmol, 0.2 equiv.) TMSCl (0.60 mmol, 3.0 equiv.) and 2,2'-DPPA (0.12 mmol, 0.6 equiv.) were dissolved in a solution of HFIP (302 mg, 1.80 mmol, 9.0 equiv.) in CH_2Cl_2 (0.9 mL) and stirred at ambient temperature. After 8 h, the reaction mixture was quenched with a saturated solution of $Na_2S_2O_3$ and extracted with CH_2Cl_2 (3 x). The residue was purified by flash column chromatography on silica gel to give products.
The physical and spectroscopic data were in full accordance with the data reported in literature.[34-36]

11. Preparation of fluorinated compounds

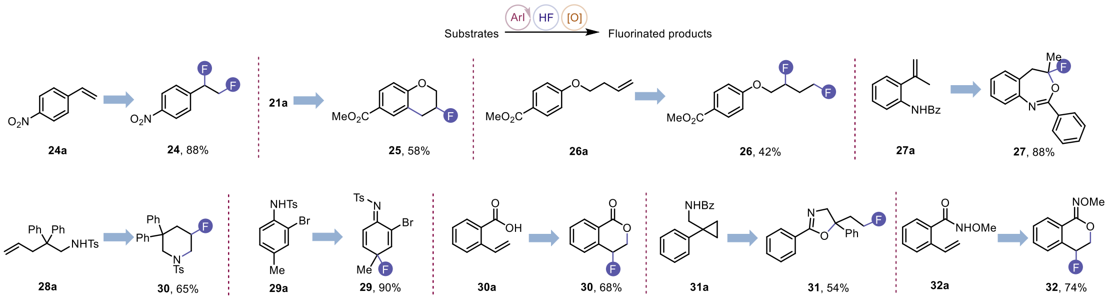

The corresponding substrates (**27a**, **28a**, **30a**, **32a**) (0.20 mmol, 1.0 equiv.), *p*-iodotoluene (8.7 mg, 0.04 mmol, 0.2 equiv.), pyr-9HF (0.14 mL, 100.0 equiv. HF) and 2,2'-DPPA (0.12 mmol, 0.6 equiv.) were dissolved in CH_2Cl_2 (0.5 mL) and stirred at ambient temperature. After 1 - 12 h, the reaction mixture was quenched with a saturated solution of $NaHCO_3$ and extracted with CH_2Cl_2 (3 x). The residue was purified by flash column chromatography on silica gel to give products.

The corresponding substrates(**24a**, **21a**, **26a**, **29a, 31a**) (0.20 mmol, 1.0 equiv.), *p*-iodotoluene (8.7 mg, 0.04 mmol, 0.2 equiv.), amine:HF = 1:5.5 (0.5 mL) and 2,2'-DPPA (0.12 mmol, 0.6 equiv.) were dissolved in CH_2Cl_2 (0.5 mL) and stirred at ambient temperature. After 1 - 12 h, the reaction mixture was quenched with a saturated solution of $NaHCO_3$ and extracted with CH_2Cl_2 (3 x). The residue was purified by flash column chromatography on silica gel to give products.
The physical and spectroscopic data was in full accordance with the data reported in literature.[37-41]

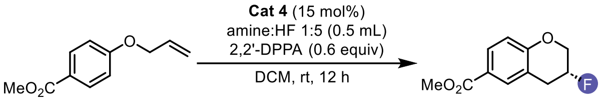

21a **25**, 55%, 92:8 er.

The corresponding substrate **21a** (0.20 mmol, 1.0 equiv.), **Cat 4** (0.015 mmol, 0.1 equiv.), amine:HF = 1:5.5 (0.5 mL) and 2,2'-DPPA (0.12 mmol, 0.6 equiv.) were dissolved in CH_2Cl_2 (0.5 mL) and stirred at ambient temperature. After 12 h, the reaction mixture was quenched with a saturated solution of $NaHCO_3$ and extracted with CH_2Cl_2 (3 x). The residue was purified by flash column chromatography on silica gel to give product **25**. HPLC (OJ-H column), Hexane : *i*PrOH = 90:10 as eluent, 1.0 mL/min, t_1 = 21.7 min, t_2 = 25.8 min.

Chiral HPLC spectrum of racemic 25

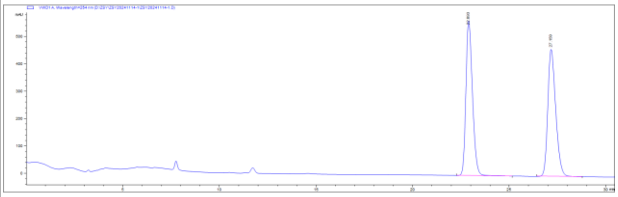

[illegible]

峰 #	保留时间 [min]	类型	峰宽 [min]	峰面积 [mAU*s]	峰高 [mAU]	峰面积 %
1	22.893	BB	0.3615	1.33828e4	566.81116	50.1642
2	27.159	BB	0.4372	1.32952e4	465.56671	49.8358

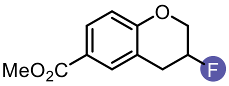

RAC-25

Chiral HPLC spectrum of 25

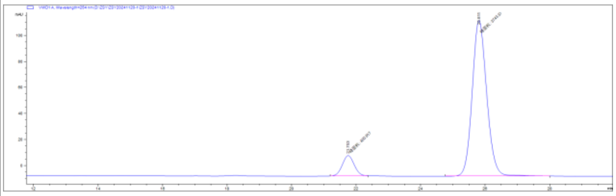

信号 1: VWD1 A, Wavelength=254 nm

峰 #	保留时间 [min]	类型	峰宽 [min]	峰面积 [mAU*s]	峰高 [mAU]	峰面积 %
1	21.753	MM	0.4272	400.91675	15.64213	7.6740
2	25.811	MM	0.5214	3743.36719	119.66173	92.3260

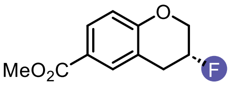

25

Figure S6. HPLC spectrum of **26**

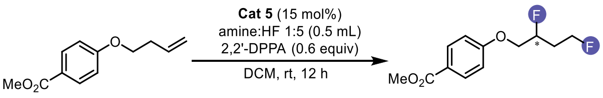

26a **26**, 45%, 68.5:31,5 er.

The corresponding substrate **26a** (0.20 mmol, 1.0 equiv.), **Cat 5** (0.015 mmol, 0.1 equiv.), amine:HF = 1:5.5 (0.5 mL) and 2,2'-DPPA (0.12 mmol, 0.6 equiv.) were dissolved in CH_2Cl_2 (0.5 mL) and stirred at ambient temperature. After 12 h, the reaction mixture was quenched with a saturated solution of $NaHCO_3$ and extracted with CH_2Cl_2 (3 x). The residue was purified by flash column chromatography on silica gel to give product **26**. HPLC (AD-H column), Hexane: *i*PrOH = 95:5 as eluent, 1.0 mL/min, t_1 = 13.3 min, t_2 = 14.2 min.

Chiral HPLC spectrum of racemic 26

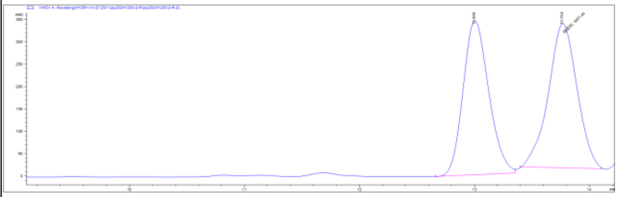

峰 #	保留时间 [min]	类型	峰宽 [min]	峰面积 [mAU*s]	峰高 [mAU]	峰面积 %
1	12.999	BV	0.2396	5344.95410	345.31161	48.8282
2	13.764	MM	0.2892	5601.49023	322.85034	51.1718

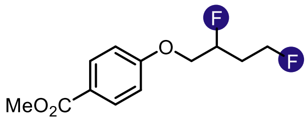

RAC-26

Chiral HPLC spectrum of 26

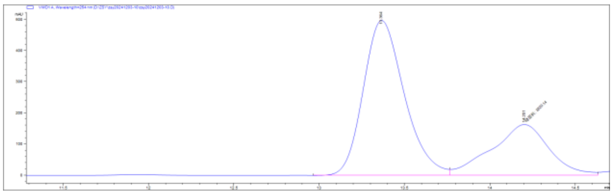

信号 1: VWD1 A, Wavelength=254 nm

峰 #	保留时间 [min]	类型	峰宽 [min]	峰面积 [mAU*s]	峰高 [mAU]	峰面积 %
1	13.364	BV	0.2560	8340.45410	498.92648	68.5859
2	14.201	MF	0.3894	3820.14331	163.51926	31.4141

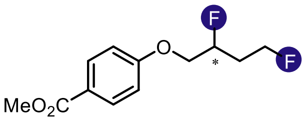

26

Figure S7. HPLC spectrum of **26**

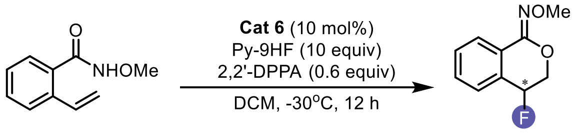

32a

32, 62%, 87.5:12.5 er.

The corresponding substrate **32a** (0.20 mmol, 1.0 equiv.), **Cat 6** (0.02 mmol, 0.1 equiv.), pyr-9HF (20 mmol, 100.0 equiv.) and 2,2'-DPPA (0.12 mmol, 0.6 equiv.) were dissolved in CH_2Cl_2 (0.5 mL) and stirred at -30 °C. After 48 h, the reaction mixture was quenched with a saturated solution of $NaHCO_3$ and extracted with CH_2Cl_2 (3 x). The residue was purified by flash column chromatography on silica gel to give product **32**. HPLC (AS-H column), Hexane : *i*PrOH = 99:1 as eluent, 1.0 mL/min, t_1 = 21.1 min, t_2 = 23.4 min.

Chiral HPLC spectrum of racemic 32

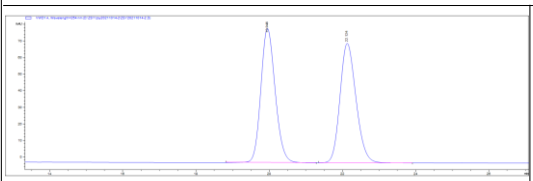

信号 1: VWD1 A, Wavelength=254 nm

峰 #	保留时间 [min]	类型	峰宽 [min]	峰面积 [mAU*s]	峰高 [mAU]	峰面积 %
1	19.946	BB	0.4204	2195.37939	80.69581	50.1384
2	22.124	BB	0.4703	2183.25977	71.89269	49.8616

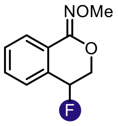

RAC-32

Chiral HPLC spectrum of 32

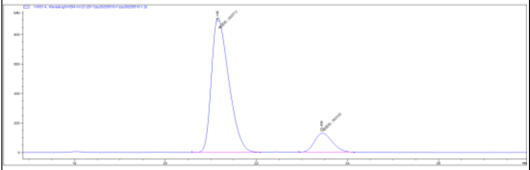

信号 1: VWD1 A, Wavelength=254 nm

峰 #	保留时间 [min]	类型	峰宽 [min]	峰面积 [mAU*s]	峰高 [mAU]	峰面积 %
1	21.145	MF	0.4412	2.42075e4	914.45526	87.2622
2	23.436	MM	0.4553	3533.62231	129.35330	12.7378

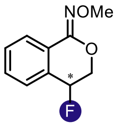

32

Figure S8. HPLC spectrum of **32**

12. Structural evaluation and analysis of cyclic [bis(acyloxyl)iodo]arene species

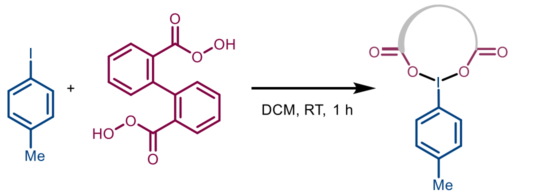

1k 2,2'-DPPA Cyclic (diacyloxyiodo)arenes

A dry round-bottom flask, flushed with argon and equipped with a magnetic stirrer bar and a septum, was charged with 4-iodotoluene **1k** (0.5 mmol), DCM (5 mL), and 2,2'-DPPA (1 mmol). The reaction mixture was allowed to react at room temperature for 1 hour. After the reaction, the product mixture was filtered to remove excess 2,2'-DPPA and resulting 2,2'-biphenyldicarboxylic acid. The filtrate was then concentrated under reduced pressure to yield a white powdery solid. **^{1}H NMR** (400 MHz, Chloroform-*d*) δ 7.94 – 7.54 (m, 3H), 7.51 – 7.30 (m, 1H), 7.23 – 7.06 (m, 4H), 7.04 – 6.65 (m, 4H), 2.39 (d, *J* = 14.5 Hz, 3H). **^{13}C NMR** (101 MHz, Chloroform-*d*) δ 171.79, 143.41, 135.04, 134.67, 134.37, 131.90, 131.67, 131.47, 130.93, 130.53, 130.43, 130.26, 129.96, 129.86, 129.75, 126.67, 77.33, 21.58. **HRMS** (ESI, m/z): calculated for $C_{63}H_{45}I_3NaO_{12}^+$ [M+Na]$^+$: 1396.9937, found: 1397.0225. and $C_{84}H_{60}I_4NaO_{16}^+$ [M+Na]$^+$: 1854.9952, found: 1855.0302.

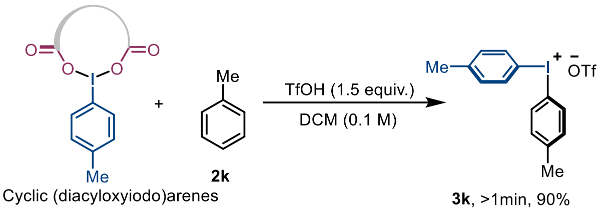

A dry round-bottom flask, flushed with argon and equipped with a magnetic stirrer bar and a septum, was prepared. Cyclic [bis(acyloxyl)iodo]arene species (0.3 mmol) and DCM (3 mL) were charged into the flask, then, **2k** (0.6 mmol) was added, and the solution was cooled to the specified temperature. TfOH (0.45 mmol) was added dropwise, resulting in a slight increase in temperature and a change in color from a clear solution to a yellow transparent solution. The solution was stirred at the specified temperature and time and then concentrated under vacuum while keeping it cold. Next, Et_2O (1-2 mL) was added, and the mixture was stirred at room temperature for 10 minutes to precipitate an off-white solid. The flask was stored in the freezer for 30 minutes, after which the solid was filtered off, washed with cold Et_2O, and dried under vacuum to obtain diaryliodonium salt **3k**.

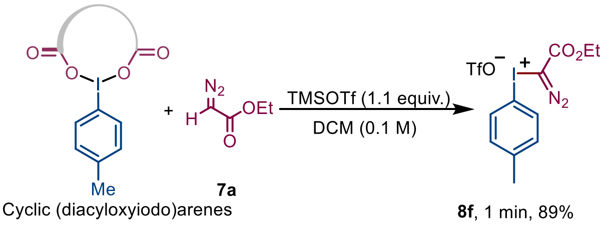

A dry round-bottom flask, flushed with argon and equipped with a magnetic stirrer bar and a septum, was charged cyclic [bis(acyloxyl)iodo]arene species (0.3 mmol) and DCM (3 mL). The resulting residue was then treated with trimethylsilyl trifluoromethanesulfonate (0.33 mmol) at room temperature. Afterward, the corresponding diazo compound **7a** (0.6 mmol) was added dropwise over a period of 10 minutes. Nitrogen evolution was observed, and the resulting yellow reaction mixture was stirred for 1 minutes at room temperature. The solvent was removed under vacuum, and the crude product was recrystallized from a mixture of diethyl ether/dichloromethane (5/1) at -30 °C for 12 hours. The product was collected by filtration, washed with cold diethyl ether, dried under high vacuum, and stored at -30 °C.

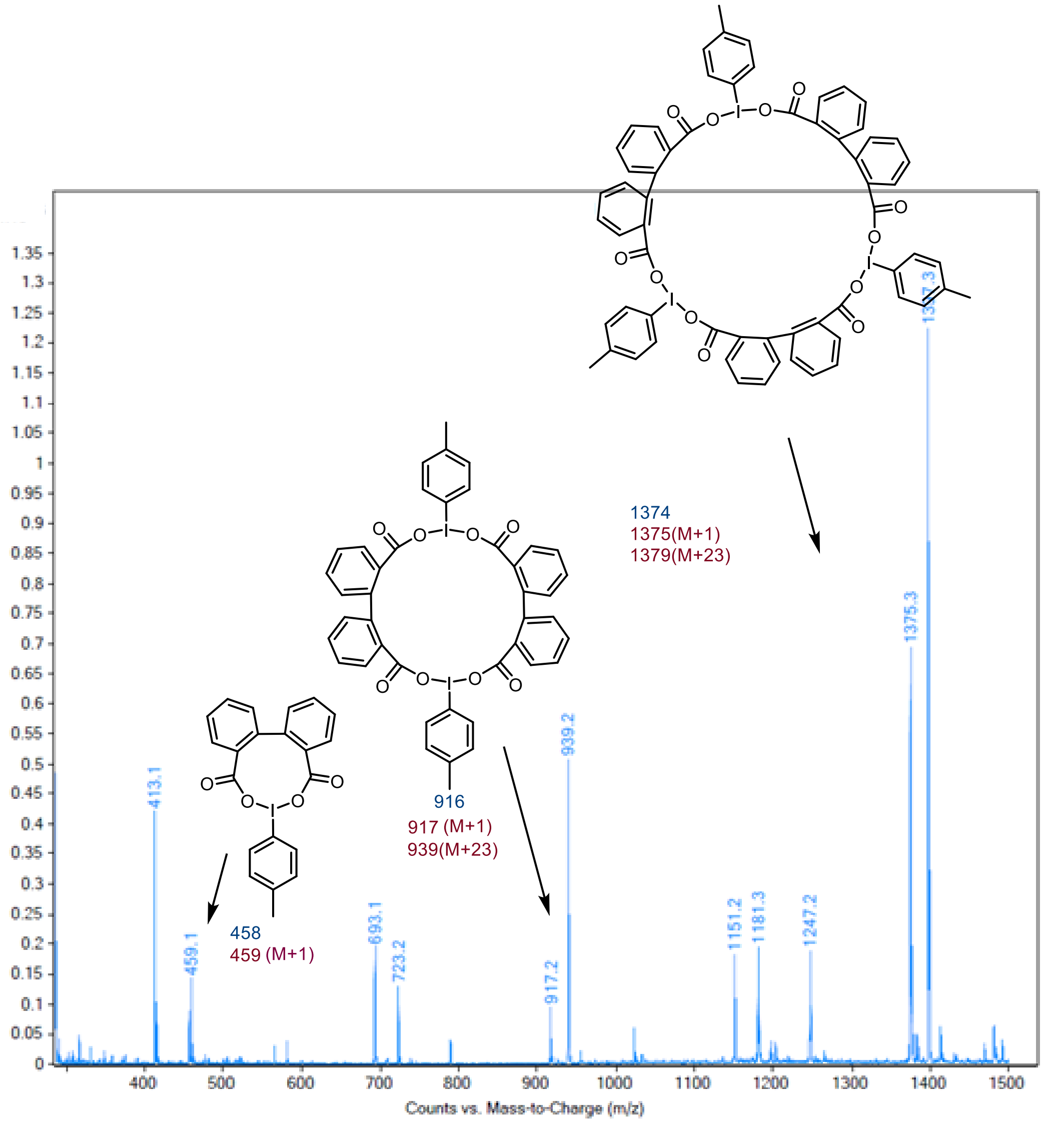

Figure S9. LC-MS of cyclic [bis(acyloxyl)iodo]arene species.

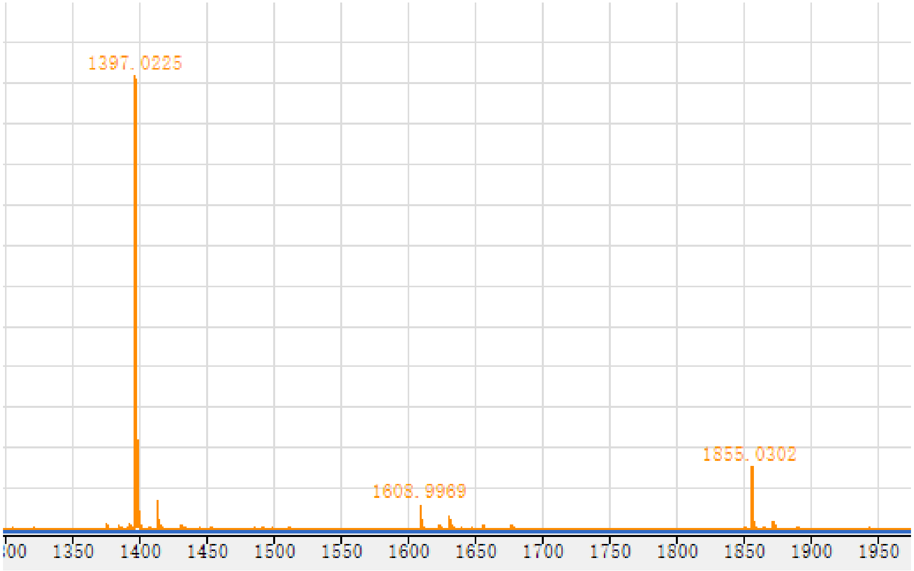

Figure S10. HRMS of cyclic [bis(acyloxyl)iodo]arene species.

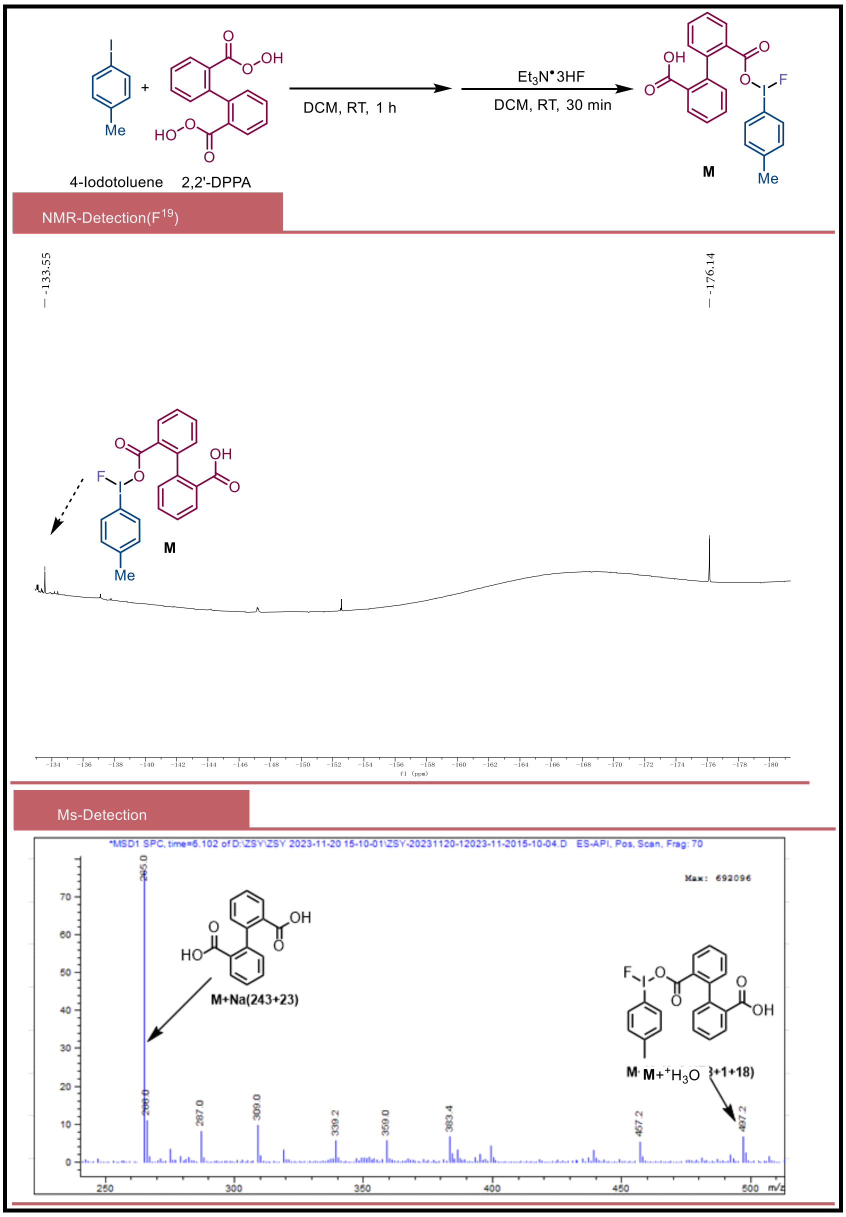

Figure S11. ^{19}F NMR and LC-MS of **M**.

13. The synthesis of DBD in aqueous solution

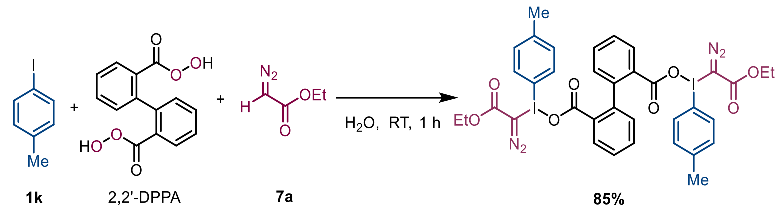

A dry round bottom flask flushed with argon and equipped with a magnetic stirrer bar and a septum was charged with aryl iodide **1k** (0.3 mmol), H_2O (3 mL) and 2,2'-DPPA (0.6 mmol) was added. The reaction mixture was reacted at room temperature for 5 minutes. After this, the corresponding diazo compound **7a** (0.6 mmol) was added dropwise for 10 minutes. Nitrogen evolution was observed, and the

resulting yellow reaction mixture was stirred for 1 hour at room temperature. Solvent was removed under vacuum and the crude was recrystallized from a mixture of diethyl ether/dichloromethane (5/1) during 12 hours at -30 °C. The product Bis((1-diazo-2-ethoxy-2-oxoethyl)(phenyl)- λ^3-iodaneyl) [1,1'-biphenyl]-2,2'-dicarboxylate was collected by filtration, washed with cold diethyl ether (20 mL), dried under high vacuum and stored at -30 °C. **^{1}H NMR** (400 MHz, Methanol-d_4) δ 8.01 (t, J = 10.4 Hz, 4H), 7.74 – 7.63 (m, 2H), 7.41 (tt, J = 12.4, 8.8, 7.7 Hz, 8H), 7.18 – 7.01 (m, 2H), 4.32 (dq, J = 14.0, 7.0, 6.5 Hz, 4H), 2.50 (s, 6H), 1.31 (t, J = 13.4 Hz, 6H). **^{13}C NMR** (101 MHz, Methanol-d_4) δ 172.74, 162.49, 143.64, 134.59, 133.24, 132.13, 131.27, 129.74, 129.52, 129.52, 128.13, 126.95, 116.16, 63.30, 20.08, 13.26. **HRMS** (ESI, m/z): calculated for $C_{36}H_{33}I_2N_4O_8^+$ [M+H]$^+$: 903.0382, found: 903.0390.

14. Biomolecule-compatibility experiments

To a solution of **1k** (40.8 mg, 0.2 mmol), 2,2'-DPPA (0.1 mmol, 27 mg), **7a** (34.2mg, 0.3mmol) and additives (0.2 mmol) in 1 X PBS, and stirred at room temperature.

14.1. Compatibility with biomolecules in 1 X PBS

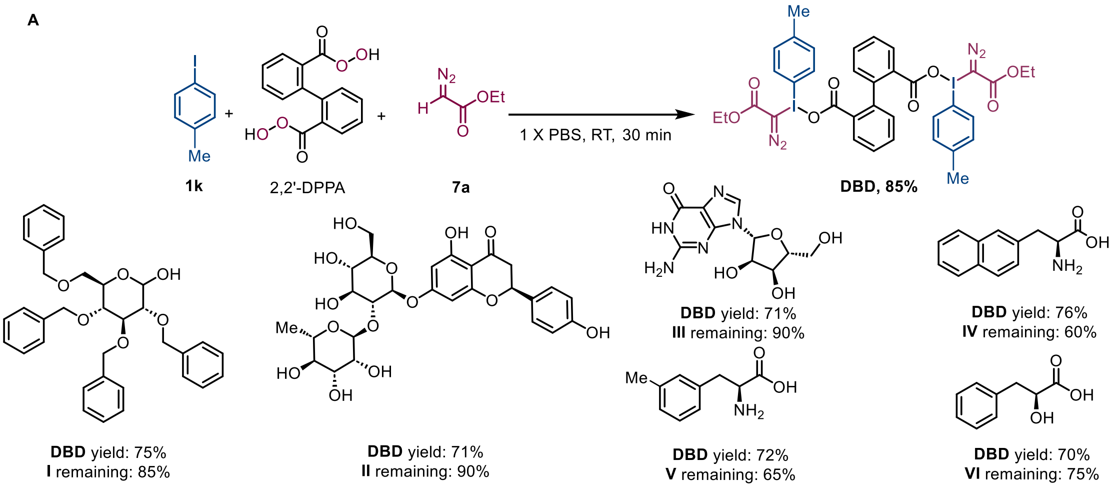

Figure S12. Compatibility with biomolecules.

[a]Isolated yields. [b]additive remaining was determined by LC-MS.

14.2. The stability of nucleic acids in the oxidation

1k (0.01 mmol, 0.8 mg), 2,2'-DPPA (0.005mol, 1.4 mg) ,**7a** (0.015 mmol, 1.7 mg) and 5 μL pUC18/19 were oscillated in 100 mL 1 X PBS for 2h. Then a portion of the reaction mixture was extracted with EA then subjected to LC-MS analysis monitored 210 nm, internal standard is diphenic acid. The agarose gel electrophoresis was conducted to confirm the pUC18/19 was not degraded.

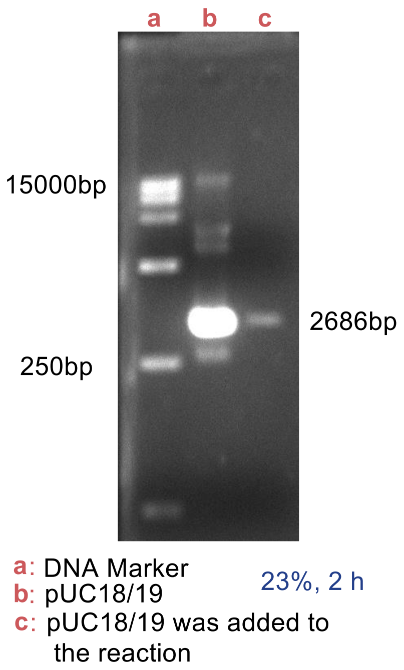

Figure S13. The electrophoretic of pUC18/19.

14.3. The activity of DNase I in the oxidation

We treated 100 μM of **1k**, 55 μM of 2,2'-DPPA 150 μM 7**a** and 0.32 μM DNase I were oscillated in 1 X PBS for 2 h. Then a portion of the reaction mixture was extracted with EA then subjected to LC-MS analysis monitored 254 nm, internal standard is diphenic acid. 10 μL the reaction mixture was added 5 μL pUC18/19, incubated at 37 °C for 2 h, then examined by agarose gel electrophoresis.

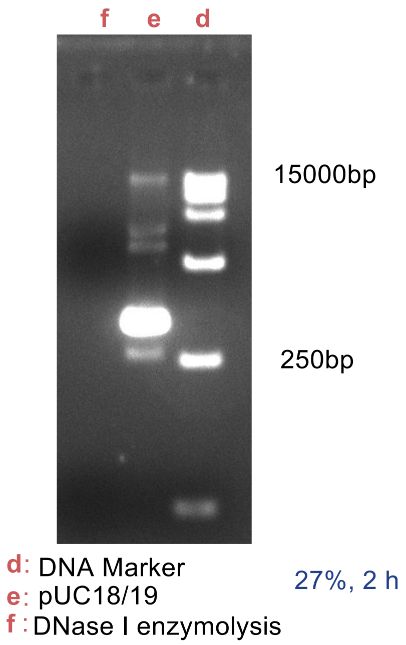

Figure S14. The activity of DNase I.

14.4. The stability of proteins in the oxidation

1k (0.01 mmol, 0.8 mg), 2,2'-DPPA (0.005mol, 1.4 mg), **7a** (0.015 mmol, 1.7 mg) and 100 mg (1mg/mL) Bovine serum albumin were oscillated in 100 mL 1 X PBS for 2 h. Then a portion of the reaction mixture was extracted with EA then subjected to LC-MS analysis monitored 210 nm, internal standard is diphenic acid. The other reaction mixture was diluted four times, and the amount of albumin bovine serum remaining was determined by Biotek Synergy HTX

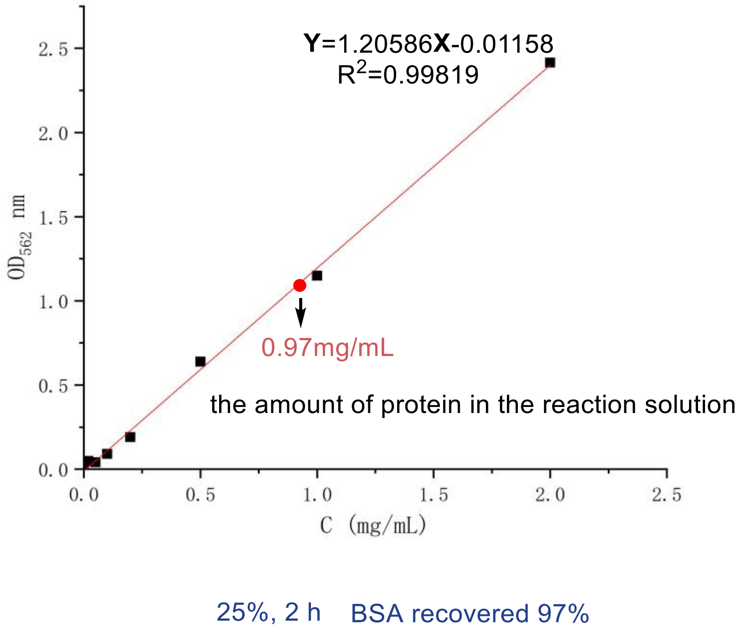

Figure S15. BSA concentration test by Biotek Synergy HTX.

14.5. Preparation of ethyl 2-diazo-2-(2-(dimethylamino)phenyl) acetate 12

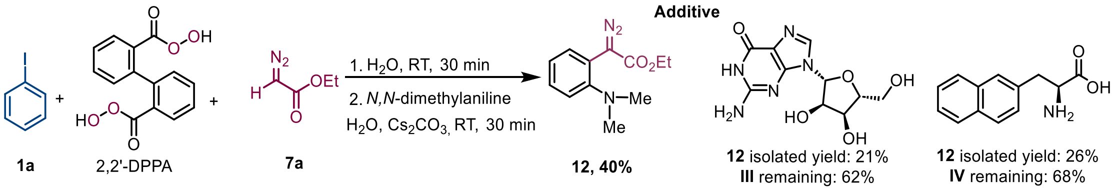

A dry round bottom flask flushed with argon and equipped with a magnetic stirrer bar and a septum was charged with aryl iodide **1a** (0.3 mmol), H_2O (3 mL) and 2,2'-DPPA (0.15 mmol) was added. The reaction mixture was reacted at room temperature for 5 minutes. After this, the corresponding diazo compound **4** (0.6 mmol) was added dropwise for 10 minutes. Nitrogen evolution was observed, and the resulting yellow reaction mixture was stirred for 30 min at room temperature. Then, *N, N*-dimethylaniline (0.1 mmol, 1 equiv.) and K_2CO_3 (0.15 mmol, 1.5 equiv.) were added, the resulting yellow reaction mixture was stirred for 30 min at room temperature. The residue was purified by flash column chromatography on silica gel to give **12** in 40% yield.

15. Chemoselective labelling at methionine residues

15.1. Preparation of amino acid 33 and 34

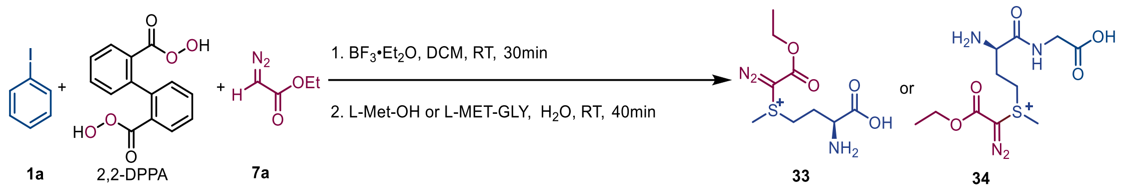

A dry round bottom flask flushed with argon and equipped with a magnetic stirrer bar and a septum was charged with aryl iodide **1a** (0.3 mmol), DCM (3mL). 2,2'-DPPA (0.17 mmol) was added, the reaction mixture was reacted at room temperature for 5 minutes. The resultant residue was treated with BF_3-Et_2O (0.33 mmol) at room temperature. After this, the corresponding diazo compound **7a** (0.45 mmol) was added dropwise for 10 minutes. Nitrogen evolution was observed, and the resulting yellow reaction mixture was stirred for 30 min at room temperature. Solvent was removed under vacuum and dissolved in H_2O (0.3 M). A round-bottomed flask equipped with a magnetic stir-bar was charged with L-Methionine (0.1 mmol) or L-methionine-glycine (0.1 mmol) and then evacuated and refilled with N_2. The resultant residue was dissolved in H_2O (1.0 M). To the flask was added 0.5 mL a freshly degassed solution of amino acids, and the resulting solution was allowed to stir for 40 min at room temperature. The resulting heterogeneous solution was then diluted with water and ethyl acetate and the layers separated by decanting the organic layer. The aqueous layer was washed twice with ethyl acetate in the same fashion. The aqueous layer was then evaporated to dryness in vacuo. The resulting faint yellow residue was then redissolved in a minimal amount of acetonitrile and precipitated with ether in a 4 °C refrigerator. The supernatant was decanted, and the residue was dried in vacuo to yield **33** or **34** as a faint yellow residue.[15]

15.2. Preparation of peptides 35

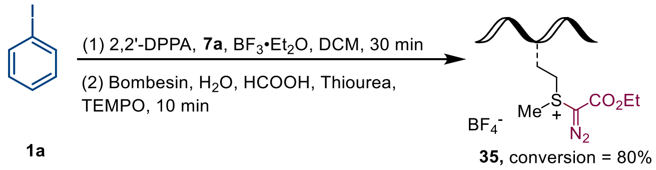

A dry round bottom flask flushed with argon and equipped with a magnetic stirrer bar and a septum was charged with aryl iodide **1a** (0.01 mmol), DCM (0.1 mL). 2,2'-DPPA (0.005 mmol) was added, the reaction mixture was reacted at room temperature for 5 minutes. The resultant residue was treated with BF_3-Et_2O (0.011 mmol) at room temperature. After this, the corresponding diazo compound **7a** (0.015 mmol) was added, the resulting yellow reaction mixture was stirred for 30 minutes at room temperature. Solvent was removed under vacuum and dissolved in H_2O (100 mM). A 2 mL vial equipped with a magnetic stirrer was charged with a solution containing the bombesin (1 mM in 0.1M thiourea (aq.), 20 μL). To the vial was added thiourea (100 mM in H_2O, 20 μL), formic acid (100 mM, 10 μL), and H_2O (20 μL). The vial was chilled in an ice bath. To the chilled solution was added TEMPO (33 mM in H_2O, 30 μL) and the vial was stirred vigorously. To the stirring solution was added the iodonium salt (100 μL) and the resulting solution stirred for 10 minutes at 0° C. The resulting mixture was then extracted twice with diethyl ether or ethyl acetate. The remaining organic volatiles were then removed from the aqueous layer using a rotary evaporator. The resulting solution was then analyzed directly via LC-MS.[15]

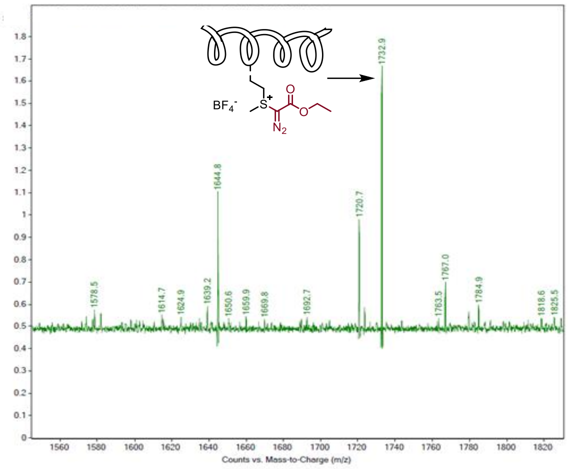

Figure S16. LC-MS of peptides **35**.

15.3. Preparation of peptides 38

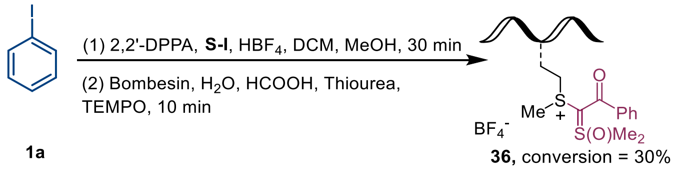

A dry round bottom flask flushed with argon and equipped with a magnetic stirrer bar and a septum was charged with aryl iodide **1a** (0.01 mmol), DCM (0.1 mL). 2,2'-DPPA (0.005 mmol) was added, the reaction mixture was reacted at room temperature for 5 minutes. The solvent was removed under reduced pressure. The resultant residue was dissolved in MeOH (0.1 mL) was treated with corresponding acid HBF_4 (0.01 mmol) at room temperature. This clear solution was added dropwise to the ice bath-cooled solution of sulfoxonium ylides **S-I** (0.01 mmol) in MeOH (0.1 mL) with stirring. The resulting reaction mixture was stirred at 0 °C for an additional 30 min, solvent was removed under vacuum and dissolved in H_2O (100 mM). A 2 mL vial equipped with a magnetic stirrer was charged with a solution containing the bombesin (1 mM in 0.1M thiourea (aq), 20 μL). To the vial was added thiourea (100 mM in H_2O, 20μL), formic acid (100 mM, 10 μL), and H_2O (20 μL). The vial was chilled in an ice bath. To the chilled solution was added TEMPO (33 mM in H_2O, 30 μL) and the vial was stirred vigorously. To the stirring solution was added the iodonium salt (100 μL) and the resulting solution stirred for 10 minutes at 0° C. The resulting mixture was then extracted twice with diethyl ether or ethyl acetate. The remaining organic volatiles were then removed from the aqueous layer using a rotary evaporator. The resulting solution was then analyzed directly via LC-MS.

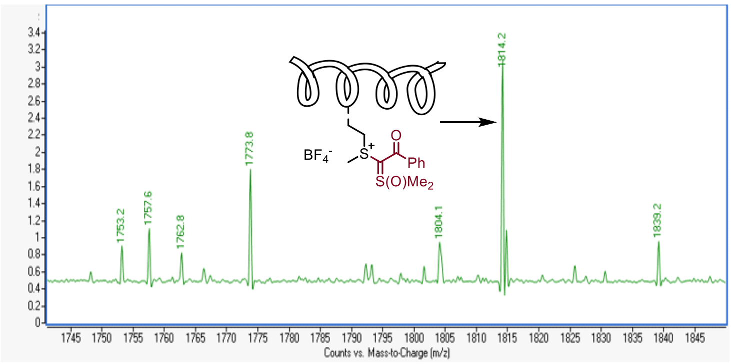

Figure S17. LC-MS of peptides **36**.

16. Computational details

We compute the pathway that would generate key intermediate **D** under the reaction conditions (Fig. S14). The results suggested that the reaction proceeds via the oxidation of two iodobenzene molecules to form the key intermediate **D**, by releasing the iodosylbenzene and one H_2O molecule. The two oxidation steps own the same energy barriers of 20.1 kcal/mol, which is feasible at room temperature, where the intra-molecular hydrogen-bonding networks and additional halogen bonding should contribute to the stability. Finally, the intermediate **D** could transform into its dimeric intermediate **E**, which is much more stable in energy with a comfortable T-type geometry of the iodine atom. After releasing the iodosylbenzene, we also calculated the pathways showing higher energy barriers leading to other minor intermediate in the reaction. The computational results are consistent with the experimental observations.

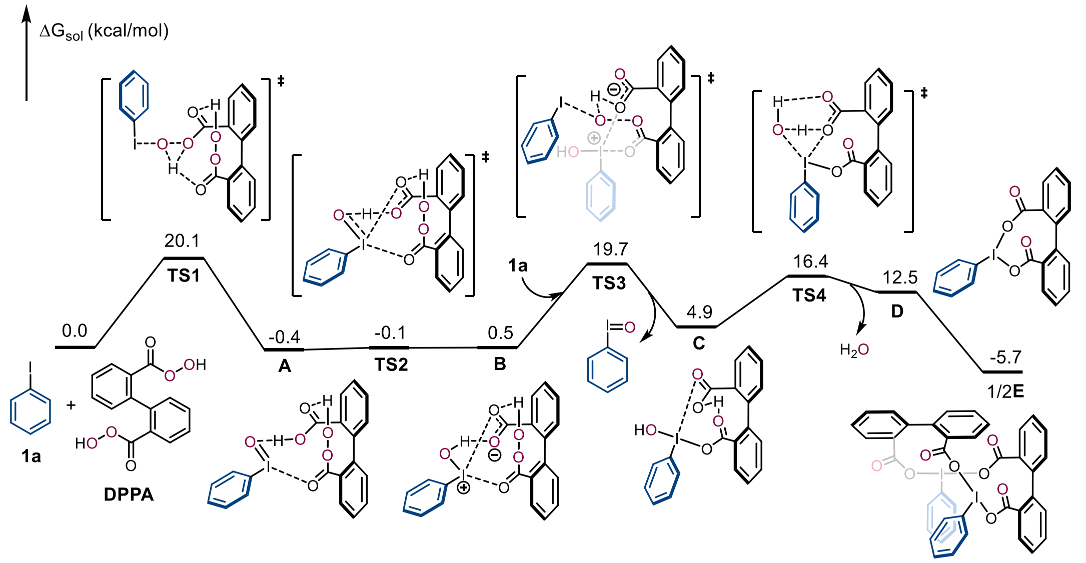

Figure S18. Free energy profile of the pathway to generate key intermediate **D**.

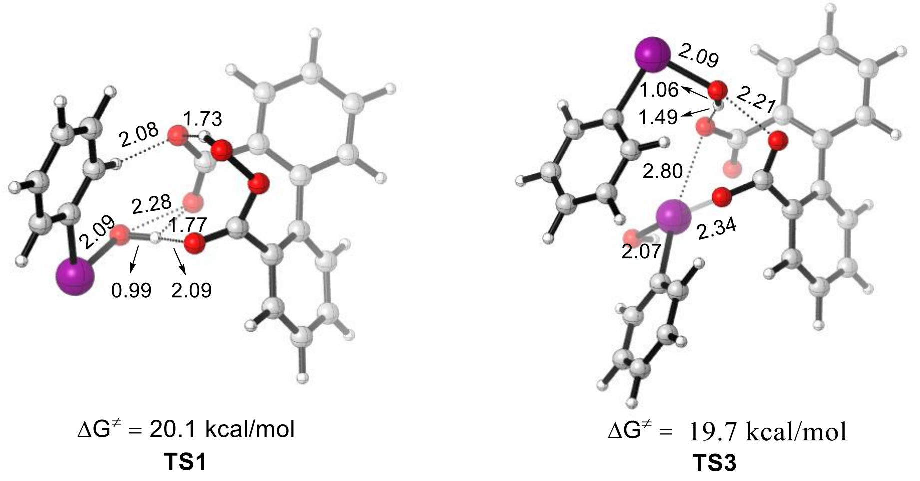

Figure S19. Computed structures of transition states **TS1** and **TS3**.

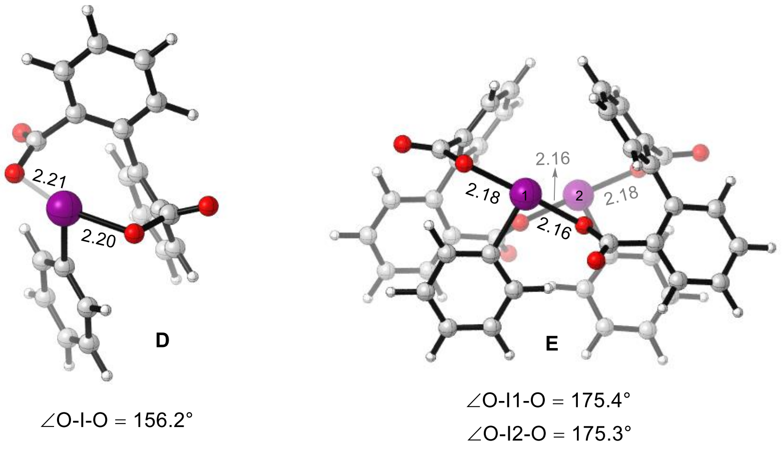

Figure S20. Computed geometries of key intermediate **D** and the dimer **E**.

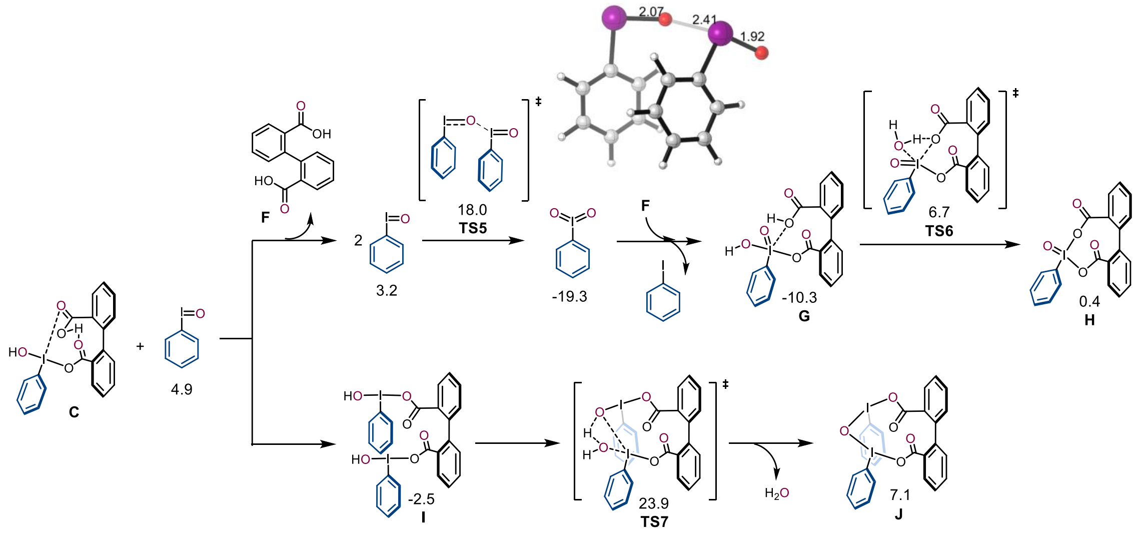

Figure S21. Computed pathways to generate minor intermediates in the reaction.

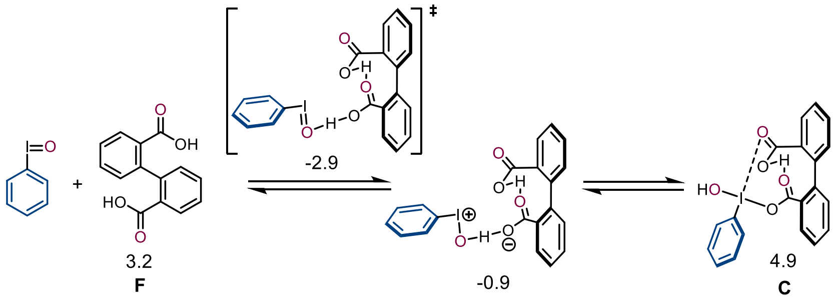

Figure S22. Free energy profile of the pathway to generate intermediate **C**.

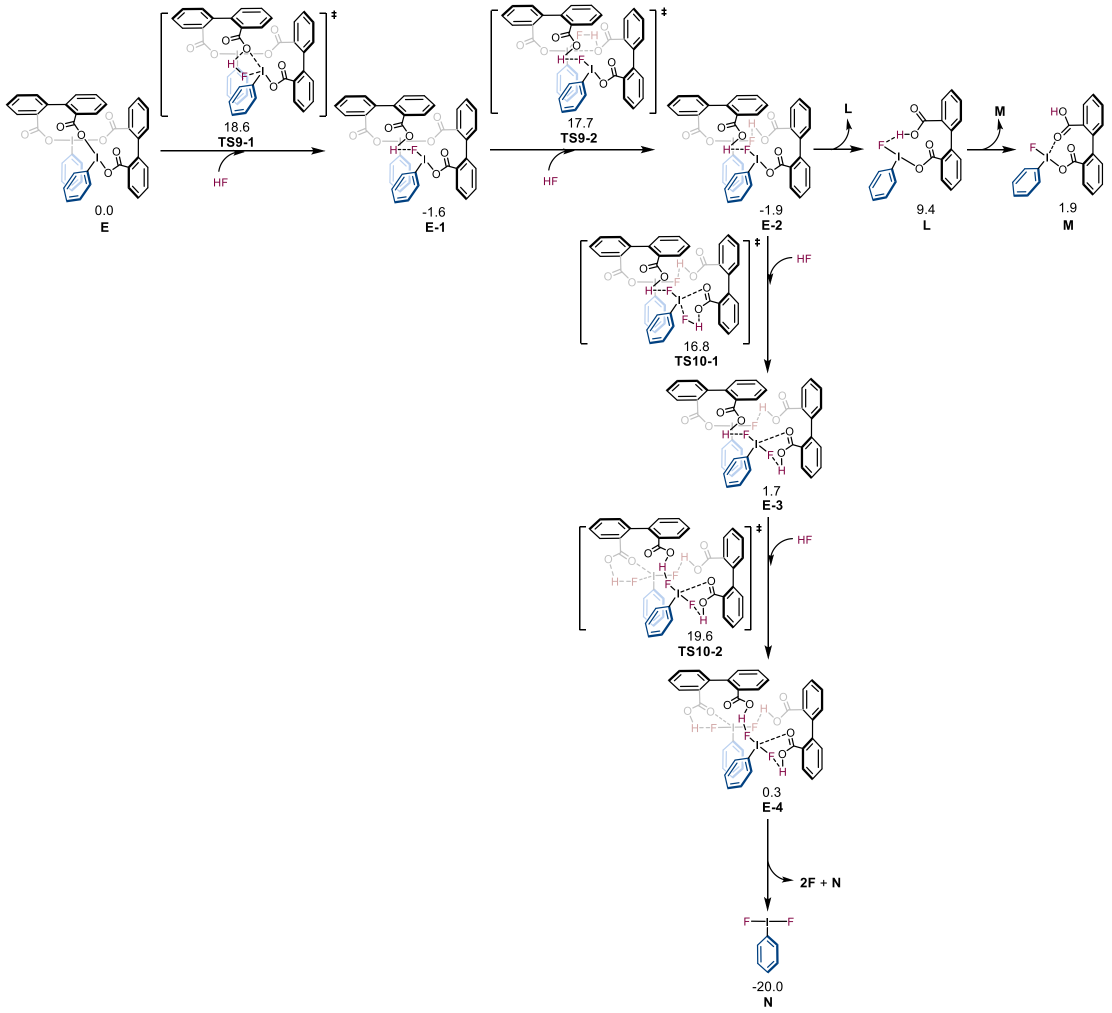

Figure S23. Free energy profile of the pathway to generate key intermediate **M**.

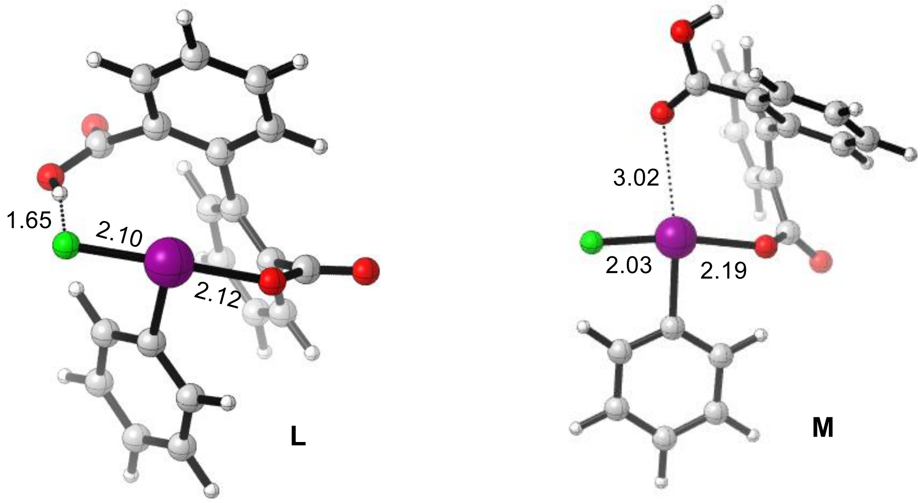

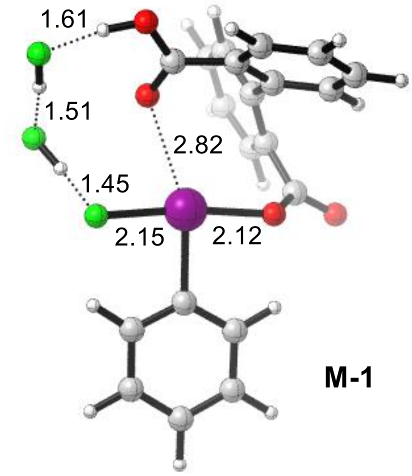

Figure S24. Computed geometries of intermediates **L, M**.

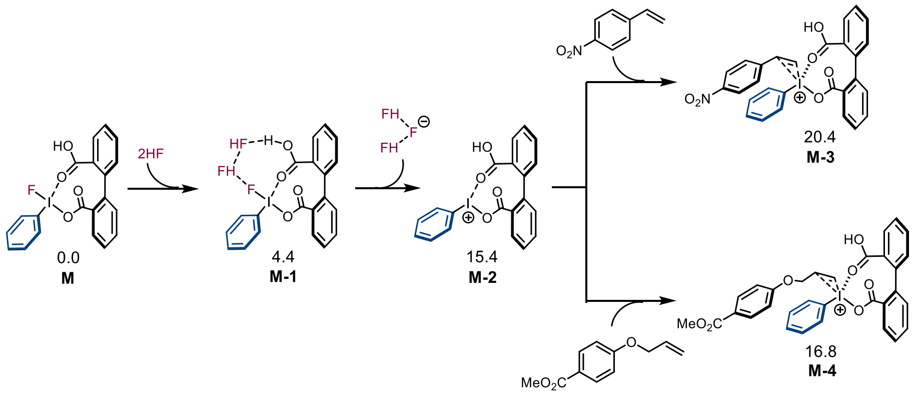

Figure S25. Computed pathways to generate olefin adducts catalyzed by iodoarene fluoride **M**.

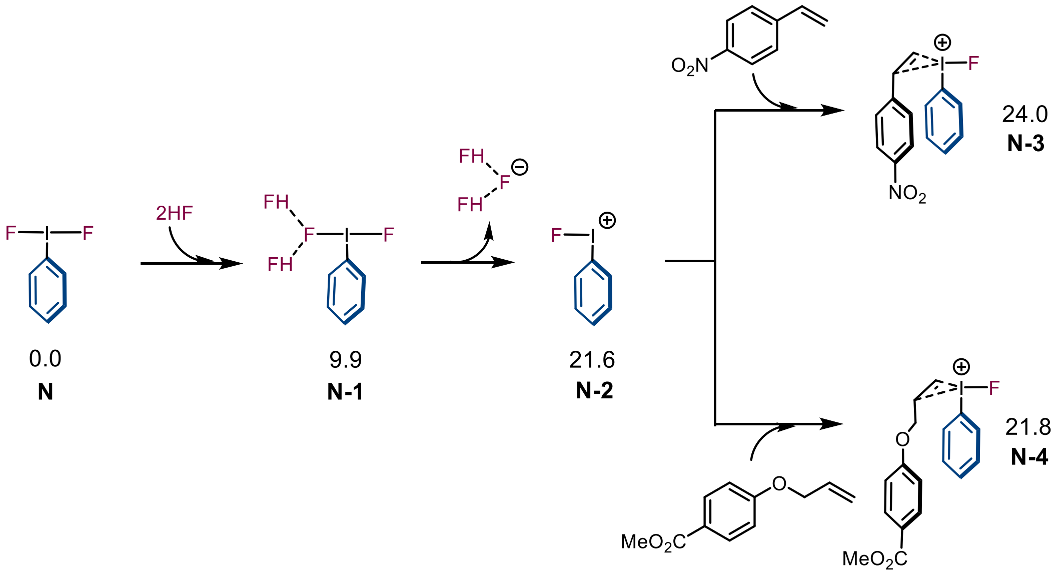

Figure S26. Computed pathways to generate olefin adducts catalyzed by iodoarene

difluoride **N**.

Table S11. Energetics of Computed Intermediates and TSs.

Complex	SCF-Done Energy	Single Point Energy in Solvent	Free Energy with Corrections in Solvent	Imaginary Frequency
1a	-242.987920	-527.257305	-527.198693	
DPPA	-990.537275	-990.718655	-990.543426	
A	-1233.505099	-1517.994816	-1517.742716	
B	-1233.503682	-1517.993459	-1517.741402	
C	-1158.378732	-1442.864701	-1442.614565	
D	-1081.956720	-1366.396076	-1366.168070	
E	-2164.021385	-2732.876150	-2732.394125	
E-1	-2264.471290	-2833.357355	-2832.864870	
E-2	-2364.916960	-2933.837197	-2933.333541	
E-3	-2465.358974	-3034.312519	-3033.795909	
E-4	-2565.805010	-3134.794265	-3134.266241	
F	-840.290226	-840.468648	-840.298706	
G	-1233.478142	-1518.008498	-1517.758594	
H	-1157.062942	-1441.535434	-1441.307167	
I	-1476.509677	-2045.279531	-2044.944758	
J	-1400.079196	-1968.805727	-1968.495208	
K	-1158.377273	-1442.870373	-1442.623727	
L	-1182.416859	-1466.898288	-1466.657726	
M	-1182.411213	-1466.900251	-1466.663715	
M-1	-1383.302490	-1667.850168	-1667.593050	
M-2	-1082.341744	-1366.839233	-1366.603427	
M-3	-1596.406922	-1880.999101	-1880.639012	
M-4	-1734.248371	-2018.903245	-2018.475985	
N	-442.559387	-726.910754	-726.851583	
N-1	-643.420519	-927.848644	-927.772181	
N-2	-342.447733	-626.839093	-626.781360	
N-3	-856.525535	-1141.002755	-1140.821160	
N-4	-994.362549	-1278.904216	-1278.655853	
TS1	-1233.478102	-1517.961608	-1517.710019	-270.71
TS2	-1233.505640	-1517.992666	-1517.742336	-165.46
TS3	-1476.470341	-2045.239918	-2044.909492	-56.13
TS4	-1158.369060	-1442.845589	-1442.596115	-441.46
TS5	-636.142744	-1204.750868	-1204.613423	-105.90
TS6	-1233.459414	-1517.978638	-1517.731449	-609.25
TS7	-1476.469713	-2045.236179	-2044.902690	-122.49
TS8	-1158.379506	-1442.869837	-1442.626862	-727.72
TS9	-1182.381354	-1466.858603	-1466.621786	-96.94
TS9-1	-2264.439051	-2833.323641	-2832.832660	-158.69
TS9-2	-2364.885950	-2933.802492	-2933.302210	-249.05
TS10	-1282.826748	-1567.335245	-1567.087184	-387.57

TS10-1	-2465.332732	-3034.284653	-3033.771867	-105.16
TS10-2	-2565.774469	-3134.761233	-3134.235550	-86.69
H_2O	-76.388362	-76.437160	-76.43431499	
HF	-100.411294	-100.460784	-100.468156	
$[FH\text{-}F\text{-}HF]^-$	-300.746305	-300.967774	-300.972054	
Iodosylbenzene	-318.058149	-602.376299	-602.318499	
Iodylbenzene	-393.169160	-677.533130	-677.474192	

Cartesian Coordinates

1a

C	0.22951900	0.45655200	0.00004600
C	1.62651000	0.44725800	0.00055300
C	2.30579100	1.66356100	0.00001800
C	1.62660700	2.87994600	-0.00100500
C	0.22964200	2.87078300	-0.00150000
C	-0.47019800	1.66368800	-0.00097800
H	-0.30785700	-0.48756400	0.00046300
H	2.16981700	-0.49082200	0.00134500
H	2.17003200	3.81795900	-0.00141100
H	-0.30768700	3.81492600	-0.00229200
H	-1.55603800	1.66375800	-0.00137000
I	4.45929900	1.66351200	0.00079700

2.2'-DPPA

C	0.08988800	-0.51415300	0.72663300
O	0.04462100	-0.47785900	1.94063800
O	1.22977400	-0.59904400	-0.00052200
C	-1.10799400	-0.42219400	-0.15678900
C	-2.20408400	-1.19518900	0.24299400
C	-1.20823100	0.44960200	-1.26433700
C	-3.40043900	-1.15570800	-0.46447300
H	-2.09877400	-1.82852500	1.11752900
C	-2.43392700	0.48676500	-1.94692400
C	-3.51242500	-0.30686100	-1.56457600
H	-4.23872600	-1.77022900	-0.15186500
H	-2.54537000	1.17712600	-2.77663500
H	-4.44496400	-0.24721500	-2.11785600
C	-0.11881400	1.33706100	-1.76684000
C	0.13087100	1.32941600	-3.14788400
C	0.68351000	2.18124300	-0.96662600
C	1.11989500	2.12522600	-3.72034100
H	-0.44758300	0.66019000	-3.77633000
C	1.69699700	2.95614300	-1.54212500
C	1.90898200	2.94627800	-2.91625900
H	1.28328900	2.08879500	-4.79332800
H	2.31331300	3.56733500	-0.89127200
H	2.68966800	3.56208000	-3.35146300
C	0.60695800	2.23986900	0.52148500
O	1.57344800	2.17662700	1.25574200
O	-0.67742900	2.32875200	0.94310200
O	-0.78842100	2.21970600	2.37811100
H	-0.59367100	1.25774600	2.49481900
O	2.41021700	-0.52182400	0.82646100
H	2.38699000	0.43482300	1.07368100

[FH-F-HF]$^-$

F	1.01899700	0.95625800	4.44533300
F	2.39783000	-0.93054200	4.04739100
H	1.81033600	-0.11733300	4.22103000
F	2.40686000	2.46870000	5.63189800
H	1.81135300	1.81773400	5.12428900

A

C	-0.65410800	0.12356500	1.69577000
O	-1.13048200	0.68176900	2.69390100
O	0.50116800	-0.48787500	1.66558800
C	-1.38955700	0.10767100	0.39382400
C	-2.70227800	-0.36656400	0.39252100
C	-0.81154600	0.60195800	-0.79046000
C	-3.44032100	-0.41123400	-0.78934100
H	-3.13490400	-0.70947800	1.32711900
C	-1.56894100	0.55857900	-1.96718100
C	-2.86794800	0.04968100	-1.97335800
H	-4.45522000	-0.79723700	-0.78199200
H	-1.13688000	0.95353200	-2.88183400
H	-3.43383700	0.02929300	-2.90022200
C	0.56433200	1.17801700	-0.86436400
C	1.46404000	0.53934300	-1.73099200
C	1.02050900	2.32471500	-0.16693100
C	2.76728400	0.99665400	-1.90809900
H	1.12724600	-0.34923000	-2.25533500
C	2.33501700	2.77702900	-0.35654200
C	3.20817200	2.12530700	-1.21721100
H	3.43638700	0.46732300	-2.58064500
H	2.65651800	3.64999200	0.19661800
H	4.22260800	2.49083000	-1.33869100
C	0.24143000	3.10075100	0.84863800
O	0.74614400	3.80824300	1.69442700
O	-1.09378200	2.89536100	0.70996900
O	-1.82222100	3.24237300	1.90835100
H	-1.82635300	2.33694800	2.31818300
O	1.79634100	-0.17513000	3.79035800
H	1.01465800	-0.40749500	2.59418200
C	4.96831200	3.82977100	1.98810500
C	3.80022900	3.57890900	2.71362500
C	3.45401200	2.25213200	2.92175200
C	4.17960800	1.17443800	2.44679100
C	5.33922300	1.45022400	1.71721300
C	5.73278800	2.77079900	1.49190100
H	5.27088800	4.85677900	1.80591100
H	3.17793600	4.39352700	3.06678900
H	3.83519400	0.16210900	2.64023900
H	5.93068400	0.62841600	1.32473100
H	6.63678700	2.97727400	0.92674800
I	1.63296800	1.75438600	3.98624200

B

C	-0.17631000	-0.03540000	1.15506800
O	-0.39195800	0.31970400	2.36936600
O	0.92627000	-0.43022400	0.69901200
C	-1.34519500	0.06178400	0.21438200
C	-2.56672800	-0.47807100	0.62241500

C	-1.23914200	0.70092900	-1.03686100
C	-3.68341500	-0.43591000	-0.21092600
H	-2.62752400	-0.93827200	1.60325100
C	-2.37579200	0.74922800	-1.85556200
C	-3.58552900	0.18188400	-1.45591700
H	-4.62233900	-0.87405200	0.11474300
H	-2.30968900	1.26144300	-2.81087100
H	-4.44912000	0.23609200	-2.11258500
C	0.00936800	1.33154600	-1.56182700
C	0.45859900	0.87443900	-2.81078700
C	0.74937300	2.36903200	-0.94190700
C	1.58413700	1.40726300	-3.43293200
H	-0.08623600	0.06512000	-3.28592200
C	1.88743100	2.89308200	-1.57313200
C	2.30589100	2.42730200	-2.81284700
H	1.89943900	1.01984600	-4.39760100
H	2.43270100	3.68000500	-1.06391400
H	3.18575800	2.85089600	-3.28747000
C	0.47196900	2.94547100	0.40695800
O	1.30791200	3.47523500	1.10943900
O	-0.83714000	2.80325600	0.74207400
O	-1.06216300	2.94058200	2.16380200
H	-1.06607700	1.96599100	2.37875300
O	2.46433300	-0.58242200	2.73910300
H	1.93437100	-0.62739400	1.84858100
C	5.56630100	3.42897900	2.63358100
C	4.26185400	2.93204400	2.58993100
C	3.90436600	1.94136500	3.50493000
C	4.79942300	1.42358600	4.44010400
C	6.10071000	1.92532600	4.45934800
C	6.48032800	2.92608300	3.56082700
H	5.86692100	4.20356300	1.93450900
H	3.53901200	3.30205500	1.86920400
H	4.49596300	0.64476700	5.13136700
H	6.81607600	1.53434100	5.17658000
H	7.49445200	3.31379000	3.58439400
I	1.90150300	1.21317800	3.39063800

C

C	-0.78177700	0.17832000	1.40838700
O	-0.90769000	0.71206800	2.64424200
O	0.01217800	-0.71835500	1.20680700
C	-1.69790700	0.74667300	0.37053000
C	-3.07393200	0.62468100	0.58394200
C	-1.20555900	1.36488000	-0.79415400
C	-3.98210500	1.07091700	-0.37469600
H	-3.42850200	0.15962400	1.49911200
C	-2.13505600	1.81746900	-1.73955700
C	-3.50706300	1.66727100	-1.54120400
H	-5.04885900	0.95768400	-0.20727700
H	-1.76974700	2.32173800	-2.62883100
H	-4.20223000	2.03144800	-2.29203900
C	0.24244700	1.57990100	-1.06607000
C	0.74250000	1.15608100	-2.30663700
C	1.13025500	2.20491800	-0.16225300
C	2.07312400	1.35920400	-2.66524600
H	0.07112200	0.64440900	-2.98947900

C	2.46679700	2.40023200	-0.53269000
C	2.94054900	1.99498100	-1.77704500
H	2.42926000	1.01925400	-3.63351100
H	3.13648600	2.88905800	0.16645400
H	3.97796500	2.16945900	-2.04737800
C	0.68638300	2.69281800	1.19605100
O	1.53722700	2.53757600	2.18847600
O	-0.42371200	3.19700600	1.36080300
H	-1.21609500	1.63953000	2.55056100
O	3.72158800	-1.19946600	2.72571500
H	3.79147200	-1.60105600	1.84100100
C	6.80331800	1.76142300	2.71472000
C	5.62212500	1.01686800	2.64547700
C	4.42561300	1.71210500	2.57292600
C	4.33811400	3.09675600	2.59032200
C	5.53414600	3.81817500	2.65972000
C	6.76112400	3.15616900	2.71729000
H	7.75521400	1.24134400	2.77008500
H	5.62384400	-0.06698300	2.66625300
H	3.37507900	3.59079700	2.55368600
H	5.49535200	4.90342400	2.67367700
H	7.68339200	3.72677000	2.77156800
I	2.59165300	0.52944500	2.45319700

D

C	0.18862200	-0.33481800	1.35649000
O	0.64490300	0.17316200	2.52095200
O	0.55728200	-1.43142600	0.98656000
C	-0.86726400	0.46132700	0.60273000
C	-2.19976600	0.16132400	0.90208100
C	-0.57249600	1.51250000	-0.29876600
C	-3.24229200	0.90113000	0.34151600
H	-2.41718100	-0.64854800	1.59253900
C	-1.63002500	2.25859600	-0.83467400
C	-2.95543600	1.96010400	-0.51831600
H	-4.27126600	0.65477300	0.58616800
H	-1.39900500	3.08226200	-1.50261500
H	-3.75834700	2.55167800	-0.94774400
C	0.82656600	1.85600000	-0.68328300
C	1.62988700	0.85169000	-1.24448800
C	1.37788400	3.14745700	-0.50703100
C	2.93563200	1.11297200	-1.65484000
H	1.21263400	-0.14386000	-1.35394500
C	2.68161100	3.40226900	-0.94838600
C	3.45927200	2.39714400	-1.51998900
H	3.53590700	0.31593600	-2.08326800
H	3.09719400	4.39584200	-0.81127000
H	4.47323000	2.61578300	-1.84078300
C	0.70763700	4.31261400	0.21375700
O	0.84964200	4.37811300	1.55956900
O	0.18336600	5.22273500	-0.39102600
C	4.77986400	0.85156900	1.67376500
C	3.40231100	0.92146300	1.91198200
C	2.87495600	2.17642700	2.15772700
C	3.61650800	3.34805600	2.19729000
C	4.98859900	3.24401600	1.96127400
C	5.56756400	2.00133300	1.69678000

H	5.22591600	-0.11621300	1.46600200
H	2.78343600	0.03376900	1.90691500
H	3.14484400	4.30906400	2.36298600
H	5.59794000	4.14260400	1.98012700
H	6.63475600	1.93046600	1.50992900
I	0.72443700	2.38499800	2.48068500

E

C	-0.28198600	0.29333600	2.48364200
O	0.49485300	-0.06036300	3.51216500
O	-0.79248600	-0.55686500	1.77842000
C	-0.55172300	1.76454300	2.28038200
C	-1.34090600	2.41131400	3.24089900
C	-0.05572100	2.48365800	1.17587300
C	-1.67557800	3.75583200	3.10668700
H	-1.69048500	1.84665000	4.10057300
C	-0.41679400	3.83537100	1.04920400
C	-1.21655800	4.46993000	1.99714200
H	-2.29777500	4.23946100	3.85357200
H	-0.05226300	4.39355800	0.19159300
H	-1.48536000	5.51429100	1.86558600
C	0.87560400	1.89865200	0.16859500
C	0.50233400	0.77737200	-0.58473100
C	2.10972400	2.52404600	-0.12299900
C	1.33280100	0.28280600	-1.59038300
H	-0.44351500	0.29522500	-0.37197400
C	2.91974600	2.04135900	-1.15416000
C	2.54125200	0.91608500	-1.88173900
H	1.02287300	-0.59080200	-2.15669800
H	3.85192400	2.55501400	-1.36467900
H	3.18338400	0.53869400	-2.67222500
C	2.61006300	3.71196200	0.64579500
O	2.46276000	3.54105100	1.96123600
O	3.12318700	4.68453100	0.12130400
C	3.82901700	-0.72023100	0.92224900
C	2.92946100	-0.33820200	1.92310800
C	3.36009200	0.61783600	2.82765600
C	4.62111800	1.18766400	2.81399400
C	5.49813500	0.78541500	1.80351800
C	5.10266000	-0.15872600	0.85583100
H	3.51106800	-1.45441900	0.18967400
H	1.94603100	-0.78245800	1.97679100
H	4.91578100	1.92105700	3.54829900
H	6.49402200	1.21771700	1.76793600
H	5.78790300	-0.45899000	0.06910100
I	1.95033700	1.33695800	4.34910200
C	1.87886600	6.68368800	5.96177300
O	1.93230100	6.91454900	4.64617700
O	2.15050300	7.56804700	6.75231300
C	1.40775500	5.32411000	6.41719300
C	0.06594500	4.99671000	6.18016700
C	2.25312700	4.40355000	7.06599700
C	-0.46238600	3.78209800	6.60826000
H	-0.56274900	5.70756500	5.65150200
C	1.69851800	3.18960800	7.50445800
C	0.35915800	2.87628600	7.28375500
H	-1.50710900	3.54848100	6.42717700

H	2.33991500	2.47998800	8.01920600
H	-0.04151200	1.93260300	7.64317000
C	3.71555600	4.62625100	7.25581000
C	4.18460400	5.73100100	7.97921300
C	4.65484800	3.67262200	6.80026900
C	5.54642200	5.89267500	8.23416800
H	3.47184400	6.46374500	8.33602100
C	6.01341600	3.82598200	7.08864400
C	6.46424200	4.93847100	7.79433000
H	5.88527000	6.76046300	8.79277600
H	6.70617100	3.06708400	6.74039900
H	7.52364600	5.05765100	8.00200200
C	4.25358600	2.47182300	5.99428900
O	3.39687100	2.79740400	5.02431100
O	4.69697800	1.35362700	6.18783600
C	6.17807600	6.43061800	4.64713500
C	4.79767400	6.39399300	4.42354500
C	4.32747700	5.44358000	3.53286000
C	5.13693200	4.56000000	2.84065100
C	6.51144400	4.62121600	3.08359700
C	7.03089400	5.54673700	3.98885000
H	6.57157600	7.15343200	5.35386400
H	4.14070700	7.08695800	4.92898400
H	4.72496100	3.84409800	2.14656700
H	7.17029800	3.93813300	2.55515700
H	8.09944900	5.58137300	4.17771100
I	2.16016100	5.26603300	3.23079200

E-1

C	-0.26698800	0.62573300	2.68687300
O	0.50943000	0.41956000	3.75239800
O	-0.81318100	-0.31246400	2.13655600
C	-0.49514500	2.04873000	2.24361500
C	-1.19013400	2.89969000	3.11462300
C	-0.05668900	2.52670700	0.99523000
C	-1.48984000	4.20891600	2.75057500
H	-1.49201200	2.52337600	4.08807500
C	-0.38993600	3.84236800	0.63295400
C	-1.09797900	4.67808800	1.49327500
H	-2.03097200	4.85569200	3.43443000
H	-0.06962800	4.21174800	-0.33746800
H	-1.34680900	5.68932300	1.18341500
C	0.80950700	1.72918300	0.07931500
C	0.35885300	0.51662600	-0.45894100
C	2.06617100	2.22309100	-0.33944500
C	1.13399700	-0.19065400	-1.37765500
H	-0.60116800	0.12970500	-0.14258200
C	2.82004200	1.52481800	-1.28795800
C	2.36404100	0.31444300	-1.80077000
H	0.76453100	-1.13216200	-1.77373600
H	3.77179300	1.94179100	-1.59847900
H	2.96249600	-0.22970900	-2.52556500
C	2.65798400	3.48924600	0.19449300
O	2.43281200	3.64196500	1.49848900
O	3.30071200	4.26506800	-0.49835000
C	3.92285300	-0.47645700	1.34146400
C	3.02708200	-0.03790700	2.32262800

C	3.36007900	1.12059100	3.00248100
C	4.50964900	1.85772900	2.78402500
C	5.38328900	1.39584400	1.79786100
C	5.09150200	0.23613300	1.07808100
H	3.68463400	-1.37306900	0.77872500
H	2.11484200	-0.58116000	2.53014700
H	4.71959300	2.76048800	3.33850200
H	6.28887300	1.95981000	1.59761100
H	5.77442000	-0.11009900	0.30821800
I	1.94320700	1.89468300	4.48665900
C	1.73063200	6.09618500	6.10031500
O	0.86271500	6.54884700	5.18159500
O	2.81843000	6.61426600	6.28685300
C	1.25135000	4.88353500	6.81923900
C	-0.05309600	4.41674000	6.60740900
C	2.14388300	4.13997200	7.61928600
C	-0.48912400	3.22661000	7.18592800
H	-0.71240300	4.99146200	5.96817100
C	1.68939000	2.94631300	8.19412500
C	0.38817700	2.48837300	7.98263600
H	-1.50321200	2.87694200	7.01596500
H	2.37727100	2.36535900	8.80078200
H	0.06477000	1.55485800	8.43371400
C	3.57328700	4.51445500	7.85673900
C	3.90488700	5.38840700	8.89727300
C	4.61221100	3.88694600	7.14456900
C	5.23578100	5.65942000	9.20513300
H	3.10674400	5.86528600	9.45757100
C	5.94819700	4.13336800	7.48139000
C	6.26375700	5.03078100	8.49670700
H	5.47177700	6.35473300	10.00551000
H	6.72562300	3.61020800	6.93388000
H	7.30203700	5.22981500	8.74549000
C	4.31915800	2.90132100	6.05726000
O	3.37479300	3.34438600	5.21661000
O	4.88539700	1.82771400	5.95819700
C	6.21633800	5.80404900	4.33628400
C	4.86011700	5.93486600	4.02339100
C	4.47111000	5.55028900	2.75005800
C	5.32858000	5.06602200	1.77305200
C	6.67875500	4.95000100	2.11773100
C	7.11828600	5.30689700	3.39433900
H	6.54806100	6.07812700	5.33237200
H	4.15820500	6.32110200	4.75206000
H	4.96449800	4.78757400	0.79091000
H	7.38282800	4.58200300	1.37701600
H	8.16759500	5.20103500	3.65326200
I	2.34834800	5.60831000	2.31624800
F	2.34600800	7.47087400	3.28802400
H	1.37590300	7.16501000	4.58745800

E-2

C	-0.40652600	1.41121200	1.92691100
O	-0.56110100	1.66891000	3.23425100
O	-0.02768800	0.33819900	1.49402600
C	-0.72685200	2.59916600	1.08220700
C	-1.48641000	3.64746500	1.62128700

C	-0.19716000	2.71426500	-0.21770100
C	-1.73678600	4.80329900	0.88423300
H	-1.86447200	3.54907500	2.63194100
C	-0.45014900	3.88725800	-0.94226700
C	-1.21362300	4.92322900	-0.40408000
H	-2.32889600	5.60490300	1.31535100
H	-0.02403700	3.98668500	-1.93588500
H	-1.39117900	5.82216500	-0.98708600
C	0.70646000	1.70957600	-0.85912100
C	0.18907700	0.61484300	-1.55731500
C	2.09046200	1.95269000	-0.91420000
C	1.02875600	-0.22659400	-2.28402700
H	-0.87821400	0.42355800	-1.51875400
C	2.92590400	1.13361000	-1.68055900
C	2.40007400	0.03590400	-2.35498500
H	0.61096900	-1.08196100	-2.80696000
H	3.98483000	1.36556100	-1.72949900
H	3.05169900	-0.60881100	-2.93737600
C	2.68450500	3.10454500	-0.16699800
O	2.30420500	3.09314900	1.11212600
O	3.43246900	3.92349400	-0.67638500
C	3.50514800	-0.05836000	1.53581600
C	2.78321500	0.21361400	2.70051700
C	3.32948900	1.13647100	3.57397800
C	4.52833800	1.79817700	3.38106200
C	5.23569900	1.48672500	2.21587400
C	4.72811200	0.56778600	1.29854600
H	3.08479000	-0.74791600	0.81155600
H	1.83112800	-0.25920700	2.88923800
H	4.91167600	2.52165100	4.08453900
H	6.18233500	1.98423000	2.03436200
H	5.28241300	0.34599800	0.39175500
I	2.15185900	1.61661100	5.36506000
C	1.65694100	5.50931800	6.51274600
O	0.77337600	5.41877900	5.51036300
O	2.56713000	6.32039100	6.53385500
C	1.42885400	4.48648700	7.57305200
C	0.18893000	3.84260300	7.67038700
C	2.48931800	4.10424200	8.41834900
C	-0.01721100	2.82926900	8.60510300
H	-0.60528700	4.13740600	6.99446000
C	2.26794200	3.08138400	9.34850400
C	1.02821700	2.44708900	9.44640400
H	-0.98403600	2.33949800	8.67123500
H	3.08852200	2.77235600	9.98888100
H	0.88457200	1.65319600	10.17351000
C	3.87369400	4.66919500	8.34342300
C	4.24831800	5.71029600	9.19842300
C	4.86493500	4.06198200	7.54882500
C	5.56719700	6.15733700	9.24698100
H	3.49064600	6.17526600	9.82115600
C	6.19687100	4.48341900	7.63250500
C	6.54843100	5.54056800	8.46647200
H	5.83202200	6.98039700	9.90435600
H	6.94227900	3.97322800	7.03078900
H	7.58024800	5.87537300	8.51679400
C	4.53695400	2.92892900	6.63195900

O	3.45047200	3.20392400	5.88721500
O	5.18652200	1.90481300	6.54885100
C	5.65155000	5.75330100	4.39115100
C	4.31149300	5.64501600	4.00766400
C	4.06082600	5.16384700	2.73305100
C	5.03690900	4.80672600	1.81725300
C	6.36708900	4.92841600	2.23179200
C	6.67339500	5.38643000	3.51450000
H	5.87496600	6.11755700	5.38808200
H	3.52974000	5.95453800	4.68684300
H	4.77961600	4.45793300	0.82419000
H	7.16018900	4.66490500	1.53790400
H	7.71039700	5.46836200	3.82676300
I	1.96483000	4.90196200	2.18395600
F	1.63453700	6.60642400	3.37989300
H	1.05500200	6.04243000	4.78160100
F	0.82033200	0.09714200	4.73969500
H	-0.19492800	0.90338600	3.75809500

E-3

C	-0.69797700	1.13867400	2.40764500
O	-0.88416200	1.05506400	3.73694300
O	-0.27765700	0.20368600	1.74623500
C	-1.01582900	2.47956800	1.84835600
C	-1.57722700	3.46519300	2.66641500
C	-0.64978900	2.79296400	0.52306500
C	-1.79471200	4.75481200	2.18716600
H	-1.82351500	3.21287000	3.69021600
C	-0.88606200	4.08755400	0.05091200
C	-1.44867100	5.06591300	0.87336600
H	-2.21867400	5.51123000	2.84023700
H	-0.60503600	4.33142500	-0.96944200
H	-1.60857400	6.06833900	0.48652500
C	-0.01427000	1.82542500	-0.42590100
C	-0.82439900	1.22234400	-1.39261200
C	1.37696300	1.58830600	-0.47389000
C	-0.28525400	0.38005000	-2.36393600
H	-1.89290200	1.41257400	-1.36918400
C	1.91440600	0.75487100	-1.46380900
C	1.08941600	0.14288700	-2.40180100
H	-0.94030900	-0.08693100	-3.09407200
H	2.98519300	0.59015200	-1.47745200
H	1.51479800	-0.50899200	-3.15889900
C	2.27675200	2.19170900	0.53443200
O	1.88136900	2.66875400	1.59572500
O	3.56954900	2.18712600	0.19923700
C	3.39301400	-1.08524700	1.16877000
C	2.69411200	-0.54470200	2.25173000
C	3.42102600	0.23355700	3.13884000
C	4.77152600	0.51631400	3.02311200
C	5.43868900	-0.04063800	1.92693200
C	4.75581900	-0.83514500	1.00575300
H	2.84979800	-1.68679800	0.44678900
H	1.63279900	-0.71361400	2.37431300
H	5.29145600	1.11942100	3.75598400
H	6.50002300	0.15411200	1.80454800
H	5.28610800	-1.25562400	0.15637200

I	2.30493100	1.09502800	4.80217000
C	1.36276900	5.45667500	5.67077300
O	0.51911000	5.37890400	4.62695600
O	2.12691400	6.39078200	5.83350500
C	1.27885100	4.28026100	6.57876500
C	0.11275600	3.50562000	6.60916200
C	2.40121800	3.90050900	7.34501800
C	0.02863700	2.37450200	7.41880600
H	-0.72017100	3.79032100	5.97727100
C	2.30160100	2.75831400	8.14961000
C	1.12800300	2.00326200	8.19547500
H	-0.88112000	1.78241400	7.43175500
H	3.16671300	2.45279700	8.73038100
H	1.07965400	1.11947500	8.82498500
C	3.72464500	4.59782700	7.28813500
C	3.95699800	5.73345800	8.07064400
C	4.79604200	4.05706000	6.55067200
C	5.21201100	6.33520900	8.10193500
H	3.13589400	6.15140100	8.64329600
C	6.06283000	4.65011300	6.60546000
C	6.26915400	5.79550600	7.36504200
H	5.36583400	7.22627700	8.70392800
H	6.87109600	4.20386600	6.03673800
H	7.24788800	6.26460400	7.38483300
C	4.63332800	2.80922500	5.74020700
O	3.52519600	2.83619600	4.99851300
O	5.44110500	1.89433300	5.76243300
C	5.58072000	7.95300300	4.65535300
C	4.51235000	7.21838700	4.13499900
C	4.82054000	6.10288300	3.37257000
C	6.11466500	5.67657600	3.10869900
C	7.16299100	6.43386300	3.63706100
C	6.89792800	7.56883600	4.40418500
H	5.37060400	8.82325900	5.26947300
H	3.48590500	7.48776800	4.34087200
H	6.29517700	4.78086700	2.53011100
H	8.18721300	6.12745100	3.44503600
H	7.71939300	8.14938300	4.81372600
I	3.17329200	4.94634700	2.57252500
F	1.97555600	6.61443000	2.84065400
H	0.82123800	6.06514300	3.97692300
F	0.99636900	-0.53674900	4.57679900
H	-0.39344800	0.24601900	4.04310900
F	4.58831600	3.45756600	2.29099300
H	4.07204100	2.56234300	0.97160400

E-4

C	-0.29902000	1.81963600	2.47518300
O	-0.37154400	2.09830000	3.78772500
O	0.07354100	0.73220600	2.06436200
C	-0.70082500	2.94721700	1.59566000
C	-1.34075300	4.06744200	2.13761000
C	-0.40857500	2.90027800	0.21489900
C	-1.73632800	5.12279300	1.31882200
H	-1.51407900	4.10598300	3.20631600
C	-0.83584700	3.95424800	-0.59635200
C	-1.49652300	5.05681700	-0.05394800

H	-2.23191700	5.98761400	1.74949200
H	-0.61242700	3.92069700	-1.65847200
H	-1.80890800	5.87030900	-0.70277500
C	0.36695700	1.78634500	-0.41023000
C	-0.31134800	0.85690800	-1.20180100
C	1.76078600	1.63076300	-0.23273000
C	0.35568900	-0.22784500	-1.77133200
H	-1.38175400	0.97313900	-1.34133200
C	2.41971500	0.53039600	-0.79223700
C	1.72234100	-0.40127500	-1.55523900
H	-0.19786400	-0.94366000	-2.37280100
H	3.48154200	0.41138400	-0.61643800
H	2.24421000	-1.25397500	-1.97857500
C	2.53111800	2.62264300	0.55533300
O	2.07968600	3.71334000	0.89211500
O	3.76662400	2.23248200	0.87575400
C	2.31498300	-2.39826400	1.62959000
C	2.16522800	-1.34780100	2.53894200
C	3.26095400	-0.52633800	2.74453800
C	4.48141100	-0.68131900	2.10510400
C	4.60426700	-1.74366000	1.20594700
C	3.52642000	-2.59817700	0.96809500
H	1.46882800	-3.05003900	1.43489100
H	1.22491800	-1.15516600	3.03406500
H	5.29145800	0.01245500	2.28045300
H	5.54814800	-1.89297500	0.68963300
H	3.63012300	-3.41728900	0.26242900
I	3.01437600	1.15763900	4.09681400
C	1.31831000	5.47140800	6.02696100
O	0.50850900	5.44467000	4.95448200
O	2.01139900	6.43486100	6.30548400
C	1.29846100	4.21129300	6.81671700
C	0.25137100	3.29859200	6.66308200
C	2.38808200	3.90209200	7.65583100
C	0.26203700	2.08249800	7.34418400
H	-0.55312000	3.53534600	5.97869200
C	2.37754600	2.68413100	8.34331000
C	1.32613100	1.77752300	8.19119400
H	-0.55067900	1.37611500	7.20677800
H	3.21844900	2.43854500	8.98541700
H	1.34751700	0.83200100	8.72548200
C	3.59464900	4.77319500	7.82512400
C	3.71874200	5.52674500	8.99534800
C	4.68212600	4.75719200	6.92176600
C	4.86762600	6.27336500	9.25479400
H	2.89413700	5.53220700	9.70120300
C	5.84372100	5.48689100	7.20090000
C	5.93467400	6.25617000	8.35597000
H	4.92958100	6.86453300	10.16426100
H	6.66027800	5.45830200	6.49034400
H	6.83181900	6.83469500	8.55465500
C	4.58962000	3.98858500	5.66223100
O	3.51889500	3.69405700	5.13724700
O	5.75555000	3.62469900	5.12327300
C	5.04558000	8.48589200	5.34307900
C	4.08313500	7.75968300	4.63721200
C	4.53380500	6.79557000	3.74950400

C	5.87609000	6.52116600	3.52435500
C	6.81713900	7.26976000	4.23538600
C	6.40472200	8.24893600	5.14043600
H	4.71877100	9.23123600	6.06143900
H	3.02748500	7.91599100	4.80133300
H	6.17489400	5.74214600	2.83724900
H	7.87436500	7.07664300	4.07807600
H	7.14361500	8.82113700	5.69380100
I	3.05372700	5.67804100	2.62309300
F	1.66643400	7.08898100	3.23719700
H	0.74752100	6.22777900	4.39490700
F	1.26151300	0.27177900	4.75480100
H	0.05208000	1.33153900	4.25493800
F	4.62367100	4.39577500	2.19548400
H	4.18060300	2.94840000	1.42659900
F	4.85128700	1.80325700	3.38743500
H	5.52985400	3.09738400	4.31241100

F

C	-0.99704000	-0.68244000	0.87582800
O	-0.04542700	-0.19734900	1.69413700
O	-1.19545900	-1.87142000	0.76569000
C	-1.80957700	0.35019600	0.14392000
C	-3.20189600	0.23347300	0.19181700
C	-1.21831100	1.41028400	-0.56482100
C	-4.01446200	1.18972000	-0.41168500
H	-3.63251400	-0.61196100	0.71857800
C	-2.04512500	2.35901200	-1.17772200
C	-3.43318000	2.26118400	-1.09017200
H	-5.09506000	1.09846100	-0.35468300
H	-1.58960000	3.16849400	-1.74103700
H	-4.05760800	3.01161500	-1.56636200
C	0.25551200	1.49311700	-0.81368600
C	0.78962200	0.74271900	-1.86546600
C	1.10829000	2.38020800	-0.12643600
C	2.12933600	0.86890400	-2.23275400
H	0.13727500	0.06189400	-2.40297600
C	2.44495300	2.53078300	-0.51920000
C	2.95746600	1.77303000	-1.56755900
H	2.52191300	0.26726700	-3.04720200
H	3.07937800	3.22538800	0.02019600
H	3.99786900	1.88163200	-1.85760800
C	0.63080200	3.07075900	1.09228500
O	1.18061700	4.28525000	1.28113600
O	-0.15894400	2.59602300	1.89749700
H	-0.13927800	0.77980700	1.78709200
H	0.84812000	4.60522600	2.14147300

G

C	-0.89667600	-0.80891500	1.02670200
O	0.44809300	-0.93141100	0.81434900
O	-1.51777100	-1.69199200	1.57793700
C	-1.47325000	0.49179900	0.58968000
C	-2.67838400	0.82686100	1.22584400
C	-0.88075300	1.40251900	-0.31630400
C	-3.28861600	2.05506800	1.00794200
H	-3.10500600	0.10666200	1.91427900

C	-1.51344000	2.63878100	-0.51872600
C	-2.69432800	2.97007200	0.13821200
H	-4.21365600	2.30093200	1.52007300
H	-1.06942100	3.34229600	-1.21546800
H	-3.15288700	3.93851100	-0.03829800
C	0.32283200	1.11421600	-1.14581500
C	0.34509400	-0.01895900	-1.97207400
C	1.40150200	2.02102100	-1.21762800
C	1.41286700	-0.26597100	-2.82962300
H	-0.49812500	-0.70229600	-1.94530600
C	2.45563600	1.78399900	-2.10937500
C	2.47538000	0.63832900	-2.90017900
H	1.40805500	-1.15420100	-3.45463000
H	3.25755900	2.51331700	-2.16955800
H	3.30521400	0.45861000	-3.57721800
C	1.48957000	3.26922100	-0.36924200
O	1.38564900	3.11653100	0.94837900
O	1.67265100	4.36040100	-0.87884800
H	0.71897800	-1.76600200	1.24310800
O	2.75507900	-0.45192300	2.83661800
H	2.27027800	-0.46046900	3.68586800
C	5.58767600	2.51642600	4.09813400
C	4.58307800	1.75873100	3.49216700
C	3.59105800	2.43562400	2.79283000
C	3.53921200	3.82017400	2.68160000
C	4.55240000	4.55611100	3.30079100
C	5.57020300	3.90903200	4.00389300
H	6.37965000	2.01327800	4.64489800
H	4.56808700	0.67640500	3.55431900
H	2.74483600	4.30082700	2.12326600
H	4.53868900	5.63949600	3.22940500
H	6.35338100	4.49226900	4.47920400
I	2.02282400	1.22851700	1.88021000
O	0.62410600	1.33932100	3.07308900

H

C	-0.52670200	0.40927100	1.50141200
O	0.53058100	-0.34910000	1.80541000
O	-1.65611300	0.03147400	1.74403400
C	-0.27233100	1.79810000	0.94976700
C	-0.71305100	2.85462700	1.75654100
C	0.40971400	2.07677700	-0.25674000
C	-0.42997800	4.17482900	1.42190500
H	-1.25295300	2.62065700	2.66784300
C	0.68040200	3.41764800	-0.58251400
C	0.28077000	4.45591000	0.25279300
H	-0.75726400	4.98013000	2.07231100
H	1.19484700	3.63622700	-1.51287500
H	0.50664000	5.48259200	-0.01913200
C	0.78048500	1.01354800	-1.23503900
C	-0.17800400	0.05928500	-1.61048400
C	2.06131800	0.96855600	-1.83767800
C	0.11398200	-0.91113800	-2.56673600
H	-1.16148600	0.09435500	-1.15244000
C	2.32786000	0.01651400	-2.82739800
C	1.36494600	-0.92627900	-3.18621800
H	-0.64058300	-1.64320800	-2.83746900

H	3.30705000	0.01135100	-3.29588800
H	1.59248200	-1.67053600	-3.94327600
C	3.21794900	1.85490500	-1.41655800
O	3.64387000	1.75235400	-0.15469300
O	3.76427800	2.59550300	-2.21080500
C	2.70761600	4.16899500	3.19696100
C	2.82834800	3.17448600	2.22077700
C	2.39716800	1.90521800	2.56358500
C	1.88401900	1.55395100	3.80623500
C	1.77276000	2.56721000	4.75812400
C	2.18150900	3.86852300	4.45274500
H	3.02889600	5.17922300	2.96297100
H	3.23608600	3.37916700	1.24000800
H	1.55279400	0.54460300	4.02060600
H	1.36644800	2.33426500	5.73751500
H	2.09046200	4.65041700	5.20060200
I	2.56988700	0.25852800	1.15652100
O	3.68134800	-0.77435700	2.18606500

I

C	-0.71184800	0.68994600	1.12715500
O	-0.78869800	0.89585400	2.43892300
O	-0.12718900	-0.26426000	0.62849000
C	-1.38066300	1.77553300	0.34294800
C	-2.40569300	2.51306100	0.94636600
C	-0.94413000	2.10685300	-0.95553100
C	-3.02356600	3.56748900	0.27817500
H	-2.71068000	2.24545500	1.95159800
C	-1.56932100	3.18014000	-1.60628600
C	-2.59586100	3.90648100	-1.00378300
H	-3.82490700	4.12166500	0.75835500
H	-1.22993200	3.44932600	-2.60195000
H	-3.05636200	4.73323700	-1.53753700
C	0.08213400	1.35897500	-1.74641300
C	-0.39523700	0.75679600	-2.91969000
C	1.46807500	1.28068400	-1.47050900
C	0.44951500	0.09035900	-3.80502400
H	-1.45951700	0.80834700	-3.12792000
C	2.30760300	0.61882000	-2.37869100
C	1.81459800	0.02596200	-3.53705100
H	0.03902100	-0.37095100	-4.69899800
H	3.36717900	0.59075600	-2.15691800
H	2.49043400	-0.47987500	-4.22050400
C	2.09659800	1.83072600	-0.21977600
O	3.39903700	1.62178700	-0.12021400
O	1.44301500	2.42094000	0.64395400
O	2.62639700	-0.63861700	4.60415300
H	3.19666200	0.15065900	4.66835800
O	4.69490800	-1.33634100	2.76069800
H	3.94982800	-1.36942800	3.41179000
C	5.36202700	2.23574700	4.90328500
C	5.19174100	1.26955600	3.90662500
C	4.40131500	1.60906400	2.81603100
C	3.79445000	2.83917600	2.64449100
C	3.98728900	3.78640200	3.65672500
C	4.75893900	3.48883500	4.77900200
H	5.97081700	2.00027100	5.77124100

H	5.63453200	0.28185500	3.97750200
H	3.15498100	3.03735900	1.79173100
H	3.50362500	4.75326000	3.56724500
H	4.88766600	4.23175100	5.56018100
I	4.08979400	0.02257600	1.33381400
C	0.65374300	4.21431400	4.47982900
C	0.59214800	2.99922700	3.79255600
C	0.95603500	1.85780900	4.49296800
C	1.39370500	1.86050600	5.80963000
C	1.45641600	3.09225900	6.46781800
C	1.08065700	4.26274900	5.80806900
H	0.37509600	5.12583800	3.95935000
H	0.28746600	2.94993400	2.75612900
H	1.68468100	0.94106500	6.30468300
H	1.79061700	3.12545300	7.50047600
H	1.12446500	5.21476000	6.32876500
I	0.90192500	-0.05013300	3.46059000

J

C	-0.62050800	0.69768200	1.10281300
O	-0.67902700	0.79904100	2.43801400
O	-0.29495700	-0.32825200	0.51899800
C	-0.87452800	1.99077500	0.38669900
C	-1.51977100	3.03245600	1.06851700
C	-0.37917200	2.20866300	-0.91798900
C	-1.71385600	4.27274100	0.46981500
H	-1.86135900	2.84753400	2.08030400
C	-0.57845300	3.46672500	-1.50154400
C	-1.23744400	4.48950500	-0.82367600
H	-2.22725100	5.06391700	1.00875900
H	-0.19436500	3.63866500	-2.50252000
H	-1.37453700	5.45418900	-1.30480800
C	0.28683700	1.17308800	-1.77454000
C	-0.53690100	0.48456000	-2.67350900
C	1.67726200	0.91694300	-1.80447600
C	-0.01698900	-0.43673600	-3.57976000
H	-1.60536000	0.67609200	-2.64780900
C	2.18767400	-0.01898400	-2.71564500
C	1.35489000	-0.68898300	-3.60555100
H	-0.68391200	-0.95674100	-4.26204300
H	3.25551300	-0.20424100	-2.70655500
H	1.77075500	-1.40447400	-4.30919000
C	2.61647400	1.55692400	-0.82517800
O	3.83451700	1.03513300	-0.76535200
O	2.23493200	2.46241500	-0.07949100
O	3.37418800	-0.49148600	3.29282700
C	5.37155200	3.26684000	4.04529300
C	4.90331800	2.05736900	3.52562900
C	4.70483100	1.99651000	2.15713200
C	4.95043600	3.03118300	1.27396100
C	5.42129400	4.22815100	1.82334700
C	5.62857100	4.34645700	3.19846600
H	5.52390500	3.35948200	5.11622300
H	4.66321100	1.20967200	4.15697600
H	4.75935500	2.91235200	0.21558100
H	5.61837100	5.06893100	1.16482700
H	5.98995100	5.28337500	3.61205000

I	3.95182600	0.10101200	1.37325200
C	1.92196700	4.17470100	3.38431500
C	1.63486700	2.88925400	2.92093600
C	1.58179800	1.86997300	3.86300600
C	1.79859800	2.06276200	5.22290000
C	2.08164200	3.35876300	5.65925400
C	2.14334700	4.40926500	4.74173300
H	1.98705700	4.98479100	2.66534000
H	1.49805600	2.71161600	1.86124900
H	1.74893400	1.23836900	5.92543600
H	2.24966500	3.54080500	6.71657700
H	2.37014900	5.41354800	5.08704100
I	1.22207400	-0.13083600	3.17447100

K

C	-1.08462700	-0.97348100	-0.99685000
O	0.22815500	-0.77204700	-1.20674200
O	-1.60360300	-2.04927700	-1.20403200
C	-1.83908700	0.19773300	-0.42886400
C	-2.70071600	-0.06662900	0.64144700
C	-1.69111100	1.51454300	-0.90239700
C	-3.37536600	0.96783200	1.28487000
H	-2.82137400	-1.09646200	0.96190800
C	-2.38415000	2.54395600	-0.25354300
C	-3.20649800	2.28007000	0.84122300
H	-4.02962400	0.75179400	2.12449300
H	-2.28166100	3.55880600	-0.62658600
H	-3.72697700	3.09655700	1.33376600
C	-0.94432200	1.85949400	-2.15449300
C	-1.61760600	1.71263900	-3.37313300
C	0.33791200	2.44273400	-2.16092600
C	-1.04063100	2.13460500	-4.57077900
H	-2.60955700	1.27117100	-3.37104100
C	0.89671000	2.89867400	-3.36051300
C	0.21581500	2.74125300	-4.56456300
H	-1.57909900	2.00024700	-5.50453400
H	1.87692500	3.36181400	-3.32838200
H	0.66441500	3.08495300	-5.49193900
C	1.18240000	2.51555400	-0.92557000
O	2.01553500	3.44434200	-0.79679700
O	1.03030400	1.55392000	-0.08266000
H	0.49998700	0.11045200	-0.84368600
H	2.58896100	3.34080000	0.60353400
O	2.86165300	3.13506100	1.58818300
C	6.47698000	2.16766500	3.84867600
C	5.41457000	2.17280900	2.94347700
C	4.42674500	1.20333400	3.08075800
C	4.46153500	0.23862800	4.08301700
C	5.53933200	0.23945600	4.97242400
C	6.54257900	1.20182800	4.85564600
H	7.25453600	2.92084200	3.76305600
H	5.33958300	2.92578100	2.16756100
H	3.66855700	-0.49641600	4.18851200
H	5.58247800	-0.50524300	5.76144100
H	7.37430200	1.20309600	5.55351000
I	2.80501900	1.14020000	1.65988500

L

C	0.13690000	-0.90195400	0.73003700
O	0.60598600	-1.06438700	1.97950600
O	0.39950700	-1.71330800	-0.13027900
C	-0.79196700	0.26671400	0.49495400
C	-2.04330200	0.17815000	1.11524000
C	-0.47463800	1.39808200	-0.29753200
C	-3.00443500	1.17452300	0.94558800
H	-2.26112900	-0.68326100	1.74019200
C	-1.46868600	2.37306400	-0.47854700
C	-2.71702100	2.27019000	0.13289400
H	-3.96995400	1.08666000	1.43438600
H	-1.25046200	3.23399000	-1.10123900
H	-3.45879800	3.04688500	-0.02664600
C	0.89766800	1.66940600	-0.80790000
C	1.63407300	0.66358200	-1.45718200
C	1.49730100	2.94318700	-0.65602300
C	2.93478100	0.88653900	-1.89889900
H	1.17145400	-0.30150300	-1.61288200
C	2.79482600	3.16233800	-1.13796100
C	3.52559600	2.13775600	-1.72949900
H	3.47813300	0.08275000	-2.38675300
H	3.22976000	4.15032100	-1.03028100
H	4.53901700	2.32305700	-2.07268500
C	0.85061200	4.18568900	-0.07700900
O	0.62020200	4.29957800	1.24849300
O	0.64261900	5.15131700	-0.78094600
C	4.56389000	1.01052000	1.85086900
C	3.19774400	1.12548500	2.12352700
C	2.69536600	2.40620800	2.28423500
C	3.45567200	3.56348900	2.20027300
C	4.81878400	3.41665500	1.93564200
C	5.36888300	2.14605700	1.75791700
H	4.98811700	0.02220600	1.70414900
H	2.56888000	0.24804200	2.19496400
H	3.00690100	4.54426000	2.30476300
H	5.44379700	4.30172100	1.86596600
H	6.42795500	2.04135000	1.54343300
I	0.56605800	2.64771800	2.56962100
F	0.67135800	0.88619500	3.71094600
H	0.42049900	-0.31119600	2.60047900

M

C	-1.31439200	-0.62468300	0.49894800
O	-2.15445600	-1.68952300	0.49221400
O	-0.25088500	-0.70233500	1.07327300
C	-1.82086700	0.60208100	-0.19254300
C	-3.11933800	1.04266500	0.10641500
C	-0.99040700	1.35982700	-1.04300500
C	-3.59952900	2.24047600	-0.41540400
H	-3.73600500	0.46671200	0.79184500
C	-1.49953200	2.55539400	-1.56906200
C	-2.78188900	2.99782800	-1.25531500
H	-4.59814600	2.58250200	-0.16209400
H	-0.87483200	3.13927100	-2.23740700
H	-3.14368500	3.93319700	-1.67134600
C	0.35549700	0.89310900	-1.47208800

C	0.49757500	-0.38361800	-2.03283600
C	1.48066200	1.74154300	-1.43190900
C	1.72231100	-0.81850200	-2.53246900
H	-0.36979500	-1.03496900	-2.09177000
C	2.69689900	1.31400200	-1.97479100
C	2.82671100	0.03528800	-2.51027800
H	1.80906000	-1.81601500	-2.95273500
H	3.53972700	1.99741500	-1.95447900
H	3.78125800	-0.29271500	-2.91066900
C	1.44613500	3.10417900	-0.79191400
O	0.86152100	3.18855300	0.40179900
O	1.92630300	4.07902400	-1.34596300
C	4.19511900	3.89988000	4.45960000
C	3.33038400	2.93919300	3.92857000
C	2.64112900	3.26650100	2.77066300
C	2.76096700	4.48268900	2.11595000
C	3.63574300	5.42373500	2.66929300
C	4.34862500	5.13737600	3.83354700
H	4.74804000	3.67068400	5.36597400
H	3.19349700	1.97014800	4.39045300
H	2.20520200	4.69560700	1.21151500
H	3.75228900	6.38428800	2.17611900
H	5.02428300	5.87750700	4.25208700
I	1.31732000	1.72240300	1.96217400
H	-2.91576200	-1.49174400	-0.07928100
F	1.86661800	0.60603200	3.56344100

N

C	-2.04727300	-1.52306100	-0.44058000
C	-0.85413100	-1.03804100	0.06520300
C	-0.62452500	-0.72844200	1.39416000
C	-1.68680800	-0.92542100	2.28153000
C	-2.91220200	-1.41276600	1.82343700
C	-3.09077100	-1.70950700	0.47104400
H	-2.14450600	-1.74088900	-1.49658700
H	-1.54781000	-0.69466100	3.33360500
H	-3.73000900	-1.56166900	2.52212400
H	-4.04402600	-2.08858100	0.11461700
I	0.78259100	-0.73985100	-1.33317300
F	-0.49157200	-1.41589600	-2.76334100
F	1.85797500	-0.10077100	0.26718600
H	0.34092200	-0.35267400	1.70843100

M-1

C	-1.20416600	-0.46230100	0.66171600
O	-2.09526500	-1.35931500	1.06151900
O	-0.02300500	-0.51635300	0.99691100
C	-1.76647500	0.64462000	-0.16343100
C	-3.08592600	1.04783600	0.08465500
C	-0.96495700	1.37053800	-1.07552600
C	-3.61172800	2.18143200	-0.52866400
H	-3.68168400	0.47249500	0.78401300
C	-1.51578000	2.50643200	-1.68483800
C	-2.81770900	2.91793300	-1.40803400
H	-4.63091500	2.48990300	-0.31720900
H	-0.91500100	3.06066000	-2.39902800

H	-3.21398400	3.80588300	-1.89155000
C	0.38913700	0.92641300	-1.51223200
C	0.55525700	-0.37275000	-2.01289200
C	1.48935100	1.80809500	-1.56308100
C	1.77319400	-0.79500800	-2.53624300
H	-0.29337700	-1.04963600	-2.00376100
C	2.70026700	1.39045900	-2.13009400
C	2.85167200	0.09028200	-2.60093400
H	1.87561600	-1.80992500	-2.90845600
H	3.52126400	2.09847300	-2.18127300
H	3.80029500	-0.22907900	-3.02144100
C	1.44819600	3.20872600	-1.02468700
O	0.98613100	3.38616100	0.23262700
O	1.81168800	4.17119500	-1.66786800
C	4.06267600	3.74553300	4.54323300
C	3.19885300	2.85552000	3.89917100
C	2.57718800	3.29468500	2.73951200
C	2.76533800	4.55258500	2.18711600
C	3.63654100	5.42085000	2.85289700
C	4.28126800	5.02202100	4.02391200
H	4.56371600	3.42973100	5.45350100
H	3.01500800	1.86056600	4.28286100
H	2.26107000	4.84924500	1.27541600
H	3.80433600	6.41274900	2.44402500
H	4.95518900	5.70566800	4.53130500
I	1.25051600	1.89058600	1.71309600
H	-1.62443400	-1.95824800	1.71436300
F	1.58466400	0.57934900	3.37952200
F	-0.75165600	-2.70182300	2.83794400
H	0.15370600	-2.33050600	2.83972800
F	1.56574700	-1.82777400	2.98749500
H	1.56187800	-0.84879300	3.10870500

M-2

C	-1.30237800	-0.28789300	0.75330300
O	-2.27978000	-1.16621900	1.00929000
O	-0.18786900	-0.38959600	1.25309900
C	-1.72942500	0.82291400	-0.13658100
C	-3.00082500	1.37796300	0.06249900
C	-0.84585400	1.38503900	-1.08926200
C	-3.39271200	2.51641500	-0.63766900
H	-3.66827300	0.92249800	0.78552800
C	-1.26966800	2.52111600	-1.79371900
C	-2.51975800	3.09236900	-1.56121500
H	-4.37432900	2.94611900	-0.46710900
H	-0.62608100	2.93808400	-2.56177400
H	-2.81933400	3.97218000	-2.12177000
C	0.44108500	0.74260900	-1.47717400
C	0.46208400	-0.61470700	-1.82590000
C	1.63062000	1.48608200	-1.64858600
C	1.61715000	-1.21711700	-2.31830400
H	-0.45323300	-1.19232400	-1.74388500
C	2.77818100	0.88941600	-2.19048800
C	2.77867800	-0.46462700	-2.50891300
H	1.60234700	-2.27032400	-2.58026700
H	3.66563100	1.49492800	-2.34421400
H	3.67244600	-0.92655600	-2.91495800

C	1.74017900	2.92346300	-1.29934700
O	1.34320000	3.36461500	-0.04786700
O	2.15877100	3.80878700	-2.00132800
C	4.65099000	4.07306300	3.45367400
C	3.87165400	3.18174200	2.72074900
C	2.48396800	3.36506400	2.72901200
C	1.85353900	4.38851300	3.44569300
C	2.65630300	5.26750100	4.16845000
C	4.04598300	5.10958100	4.17209000
H	5.72994700	3.95847500	3.46180800
H	4.33149000	2.37660200	2.15877800
H	0.77582000	4.50499500	3.43269100
H	2.19572700	6.07562700	4.72712900
H	4.66230100	5.79833800	4.74091800
I	1.28812200	2.04926000	1.58738400
H	-1.93095700	-1.81731700	1.64934700

M-3

C	-1.71761800	-0.08511000	0.71958900
O	-2.40874500	-1.23624800	0.71212600
O	-1.12399500	0.24473200	1.73254200
C	-1.77801800	0.74143500	-0.52317800
C	-3.04722700	1.03056500	-1.04578200
C	-0.62034600	1.30463700	-1.10744300
C	-3.18927100	1.89713700	-2.12744700
H	-3.93143600	0.61210200	-0.57207500
C	-0.78753300	2.15762000	-2.20859100
C	-2.05372100	2.46254600	-2.70615800
H	-4.17746200	2.12839700	-2.51109600
H	0.09003600	2.56810600	-2.69658500
H	-2.14810400	3.13072700	-3.55603700
C	0.76090000	0.95236100	-0.67946600
C	1.13634200	-0.38691400	-0.50691500
C	1.75302300	1.94494400	-0.52742800
C	2.44996100	-0.73602800	-0.19560200
H	0.39529400	-1.16748900	-0.64939900
C	3.08201300	1.58989800	-0.27232700
C	3.43122900	0.25220200	-0.09058700
H	2.71295300	-1.78222000	-0.07348500
H	3.83452400	2.37072900	-0.21252100
H	4.46344500	-0.01716600	0.11038400
C	1.43461900	3.41015500	-0.57233000
O	0.57660500	3.91258200	0.36634200
O	1.89933300	4.19399000	-1.36273200
C	3.99478300	4.44491500	3.54526900
C	3.02464600	3.70243600	2.87371800
C	1.68751000	3.94401900	3.18464700
C	1.27960500	4.88413900	4.12895700
C	2.27068100	5.60417000	4.79876000
C	3.61970600	5.38552500	4.50778100
H	5.04420600	4.28152500	3.32224200
H	3.30647700	2.95093800	2.14630300
H	0.23004400	5.06303500	4.33449100
H	1.98298200	6.34432600	5.53853500
H	4.38339900	5.94991600	5.03288700
I	0.22939300	2.78572700	2.14646700
H	-2.77637600	-1.40344200	-0.17369400

C	4.76756000	1.46909700	5.01215700
C	3.82338600	2.03810600	5.86512500
C	2.48267700	1.74974100	5.65808700
C	2.08793800	0.90632900	4.60142800
C	3.07268200	0.31473900	3.78683000
C	4.41934000	0.59482600	3.98645500
H	4.14986800	2.70064500	6.65605000
H	1.73906200	2.18067200	6.31900200
H	2.77227400	-0.33601500	2.97103400
H	5.19366300	0.16742500	3.36197200
N	6.18438900	1.84546100	5.17276300
O	6.43571500	2.74841800	5.96755100
O	7.00665700	1.25095600	4.48215200
C	0.68857200	0.68913800	4.25593300
H	0.47577200	-0.12997700	3.57217700
C	-0.36509400	1.46971100	4.63387300
H	-1.37293900	1.19112700	4.34328800
H	-0.25498100	2.30427000	5.32056100

M-4

C	-1.99740700	0.17682300	0.22763000
O	-3.10933800	-0.56438200	0.11850000
O	-1.13998400	-0.06822900	1.06987300
C	-1.96016500	1.31784100	-0.71920700
C	-3.15037600	2.02557200	-0.94190100
C	-0.74986000	1.75114900	-1.30916500
C	-3.14798700	3.18966500	-1.70534600
H	-4.07039300	1.66645900	-0.49429800
C	-0.77635700	2.91651900	-2.08780500
C	-1.95452800	3.63842200	-2.27160600
H	-4.07142200	3.73771100	-1.86120600
H	0.13349800	3.24269100	-2.58094100
H	-1.94153200	4.53863800	-2.87791500
C	0.50885700	0.95747300	-1.25572600
C	0.49163700	-0.40041800	-1.60501000
C	1.75896400	1.55973900	-0.99468700
C	1.66852100	-1.14063500	-1.68353100
H	-0.45617600	-0.86752300	-1.85460400
C	2.94448500	0.82498200	-1.12092900
C	2.90136000	-0.52750700	-1.44757700
H	1.62578100	-2.18997200	-1.95817700
H	3.89355200	1.32394200	-0.95180100
H	3.82221700	-1.09521700	-1.53330500
C	1.88944300	2.99086900	-0.58978800
O	1.18100900	3.43081900	0.49685600
O	2.57623600	3.81226400	-1.14694600
C	3.75295800	3.78011300	4.52897300
C	3.02966500	2.93012900	3.69291100
C	1.71902200	3.28529100	3.38028300
C	1.10602400	4.44362800	3.86117700
C	1.85309400	5.27757400	4.69329800
C	3.16822800	4.94737600	5.02528300
H	4.77251400	3.52511000	4.79557600
H	3.47713600	2.02146900	3.30698300
H	0.09024800	4.70259900	3.58428100
H	1.40231800	6.18550400	5.08049900
H	3.73709400	5.59400600	5.68416500

I	0.62530600	2.06399800	2.02090900
H	-3.07365800	-1.25157800	0.81166900
C	3.45730500	3.80607500	8.49502500
C	2.10021000	4.09290100	8.32357400
C	1.29572700	3.27902500	7.52595600
C	1.86368100	2.17308400	6.88825700
C	3.21431600	1.85589400	7.08241900
C	4.00336200	2.66980500	7.87927300
H	1.67100400	4.95658700	8.81780900
H	0.24546600	3.51923600	7.41285100
H	3.62061300	0.97708400	6.59268600
H	5.05334200	2.44884900	8.03755300
O	5.55231900	4.44925200	9.44703600
C	4.36293600	4.65839100	9.31263300
O	3.71639900	5.69697400	9.87952900
C	4.53349700	6.55448100	10.69689400
H	4.97324700	5.98665200	11.52013100
H	5.33525400	6.99739600	10.10098400
H	3.86070100	7.32403900	11.07416500
O	1.18790400	1.34610000	6.02888500
C	-0.15704200	1.63441000	5.72385700
H	-0.81339100	1.43893000	6.58683400
H	-0.28076100	2.69706100	5.46260800
C	-0.58580500	0.78823200	4.56807300
H	-1.62127100	0.91208600	4.25447200
C	0.20645300	-0.10972200	3.94013400
H	-0.18205600	-0.72263800	3.13378900
H	1.22085300	-0.28216500	4.28408800

N-1

C	-1.57997700	-1.85882000	-0.30630100
C	-0.56877200	-1.06836100	0.20634800
C	-0.61497400	-0.37177600	1.40110800
C	-1.79534300	-0.47732100	2.14122500
C	-2.85611300	-1.25264000	1.67055200
C	-2.74524300	-1.94142900	0.46255100
H	-1.47709200	-2.42266500	-1.22466700
H	-1.87722900	0.05499600	3.08387400
H	-3.77051100	-1.32486900	2.25119800
H	-3.56397100	-2.55744500	0.10402100
I	1.27937500	-0.84767800	-0.92713100
F	0.09695300	-1.14570000	-2.69895200
F	2.19281300	-0.54669400	0.80893100
H	0.22989800	0.20805800	1.74968400
F	-1.59355400	0.66645400	-2.15646800
H	-1.05930100	-0.01381100	-2.54972100
F	-0.64300300	-3.53030900	-2.78179600
H	-0.34247800	-2.63213400	-2.92478500

N-2

C	-1.44115200	-1.79284800	-0.21064400
C	-0.55257900	-0.82666600	0.28959700
C	-0.77403600	-0.09666400	1.46911300
C	-1.95315000	-0.34653100	2.16189800
C	-2.86270000	-1.29946500	1.68450400
C	-2.61021300	-2.01666600	0.50756200

H	-1.23160500	-2.34359900	-1.12042800
H	-2.16104500	0.19979700	3.07582500
H	-3.77877600	-1.48540200	2.23602600
H	-3.32279000	-2.75293500	0.15103400
I	1.22630600	-0.48550700	-0.76146600
F	2.37470900	-1.79528000	0.10901700
H	-0.06010900	0.63512200	1.82935400

N-3

C	-0.74526200	-2.02921400	-0.67951100
C	-0.60316600	-0.85962200	0.06577300
C	-0.91173200	-0.76089900	1.42250500
C	-1.36938800	-1.91339300	2.06252700
C	-1.51020500	-3.10825300	1.35118300
C	-1.20645000	-3.16351200	-0.01176300
H	-0.50719000	-2.06637600	-1.73545300
H	-1.62017000	-1.87047700	3.11749400
H	-1.85291700	-4.00270300	1.86074100
H	-1.32886000	-4.09023600	-0.56309700
I	0.00891400	0.90468100	-0.96286900
F	-1.85735700	1.26169600	-1.54418100
H	-0.80897200	0.17158500	1.96571800
C	1.82256500	-4.43887600	0.53774800
C	1.80996400	-3.45271400	1.52369900
C	2.18109600	-2.16494100	1.17689100
C	2.54745600	-1.85924300	-0.15165900
C	2.60600400	-2.89718400	-1.10583800
C	2.24607100	-4.19548900	-0.76544700
H	1.49598300	-3.70656600	2.52761200
H	2.18348600	-1.39370700	1.93801400
H	2.91519900	-2.67451000	-2.12289900
H	2.26598500	-5.00518800	-1.48429700
N	1.31851000	-5.78378800	0.88292900
O	0.71528800	-5.88978300	1.94868000
O	1.51063400	-6.68065600	0.07087000
C	2.80443100	-0.50198700	-0.58865800
H	3.20367400	-0.39561300	-1.59667800
C	2.54420300	0.64559900	0.11383900
H	2.88709300	1.60399800	-0.26683400
H	2.23287000	0.62625200	1.15423900

N-4

C	-1.35391400	-3.47970200	0.08163000
C	-1.00756200	-2.51515300	1.03039200
C	-0.40392600	-2.80269200	2.25227700
C	-0.11768600	-4.14068000	2.52322300
C	-0.44049800	-5.13500100	1.59690600
C	-1.05490200	-4.80767500	0.38576100
H	-1.84596600	-3.21949200	-0.84844600
H	0.36289300	-4.40034500	3.45956000
H	-0.20414600	-6.16984300	1.81816700
H	-1.30821500	-5.58416200	-0.32854400
I	-1.60752700	-0.50299800	0.65311000
F	-3.40758500	-0.83591700	1.39269600
H	-0.15312800	-2.02604900	2.96465400

C	3.15136900	-5.84651500	1.38925200
C	2.69111900	-5.66021400	0.08283500
C	2.35153900	-4.38737100	-0.37740000
C	2.46814300	-3.29796500	0.48824500
C	2.96528500	-3.46632400	1.78624000
C	3.30261600	-4.73522000	2.23145100
H	2.60058900	-6.51447700	-0.57781100
H	2.00456600	-4.26807400	-1.39682600
H	3.07051200	-2.59571500	2.42527800
H	3.68293900	-4.89315400	3.23484000
O	3.86022200	-7.37525700	3.08181300
C	3.49747700	-7.18819700	1.93787800
O	3.34968500	-8.16713500	1.02517900
C	3.68556400	-9.49079200	1.48431600
H	4.73043800	-9.52666700	1.80066600
H	3.04740300	-9.77436200	2.32460900
H	3.51693300	-10.14573200	0.63027200
O	2.08936000	-2.01510200	0.17060600
C	1.51696800	-1.76923300	-1.08944200
H	2.26373200	-1.84119700	-1.89697300
H	0.73019000	-2.50488500	-1.32042000
C	0.92840000	-0.39714200	-1.09625300
H	0.47118900	-0.08505500	-2.03422900
C	0.98303900	0.45808600	-0.04545800
H	0.63014200	1.48195400	-0.13928900
H	1.51899500	0.18532300	0.85774100

TS1

C	0.10733900	-0.57816200	0.67191800
O	-0.04008300	-0.55207200	1.92201900
O	1.19889900	-0.67353900	0.03079000
C	-1.14458700	-0.43590200	-0.17062500
C	-2.25413900	-1.19350400	0.21472100
C	-1.24158100	0.45053300	-1.26394100
C	-3.45669600	-1.11715300	-0.48341400
H	-2.15513600	-1.84199300	1.07883400
C	-2.47111000	0.53495400	-1.93716700
C	-3.56501400	-0.24270800	-1.56380500
H	-4.30443300	-1.72327300	-0.17702900
H	-2.57178200	1.24415500	-2.75319300
H	-4.50092900	-0.15288200	-2.10793000
C	-0.13180200	1.31277200	-1.76626400
C	0.10763200	1.30826200	-3.15026800
C	0.69253500	2.14283200	-0.97212400
C	1.10154800	2.09144900	-3.73144200
H	-0.48872300	0.65049200	-3.77404200
C	1.70836400	2.90854500	-1.55768700
C	1.90881900	2.90195300	-2.93350000
H	1.25308300	2.05589900	-4.80655200
H	2.33525700	3.51267800	-0.90987800
H	2.69096100	3.51183500	-3.37531100
C	0.63895700	2.19501000	0.51725500
O	1.62854300	2.17809400	1.22514500
O	-0.63610700	2.24346500	0.96005600
O	-0.73291400	2.00666000	2.38265800
H	-0.63192600	1.00968300	2.36880200
O	3.00708600	-0.37629900	1.38073300

H	2.41550600	0.26419600	0.90599100
C	2.67942700	2.18748500	6.24088900
C	3.55156100	1.94441100	5.17583100
C	3.07161700	1.15367200	4.14231700
C	1.81130900	0.59369300	4.08102500
C	0.96459800	0.86114400	5.16328300
C	1.39156500	1.64833900	6.23231700
H	3.01465200	2.80343700	7.06992300
H	4.55075200	2.36717900	5.16989700
H	1.46676300	-0.00509000	3.24120800
H	-0.03859500	0.44642000	5.14977800
H	0.71893800	1.84785500	7.06078900
I	4.39726400	0.77305100	2.44559400

TS2

C	-0.58254700	0.00538900	1.66779000
O	-1.02982300	0.56477900	2.70160000
O	0.53605500	-0.61201900	1.58621500
C	-1.38367600	0.07195900	0.39930700
C	-2.68848700	-0.42226300	0.40127300
C	-0.85433000	0.66032900	-0.76415200
C	-3.46403000	-0.38955500	-0.75741100
H	-3.08740500	-0.84086500	1.32008200
C	-1.64608800	0.69409100	-1.91788500
C	-2.93831900	0.16759800	-1.92150700
H	-4.47296800	-0.79140800	-0.74723900
H	-1.24662000	1.15893500	-2.81463200
H	-3.53379800	0.20646300	-2.82910700
C	0.51608700	1.25280600	-0.83901600
C	1.42027100	0.63265900	-1.71425100
C	0.96090500	2.40343800	-0.13975700
C	2.71572000	1.10964400	-1.89640400
H	1.09214500	-0.25740900	-2.24127600
C	2.26602100	2.88071900	-0.34053700
C	3.14430300	2.24478900	-1.20688500
H	3.38904100	0.59193700	-2.57383500
H	2.57489700	3.76010300	0.20973100
H	4.15219500	2.62652300	-1.33553400
C	0.18787900	3.13624700	0.90841000
O	0.70049000	3.84829000	1.75212100
O	-1.13693100	2.87503100	0.82637100
O	-1.81363600	3.10004000	2.08457800
H	-1.76867300	2.15606200	2.41058100
O	1.84500400	-0.28528500	3.61757200
H	1.22003000	-0.53511700	2.68379800
C	5.04034800	3.77196100	2.01706800
C	3.82795000	3.48706100	2.65104100
C	3.49996700	2.15031900	2.82658100
C	4.28683000	1.09373700	2.40188900
C	5.49108100	1.40523500	1.76455900
C	5.86606300	2.73638400	1.57407200
H	5.32873800	4.80759100	1.86386500
H	3.15878400	4.28375200	2.95610600
H	3.96319300	0.06982500	2.55815100
H	6.13105400	0.60055900	1.41537800
H	6.80357100	2.96862100	1.07805300
I	1.58081500	1.65450200	3.69453600

TS3

C	-0.42552000	-0.03272200	1.24177400
O	-0.41180800	0.57092300	2.39081100
O	0.40874900	-0.87014200	0.88327700
C	-1.52983800	0.39541200	0.30376300
C	-2.85680700	0.09298700	0.61642000
C	-1.23365000	1.09884200	-0.87794000
C	-3.89247100	0.44528400	-0.24853400
H	-3.07097600	-0.43768100	1.54030200
C	-2.28124600	1.45013600	-1.73739800
C	-3.60211900	1.12481400	-1.43143800
H	-4.91932800	0.19126500	0.00011300
H	-2.05284200	2.00461400	-2.64328700
H	-4.40080200	1.41077700	-2.11015700
C	0.16380200	1.45614600	-1.25062100
C	0.68208600	0.93216100	-2.44252400
C	1.00268700	2.26962200	-0.45679100
C	1.98878300	1.19612400	-2.84760200
H	0.04729700	0.28609000	-3.04117500
C	2.32056300	2.51628800	-0.86411000
C	2.81614600	1.99158000	-2.05347100
H	2.36248500	0.77220900	-3.77555600
H	2.95401300	3.13404800	-0.23753500
H	3.83772600	2.20137300	-2.35727700
C	0.52626900	2.91687700	0.81651900
O	1.38589400	3.04319300	1.78925000
O	-0.64649800	3.32063700	0.88341000
O	-1.39142300	2.83709900	2.90898900
H	-1.05876000	1.90564000	2.54079200
O	3.33286800	-0.71928000	3.00987700
H	3.13313900	-1.32855900	2.27469000
C	6.63979000	1.99229100	2.64036900
C	5.41235800	1.32274300	2.64548600
C	4.26045400	2.05708300	2.39812000
C	4.28338300	3.42671900	2.15575000
C	5.52009200	4.07763000	2.15402500
C	6.69589800	3.36495600	2.39559700
H	7.55184000	1.43278800	2.82861500
H	5.34417600	0.25839700	2.84222700
H	3.36097200	3.96437700	1.97179900
H	5.55800200	5.14618700	1.96119900
H	7.65304200	3.87819700	2.39268700
I	2.35229500	1.01942600	2.46051900
C	2.41599800	3.62865600	5.05184900
C	1.04695400	3.52006600	4.79771500
C	0.37992000	2.44362100	5.36638300
C	0.99535400	1.46543400	6.13584000
C	2.36742300	1.59751200	6.37020800
C	3.07186600	2.67616000	5.83366500
H	2.97365000	4.44561600	4.60695200
H	0.54305600	4.20548200	4.12850500
H	0.44617400	0.61445500	6.52261300
H	2.88116300	0.84053500	6.95430100
H	4.14068600	2.76138400	6.00198900
I	-1.71914300	2.25370900	4.89287900

TS4

C	-0.34331800	-0.22280800	1.77890500
O	-0.86167700	-0.33467900	2.90852200
O	0.89629700	-0.50996200	1.53252500
C	-1.17248000	0.44590600	0.70952400
C	-2.56132800	0.27634300	0.80953300
C	-0.66004300	1.39716500	-0.20294800
C	-3.44329600	1.00868100	0.02343900
H	-2.93113600	-0.42562900	1.54847700
C	-1.56866600	2.17054500	-0.94828400
C	-2.94309800	1.97869100	-0.84652800
H	-4.51371600	0.84587800	0.10994900
H	-1.17561600	2.92474600	-1.62241800
H	-3.61768600	2.58429300	-1.44477400
C	0.78284200	1.63398800	-0.51129600
C	1.51851800	0.63277400	-1.16267200
C	1.38462400	2.89570800	-0.33759300
C	2.79325400	0.88115600	-1.66268300
H	1.06380900	-0.34401700	-1.28086800
C	2.66252600	3.14694800	-0.85714700
C	3.36401200	2.14838800	-1.52372900
H	3.33538500	0.08956000	-2.17211800
H	3.10425300	4.12933200	-0.72207200
H	4.35351700	2.35423000	-1.92031300
C	0.73224100	4.05298700	0.38027600
O	0.82262200	4.10872100	1.73831100
O	0.24124500	4.99501900	-0.19685600
H	0.80661400	-0.03775300	4.14112100
O	1.65472700	0.23850600	3.72469800
H	1.44725500	-0.29408000	2.72776100
C	5.04341500	1.16259200	1.25598800
C	3.70151100	1.15391000	1.64372400
C	3.22226100	2.28951500	2.27590600
C	3.97545200	3.43180500	2.51437900
C	5.30874600	3.41774900	2.10006000
C	5.84132500	2.28404000	1.48210000
H	5.45036000	0.29179600	0.75188800
H	3.05720000	0.31493100	1.41448900
H	3.54739800	4.30611000	2.99111000
H	5.92767400	4.29323300	2.27068100
H	6.88087200	2.27973900	1.16887900
I	1.15001900	2.33492700	2.85603600

TS5

O	1.38760500	1.17110700	3.93906300
O	3.71833100	-0.46750800	0.94447300
C	6.39258700	2.43498300	2.83256400
C	5.34828400	1.63881700	2.35286000
C	4.05451400	2.07308200	2.57561100
C	3.73860400	3.25636300	3.22450500
C	4.79384900	4.04543700	3.68689000
C	6.11492600	3.63112000	3.49926100
H	7.42197500	2.11997800	2.68479100
H	5.50177000	0.70054000	1.81981300
H	2.70641900	3.54306300	3.38683000
H	4.58075100	4.97328600	4.20975200
H	6.92985700	4.24435400	3.87282900

I	2.51789000	0.71264000	1.86227700
C	4.99790900	1.46952300	6.25297400
C	3.66522000	1.43212100	5.83916600
C	2.81494100	2.41261700	6.32239200
C	3.20844200	3.41961800	7.19120400
C	4.54776100	3.43590300	7.59456400
C	5.43723400	2.46792000	7.12455400
H	5.69225500	0.72561900	5.87529000
H	3.29061600	0.70255100	5.12882300
H	2.51429100	4.17747400	7.54036000
H	4.89102400	4.21459400	8.26949200
H	6.47749000	2.49620800	7.43445200
I	0.79384200	2.35554100	5.52553500

TS6

C	-0.64969200	-0.67974000	1.26244800
O	0.65259100	-0.67482400	1.04560900
O	-1.23018600	-1.50330100	1.95415200
C	-1.39488500	0.49599500	0.65678200
C	-2.63006800	0.78614700	1.24790500
C	-0.88103200	1.37584300	-0.32780900
C	-3.34340200	1.93440800	0.91823500
H	-2.99534100	0.09335200	1.99752200
C	-1.60968100	2.53513000	-0.64404800
C	-2.82209700	2.82213200	-0.02257000
H	-4.29326600	2.14199400	1.40225900
H	-1.22623000	3.20350600	-1.40880300
H	-3.36088600	3.72773200	-0.28550100
C	0.34021000	1.11110800	-1.14256900
C	0.46023400	-0.09583400	-1.84810000
C	1.34442800	2.08759000	-1.32315500
C	1.53247500	-0.33281400	-2.70077100
H	-0.31182900	-0.84699800	-1.72180600
C	2.40612300	1.85837500	-2.21192300
C	2.51019200	0.64832000	-2.88989400
H	1.60005100	-1.27914000	-3.22911000
H	3.14747500	2.63859800	-2.35582100
H	3.34111400	0.47431700	-3.56676400
C	1.37164300	3.40542900	-0.59480600
O	1.51679700	3.37868200	0.76063800
O	1.34811400	4.47831100	-1.15016300
H	1.47940600	-0.91961700	2.05561700
O	2.28103700	-0.53338400	2.76798100
H	1.84133100	-0.46836100	3.63762500
C	5.61073500	2.23573600	3.89369100
C	4.53911000	1.63851600	3.22598700
C	3.53541200	2.46793000	2.73596500
C	3.53648500	3.84964100	2.89367000
C	4.61650500	4.42370800	3.56667900
C	5.64655900	3.62101500	4.06275600
H	6.41097000	1.61448000	4.28421500
H	4.48528300	0.56198800	3.10210600
H	2.73565900	4.45865700	2.49034000
H	4.64800900	5.50046000	3.70233500
H	6.48154400	4.07841500	4.58482700
I	1.86235100	1.53869500	1.72373200
O	0.56007300	1.81902000	2.97904700

TS7

C	-0.33598200	0.95255900	1.08086200
O	-0.28077100	1.30498100	2.38596000
O	0.08069100	-0.11637600	0.67399600
C	-0.94773600	2.03712700	0.25998100
C	-1.80311800	2.95694500	0.87915900
C	-0.62032300	2.17465300	-1.10586300
C	-2.35961200	4.01284500	0.16297200
H	-2.02898900	2.83381600	1.93183300
C	-1.18456300	3.25269700	-1.80218600
C	-2.04011300	4.16400700	-1.18445600
H	-3.02826100	4.71202000	0.65619100
H	-0.93333200	3.37321600	-2.85129600
H	-2.45482800	4.98769300	-1.75891900
C	0.19761200	1.21978300	-1.91692800
C	-0.50971500	0.54669900	-2.92525400
C	1.59257100	1.01564100	-1.81941100
C	0.12413700	-0.31311600	-3.81950800
H	-1.58245300	0.70105000	-2.99585400
C	2.21653700	0.15626500	-2.73429000
C	1.50097500	-0.50526500	-3.72808600
H	-0.45720300	-0.82421200	-4.58208700
H	3.28765600	0.02525800	-2.63993800
H	2.01393400	-1.16577900	-4.42163300
C	2.45668700	1.62108700	-0.73405800
O	3.70223600	1.26197500	-0.75460600
O	1.94415500	2.35228400	0.13377400
O	2.93203600	-0.36997100	5.46667500
H	3.35081700	0.46328200	5.73634000
O	3.72814700	-0.91635600	3.07388300
H	3.49467000	-0.69094200	4.70928500
C	5.72264200	2.82842600	4.08538800
C	5.21075400	1.67421500	3.48811800
C	4.84819800	1.74328400	2.15260000
C	4.98417100	2.87812900	1.37121200
C	5.50514900	4.01963000	1.98866600
C	5.86595400	3.99879600	3.33757800
H	6.01149100	2.80923400	5.13251300
H	5.08825900	0.74482400	4.03333600
H	4.67078600	2.85768400	0.33374200
H	5.62092300	4.92969500	1.40674600
H	6.26319400	4.89445700	3.80621800
I	3.99732500	-0.08202700	1.31651500
C	2.40738400	4.38569000	3.65901800
C	2.07628000	3.11039900	3.19315700
C	1.72503200	2.16762800	4.15096400
C	1.67299100	2.41667700	5.51767000
C	2.01729600	3.69976800	5.95027300
C	2.38588700	4.67722700	5.02342300
H	2.69848600	5.14285900	2.93889600
H	2.09820500	2.88065400	2.12917300
H	1.36787700	1.65032400	6.22081800
H	1.98389300	3.93085400	7.01077500
H	2.65445300	5.67134300	5.36807100
I	1.22445900	0.21022600	3.40299700

TS8

C	-1.34607200	-0.95979500	-0.95168500
O	-0.01212100	-0.84990800	-1.07746400
O	-1.92341800	-2.00032500	-1.18207800
C	-2.05388800	0.26980000	-0.45133300
C	-2.98786300	0.09112200	0.57484000
C	-1.79703000	1.56003600	-0.94972400
C	-3.62927100	1.18449000	1.15146700
H	-3.19118400	-0.91899200	0.91557400
C	-2.45705800	2.64996700	-0.36903300
C	-3.35333700	2.47000100	0.68398600
H	-4.34064200	1.03473100	1.95839500
H	-2.27031300	3.64502800	-0.76228000
H	-3.84699100	3.33137400	1.12488500
C	-0.96179400	1.81775300	-2.16624400
C	-1.57212000	1.67624900	-3.41761900
C	0.35387900	2.31909000	-2.11397600
C	-0.90126900	2.02442200	-4.59002400
H	-2.58892700	1.29840500	-3.46205800
C	1.00993900	2.70199500	-3.29010000
C	0.38941100	2.55046800	-4.52704200
H	-1.39313200	1.89565000	-5.54986500
H	2.01585900	3.10052400	-3.21648300
H	0.91141600	2.83558300	-5.43552500
C	1.11588300	2.35993100	-0.83240200
O	2.00088700	3.27712300	-0.69853000
O	0.89792000	1.46165300	0.02976200
H	0.29702300	0.01340700	-0.70077400
H	2.50089100	3.18962100	0.39527200
O	2.89371900	3.07562300	1.54437300
C	6.34786400	2.43589000	4.12212200
C	5.20736400	2.40809400	3.31456600
C	4.52813200	1.20510600	3.19446300
C	4.92842600	0.03818900	3.83479000
C	6.07115800	0.08718900	4.63709100
C	6.77797800	1.28249800	4.78008600
H	6.89804600	3.36553900	4.23370200
H	4.84760800	3.28746300	2.79143600
H	4.37933500	-0.89287600	3.72322200
H	6.40387300	-0.81170200	5.14741400
H	7.66496400	1.31386000	5.40539900
I	2.76983300	1.14881000	1.93475000

TS9

C	0.01067800	-0.65085000	1.05215500
O	0.77049900	-0.21782300	2.02950800
O	0.02049700	-1.79992400	0.63488500
C	-0.97116100	0.35805700	0.47089200
C	-2.32222300	0.14725800	0.76436300
C	-0.59794100	1.50025800	-0.27583800
C	-3.29775000	1.06407300	0.37142900
H	-2.60162900	-0.74359700	1.31894500
C	-1.59127400	2.40937100	-0.67331800
C	-2.92972400	2.20363900	-0.34379900
H	-4.33980700	0.88534200	0.62006200
H	-1.30672400	3.27586800	-1.26270100
H	-3.68017100	2.92175800	-0.66029500

C	0.81008700	1.75399900	-0.67835900
C	1.55473500	0.71897500	-1.26400800
C	1.42336900	3.01979900	-0.53347400
C	2.85698100	0.92851200	-1.71235700
H	1.09294300	-0.25716200	-1.36992400
C	2.71755900	3.23256400	-1.02654200
C	3.43804000	2.19202300	-1.60614000
H	3.40977500	0.10644200	-2.15723800
H	3.16430000	4.21566400	-0.92106000
H	4.44745500	2.36765400	-1.96464700
C	0.82357800	4.21514600	0.16538200
O	0.63233900	4.19611600	1.52631700
O	0.62310800	5.27264700	-0.38219500
C	4.81337800	1.09630600	1.55203600
C	3.43712700	1.08211700	1.79764100
C	2.86711700	2.27105600	2.21847700
C	3.56256300	3.45743500	2.41347800
C	4.93290800	3.44123100	2.14753400
C	5.55496600	2.26539300	1.72092600
H	5.29613500	0.18409600	1.21545000
H	2.83232500	0.19443400	1.65659100
H	3.06487500	4.36598900	2.73189000
H	5.50943900	4.35143000	2.28158300
H	6.62204600	2.26269200	1.52072700
I	0.72789800	2.33827900	2.50939200
F	1.37718800	0.28989000	4.32876300
H	1.14644100	-0.25808200	3.55927700

TS9-1

C	-0.14323100	0.27109400	2.68146500
O	0.66080800	0.03693600	3.71849300
O	-0.60566800	-0.65356100	2.03800600
C	-0.50362100	1.70483900	2.38297400
C	-1.22542500	2.41299400	3.35322900
C	-0.14475200	2.33650200	1.17760300
C	-1.61766300	3.73060900	3.13704600
H	-1.46834600	1.92118400	4.29039800
C	-0.56791400	3.65941000	0.96586400
C	-1.29261900	4.35580100	1.93026300
H	-2.17706000	4.26443600	3.89877700
H	-0.30670400	4.14859700	0.03141100
H	-1.60624500	5.37802200	1.73914800
C	0.74723600	1.70391400	0.16422000
C	0.39143400	0.51091100	-0.47764100
C	1.93831600	2.34836200	-0.24155600
C	1.19744900	-0.02981000	-1.47934800
H	-0.51726600	0.00707200	-0.17549800
C	2.72550800	1.81829400	-1.26928200
C	2.36281500	0.62399300	-1.88316300
H	0.90212100	-0.96028200	-1.95531600
H	3.62569000	2.34746900	-1.56244300
H	2.98261600	0.20769100	-2.67156100
C	2.41606900	3.61238600	0.38926800
O	2.29402100	3.54820900	1.73209400
O	2.88639200	4.55306300	-0.21924200
C	4.05657600	-0.34859800	1.14820300
C	3.16169200	-0.08007100	2.18959300

C	3.43882700	1.01518800	2.98964900
C	4.53841200	1.84097300	2.83266800
C	5.41218300	1.55052600	1.78258600
C	5.17127600	0.46297700	0.94157700
H	3.86058900	-1.19253700	0.49492700
H	2.29204900	-0.70246400	2.35295000
H	4.70716800	2.68802900	3.48187400
H	6.27482600	2.19155700	1.62943800
H	5.85253300	0.24859300	0.12369900
I	2.00250400	1.55312200	4.55846200
C	1.98324000	6.39464500	5.92279100
O	1.36641900	6.81475500	4.84172000
O	3.00513500	6.91497900	6.37005700
C	1.37902600	5.17274200	6.56532400
C	0.05853300	4.82762300	6.24948500
C	2.13531100	4.32359600	7.40104000
C	-0.53067800	3.67286500	6.75834400
H	-0.49053400	5.48319700	5.58397200
C	1.53096800	3.16250800	7.90499600
C	0.21153500	2.83404300	7.59250500
H	-1.56076500	3.43084200	6.51229100
H	2.11547500	2.50486100	8.54210100
H	-0.23204200	1.93033200	8.00127100
C	3.57615900	4.53715200	7.74293500
C	3.94289900	5.49050900	8.70045900
C	4.57719300	3.69514400	7.21794600
C	5.26474800	5.61874100	9.11436100
H	3.17817000	6.14392100	9.10459600
C	5.90450300	3.81058600	7.65674600
C	6.25289600	4.77687800	8.59268300
H	5.52647600	6.37587300	9.84771500
H	6.64350600	3.12773500	7.25022300
H	7.28335100	4.87057400	8.92274200
C	4.27311900	2.63778800	6.20531700
O	3.36029900	3.04597600	5.30844200
O	4.81000600	1.54518400	6.18781900
C	6.33373000	5.46686200	4.34862200
C	4.94098700	5.49712900	4.24316000
C	4.40370700	5.43472900	2.96534200
C	5.15299800	5.37266400	1.79782500
C	6.54288100	5.33292600	1.93901600
C	7.12959000	5.37416900	3.20604600
H	6.77818500	5.50675800	5.33789300
H	4.33112000	5.58336600	5.13102800
H	4.67984500	5.33531500	0.82508900
H	7.16134500	5.27692500	1.04805700
H	8.21069500	5.34056400	3.30144500
I	2.24514700	5.34188700	2.80425500
F	2.83623500	7.88997500	3.30649500
H	2.23496900	7.70817200	4.10132800

TS9-2

C	-0.37177500	1.12035000	2.28767300
O	-0.39380500	1.18793000	3.60432100
O	-0.19118000	0.08043600	1.66204800
C	-0.59299900	2.44041700	1.60313500
C	-1.28673500	3.44266700	2.29476800

C	-0.07806400	2.71331400	0.31889500
C	-1.50955900	4.69515900	1.72700700
H	-1.63796200	3.22002800	3.29555100
C	-0.31557000	3.97709400	-0.24420700
C	-1.02650800	4.96056100	0.44321900
H	-2.05518300	5.45523400	2.27856400
H	0.08491000	4.19113500	-1.23097100
H	-1.19624800	5.92864000	-0.01987200
C	0.80937300	1.79205800	-0.45462600
C	0.31043800	0.64499500	-1.08104300
C	2.15120800	2.15395000	-0.68981300
C	1.12285600	-0.12545000	-1.90954800
H	-0.71721100	0.35588500	-0.90064500
C	2.95554400	1.39574500	-1.54909300
C	2.44789700	0.25007500	-2.15067000
H	0.71728200	-1.01847100	-2.37627700
H	3.97718500	1.71654100	-1.72330800
H	3.07607700	-0.34560300	-2.80665300
C	2.75817900	3.35500000	-0.04279100
O	2.41023000	3.44996700	1.24503300
O	3.50568200	4.12898600	-0.62111700
C	3.56751200	-0.42719800	1.52741100
C	2.78164800	-0.02053000	2.61063900
C	3.20897000	1.10738500	3.28562500
C	4.34315600	1.84113400	2.99077600
C	5.11823000	1.38851700	1.92086000
C	4.72648800	0.26845600	1.18632700
H	3.24881900	-1.28800000	0.94895900
H	1.88922400	-0.55700700	2.90051800
H	4.62190900	2.72707300	3.54332900
H	6.01841200	1.93636900	1.66133100
H	5.32286300	-0.05864800	0.34042300
I	1.90316000	1.89911400	4.86446200
C	1.67352100	5.85138800	6.28147700
O	0.81366500	6.02794100	5.27183500
O	2.68740300	6.51548700	6.42163800
C	1.29015400	4.72650100	7.18138500
C	-0.00036400	4.18453100	7.11821300
C	2.26104300	4.13355400	8.01414500
C	-0.34422800	3.06849300	7.87951300
H	-0.72330700	4.63810200	6.45047100
C	1.90080600	3.01227700	8.77151800
C	0.61130200	2.48110900	8.70944100
H	-1.34601100	2.65527400	7.81587000
H	2.65151600	2.54210800	9.39944900
H	0.36073000	1.60371100	9.29793700
C	3.69333600	4.56498900	8.07393800
C	4.11058200	5.52192800	9.00368800
C	4.67519100	3.89778900	7.31522400
C	5.46095200	5.82768400	9.15669200
H	3.36267700	6.03422300	9.59994300
C	6.03496100	4.17873000	7.49559100
C	6.42894000	5.15390500	8.40535400
H	5.76125100	6.58738900	9.87229900
H	6.76573100	3.62893700	6.91142100
H	7.48226500	5.38147400	8.53781200
C	4.29117700	2.83139000	6.34938400

O	3.28735000	3.28438500	5.53741800
O	4.78945300	1.73501800	6.26161400
C	5.90637000	5.82362400	4.42198500
C	4.56922800	5.86958500	4.01572900
C	4.28153200	5.40561400	2.74202500
C	5.22702900	4.92753000	1.84681800
C	6.55512400	4.89476700	2.28336500
C	6.89210700	5.32885000	3.56718600
H	6.15681500	6.16787000	5.41975700
H	3.81120900	6.26792500	4.67696100
H	4.94605400	4.59976800	0.85258300
H	7.32380500	4.53313400	1.60647400
H	7.92623500	5.29088900	3.89678600
I	2.18680300	5.34835800	2.16201300
F	2.01530200	7.13384800	3.28016700
H	1.23002900	6.65628400	4.61301600
F	0.76692400	-0.49782800	4.69264500
H	0.05479700	0.08834600	4.14495000

TS10

C	0.57274300	-0.69054300	0.68002900
O	0.84144000	-0.99609400	1.98046600
O	1.16763000	-1.25967700	-0.20277300
C	-0.50688900	0.33647600	0.48019800
C	-1.67060400	0.20092100	1.25860900
C	-0.37642000	1.42573300	-0.41003200
C	-2.70698200	1.12391500	1.16390900
H	-1.76037100	-0.64316300	1.93574300
C	-1.43564000	2.34146400	-0.49039700
C	-2.58362900	2.20292000	0.28400700
H	-3.60473300	0.99795400	1.76165900
H	-1.33283400	3.18895900	-1.15773300
H	-3.38302600	2.93296300	0.19798600
C	0.82550500	1.63310800	-1.27571200
C	1.02370900	0.69309300	-2.29895000
C	1.73665700	2.71334900	-1.16355000
C	2.08457000	0.79576900	-3.19237600
H	0.33384000	-0.13780400	-2.37665600
C	2.81436900	2.78637400	-2.06241500
C	2.99119200	1.84842300	-3.07076300
H	2.20403500	0.05032400	-3.97311400
H	3.50667000	3.61082300	-1.94000400
H	3.82960300	1.93721700	-3.75545500
C	1.70434700	3.83024800	-0.15151800
O	0.59873600	3.95898000	0.57725500
O	2.64782900	4.60177900	-0.03275200
C	4.50458700	1.31656900	2.66192400
C	3.10815400	1.34083600	2.68100100
C	2.51377200	2.58854700	2.80969200
C	3.19136600	3.79481400	2.88733100
C	4.58640100	3.73080400	2.85201300
C	5.23772600	2.50206100	2.74629500
H	5.01022600	0.36078300	2.56679400
H	2.53982800	0.42337800	2.59904300
H	2.65406300	4.73368200	2.92584300
H	5.15457200	4.65428900	2.89717200
H	6.32238400	2.46704700	2.71703300

I	0.35209400	2.67588800	2.78089000
F	0.28771700	0.95297600	3.82706400
H	0.41506200	-0.37008600	2.60352000
F	0.65290400	5.22560500	2.49445100
H	0.69015900	4.88173600	1.45402800

TS10-1

C	-0.39953900	1.17735300	2.23714800
O	-0.65906700	1.17937900	3.55804100
O	-0.01371600	0.18587700	1.64572000
C	-0.59636000	2.51506300	1.60911200
C	-0.87353300	3.62540300	2.41689200
C	-0.36279300	2.69435500	0.23090600
C	-0.93681800	4.90767800	1.87363600
H	-0.99444700	3.48141100	3.48270000
C	-0.47588400	3.98075800	-0.30987700
C	-0.75226700	5.08236300	0.49943100
H	-1.12240000	5.76074600	2.51755300
H	-0.30190600	4.11655500	-1.37319700
H	-0.80684900	6.07558400	0.06289300
C	0.07792800	1.60014600	-0.68605100
C	-0.86849400	0.79712100	-1.32789800
C	1.43830500	1.44248400	-1.01742000
C	-0.47586500	-0.16892100	-2.25224900
H	-1.91891700	0.92745500	-1.08647500
C	1.82442200	0.48585800	-1.96338900
C	0.87456000	-0.32884600	-2.57135600
H	-1.22600600	-0.79430200	-2.72793300
H	2.87700300	0.39899500	-2.21025900
H	1.18206500	-1.07721100	-3.29570600
C	2.49246800	2.28658500	-0.38156700
O	2.25512700	2.69476900	0.80453400
O	3.54599500	2.53744200	-1.03521200
C	3.58137900	-0.37253900	1.35644700
C	2.88482500	-0.17679100	2.55366000
C	3.36679100	0.80775600	3.39450700
C	4.46463000	1.60973600	3.13615700
C	5.13612400	1.38998400	1.93086500
C	4.70072100	0.39957000	1.04996800
H	3.21770000	-1.12107800	0.65971700
H	1.99614300	-0.74278800	2.79224100
H	4.79112100	2.37609500	3.82457900
H	5.97761800	2.02300800	1.67611400
H	5.21889200	0.25250800	0.10807800
I	2.24582000	1.16647100	5.24706100
C	1.60969400	5.32385400	5.96083100
O	0.77008200	5.25357400	4.91402900
O	2.54467400	6.10182800	6.01133500
C	1.28918000	4.32431500	7.01891300
C	0.04352100	3.68459000	7.02193300
C	2.27420900	3.94967000	7.95477500
C	-0.24262400	2.67975900	7.94400300
H	-0.69138300	3.97096700	6.27920700
C	1.97402600	2.93455800	8.87149600
C	0.72962400	2.30117900	8.87042500
H	-1.21221500	2.19140400	7.93178700
H	2.73659400	2.63173600	9.58271800
H	0.52516600	1.51302800	9.58923200

C	3.66068800	4.51397700	8.00406900
C	3.93376300	5.61544700	8.82107500
C	4.74030400	3.85036600	7.38759500
C	5.24045900	6.06072600	9.01021600
H	3.10759500	6.12621600	9.30522800
C	6.05558700	4.27377900	7.61459000
C	6.30838000	5.38525700	8.41314300
H	5.42700300	6.92811000	9.63675100
H	6.86569000	3.71909700	7.15189500
H	7.32896900	5.71837200	8.57665000
C	4.52277100	2.66341400	6.50654000
O	3.46387400	2.85002500	5.69447900
O	5.22565600	1.67249800	6.50355800
C	5.82612900	6.10020000	4.51136800
C	4.71969000	5.44213000	3.96692500
C	4.47504600	5.60771000	2.60951600
C	5.28802900	6.36021300	1.76573600
C	6.38974000	6.99913200	2.33040700
C	6.65424800	6.87207600	3.69807800
H	6.02037300	6.00447400	5.57390700
H	4.07556500	4.84685500	4.59890400
H	5.08253900	6.42320400	0.70559200
H	7.04128400	7.59533600	1.69863400
H	7.51340300	7.37805600	4.12863600
I	2.64276200	4.78611900	1.85055700
F	1.76156100	6.40986700	2.79321900
H	1.11795900	5.85229500	4.20027000
F	1.01424000	-0.44311100	4.69480000
H	-0.24888300	0.35822000	3.93719400
F	4.71761100	3.94328100	0.47518200
H	4.28817500	3.29968400	-0.24699300

TS10-2

C	-0.45435700	1.44134700	2.39649400
O	-0.30317600	1.65587800	3.71414900
O	-0.02416100	0.44232500	1.84294100
C	-1.16594700	2.54482500	1.70108100
C	-1.93402000	3.45198100	2.43677700
C	-1.02495300	2.70616200	0.30642400
C	-2.58283200	4.51345700	1.80920000
H	-2.01862200	3.31622600	3.50851400
C	-1.68564900	3.77492800	-0.30810200
C	-2.45592300	4.67455900	0.43115500
H	-3.17654900	5.20931800	2.39398400
H	-1.57661900	3.90834600	-1.38021500
H	-2.95109000	5.49974200	-0.07266200
C	-0.24476500	1.78765700	-0.58125800
C	-0.98223500	1.01582000	-1.48653700
C	1.16631000	1.70385500	-0.62915000
C	-0.35973800	0.16357600	-2.39741900
H	-2.06549400	1.07907300	-1.45752100
C	1.78420300	0.84928800	-1.55319900
C	1.03121300	0.07690400	-2.43221400
H	-0.96355700	-0.43048500	-3.07758500
H	2.86614200	0.80095200	-1.56842400
H	1.52614900	-0.58187100	-3.13936500
C	2.02113400	2.47963400	0.30340600

O	1.58164800	3.17558300	1.21480600
O	3.33178900	2.34629600	0.08208000
C	3.40076200	-1.00882600	1.18175100
C	3.03785800	-0.07697300	2.15756900
C	3.96925100	0.21238600	3.14415000
C	5.22872300	-0.36693400	3.21787800
C	5.56530400	-1.29033200	2.22639500
C	4.65921600	-1.60740100	1.21131700
H	2.68379200	-1.24593800	0.40212900
H	2.04269600	0.34683700	2.14208800
H	5.93088700	-0.08132300	3.98588800
H	6.54436900	-1.75928500	2.25463600
H	4.93711400	-2.32427500	0.44425700
I	3.22054700	1.52495300	4.72321400
C	1.51909400	5.72775800	5.76575300
O	0.62096900	5.83316300	4.76580700
O	2.37821500	6.56716400	5.96131600
C	1.37309700	4.47008900	6.54997600
C	0.39256600	3.53664100	6.19374300
C	2.31180500	4.15232300	7.55218800
C	0.32529500	2.29589200	6.82518100
H	-0.28587700	3.77105900	5.38452100
C	2.21566200	2.91421500	8.19728200
C	1.23759300	1.98681600	7.83662900
H	-0.42600600	1.57536100	6.52041500
H	2.94057400	2.66977800	8.96803600
H	1.19627000	1.02186700	8.33370500
C	3.45201500	5.03868100	7.93208000
C	3.28253200	5.99424900	8.93781400
C	4.73706600	4.84438100	7.39017600
C	4.35459700	6.77030600	9.37338600
H	2.29590900	6.13327400	9.36899300
C	5.81511800	5.61041400	7.84929700
C	5.62601500	6.58084400	8.82730500
H	4.19795300	7.51957000	10.14434300
H	6.79356000	5.43208500	7.41967000
H	6.46474500	7.18039400	9.16870500
C	4.99576400	3.82974000	6.32720200
O	4.03576500	3.60371200	5.50434900
O	6.12682100	3.27663700	6.28014900
C	5.83151600	7.83319100	4.74064000
C	4.71862700	7.30218300	4.08369800
C	4.84596700	6.02779100	3.55785600
C	5.98356900	5.24369300	3.66040200
C	7.07974200	5.80413300	4.32259100
C	7.00816200	7.09238400	4.85352000
H	5.76256400	8.82541800	5.17588300
H	3.78497400	7.84259200	4.01636900
H	6.00390200	4.22234300	3.30559600
H	7.98051800	5.20955100	4.43854100
H	7.86501100	7.51318900	5.37144200
I	3.09991000	5.17931600	2.57256500
F	2.11513400	6.96020800	2.94108000
H	0.97706100	6.52617100	4.15504200
F	1.65527500	0.17509300	4.75277000
H	0.29301700	0.95203900	4.07559500
F	4.28007700	3.51344200	2.22602700

H	3.80395400	2.78801000	0.84042000
F	5.94403000	2.03664600	4.24702100
H	6.12779000	2.54198500	5.12926800

H_2O

O	-3.62833000	-2.75743700	0.09582700
H	-2.66094200	-2.70811600	0.09582700
H	-3.90475900	-1.82907300	0.09582700

Iodosylbenzene

O	-2.70464800	0.23318800	3.03685400
C	-4.69119600	-3.08343900	0.96276000
C	-3.86752300	-2.22281300	1.69297200
C	-2.73169100	-2.76034800	2.26943800
C	-2.36288400	-4.09305100	2.16767900
C	-3.20121800	-4.93767800	1.43253900
C	-4.35858000	-4.43436100	0.83389900
H	-5.59229600	-2.69679000	0.49523400
H	-4.07577200	-1.16127500	1.82757000
H	-1.46102400	-4.47659300	2.63616800
H	-2.94492500	-5.98839400	1.33091500
H	-5.00257000	-5.09856000	0.26525800
I	-1.54920400	-1.29172000	3.37569200

Iodylbenzene

O	-2.25201400	0.24074900	2.86141500
C	-4.68781200	-3.08621500	0.96213500
C	-4.02459500	-2.20545900	1.82044200
C	-2.91206500	-2.68376500	2.49471300
C	-2.40045400	-3.96273000	2.34092700
C	-3.07660600	-4.82952100	1.47852400
C	-4.21694700	-4.39184800	0.79964700
H	-5.56202300	-2.74858200	0.41336300
H	-4.34065400	-1.17310600	1.93935000
H	-1.48937200	-4.25831700	2.85309700
H	-2.70562500	-5.83915100	1.32882500
H	-4.73677800	-5.07064600	0.13007400
I	-1.84371800	-1.31002200	3.79683900
O	-0.15168800	-2.02568200	3.5293950

17 Supplemental references

[1] M. J. Frisch, G. W. Trucks, H. B. Schlegel, G. E. Scuseria, M. A. Robb, J. R. Cheeseman, G. Scalmani, V. Barone, G. A. Petersson, H. Nakatsuji, X. Li, M. Caricato, A. V. Marenich, J. Bloino, B. G. Janesko, R. Gomperts, B. Mennucci, H. P. Hratchian, J. V. Ortiz, A. F. Izmaylov, J. L. Sonnenberg, D. W. Young, F. Ding, F. Lipparini, F. Egidi, J. Goings, B. Peng, A. Petrone, T. Henderson, D. Ranasinghe, V. G. Zakrzewski, J. Gao, N. Rega, G. Zheng, W. Liang, M. Hada, M. Ehara, K. Toyota, R. Fukuda, J. Hasegawa, M. Ishida, T. Nakajima, Y. Honda, O. Kitao, H. Nakai, T. Vreven, K. Throssell, J. Montgomery, J. E. Peralta, F. Ogliaro, M. J. Bearpark, J. J. Heyd, E. N. Brothers, K. N. Kudin, V. N. Staroverov, T. A. Keith, R. Kobayashi, J. Normand, K. Raghavachari, A. P. Rendell, J. C. Burant, S. S. Iyengar, J. Tomasi, M. Cossi, J. M. Millam, M. Klene, C. Adamo, R. Cammi, J. W. Ochterski, R. L. Martin, K. Morokuma, O. Farkas, J. B. Foresman, D. J. Fox, *Gaussian 16, Revision A.03*, Gaussian, Inc, CT, Wallingford **2016**.

[2] S. Grimme, J. Antony, S. Ehrlich, H. A. Krieg, *J. Chem. Phys.* **2010**, *132*, 154104.

[3] S. Grimme, S. Ehrlich, L. Goerigk, *J. Comput. Chem.* **2011**, *32*, 1456.

[4] C. Lee, W. Yang, R. G. Parr, *Phys. Rev. B: Condens. Matter*. **1988**, *37*, 785.

[5] Y. Zhao, D. G. Truhlar, *Theor. Chem. Acc.* **2008**, *120*, 215.

[6] A. V. Marenich, C. J. Cramer, D. G. Truhlar, *J. Phys. Chem. B.* **2009**, *113*, 6378.

[7] A. V. Marenich, C. J. Cramer, D. G. Truhlar, *J. Phys. Chem. B*. **2009,** *113*, 4538.

[8] K. Fukui, *Acc. Chem. Res.* **1981**, *14*, 363.

[9] Hratchian, H. P.; Schlegel, H. B. Accurate reaction paths using a Hessian based predictor–corrector integrator. *J. Chem. Phys.* **2004**,*120*, 9918–9924.

[10] H. P. Hratchian, H. B. Schlegel, *J. Chem. Theory. Comput.* **2005**, *1*, 61.

[11] C. Y. Legault, Lview; Université de Sherbrooke, Sherbrooke, Canada, **2009**; http://www.cylview.org.

[12]. Zhu, S.; Shi K.; Zhu, H.; Jia, Z. K.; Xia, X. F.; Wang, D.; Zou, L. Copper-catalyzed annulation or homocoupling of sulfoxonium ylides: synthesis of 2,3-diaroylquinolines or *α, α, β*-tricarbonyl sulfoxonium ylides. *Org. Lett.* **2007**, *22*, 1504.

[13]. Toma, T.; Shimokawa, J.; Fukuyama, T. *N,N*'-Ditosylhydrazine: a Convenient reagent for facile synthesis of diazoacetates *Org. Lett.* **2020**, *9*, 319.

[14]. L. Jiang, Z. Wang, M. Armstrong, M. G. Suero, *Angew. Chem., Int. Ed.* **2021**, *60*, 6177.

[15] M. T. Taylor, J. E. Nelson, M. G. Suero, M. J. Gaunt, *Nature* **2018,** *562*, 563.

[16] Z. Wang, A. G. Herraiz, A. M. Hoyo, M. G. Suero, *Nature* **2018**, *554*, 86–91.

[17] F. P. Wu, C. C. Chintawar, R. Lalisse, P. Mukherjee, S. Dutta, J. Tyler, C. G. Daniliuc, O. Gutierrez, F. Glorius, *Nat. Catal.* **2024**, *7*, 242..

[18] K. Matcha, R. Narayan, A. P. Antonchick, *Angew. Chem., Int. Ed.* **2013**, 52, 7985.

[19] M. Uyanik, K. Yasui, K. Ishihara, *Angew. Chem. Int. Ed.* **2010**, *49*, 2175.

[20] S. Gan, J. Yin, Z. Yu, L. Song, L. Shi, *Green Chem*. **2022**, *24*, 2232.

[21] M. Bielawski, B. Olofsson, *Chem. Commun*. **2007**, 24, 2521.

[22] M. Bielawski, M. Zhu, B. Olofsson, *Adv. Synth. Catal*. **2007**, 349, 2610.

[23] A. J. To, G. K. Murphy, *New J. chem*. **2022**, *46*, 15313.

[24] J. Borrel, J. Waser, *Org. Lett*. **2022**, *24*, 142.

[25] M. J. Bouma, B. Olofsson, *Chem. Eur. J.* **2012**, *18*, 14242.

[26] E. Stridfeldt, A. Seemann, M. J. Bouma, C. Dey, A. Ertan, B. Olofssonm, *Chem. Eur. J.* **2016***, 22*, 16066.

[27] T. Kitamura, S. Mizuno, K. Muta, J. Oyamada, *J. Org. Chem*. **2018**, *83*, 2773.

[28] L. Li, K. Deng, Y. Xing, C. Ma, S. F. Ni, Z. Wang, Y. Huang, *Nat. Commun*. **2022**, 13, 6588.
[29] H. Chen, J. Han, L. Wang, *Angew. Chem., Int. Ed.* **2018**, 57, 12313.
[30] P. Finkelstein, J. C. Reisenbuaer, B. B. Botlik, O. Green, A. Florin, B. Morandi, *Chem. Sci*. **2023**, *14*, 2954.
[31] M. A. Ansari, G. Kumar, M. S. Singh, *Org. Lett*. **2022**, *24*, 2815..
[32] L. Liu, Y. Zhang, W. Zhao, J. Li, *Org. Lett*. **2023**, *25*, 6251.
[33] Q. He, Q. Zhang, A. B. Rolka, M. G. Suero, *J. Am. Chem. Soc*. **2024**, 146, 12294.
[34] J. C. Sarie, J. Neufeld, C. G. Daniliuc, R. Gilmour, *ACS Catal*. **2019,** 9, 7232.
[35] T. Patra, S. Arepally, J. Seitz, T. Wirth, *Nat. Commun*. **2024**, *15*, 6329.
[36] S. Chong, Y. Su, L. Wu, W. Zhang, J. Ma, X. Chen, D. Huang, K. H. Wang, Y. Hu, *Synthesis* **2016**, *48*, 1359.
[37] F. Scheidt, M. Schäfer, J. C. Sarie, C. G. Daniliuc, J. J. Molloy, R. Gilmour, *Angew. Chem., Int. Ed*. **2018**, *57*, 16431.
[38] C. G. Sarie, C. Thiehoff, J. Neufeld, C. G. Daniliuc, R. Gilmour, *Angew. Chem. Int. Ed*. **2020**, *59*, 15069.
[39] A. C. Dean, E. H. Randle, A. J. D. Lacey, G. A. Marczak Giorio, S. Doobary, B. D. Cons, A. J. J. Lennox, *Angew. Chem., Int. Ed*. **2024**, *63*, e202404666.
[40] W. W. Zhu, X. Zhen, J. Y. Wu, Y. P. Cheng, J. K. An, X. Y. Ma, J. K. Liu, Y. J. Qin, H. Zhu, J. J. Xue, X. X. Jiang, *Nat. Commun*. 2021, **2021**, *12*, 3957.
[41] E. M. Woerly, S. M. Banik, E. N. Jacobsen, *J. Am. Chem. Soc*. **2016**, *138*, 13858.

18. Characterization of compounds

Diphenyliodonium triflate (3a)

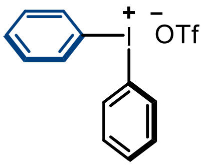

Compound **3a** was prepared according to the **general procedure A** from iodobenzene (0.3 mmol, 61.2 mg) and benzene (0.6 mmol, 46.8 mg). Obtained as a white solid (108.4 mg, 84%). **^{1}H NMR** (400 MHz, DMSO-d_6) δ 8.25 (d, J = 8.2 Hz, 1H), 7.67 (t, J = 7.4 Hz, 1H), 7.53 (t, J = 7.8 Hz, 2H).**^{13}C NMR** (101 MHz, DMSO-d_6) δ 135.69, 132.59, 132.29, 117.03.

Phenyl(p-tolyl)iodonium triflate (3b)

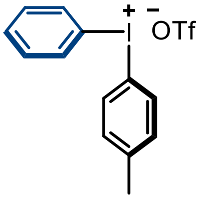

Compound **3b** was prepared according to the **general procedure A** from iodobenzene (0.3 mmol 61.2 mg) and toluene (0.6 mmol, 55.3 mg). Obtained as a white solid (122.6 mg, 92%). **^{1}H NMR** (400 MHz, DMSO-d_6) δ 8.24 (d, J = 9.5 Hz, 2H), 8.15 (d, J = 8.4 Hz, 2H), 7.67 (t, J = 7.5 Hz, 1H), 7.54 (t, J = 7.8 Hz, 2H), 7.36 (d, J = 8.2 Hz, 2H), 2.36 (s, 3H). **^{13}C NMR** (101 MHz, DMSO-d_6) δ 142.58, 135.17, 135.01, 132.37, 131.95, 131.71, 116.63, 112.86, 20.84.

(4-Methoxyphenyl)(phenyl)iodonium triflate (3c)

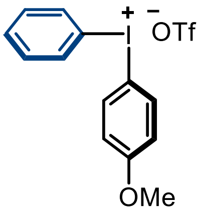

Compound **3c** was prepared according to the **general procedure A** from iodobenzene (0.3 mmol 61.2 mg) and anisole (0.6 mmol, 65.1 mg). Obtained as a white solid (113.1 mg, 82%). **^{1}H NMR** (400 MHz, Methanol-d_4) δ 8.99 (td, J = 9.1, 8.5, 1.7 Hz, 4H), 8.50 – 8.42 (m, 1H), 8.33 (t, J = 7.8 Hz, 2H), 7.93 – 7.84 (m, 2H), 4.60 (s, 3H). **^{13}C NMR** (101 MHz, Methanol-d_4) δ 163.31, 138.56, 136.12, 133.19, 132.98, 118.79, 106.69, 57.02.

(4-Bromophenyl)(phenyl)iodonium triflate (3d)

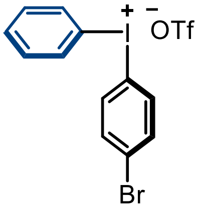

Compound **3d** was prepared according to the **general procedure A** from iodobenzene (0.3 mmol 61.2 mg) and bromobenzene (0.6 mmol, 94.2 mg). Obtained as a white solid (114.5 mg, 75%). **^{1}H NMR** (400 MHz, Methanol-d_4) δ 8.25 – 8.17 (m, 2H), 8.10 (d, J = 8.7 Hz, 1H), 7.92 (s, 2H), 7.73 (d, J = 7.9 Hz, 2H), 7.58 (t, J = 7.7 Hz, 3H).**^{13}C NMR** (101 MHz, Methanol-d_4) δ 141.02, 136.72, 136.40, 135.13, 134.95, 132.50, 131.94.

(4-Iodophenyl)(phenyl)iodonium triflate (3e)

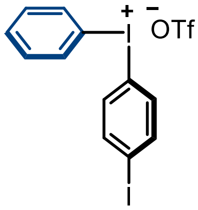

Compound **3e** was prepared according to the **general procedure A** from iodobenzene (0.9 mmol, 183.6 mg). Obtained as a white solid (136.7 mg, 82%). **^{1}H NMR** (400 MHz, DMSO-d_6) δ 8.27 – 8.20 (m, 2H), 8.00 (d, J = 8.5 Hz, 2H), 7.90 (d, J = 8.6 Hz, 2H), 7.71 – 7.62 (m, 1H), 7.54 (dd, J = 9.1, 6.5 Hz, 2H). **^{13}C NMR** (101 MHz, DMSO-d_6) δ 140.93, 137.30, 135.70, 132.68, 132.34, 117.20, 116.37.

(2-Bromo-4,6-dimethylphenyl)(phenyl)iodonium triflate and (4-bromo-2,6-dimethylphenyl)(phenyl)iodonium triflate (3f + 3f')

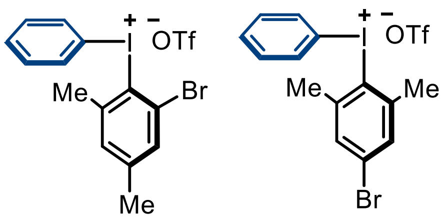

Compound **3f** and **3f'** was prepared according to the **general procedure A** from iodobenzene (0.3 mmol 61.2 mg) and 5-bromo-m-xylene (0.6 mmol, 111.1 mg). Obtained as a white solid (141.8 mg, 88%). **^{1}H NMR** (400 MHz, Methanol-d_4) δ 8.15 – 8.09 (m, 1H), 8.00 (dd, J = 8.5, 1.2 Hz, 1H), 7.76 – 7.67 (m, 1H), 7.65 (s, 2H), 7.57 (td, J = 7.8, 4.2 Hz, 2H), 7.41 (d, J = 2.0 Hz, 1H), 2.82 (s, 2H), 2.73 (s, 3H), 2.40 (s, 2H). **^{13}C NMR** (101 MHz, Methanol-d_4) δ 144.35, 134.58, 134.27, 132.38, 132.23, 132.10, 132.00, 131.93, 131.86, 131.10, 48.31, 48.09, 47.88, 47.67, 47.45, 47.24, 47.03, 26.74, 25.56, 19.60.

Mesityl(phenyl)iodonium triflate (3g)

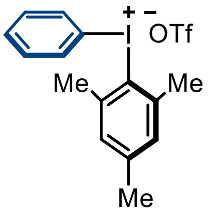

Compound **3g** was prepared according to the **general procedure A** from iodobenzene (0.3 mmol 61.2 mg) and mesitylene (0.6 mmol, 72.1 mg). Obtained as a white solid (113.5 mg, 80%). **^{1}H NMR** (400 MHz, DMSO-d_6) δ 8.00 (d, J = 8.3 Hz, 2H), 7.65 (d, J = 8.7 Hz, 1H), 7.52 (t, J = 7.8 Hz, 2H), 7.24 (s, 2H), 2.62 (s, 6H), 2.32 (s, 3H). **^{13}C NMR** (101 MHz, DMSO-d_6) δ 143.65, 142.10, 135.00, 132.41, 132.32, 130.31, 123.07, 115.02, 26.81, 21.02.

Phenyl(thiophen-2-yl)iodonium triflate (3h)

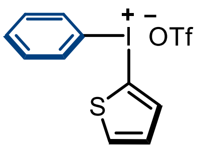

Compound **3h** was prepared according to the **general procedure A** from iodobenzene (0.3 mmol 61.2 mg) and thiophene (0.6 mmol, 50.5 mg). Obtained as a white solid (98.1 mg, 75%). **^{1}H NMR** (400 MHz, Methanol-d_4) δ 8.23 (d, J = 4.6 Hz, 2H), 8.07 (d, J = 7.3 Hz, 1H), 7.94 (d, J = 5.7 Hz, 1H), 7.73 (d, J = 7.4 Hz, 1H), 7.58 (d, J = 7.6 Hz, 2H), 7.24 (d, J = 5.9 Hz, 1H). **^{13}C NMR** (101 MHz, Methanol-d_4) δ 140.86, 137.29, 134.42, 132.41, 131.83, 129.54.

***o*-Tolyl(*p*-tolyl)iodonium triflate (3i)**

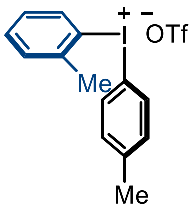

Compound **3i** was prepared according to the **general procedure A** from 2-iodotoluene (0.3 mmol, 65.4 mg) and toluene (0.6 mmol, 55.3 mg). Obtained as a white solid (115.5 mg, 84%). **^{1}H NMR** (400 MHz, Methanol-d_4) δ 8.30 (d, J = 8.2 Hz, 1H), 8.00 (d, J = 8.5 Hz, 2H), 7.68 – 7.51 (m, 2H), 7.36 (dd, J = 8.0, 6.0 Hz, 3H), 2.69 (s, 3H), 2.42 (s, 3H). **^{13}C NMR** (101 MHz, Methanol-d_4) δ 143.63, 141.06, 137.14, 134.80, 133.18, 132.60, 131.58, 129.35, 119.66, 110.10, 24.27, 19.96.

***m*-Tolyl(*p*-tolyl)iodonium triflate (3j)**

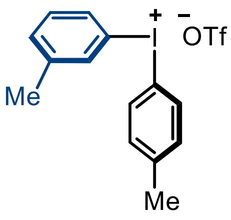

Compound **3j** was prepared according to the *general procedure A* from 3-iodotoluene (0.3 mmol, 65.4 mg) and toluene (0.6 mmol, 55.3 mg). Obtained as a white solid (115.5 mg, 84%). **^{1}H NMR** (400 MHz, DMSO-d_6) δ 8.16 – 8.08 (m, 3H), 8.03 (d, J = 7.8 Hz, 1H), 7.49 (d, J = 7.7 Hz, 1H), 7.42 (t, J = 7.8 Hz, 1H), 7.35 (d, J = 8.0 Hz, 2H), 2.35 (s, 6H). **^{13}C NMR** (101 MHz, DMSO-d_6) δ 143.05, 142.25, 135.70, 135.64, 133.14, 132.87, 132.60, 131.90, 116.94, 113.27, 21.34, 21.24.

***p*-Tolyl(*p*-tolyl)iodonium triflate (3k)**

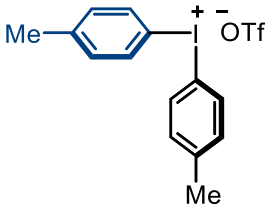

Compound **3k** was prepared according to the **general procedure A** from 4-iodotoluene (0.3 mmol, 65.4 mg) and toluene (0.6 mmol, 55.3 mg). Obtained as a white solid (116.7 mg, 85%). **^{1}H NMR** (400 MHz, Methanol-*d*$_4$) δ 8.04 (d, *J* = 8.4 Hz, 4H), 7.37 (d, *J* = 8.2 Hz, 4H), 2.42 (s, 6H). **^{13}C NMR** (101 MHz, Methanol-*d*$_4$) δ 143.66, 134.94, 132.49, 111.03, 19.97.

(4-Nitrophenyl)(phenyl)iodonium triflate (3l)

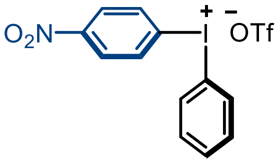

Compound **3l** was prepared according to the **general procedure A** from 4-iodonitrobenzene (0.3 mmol, 74.7 mg) and benzene (0.6 mmol, 46.9 mg). Obtained as a yellow solid (79.8 mg ,56%). **^{1}H NMR** (400 MHz, Methanol-*d*$_4$) δ 8.45 (d, *J* = 7.2 Hz, 2H), 8.34 (d, *J* = 7.0 Hz,2H), 8.28 (d, *J* = 8.2 Hz, 2H), 7.76 (t, *J* = 7.4 Hz, 1H), 7.68 – 7.55 (m, 2H). **^{13}C NMR** (101 MHz, Methanol-*d*$_4$) δ 136.32, 135.51, 132.77, 132.11, 126.16.

Bis(4-chlorophenyl)iodonium triflate (3m)

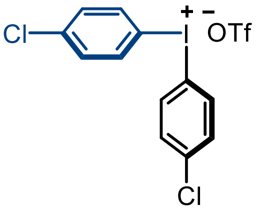

Compound **3l** was prepared according to the **general procedure A** from 4-chlorophenyl iodide (0.3 mmol, 71.5 mg) and chlorobenzene (0.6 mmol, 67.5 mg). Obtained as a white solid in 55% yield. **^{1}H NMR** (400 MHz, DMSO-*d*$_6$) δ 8.29 (d, *J* = 8.8 Hz, 4H), 7.66 (d, *J* = 8.7 Hz, 4H). **^{19}F NMR** (376 MHz, DMSO-*d*$_6$) δ -77.71. **^{13}C NMR** (101 MHz, DMSO-*d*$_6$) δ 138.01, 137.51, 132.34, 115.23.

Dibenzo[b,d]iodol-5-ium trifluoromethanesulfonate (3n)

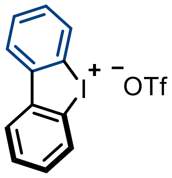

Prepared according to the **general procedure B**, **3n** was obtained as a white solid (107.9 mg, 84% yield**)**. **^{1}H NMR** (400 MHz, Methanol-*d*$_4$) δ 8.35 (d, *J* = 7.7 Hz, 2H), 8.13 (d, *J* = 8.3 Hz, 2H), 7.86 (t, *J* = 7.6 Hz, 2H), 7.70 (t, *J* = 7.8 Hz, 2H). **^{13}C NMR** (101 MHz, Methanol-*d*$_4$) δ 142.30, 131.12, 131.01, 130.25, 127.01, 120.07.

Dibenzo[b,d]iodol-5-ium tetrafluoroborate (3n')

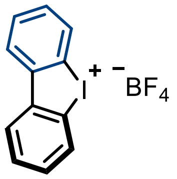

Prepared according to the general procedure B, **3n'** was obtained as a white solid (98.5 mg, 89% yield**)**. **^{1}H NMR** (400 MHz, Methanol-*d*$_4$) δ 8.30 (d, *J* = 7.9 Hz, 2H), 8.09 (d, *J* = 8.2 Hz, 2H), 7.84 (t, *J* = 6.0 Hz, 2H), 7.69 (t, *J* = 6.0 Hz, 2H). **^{19}F NMR** (376 MHz, Methanol-*d*$_4$) δ -153.40. **^{13}C NMR** (101 MHz, Methanol-*d*$_4$) δ 142.22, 131.15, 131.02, 130.24, 127.00, 120.00.

(3,5-Dimethylisoxazol-4-yl)(4-methoxyphenyl)iodonium triflate (DMIX-I)

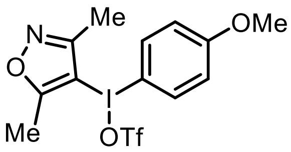

^{1}H NMR (400 MHz, DMSO-*d*$_6$) δ 8.19 (dd, *J* = 9.0, 3.2 Hz, 2H), 7.10 (dd, *J* = 9.1, 3.2 Hz, 2H), 3.83 (d, *J* = 3.2 Hz, 3H), 2.74 (d, *J* = 3.2 Hz, 3H), 2.38 (d, *J* = 3.2 Hz, 3H). **^{13}C NMR** (101 MHz, DMSO-*d*$_6$) δ 176.08, 162.58, 160.89, 137.55, 120.8 (q, *J* = 322.2 Hz 118.09, 106.31, 86.63, 12.77, 11.40. **^{19}F NMR** (376 MHz, DMSO-*d*$_6$) δ -77.73.

1-(*p*Methylbenzenesulfonyloxy)-1,2-benziodoxol-3-(1H)-one (3aa)

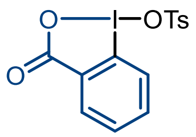

Prepared according to the general procedure, **3aa** was obtained as a white solid (1.2 g, 72% yield). **^{1}H NMR** (400 MHz, DMSO-d_6) δ 8.01 (dd, J = 7.5, 1.5 Hz, 1H), 7.98 – 7.93 (m, 1H), 7.83 (dd, J = 8.1, 0.9 Hz, 1H), 7.70 (td, J = 7.4, 1.0 Hz, 1H), 7.51 – 7.46 (m, 2H), 7.15 – 7.10 (m, 2H), 2.28 (s, 3H, CH_3). **^{13}C NMR** (101 MHz, DMSO-d_6) δ 168.39, 145.36, 138.72, 135.05, 131.97, 131.62, 130.91, 128.77, 126.84, 126.02, 120.99, 21.32.

1-Phenylethynyl-1H-1λ^3-benzo[d][1,2]iodoxol-3-one (3ab)

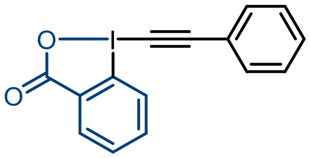

Prepared according to the general procedure, **3ac** was obtained as a white solid (1.0 g, 75% yield). **^{1}H NMR** (400 MHz, Chloroform-d) δ 8.45-8.41 (m, 1H), 8.28-8.23 (m, 1H), 7.81-7.75 (m, 2H), 7.62-7.59 (m, 2H), 7.51-7.41 (m, 3H). **^{13}C NMR** (101 MHz, Chloroform-d) δ 166.81, 134.99, 132.94, 132.52, 131.65, 131.46, 130.85, 128.85, 126.45, 120.64, 116.29, 106.62, 50.22.

(E)-1-Styryl-1,2-benziodoxol-3-(1H)-one (3ac)

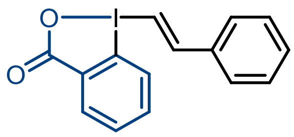

Prepared according to the general procedure, **3ab** was obtained as a white solid (1.1g, 82% yield). **^{1}H NMR** (400 MHz, Methanol-d_4) δ 8.31-8.22 (m, 1H), 7.96 (d, J = 15.5 Hz, 1H), 7.76-7.61 (m, 6H), 7.55-7.42 (m, 3H). **^{13}C NMR** (101 MHz, Methanol-d_4) δ 170.77, 156.43, 137.34, 135.98, 133.93, 132.82, 132.46, 131.72, 130.98, 130.89, 129.68, 116.17, 100.64.

(E)-2-Fluoro-1-hexen-1-yl(phenyl)iodonium tetrafluoroboratem (3ad)

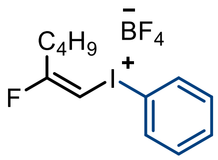

Prepared according to the general procedure, **3ad** was obtained as a brownish oil (121.5 mg, 62% yield). **^{1}H NMR** (400 MHz, Chloroform-d) δ 7.97 (d, J = 8.5 Hz, 2H), 7.60 (d, J = 7.4 Hz, 1H), 7.47 (t, J = 7.9 Hz, 2H), 6.71 (d, J = 14.5 Hz, 1H), 2.78 (dt, J = 22.2, 7.6 Hz, 2H), 1.48 (t, J = 7.2 Hz, 2H), 1.37 – 1.19 (m, 2H), 0.87 (t, J = 7.3 Hz, 3H). **^{13}C NMR** (101 MHz, Chloroform-d) 13.3, 21.6, 27.5, 31.5 (d, J= 24.0 Hz), 78.5 (d, J= 47.2 Hz), 112.1,132.1, 132.5, 134.5, 175.7 (d, J= 285.0 Hz). **^{19}F NMR** (376 MHz, Chloroform-d) δ -65.62 (td, J = 22.0, 14.6 Hz), -146.02 (d, J = 20.7 Hz).

(1-(Dimethyl(oxo)-λ^6-sulfanylidene)-2-oxo-2-phenylethyl)(phenyl)-λ^3-iodanyl trifluoromethanesulfon-ate (3ae)

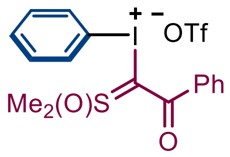

Prepared according to the general procedure, **3ae** was collected as a white solid (2.42 g, 88% yield). **^{1}H NMR** (400 MHz, CD_3CN) δ 7.82 – 7.77 (m, 2H), 7.75 – 7.70 (m, 1H), 7.64 – 7.55 (m, 3H), 7.51 (qt, J = 8.4, 1.3 Hz, 4H), 3.76 (s, 6H). **^{13}C NMR** (101 MHz, CD_3CN) δ188.87, 138.77, 132.96, 132.33, 132.02, 131.29, 128.51, 127.41, 120.90(q, J = 316.6 Hz), 117.42, 61.47, 42.44. **^{19}F NMR** (376 MHz, CD_3CN) δ -79.23.

(1-(Dimethyl(oxo)-λ^6-sulfaneylidene)-2-oxo-2-phenylethyl)(phenyl)iodonium tetrafluoroborate (3ae')

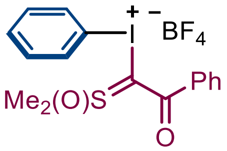

Prepared according to the general procedure, **3ae** was collected as a white solid (2.1 g, 85% yield).**^{1}H NMR** (400 MHz, CD_3CN) δ 7.83 – 7.78 (m, 1H), 7.77 – 7.70 (m, 1H), 7.64 – 7.56 (m, 2H), 7.55 – 7.46 (m, 2H), 3.76 (s, 3H). **^{13}C NMR** (101 MHz, CD_3CN) δ 188.93, 138.69, 133.01, 132.55, 132.20, 131.44, 128.62, 127.46, 117.45, 60.95, 42.55. **^{19}F NMR** (376 MHz, CD_3CN) δ -150.62 (d, *J* = 20.4 Hz).

2-(2-Iodophenoxy)-1,3,5-trimethylbenzene (5)

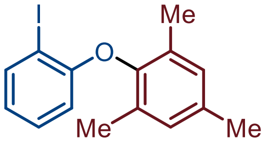

Prepared according to the general procedure, **7** was collected as a colorless oil (253.6 mg, 75% yield). **^{1}H NMR** (400 MHz, Chloroform-*d*) δ 7.83 (dd, *J* = 7.7, 1.4 Hz, 1H), 7.12 (ddd, *J* = 8.3, 7.4, 1.6 Hz,1H), 6.91 (s, 2H), 6.72 (td, *J* = 7.6, 1.4 Hz, 1H), 6.31 (dd, *J* = 8.2, 1.4 Hz, 1H), 2.30 (s, 3H), 2.08 (s, 6H). **^{13}C NMR (**101 MHz, Chloroform-*d*) δ 156.49, 149.16, 139.72, 134.89, 130.82, 129.67, 129.45, 123.06, 112.95, 85.07, 20.87, 16.29.

Isoquinoline (6)

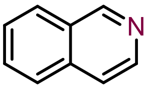

9

Prepared according to the general procedure, **6** was collected as a yellow oil (7.8 mg, 30% yield). **^{1}H NMR** (400 MHz, Chloroform-*d*) δ 9.20 (s, 1H), 8.48 (d, *J* = 5.8 Hz, 1H), 7.88 (d, *J* = 8.2 Hz, 1H), 7.73 (d, *J* = 8.2 Hz, 1H), 7.61 (t, *J* = 8.2 Hz, 1H), 7.56 (d, *J* = 5.8 Hz, 1H), 7.52 (t, *J* = 7.5 Hz, 1H). **^{13}C NMR** (101 MHz, Chloroform-*d*) δ 152.57, 143.04, 135.77, 130.36, 128.69, 127.62, 127.26, 126.48, 120.49.

(1-Diazo-2-ethoxy-2-oxoethyl)(phenyl)iodonium trifluoromethanesulfonate (8a)

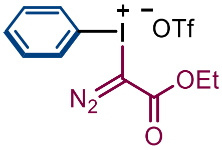

Prepared according to the **general procedure C**, **8a** was collected as a yellow solid (121.6 mg, 87% yield). **^{1}H NMR** (400 MHz, Chloroform-*d*) δ 8.11 (d, *J* = 8.0 Hz, 2H), 7.67 (t, *J* = 7.5 Hz, 1H), 7.51 (t, *J* = 7.8 Hz, 2H), 4.33 (q, *J* = 7.1 Hz, 2H), 1.32 (t, *J* = 7.1 Hz, 3H). **^{13}C NMR** (101 MHz, Chloroform-*d*) δ 161.54, 135.39, 133.00, 131.93, 119.92 (q, *J* = 320.2), 117.29, 64.17, 14.31. **^{19}F NMR** (376 MHz, Chloroform-*d*) δ -78.42.

(1-Diazo-2-ethoxy-2-oxoethyl)(phenyl)iodonium tetrafluoroborate (8a')

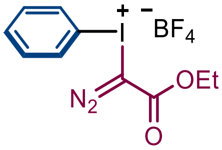

Prepared according to the **general procedure C**, **8a'** was collected as a yellow solid (96.9 mg, 80% yield). **^{1}H NMR** (400 MHz, Acetonitrile-*d*$_3$) δ 8.17 (d, *J* = 8.4 Hz, 2H), 7.80 (t, *J* = 7.5 Hz, 1H), 7.63 (t, *J* = 7.9 Hz, 2H), 4.32 (q, *J* = 7.1 Hz, 2H), 1.29 (t, *J* = 7.1 Hz, 3H). **^{13}C NMR** (101 MHz, Acetonitrile-*d*$_3$) δ 135.70, 133.68, 132.32, 117.63, 64.40, 13.57. **^{19}F NMR** (376 MHz, Acetonitrile-*d*$_3$) δ -149.73 (d, *J* = 20.3 Hz).

(1-Diazo-2-ethoxy-2-oxoethyl)(4-fluorophenyl)iodonium trifluoromethanesulfonate (8b)

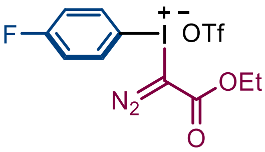

Prepared according to the **general procedure C**, **8b** was collected as a yellow solid (127.8 mg, 88% yield). ^{1}H NMR (400 MHz, Acetonitrile-*d*$_3$) δ 8.17 (d, *J* = 8.4 Hz, 2H), 7.63 (t, *J* = 7.9 Hz, 2H), 4.32 (q, *J* = 7.1 Hz, 2H), 1.29 (t, *J* = 7.1 Hz, 3H). **^{13}C NMR** (101 MHz, Chloroform-*d*) 166.5, 164.0, 161.73,

138.29 (d, J = 9.1 Hz), 119.4 (q, J = 320.2), 110.4, 64.0,14.2 **^{19}F NMR** (376 MHz, Chloroform-*d*) δ -78.54, -103.73 –103.85 (m).

(1-Diazo-2-ethoxy-2-oxoethyl)(4-fluorophenyl)iodonium tetrafluoroborate (8b')

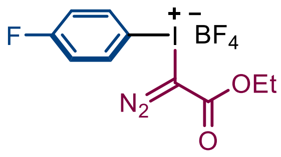

Prepared according to the **general procedure C**, **8b'** was collected as a yellow solid (86.1 mg, 68% yield). **^{1}H NMR** (400 MHz, Methanol-*d*$_4$) δ 8.30 (dd, J = 8.8, 4.8 Hz, 2H), 7.41 (t, J = 8.5 Hz, 2H), 4.36 (q, J = 7.1 Hz, 2H), 1.32 (t, J = 7.0 Hz, 3H). **^{13}C NMR** (101 MHz, Methanol-*d*$_4$) δ 166.67, 164.14, 161.74, 138.17 (d, J = 9.4 Hz), 119.21, 118.98, 111.06, 63.84, 13.22. **^{19}F NMR** (376 MHz, Methanol-*d*$_4$) δ -106.02, -153.39.

(4-Chlorophenyl)(1-diazo-2-ethoxy-2-oxoethyl)iodonium trifluoromethanesulfonate (8c)

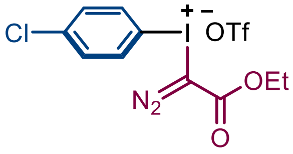

Prepared according to the **general procedure C**, **8c** was collected as a yellow solid (120.1 mg, 80% yield). **^{1}H NMR** (400 MHz, Chloroform-*d*) δ 8.08 (d, J = 8.7 Hz, 2H), 7.43 (d, J = 8.8 Hz, 2H), 4.32 (q, J = 7.1 Hz, 2H), 1.31 (t, J = 7.1 Hz, 3H). **^{13}C NMR** (101 MHz, Chloroform-*d*) δ 161.59, 139.93, 136.90, 131.93, 119.77 (q, J = 319.1 Hz), 114.20, 64.14, 14.27. **^{19}F NMR** (376 MHz, Chloroform-*d*) δ -78.48.

(4-Bromophenyl)(1-diazo-2-ethoxy-2-oxoethyl)iodonium trifluoromethanesulfonate (8d)

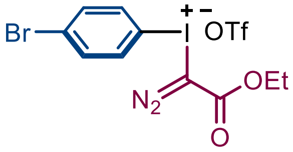

Prepared according to the **general procedure C**, **8d** was collected as a yellow solid (135.7 mg, 83% yield). **^{1}H NMR** (400 MHz, Chloroform-*d*) δ 7.94 (d, J = 8.7 Hz, 2H), 7.54 (d, J = 8.7 Hz, 2H), 4.28 (q, J = 7.1 Hz, 2H), 1.28 (t, J = 7.1 Hz, 3H). **^{13}C NMR** (101 MHz, Chloroform-*d*) δ 161.80, 136.75, 134.83, 128.1, 119.92 (q, J = 320.2), 115.90, 64.00, 14.29. **^{19}F NMR** (376 MHz, Chloroform-*d*) δ -78.42.

(4-(tert-butyl)phenyl)(1-diazo-2-ethoxy-2-oxoethyl)iodonium trifluoromethanesulfonate (8e)

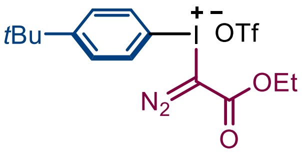

Prepared according to the **general procedure C**, **8e** was collected as a yellow solid (133.2 mg, 85% yield). **^{1}H NMR** (400 MHz, Chloroform-*d*) δ 7.97 (d, J = 8.4 Hz, 2H), 7.46 (d, J = 8.9 Hz, 2H), 4.30 (q, J = 7.1 Hz, 2H), 1.33 (s, 9H), 1.30 (t, J = 7.1 Hz, 3H). **^{13}C NMR** (101 MHz, Chloroform-*d*) δ 161.82, 156.80, 135.01, 129.14, 119.94 (q, J = 319.7 Hz), 114.02, 63.90, 35.30, 31.02, 14.25. **^{19}F NMR** (376 MHz, Chloroform-*d*) δ -78.45.

(1-diazo-2-ethoxy-2-oxoethyl)(p-tolyl)iodonium trifluoromethanesulfonate (8f)

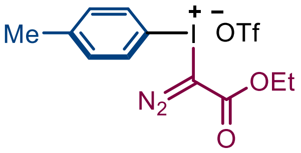

Prepared according to the **general procedure C**, **8f** was collected as a yellow solid (120.0 mg, 84% yield). **^{1}H NMR** (400 MHz, Chloroform-*d*) δ 7.98 (d, J = 8.2 Hz, 2H), 7.30 (d, J = 8.3 Hz, 2H), 4.33 (q, J = 7.1 Hz, 2H), 2.46 (s, 3H), 1.32 (t, J = 7.1 Hz, 3H). **^{13}C NMR** (101 MHz, Chloroform-*d*) δ 161.49, 144.24, 135.37, 132.72, 119.94 (q, J = 319.7 Hz), 113.36, 64.09, 21.60, 14.28. **^{19}F NMR** (376 MHz, Chloroform-*d*) δ -78.26.

(4-Cyanophenyl)(1-diazo-2-ethoxy-2-oxoethyl)iodonium trifluoromethanesulfonate (8g)

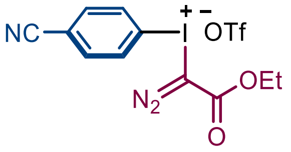

Prepared according to the **general procedure C**, **8g** was collected as a yellow solid (66.3 mg, 45% yield). **^{1}H NMR** (400 MHz, Chloroform-*d*) δ 8.33 (d, *J* = 8.3 Hz, 1H), 7.75 (d, *J* = 8.3 Hz, 1H), 4.34 (q, *J* = 7.1 Hz, 1H), 1.32 (t, *J* = 7.1 Hz, 2H). **^{13}C NMR** (101 MHz, Chloroform-*d*) δ 161.52, 136.12, 134.61, 121.24, 119.94 (q, *J* = 319.7 Hz), 116.94, 116.78, 64.37, 14.26. **^{19}F NMR** (376 MHz, Chloroform-*d*) δ -78.58.

(1-Diazo-2-ethoxy-2-oxoethyl)(4-(methoxycarbonyl)phenyl)iodonium tetrafluoroborate (8h)

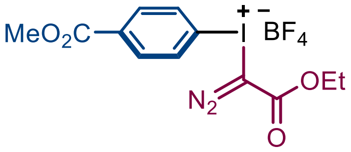

Prepared according to the **general procedure C**, **8h** was collected as a yellow solid (108.1 mg, 78% yield). **^{1}H NMR** (400 MHz, Methanol-*d*$_4$) δ 8.32 (d, *J* = 8.2 Hz, 1H), 8.16 (d, *J* = 8.1 Hz, 1H), 4.34 (q, *J* = 7.0 Hz, 1H), 3.99 (s, 1H), 1.32 (t, *J* = 7.1 Hz, 2H). **^{13}C NMR** (101 MHz, Methanol-*d*$_4$) δ 165.41, 161.95, 134.98, 133.94, 132.08, 122.51, 63.75, 51.98, 13.23. **^{19}F NMR** (376 MHz, Methanol-*d*$_4$) δ -153.48 (d, *J* = 19.3 Hz).

(1-Diazo-2-ethoxy-2-oxoethyl)(m-tolyl)iodonium trifluoromethanesulfonate (8i)

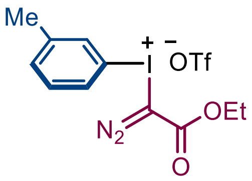

Prepared according to the **general procedure C**, **8i** was collected as a yellow solid (126.8 mg, 88% yield). **^{1}H NMR** (400 MHz, Chloroform-*d*) δ 7.92 – 7.81 (m, 2H), 7.46 (d, *J* = 7.5 Hz, 1H), 7.37 (t, *J* = 7.8 Hz, 1H), 4.34 (q, *J* = 7.1 Hz, 2H), 1.33 (t, *J* = 7.1 Hz, 3H). **^{13}C NMR** (101 MHz, Chloroform-*d*) δ 161.53, 142.72, 135.44, 133.79, 132.19, 131.55, 119.94 (q, *J* = 319.7 Hz), 117.33, 64.08, 21.46, 14.30. **^{19}F NMR** (376 MHz, Chloroform-*d*) δ -78.39.

(1-Diazo-2-ethoxy-2-oxoethyl)(o-tolyl)iodonium trifluoromethanesulfonate (8j)

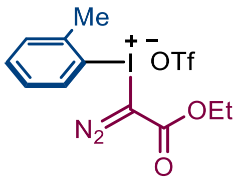

Prepared according to the **general procedure C**, **8j** was collected as a yellow solid (115.2 mg, 80% yield). **^{1}H NMR** (400 MHz, Chloroform-*d*) δ 8.16 (d, *J* = 8.1 Hz, 1H), 7.58 (t, *J* = 7.4 Hz, 1H), 7.50 (d, *J* = 7.6 Hz, 1H), 7.32 – 7.22 (m, 1H), 4.31 (q, *J* = 7.0 Hz, 2H), 2.74 (s, 3H), 1.31 (t, *J* = 7.2 Hz, 3H). **^{13}C NMR** (101 MHz, Chloroform-*d*) δ 161.33, 141.51, 137.62, 133.75, 131.84, 129.44, 122.51, 119.94 (q, *J* = 319.7 Hz), 64.10, 25.59, 14.25. **^{19}F NMR** (376 MHz, Chloroform-*d*) δ -78.39.

(1-Diazo-2-ethoxy-2-oxoethyl)(o-tolyl)iodonium tetrafluoroborate (8j')

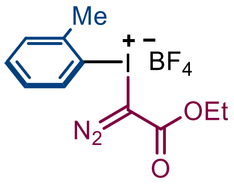

Prepared according to the **general procedure C**, **8j'** was collected as a yellow solid (94.1 mg, 75% yield). **^{1}H NMR** (400 MHz, Methanol-*d*$_4$) δ 8.53 – 8.15 (m, 1H), 7.68 (d, *J* = 7.3 Hz, 1H), 7.63 (d, *J* = 6.8 Hz, 1H), 7.37 (d, *J* = 7.8 Hz, 1H), 4.30 (q, *J* = 7.2 Hz, 2H), 2.74 (s, 3H), 1.27 (t, *J* = 7.2 Hz, 3H). **^{13}C NMR** (101 MHz, Methanol-*d*$_4$) δ 161.55, 141.30, 137.19, 133.77, 131.72, 129.31, 122.27, 63.81, 24.17, 13.20. **^{19}F NMR** (376 MHz, Chloroform-*d*) δ -152.29 (d, *J* = 331.3 Hz).

(1-Diazo-2-ethoxy-2-oxoethyl)(3-(trifluoromethyl)phenyl)iodonium trifluoromethanesulfonate (8k)

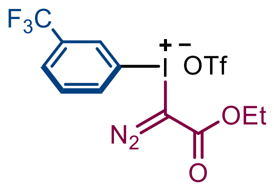

Prepared according to the **general procedure C**, **8k** was collected as a yellow solid (136.2 mg, 85% yield). **^{1}H NMR** (400 MHz, Chloroform-*d*) δ 8.38 (s, 1H), 8.34 (d, J = 8.3 Hz, 1H), 7.88 (d, J = 7.9 Hz, 1H), 7.61 (t, J = 8.1 Hz, 1H), 4.33 (q, J = 7.1 Hz, 2H), 1.32 (t, J = 7.1 Hz, 3H). **^{13}C NMR** (101 MHz, Chloroform-*d*) δ 162.25, 140.17, 133.52, 133.40 (q, J = 33.3), 133.13 (q, J = 4.0), 130.62 (q, J = 3.6), 123.02 (q, J = 273.7), 123.23, 118.33, 64.79, 14.45; **^{19}F NMR** (376 MHz, Chloroform-*d*) δ -62.75, -78.56.

(1-Diazo-2-ethoxy-2-oxoethyl)(3-(methoxycarbonyl)phenyl)iodonium tetrafluoroborate (8l)

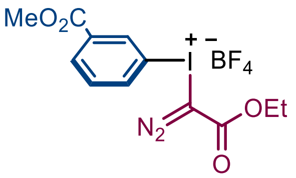

Prepared according to the **general procedure C**, **8l** was collected as a yellow solid (108.1 mg, 78% yield). **^{1}H NMR** (400 MHz, Methanol-*d*4) δ 8.82 (s, 1H), 8.39 (dd, J = 29.1, 7.9 Hz, 2H), 7.73 (t, J = 8.0 Hz, 1H), 4.34 (q, J = 7.3 Hz, 2H), 4.00 (s, 3H), 1.32 (t, J = 7.2 Hz, 3H). **^{13}C NMR** (101 MHz, Methanol-*d*4) δ 164.68, 161.91, 138.97, 135.54, 133.26, 131.77, 130.15, 117.97, 63.78, 52.02, 13.20. **^{19}F NMR** (376 MHz, Methanol-*d*4) δ -154.19 (d, J = 19.6 Hz). **HRMS** (ESI, m/z): calculated for $C_{12}H_{11}ClIN_2O_4^+$ $[M-BF_4]^+$: 408.9447, found: 408.9450.

(1-Diazo-2-ethoxy-2-oxoethyl)(2,4-difluorophenyl)iodonium tetrafluoroborate (8m)

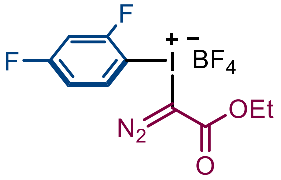

Prepared according to the **general procedure C**, **8m** was collected as a yellow solid (118.8 mg, 90% yield). **^{1}H NMR** (400 MHz, Chloroform-*d*) δ 8.25 (dt, J = 6.4, 9.0 Hz, 1H), 7.18 (td, J = 2.7, 8.8 Hz, 1H), 7.15 (td, J = 2.5, 8.6 Hz, 1H), 4.30 (q, J = 7.1 Hz, 2H), 1.30 (t, J = 7.2 Hz, 3H). **^{13}C NMR** (101 MHz, Chloroform-*d*) 166.9 (dd, J = 12.2, 258 Hz), 161.3 (dd, J = 14 Hz, 254 Hz), 160.4, 139.0 (d, J = 11 Hz), 115.4, (dd, J = 2.8, 23 Hz), 106.2 (t, J = 27 Hz, 1C), 96.5 (d, J = 23 Hz), 64.3, 41.3, 13.3. **^{19}F NMR** (376 MHz, Chloroform-*d*) δ -90.92(d, J = 12 Hz), -97.35 (d, J = 12 Hz), -144.62 – -145.00 (m).

(3-Chloro-5-(methoxycarbonyl)phenyl)(1-diazo-2-ethoxy-2-oxoethyl)iodonium tetrafluoroborate (8n)

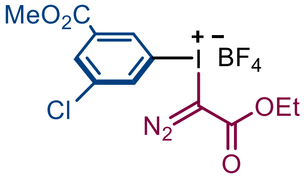

5n

Prepared according to the **general procedure C**, **8n** was collected as a yellow solid (116.2 mg, 78% yield). **^{1}H NMR** (400 MHz, Methanol-*d*4) δ 8.75 (s, 1H), 8.54 (s, 1H), 8.31 (s, 1H), 4.36 (q, J = 7.1 Hz, 2H), 4.01 (s, 3H), 1.33 (t, J = 7.1 Hz, 3H). **^{13}C NMR** (101 MHz, Methanol-*d*4) δ 163.64, 161.96, 138.20, 136.27, 134.39, 133.78, 133.24, 118.08, 63.83, 52.32, 13.22. **^{19}F NMR** (376 MHz, Methanol-*d*4) δ -154.19 (d, J = 19.6 Hz). **HRMS** (ESI, m/z): calculated for $C_{12}H_{11}ClIN_2O_4^+$ $[M-BF_4]^+$: 408.9447, found: 408.9450.

(2-(Benzyloxy)-1-diazo-2-oxoethyl)(phenyl)iodonium tetrafluoroborate (8o)

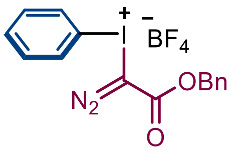

Prepared according to the **general procedure C**, **8o** was collected as a yellow solid (90.9 mg, 65% yield). **^{1}H NMR** (400 MHz, Chloroform-*d*) δ 7.72 (d, J = 25.0 Hz, 2H), 7.51 (s, 1H), 7.47 – 7.21 (m, 6H), 7.14 (s, 1H), 5.25 (s, 2H). **^{13}C NMR** (101 MHz, Chloroform-*d*) δ 163.98, 135.14, 133.77, 131.38, 130.00, 128.80, 128.56, 127.05, 122.38, 68.30. **^{19}F NMR** (376 MHz, Methanol-*d*4) δ -

154.19 (d, J = 19.6 Hz). **HRMS** (ESI, m/z): calculated for $C_{15}H_{12}IN_2O_2^+$ [M-BF$_4$]$^+$: 378.9938, found: 378.9930.

(2-(Allyloxy)-1-diazo-2-oxoethyl)(phenyl)iodonium tetrafluoroborate (8p)

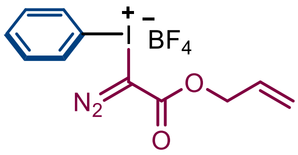

Prepared according to the **general procedure C**, **8p** was collected as a yellow solid (89.8 mg, 72% yield). **^{1}H NMR** (400 MHz, Methanol-d_4) δ 8.24 (d, J = 8.0 Hz, 2H), 7.80 (t, J = 7.5 Hz, 1H), 7.63 (t, J = 7.8 Hz, 2H), 5.96 (m, 1H), 5.48 – 5.18 (m, 2H), 4.78 (d, J = 5.8 Hz, 2H). **^{13}C NMR** (101 MHz, Methanol-d_4) δ 161.64, 135.02, 133.02, 131.84, 131.26, 118.63, 117.72, 67.87. **^{19}F NMR** (376 MHz, Methanol-d_4) δ -154.35 (d, J = 19.4 Hz). **HRMS** (ESI, m/z): calculated for $C_{11}H_{10}IN_2O_2^+$ [M-BF$_4$]$^+$: 328.9781, found: 328.9780.

(1-Diazo-2-oxo-2-(prop-2-yn-1-yloxy)ethyl)(phenyl)iodonium tetrafluoroborate (8q)

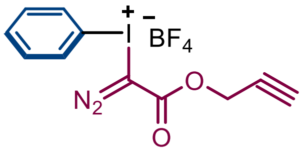

Prepared according to the **general procedure C**, **8q** was collected as a yellow solid (96.9 mg, 78% yield). **^{1}H NMR** (400 MHz, Methanol-d_4) δ 8.23 (d, J = 7.7 Hz, 2H), 7.76 (t, J = 7.5 Hz, 1H), 7.61 (t, J = 7.7 Hz, 2H), 4.80 (s, 2H), 3.09 (s, 1H). **^{13}C NMR** (101 MHz, Methanol-d_4) δ 161.80, 132.69, 131.70, 130.81, 118.99, 76.54, 76.44, 54.24. **^{19}F NMR** (376 MHz, Methanol-d_4) δ -153.06 (d, J = 18.9 Hz). **HRMS** (ESI, m/z): calculated for $C_{11}H_8IN_2O_2^+$ [M-BF$_4$]$^+$: 326.9625, found: 326.9628.

(1-Diazo-2-oxo-2-phenylethyl)(phenyl)iodonium trifluoromethanesulfonate (8r)

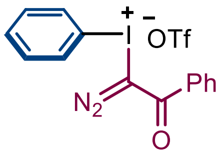

Prepared according to the **general procedure C**,**8r** was collected as a yellow solid (97.1 mg, 65% yield). **^{1}H NMR** (400 MHz, Chloroform-d) δ 8.19 (d, J = 10.6 Hz, 0H), 7.64 (d, J = 7.6 Hz, 1H), 7.59 – 7.53 (m, 0H), 7.47 (t, J = 6.7 Hz, 1H). **^{13}C NMR** (101 MHz, Methanol-d_4) δ 183.8, 137.2, 134.6, 134.5, 133.2, 131.6, 130.3, 128.9, 121.9 (q, J = 320.0 Hz), 116.4. **^{19}F NMR** (376 MHz, Chloroform-d) δ -78.10.

(1-Diazo-1-(diethoxyphosphonyl)methyl)(phenyl)iodonium trifluoromethanesulfonate (2ePO(OEt)$_2$) (8s)

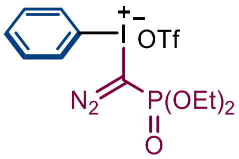

Prepared according to the **general procedure C**, **8s**was collected as a yellow solid (127.34 mg, 78% yield). **^{1}H NMR** (400 MHz, Chloroform-d) δ 8.17 - 8.15 (m, 2H), 7.80-7.76 (m, 1H), 7.62 - 7.58 (m, 2H), 3.62 (s, 3H), 3.60 (s, 3H). **^{13}C NMR** (101 MHz, Methanol-d_4) δ 136.8, 134.6, 133.2, 121.8 (q, J = 321.3 Hz), 117.6, 55.1, 55.1. **^{19}F NMR** (376 MHz, Chloroform-d) δ -78.10.

(1-Diazo-2-(((1R,2S,5R)-2-isopropyl-5-methylcyclohexyl)oxy)-2-oxoethyl)(phenyl)iodonium tetrafluoroborate (8t)

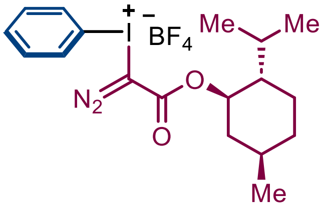

Prepared according to the **general procedure C**, **8t** was collected as a yellow solid (115.7 mg, 75% yield). **^{1}H NMR** (400 MHz, Methanol-d_4) δ 8.22 (d, J = 8.0 Hz, 2H), 7.77 (t, J = 7.7 Hz, 1H), 7.62 (t, J = 7.8 Hz, 2H), 4.84 (td, J = 11.1, 4.2 Hz, 1H), 1.95 (s, 1H), 1.81 – 1.59 (m, 2H), 1.54 – 1.37 (m, 3H), 1.10 (s, 2H), 1.06 (d, J = 11.5 Hz, 1H), 0.95 (d, J = 6.4 Hz, 4H), 0.88 (d, J = 7.0 Hz, 3H), 0.73 (d, J = 6.9 Hz, 3H). **^{13}C NMR** (101 MHz, Methanol-d_4) δ 161.59, 134.72, 132.66, 131.64, 118.72, 78.16, 40.63, 33.73, 31.38, 26.21, 23.12, 20.96, 19.70, 15.27. **^{19}F NMR** (376 MHz, Methanol-d_4) δ -153.99 (d, J = 19.8 Hz). **HRMS** (ESI, m/z): calculated for $C_{18}H_{24}IN_2O_2^+$ $[M-BF_4]^+$: 427.0877, found: 427.0880.

(1-diazo-2-((6-((1R,4aS,10aR)-7-isopropyl-1,4a-dimethyl-1,2,3,4,4a,9,10,10a-octahydrophenanthrene-1-carboxamido)hexyl)oxy)-2-oxoethyl)(phenyl)iodonium trifluoromethanesulfonate (8u)

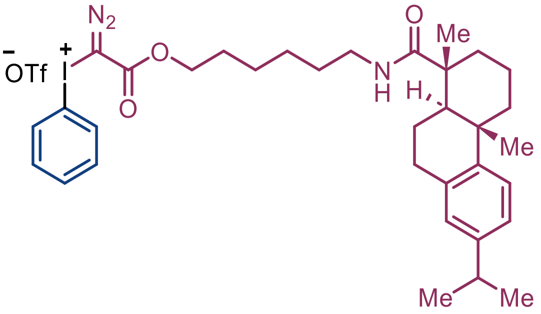

Prepared according to the **general procedure C**, **8u** was collected as a yellow solid (135.3 mg, 55% yield). **^{1}H NMR** (400 MHz, Chloroform-d) δ 8.14 (d, J = 7.9 Hz, 2H), 7.61 (t, J = 7.7 Hz, 1H), 7.48 (t, J = 8.1 Hz, 2H), 7.02 (d, J = 8.2 Hz, 1H), 6.90 (s, 1H), 4.24 – 4.18 (m, 2H), 3.28 (h, J = 7.0, 5.5 Hz, 2H), 2.96 – 2.77 (m, 3H), 2.33 (d, J = 12.9 Hz, 1H), 2.14 (d, J = 12.6 Hz, 1H), 1.92 – 1.72 (m, 6H), 1.71 – 1.61 (m, 5H), 1.55 – 1.47 (m, 4H), 1.28 (s, 3H), 1.24 (q, J = 2.3 Hz, 9H). **^{13}C NMR** (101 MHz, Chloroform-d) δ 178.48, 163.14, 147.07, 145.78, 134.67, 133.88, 131.88, 131.79, 126.96, 124.14, 123.90, 122.96, 120.90 (q, J = 316.6 Hz),67.09, 47.29, 45.60, 39.57, 37.37, 37.12, 33.50, 30.05, 29.54, 28.57, 26.96, 26.38, 25.39, 25.30, 24.05, 21.16, 18.81, 16.62. **^{19}F NMR** (376 MHz, Chloroform-d) δ -78.27. **HRMS** (ESI, m/z): calculated for $C_{34}H_{45}IN_3O_3^+$ $[M-OTf]^+$: 670.2500, found: 670.2500.

(1-diazo-2-((6-((9Z,12Z)-octadeca-9,12-dienamido)hexyl)oxy)-2-oxoethyl)(phenyl)iodonium trifluoromethanesulfonate (8v)

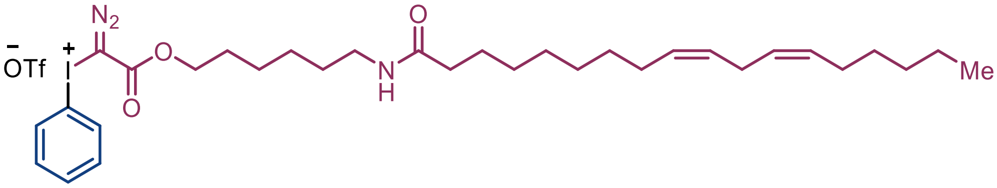

Prepared according to the **general procedure C**, **8v** was collected as a yellow solid (139.1 mg, 58% yield). **^{1}H NMR** (400 MHz, Chloroform-d) δ 8.07 (d, J = 7.9 Hz, 2H), 7.60 (t, J = 7.5 Hz, 1H), 7.46 (t, J = 7.8 Hz, 2H), 5.35 (q, J = 7.1 Hz, 2H), 4.33 – 4.10 (m, 3H), 3.23 (q, J = 6.8 Hz, 3H), 2.77 (t, J = 6.6 Hz, 1H), 2.16 (t, J = 7.8 Hz, 3H), 2.05 (s, 5H), 1.97 (s, 2H), 1.50 (t, J = 6.9 Hz, 4H), 1.33 (d, J = 16.1 Hz, 20H). **^{13}C NMR** (101 MHz, Chloroform-d) δ 173.42, 163.86, 133.85, 131.74, 131.63, 130.28, 130.10, 128.08, 127.94, 122.74, 66.81, 39.26, 36.86, 34.53, 31.55, 29.66, 29.52, 29.38, 29.34, 29.21, 28.57, 26.37, 25.88, 25.79, 25.66, 25.46, 22.61, 22.25, 21.32, 14.13. **^{19}F NMR** (376 MHz, Chloroform-d) δ -78.27. **HRMS** (ESI, m/z): calculated for $C_{32}H_{49}IN_3O_3^+$ $[M-OTf]^+$: 650.2813, found: 650.2810.

(1-Diazo-2-ethoxy-2-oxoethyl)(methyloxy carbonylphenyl)iodonium trifluoromethanesulfonate (8aa)

Prepared according to the **general procedure C**, **8aa** was collected as a yellow solid (133.7 mg, 85% yield).

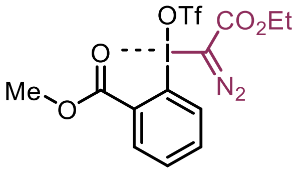

^{1}H NMR (400 MHz, Chloroform-*d*) δ 8.31 (d, *J* = 7.6 Hz, 1H), 7.97 (t, *J* = 7.8 Hz, 1H), 7.89 (d, *J* = 8.3 Hz, 1H), 7.81 (t, *J* = 7.5 Hz, 2H), 4.34 (q, *J* = 7.2 Hz, 2H), 4.16 (s, 3H), 1.30 (t, *J* = 7.2 Hz, 3H). **^{13}C NMR** (101 MHz, Chloroform-*d*) δ 170.36, 161.45, 138.17, 133.22, 132.08, 128.76, 125.76, 120.90(q, *J* = 316.6 Hz) 115.61, 64.39, 55.72, 14.22. **^{19}F NMR** (376 MHz, Chloroform-*d*) δ -78.38. **HRMS** (ESI, m/z): calculated for $C_{12}H_{12}IN_2O_4^+$ [M-OTf]$^+$: 374.9836, found: 374.9840.

(1-Diazo-2-ethoxy-2-oxoethyl)(2-((pentyloxy)carbonyl)phenyl)iodonium trifluoromethanesulfonate (8ab)

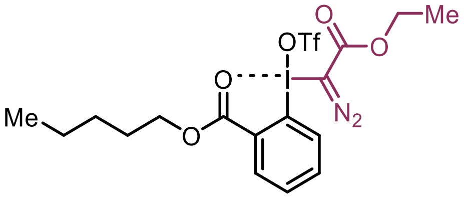

Prepared according to the **general procedure C**, **8ab** was collected as a yellow solid (125.3 mg, 72% yield). **^{1}H NMR** (400 MHz, Chloroform-*d*) δ 8.33 (d, *J* = 7.6 Hz, 1H), 7.98 (t, *J* = 7.8 Hz, 1H), 7.89 (d, *J* = 8.3 Hz, 1H), 7.83 (t, *J* = 7.4 Hz, 1H), 4.57 (t, *J* = 6.7 Hz, 2H), 4.38 (q, *J* = 6.9 Hz, 2H), 1.88 (t, *J* = 7.1 Hz, 2H), 1.46 (m, 4H), 1.34 (t, *J* = 7.2 Hz, 3H), 0.97 (t, *J* = 7.0 Hz, 3H). **^{13}C NMR** (101 MHz, Chloroform-*d*) δ 169.85, 161.48, 137.94, 133.06, 131.97, 128.70, 126.11, 120.90 (q, *J* = 316.6 Hz), 115.78, 69.83, 64.40, 28.07, 27.88, 22.27, 14.27, 13.95. **^{19}F NMR** (376 MHz, Chloroform-*d*) δ -78.35. **HRMS** (ESI, m/z): calculated for $C_{16}H_{20}IN_2O_4^+$ [M-OTf]$^+$: 431.0462, found: 431.0465.

(1-Diazo-2-(((1R,2S,5R)-2-isopropyl-5-methylcyclohexyl)oxy)-2-oxoethyl)(2-(methoxycarbonyl)phenyl)iodonium trifluoromethanesulfonate (8ac)

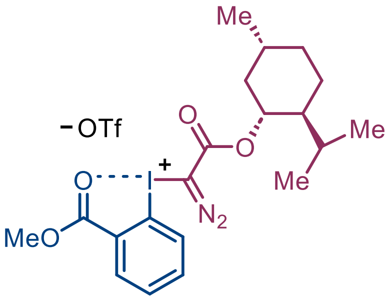

Prepared according to the **general procedure C**, **8ac** was collected as a yellow solid (133.2 mg, 70% yield). **^{1}H NMR** (400 MHz, Chloroform-*d*) δ 8.30 (d, *J* = 7.6 Hz, 1H), 7.87 (t, *J* = 7.7 Hz, 1H), 7.78 (t, *J* = 7.3 Hz, 3H), 4.83 (td, *J* = 11.0, 4.3 Hz, 1H), 4.17 (s, 3H), 2.06 – 1.99 (m, 1H), 1.66 (d, *J* = 12.1 Hz, 3H), 1.53 – 1.45 (m, 2H), 1.15 – 1.05 (m, 2H), 0.92 – 0.89 (m, 4H), 0.85 (d, *J* = 6.3 Hz, 3H), 0.73 (d, *J* = 6.9 Hz, 3H). **^{13}C NMR** (101 MHz, Chloroform-*d*) δ 170.13, 160.97, 137.50, 133.14, 131.87, 128.64, 126.19, 120.90 (q, *J* = 316.6 Hz), 116.35, 79.20, 55.73, 47.03, 40.75, 33.95, 31.52, 23.32, 21.94, 20.72, 16.21. **^{19}F NMR** (376 MHz, Chloroform-*d*) δ -78.35. **HRMS** (ESI, m/z): calculated for $C_{20}H_{26}IN_2O_4^+$ [M-OTf]$^+$: 485.0932, found: 485.0930.

(1-Diazo-2-ethoxy-2-oxoethyl)(2-(2-ethoxy-2-oxoethoxy)carbonylphenyl)iodonium trifluoromethanesulfonate (8ad)

Prepared according to the **general procedure C**, **8ad** was collected as a yellow solid (89.5 mg, 50% yield).

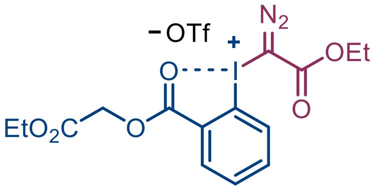

^{1}H NMR (400 MHz, Chloroform-*d*) δ 8.40 (dd, *J* = 7.6, 1.6 Hz, 1H), 7.95 (ddd, *J* = 8.8, 7.2, 1.7 Hz, 1H), 7.86 (dd, *J* = 8.3, 1.1 Hz, 1H), 7.81 (td, *J* = 7.4, 1.1 Hz, 1H), 5.03 (s, 2H), 4.32 (dq, *J* = 19.7, 7.2 Hz, 4H), 1.31 (dt, *J* = 10.7, 7.3 Hz, 6H). **^{13}C NMR** (101 MHz, Chloroform-*d*) δ 169.3, 165.8, 161.1, 138.2, 133.5, 131.9, 128.9, 125.4, 120.3 (q, *J* = 319.5 Hz), 116.1, 64.4, 63.8, 62.4, 14.2, 14.0. **^{19}F NMR** (376 MHz, DMSO-d_6) δ -77.74.

Ethyl 1-benzoyl-1H-diazirine-3-carboxylate (10)

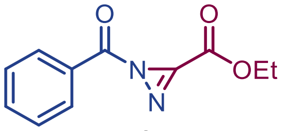

8

Prepared according to the general procedure, **10** was collected as a white solid (46.4 mg, 85% yield). **^{1}H NMR** (400 MHz, Chloroform-*d*) δ 8.24 – 8.16 (m, 2H), 7.69 – 7.61 (m, 1H), 7.61 – 7.52 (m, 2H), 4.59 (q, *J* = 7.1 Hz, 2H), 1.52 (t, *J* = 7.1 Hz, 3H). **^{13}C NMR** (101 MHz, Chloroform-*d*) δ 166.56, 156.57, 154.52, 132.92, 129.33, 127.71, 122.81, 63.63, 14.18.

Ethyl 2-diazo-2-(2-(dimethylamino)phenyl)acetate (12)

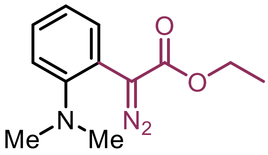

Prepared according to the general procedure, **12** was collected as a yellow solid (139.9 mg, 60% yield). **^{1}H NMR** (400 MHz, Chloroform-*d*) δ 7.56 (dd, *J* = 7.9, 1.5 Hz, 1H), 7.27 (td, *J* = 7.7, 1.7 Hz, 1H), 7.14 – 7.05 (m, 2H), 4.36 (q, *J* = 7.1 Hz, 2H), 2.74 (s, 6H), 1.37 (t, *J* = 7.1 Hz, 3H). **^{13}C NMR** (101 MHz, Chloroform-*d*) δ 150.75, 131.03, 128.27, 122.91, 119.02, 118.69, 61.00, 43.10, 14.62.

Ethyl 2-diazo-4-methoxy-4-phenylbutanoate (14)

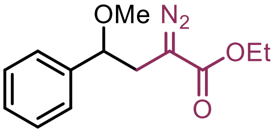

Prepared according to the general procedure, **14** was collected as a yellow oil (37.2 mg, 75%). **^{1}H NMR** (400 MHz, Chloroform-*d*) δ 7.39 – 7.29 (m, 5H), 4.31 (dd, *J* = 8.3, 4.7 Hz, 1H), 4.20 (q, *J* = 7.1 Hz, 2H), 3.24 (s, 3H), 2.66 (dd, *J* = 15.2, 8.3 Hz, 1H), 2.57 (dd, *J* = 15.1, 4.7 Hz, 1H), 1.26 (t, *J* = 7.1 Hz, 3H).**^{13}C NMR** (101 MHz, Chloroform-*d*) δ 140.81, 128.66, 128.09, 126.51, 82.80, 60.81, 56.87, 14.56.

Ethyl-2-naphthoate (15)

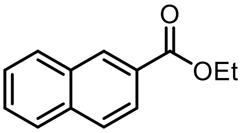

Prepared according to the general procedure, **15** was collected as a yellow oil (39.4 mg, 82%).**^{1}H NMR** (400 MHz, Chloroform-*d*) δ 8.66 (s, 1H), 8.16 – 7.97 (m, 2H), 7.91 (d, *J* = 8.9 Hz, 2H), 7.66 – 7.53 (m, 2H). 4.49 (q, *J* = 7.1 Hz, 2H), 1.49 (t, *J* = 7.2 Hz, 3H). **^{13}C NMR** (101 MHz, Chloroform-*d*) δ 166.87, 135.56, 132.58, 131.02, 129.41, 128.22, 128.16, 127.82, 126.66, 125.33, 61.17, 14.47.

3-(Azidomethyl)-1,3-dimethylindolin-2-one (19a)

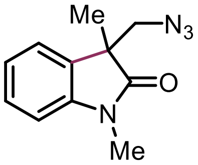

Prepared according to the general procedure, **19a** was collected as a yellow oil (21.6 mg, 50% yield). **^{1}H NMR** (400 MHz, Chloroform-*d*) 7.31 (td, J = 7.8, 1.2 Hz, 1H), 7.28 – 7.25 (m, 1H), 7.09 (td, J = 7.5, 1.2 Hz, 1H), 6.87 (d, J = 7.8 Hz, 1H), 3.64 –

3.60 (m, 2H), 3.22 (s, 3H), 1.36 (s, 3H). **^{13}C NMR** (101 MHz, Chloroform-*d*) δ 178.27, 143.54, 131.44, 128.78, 123.10, 122.87, 108.44, 57.32, 48.84, 26.45, 20.53.

3-(Azidomethyl)-1,3,5-trimethylindolin-2-one (19b)

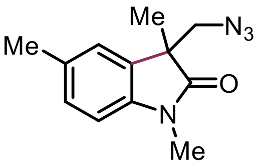

Prepared according to the general procedure, **19b** was collected as a yellow oil (20.3 mg, 44% yield). **^{1}H NMR** (400 MHz, Chloroform-*d*) δ 7.18 – 7.11 (m, 2H), 6.80 (d, *J* = 7.8 Hz, 1H), 3.66 (s, 2H), 3.25 (s, 3H), 2.40 (s, 3H), 1.40 (s, 3H). **^{13}C NMR** (101 MHz, Chloroform-*d*) δ 178.22, 141.13, 132.44, 131.49, 128.97, 123.95, 108.15, 57.33, 48.84, 26.45, 21.19, 21.14, 20.57.

3-(Azidomethyl)-5-chloro-1,3-dimethylindolin-2-one (19c)

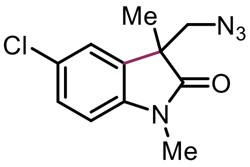

Prepared according to the general procedure, **19c** was collected as a yellow oil (30 mg, 60% yield). **^{1}H NMR** (400 MHz, Chloroform-*d*) δ 7.36 – 7.27 (m, 2H), 6.84 (d, *J* = 8.3 Hz, 1H), 3.68 (s, 2H), 3.26 (s, 3H), 1.41 (s, 3H). **^{13}C NMR** (101 MHz, Chloroform-*d*) δ 177.83, 142.06, 133.20, 128.66, 128.33, 123.76, 109.35, 57.07, 49.03, 26.57, 20.42.

3-(Azidomethyl)-1-ethyl-3-methylindolin-2-one (19d)

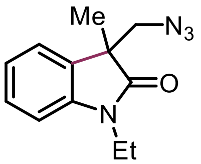

Prepared according to the general procedure, **19d** was collected as a yellow oil (21.6 mg, 47% yield). **^{1}H NMR** (400 MHz, Chloroform-*d*) δ 7.40 – 7.26 (m, 2H), 7.13 (td, *J* = 7.5, 1.0 Hz, 1H), 6.94 (d, *J* = 7.8 Hz, 1H), 3.92 – 3.70 (m, 2H), 3.66 (s, 2H), 1.41 (s, 3H), 1.32 (t, *J* = 7.2 Hz, 3H). **^{13}C NMR** (101 MHz, Chloroform-*d*) δ 177.88, 142.64, 131.73, 128.69, 123.26, 122.62, 108.57, 57.41, 48.76, 34.86, 20.48, 12.66.

3-(Azidomethyl)-1-benzyl-3-methylindolin-2-one (19e)

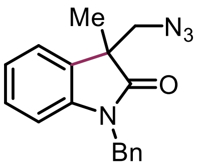

Prepared according to the general procedure, **19e** was collected as a colorless oil (28.6 mg, 49% yield). **^{1}H NMR** (400 MHz, Chloroform-*d*) δ 7.38 – 7.25 (m, 6H), 7.24 (td, *J* = 7.7, 1.3 Hz, 1H), 7.10 (td, *J* = 7.5 Hz, 1H), 6.78 (d, *J* = 7.8 Hz, 1H), 5.13 – 4.82 (m, 2H), 3.75 (s, 2H).1.47 (s, 3H). **^{13}C NMR** (101 MHz, Chloroform-*d*) δ 178.45, 142.67, 135.69, 131.43, 128.88, 128.68, 127.69, 127.17, 123.08, 122.87, 109.52, 57.50, 48.89, 43.87, 20.74.

1-Chloro-4-(1,2-dichloroethyl)benzene (20)

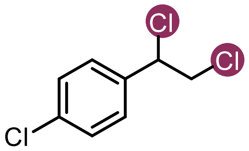

Prepared according to the general procedure, **22** was collected as a colorless oil (27.2 mg, 65%).**^{1}H NMR** (400 MHz, Chloroform-*d*) δ 7.39 (m, 4H), 5.00 (dd, *J* = 8.4, 6.1 Hz, 1H), 4.02 (dd, *J* = 11.3, 6.1 Hz, 1H), 3.91 (dd, *J* = 11.3, 8.4 Hz, 1H). **^{13}C NMR** (101 MHz, Chloroform-*d*) δ 136.55, 135.11, 129.10, 128.90, 60.71, 48.09.

Methyl 4-(2,3-dichloropropoxy)benzoate (21)

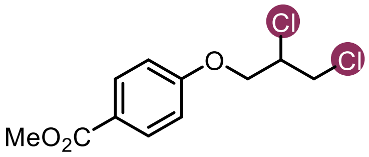

Prepared according to the general procedure, **23** was collected as a colorless oil (36.3 mg, 69%).**^{1}H NMR** (400 MHz, Chloroform-*d*) δ 8.04 (d, *J* = 8.9 Hz, 2H), 6.98 (d, *J* = 8.9 Hz, 2H), 4.50 – 4.22 (m, 3H), 3.95 – 4.10 (m, *J* = 16.9 Hz, 5H). **^{13}C NMR** (101 MHz, Chloroform-*d*) δ 166.71, 161.65, 131.78, 123.65, 114.32, 68.16, 56.98, 52.03, 44.78. **HRMS** (ESI, m/z): calculated for $C_{11}H_{13}Cl_2O_3^+$ [M+H]$^+$: 263. 0236, found: 263. 0235.

5-(Chloromethyl)-5-phenyldihydrofuran-2(3H)-one (22)

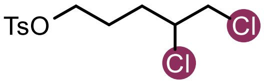

Prepared according to the general procedure, **24** was collected as a colorless oil (49 mg, 79%).**^{1}H NMR** (400 MHz, Chloroform-*d*) δ 7.82 (d, *J* = 8.8 Hz, 2H), 7.39 (d, *J* = 7.8 Hz, 2H), 4.14 – 4.09 (m, 2H), 4.03 – 3.97 (h, *J* = 4.5 Hz, 1H), 3.79 – 3.75 (m, 1H), 3.64 – 3.60 (m, 1H), 2.49 (s, 3H), 2.16 – 2.06 (m, 1H), 2.03 – 1.98 (m, 1H), 1.85 – 1.69 (m, 2H). **^{13}C NMR** (101 MHz, Chloroform-*d*) δ 145.03, 132.95, 130.00, 127.97, 69.54, 60.15, 47.97, 31.18, 25.57, 21.72.

Tert-butyl-2-chloro-1-oxo-indan-2-carboxylate (23)

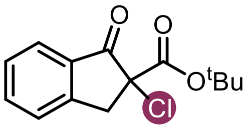

Prepared according to the general procedure, **25** was collected as a colorless oil (40 mg, 75%).**^{1}H NMR** (400 MHz, Chloroform-d) δ 7.87 (d, *J* = 8.7 Hz, 1H), 7.77 – 7.68 (m, 1H), 7.58 – 7.41 (m, 2H), 3.76 (dd, *J* = 17.5, 10.8 Hz, 1H), 3.44 (dd, *J* = 22.9, 17.5 Hz, 1H), 1.47 (s, 9H). **^{13}C NMR** (101 MHz, Chloroform-*d*) δ 195.65, 165.95, 150.72, 136.24, 132.87, 128.51, 126.27, 125.87, 84.48, 68.81, 43.58, 27.75.

1-(1,2-Difluoroethyl)-4-nitrobenzene (24)

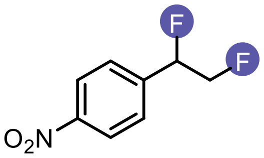

Prepared according to the general procedure, **24** was collected as a yellow solid (32.9 mg, 88%).**^{1}H NMR** (400 MHz, Chloroform-*d*) δ = 8.28 (m, 2H), 7.57 (m, 2H), 5.81 (ddd, *J* = 47.9, 7.0, 3.1 Hz, 1H), 4.54 – 4.31 (m, 2H). **^{13}C NMR** (101 MHz, Chloroform-*d*) δ = 148.5, 141.9 (dd, $^{2}J_{CF}$ = 20.5 Hz, 3JCF = 6.6 Hz), 126.9 (d, $^{3}J_{CF}$ = 7.5 Hz), 124.1, 91.1 (dd, $^{1}J_{CF}$ = 179.8 Hz, $^{2}J_{CF}$ = 20.5 Hz), 84.1 (dd, $^{1}J_{CF}$ = 180.1 Hz, $^{2}J_{CF}$ = 24.8 Hz). **^{19}F NMR** (376 MHz, Chloroform-*d*) δ = -190.0 (dddd, $^{2}J_{FH}$ = 47.3 Hz, $^{3}J_{FH}$ = 24.8, 23.0 Hz, $^{3}J_{FF}$ = 14.4 Hz, 1F), -226.4 (tdd, $^{2}J_{FH}$ = 47.1 Hz, $^{3}J_{FH}$ = 17.9 Hz, $^{3}J_{FF}$ = 14.4 Hz, 1F).

Methyl 3-fluorochromane-6-carboxylate (25)

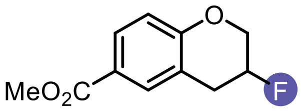

Prepared according to the general procedure, **25** was collected as a colorless oil (24.4 mg, 58%).**^{1}H NMR** (400 MHz, Chloroform-*d*) δ 7.84 (d, *J* = 10.1 Hz, 3H), 6.91 (d, *J* = 8.4 Hz, 2H), 5.17 (dd, *J* = 47.2, 4.1 Hz, 1H), 4.51 – 4.42 (m, 1H), 4.29 – 4.09 (m, 2H), 3.91 (s, 4H), 3.33 – 3.02 (m, 4H). **^{13}C NMR** (101 MHz, Chloroform-*d*) δ 166.78, 157.57, 132.25, 129.69, 123.10, 117.91, 116.68, 83.55 (d, *J* = 175.5 Hz), 67.33 (d, *J* = 21.9 Hz), 51.95, 30.38 (d, *J* = 23.0 Hz). **^{19}F NMR** (376 MHz, Chloroform-*d*) δ -187.31 (dddd, *J* = 79.5, 31.9, 19.6, 9.4 Hz).

Methyl 4-(2,4-difluorobutoxy)benzoate (26)

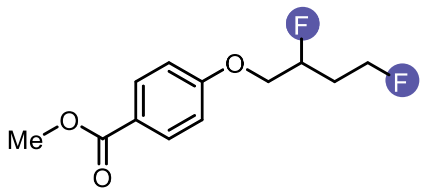

Prepared according to the general procedure, **26** was collected as a colorless oil (20.5 mg, 42%).**^{1}H NMR** (400 MHz, Chloroform-*d*) δ 8.03 (d, *J* = 8.8 Hz, 2H), 6.97 (d, *J* = 8.5 Hz, 2H), 5.19 – 4.99 (m, 1H), 4.82 – 4.56 (m, 2H), 4.32 – 4.14 (m, 2H), 3.92 (s, 3H), 2.36 – 2.04 (m, 2H). **^{13}C NMR** (101 MHz, Chloroform-*d*) δ 166.8, 162.1, 131.8, 123.4, 114.3, 88.1 (dd, *J* = 173.6, 3.4 Hz,), 80.0 (dd, *J* = 165.6, 5.0 Hz), 69.5 (d, *J* = 22.7 Hz), 52.0, 32.7 (t, *J* = 20.5 Hz). **^{19}F NMR** (376 MHz, Chloroform-*d*) δ -190.38 (dp, *J* = 45.3, 22.5 Hz), -221.42 (ddd, *J* = 74.2, 49.1, 27.3 Hz).

4-Fluoro-4-methyl-2-phenyl-4,5-dihydrobenzo[d]-1,3-oxazepine (27)

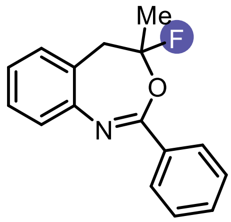

Prepared according to the general procedure, **27** was collected as a white solid (44.9 mg, 88%).**^{1}H NMR** (400 MHz, Chloroform-*d*) δ 8.24 (d, *J* = 9.7 Hz, 2H), 7.52 (dq, *J* = 14.6, 8.3, 7.7 Hz, 3H), 7.44 – 7.38 (m, 1H), 7.35 (d, *J* = 7.8 Hz, 1H), 7.30 – 7.20 (m, 2H), 3.27 (dd, *J* = 14.2, 9.2 Hz, 1H), 3.13 (dd, *J* = 14.1, 2.6 Hz, 1H), 1.74 (d, *J* = 17.7 Hz, 3H). **^{13}C NMR** (101 MHz, Chloroform-*d*) δ = 153.3 (d, *J* = 2.3 Hz), 144.3, 133.7, 131.4, 129.1, 128.9, 128.4, 128.3, 127.5 (d, *J* = 9.6 Hz), 125.7, 125.5, 122.9 (d, *J* = 228.4 Hz), 42.36 (d, *J* = 30.9 Hz), 24.61 (d, *J* = 29.9 Hz). **^{19}F NMR** (376 MHz, Chloroform-*d*) δ -71.98 – -72.32 (m).

5-Fluoro-3,3-diphenyl-1-tosylpiperidine (28)

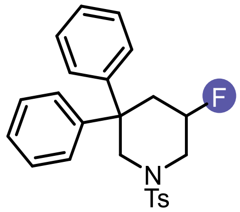

Prepared according to the general procedure, **28** was collected as a white solid (52.2 mg, 65%).**^{1}H NMR** (400 MHz, Chloroform-*d*) δ 7.64 (d, J = 8.2 Hz, 2H), 7.47 (d, J = 7.7 Hz, 2H), 7.36 – 7.12 (m, 10H), 4.64 – 4.42 (m, 2H), 4.07 – 3.97 (m, 1H), 3.01 – 2.90 (m, 1H), 2.47 – 2.35 (m, 4H), 2.29 (td, J = 10.0, 5.5 Hz, 1H), 2.16 (dd, J = 20.3, 11.9 Hz, 1H). **^{13}C NMR** (101 MHz, Chloroform-*d*) δ 145.3, 144.1, 143.1, 132.1, 129.9, 128.6, 128.6, 127.7, 127.67, 126.8, 126.5, 126.4, 85.54 (d, J = 173.7 Hz), 53.8, 49.79 (d, J = 31.1 Hz), 46.40 (d, J = 11.0 Hz), 41.01 (d, J = 18.8 Hz), 21.5. **^{19}F NMR** (376 MHz, Chloroform-*d* 3) δ -185.51.

(E)-N-(2-bromo-4-fluoro-4-methylcyclohexa-2,5-dien-1-ylidene)-4-methylbenzenesulfonamide (29)

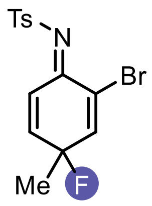

Prepared according to the general procedure, **29** was collected as a white solid (64.5 mg, 90%).**^{1}H NMR** (400 MHz, Chloroform-*d*) δ 7.95 (d, *J* = 8.3 Hz, 2H), 7.60 (d, *J* = 10.1 Hz, 1H), 7.38 (d, *J* = 8.0 Hz, 2H), 7.33 – 7.23 (m, 1H), 6.91 – 6.82 (m, 1H), 2.48 (s, 3H), 1.67 (d, *J* = 21.0 Hz, 3H). **^{13}C NMR** (101 MHz, Chloroform-*d*) δ 158.80 (d, *J* = 6.1 Hz), 146.60 (d, *J* = 22.3 Hz), 145.14 (d, *J* = 21.0 Hz), 144.13, 137.69, 129.57, 127.21, 121.23 (d, *J* = 8.3 Hz), 88.07 (d, *J* = 166.5 Hz), 25.20 (d, *J* = 27.3 Hz), 21.61. **^{19}F NMR** (376 MHz, Chloroform-*d*) δ -142.69 (m). **HRMS** (ESI, m/z): calculated for $C_{14}H_{14}BrFNO_2S^+$ $[M+H]^+$: 357.9907, found: 357.9917.

4-fluoroisochroman-1-one (30)

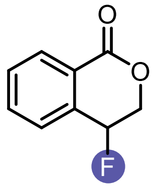

Prepared according to the general procedure, **30** was collected as a colorless oil (22.6 mg, 68%). **^{1}H NMR** (400 MHz, Chloroform-*d*) δ 8.21 (d, *J* = 7.5 Hz, 1H), 7.72 (t, *J* =7.5 Hz, 1H), 7.64 (*t*, J = 7.5 Hz, 1H), 7.56 (d, *J* = 7.5 Hz, 1H), 5.59 (ap. dt, *J* = 49.5, 2.5 Hz, 1H), 4.86 (ddd, *J* = 16.1, 13.2 Hz, 3.4 Hz, 1H), 4.64 (ddd, *J* = 33.5, 12.5, 2.5 Hz, 1H). **^{13}C NMR** (101 MHz, Chloroform-*d*) δ 163.1, 134.5 (d, *J* = 17.5 Hz), 134.4 (d, *J* = 3.0 Hz), 131.2 (d, *J* = 3.5 Hz), 130.5 (d, *J* = 2.0 Hz), 128.1 (d, *J* = 3.5 Hz), 124.8 (d, *J* = 2.0 Hz), 82.9 (d, *J* = 177.5 Hz), 69.7 (d, *J* = 24.5 Hz). **^{19}F NMR** (376 MHz, Chloroform-*d*) δ -171.5 (ddd, *J* = 49.5, 34.0, 12.0 Hz).

5-(2-Fluoroethyl)-2,5-diphenyl-4,5-dihydrooxazole (31)

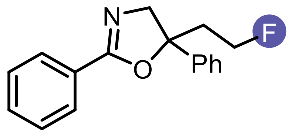

Prepared according to the general procedure, **31** was collected as a colorless oil (29.1 mg, 54%)**^{1}H NMR** (400 MHz, Chloroform-*d*) δ 8.08 (d, *J* = 7.4 Hz, 2H), 7.66 – 7.29 (m, 8H), 4.80 – 4.48 (m, 2H), 4.46 – 4.19 (m, 2H), 2.65 – 2.47 (m, 2H). **^{13}C NMR** (101 MHz, Chloroform-*d*) δ 143.54, 131.67, 128.81, 128.55, 128.31, 127.74, 124.42, 87.38 (d, *J* = 7.4 Hz), 80.25 (d, *J* = 165.2 Hz), 67.94, 41.56 (d, *J* = 19.5 Hz). **^{19}F NMR** (376 MHz, Chloroform-*d*) δ -220.61 (tt, *J* = 45.9, 20.9 Hz). **HRMS** (ESI, m/z): calculated for $C_{17}H_{17}FNO^{+}$ $[M+H]^{+}$: 270.1289, found: 270.1289.

(E)-4-fluoroisochroman-1-one O-methyl oxime (32)

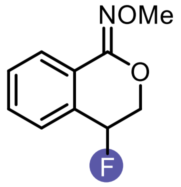

Prepared according to the general procedure, **32** was collected as a white solid (28.9 mg, 74%).**^{1}H NMR** (400 MHz, Chloroform-*d*) δ 8.08 – 7.98 (m, 1H), 7.50 (qd, *J* = 5.8, 3.4 Hz, 3H), 5.56 (dt, *J* = 49.3, 2.5 Hz, 1H), 4.74 (ddd, *J* = 12.1, 10.8, 2.8 Hz, 1H), 4.43 (ddd, *J* = 31.3, 12.6, 2.1 Hz, 1H), 4.01 (d, *J* = 1.3 Hz, 3H). **^{13}C NMR** (101 MHz, Chloroform-*d*) δ 148.59, 130.61 (d, *J* = 3.2 Hz), 130.03 (d, *J* = 17.9 Hz), 128.51 (d, *J* = 4.1 Hz), 125.36, 125.19, 83.65 (d, *J* = 176.4 Hz), 68.74 (d, *J* = 24.0 Hz), 62.93. **^{19}F NMR** (376 MHz, Chloroform-*d*) δ -172.29 (t, *J* = 40.5 Hz). **HRMS** (ESI, m/z): calculated for $C_{10}H_{11}FNO_2^{+}$ $[M+H]^{+}$: 196.0768, found: 196.0768.

((S)-3-Amino-3-carboxypropyl)(1-diazo-2-ethoxy-2-oxoethyl)(methyl)sulfonium tetrafluoroborate (33)

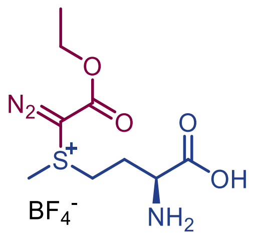

Prepared according to the general procedure, **33** was collected as a yellow solid (78.5 mg, 75% yield). **^{1}H NMR** (400 MHz, Deuterium Oxide) δ 4.53 (t, *J* = 9.2 Hz, 1H), 4.42 – 4.23 (m, 3H), 4.13 (q, *J* = 6.7 Hz, 1H), 2.78 – 2.62 (m, 2H), 2.44 – 2.31 (m, 3H), 1.27 (t, *J* = 7.2 Hz, 3H). **^{13}C NMR** (101 MHz, Deuterium Oxide) δ 174.60, 161.42, 67.41, 64.65, 51.38, 51.12, 48.58, 40.66, 36.50, 26.75, 24.96, 13.39. **^{19}F NMR** (376 MHz, Deuterium Oxide) δ -150.38 (d, *J* = 19.1 Hz). **HRMS** (ESI, m/z): calculated for $C_9H_{16}N_4O_4S^{+}$ $[M-BF_4]^{+}$: 262.0865, found: 262.0862.

((S)-3-Amino-4-((carboxymethyl)amino)-4-oxobutyl)(1-diazo-2-ethoxy-2-oxoethyl)(methyl)sulfonium tetrafluoroborate (34)

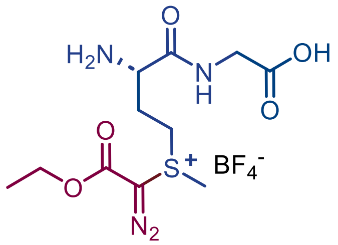

Prepared according to the general procedure, **34** was collected as a yellow solid (82.9 mg, 68% yield). **^{1}H NMR** (400 MHz, Deuterium Oxide) δ 4.39 (app q, *J* = 6.9 Hz, 2H), 4.24 (app t, *J* = 8.5 Hz, 1H), 4.06 – 3.97 (m, 3H), 3.80-3.71 (m, 1H), 3.31 (d, *J* = 2.0 Hz, 3H), 2.56 – 2.38 (m, 2H), 1.32 (app t, *J* = 7.4 Hz, 3H). **^{13}C NMR** (101 MHz, Deuterium Oxide) δ 172.83, 168.33, 168.23, 161.33, 64.66, 51.41, 51.30, 41.38, 39.56, 26.49, 25.74, 25.68, 13.40. **^{19}F NMR** (376 MHz, Deuterium Oxide) δ -150.19 (d, *J* = 19.7 Hz). **HRMS** (ESI, m/z): calculated for $C_{11}H_{19}N_4O_5S^+$ $[M-BF_4]^+$: 319.1071, found: 319.1070.

(1-Diazo-2-ethoxy-2-oxoethyl)(phenyl)- λ_3-iodaneyl 3-chlorobenzoate (DICB)

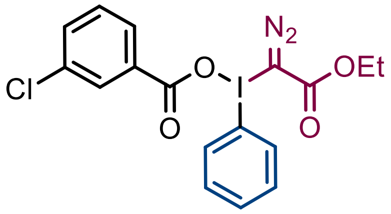

Prepared according to the general procedure, **DICB** was collected as a yellow solid (112.0 mg, 79% yield). **^{1}H NMR** (400 MHz, Chloroform-*d*) δ 8.12 (d, *J* = 8.1 Hz, 1H), 8.07 (s, 1H), 7.99 (d, *J* = 7.8 Hz, 1H), 7.60 (dd, *J* = 17.0, 7.9 Hz, 2H), 7.45 (dt, *J* = 20.5, 7.6 Hz, 3H), 7.36 – 7.27 (m, 1H), 4.33 – 4.23 (m, 2H), 1.30 (t, *J* = 10.2 Hz, 3H). **^{13}C NMR** (101 MHz, Chloroform-*d*) δ 169.73, 163.05, 137.52, 134.36, 133.52, 132.15, 131.74, 130.21, 129.82, 128.28, 121.16, 63.42, 14.35. **HRMS** (ESI, m/z): calculated for $C_{17}H_{16}ClN_2O_4^+$ $[M+H]^+$: 472.9760, found: 472.9765.

19 NMR spectra of compounds

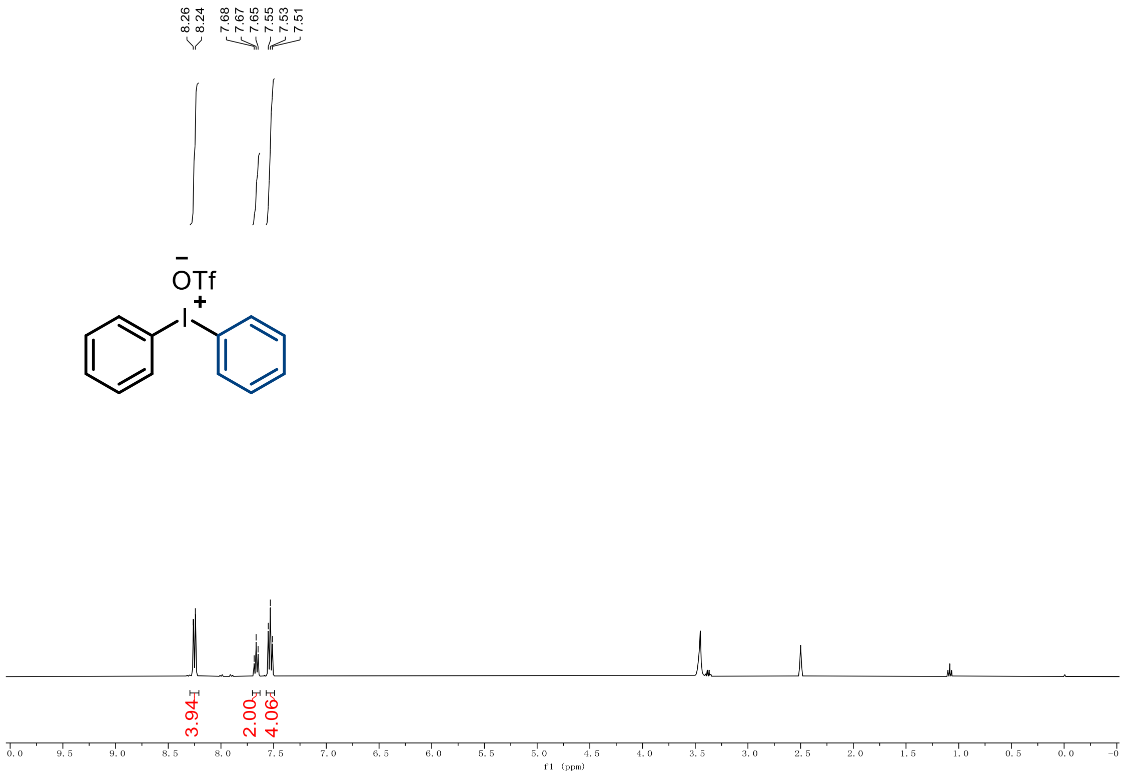

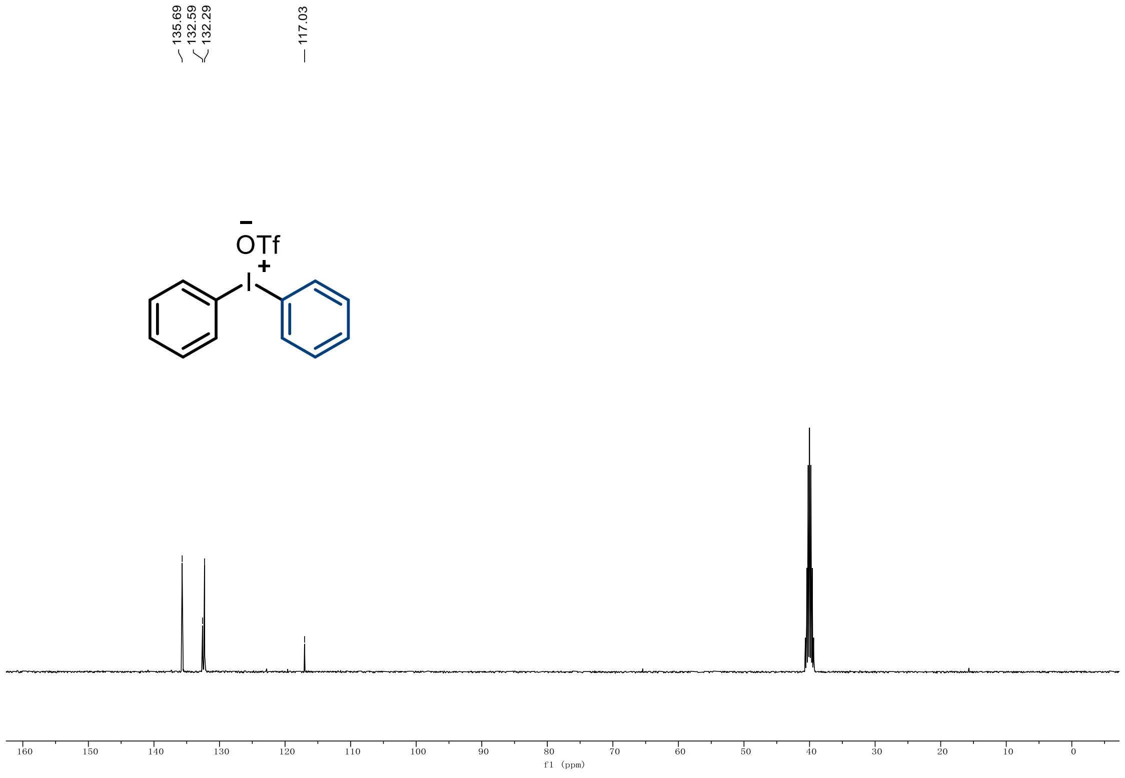

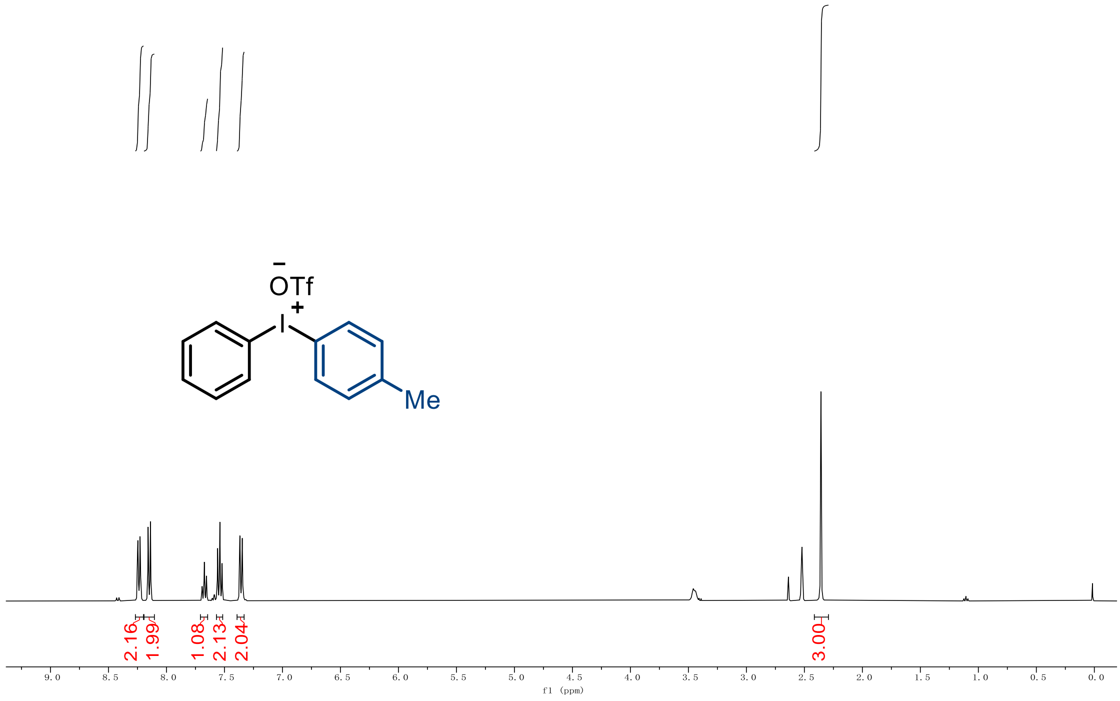
OTf
I
Me
2.16
1.99
1.08
2.13
2.04
3.00
9.0
8.5
8.0
7.5
7.0
6.5
6.0
5.5
5.0
4.5
4.0
3.5
3.0
2.5
2.0
1.5
1.0
0.5
0.0
f1 (ppm)

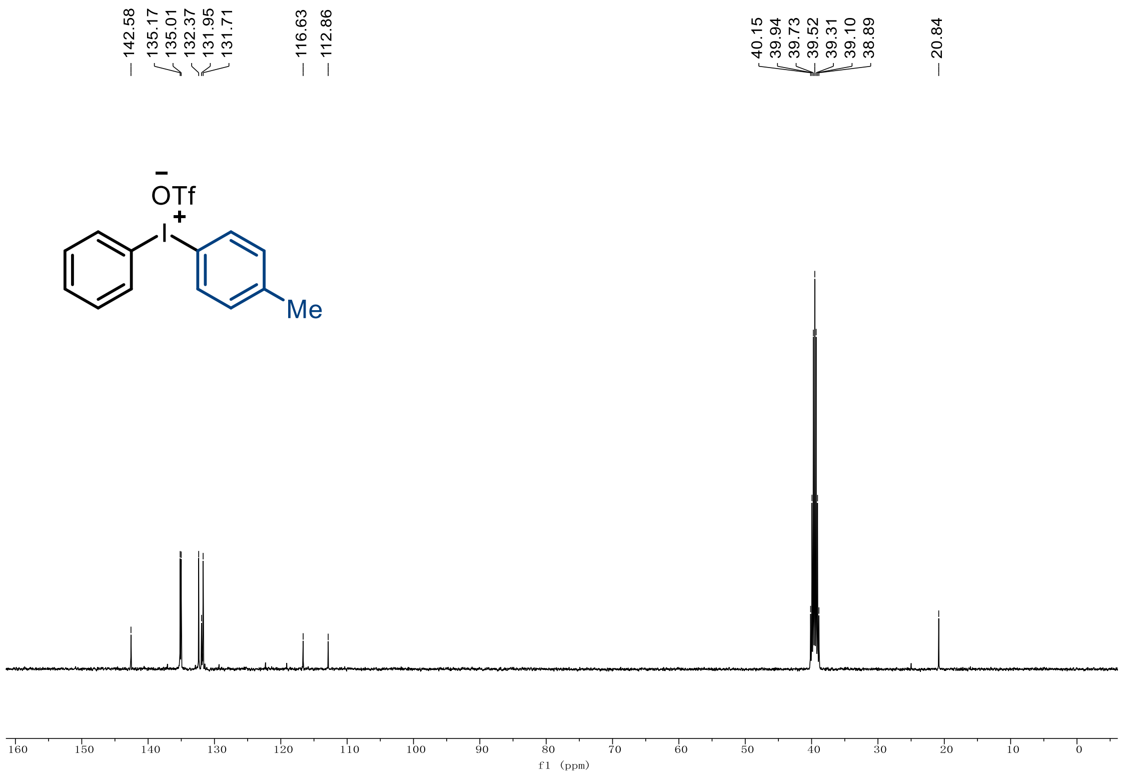
142.58
135.17
135.01
132.37
131.95
131.71
116.63
112.86
40.15
39.94
39.73
39.52
39.31
39.10
38.89
20.84
OTf
I
Me
160
150
140
130
120
110
100
90
80
70
60
50
40
30
20
10
0
f1 (ppm)

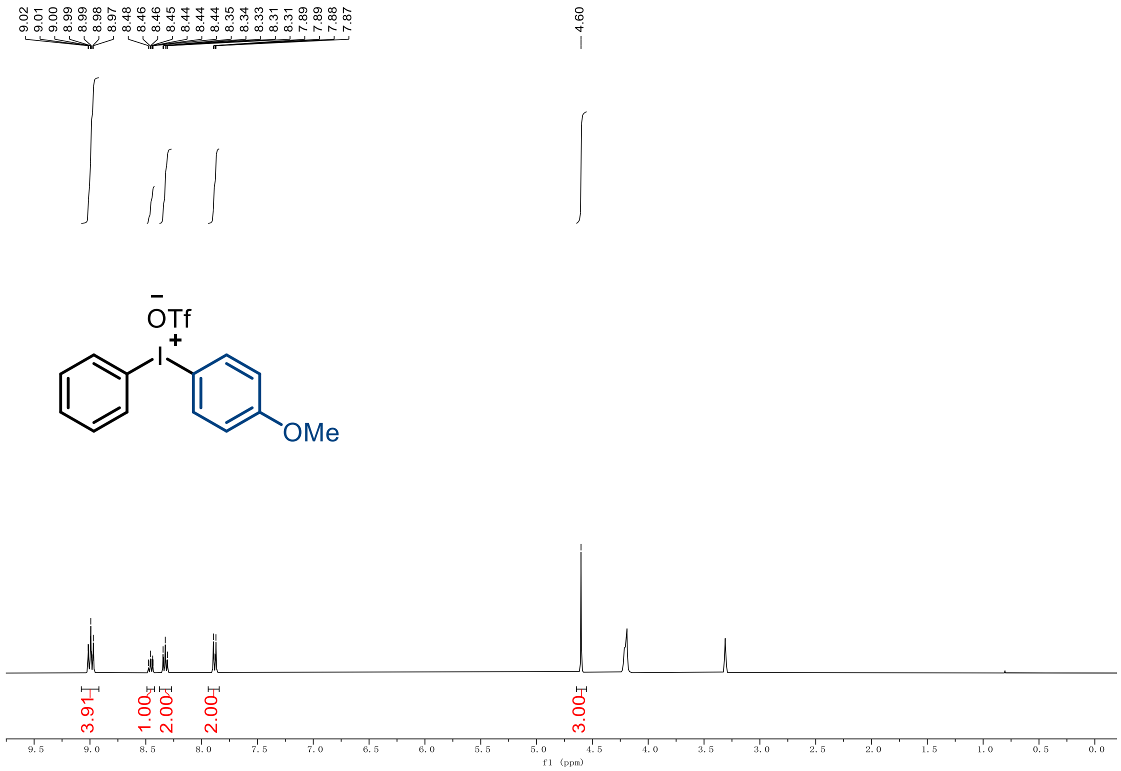
9.02
9.01
9.00
8.99
8.99
8.98
8.97
8.48
8.46
8.46
8.45
8.44
8.44
8.44
8.35
8.34
8.33
8.31
8.31
7.89
7.89
7.88
7.87
4.60
OTf
I
OMe
3.91
1.00
2.00
2.00
3.00
f1 (ppm)

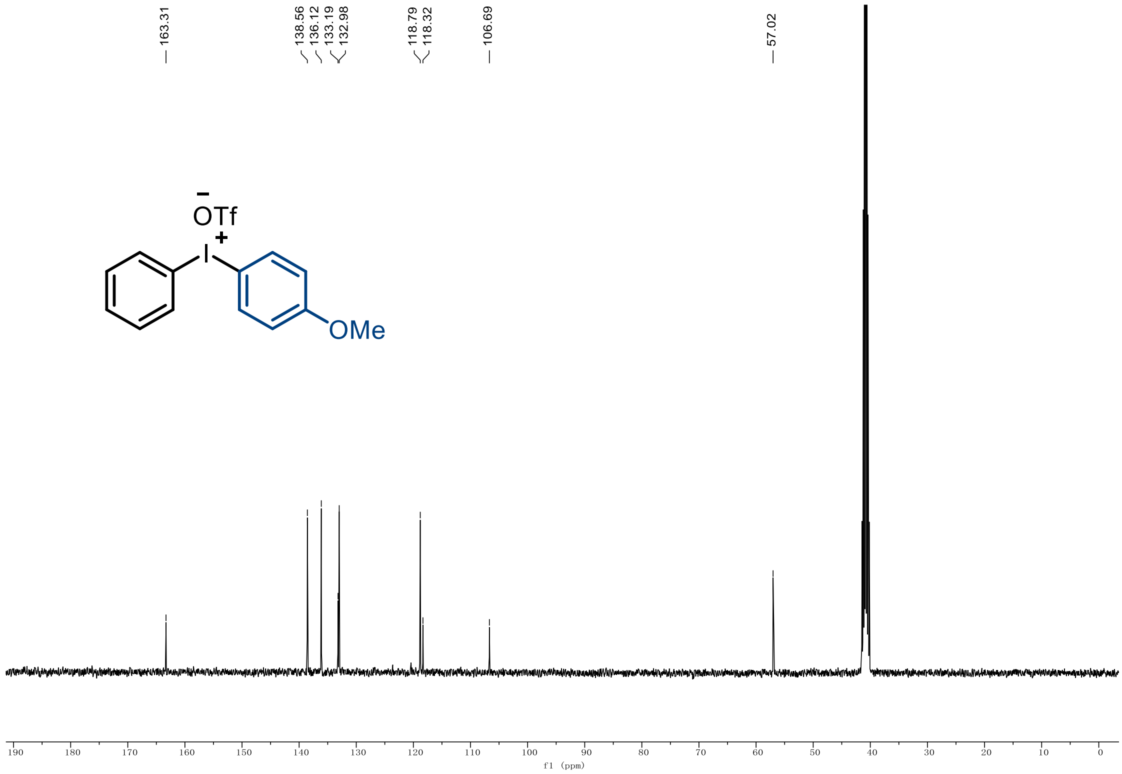
163.31
138.56
136.12
133.19
132.98
118.79
118.32
106.69
57.02
OTf
I
OMe
f1 (ppm)

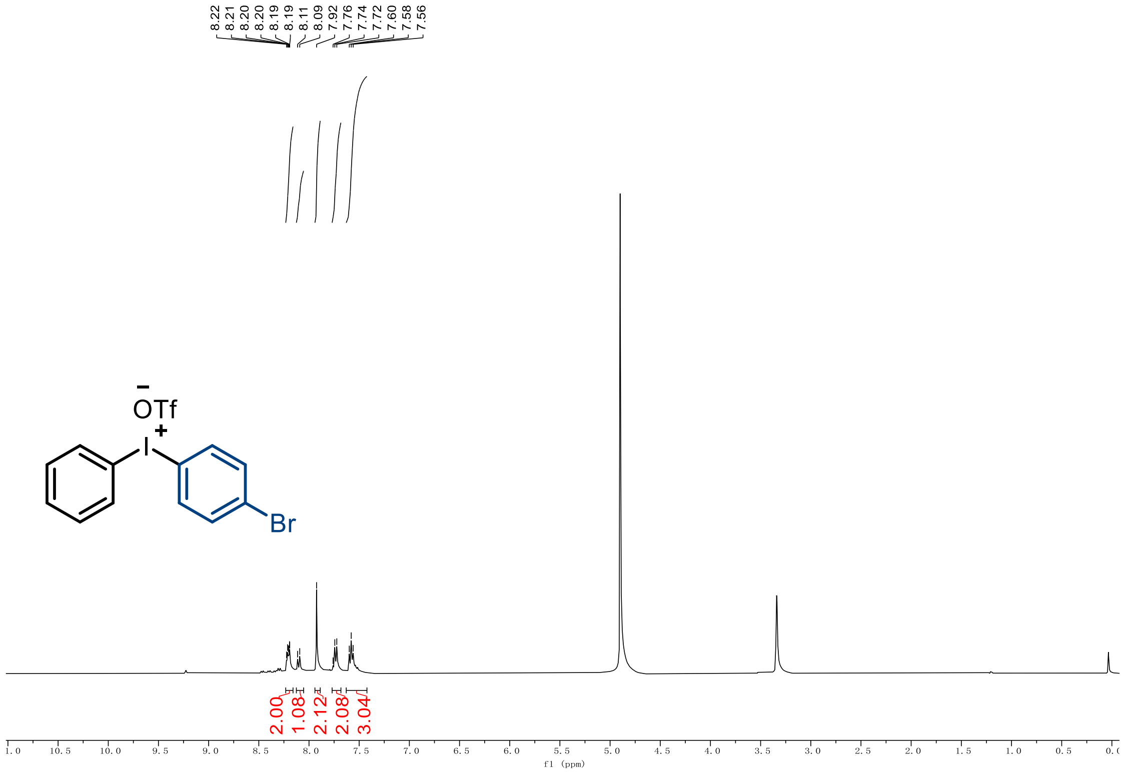
8.22
8.21
8.20
8.20
8.19
8.19
8.11
8.09
7.92
7.76
7.74
7.72
7.60
7.58
7.56
OTf
I
Br
2.00
1.08
2.12
2.08
3.04
f1 (ppm)

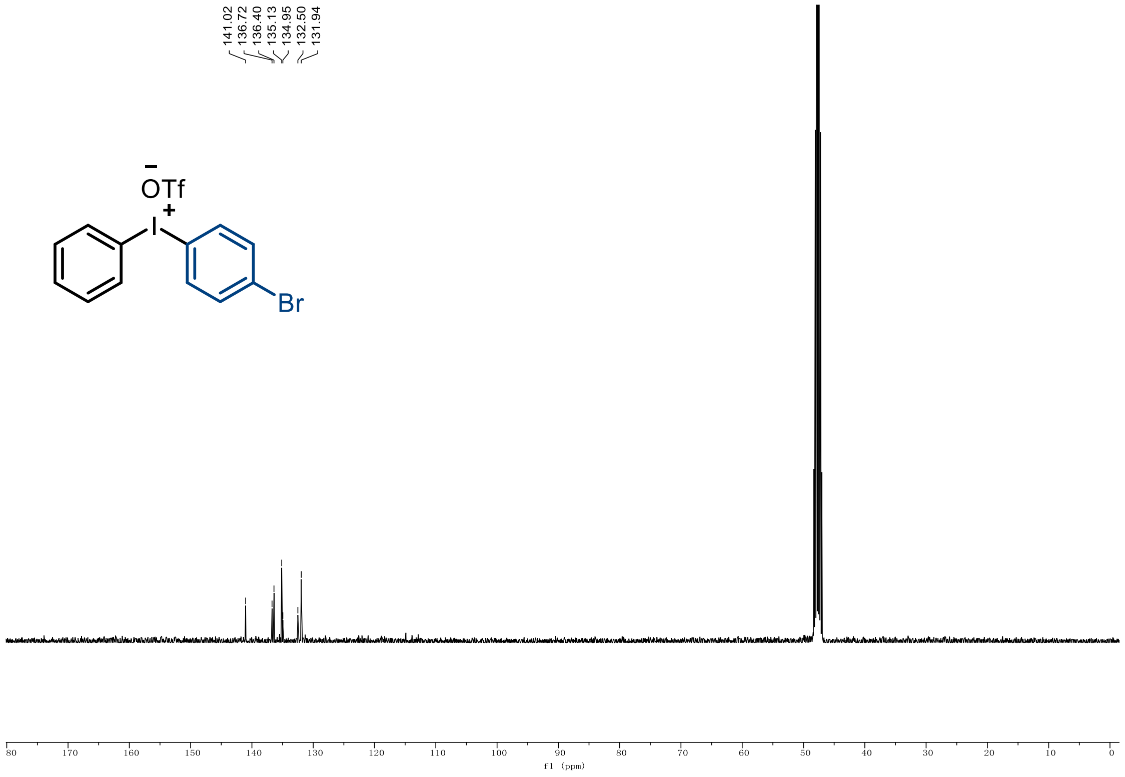
141.02
136.72
136.40
135.13
134.95
132.50
131.94
OTf
I
Br
f1 (ppm)

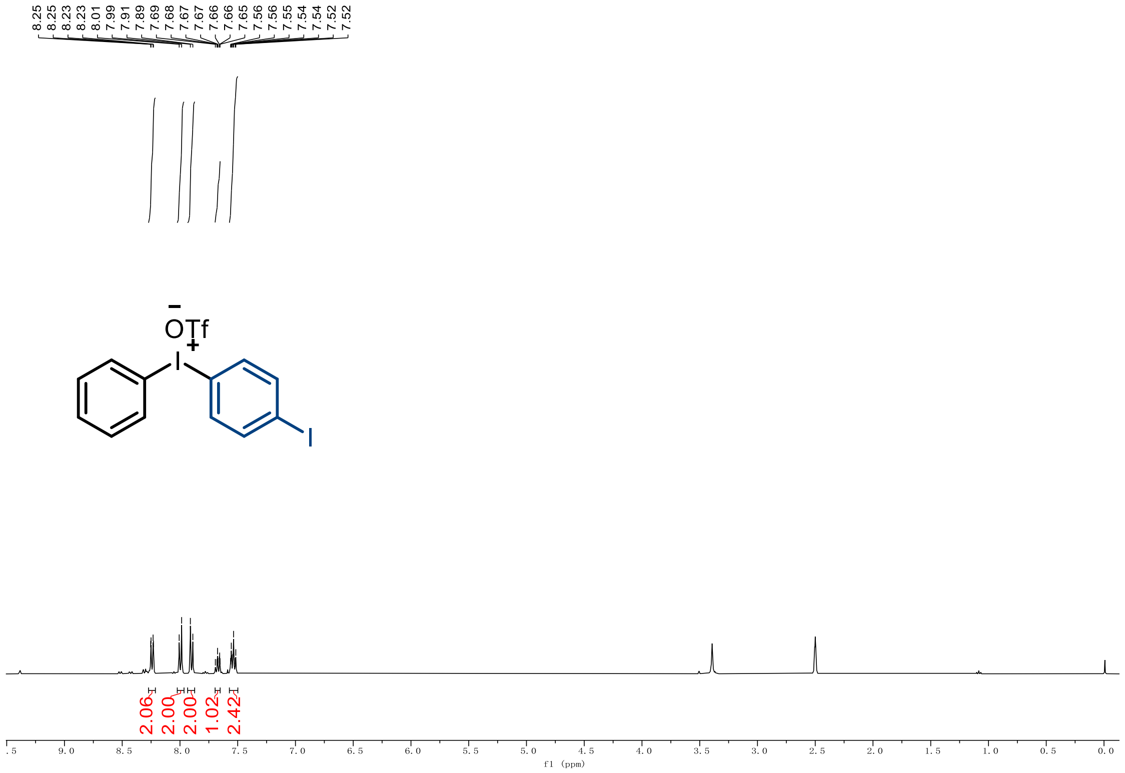
8.25
8.25
8.23
8.23
8.01
7.99
7.91
7.89
7.69
7.68
7.67
7.67
7.66
7.66
7.65
7.56
7.56
7.55
7.54
7.54
7.52
7.52
OTf
I
2.06
2.00
2.00
1.02
2.42
f1 (ppm)

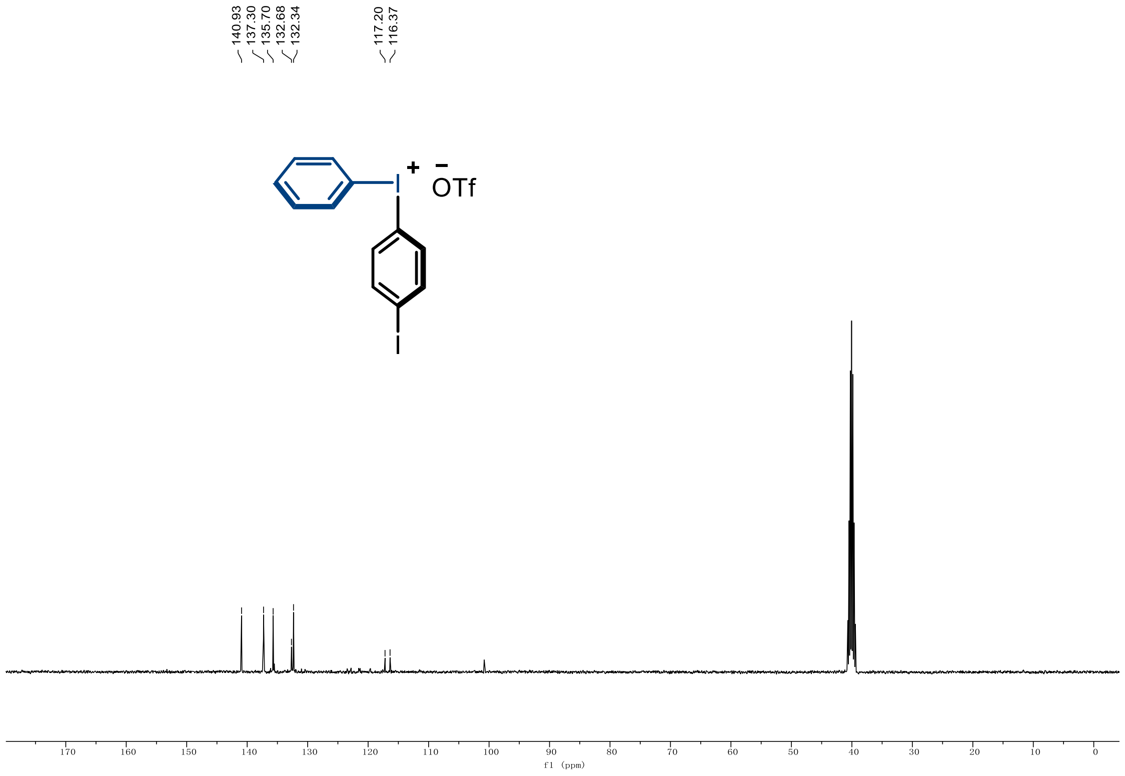
140.93
137.30
135.70
132.68
132.34
117.20
116.37
OTf
I
f1 (ppm)

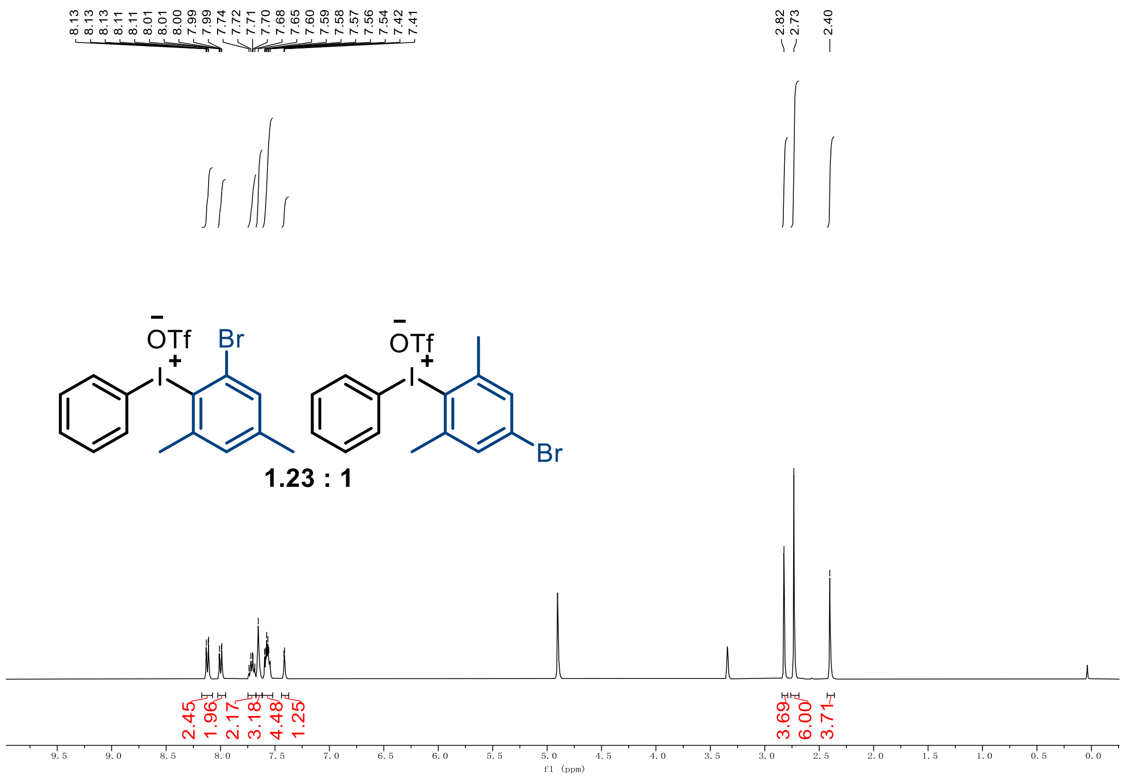

8.13
8.13
8.13
8.11
8.11
8.01
8.01
8.00
7.99
7.99
7.74
7.72
7.71
7.70
7.68
7.65
7.60
7.59
7.58
7.57
7.56
7.54
7.42
7.41
2.82
2.73
2.40
OTf
Br
Br
1.23 : 1
2.45
1.96
2.17
3.18
4.48
1.25
3.69
6.00
3.71
f1 (ppm)

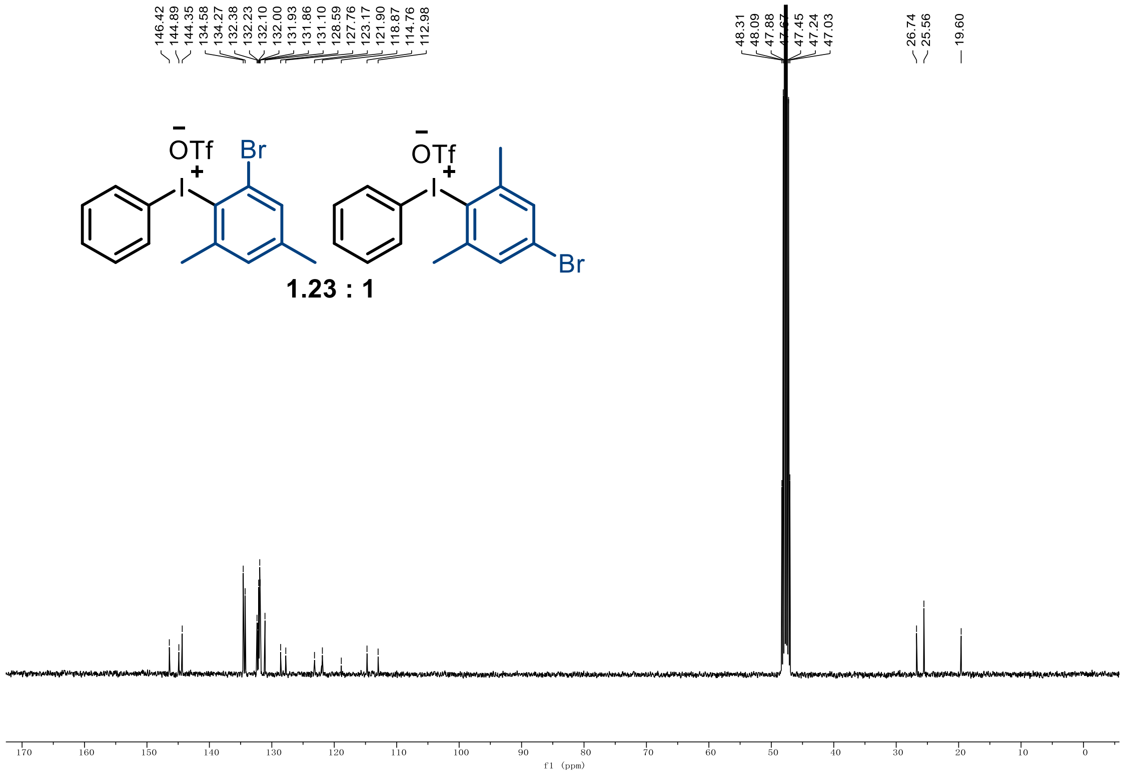

146.42
144.89
144.35
134.58
134.27
132.38
132.23
132.10
132.00
131.93
131.86
131.10
128.59
127.76
123.17
121.90
118.87
114.76
112.98
48.31
48.09
47.88
47.45
47.24
47.03
26.74
25.56
19.60
OTf
Br
Br
1.23 : 1
f1 (ppm)

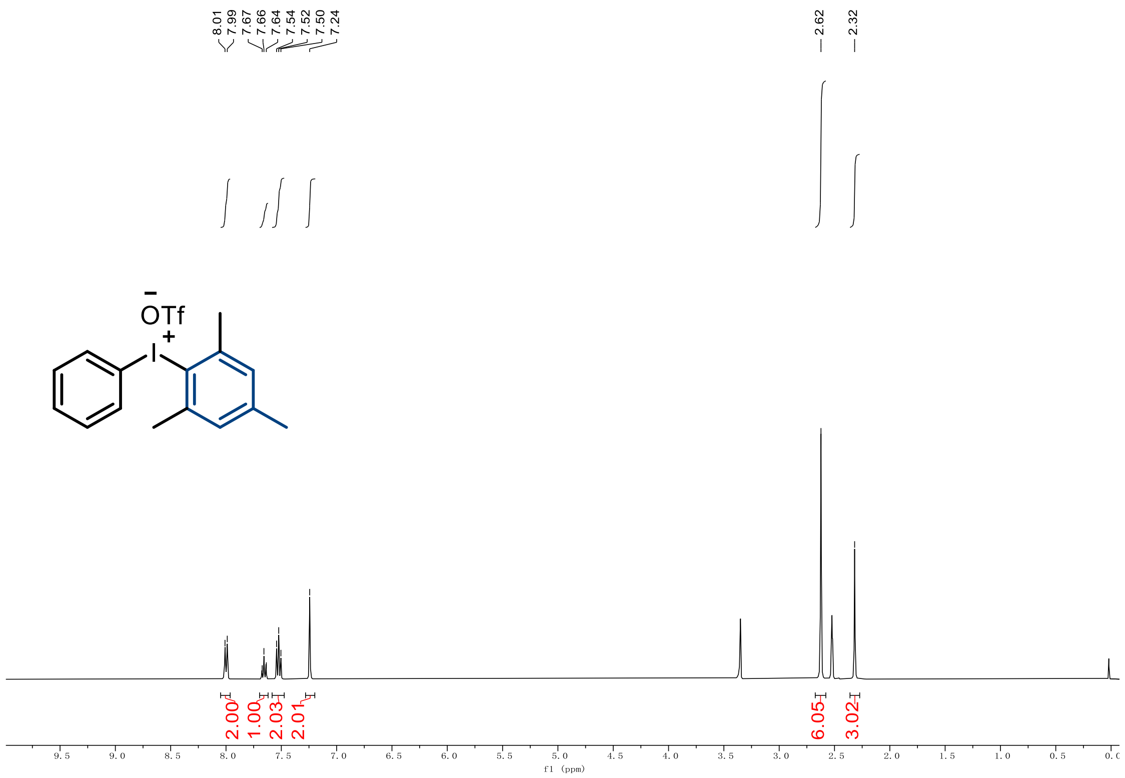
8.01
7.99
7.67
7.66
7.64
7.54
7.52
7.50
7.24
2.62
2.32
OTf
I
2.00
1.00
2.03
2.01
6.05
3.02
f1 (ppm)

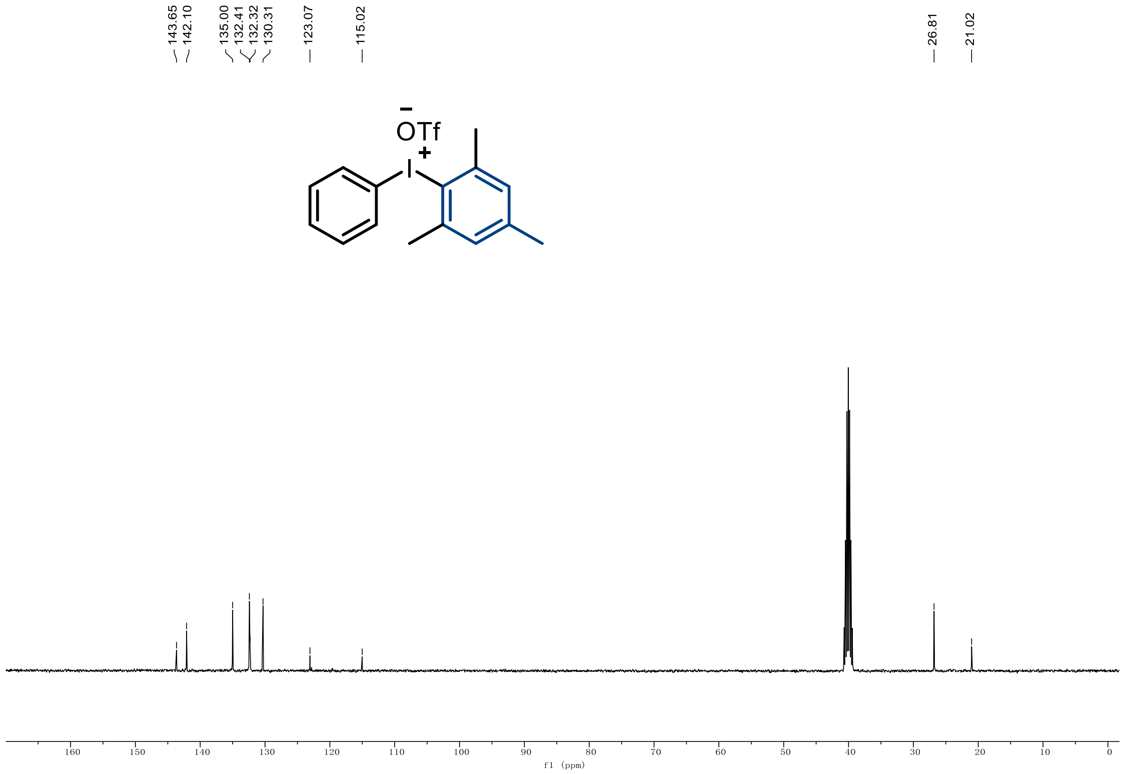
143.65
142.10
135.00
132.41
132.32
130.31
123.07
115.02
26.81
21.02
OTf
I
f1 (ppm)

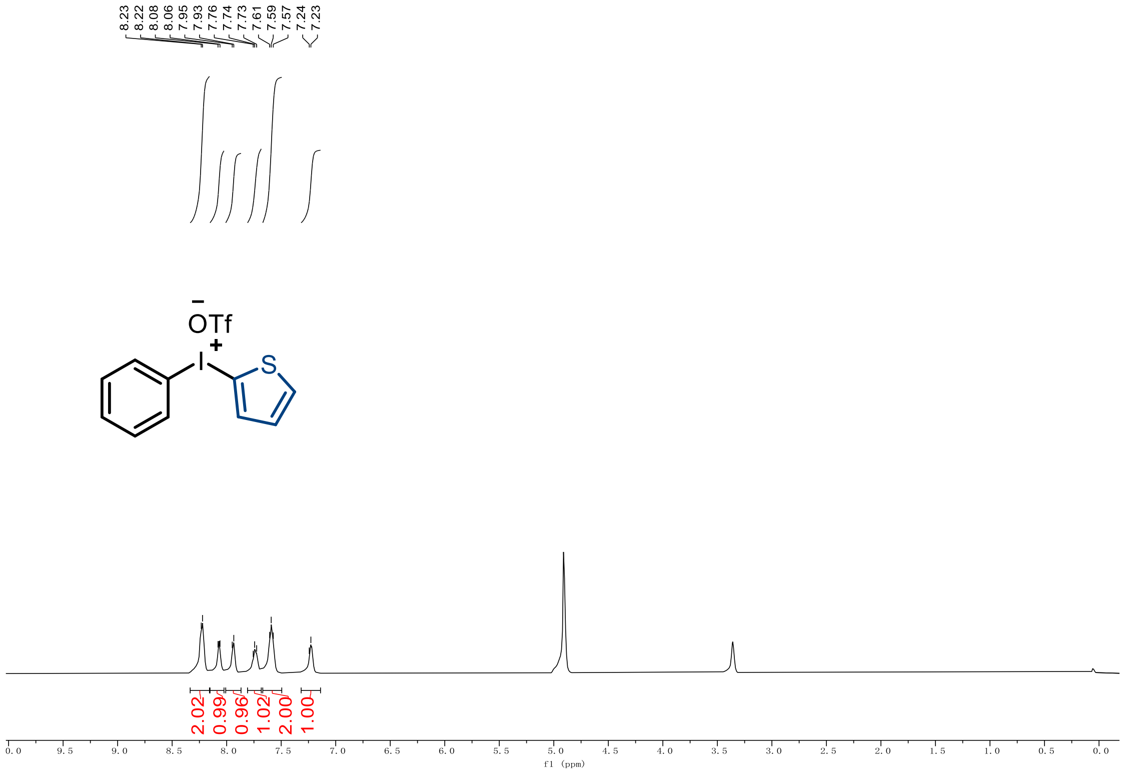
8.23
8.22
8.08
8.06
7.95
7.93
7.76
7.74
7.73
7.61
7.59
7.57
7.24
7.23
OTf
I
S
2.02
0.99
0.96
1.02
2.00
1.00
f1 (ppm)

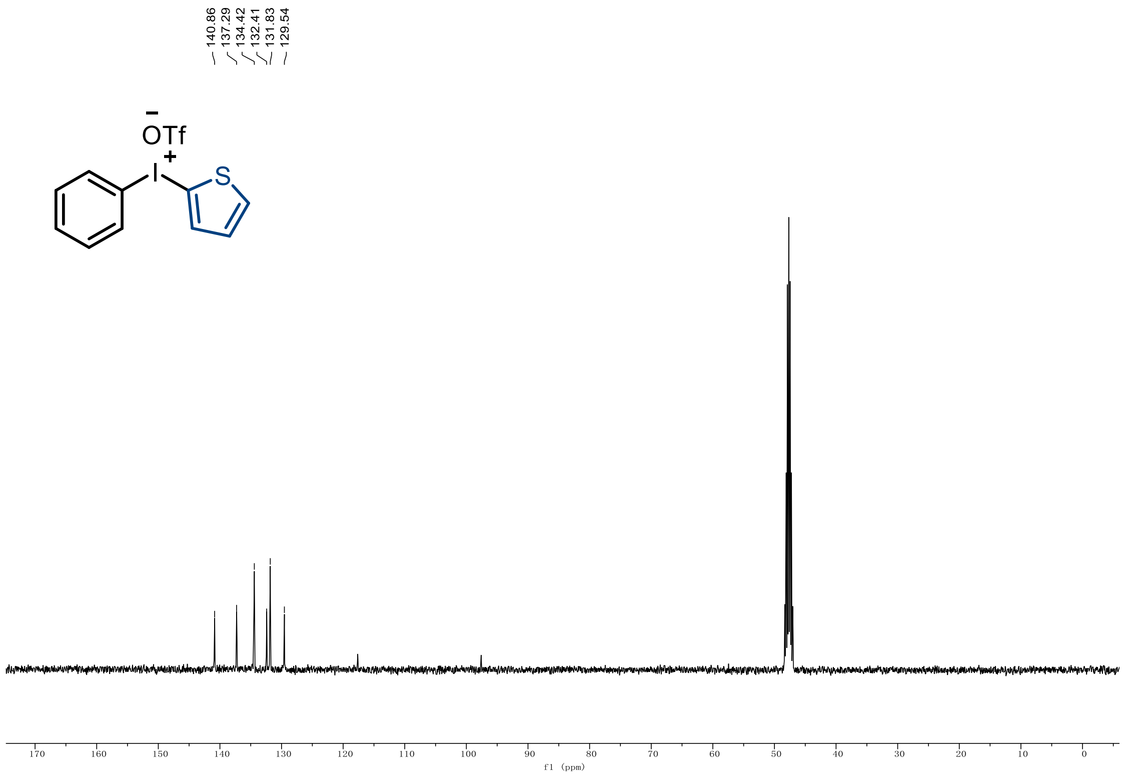
140.86
137.29
134.42
132.41
131.83
129.54
OTf
I
S
f1 (ppm)

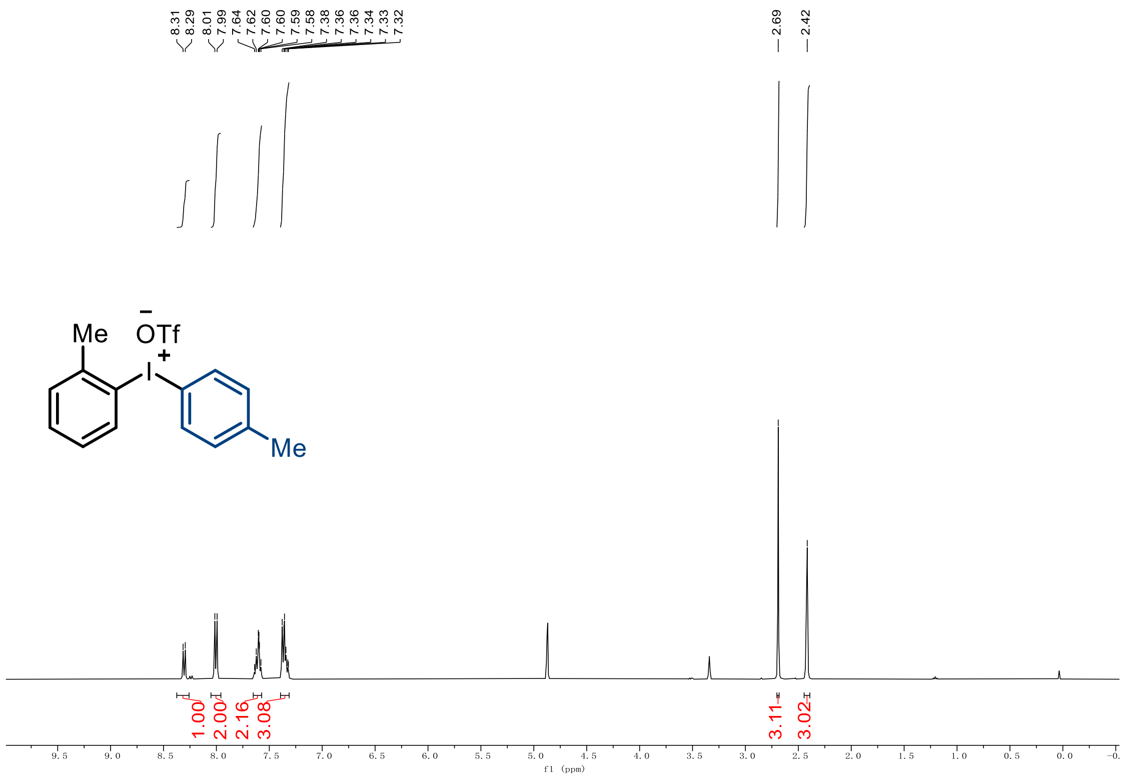

8.31
8.29
8.01
7.99
7.64
7.62
7.60
7.60
7.59
7.58
7.38
7.36
7.36
7.34
7.33
7.32
2.69
2.42
Me
OTf
I
Me
1.00
2.00
2.16
3.08
3.11
3.02
9.5
9.0
8.5
8.0
7.5
7.0
6.5
6.0
5.5
5.0
4.5
4.0
3.5
3.0
2.5
2.0
1.5
1.0
0.5
0.0
f1 (ppm)

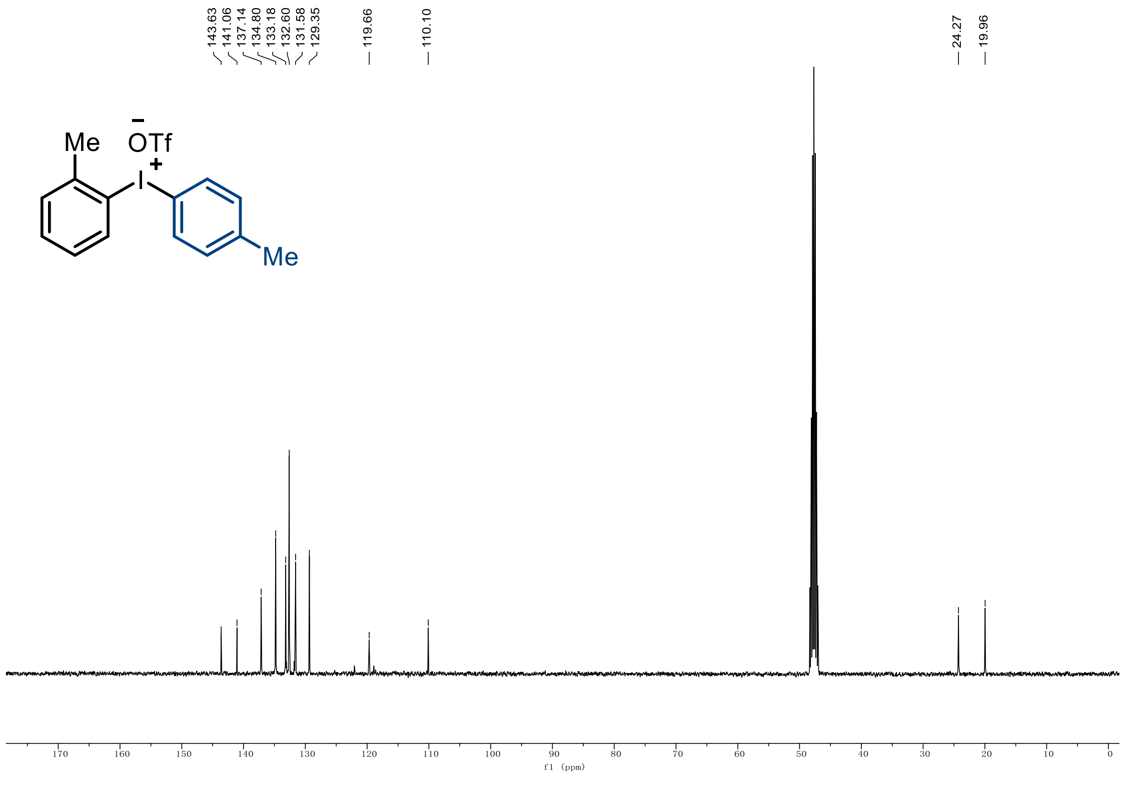

143.63
141.06
137.14
134.80
133.18
132.60
131.58
129.35
119.66
110.10
24.27
19.96
Me
OTf
I
Me
170
160
150
140
130
120
110
100
90
80
70
60
50
40
30
20
10
0
f1 (ppm)

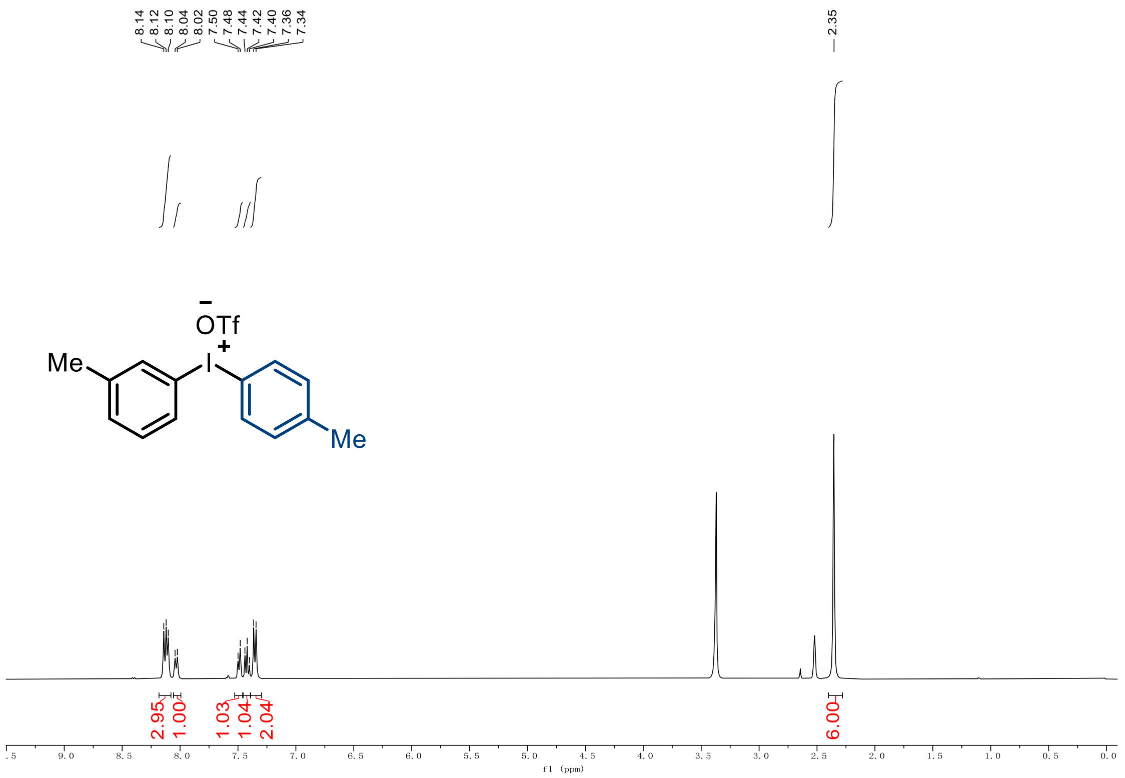

8.14
8.12
8.10
8.04
8.02
7.50
7.48
7.44
7.42
7.40
7.36
7.34
2.35
OTf
Me
I
Me
2.95
1.00
1.03
1.04
2.04
6.00
f1 (ppm)

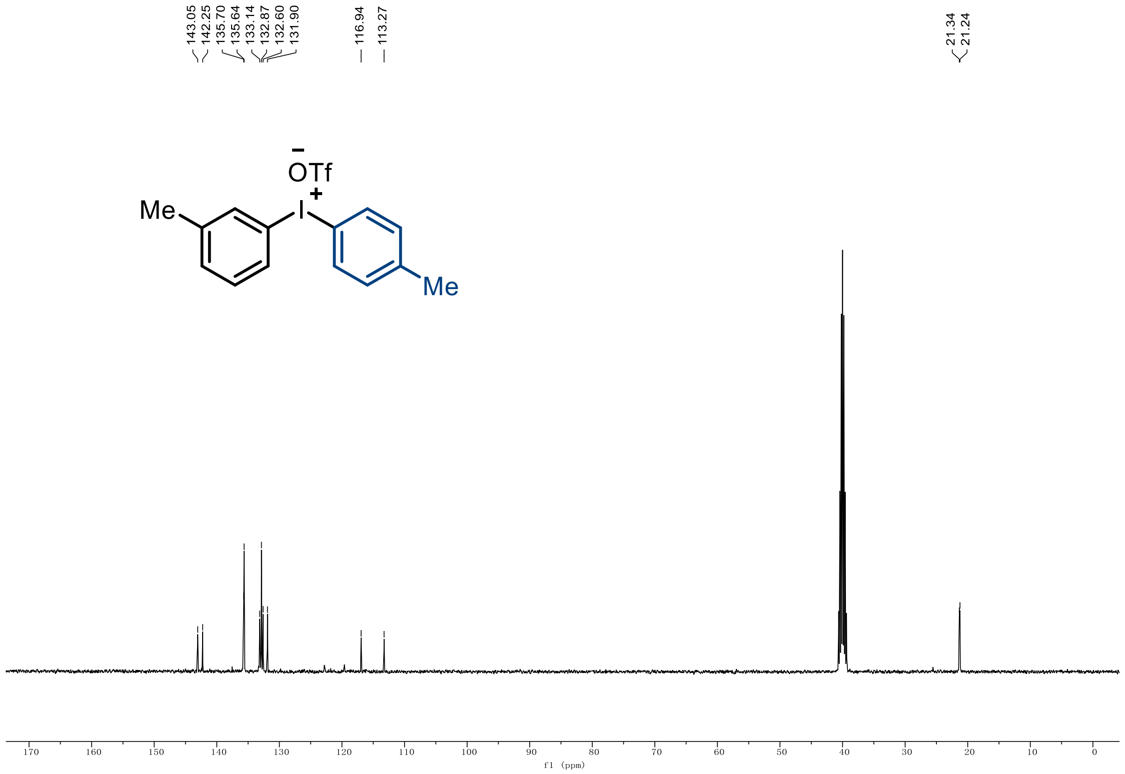

143.05
142.25
135.70
135.64
133.14
132.87
132.60
131.90
116.94
113.27
21.34
21.24
OTf
Me
I
Me
f1 (ppm)

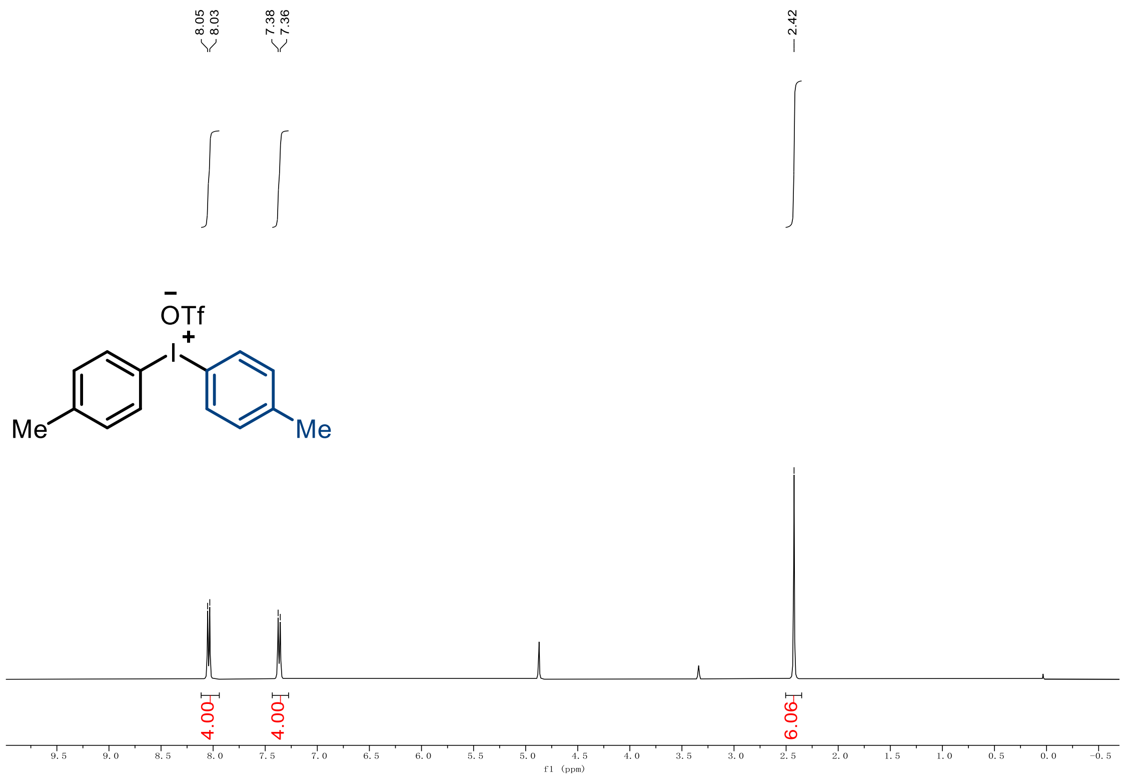
8.05
8.03
7.38
7.36
2.42
OTf
I
Me
Me
4.00
4.00
6.06
f1 (ppm)

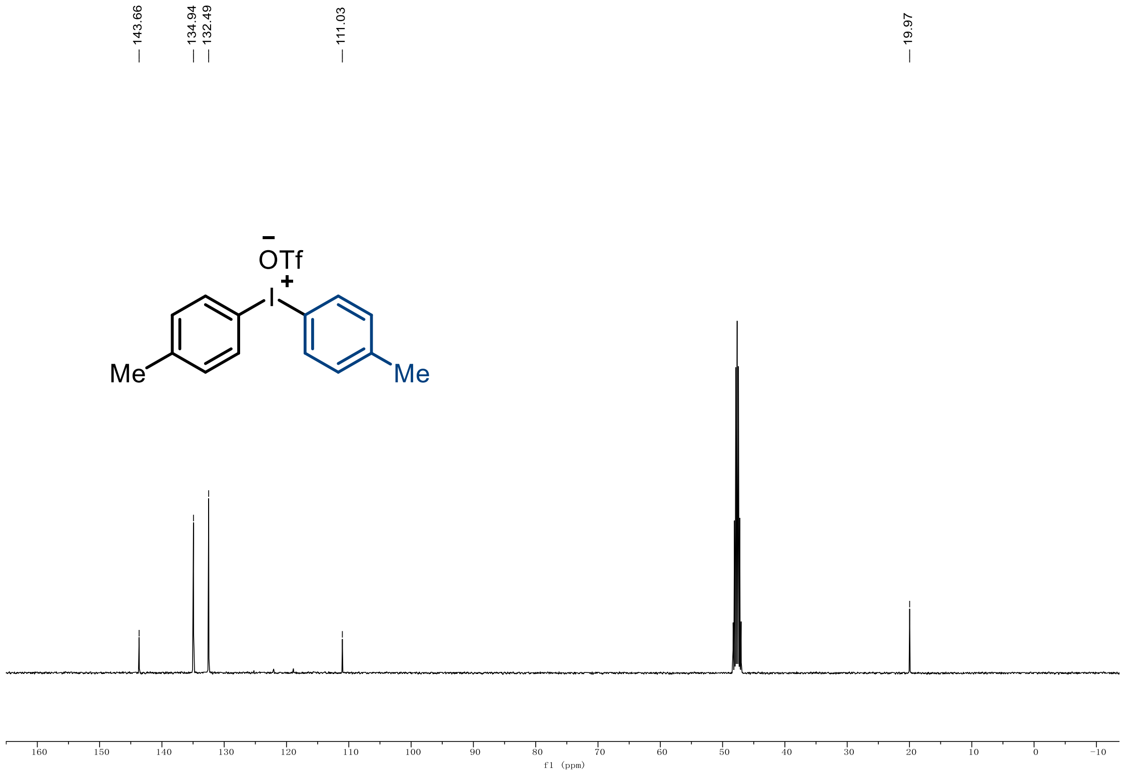
143.66
134.94
132.49
111.03
19.97
OTf
I
Me
Me
f1 (ppm)

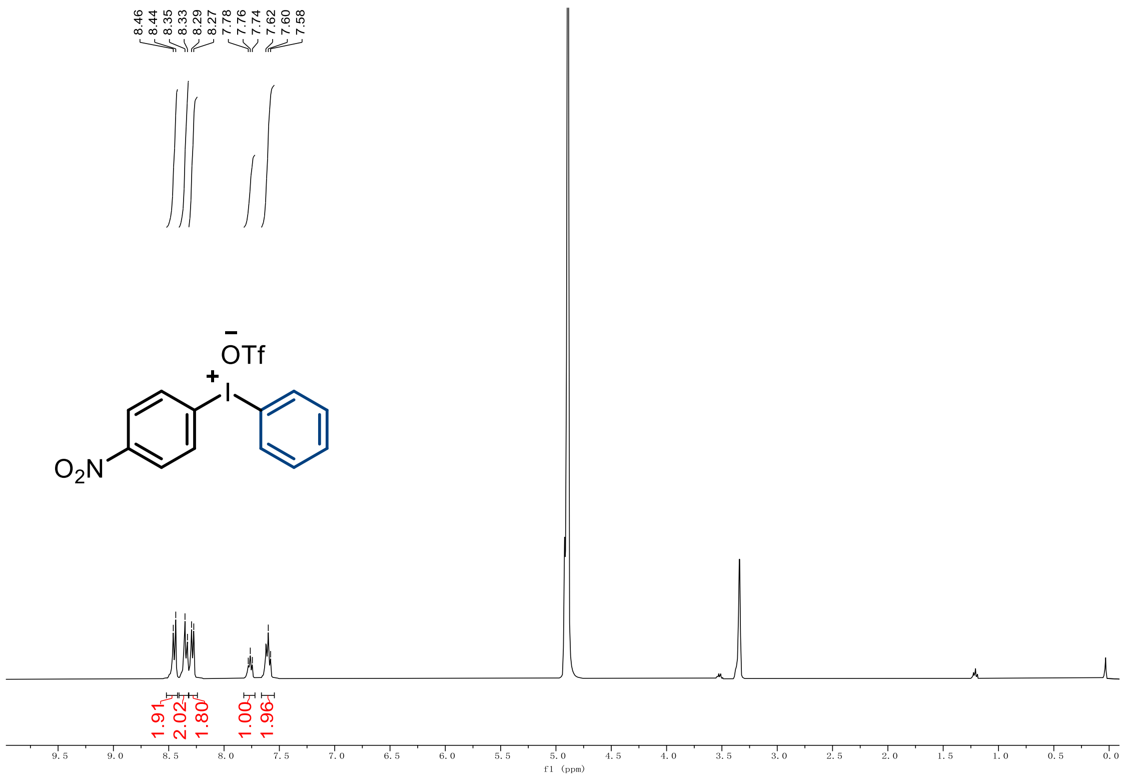
8.46
8.44
8.35
8.33
8.29
8.27
7.78
7.76
7.74
7.62
7.60
7.58
OTf
I
O_2N
1.91
2.02
1.80
1.00
1.96
9.5
9.0
8.5
8.0
7.5
7.0
6.5
6.0
5.5
5.0
4.5
4.0
3.5
3.0
2.5
2.0
1.5
1.0
0.5
0.0
f1 (ppm)

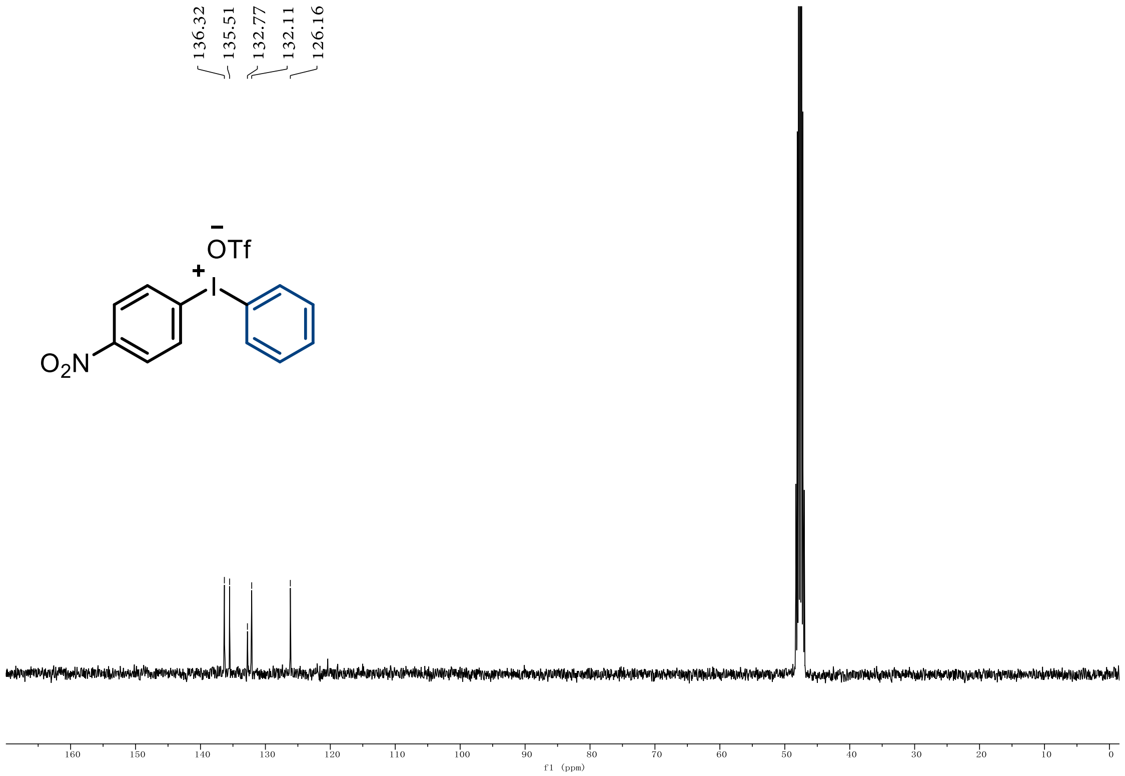
136.32
135.51
132.77
132.11
126.16
OTf
I
O_2N
160
150
140
130
120
110
100
90
80
70
60
50
40
30
20
10
0
f1 (ppm)

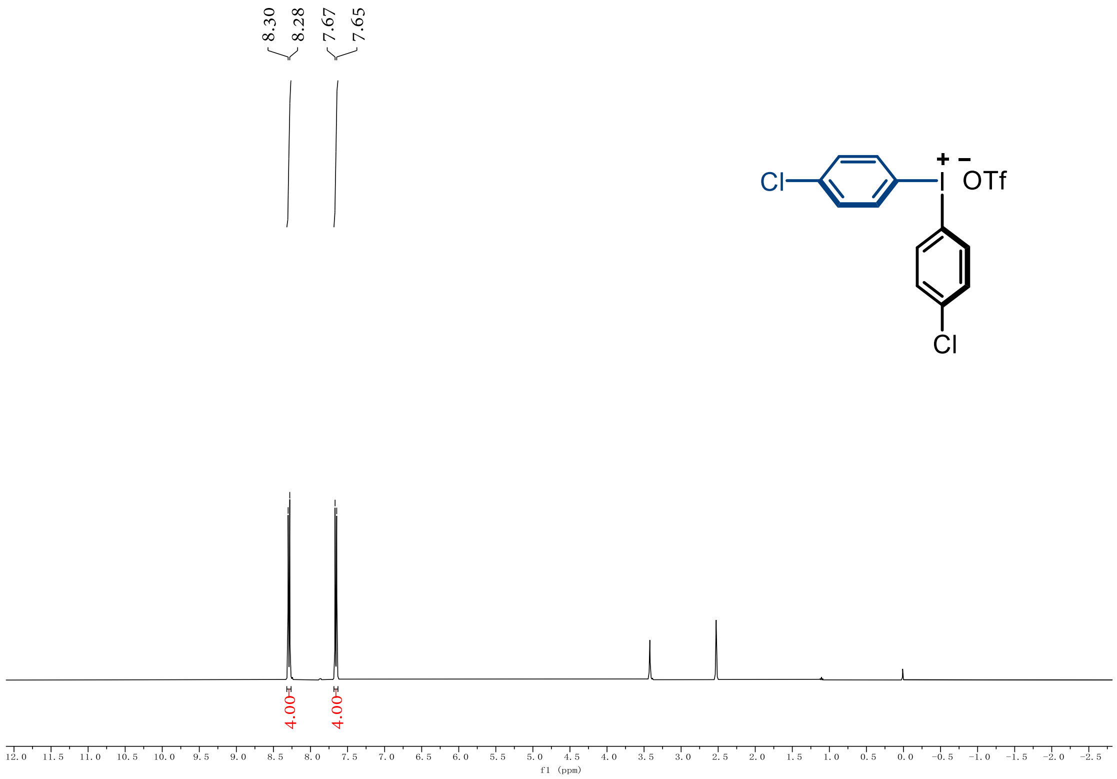
8.30
8.28
7.67
7.65
Cl
I
OTf
Cl
4.00
4.00
f1 (ppm)

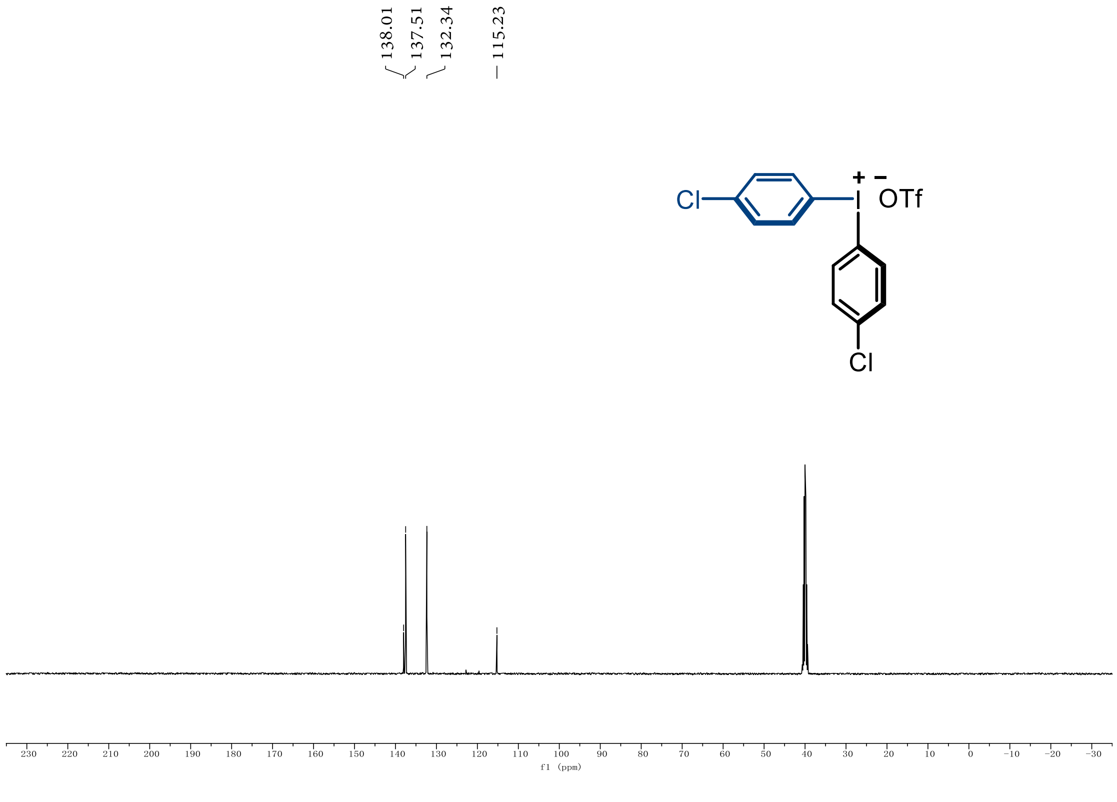
138.01
137.51
132.34
115.23
Cl
I
OTf
Cl
f1 (ppm)

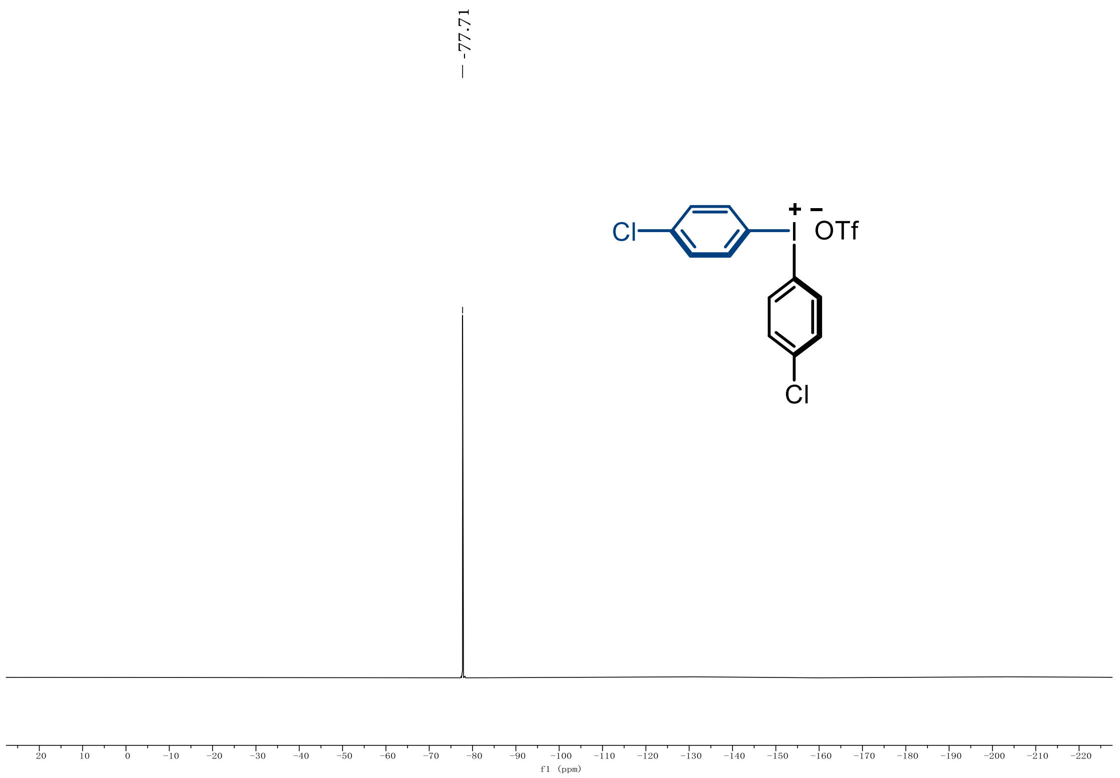
-77.71
Cl
I
OTf
Cl
f1 (ppm)

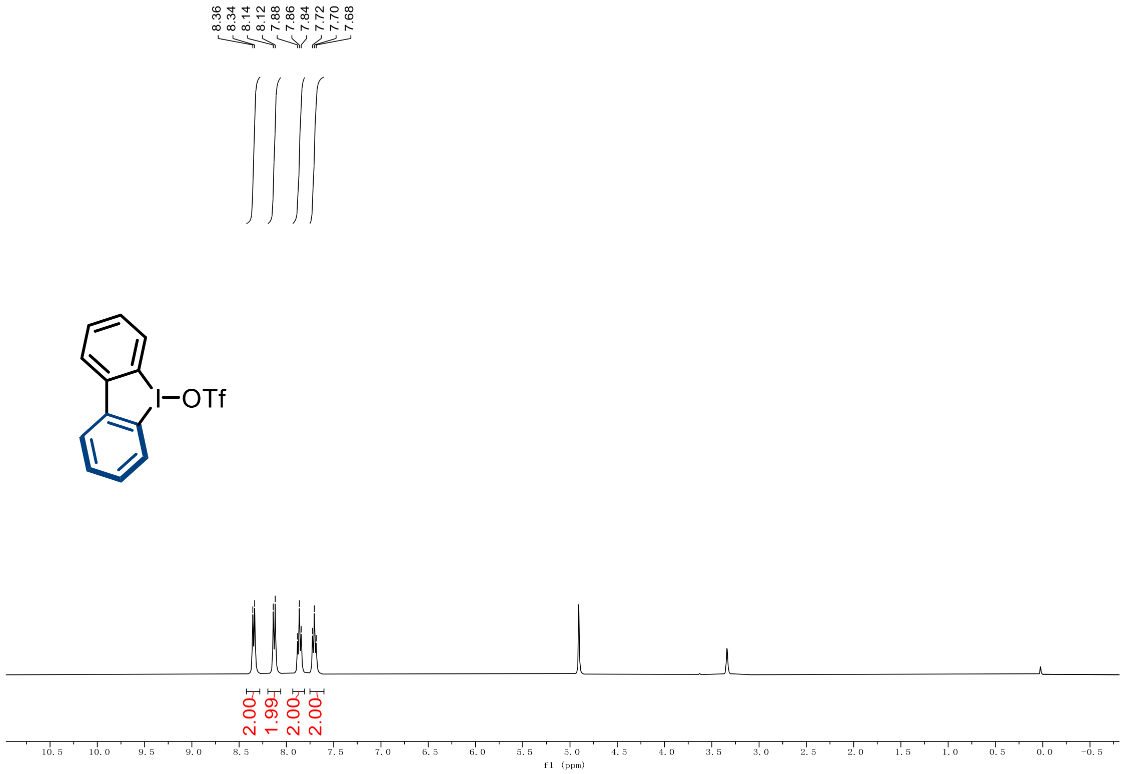
8.36
8.34
8.14
8.12
7.88
7.86
7.84
7.72
7.70
7.68
I–OTf
2.00
1.99
2.00
2.00
f1 (ppm)

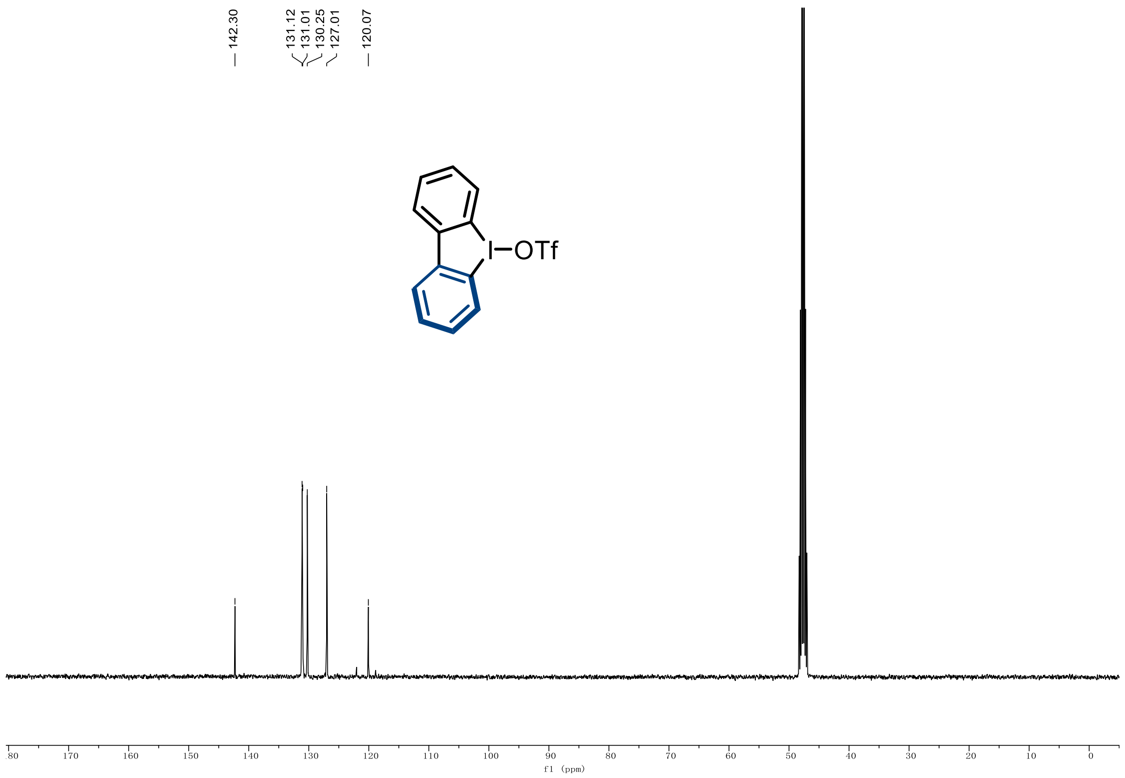
142.30
131.12
131.01
130.25
127.01
120.07
I–OTf
f1 (ppm)

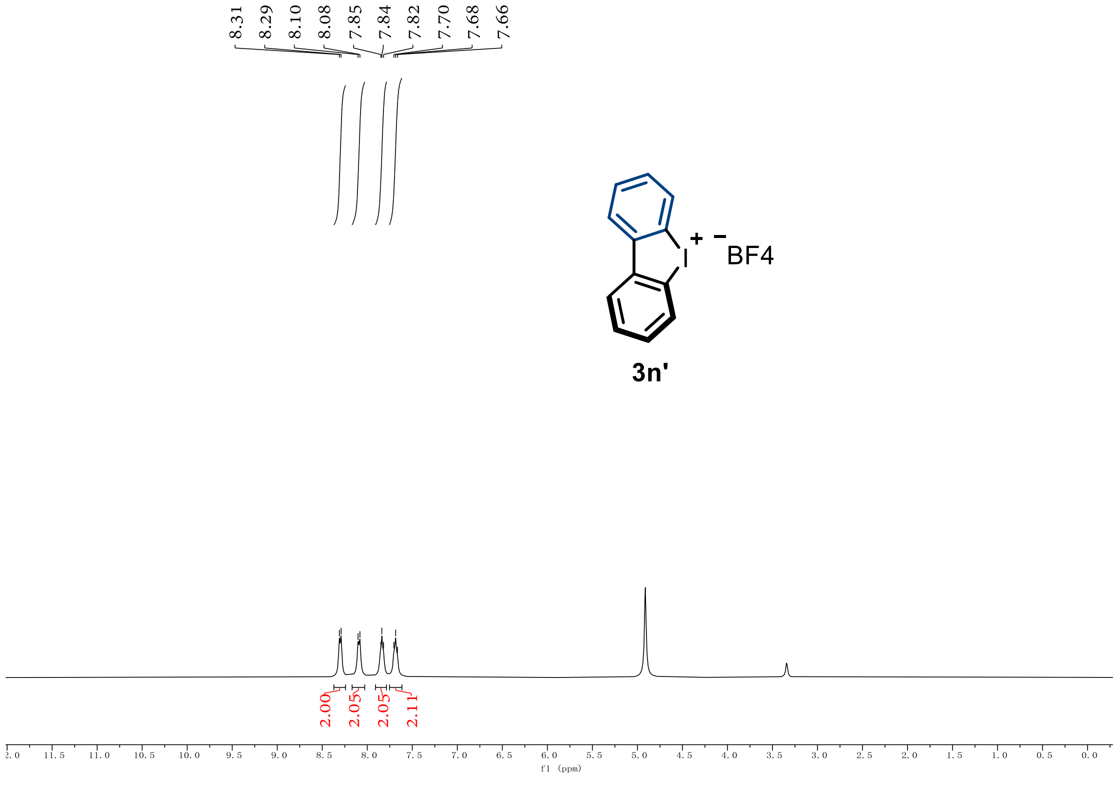
8.31
8.29
8.10
8.08
7.85
7.84
7.82
7.70
7.68
7.66
BF4
2.00
2.05
2.05
2.11
f1 (ppm)

3n'

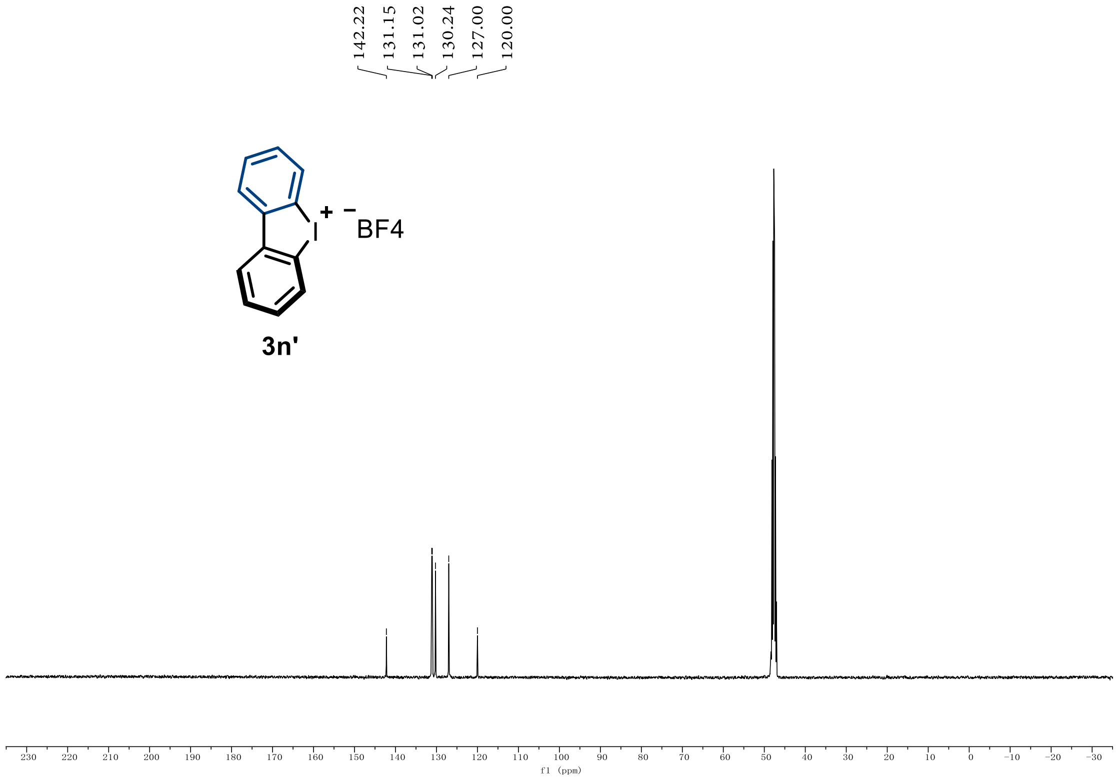
142.22
131.15
131.02
130.24
127.00
120.00
BF4
3n'
230 220 210 200 190 180 170 160 150 140 130 120 110 100 90 80 70 60 50 40 30 20 10 0 -10 -20 -30
f1 (ppm)

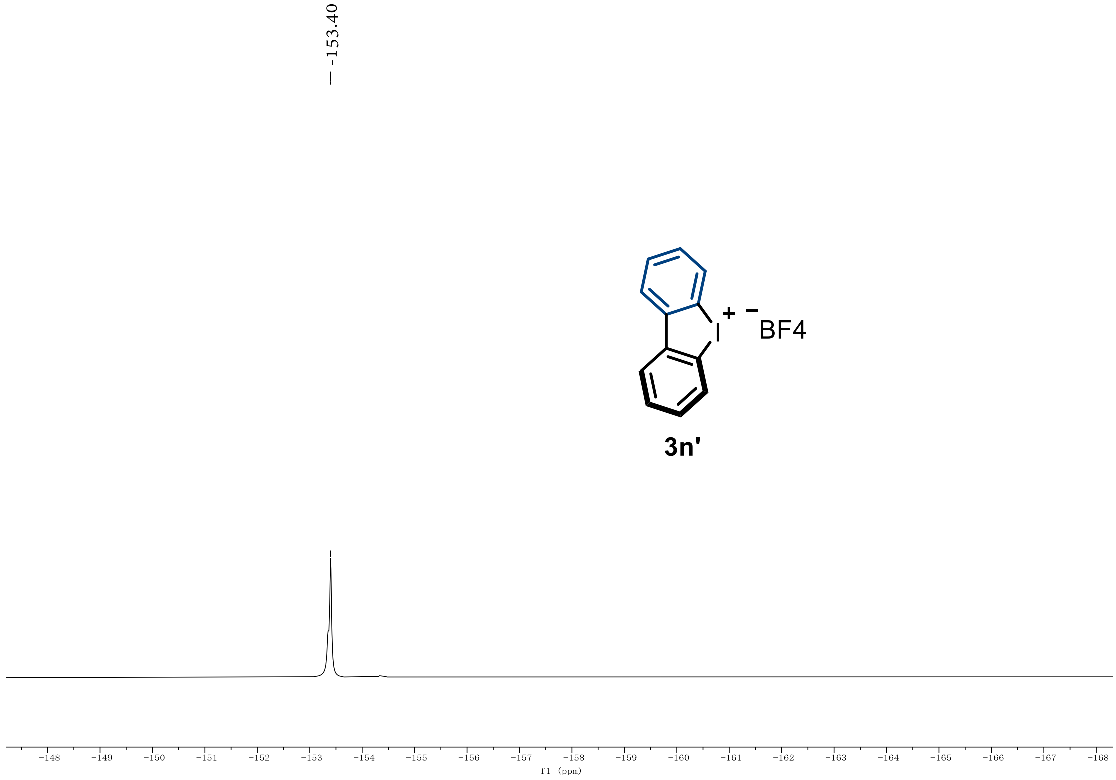
-153.40
BF4
3n'
-148 -149 -150 -151 -152 -153 -154 -155 -156 -157 -158 -159 -160 -161 -162 -163 -164 -165 -166 -167 -168
f1 (ppm)

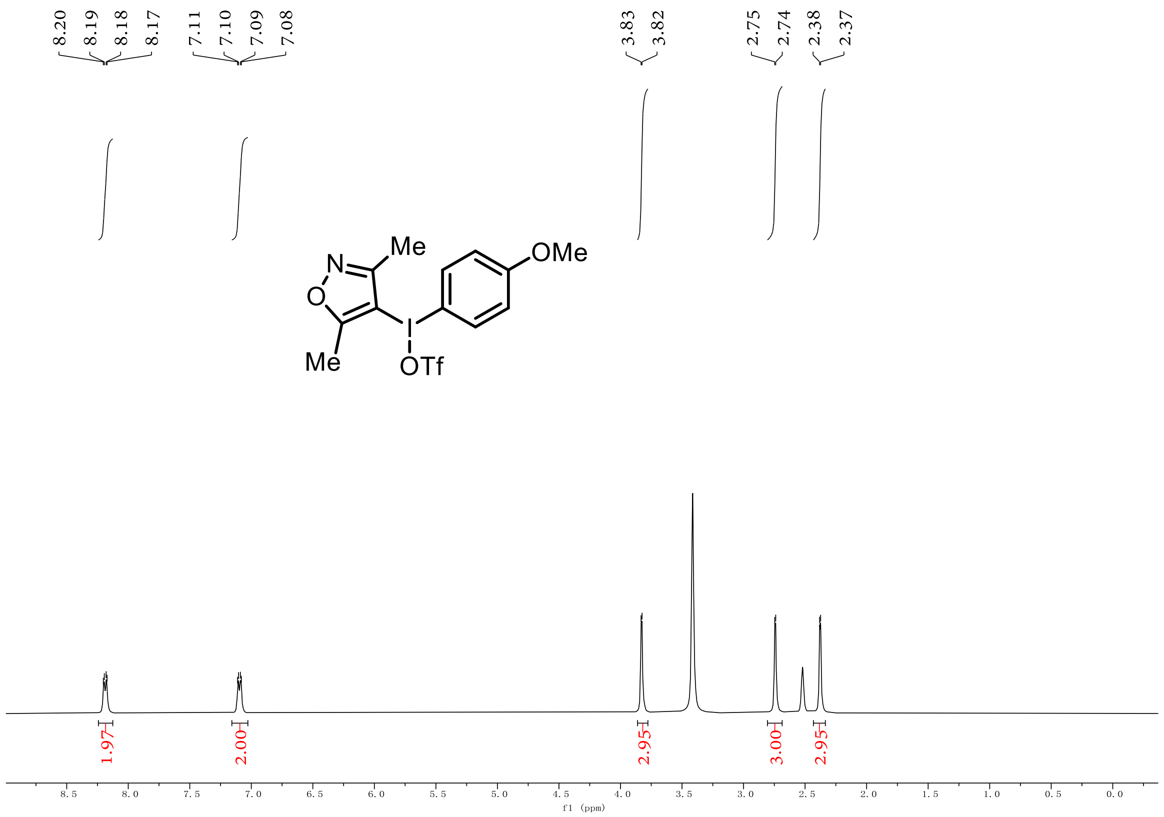
8.20
8.19
8.18
8.17
7.11
7.10
7.09
7.08
3.83
3.82
2.75
2.74
2.38
2.37
Me
OMe
N
O
I
Me
OTf
1.97
2.00
2.95
3.00
2.95
f1 (ppm)

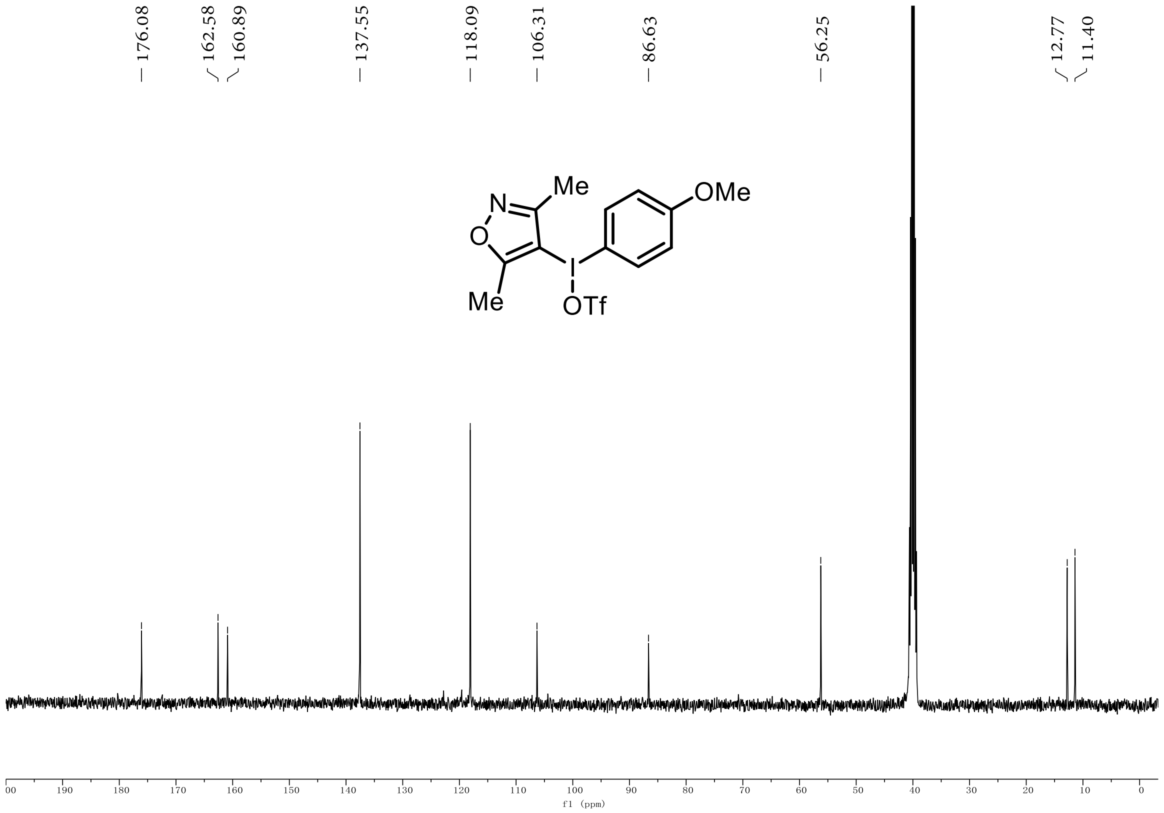
176.08
162.58
160.89
137.55
118.09
106.31
86.63
56.25
12.77
11.40
Me
OMe
N
O
I
Me
OTf
f1 (ppm)

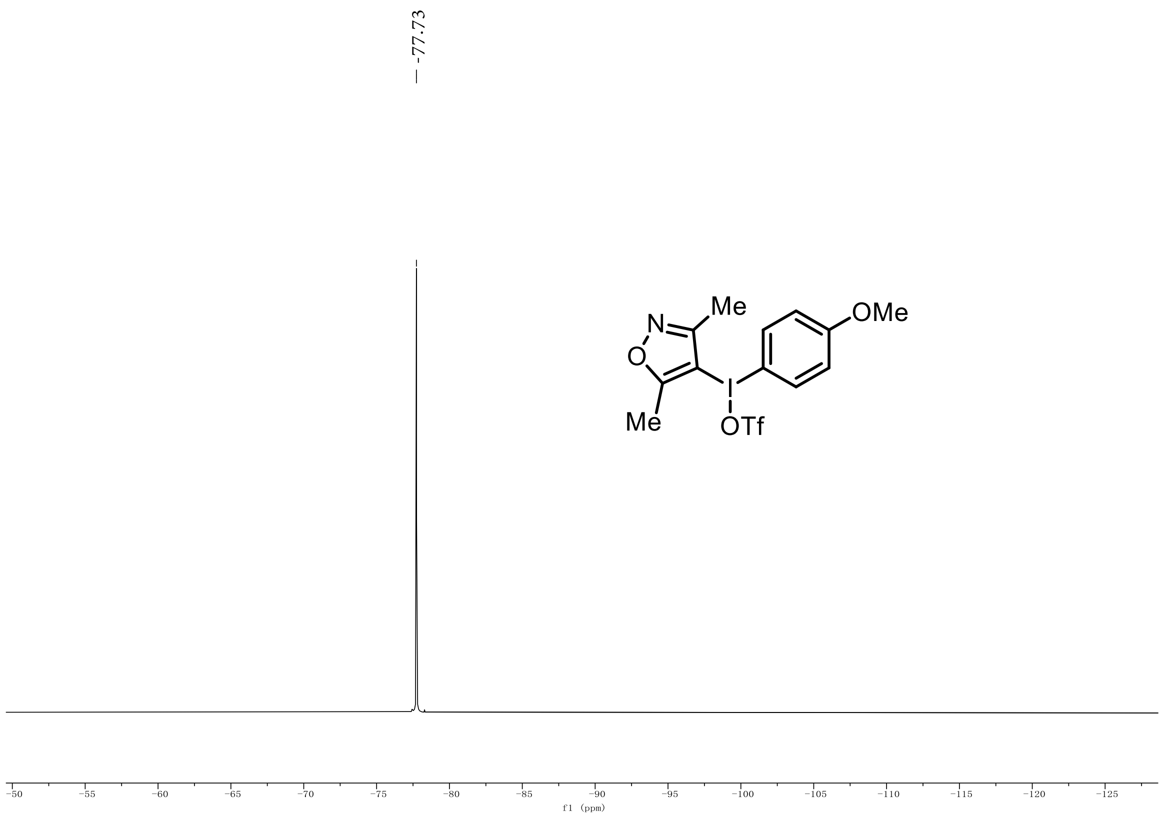
-77.73
Me
N
O
Me
I
OTf
OMe
f1 (ppm)

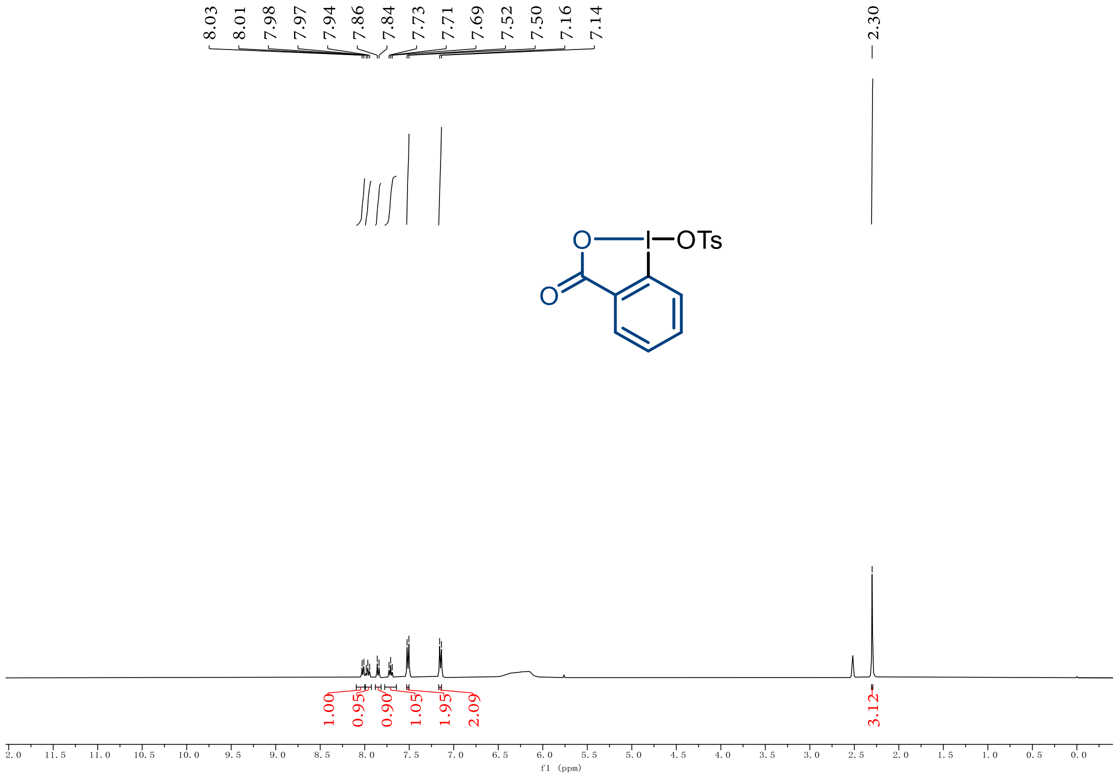
8.03
8.01
7.98
7.97
7.94
7.86
7.84
7.73
7.71
7.69
7.52
7.50
7.16
7.14
2.30
O
I
OTs
O
1.00
0.95
0.90
1.05
1.95
2.09
3.12
f1 (ppm)

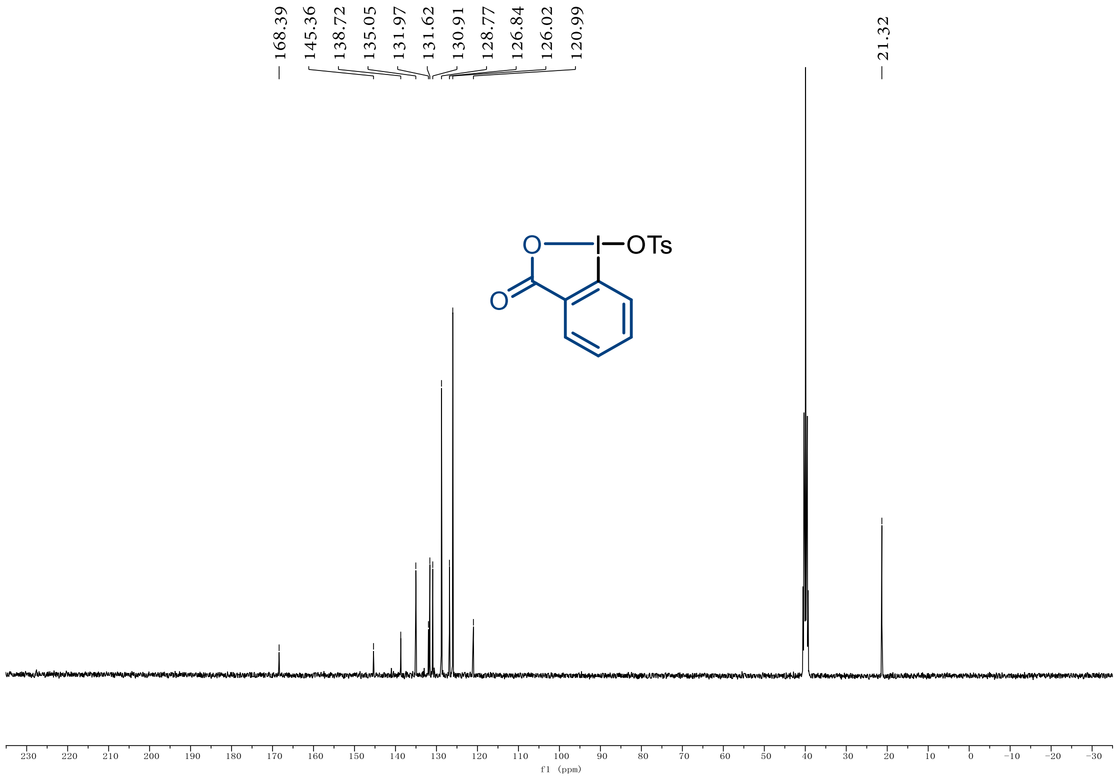
168.39
145.36
138.72
135.05
131.97
131.62
130.91
128.77
126.84
126.02
120.99
21.32
OTs
f1 (ppm)

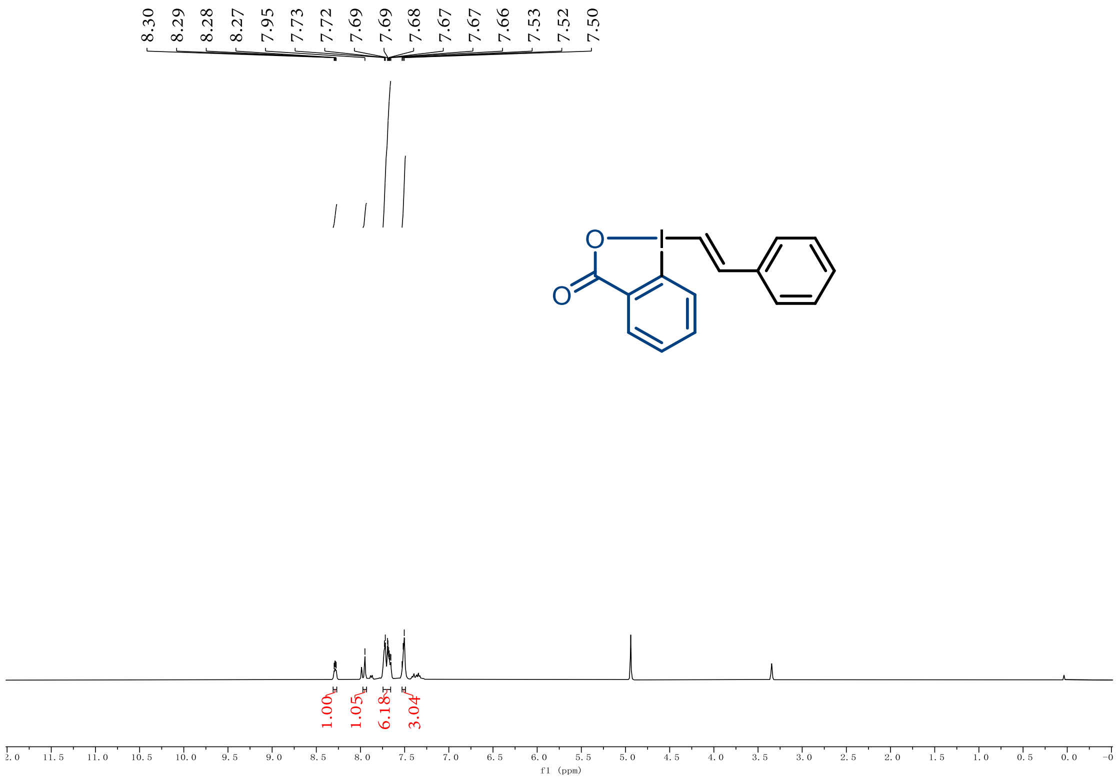
8.30
8.29
8.28
8.27
7.95
7.73
7.72
7.69
7.69
7.68
7.67
7.67
7.66
7.53
7.52
7.50
1.00
1.05
6.18
3.04
f1 (ppm)

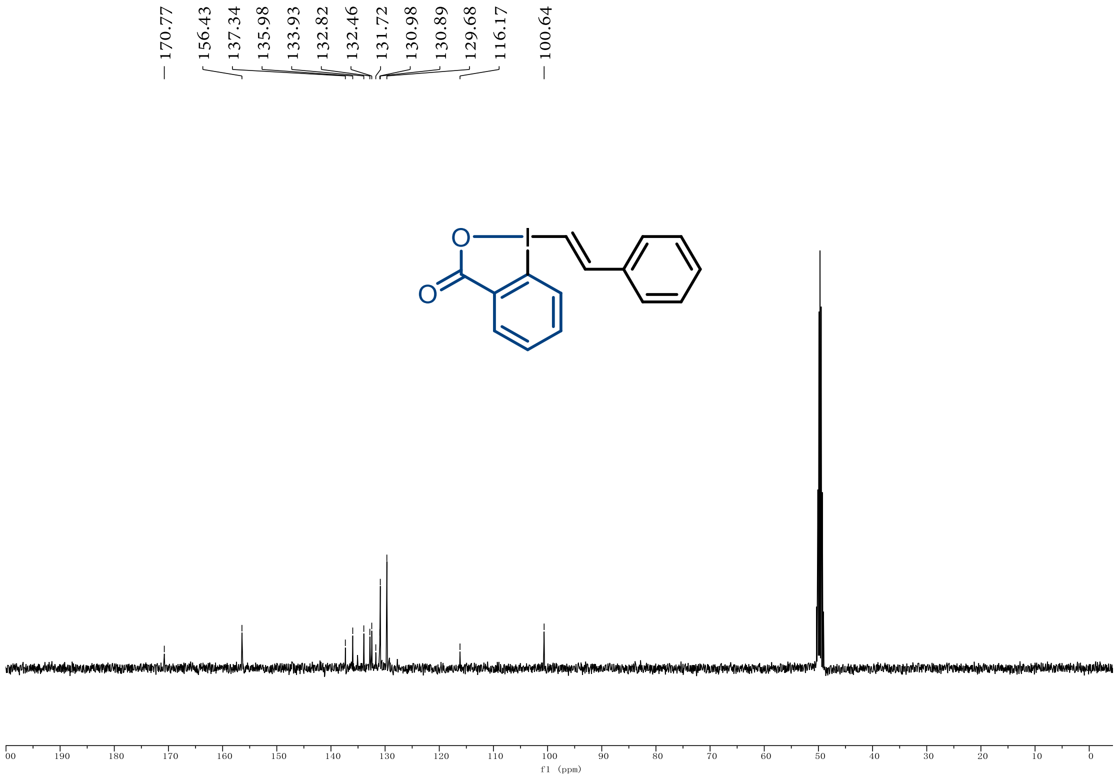
170.77
156.43
137.34
135.98
133.93
132.82
132.46
131.72
130.98
130.89
129.68
116.17
100.64
O
I
O
f1 (ppm)

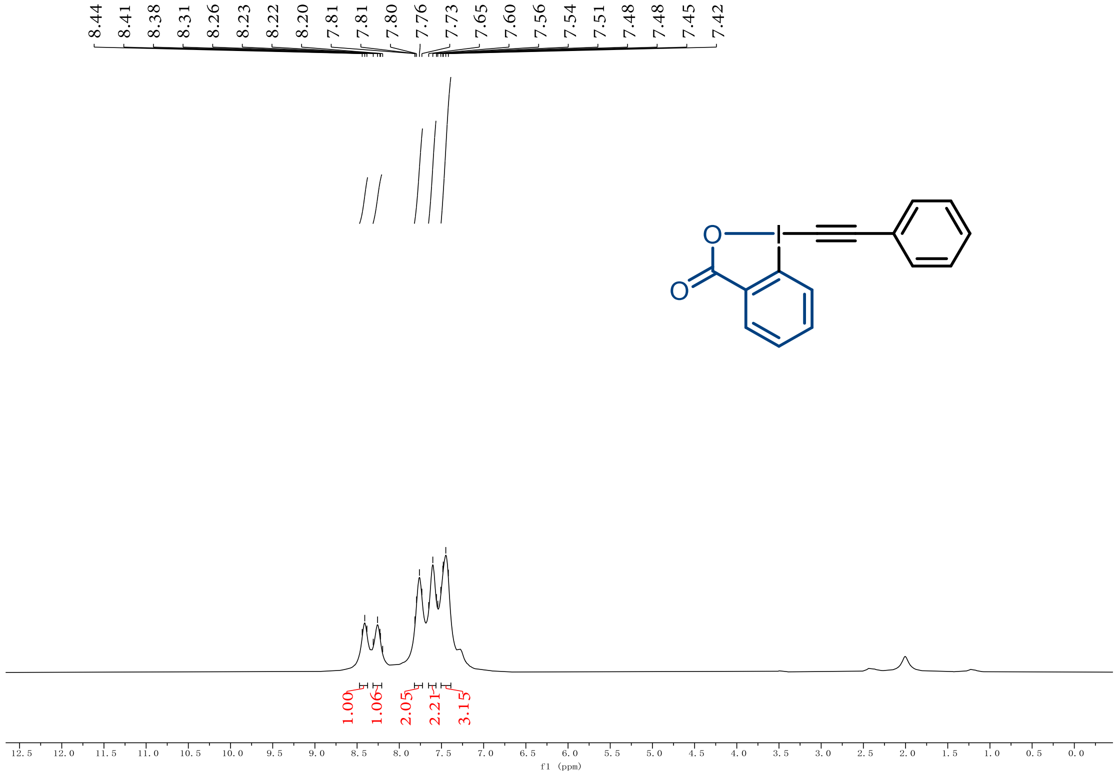
8.44
8.41
8.38
8.31
8.26
8.23
8.22
8.20
7.81
7.81
7.80
7.76
7.73
7.65
7.60
7.56
7.54
7.51
7.48
7.48
7.45
7.42
O
I
O
1.00
1.06
2.05
2.21
3.15
f1 (ppm)

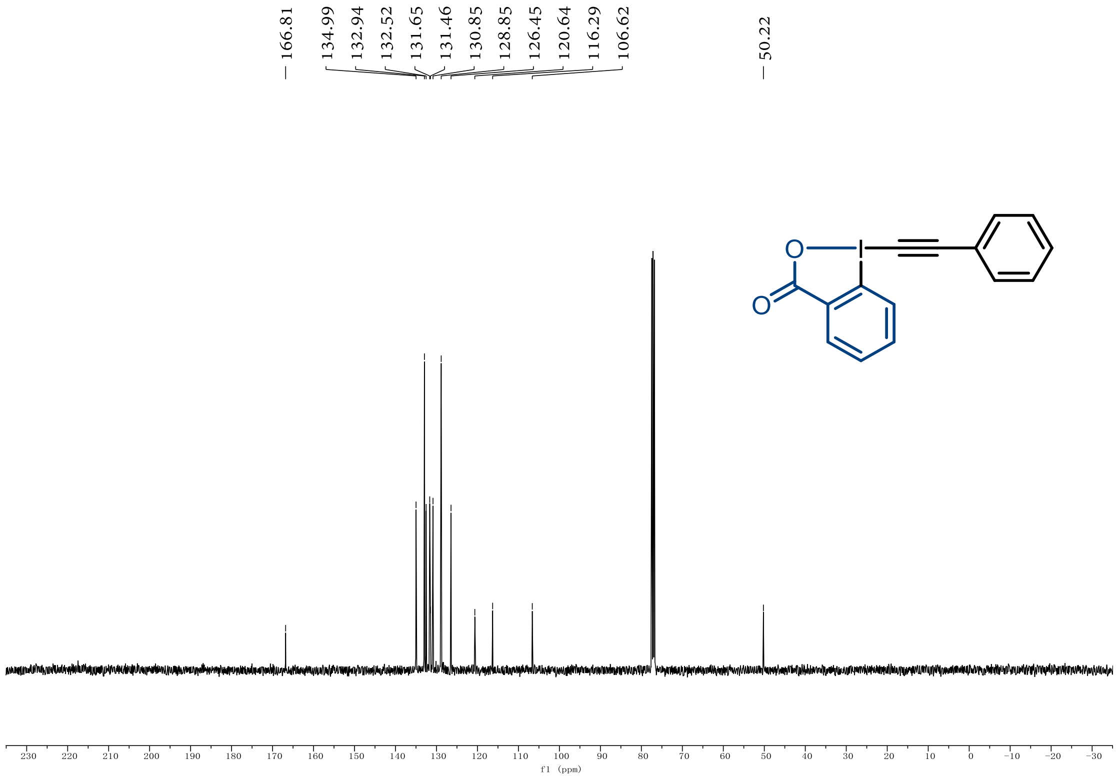

166.81
134.99
132.94
132.52
131.65
131.46
130.85
128.85
126.45
120.64
116.29
106.62
50.22
f1 (ppm)

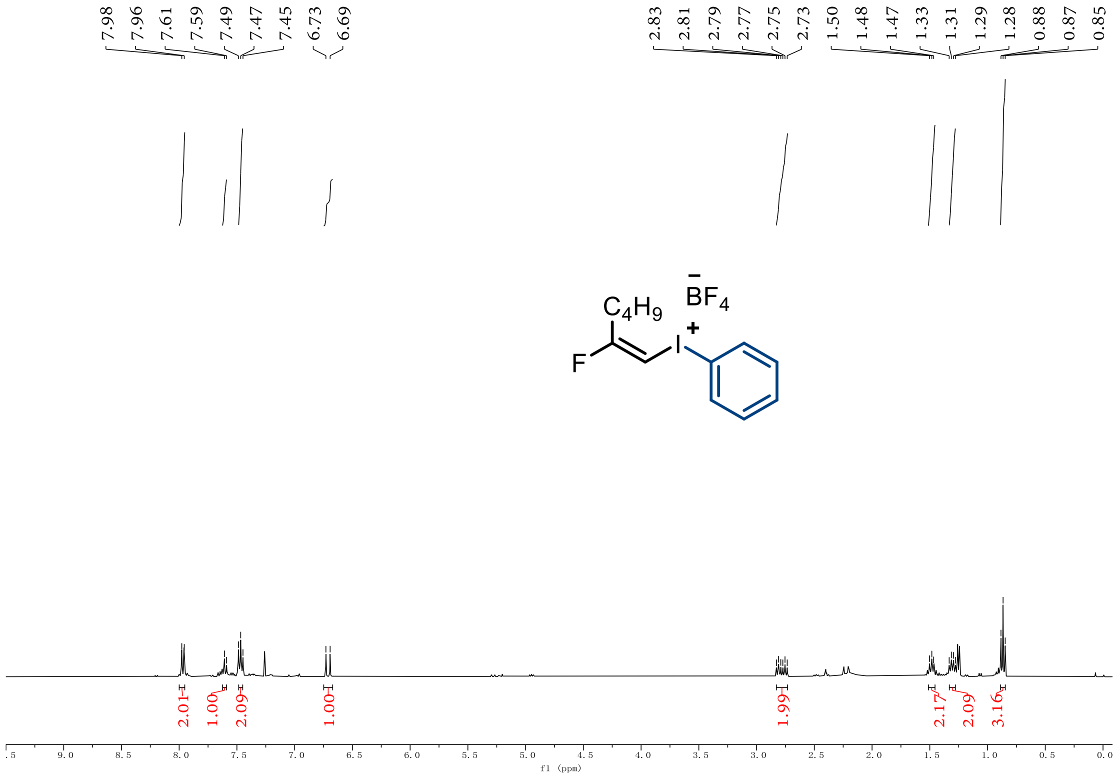

7.98
7.96
7.61
7.59
7.49
7.47
7.45
6.73
6.69
2.83
2.81
2.79
2.77
2.75
2.73
1.50
1.48
1.47
1.33
1.31
1.29
1.28
0.88
0.87
0.85
C4H9
BF4
F
I
2.01
1.00
2.09
1.00
1.99
2.17
2.09
3.16
f1 (ppm)

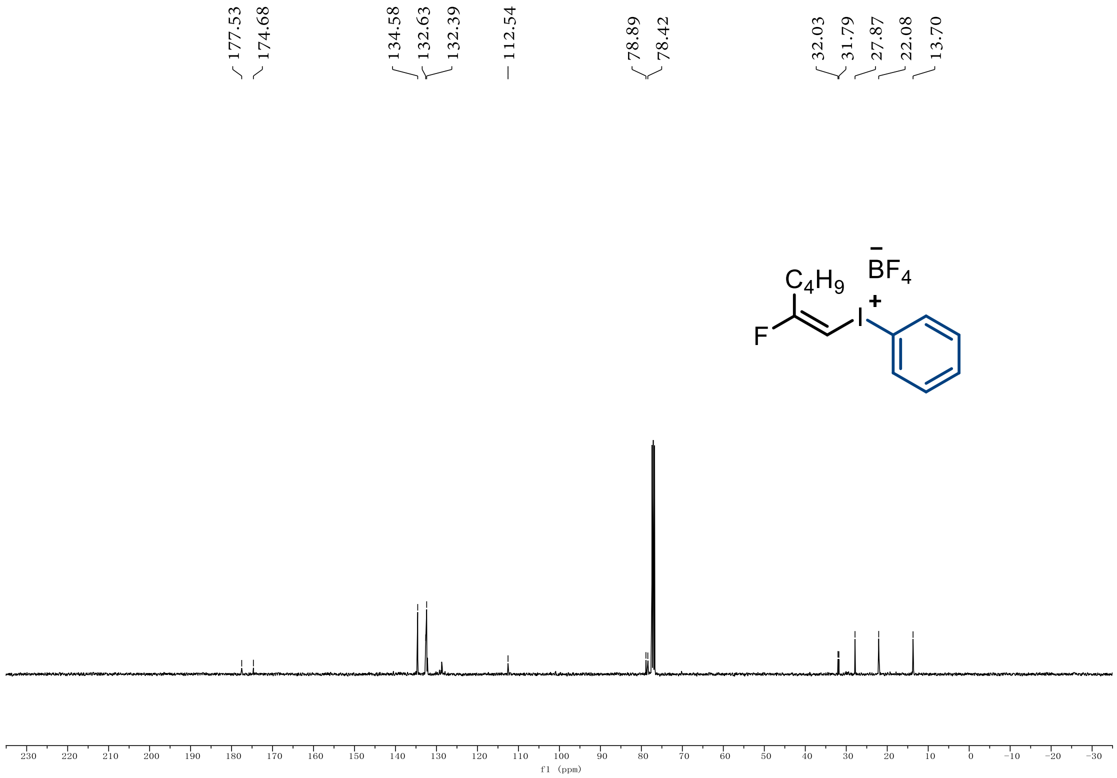
177.53
174.68
134.58
132.63
132.39
112.54
78.89
78.42
32.03
31.79
27.87
22.08
13.70
C4H9
BF4
F
I
f1 (ppm)

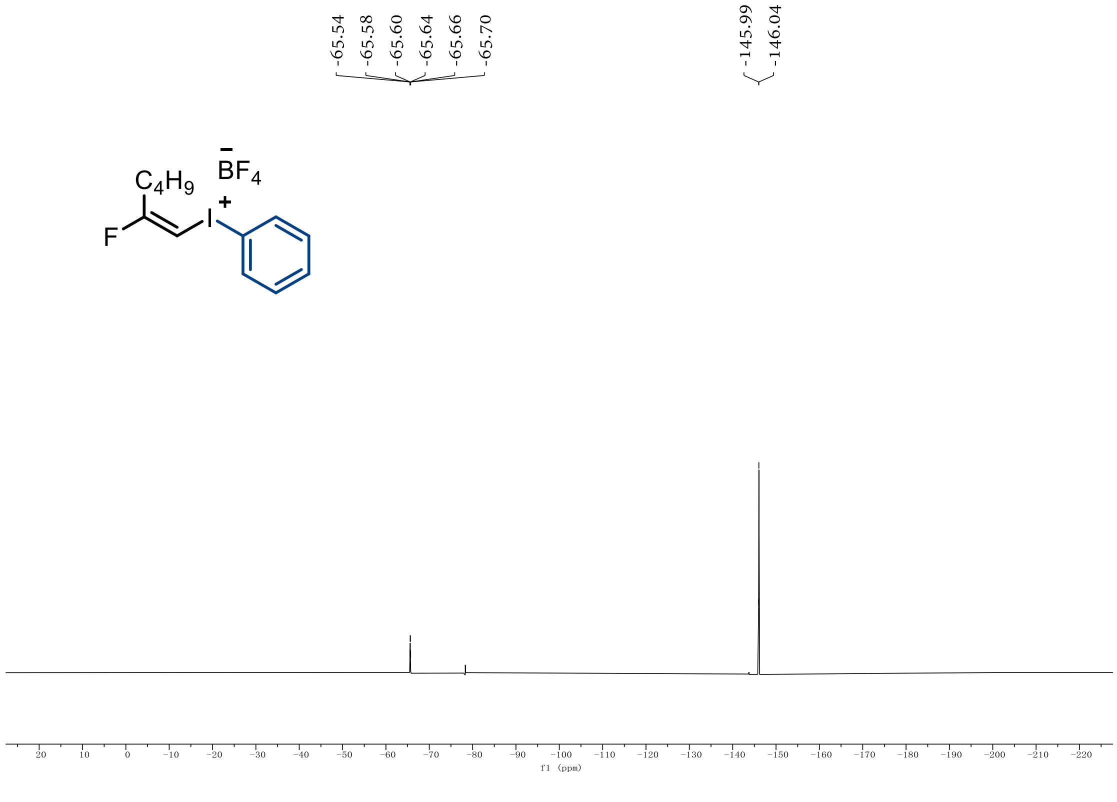
-65.54
-65.58
-65.60
-65.64
-65.66
-65.70
-145.99
-146.04
C4H9
BF4
F
I
f1 (ppm)

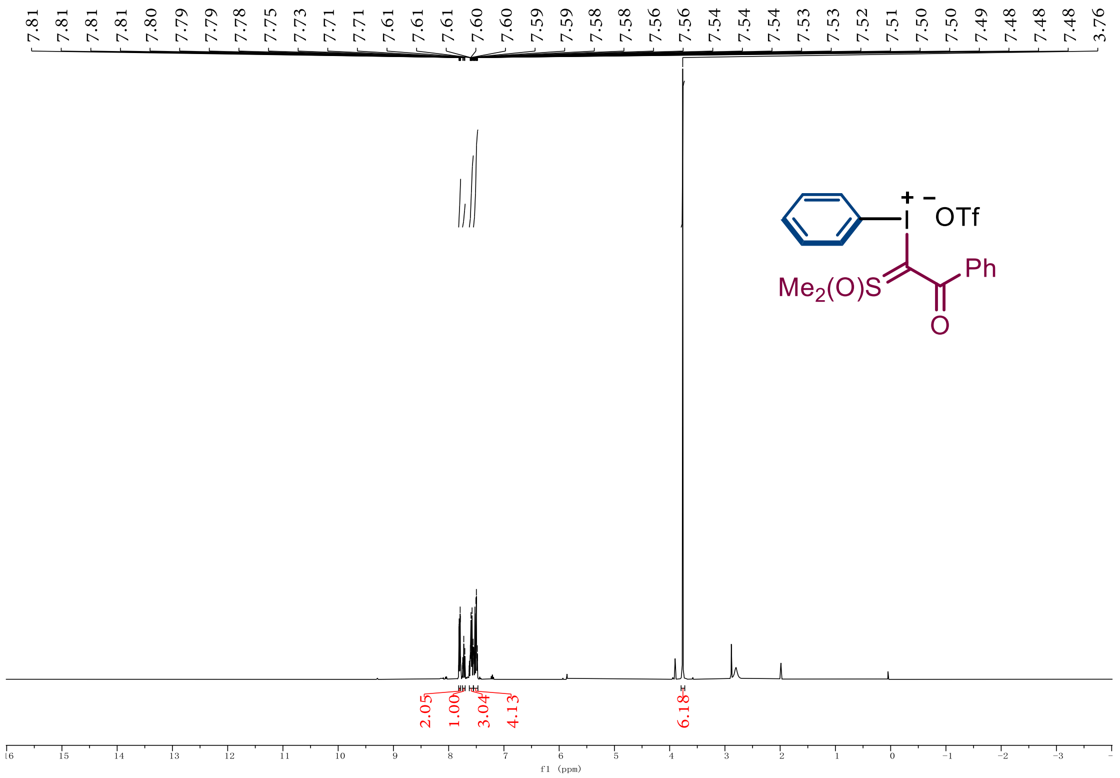

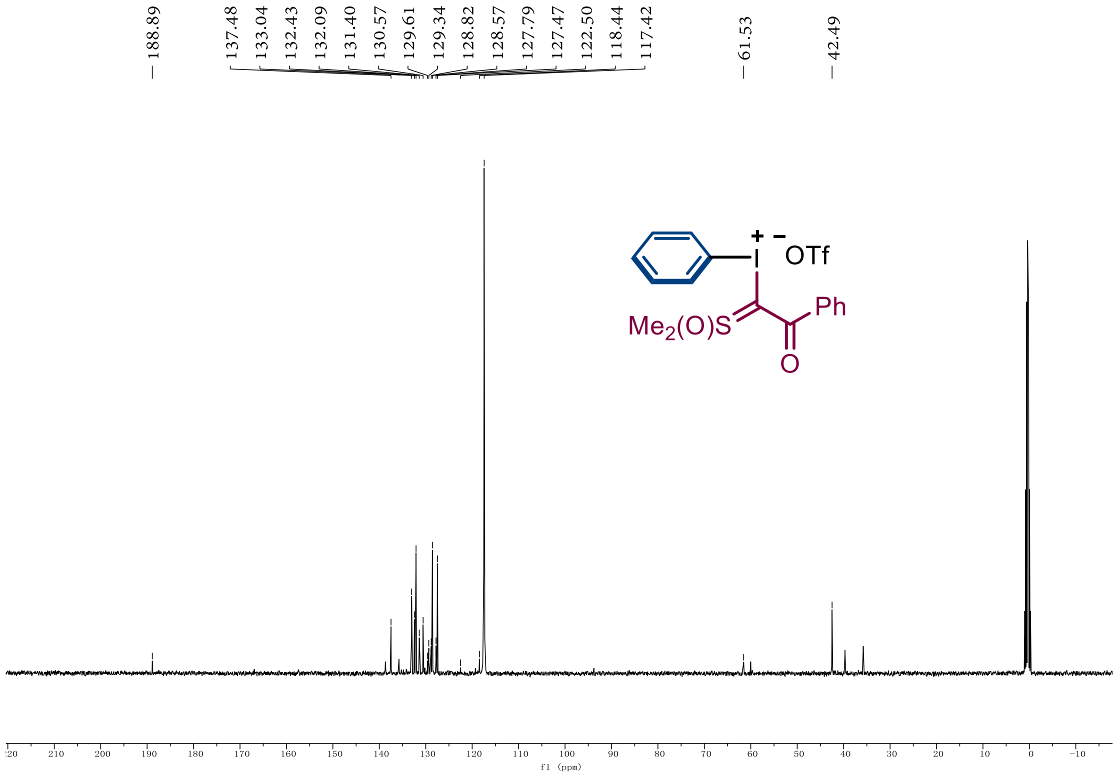

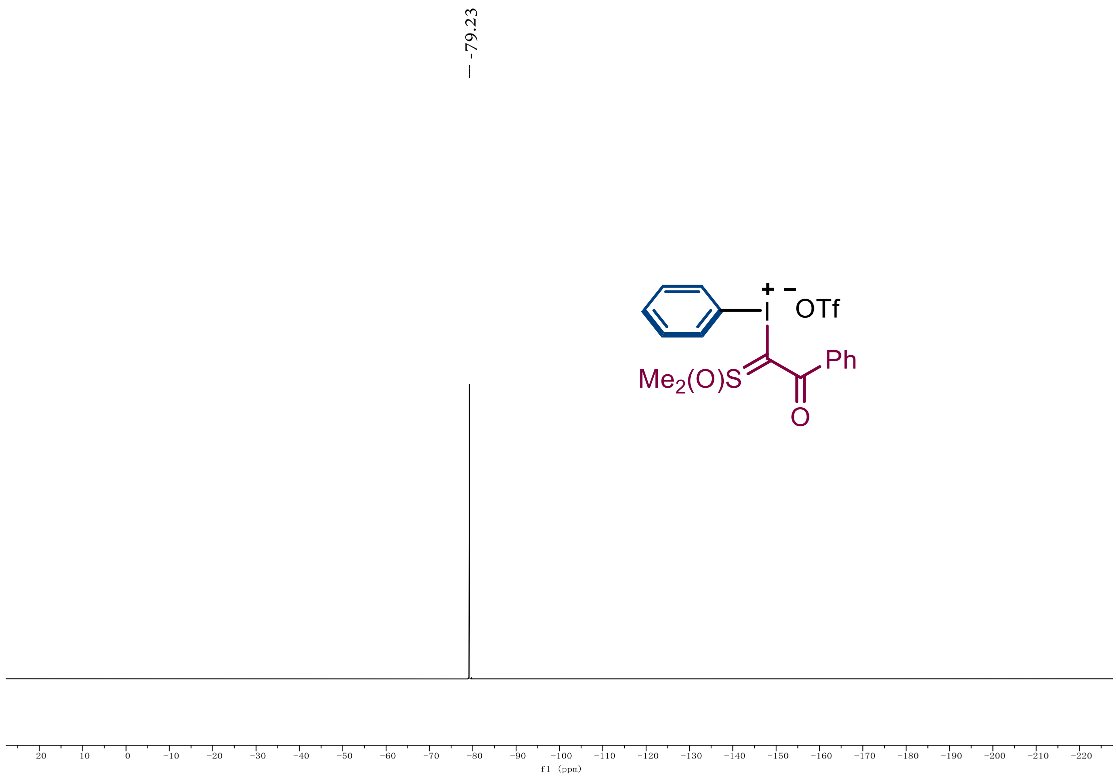

-79.23
OTf
Me2(O)S
Ph
O
f1 (ppm)

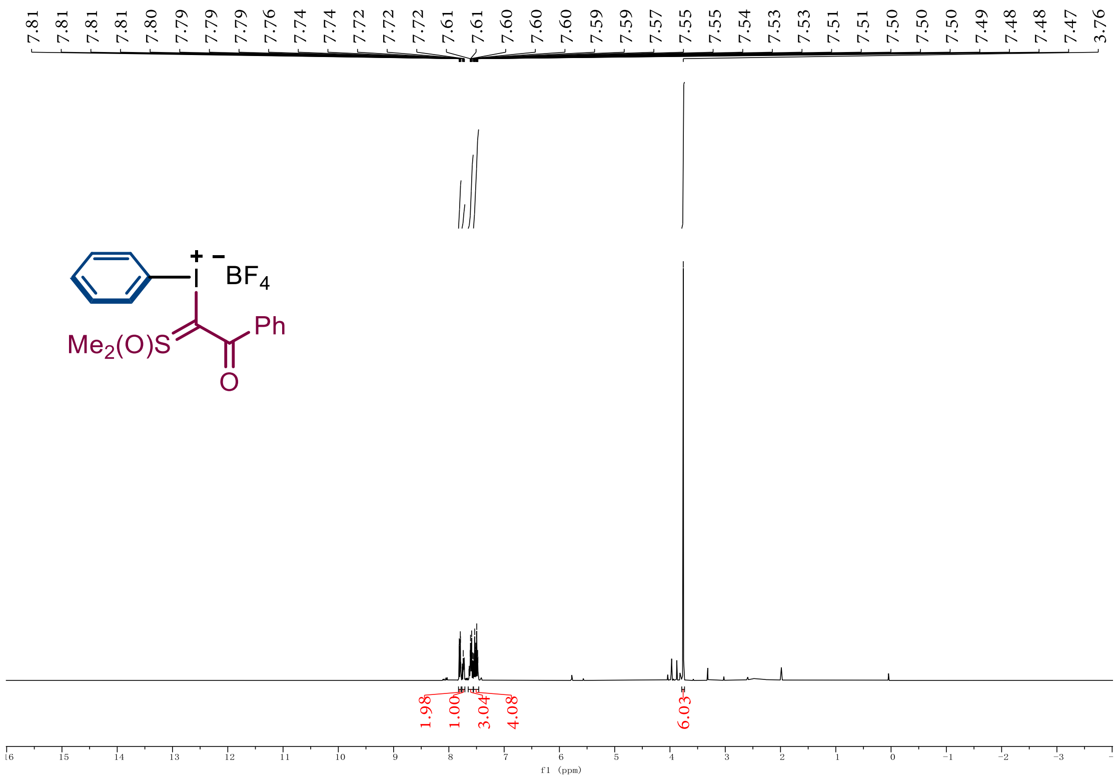

7.81
7.81
7.81
7.81
7.80
7.79
7.79
7.79
7.76
7.74
7.74
7.72
7.72
7.72
7.61
7.61
7.60
7.60
7.60
7.59
7.59
7.57
7.55
7.55
7.54
7.53
7.53
7.51
7.51
7.50
7.50
7.50
7.49
7.48
7.48
7.47
3.76
BF4
Me2(O)S
Ph
O
1.98
1.00
3.04
4.08
6.03
f1 (ppm)

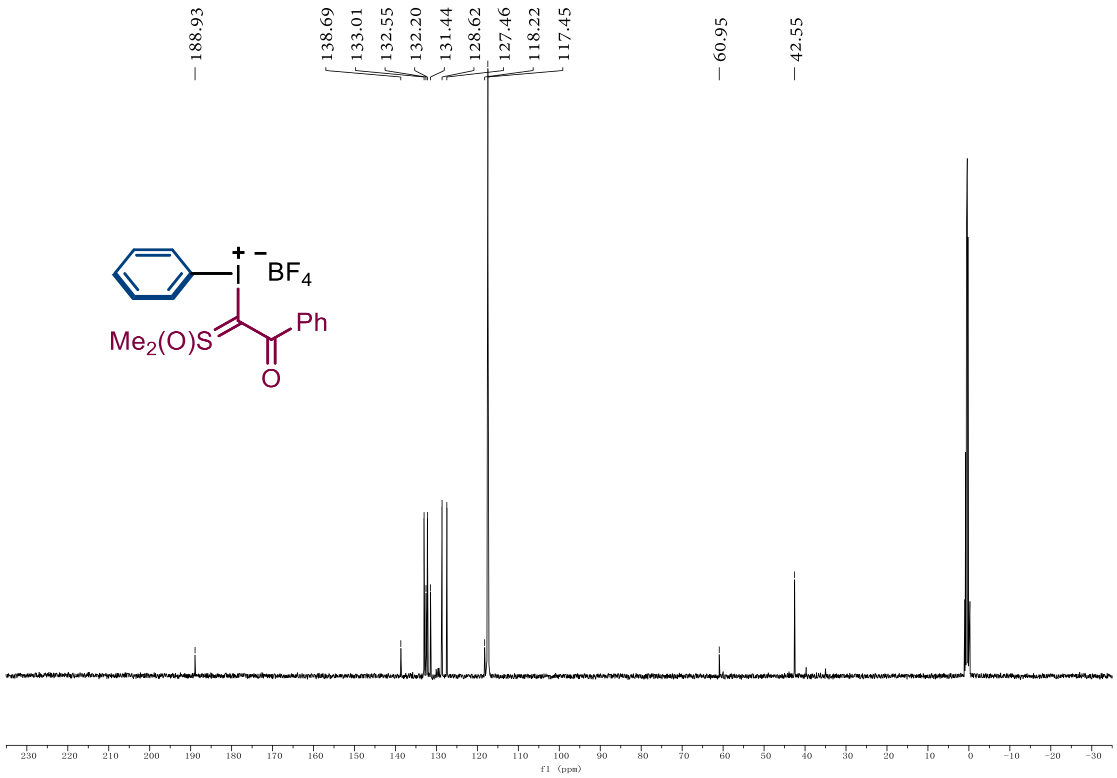
188.93
138.69
133.01
132.55
132.20
131.44
128.62
127.46
118.22
117.45
60.95
42.55
BF4
Me2(O)S
Ph
O
f1 (ppm)

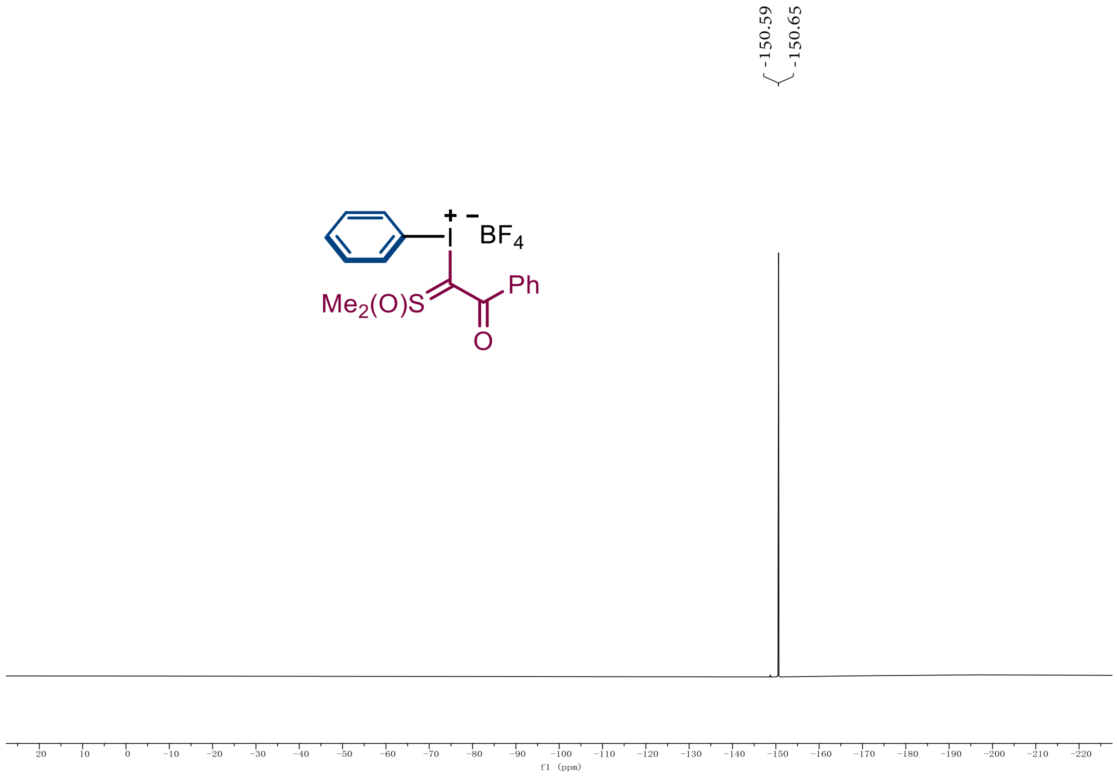
-150.59
-150.65
BF4
Me2(O)S
Ph
O
f1 (ppm)

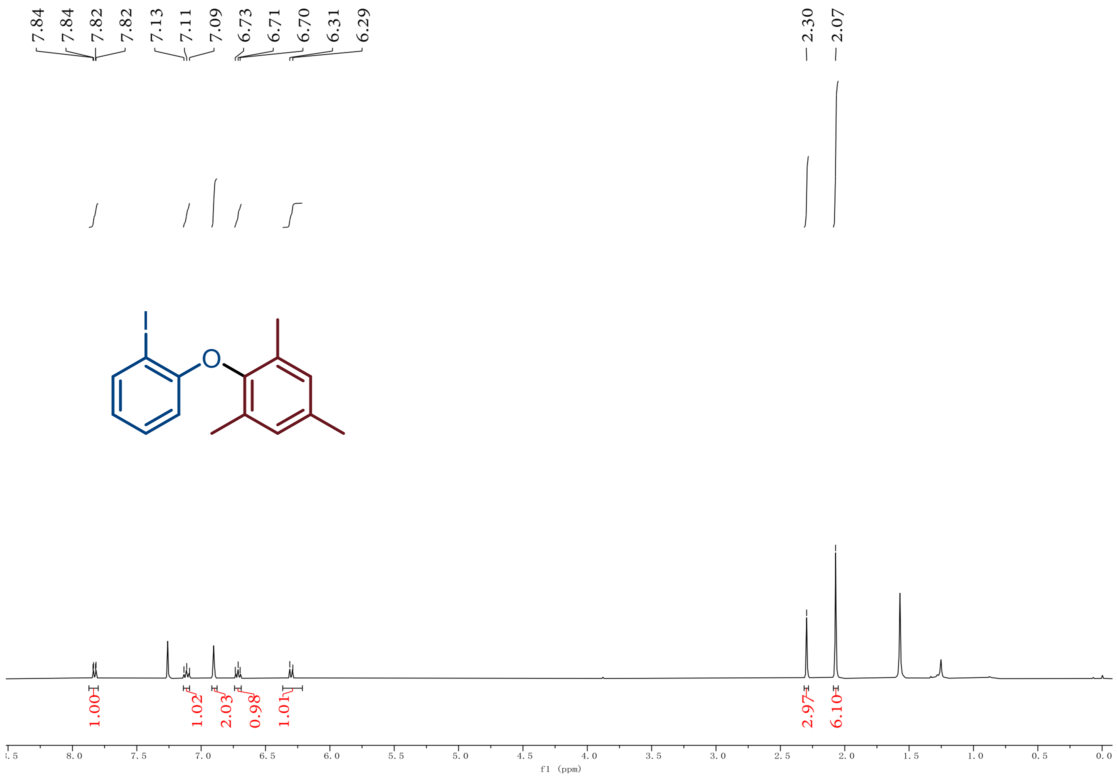
7.84
7.84
7.82
7.82
7.13
7.11
7.09
6.73
6.71
6.70
6.31
6.29
2.30
2.07
I
O
1.00
1.02
2.03
0.98
1.01
2.97
6.10
f1 (ppm)

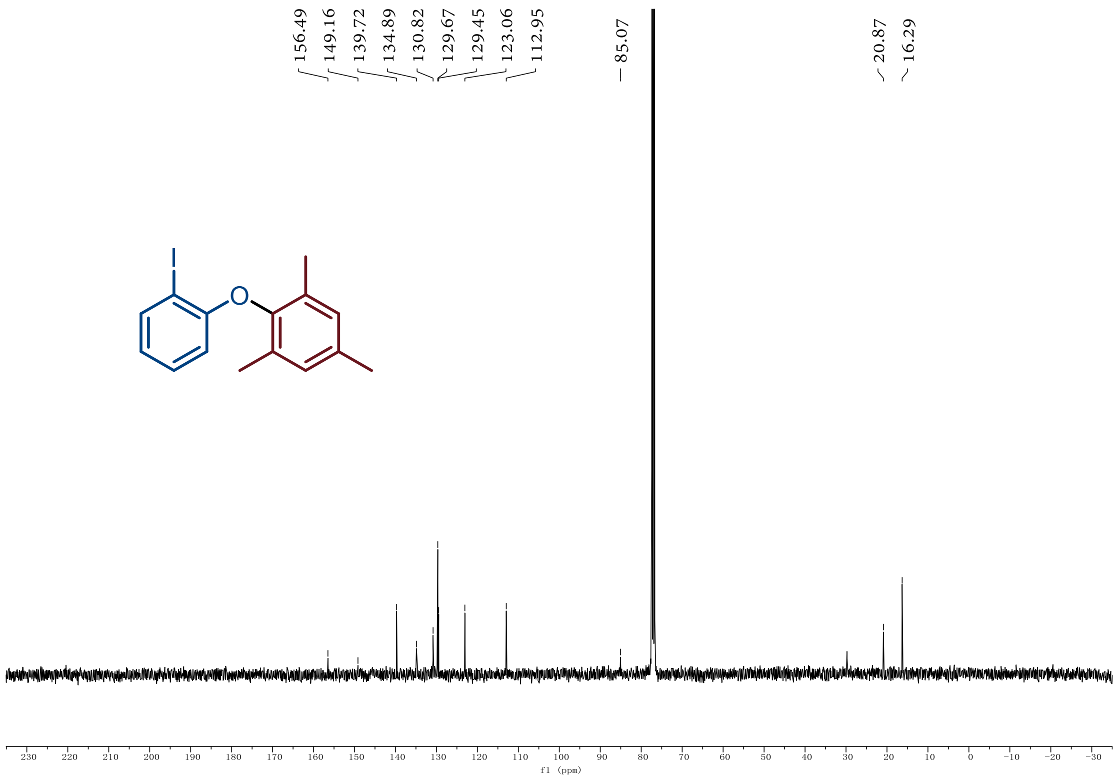
156.49
149.16
139.72
134.89
130.82
129.67
129.45
123.06
112.95
85.07
20.87
16.29
I
O
f1 (ppm)

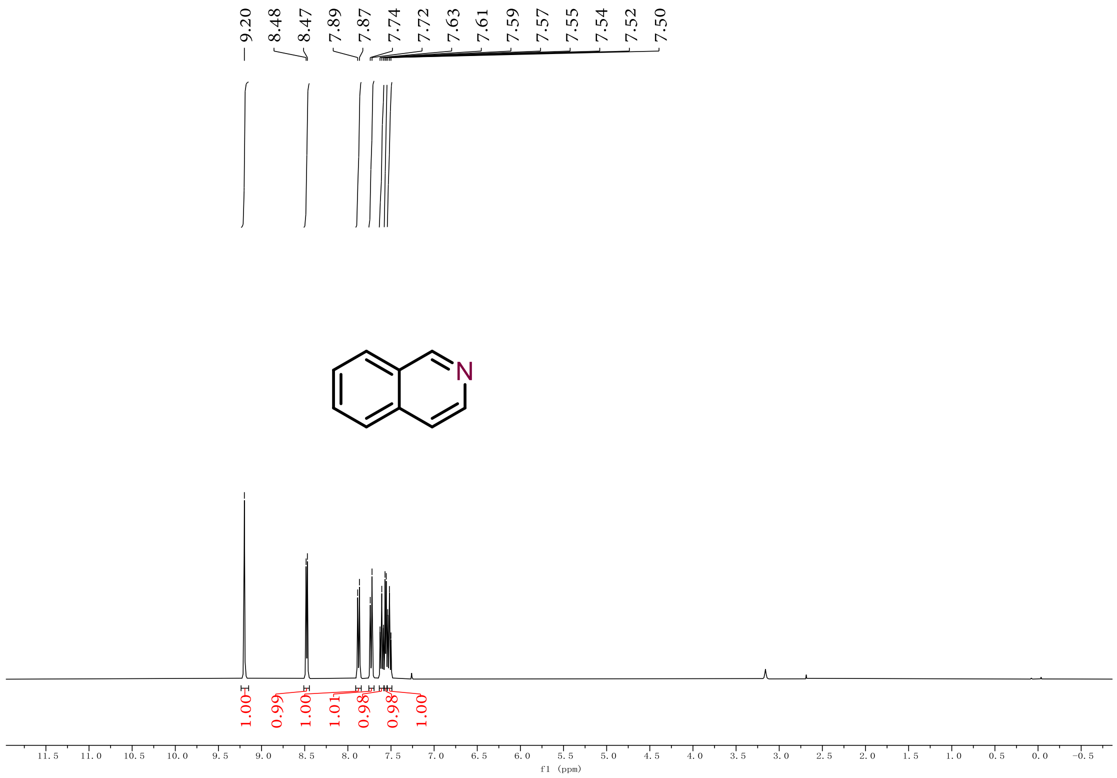
9.20
8.48
8.47
7.89
7.87
7.74
7.72
7.63
7.61
7.59
7.57
7.55
7.54
7.52
7.50
N
1.00
0.99
1.00
1.01
0.98
0.98
1.00
f1 (ppm)

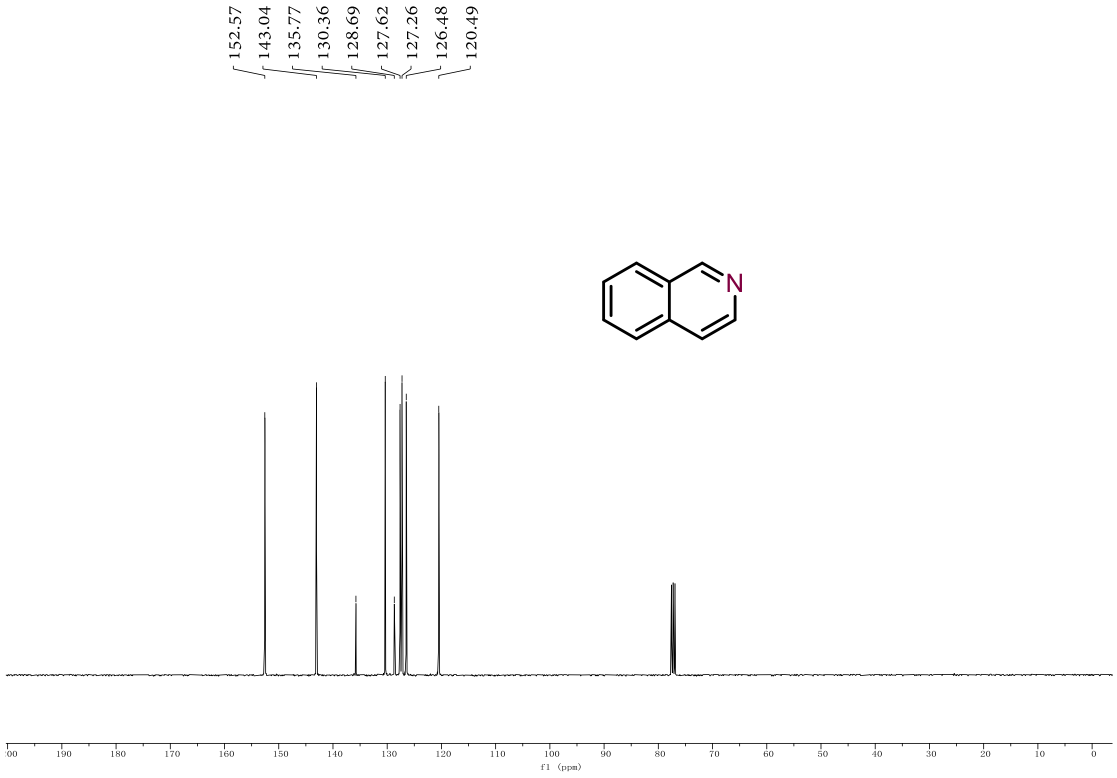
152.57
143.04
135.77
130.36
128.69
127.62
127.26
126.48
120.49
N
f1 (ppm)

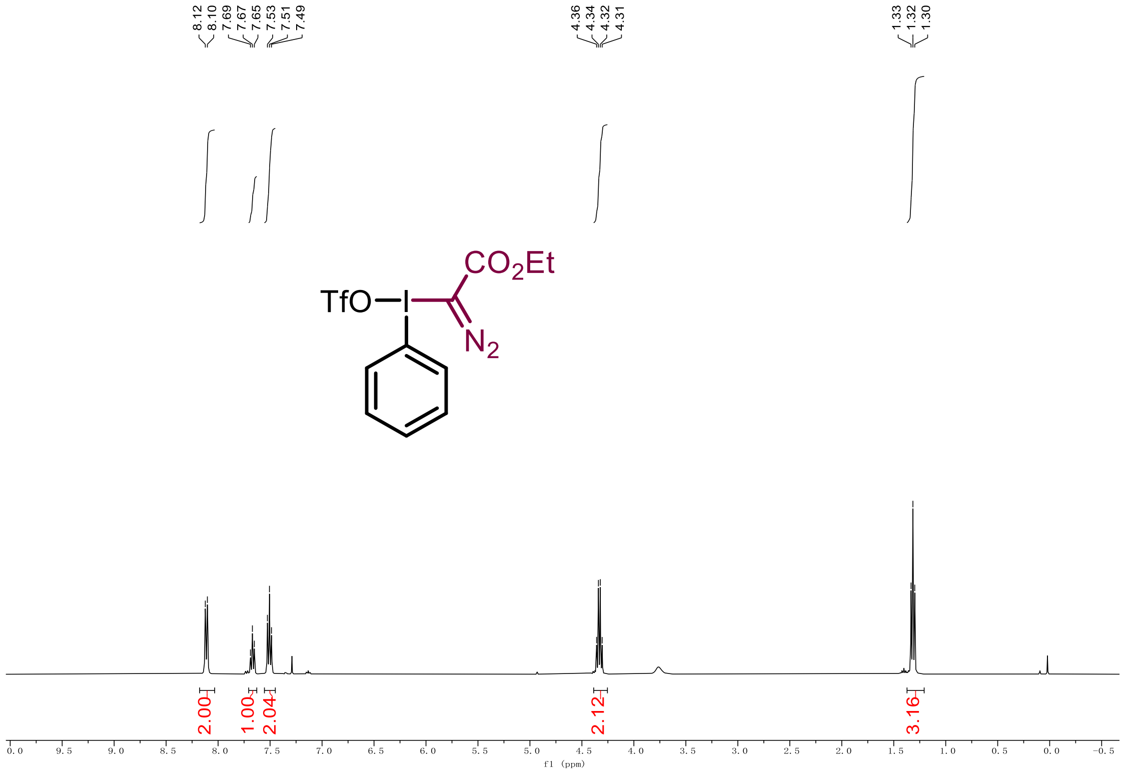
8.12
8.10
7.69
7.67
7.65
7.53
7.51
7.49
4.36
4.34
4.32
4.31
1.33
1.32
1.30
CO2Et
TfO
I
N2
2.00
1.00
2.04
2.12
3.16
f1 (ppm)

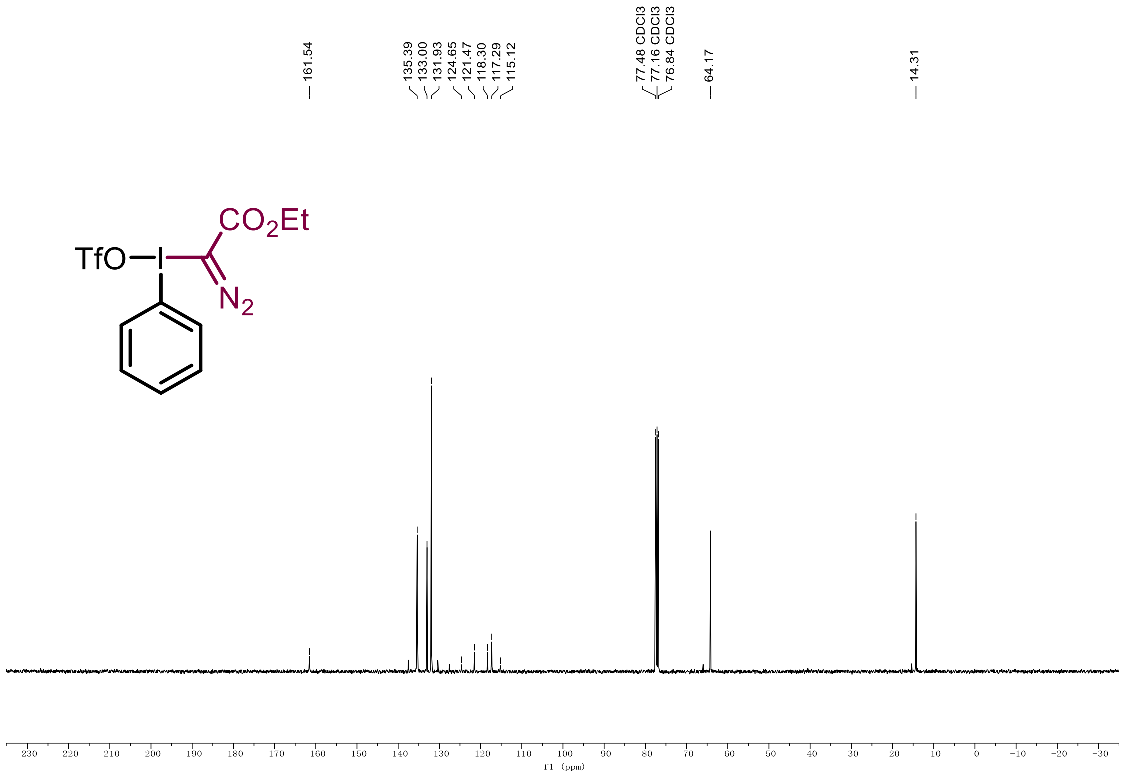
161.54
135.39
133.00
131.93
124.65
121.47
118.30
117.29
115.12
77.48 CDCl3
77.16 CDCl3
76.84 CDCl3
64.17
14.31
CO2Et
TfO
I
N2
f1 (ppm)

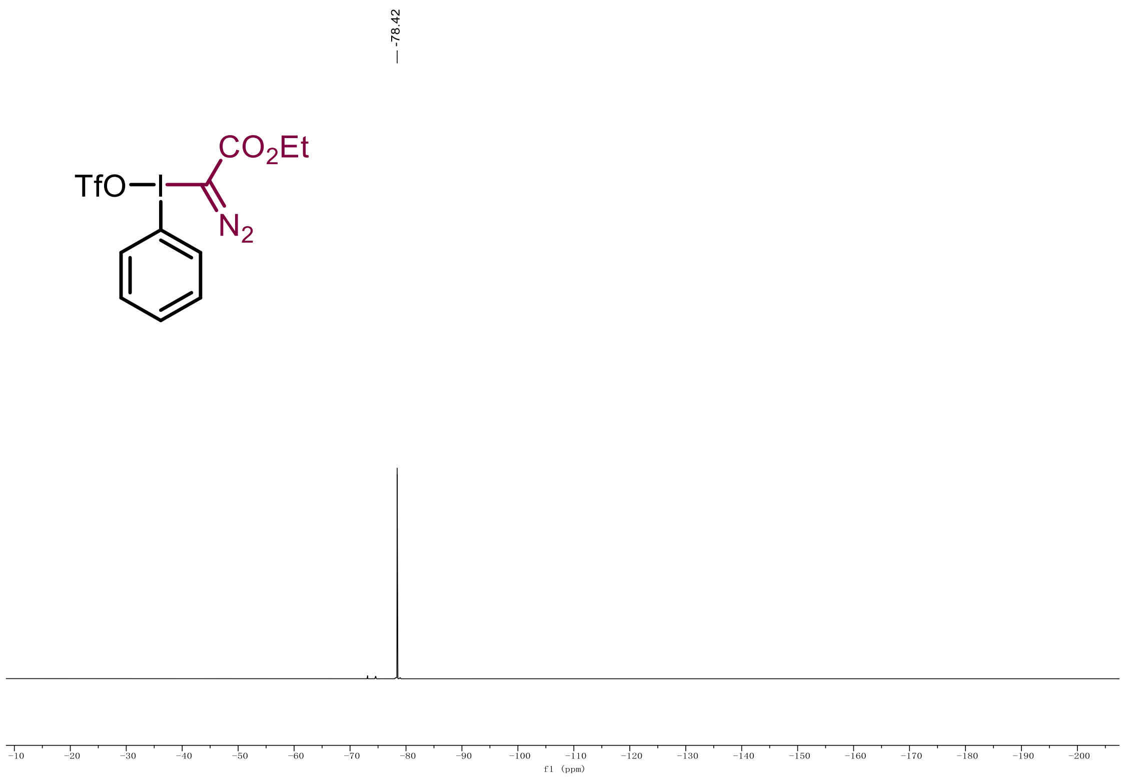
-78.42
CO2Et
TfO
I
N2
-10 -20 -30 -40 -50 -60 -70 -80 -90 -100 -110 -120 -130 -140 -150 -160 -170 -180 -190 -200
f1 (ppm)

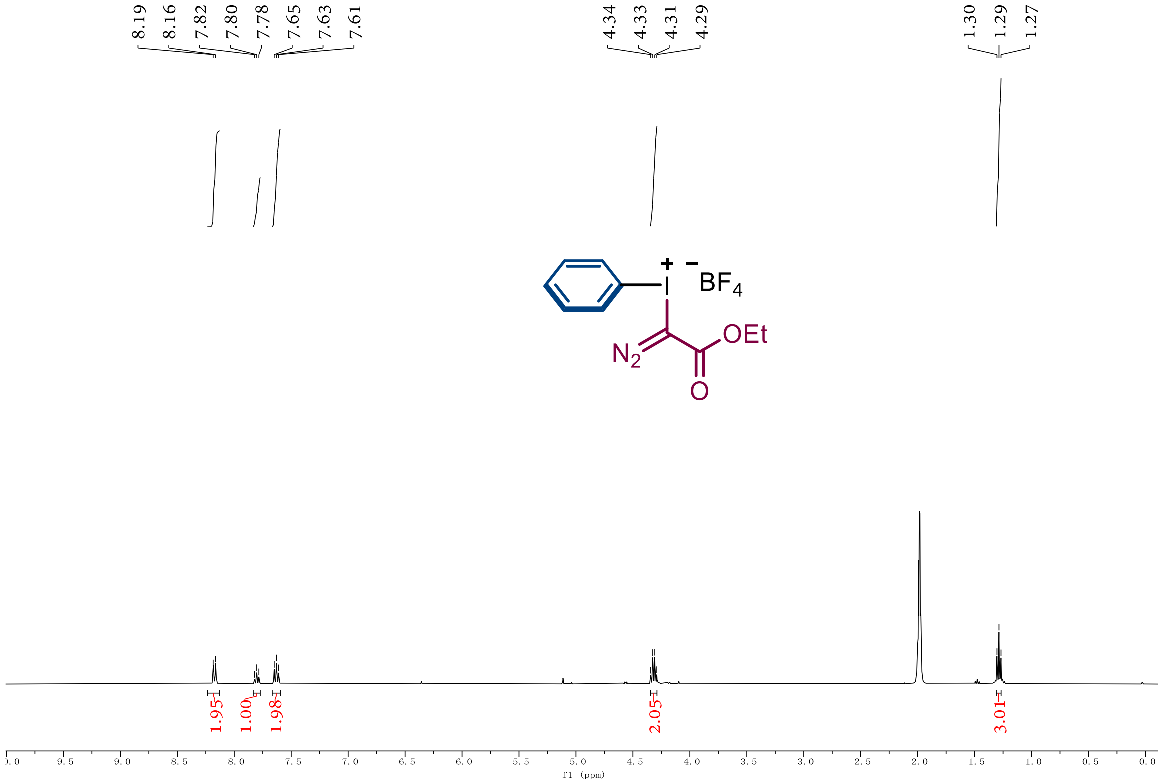
8.19 8.16 7.82 7.80 7.78 7.65 7.63 7.61
4.34 4.33 4.31 4.29
1.30 1.29 1.27
BF4
I
N2
OEt
O
1.95 1.00 1.98
2.05
3.01
9.5 9.0 8.5 8.0 7.5 7.0 6.5 6.0 5.5 5.0 4.5 4.0 3.5 3.0 2.5 2.0 1.5 1.0 0.5 0.0
f1 (ppm)

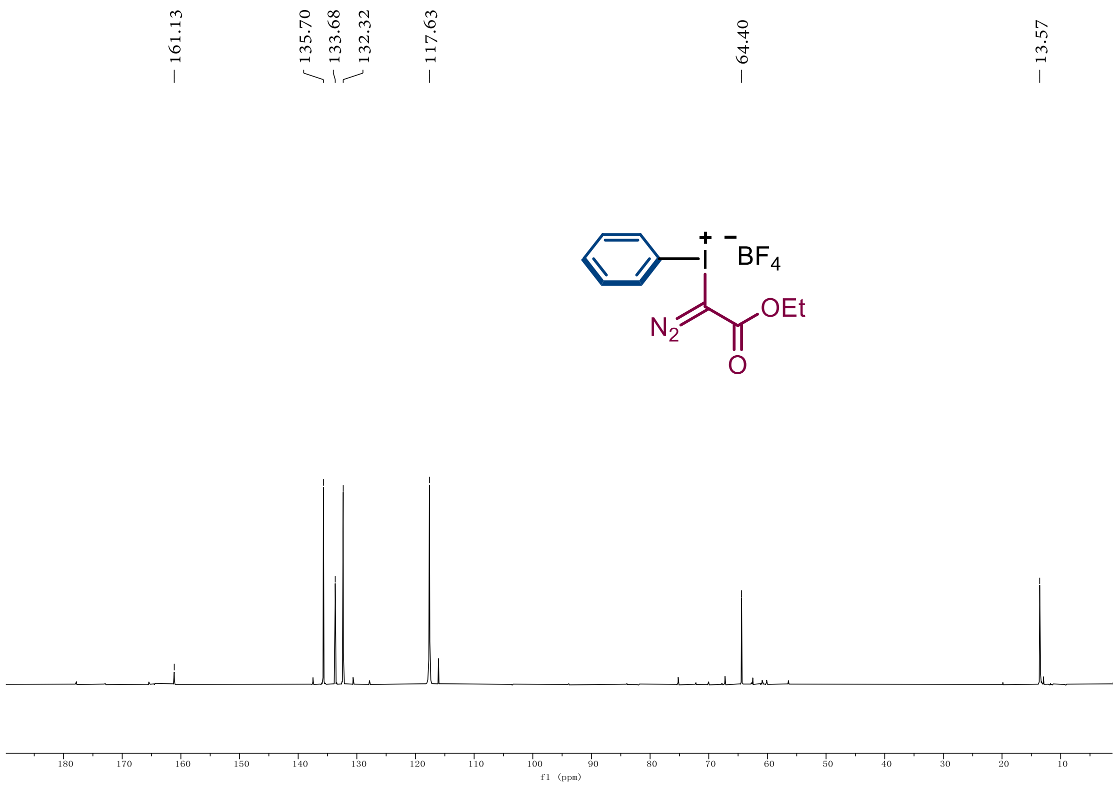
161.13
135.70
133.68
132.32
117.63
64.40
13.57
BF4
N2
OEt
O
180 170 160 150 140 130 120 110 100 90 80 70 60 50 40 30 20 10
f1 (ppm)

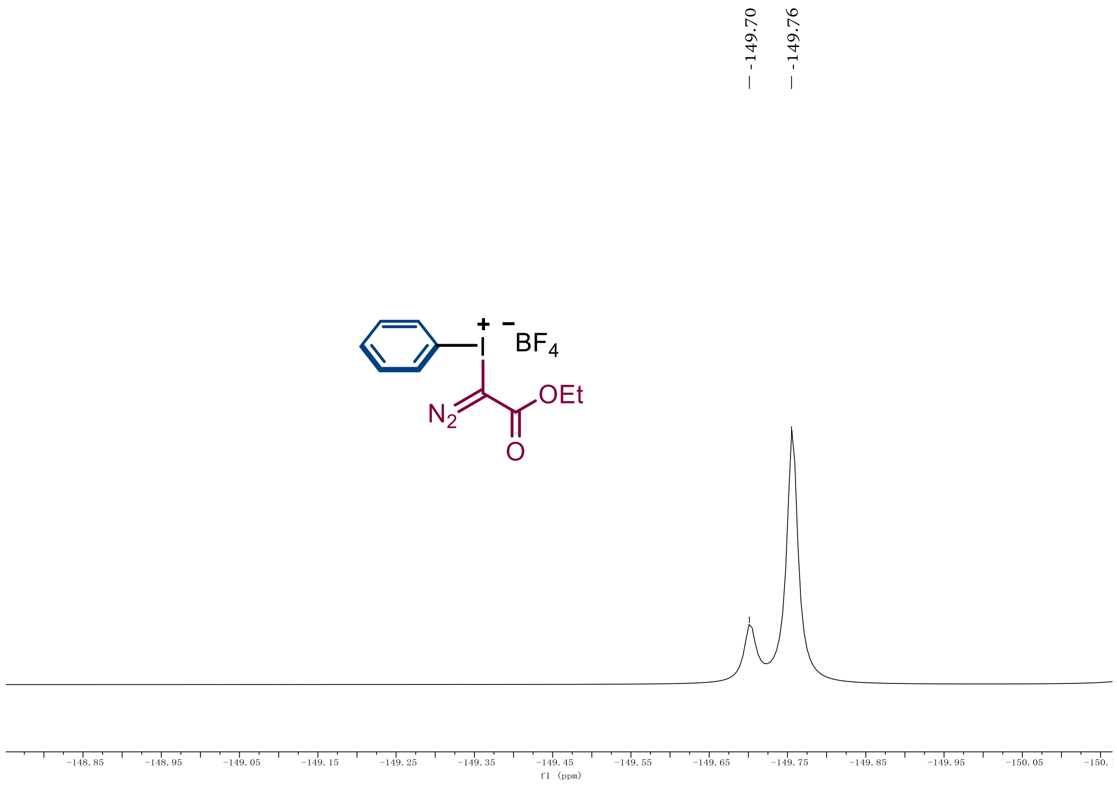
-149.70
-149.76
BF4
N2
OEt
O
-148.85 -148.95 -149.05 -149.15 -149.25 -149.35 -149.45 -149.55 -149.65 -149.75 -149.85 -149.95 -150.05
f1 (ppm)

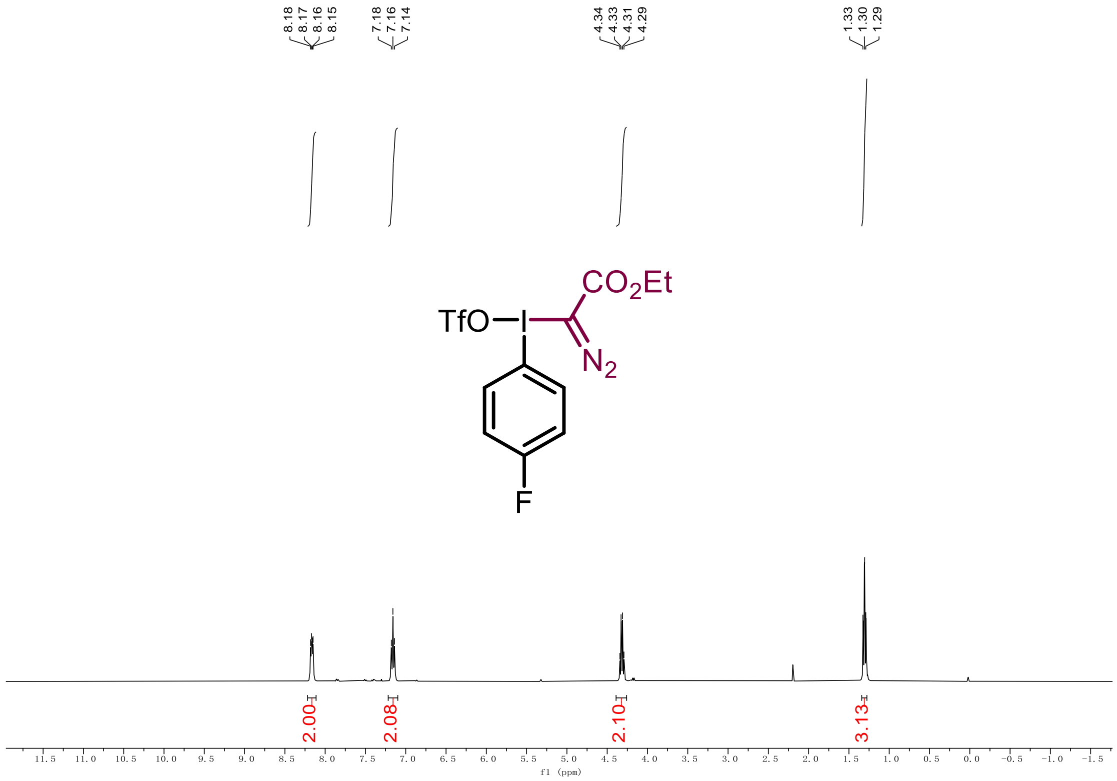

8.18
8.17
8.16
8.15
7.18
7.16
7.14
4.34
4.33
4.31
4.29
1.33
1.30
1.29
CO2Et
TfO
I
N2
F
2.00
2.08
2.10
3.13
f1 (ppm)

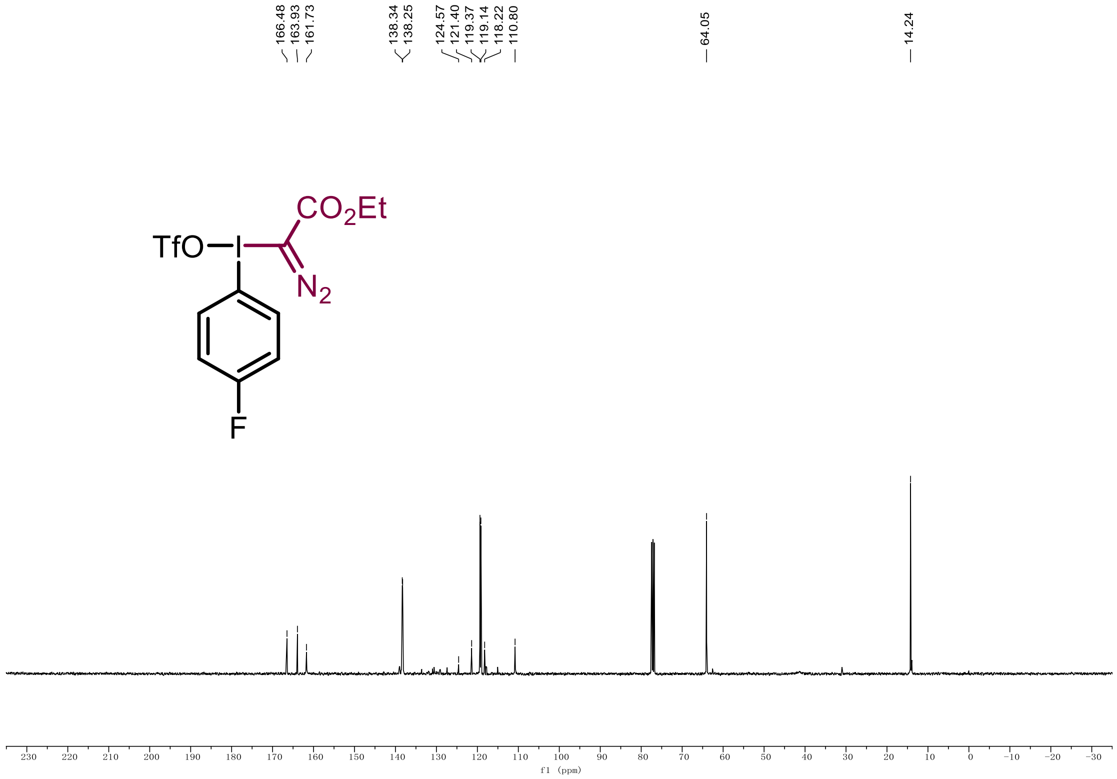

166.48
163.93
161.73
138.34
138.25
124.57
121.40
119.37
119.14
118.22
110.80
64.05
14.24
CO2Et
TfO
I
N2
F
f1 (ppm)

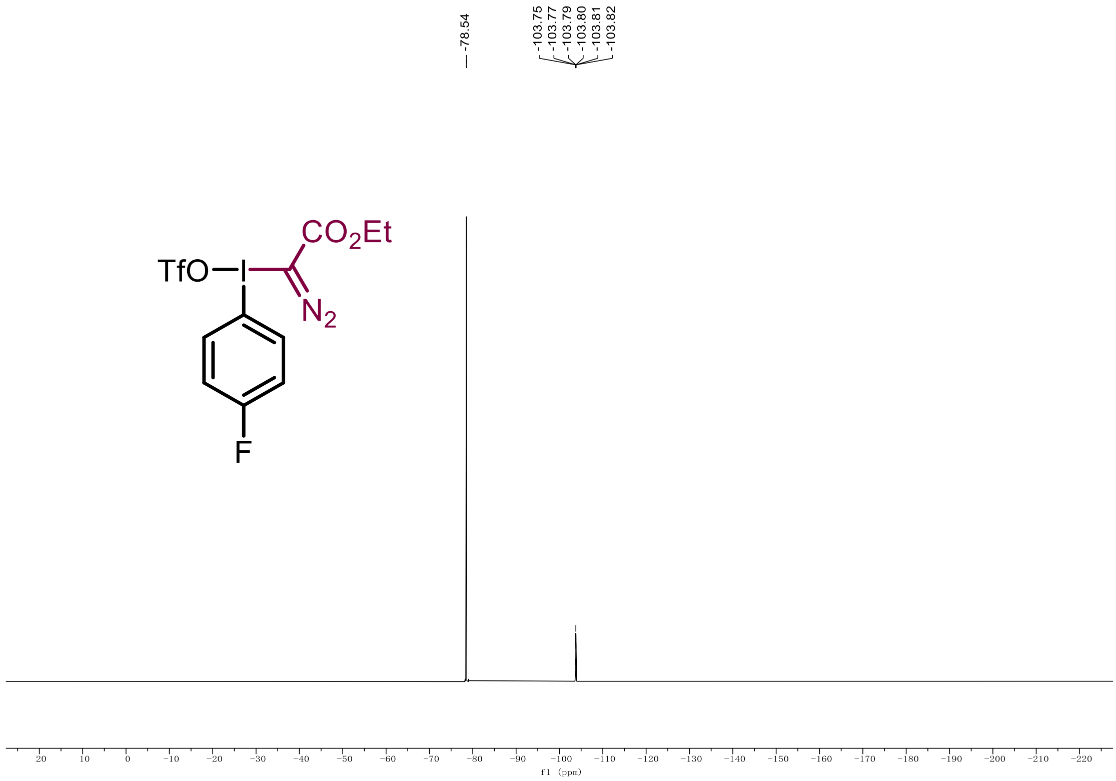
-78.54
-103.75
-103.77
-103.79
-103.80
-103.81
-103.82
CO2Et
TfO
I
N2
F
f1 (ppm)

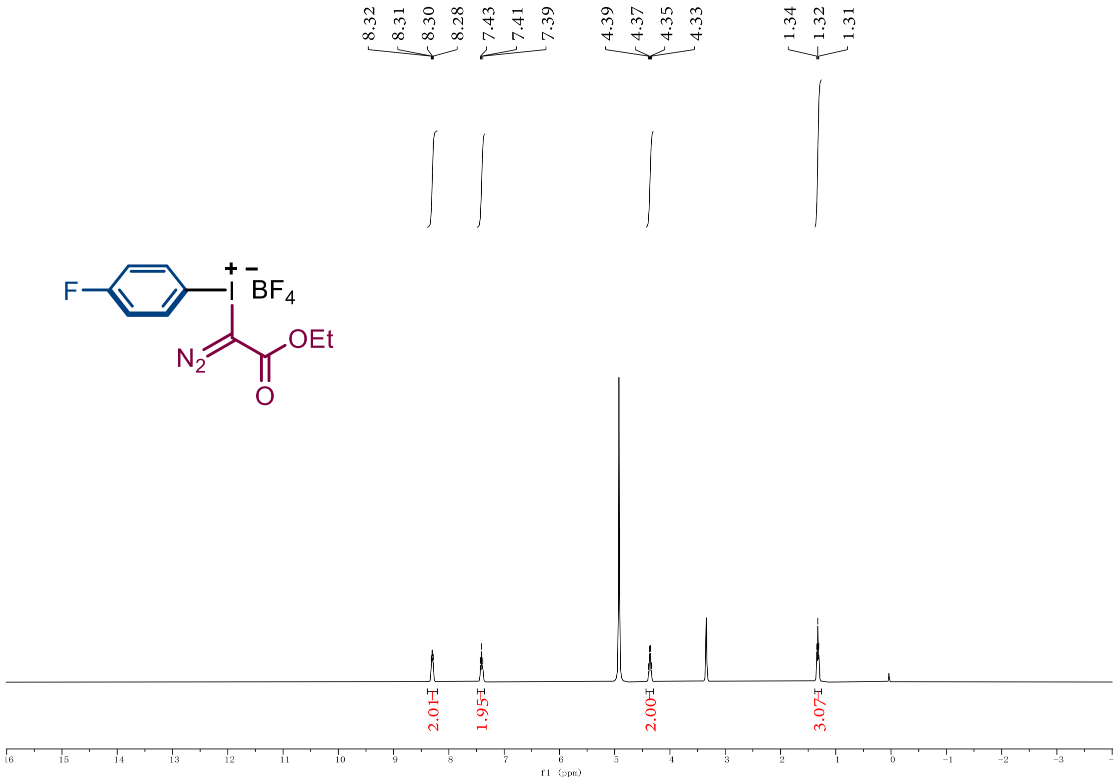
8.32
8.31
8.30
8.28
7.43
7.41
7.39
4.39
4.37
4.35
4.33
1.34
1.32
1.31
F
I
BF4
OEt
N2
O
2.01
1.95
2.00
3.07
f1 (ppm)

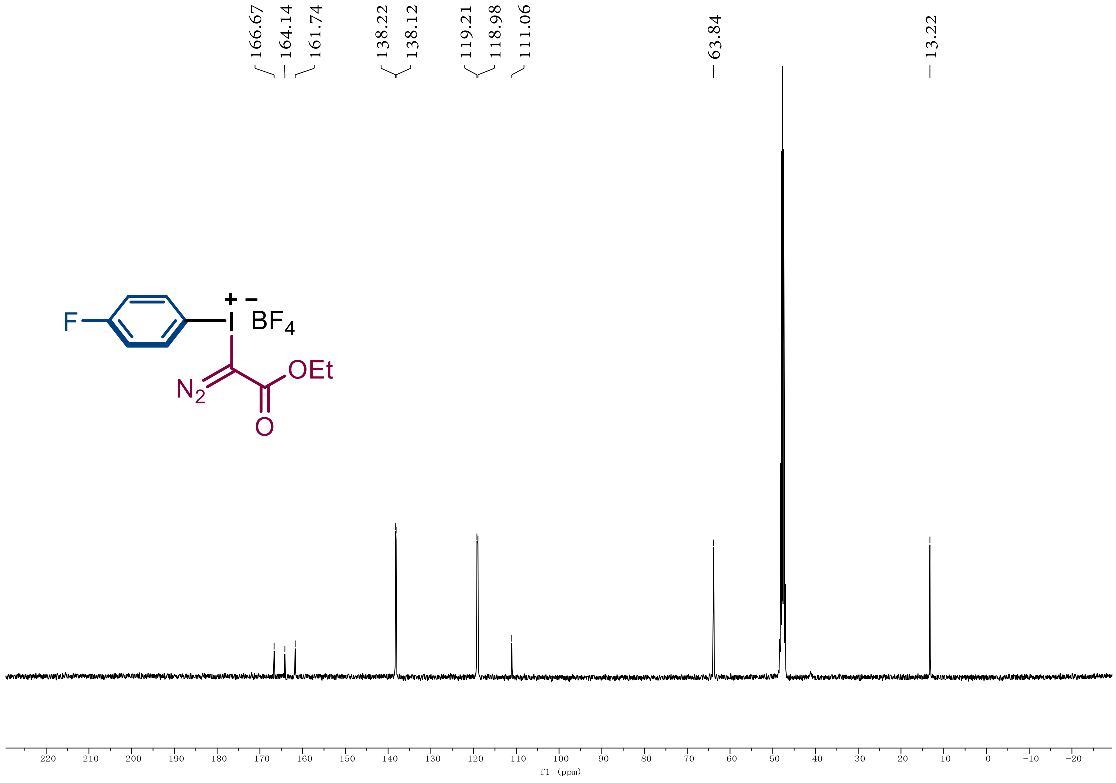
166.67
164.14
161.74
138.22
138.12
119.21
118.98
111.06
63.84
13.22
F
I
BF4
N2
OEt
O
f1 (ppm)

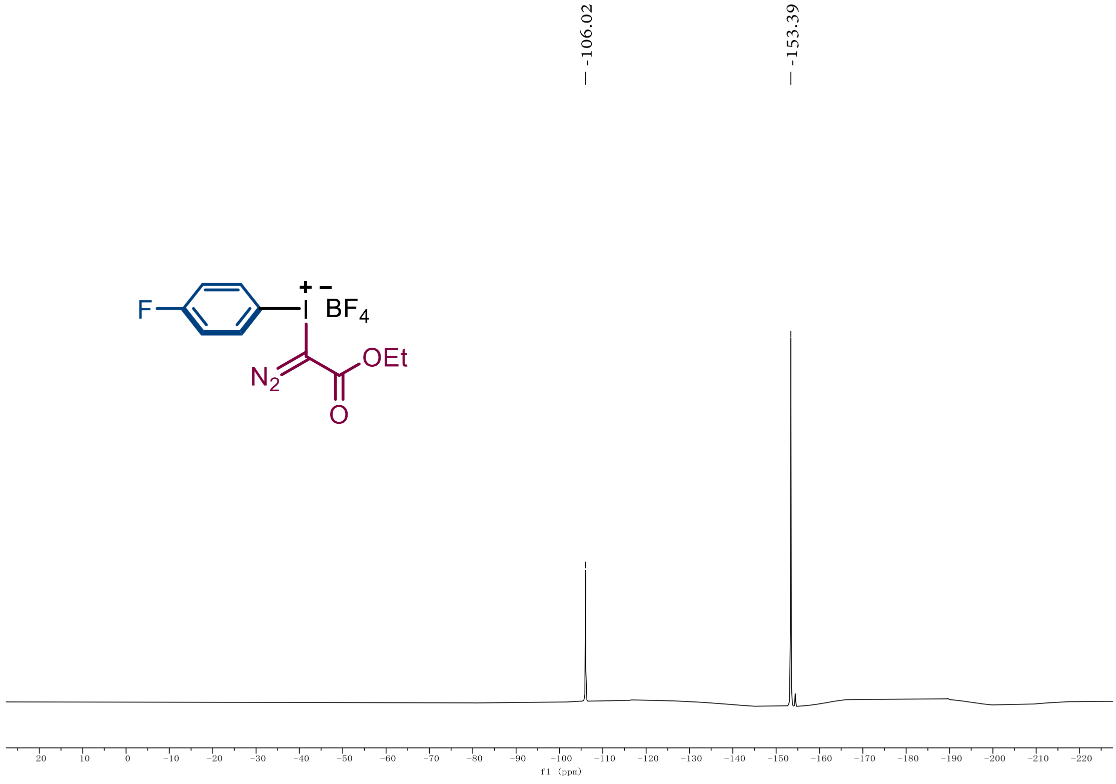
-106.02
-153.39
F
I
BF4
N2
OEt
O
f1 (ppm)

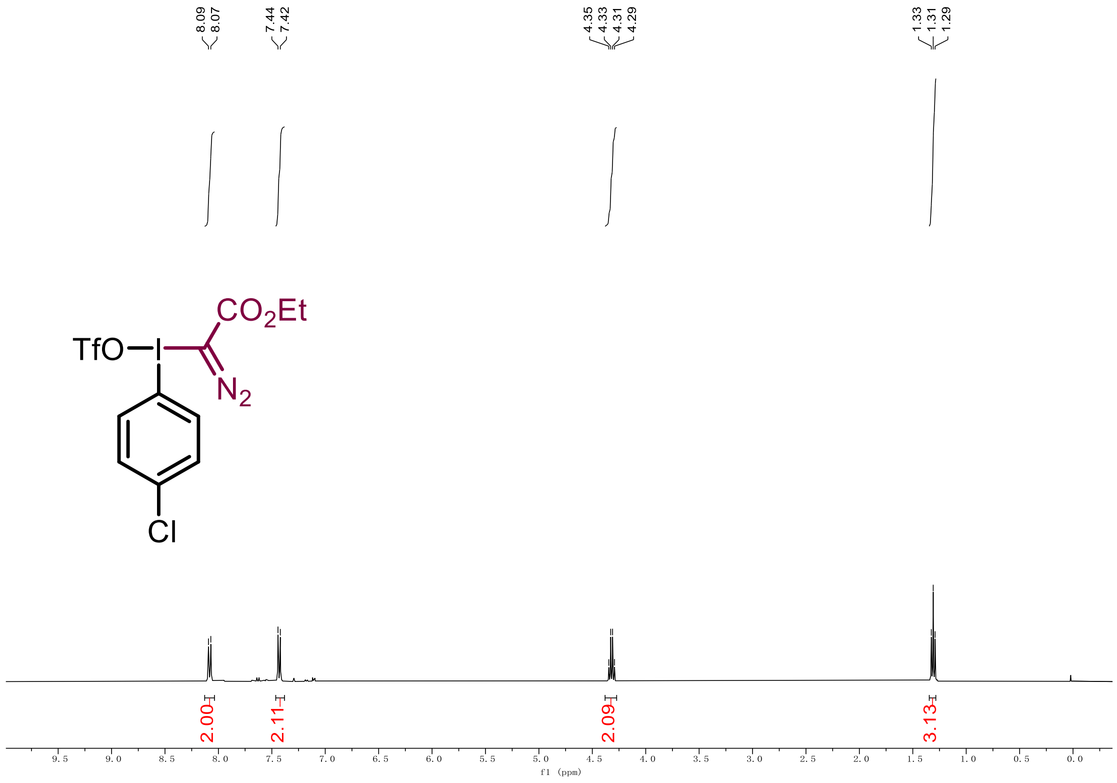
8.09
8.07
7.44
7.42
4.35
4.33
4.31
4.29
1.33
1.31
1.29
TfO
I
CO2Et
N2
Cl
2.00
2.11
2.09
3.13
f1 (ppm)

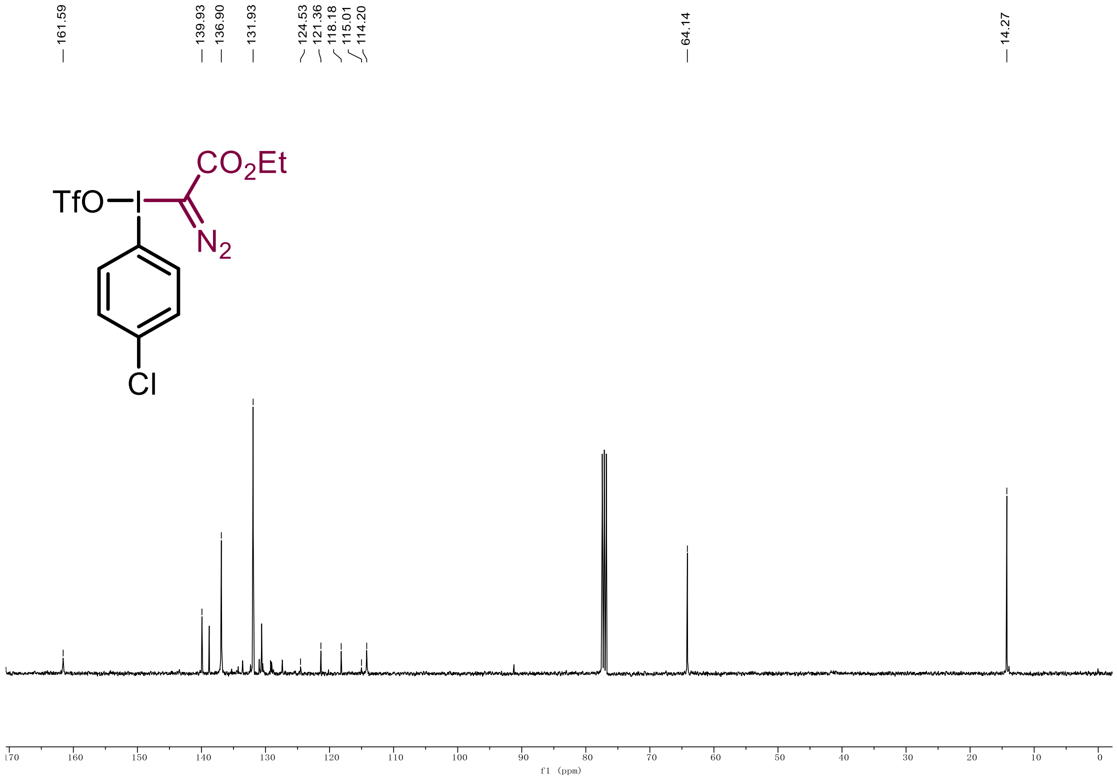
161.59
139.93
136.90
131.93
124.53
121.36
118.18
115.01
114.20
64.14
14.27
TfO
I
CO2Et
N2
Cl
f1 (ppm)

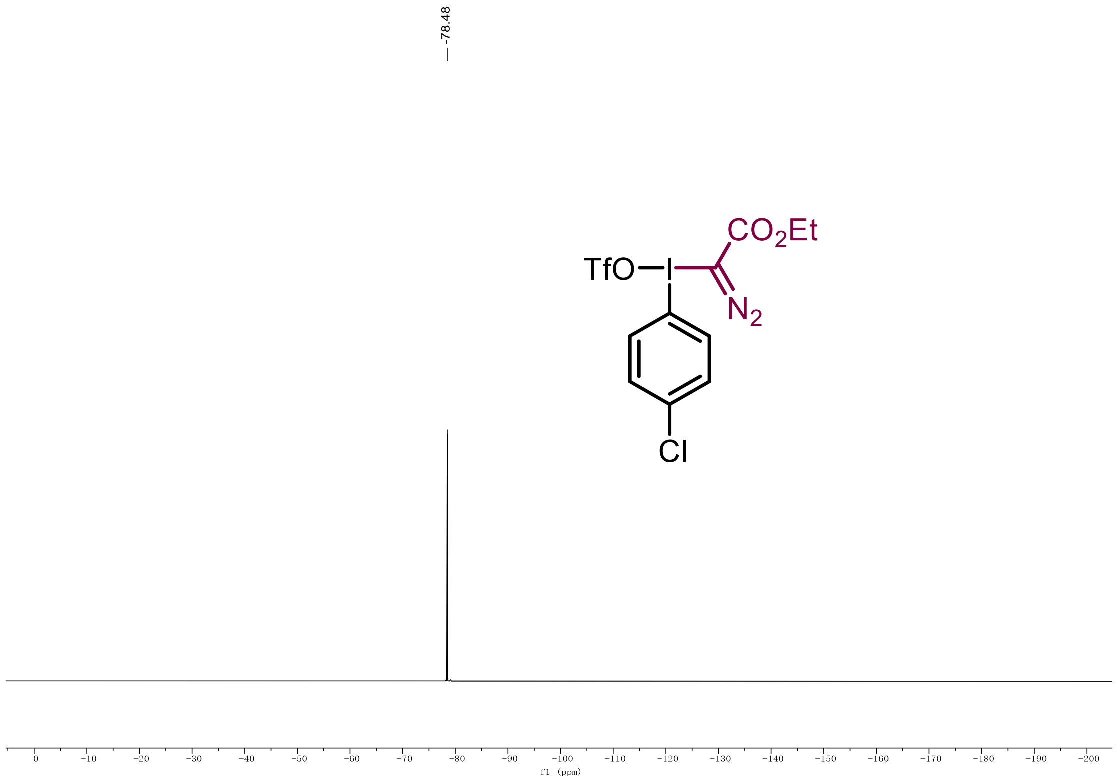
-78.48
CO2Et
TfO
I
N2
Cl
f1 (ppm)

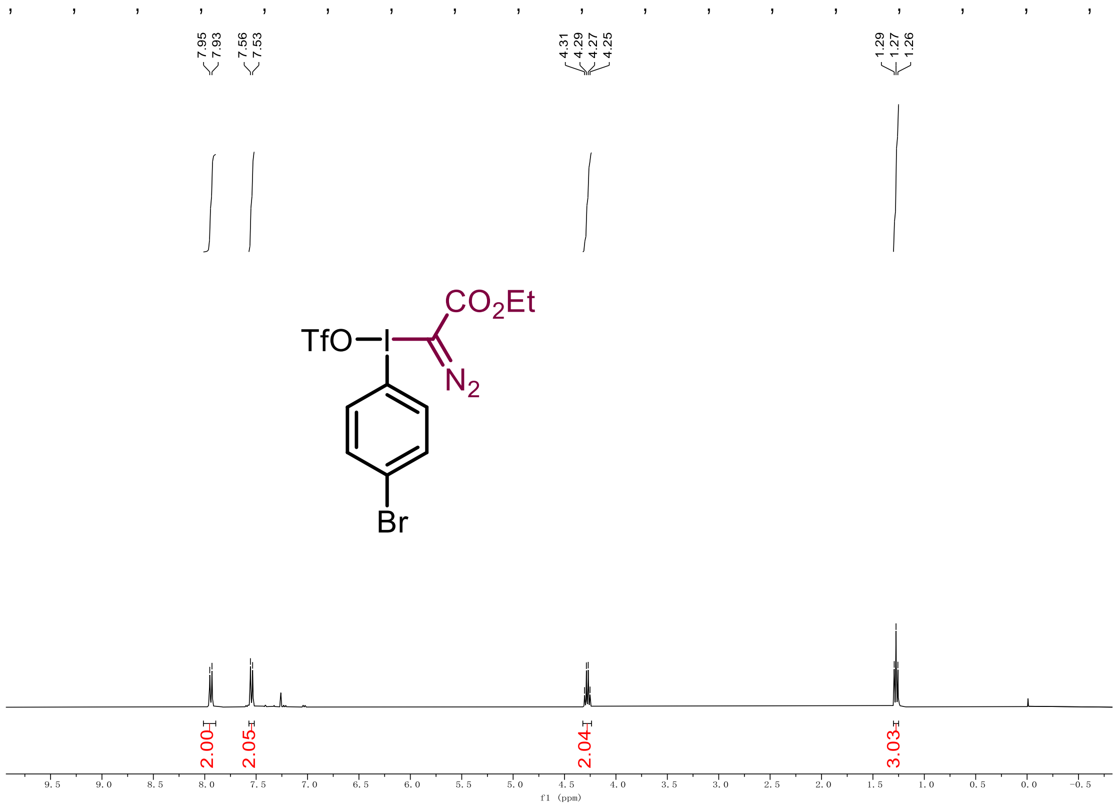
7.95
7.93
7.56
7.53
4.31
4.29
4.27
4.25
1.29
1.27
1.26
CO2Et
TfO
I
N2
Br
2.00
2.05
2.04
3.03
f1 (ppm)

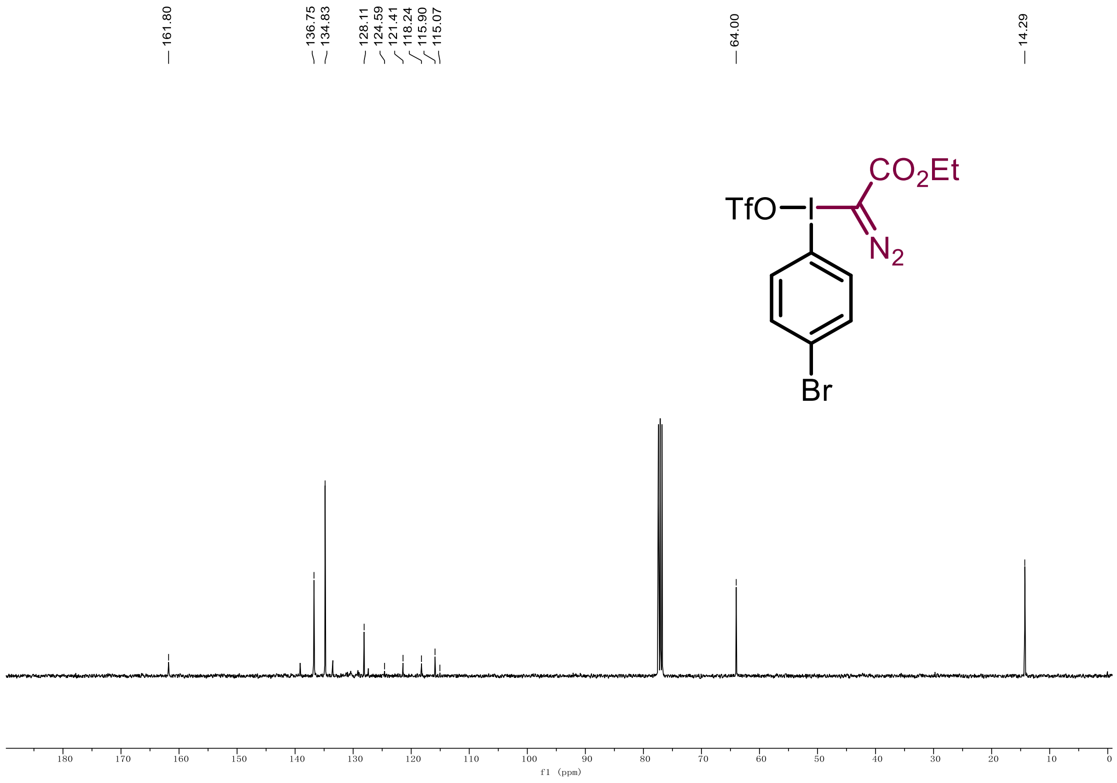
161.80
136.75
134.83
128.11
124.59
121.41
118.24
115.90
115.07
64.00
14.29
CO2Et
TfO—I
N2
Br
180 170 160 150 140 130 120 110 100 90 80 70 60 50 40 30 20 10 0
f1 (ppm)

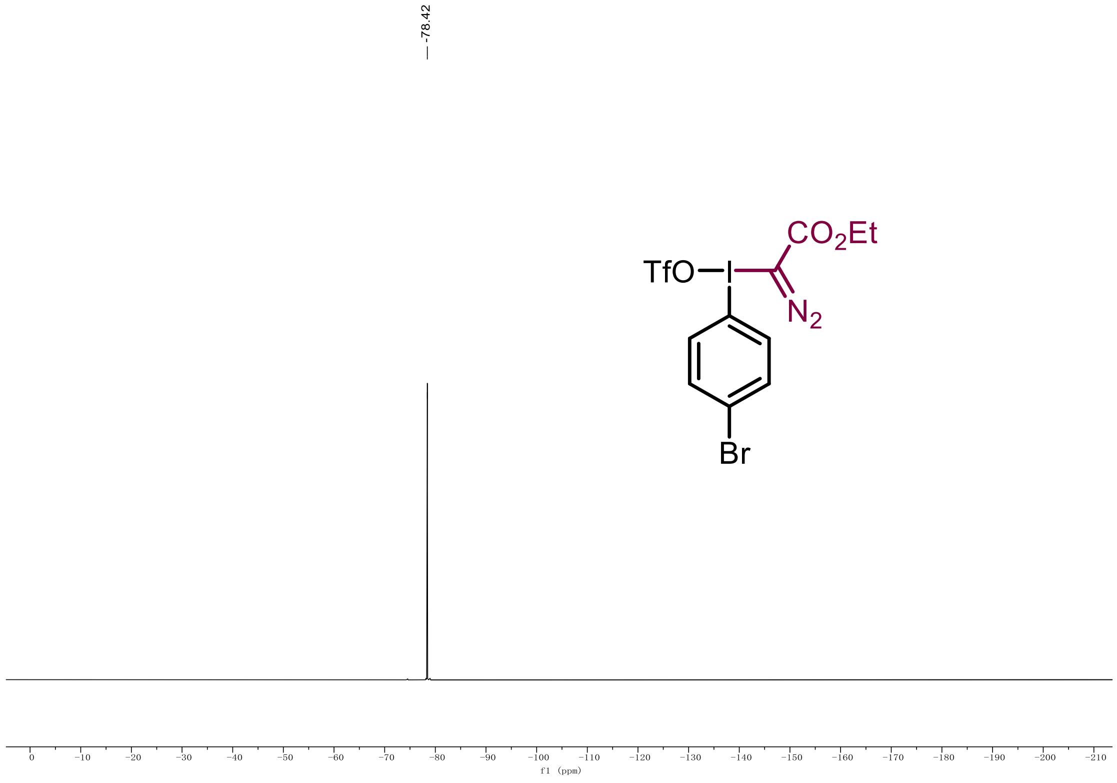
-78.42
CO2Et
TfO—I
N2
Br
0 −10 −20 −30 −40 −50 −60 −70 −80 −90 −100 −110 −120 −130 −140 −150 −160 −170 −180 −190 −200 −210
f1 (ppm)

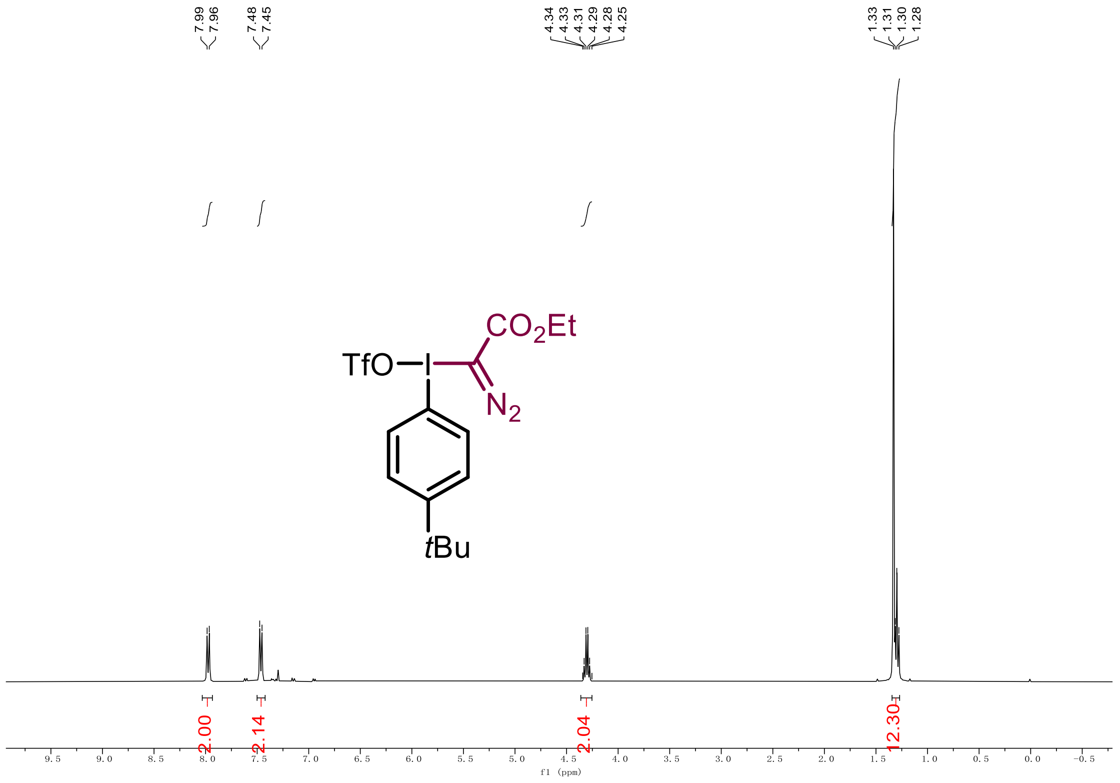
7.99
7.96
7.48
7.45
4.34
4.33
4.31
4.29
4.28
4.25
1.33
1.31
1.30
1.28
TfO
I
CO_2Et
N_2
tBu
2.00
2.14
2.04
12.30
f1 (ppm)

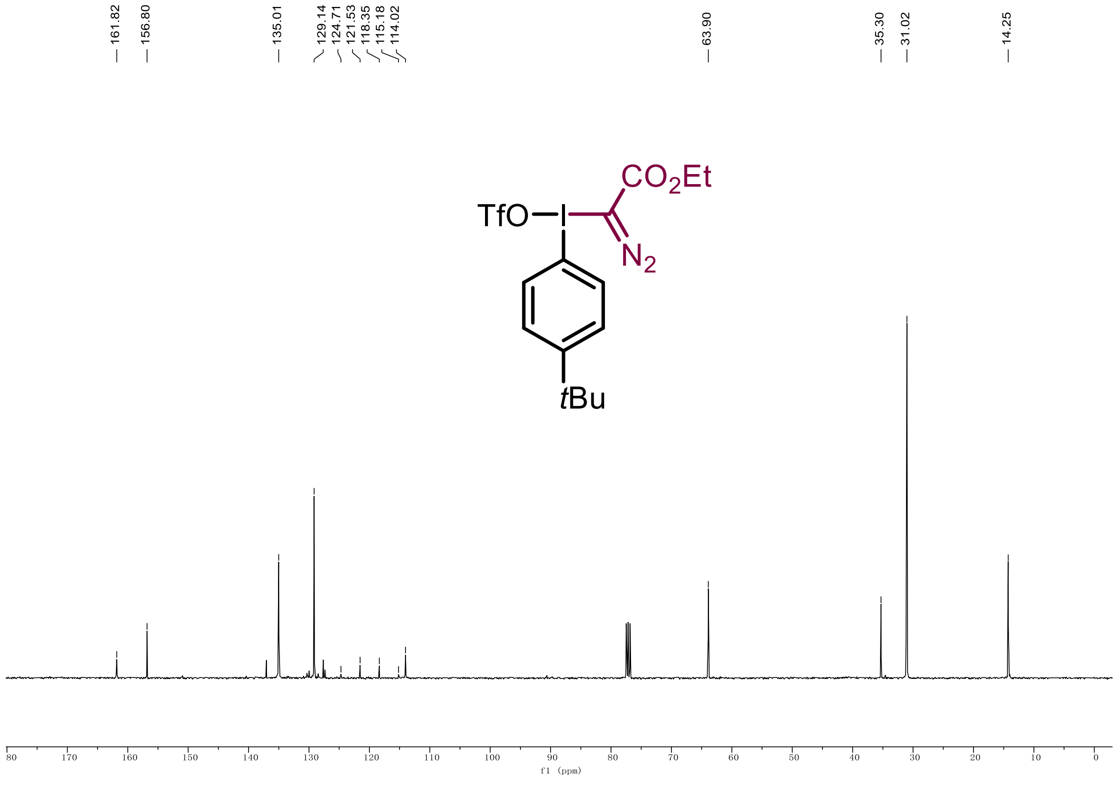
161.82
156.80
135.01
129.14
124.71
121.53
118.35
115.18
114.02
63.90
35.30
31.02
14.25
TfO
I
CO_2Et
N_2
tBu
f1 (ppm)

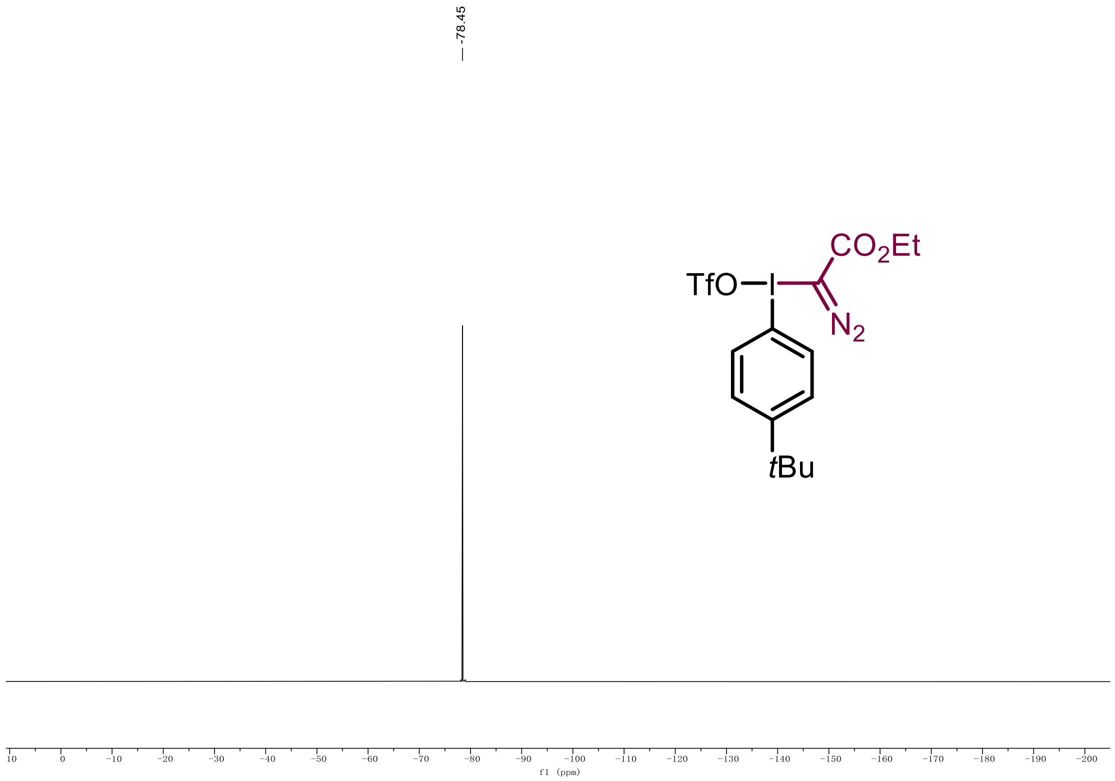
-78.45
TfO
I
CO2Et
N2
tBu
f1 (ppm)

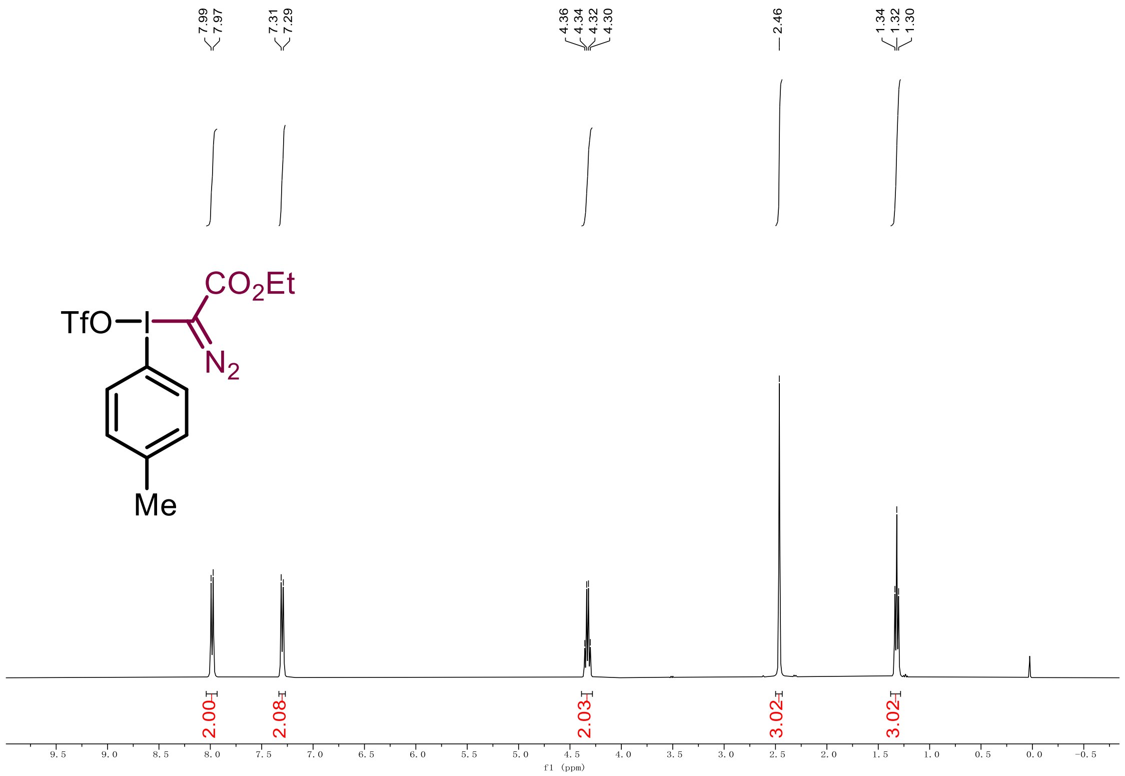
7.99
7.97
7.31
7.29
4.36
4.34
4.32
4.30
2.46
1.34
1.32
1.30
TfO
I
CO2Et
N2
Me
2.00
2.08
2.03
3.02
3.02
f1 (ppm)

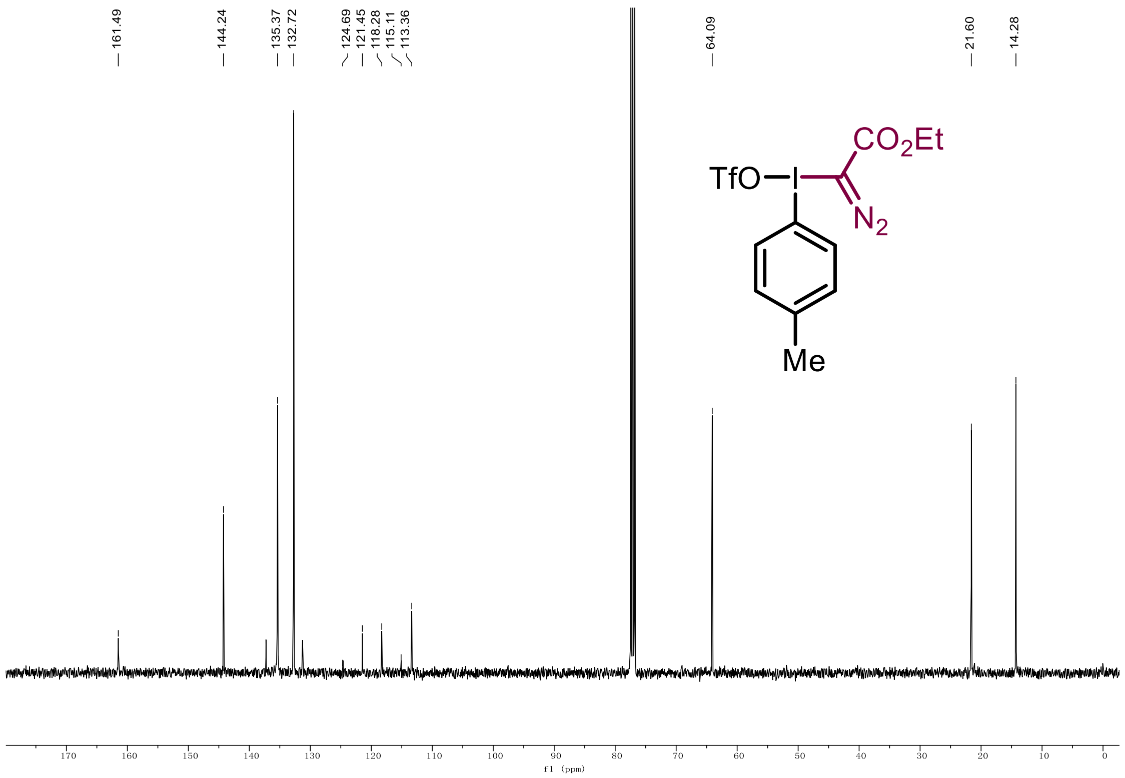
161.49
144.24
135.37
132.72
124.69
121.45
118.28
115.11
113.36
64.09
21.60
14.28
TfO
I
CO2Et
N2
Me
f1 (ppm)

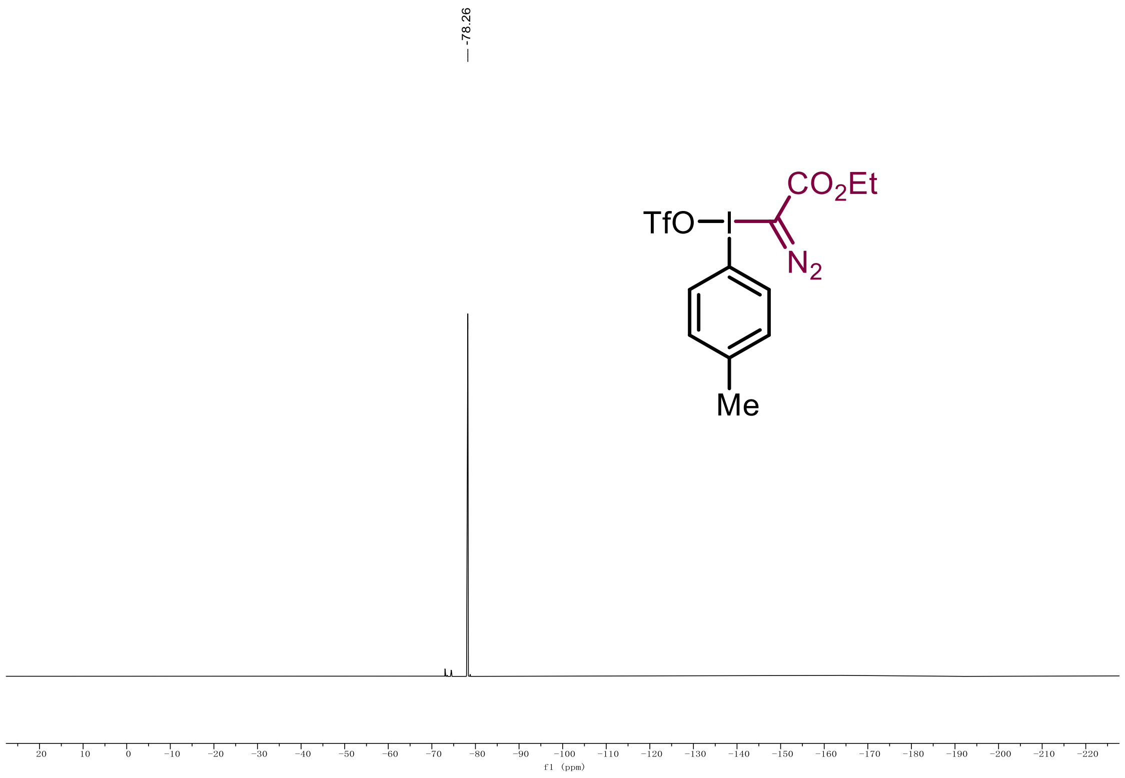
-78.26
TfO
I
CO2Et
N2
Me
f1 (ppm)

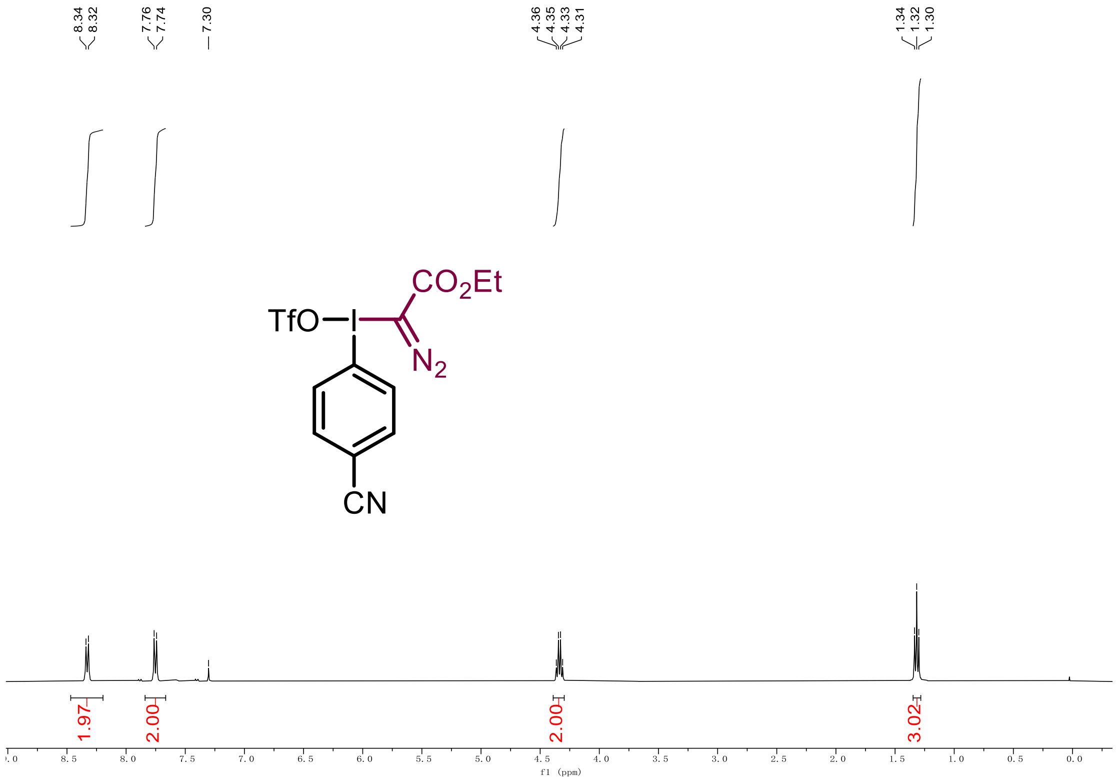
8.34
8.32
7.76
7.74
7.30
4.36
4.35
4.33
4.31
1.34
1.32
1.30
TfO
I
CO2Et
N2
CN
1.97
2.00
2.00
3.02
f1 (ppm)

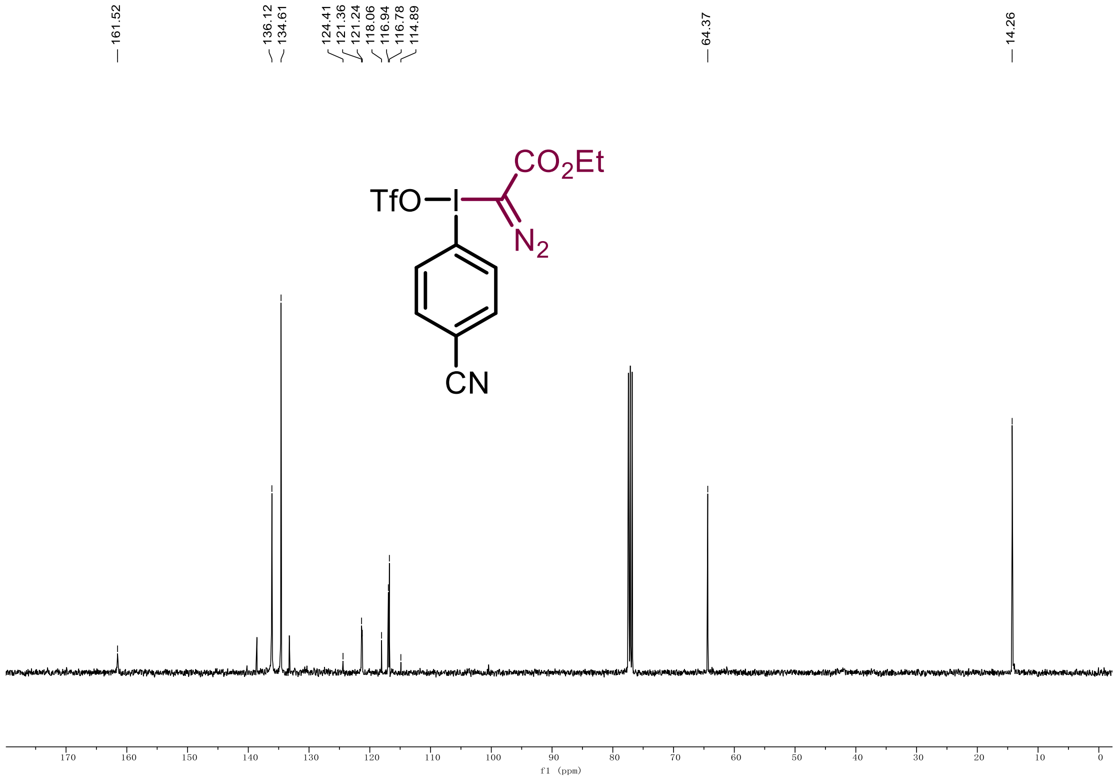
161.52
136.12
134.61
124.41
121.36
121.24
118.06
116.94
116.78
114.89
64.37
14.26
TfO
I
CO2Et
N2
CN
f1 (ppm)

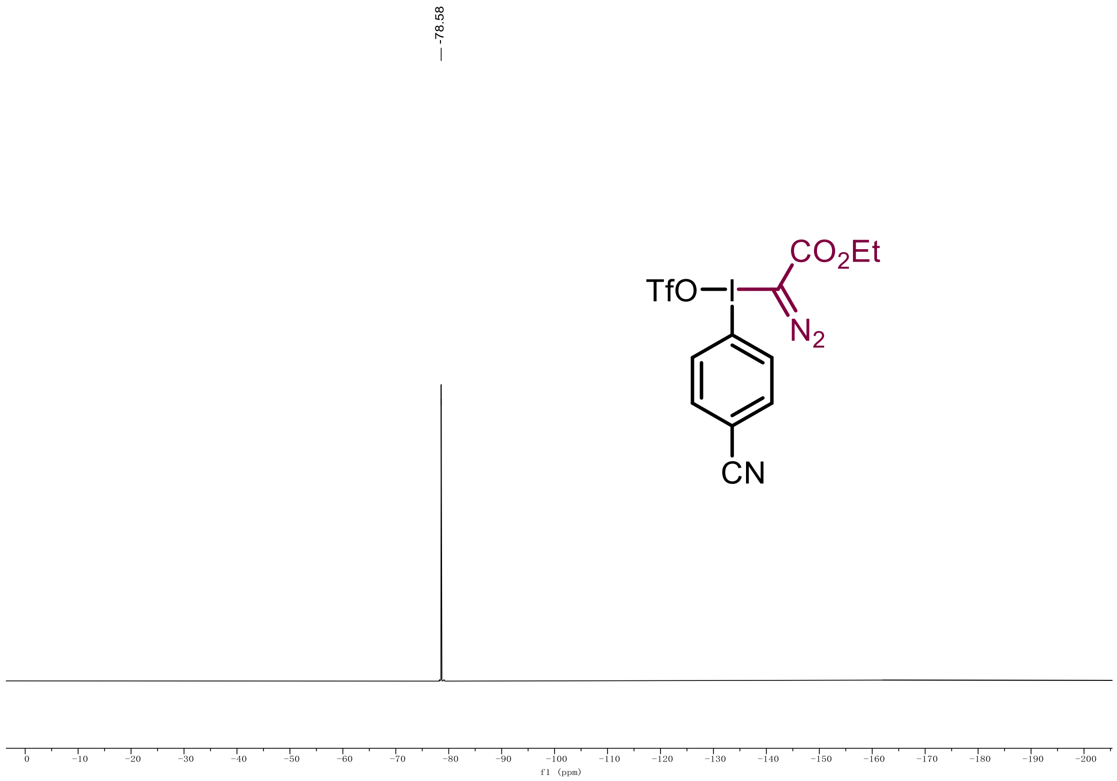
-78.58
TfO
I
CO2Et
N2
CN
f1 (ppm)

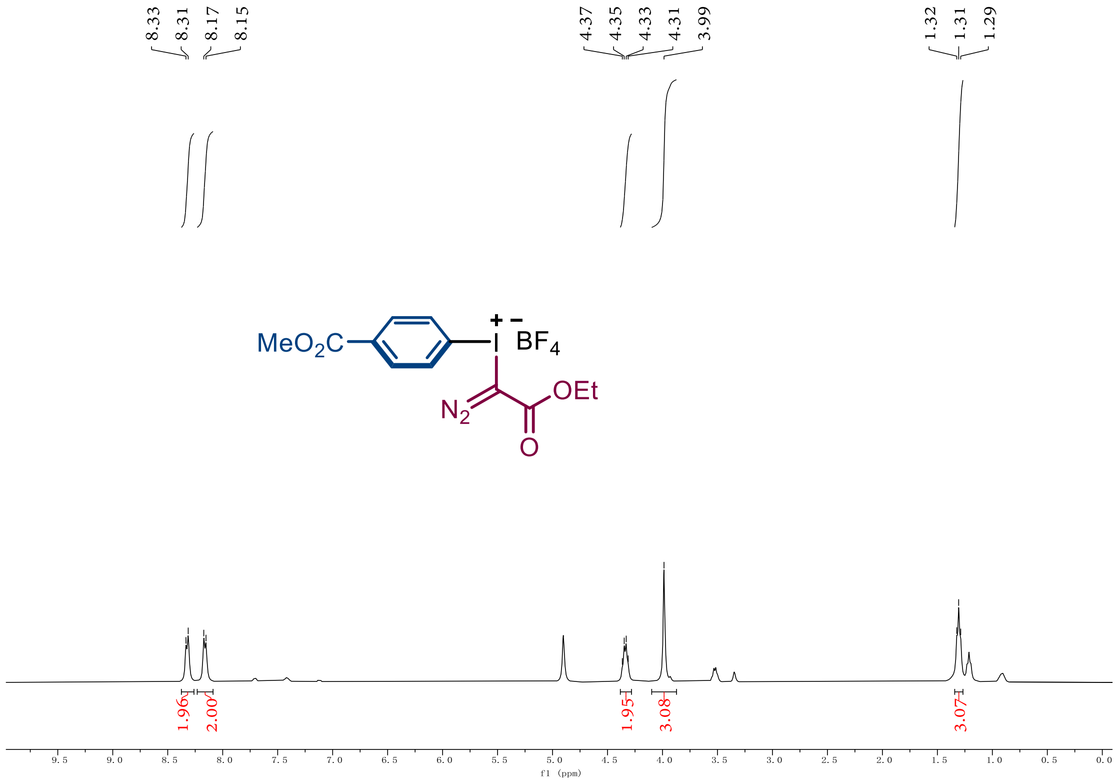
8.33
8.31
8.17
8.15
4.37
4.35
4.33
4.31
3.99
1.32
1.31
1.29
MeO2C
I
BF4
N2
OEt
O
1.96
2.00
1.95
3.08
3.07
f1 (ppm)

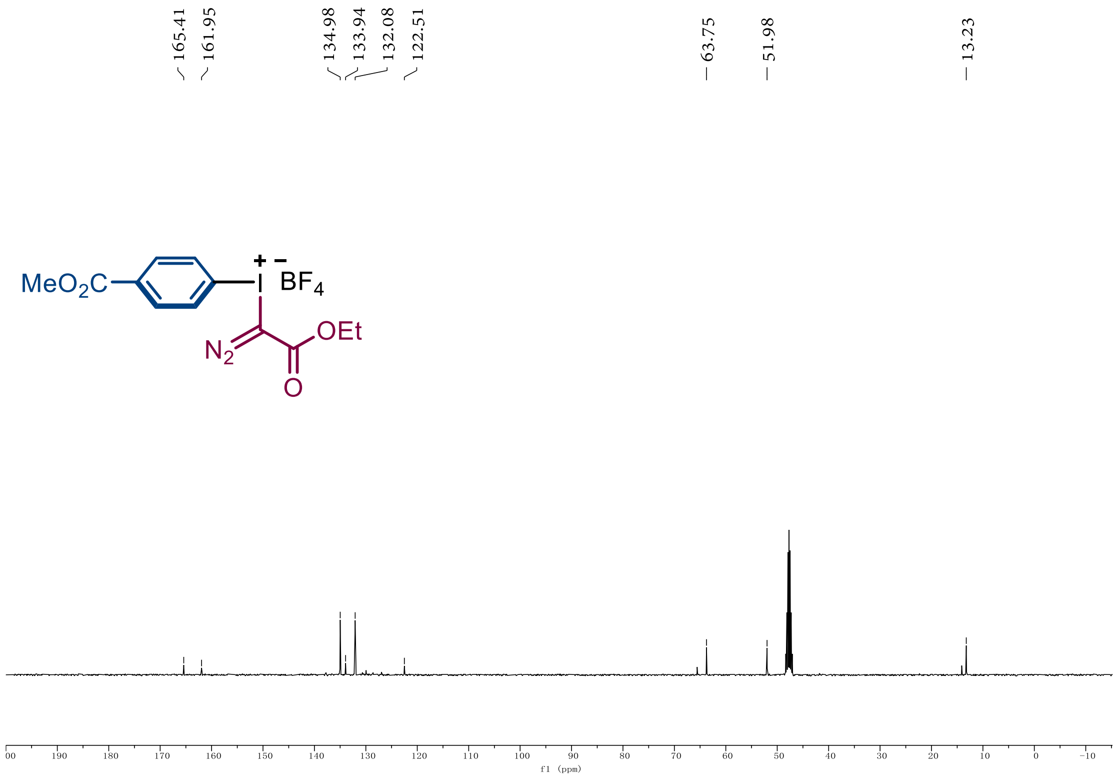
165.41
161.95
134.98
133.94
132.08
122.51
63.75
51.98
13.23
MeO2C
BF4
N2
OEt
O
f1 (ppm)

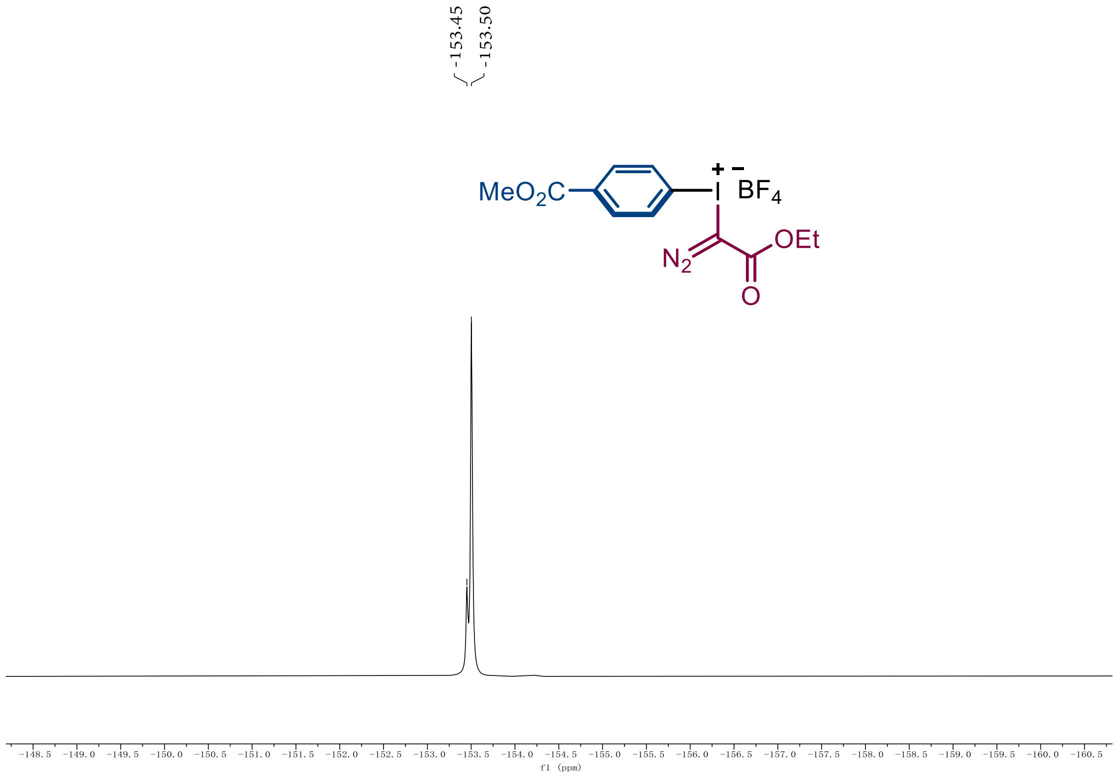
-153.45
-153.50
MeO2C
BF4
N2
OEt
O
f1 (ppm)

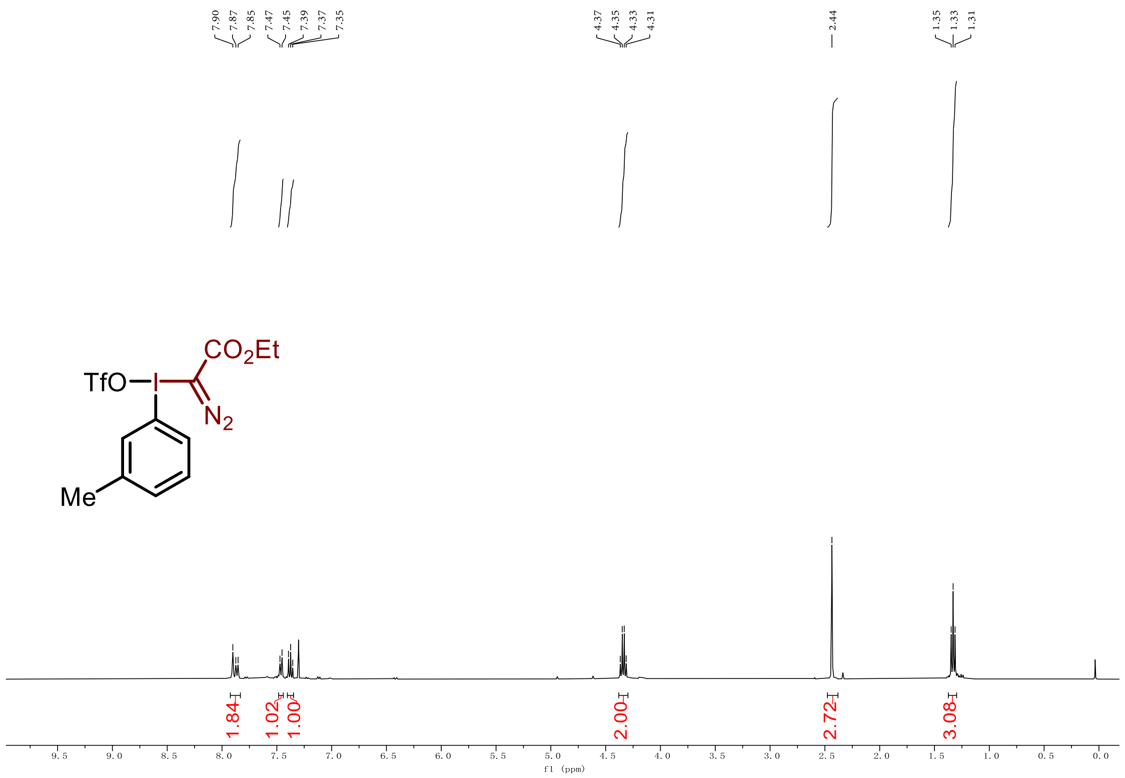
CO2Et
TfO
I
N2
Me
7.90
7.87
7.85
7.47
7.45
7.39
7.37
7.35
4.37
4.35
4.33
4.31
2.44
1.35
1.33
1.31
1.84
1.02
1.00
2.00
2.72
3.08
f1 (ppm)

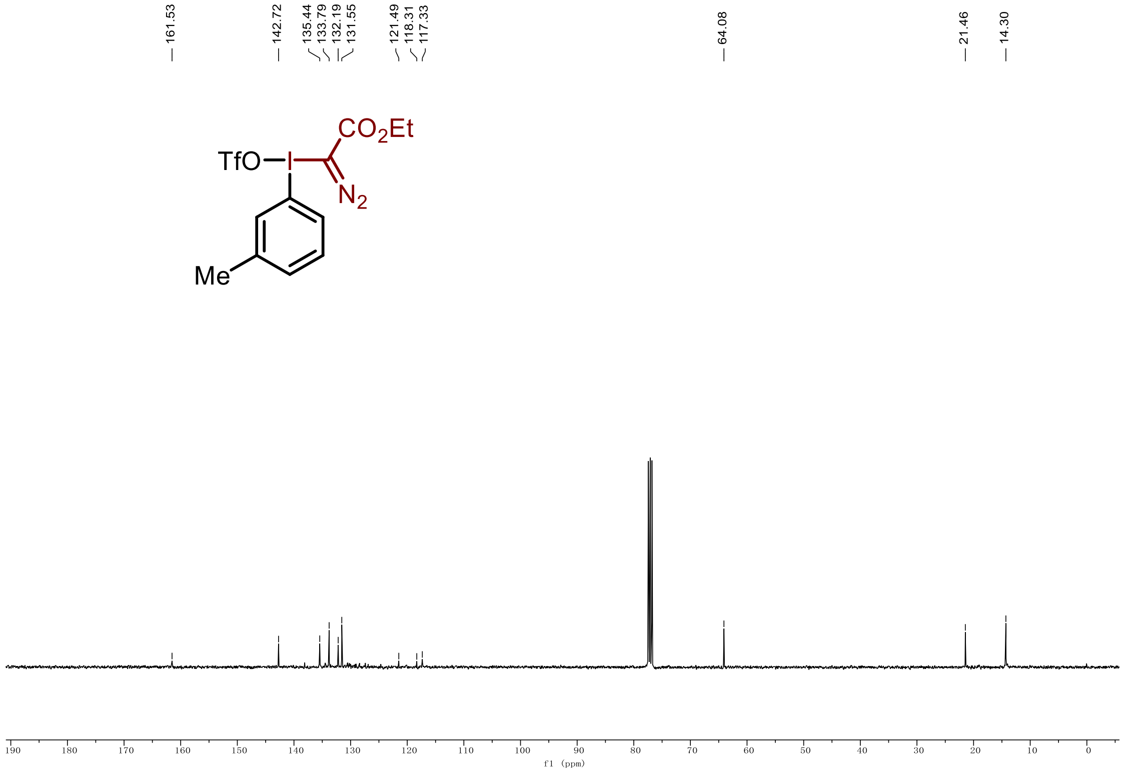
161.53
142.72
135.44
133.79
132.19
131.55
121.49
118.31
117.33
64.08
21.46
14.30
CO2Et
TfO
I
N2
Me
f1 (ppm)

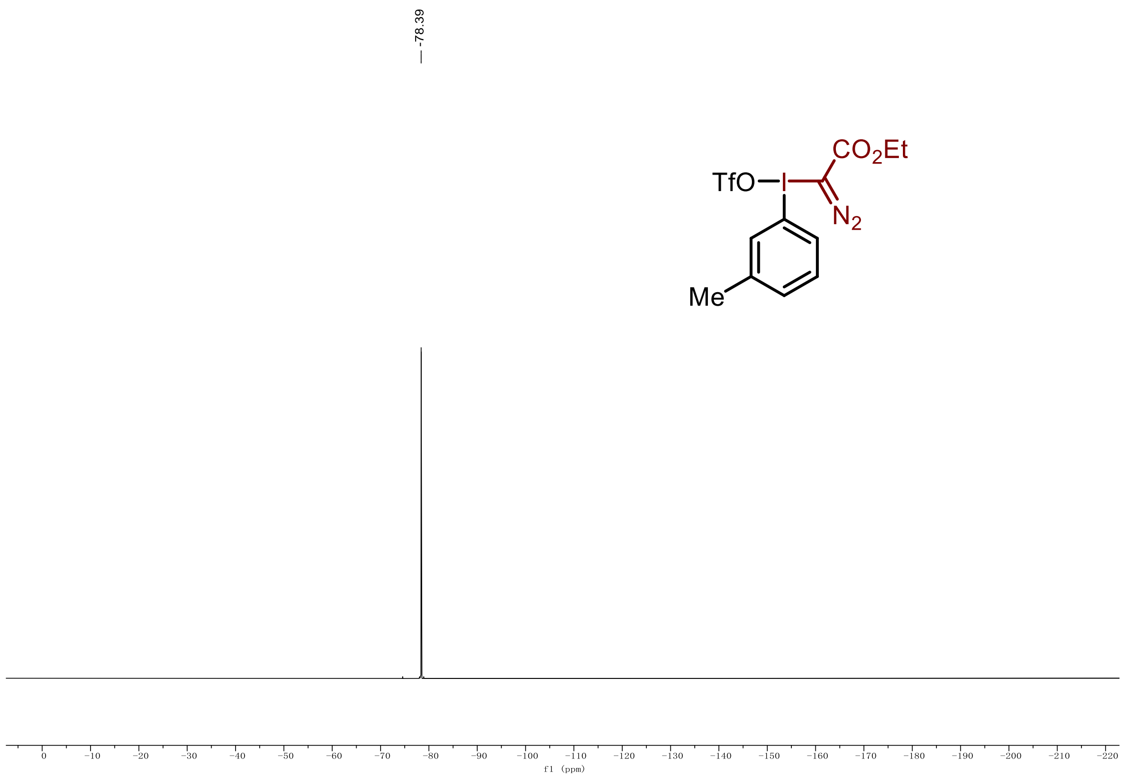
-78.39
CO2Et
TfO
I
N2
Me
f1 (ppm)

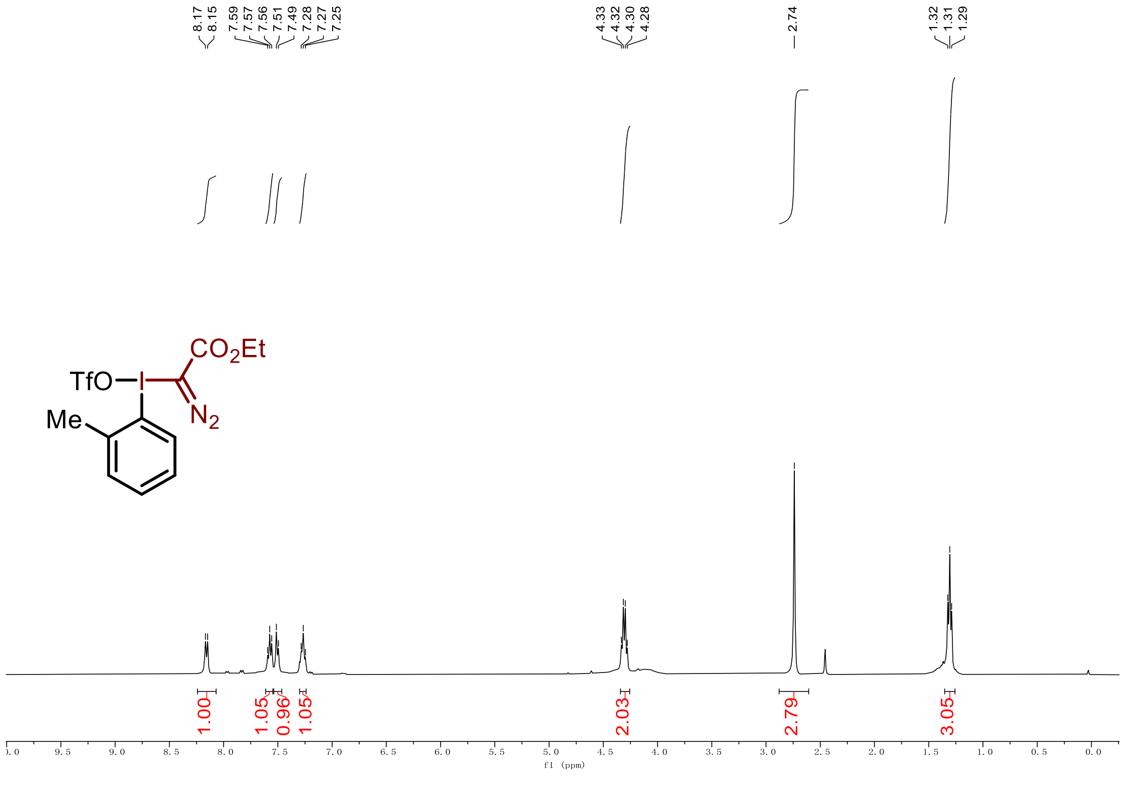
8.17
8.15
7.59
7.57
7.56
7.51
7.49
7.28
7.27
7.25
4.33
4.32
4.30
4.28
2.74
1.32
1.31
1.29
CO2Et
TfO
I
N2
Me
1.00
1.05
0.96
1.05
2.03
2.79
3.05
f1 (ppm)

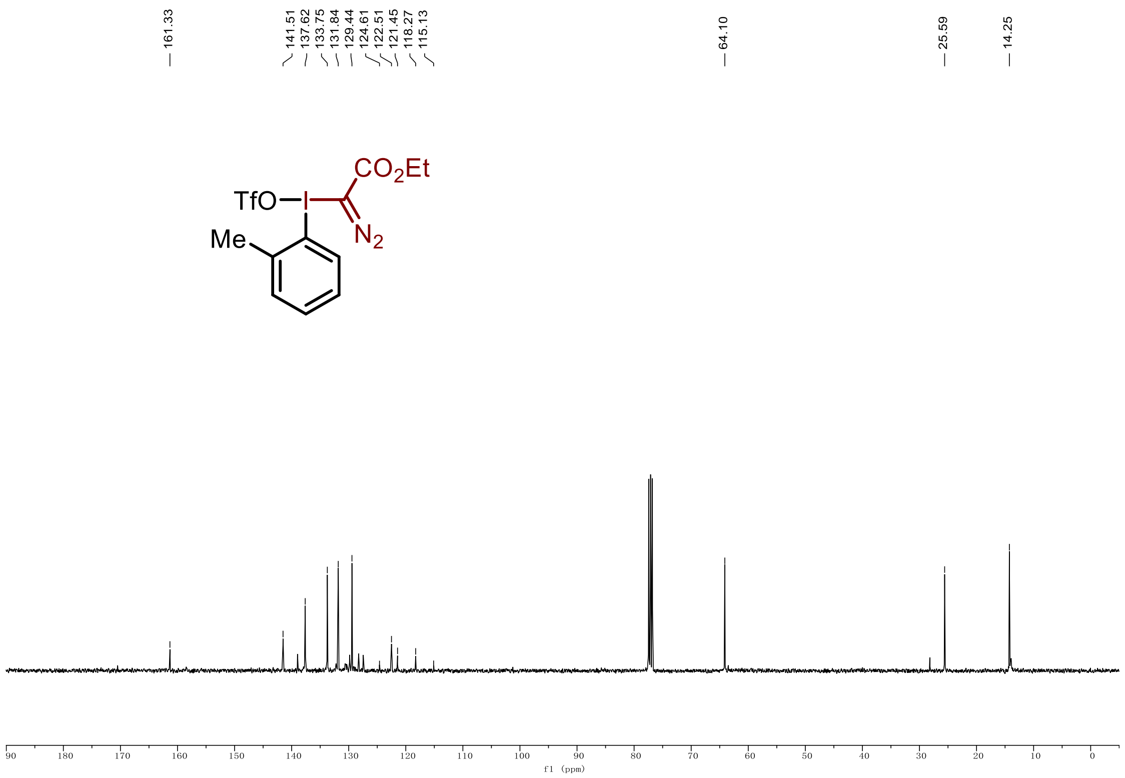

161.33
141.51
137.62
133.75
131.84
129.44
124.61
122.51
121.45
118.27
115.13
64.10
25.59
14.25
CO2Et
TfO
I
N2
Me
f1 (ppm)

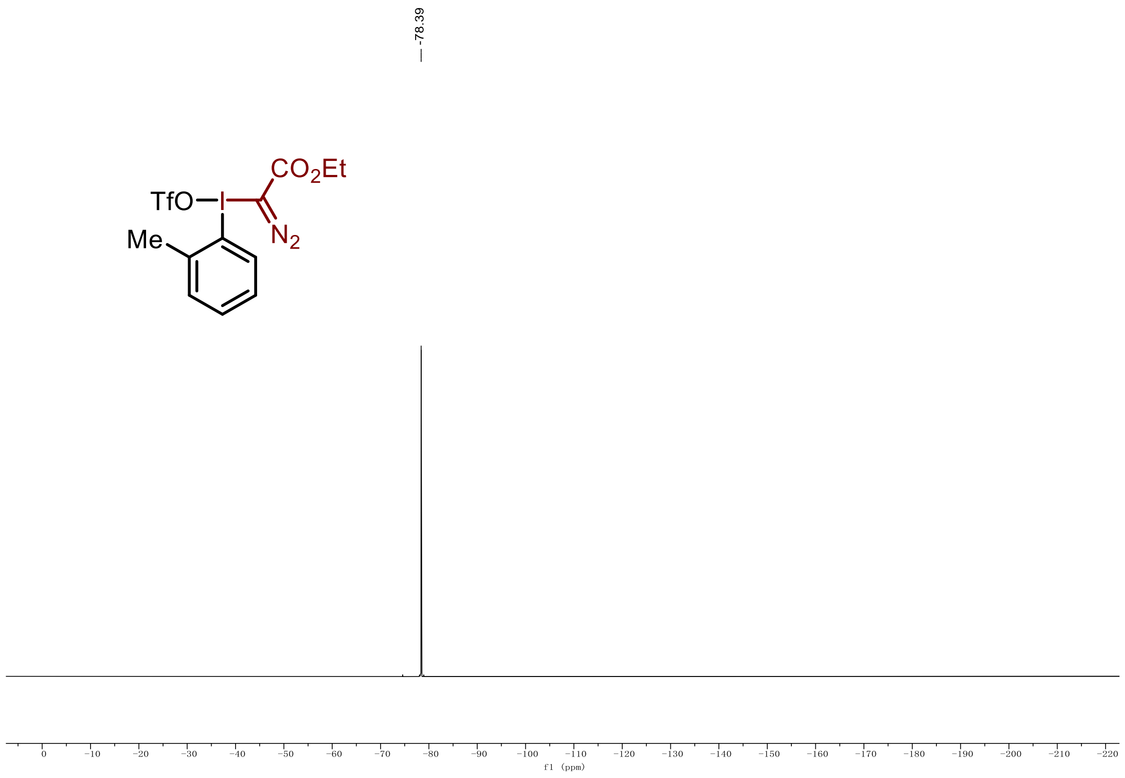

-78.39
CO2Et
TfO
I
N2
Me
f1 (ppm)

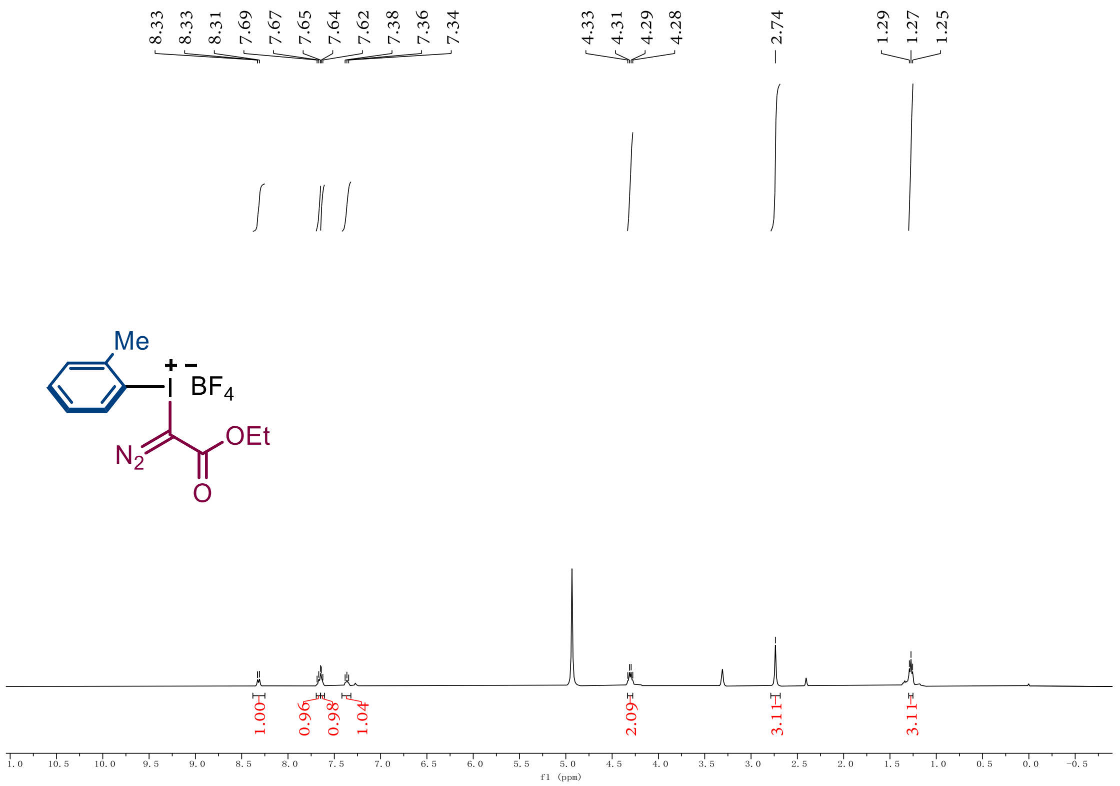
8.33
8.33
8.31
7.69
7.67
7.65
7.64
7.62
7.38
7.36
7.34
4.33
4.31
4.29
4.28
2.74
1.29
1.27
1.25
Me
BF4
OEt
N2
O
1.00
0.96
0.98
1.04
2.09
3.11
3.11
f1 (ppm)

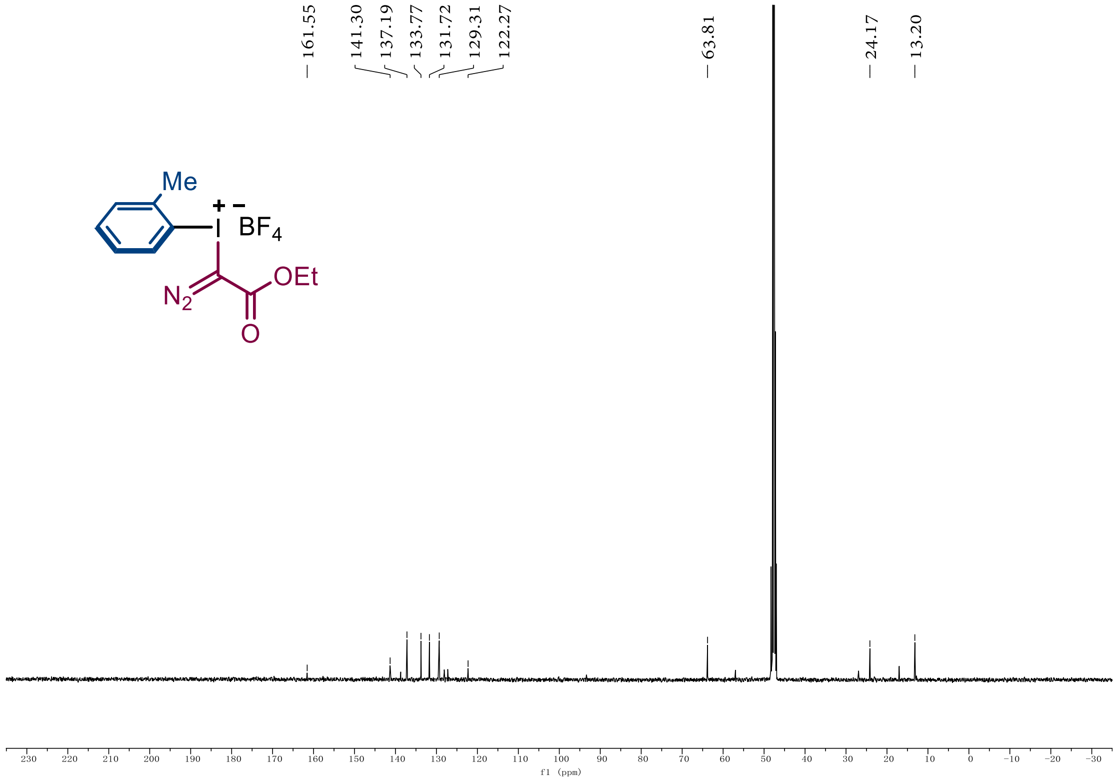
161.55
141.30
137.19
133.77
131.72
129.31
122.27
63.81
24.17
13.20
Me
BF4
OEt
N2
O
f1 (ppm)

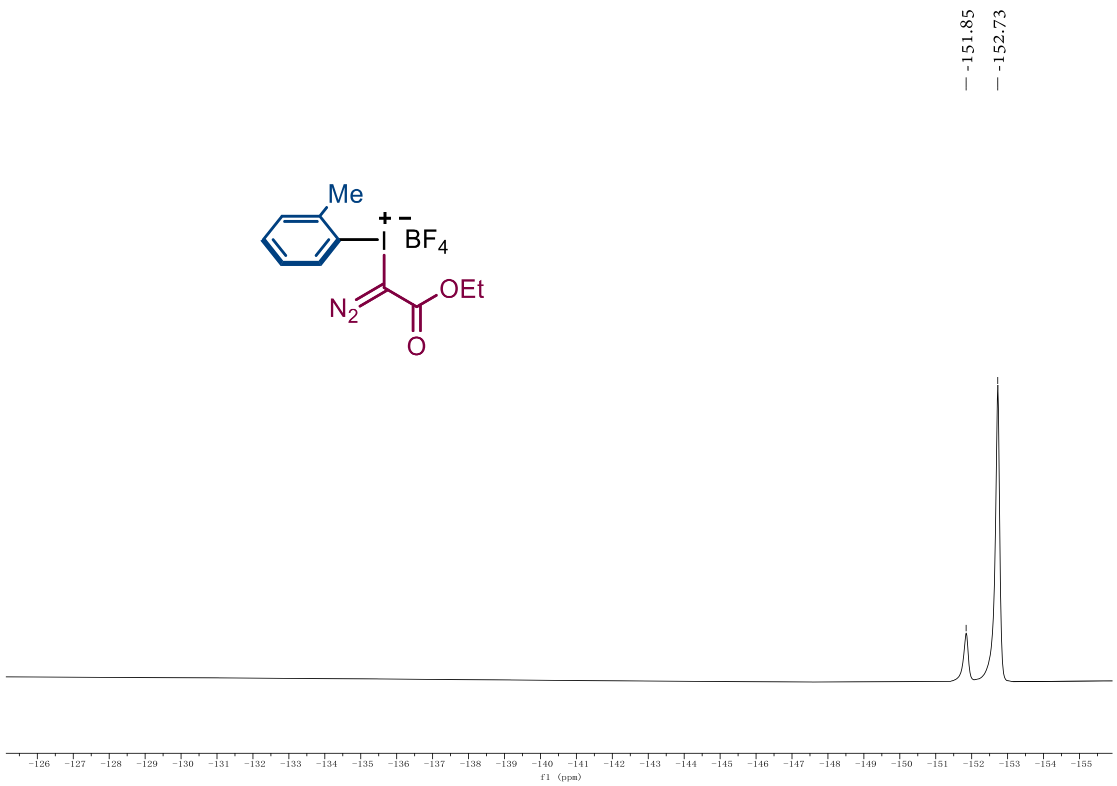
-151.85
-152.73
Me
BF4
N2
OEt
O
f1 (ppm)

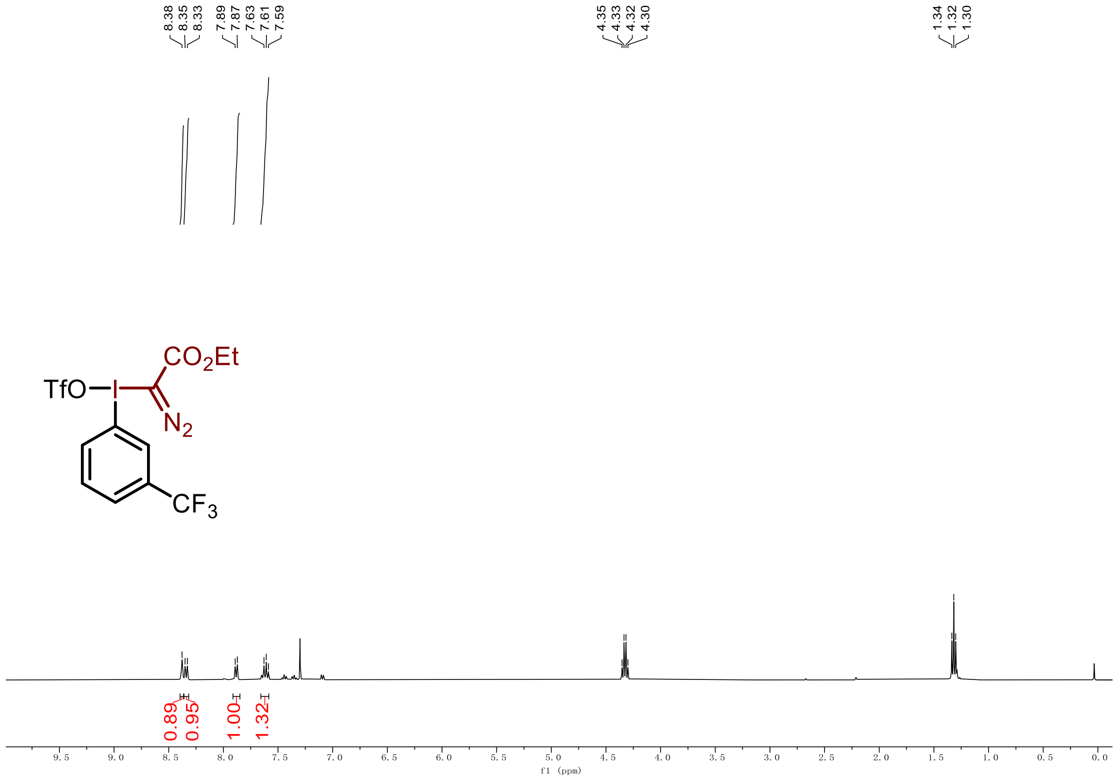
8.38
8.35
8.33
7.89
7.87
7.63
7.61
7.59
4.35
4.33
4.32
4.30
1.34
1.32
1.30
TfO
CO2Et
N2
CF3
0.89
0.95
1.00
1.32
f1 (ppm)

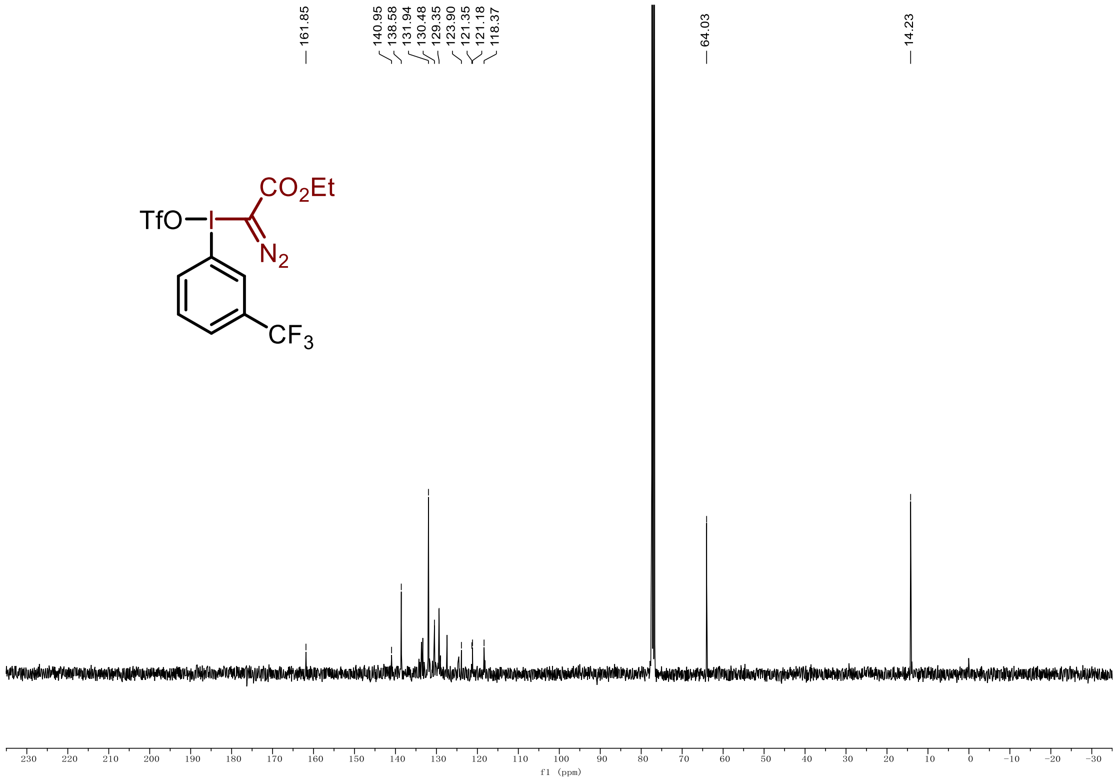

161.85
140.95
138.58
131.94
130.48
129.35
123.90
121.35
121.18
118.37
64.03
14.23
TfO
I
CO2Et
N2
CF3
f1 (ppm)

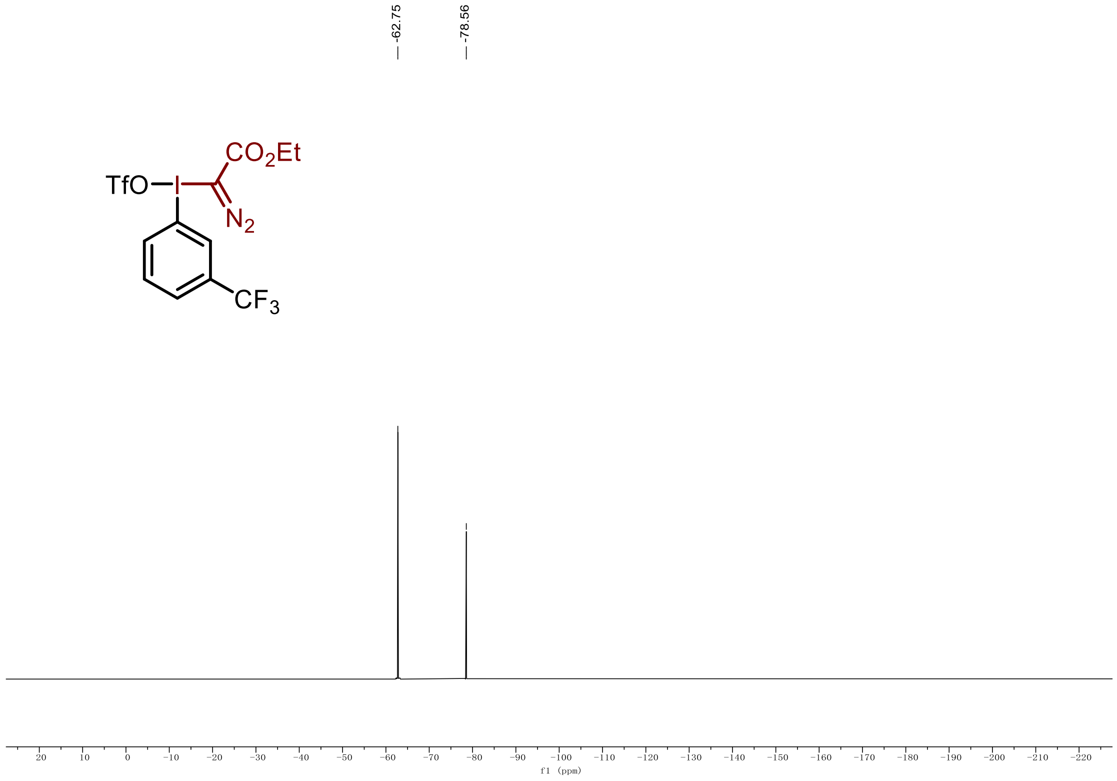

-62.75
-78.56
TfO
I
CO2Et
N2
CF3
f1 (ppm)

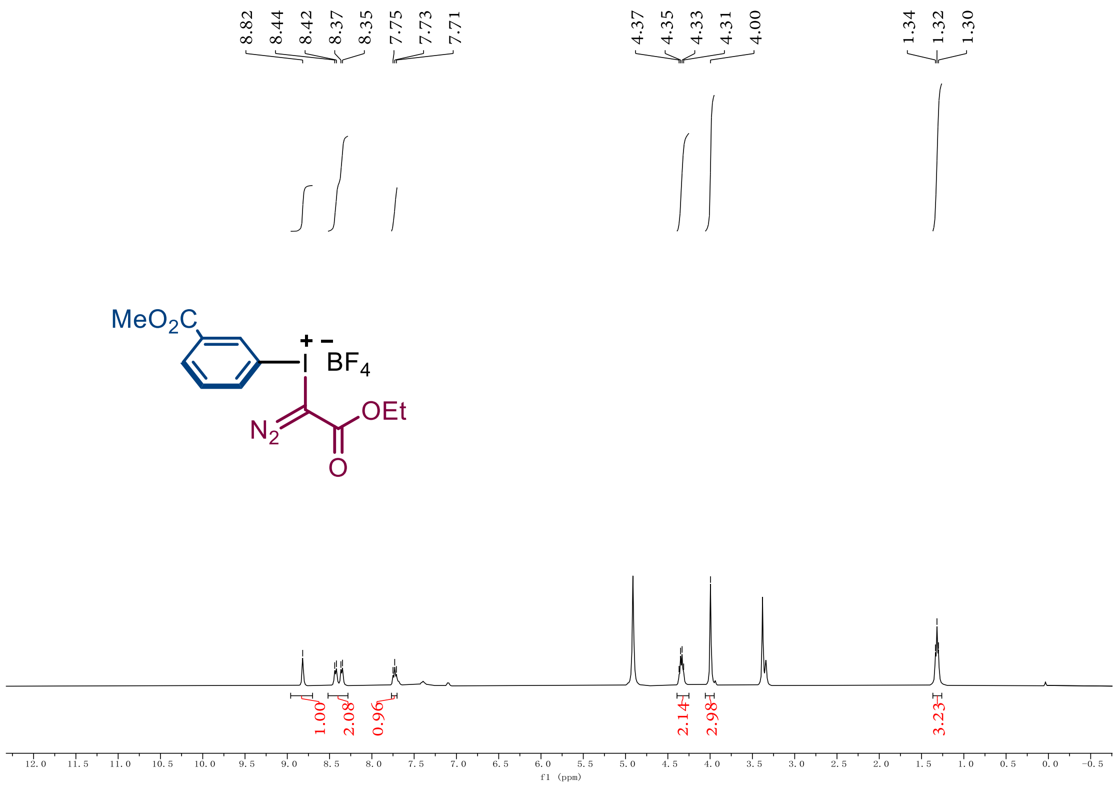

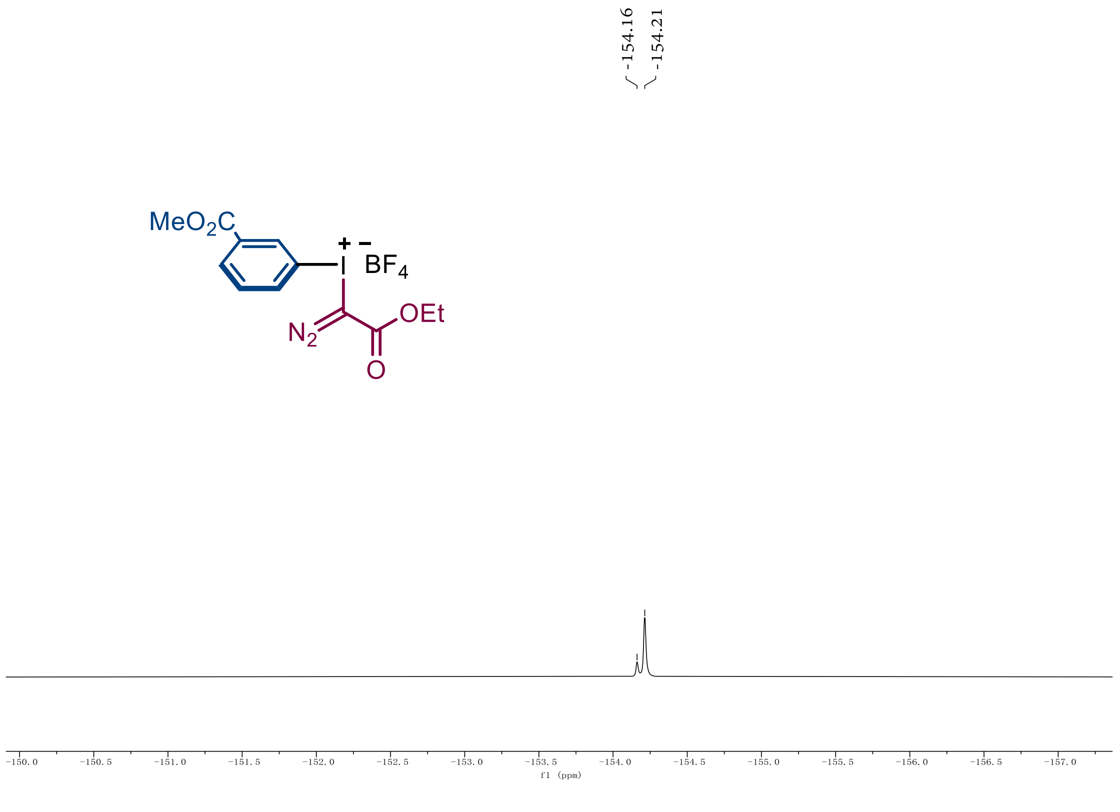

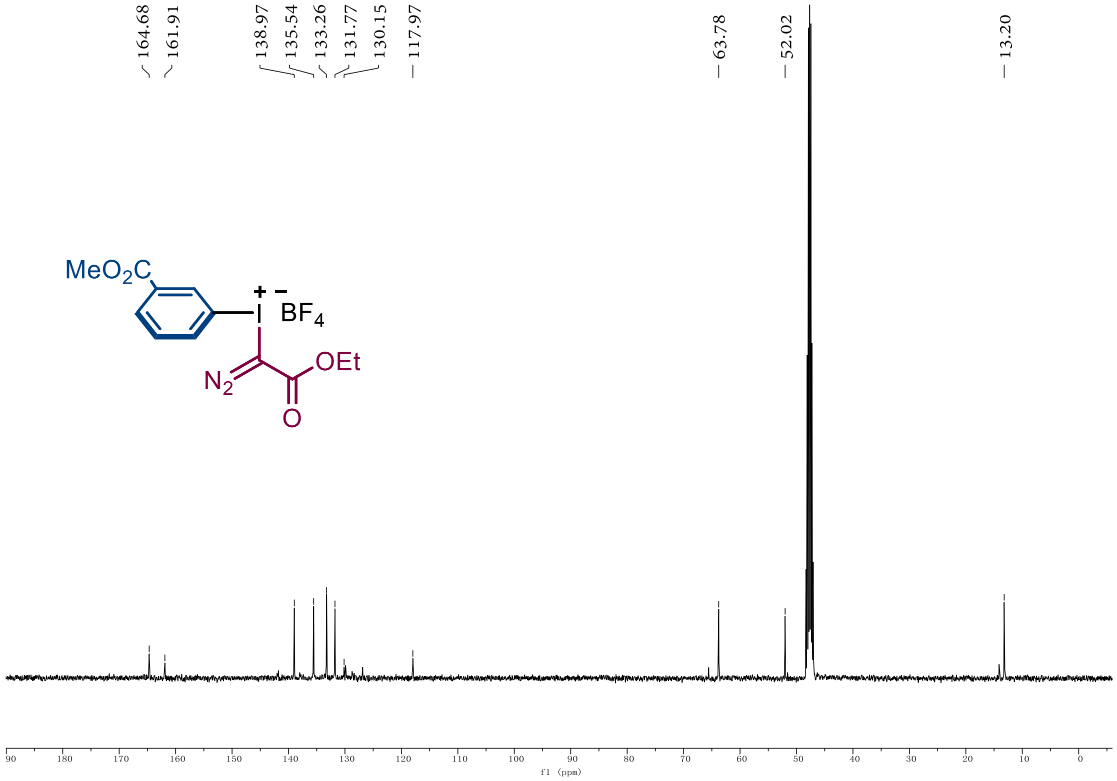
164.68
161.91
138.97
135.54
133.26
131.77
130.15
117.97
63.78
52.02
13.20
MeO2C
BF4
N2
OEt
O
f1 (ppm)

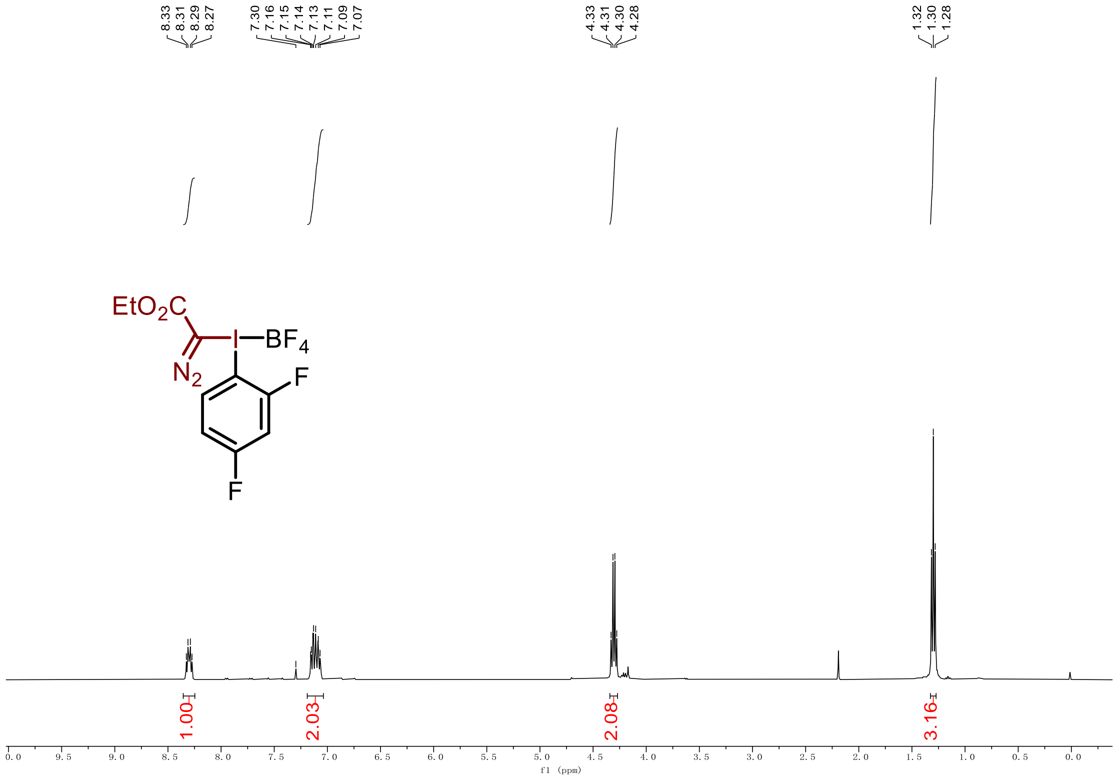
8.33
8.31
8.29
8.27
7.30
7.16
7.15
7.14
7.13
7.11
7.09
7.07
4.33
4.31
4.30
4.28
1.32
1.30
1.28
EtO2C
BF4
N2
F
F
1.00
2.03
2.08
3.16
f1 (ppm)

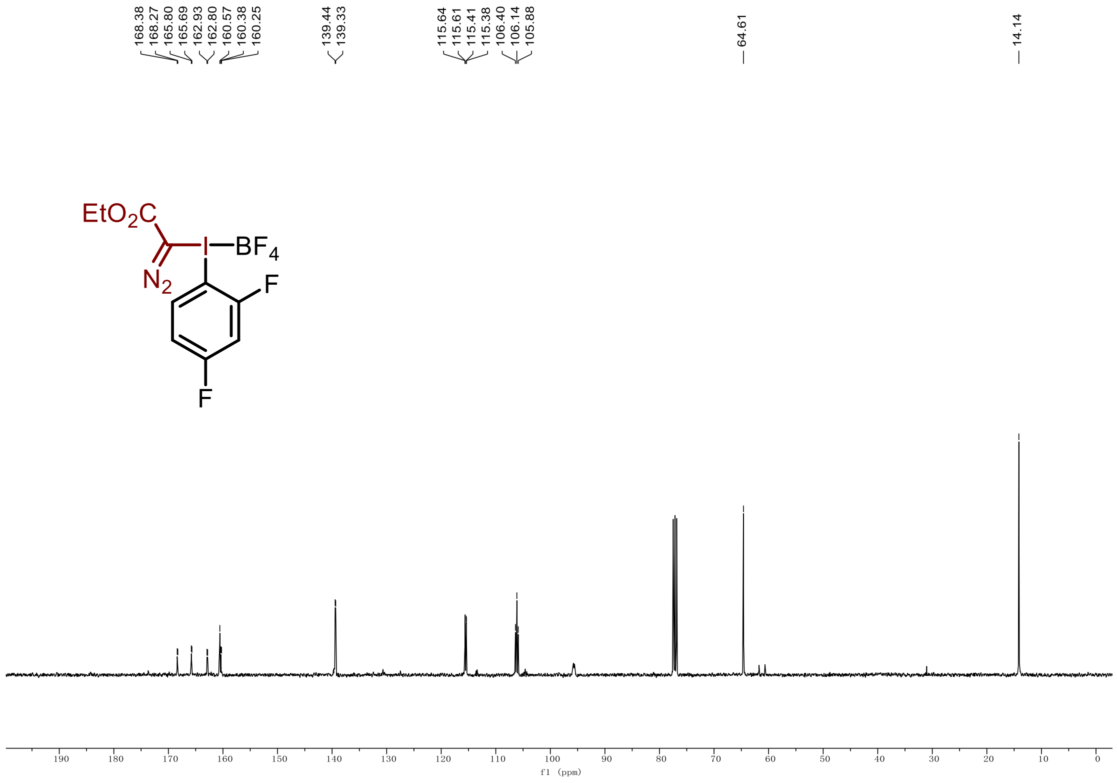
168.38
168.27
165.80
165.69
162.93
162.80
160.57
160.38
160.25
139.44
139.33
115.64
115.61
115.41
115.38
106.40
106.14
105.88
64.61
14.14
EtO2C
I
BF4
N2
F
F
190
180
170
160
150
140
130
120
110
100
90
80
70
60
50
40
30
20
10
0
f1 (ppm)

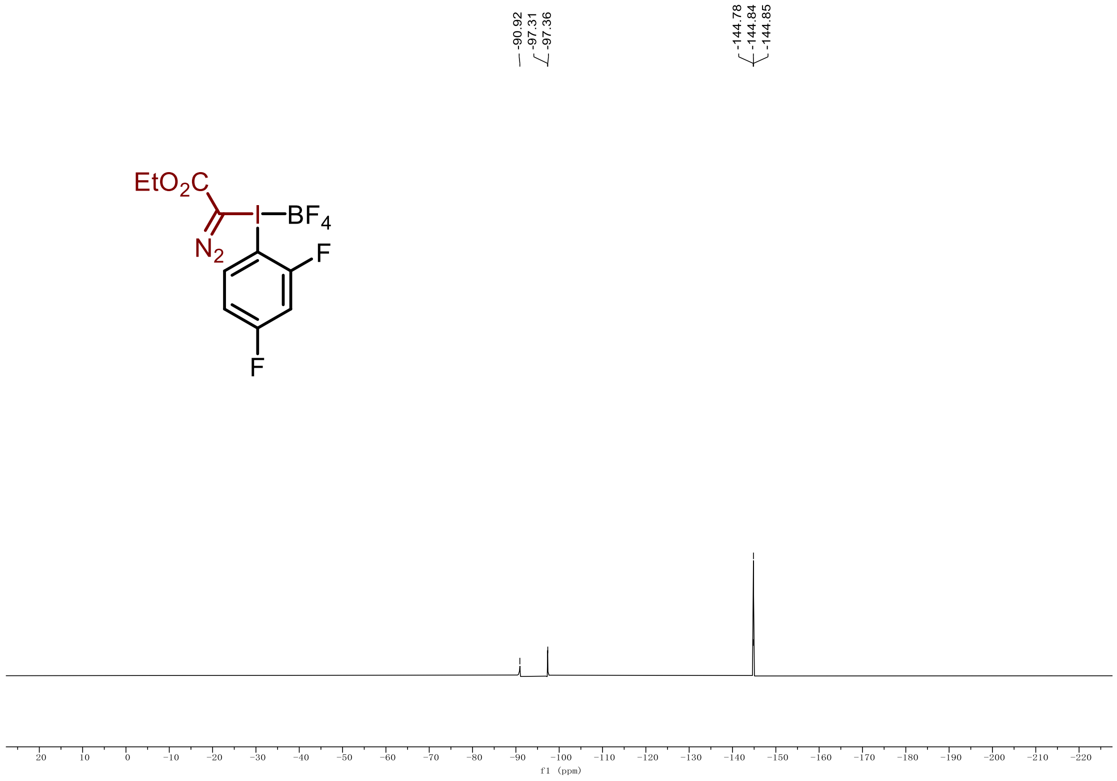
-90.92
-97.31
-97.36
-144.78
-144.84
-144.85
EtO2C
I
BF4
N2
F
F
20
10
0
−10
−20
−30
−40
−50
−60
−70
−80
−90
−100
−110
−120
−130
−140
−150
−160
−170
−180
−190
−200
−210
−220
f1 (ppm)

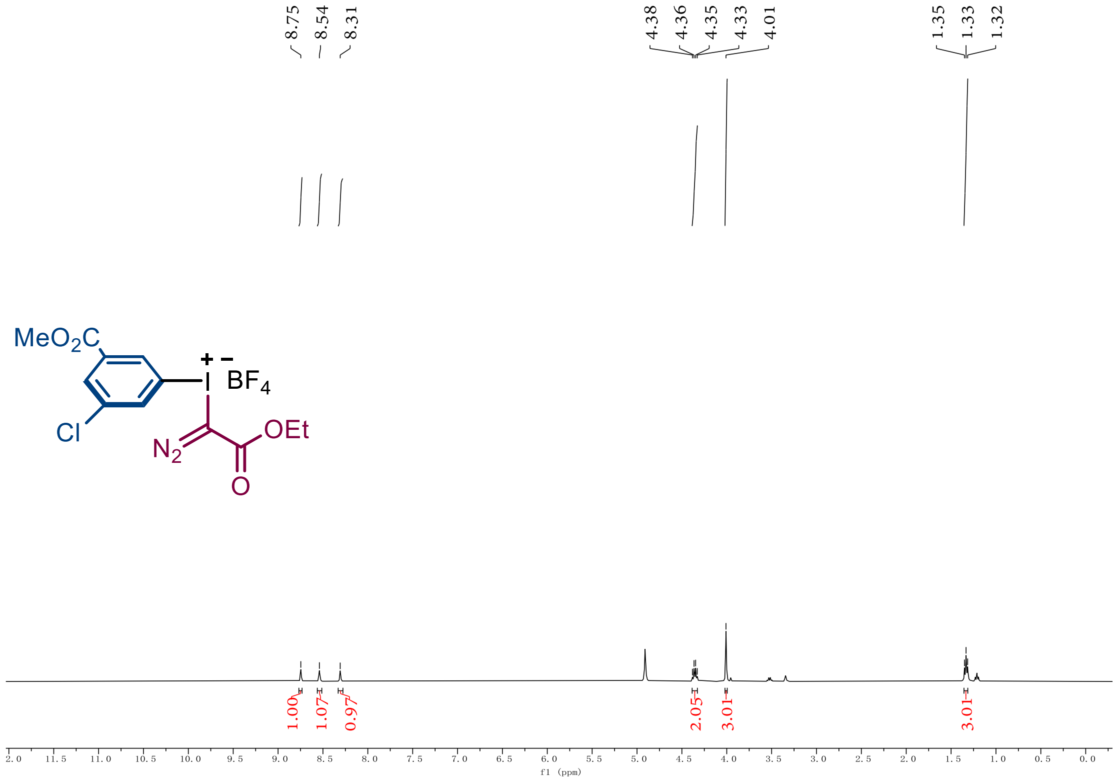
8.75
8.54
8.31
4.38
4.36
4.35
4.33
4.01
1.35
1.33
1.32
MeO2C
BF4
Cl
N2
OEt
O
1.00
1.07
0.97
2.05
3.01
3.01
f1 (ppm)

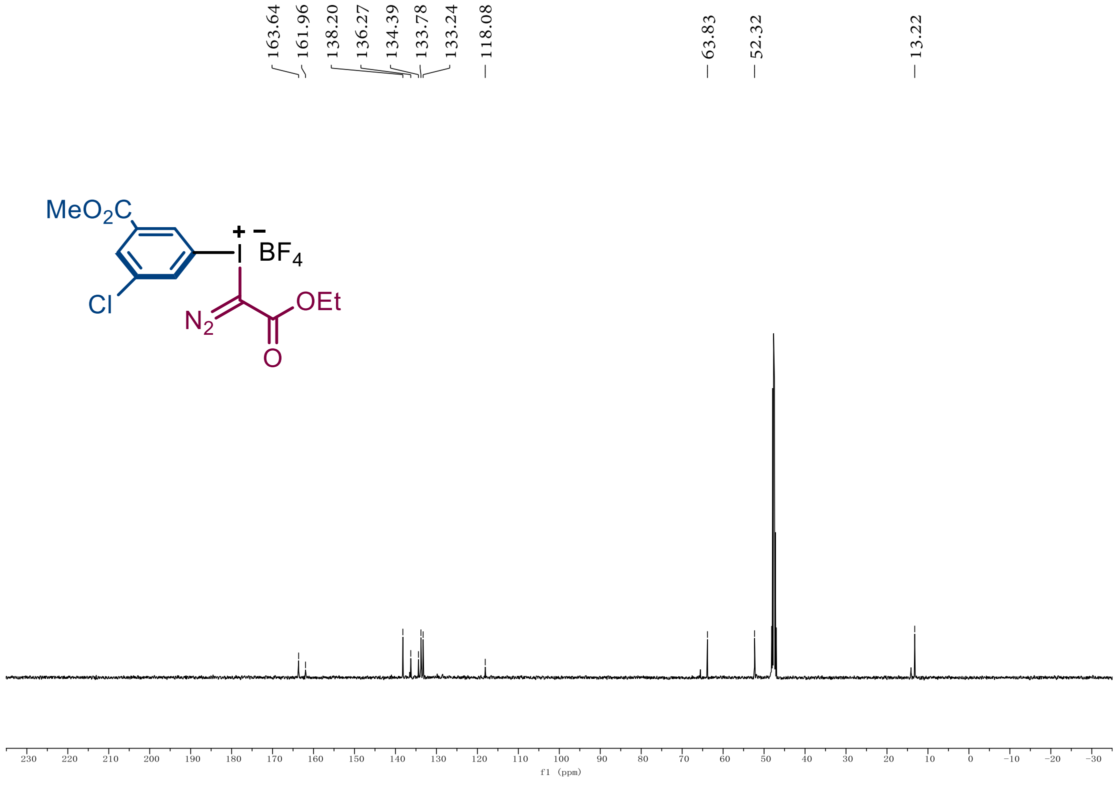
163.64
161.96
138.20
136.27
134.39
133.78
133.24
118.08
63.83
52.32
13.22
MeO2C
BF4
Cl
N2
OEt
O
f1 (ppm)

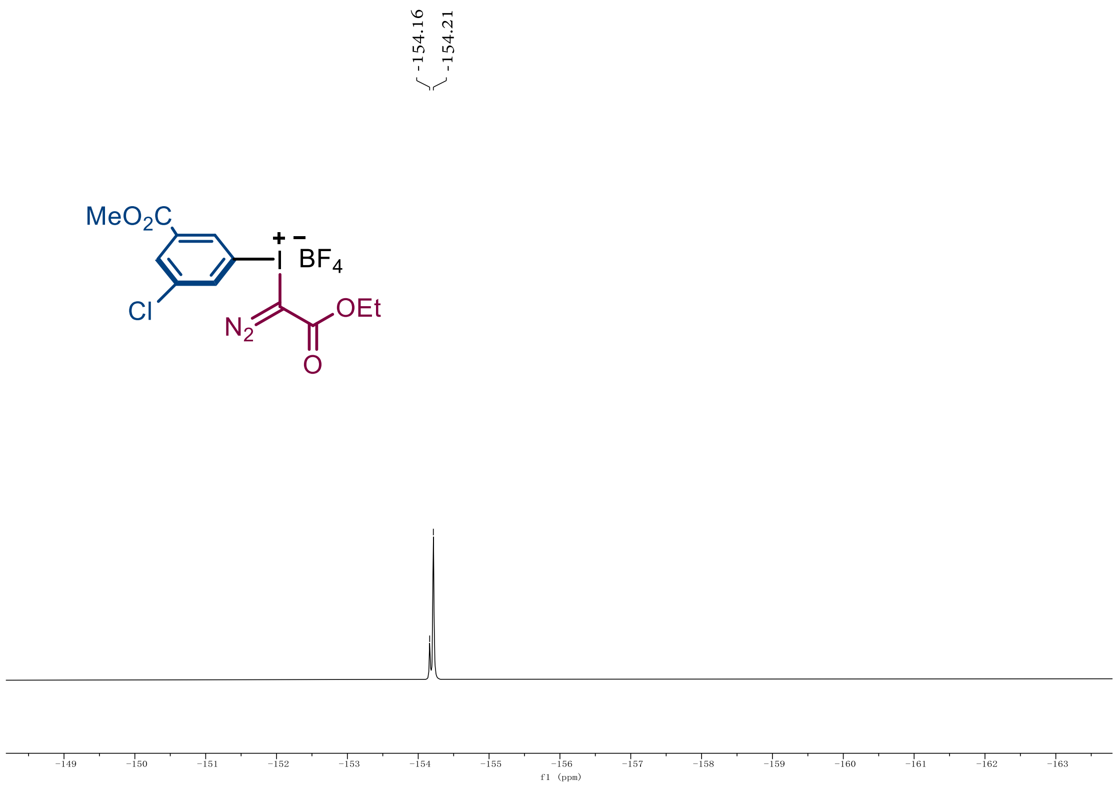

-154.16
-154.21
MeO2C
Cl
I
BF4
N2
OEt
O
-149
-150
-151
-152
-153
-154
-155
-156
-157
-158
-159
-160
-161
-162
-163
f1 (ppm)

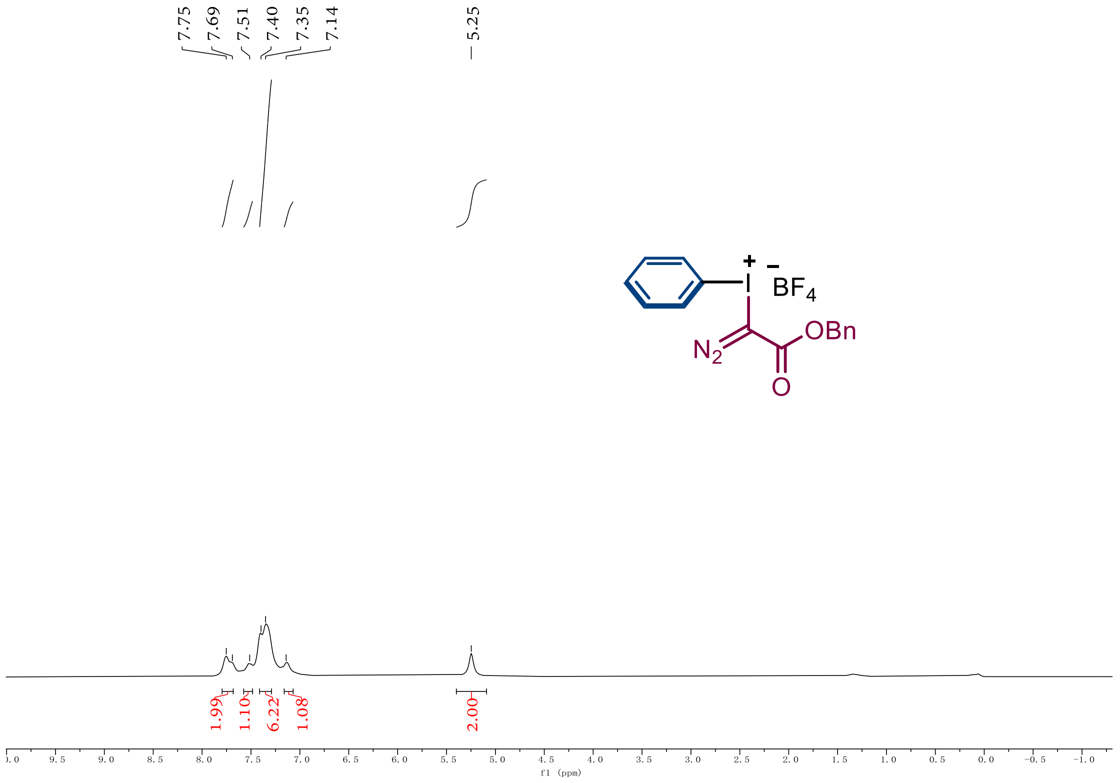

7.75
7.69
7.51
7.40
7.35
7.14
5.25
I
BF4
N2
OBn
O
1.99
1.10
6.22
1.08
2.00
9.5
9.0
8.5
8.0
7.5
7.0
6.5
6.0
5.5
5.0
4.5
4.0
3.5
3.0
2.5
2.0
1.5
1.0
0.5
0.0
-0.5
-1.0
f1 (ppm)

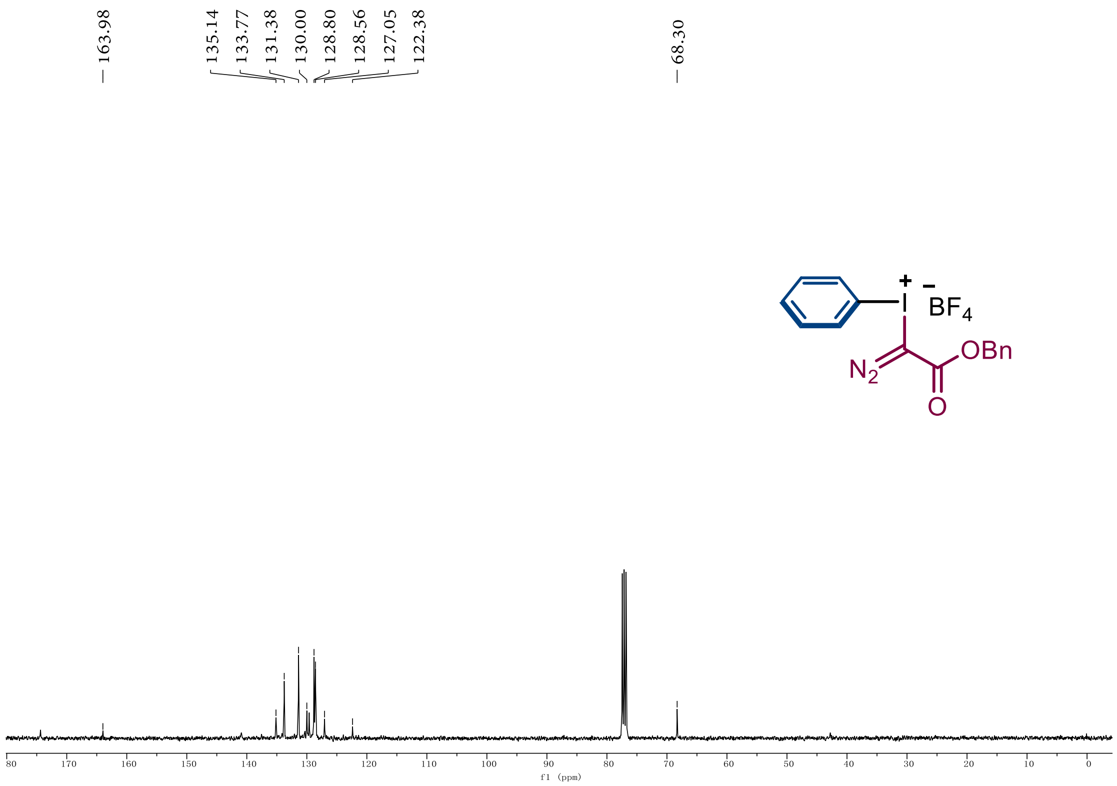
163.98
135.14
133.77
131.38
130.00
128.80
128.56
127.05
122.38
68.30
BF4
N2
OBn
O
180 170 160 150 140 130 120 110 100 90 80 70 60 50 40 30 20 10 0
f1 (ppm)

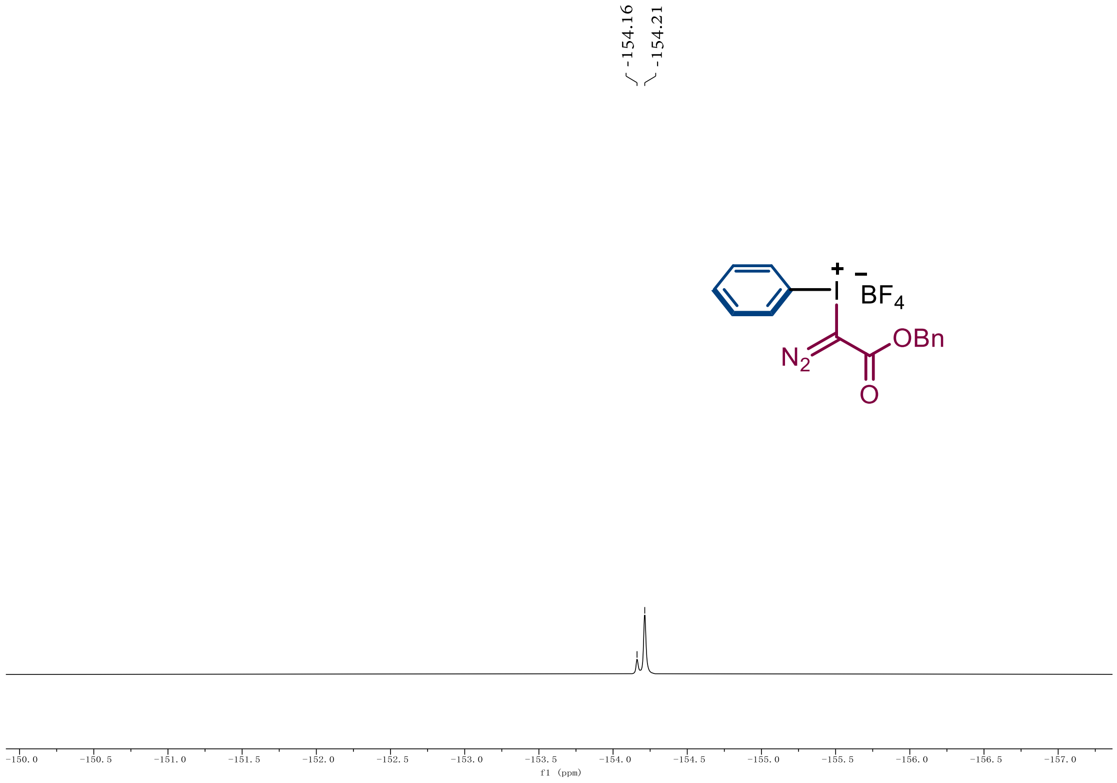
-154.16
-154.21
BF4
N2
OBn
O
-150.0 -150.5 -151.0 -151.5 -152.0 -152.5 -153.0 -153.5 -154.0 -154.5 -155.0 -155.5 -156.0 -156.5 -157.0
f1 (ppm)

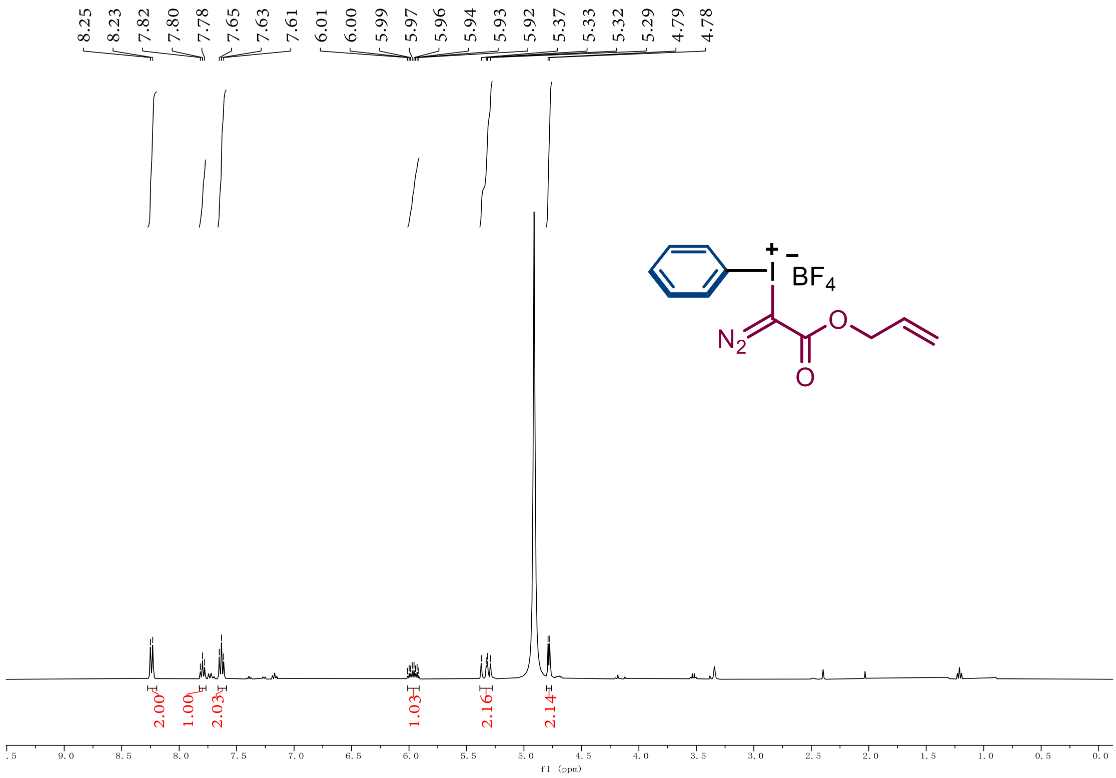
8.25
8.23
7.82
7.80
7.78
7.65
7.63
7.61
6.01
6.00
5.99
5.97
5.96
5.94
5.93
5.92
5.37
5.33
5.32
5.29
4.79
4.78
BF4
N2
O
O
2.00
1.00
2.03
1.03
2.16
2.14
f1 (ppm)

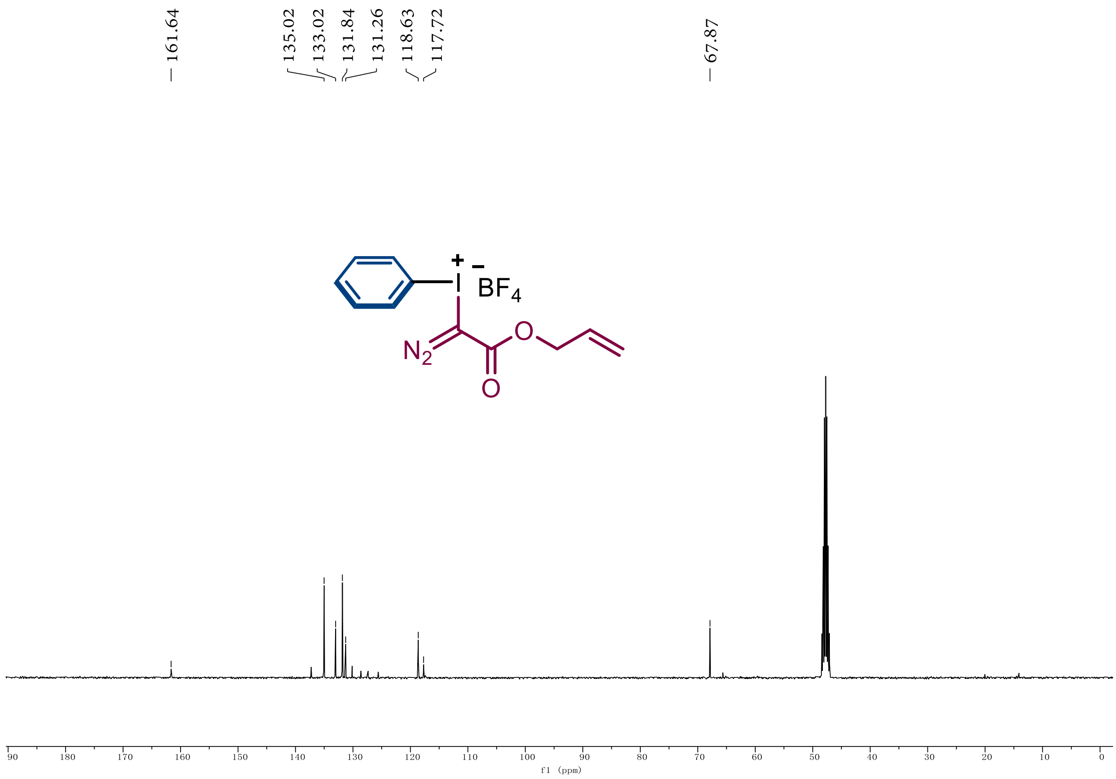
161.64
135.02
133.02
131.84
131.26
118.63
117.72
67.87
BF4
N2
O
O
f1 (ppm)

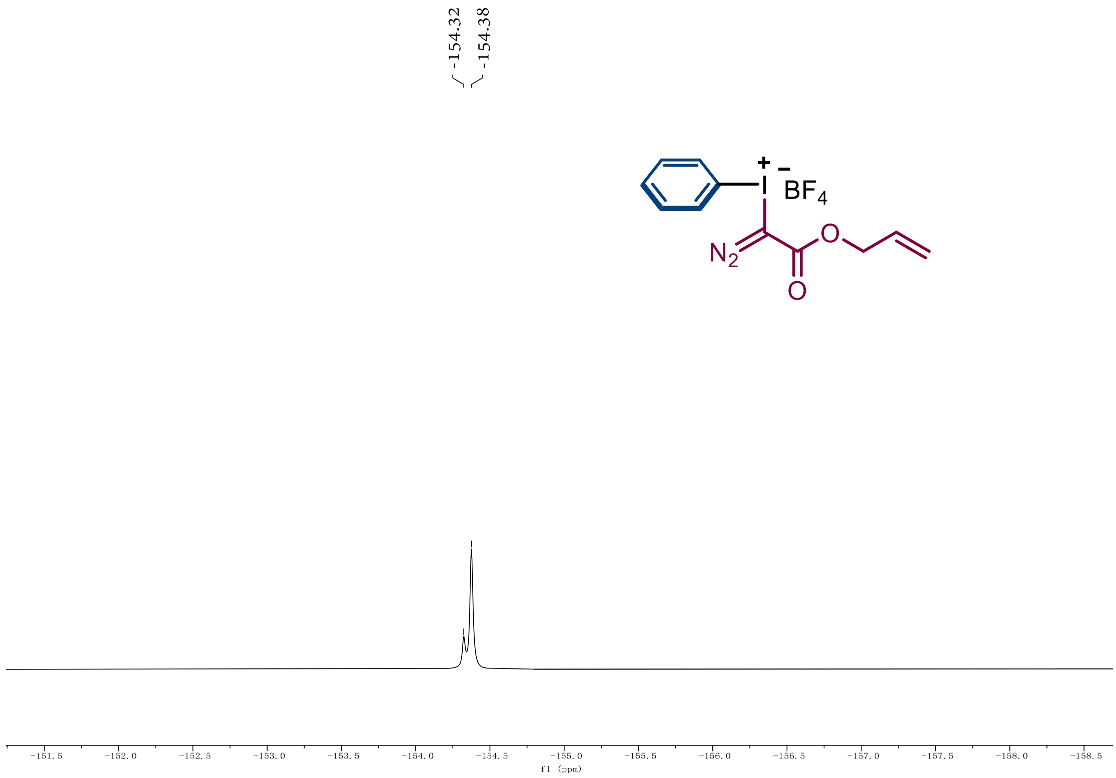
-154.32
-154.38
BF4
N2
O
O
-151.5
-152.0
-152.5
-153.0
-153.5
-154.0
-154.5
-155.0
-155.5
-156.0
-156.5
-157.0
-157.5
-158.0
-158.5
f1 (ppm)

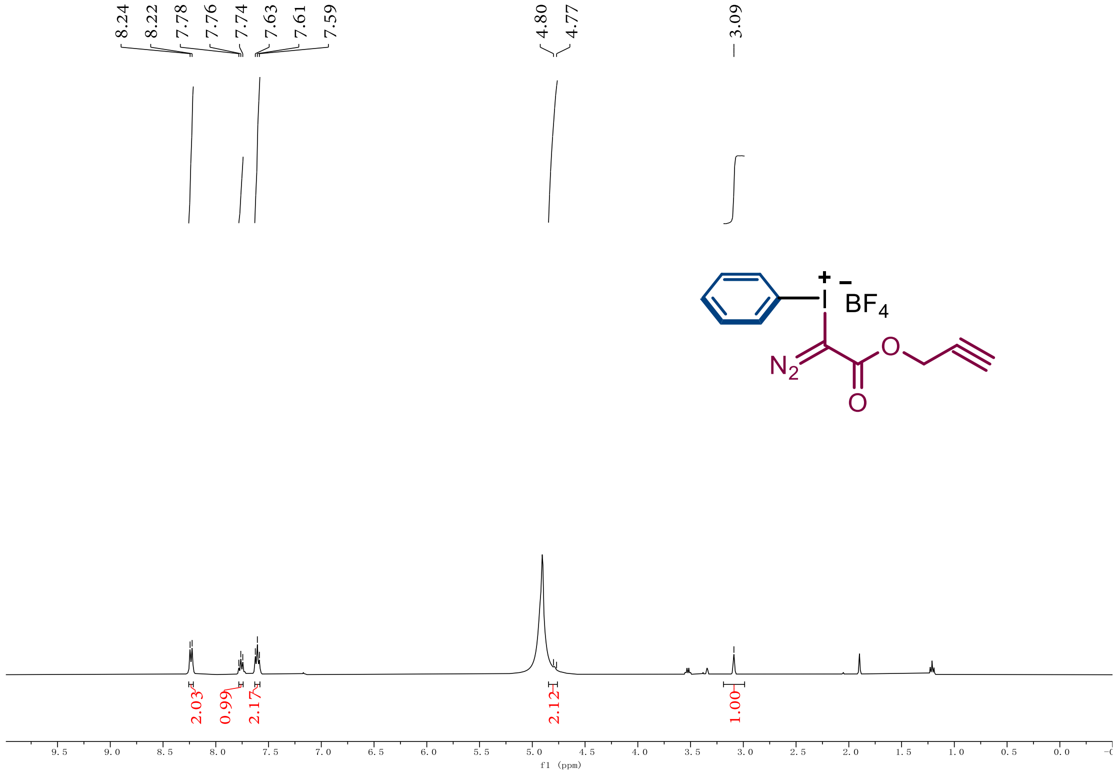
8.24
8.22
7.78
7.76
7.74
7.63
7.61
7.59
4.80
4.77
3.09
BF4
N2
O
O
2.03
0.99
2.17
2.12
1.00
9.5
9.0
8.5
8.0
7.5
7.0
6.5
6.0
5.5
5.0
4.5
4.0
3.5
3.0
2.5
2.0
1.5
1.0
0.5
0.0
f1 (ppm)

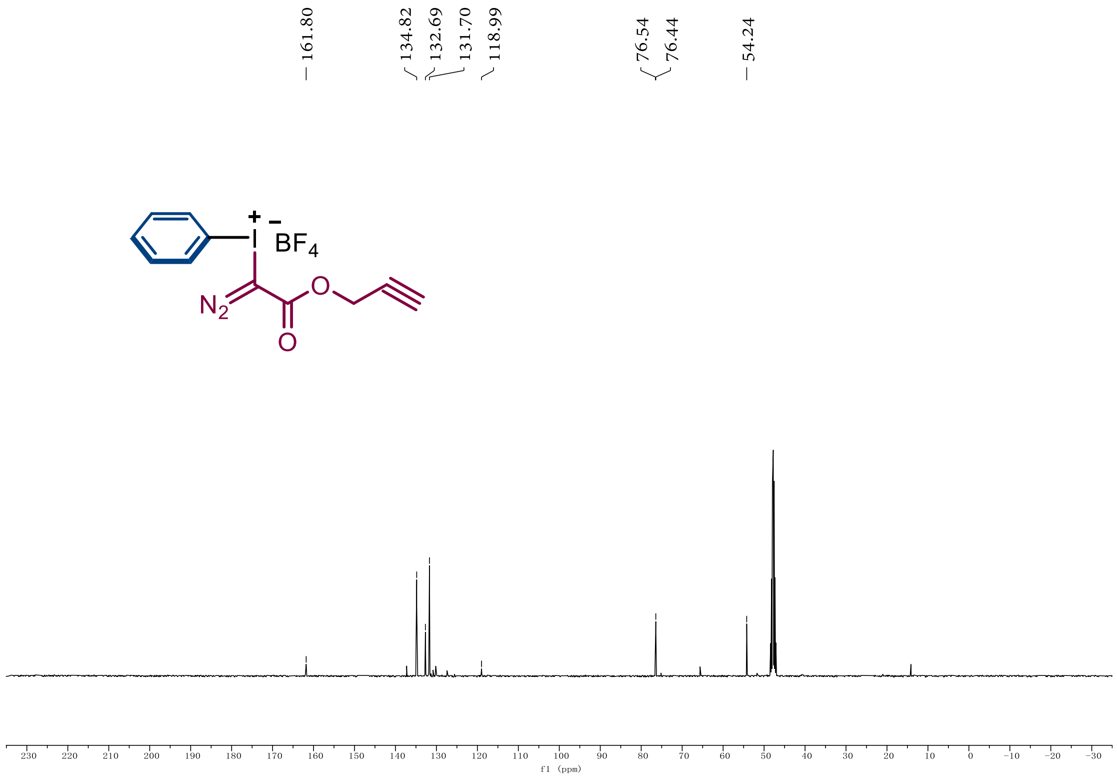
161.80
134.82
132.69
131.70
118.99
76.54
76.44
54.24
BF4
N2
O
f1 (ppm)

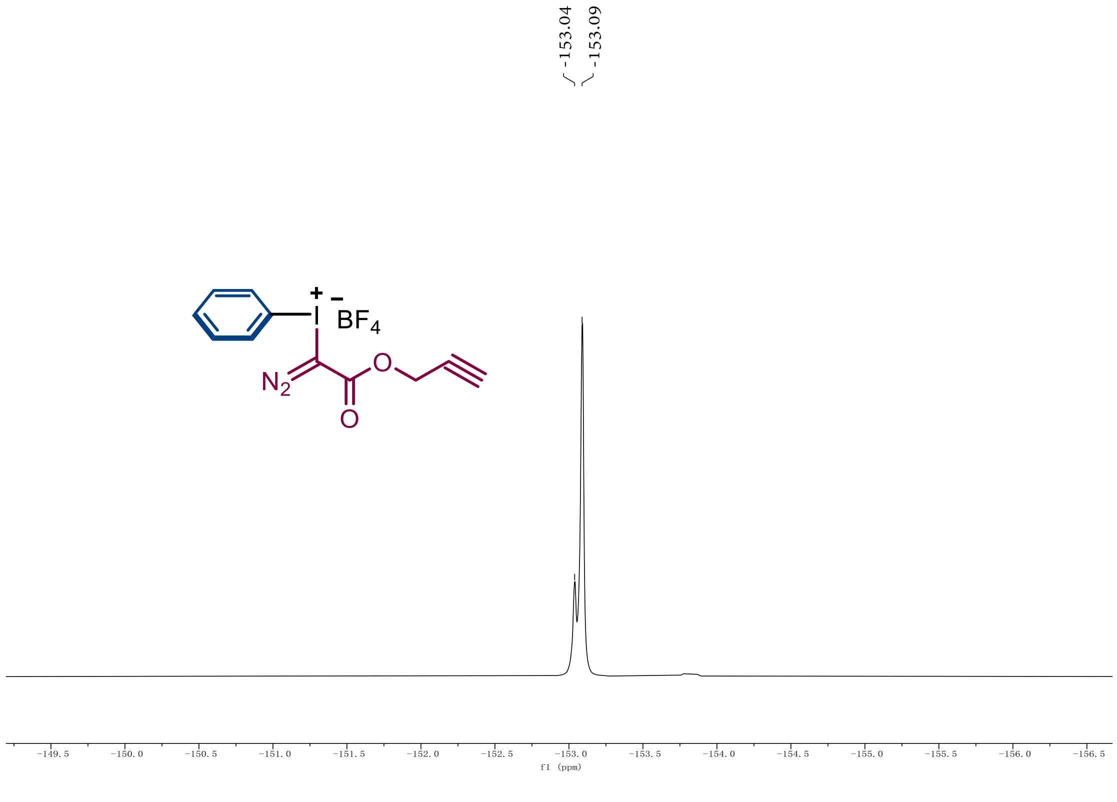
-153.04
-153.09
BF4
N2
O
f1 (ppm)

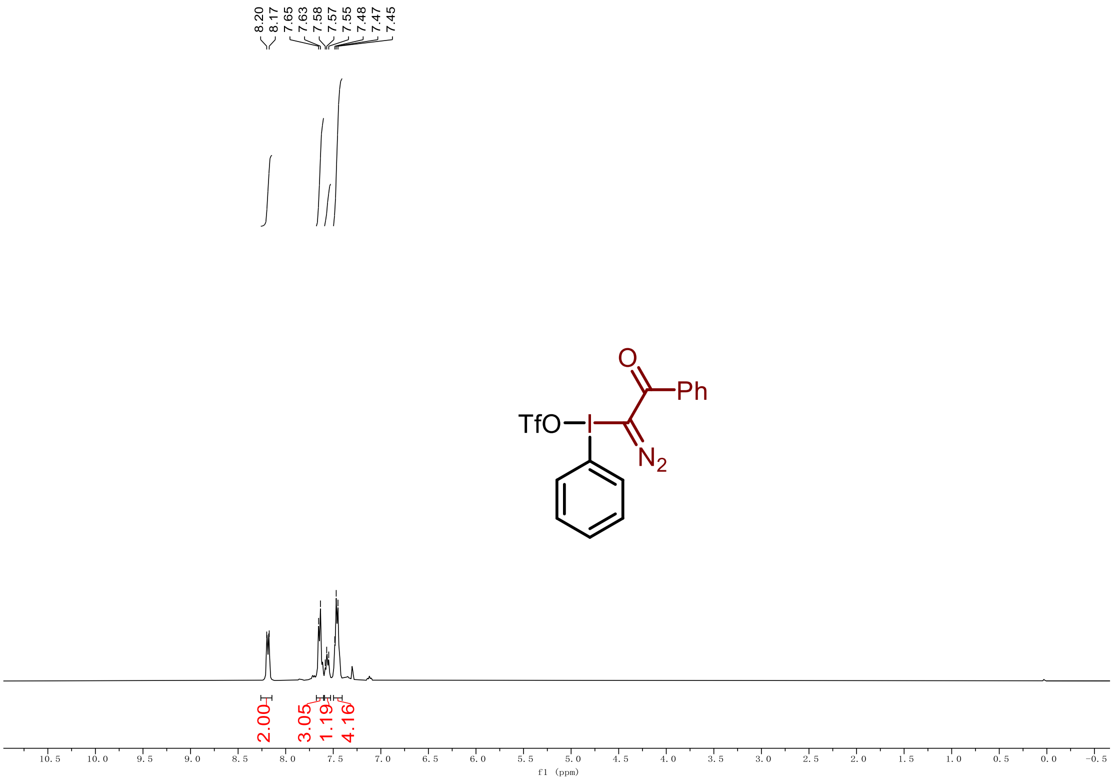
8.20
8.17
7.65
7.63
7.58
7.57
7.55
7.48
7.47
7.45
TfO
I
O
Ph
N2
2.00
3.05
1.19
4.16
f1 (ppm)

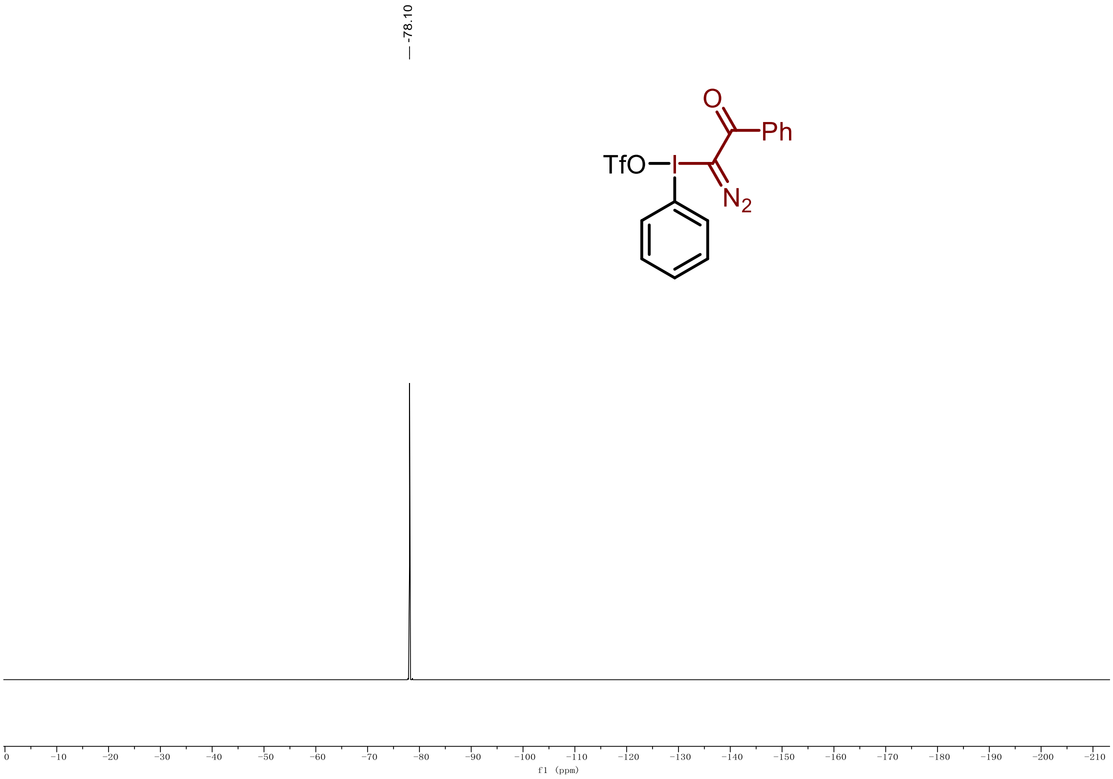
-78.10
TfO
I
O
Ph
N2
f1 (ppm)

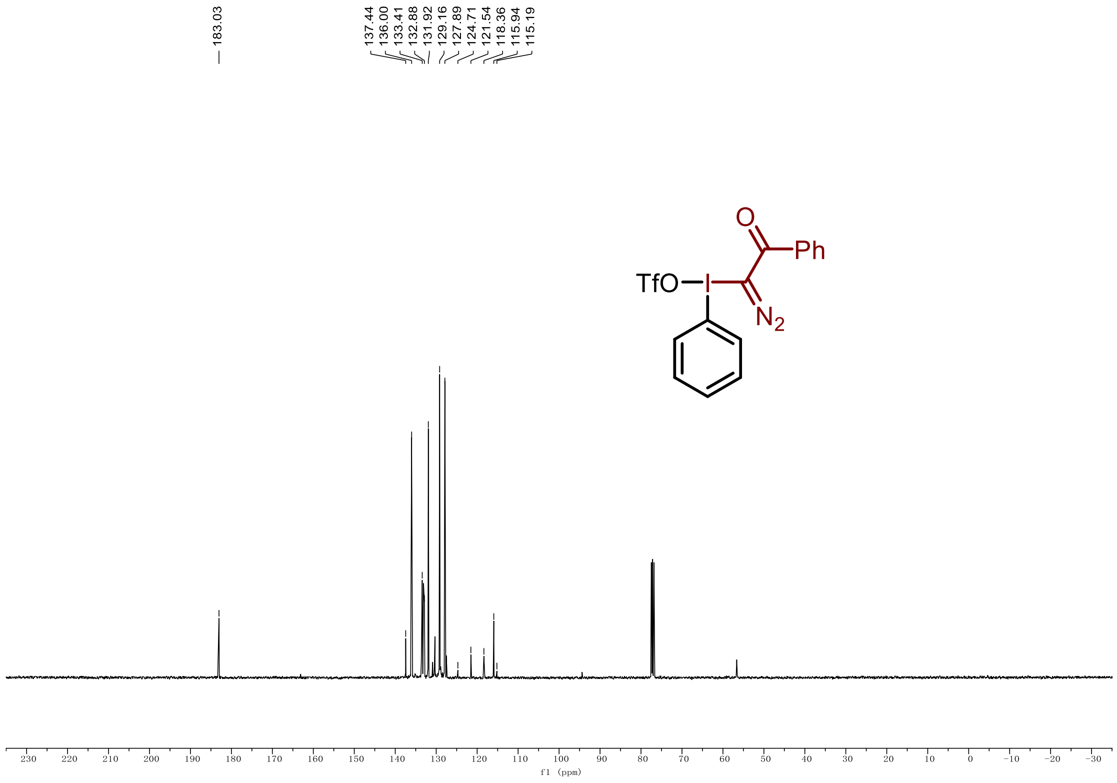
183.03
137.44
136.00
133.41
132.88
131.92
129.16
127.89
124.71
121.54
118.36
115.94
115.19
O
Ph
TfO
I
N2
230 220 210 200 190 180 170 160 150 140 130 120 110 100 90 80 70 60 50 40 30 20 10 0 -10 -20 -30
f1 (ppm)

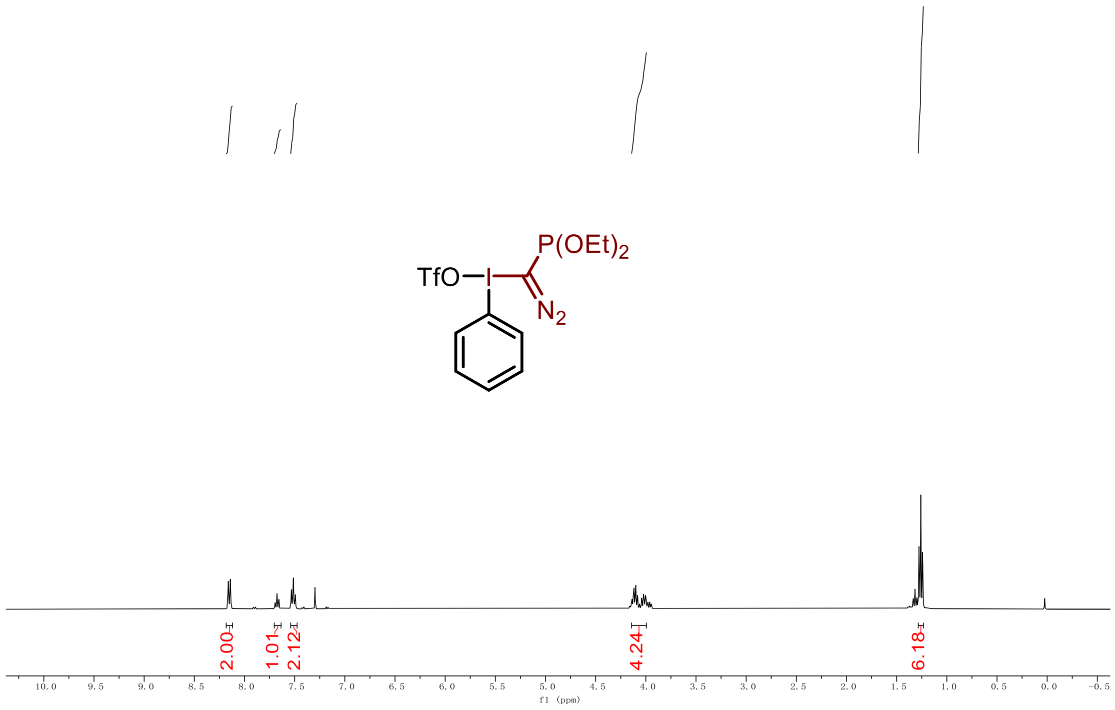
P(OEt)2
TfO
I
N2
2.00
1.01
2.12
4.24
6.18
10.0 9.5 9.0 8.5 8.0 7.5 7.0 6.5 6.0 5.5 5.0 4.5 4.0 3.5 3.0 2.5 2.0 1.5 1.0 0.5 0.0 -0.5
f1 (ppm)

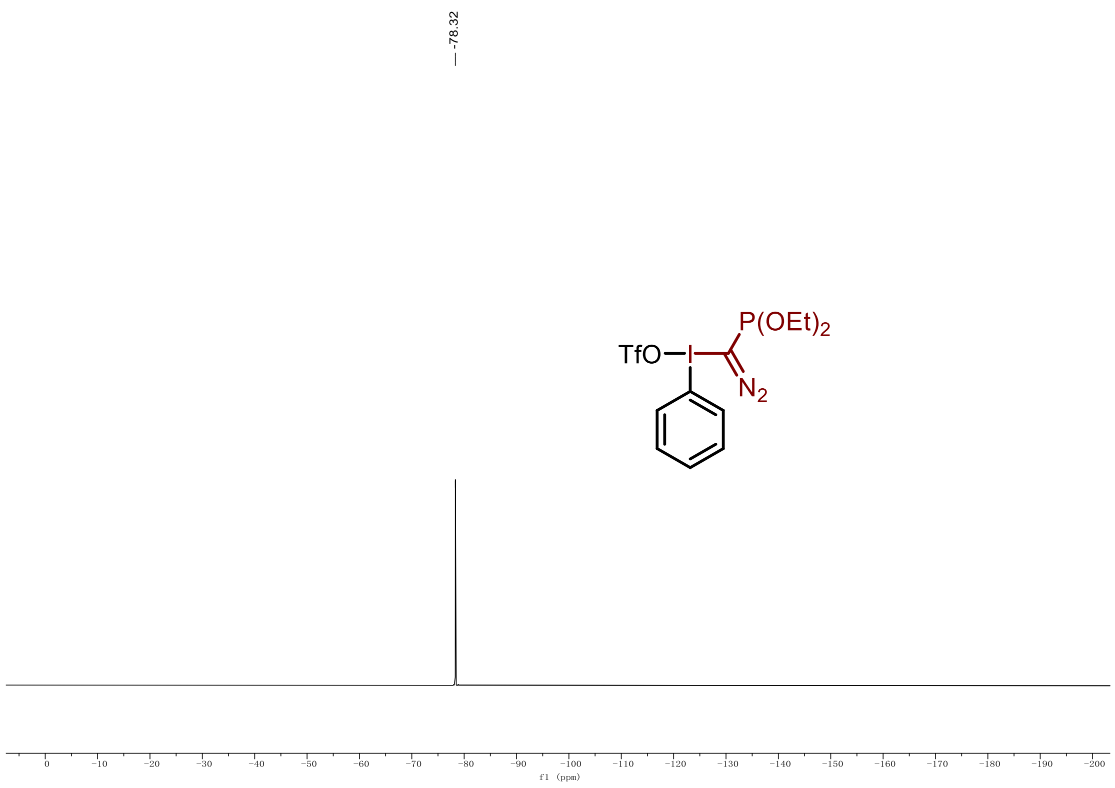
-78.32
P(OEt)2
TfO
I
N2
f1 (ppm)

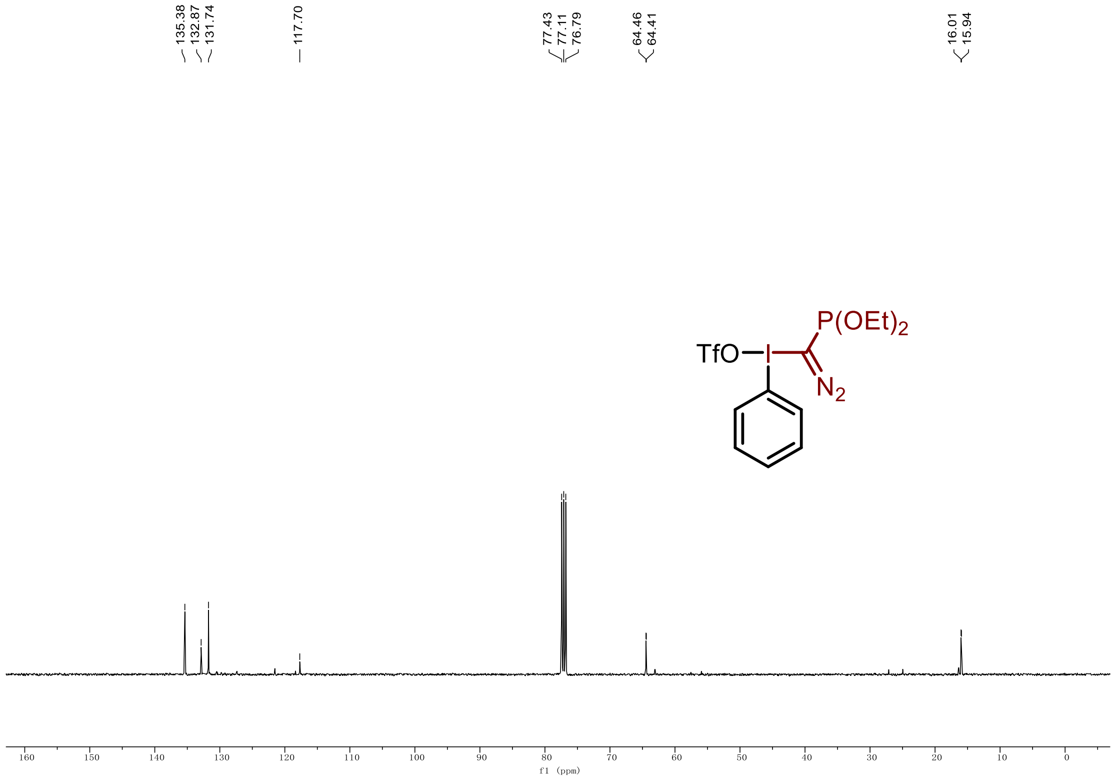
135.38
132.87
131.74
117.70
77.43
77.11
76.79
64.46
64.41
16.01
15.94
P(OEt)2
TfO
I
N2
f1 (ppm)

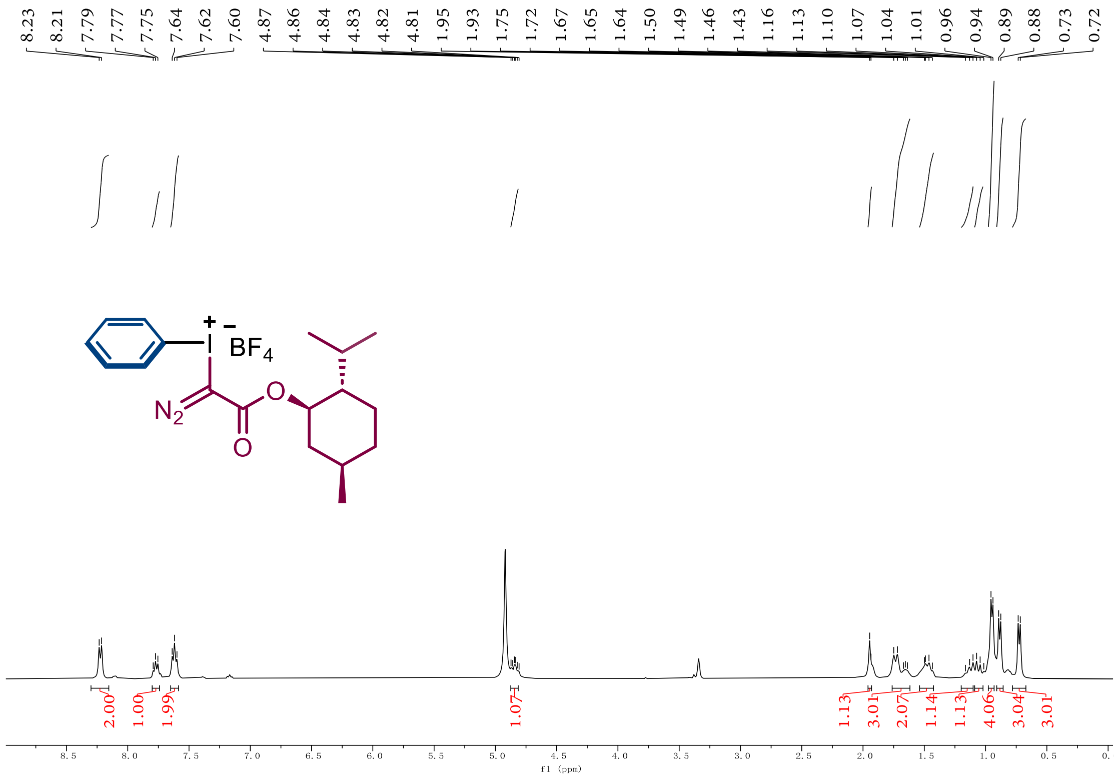

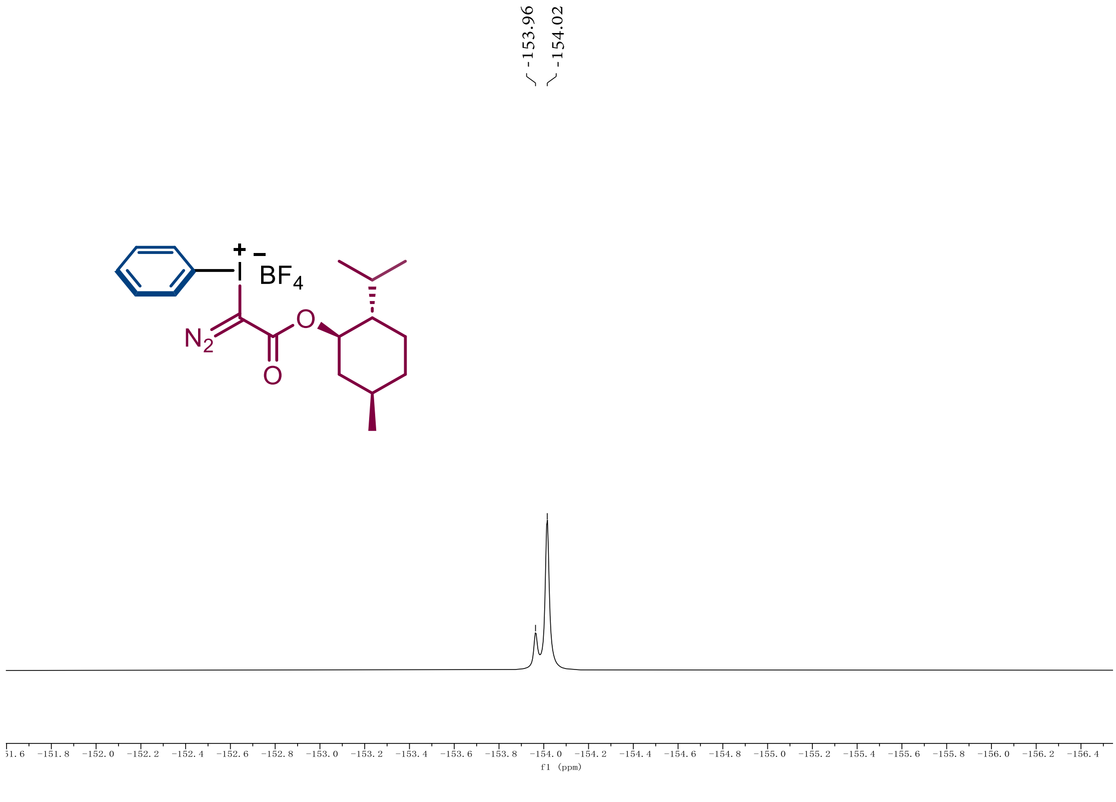

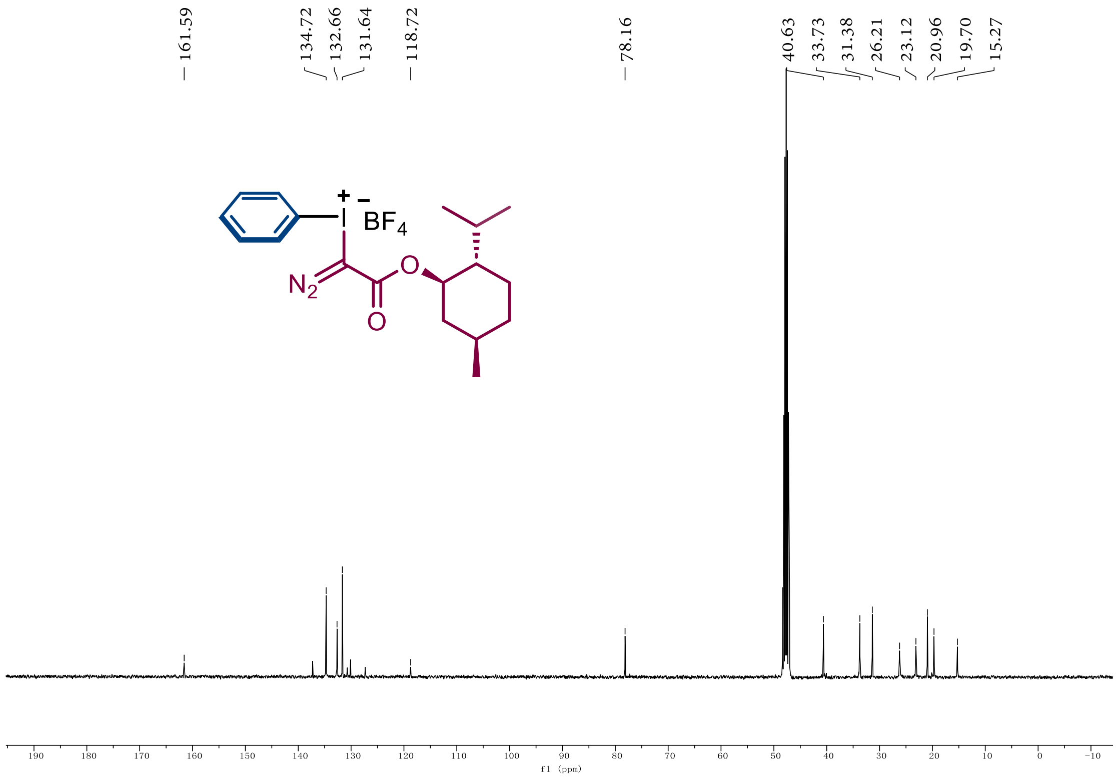

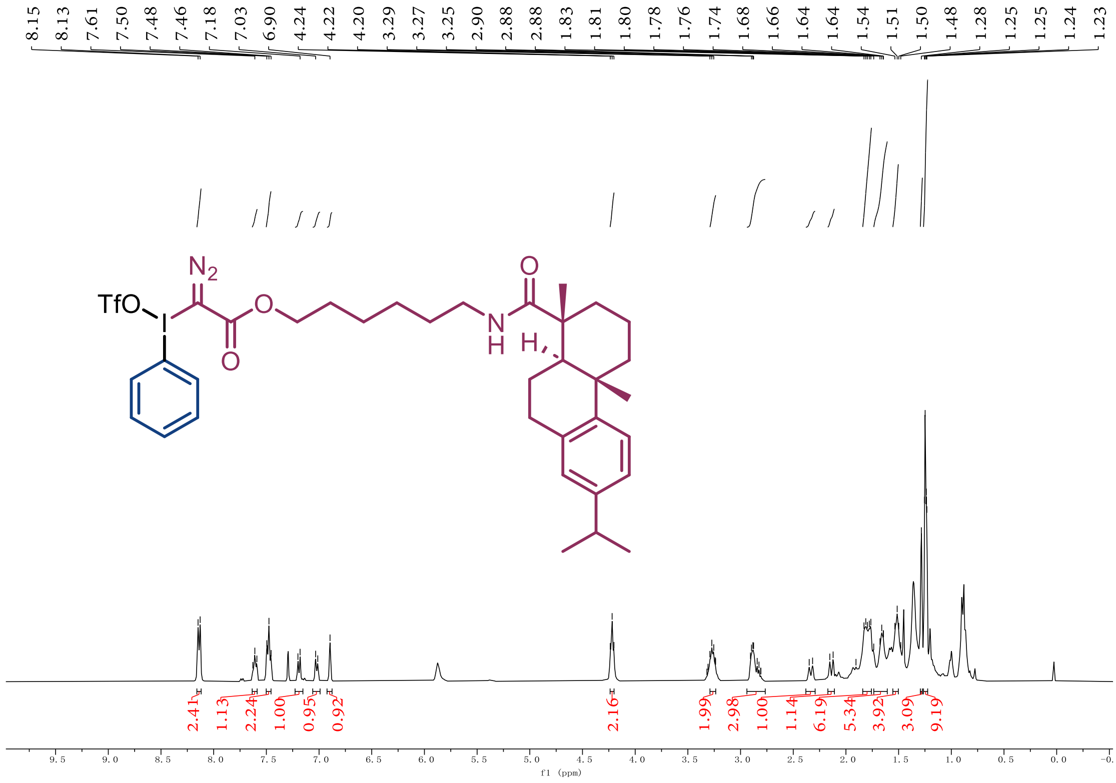

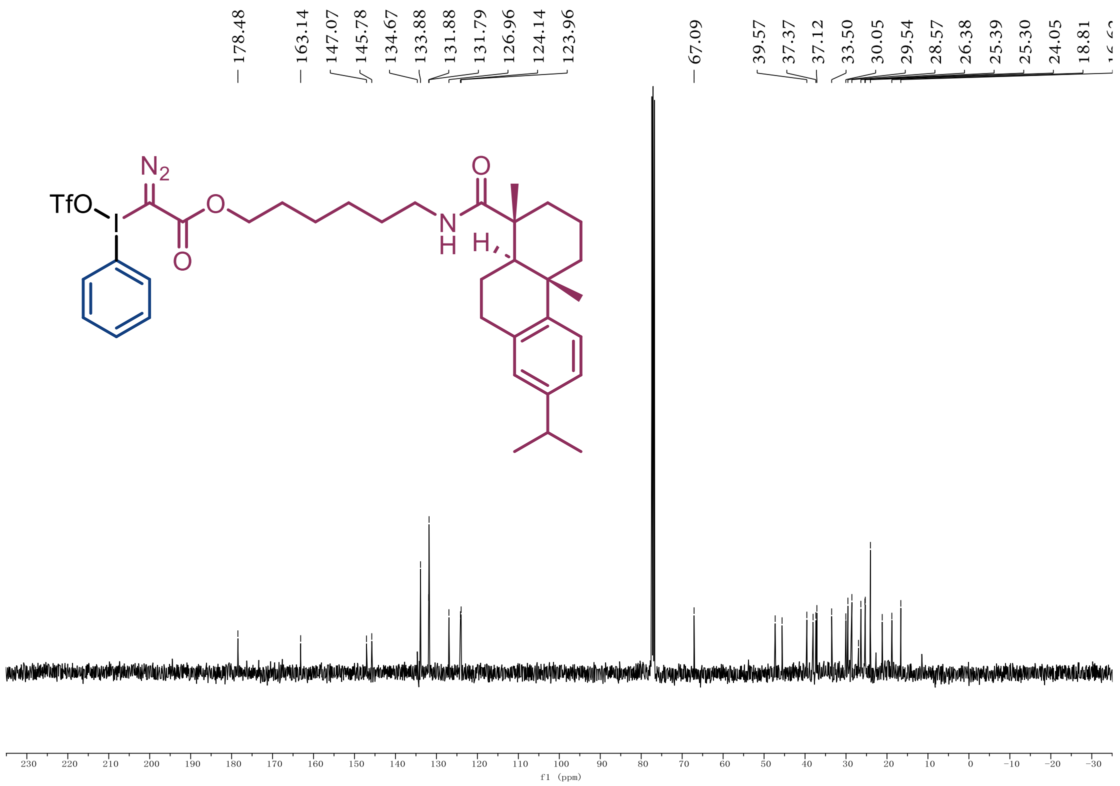
178.48
163.14
147.07
145.78
134.67
133.88
131.88
131.79
126.96
124.14
123.96
67.09
39.57
37.37
37.12
33.50
30.05
29.54
28.57
26.38
25.39
25.30
24.05
18.81
TfO
N2
f1 (ppm)

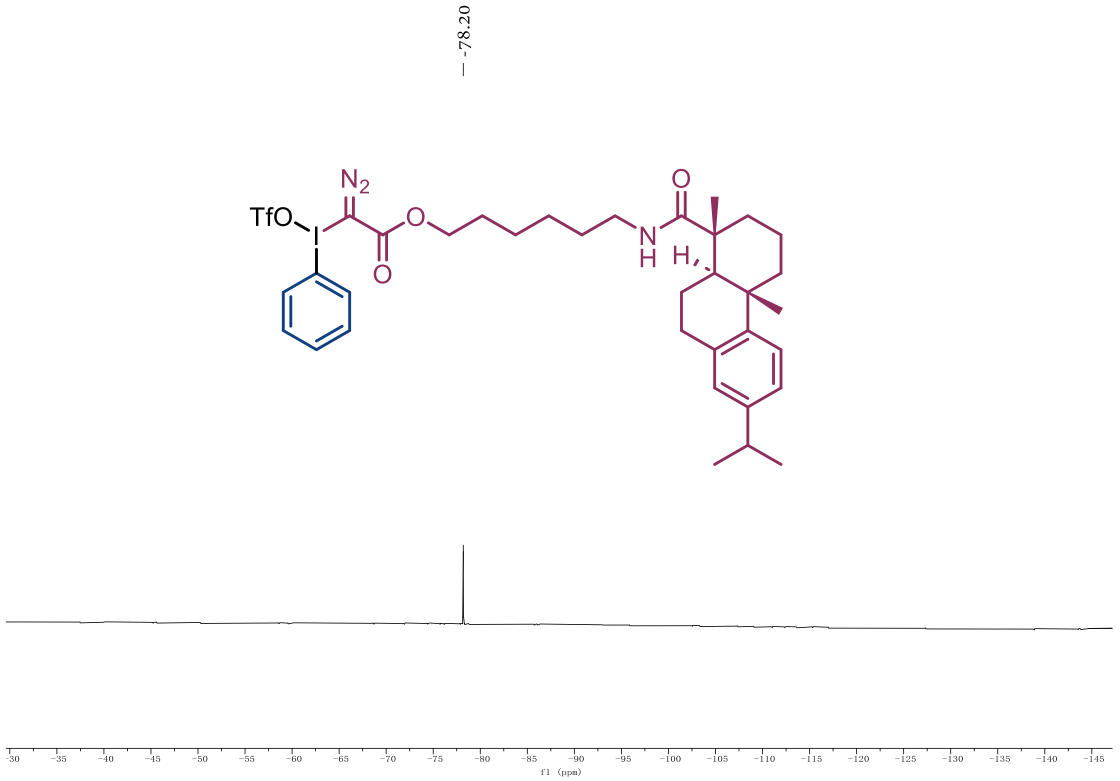
-78.20
TfO
N2
f1 (ppm)

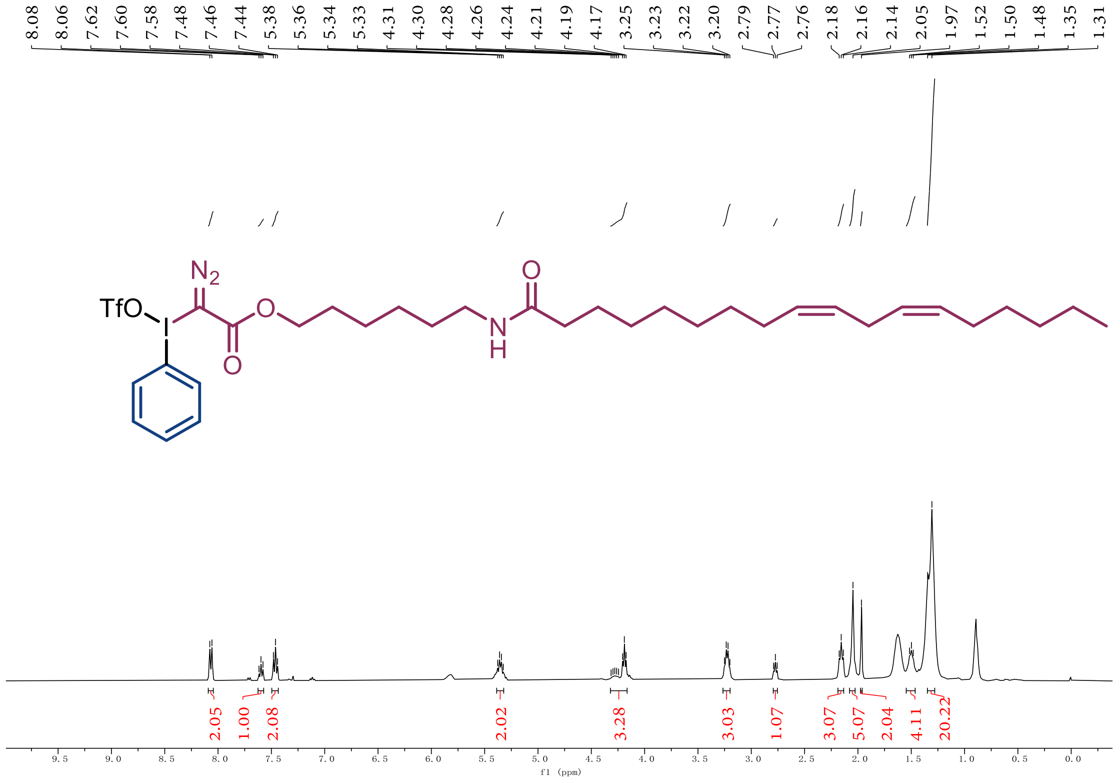

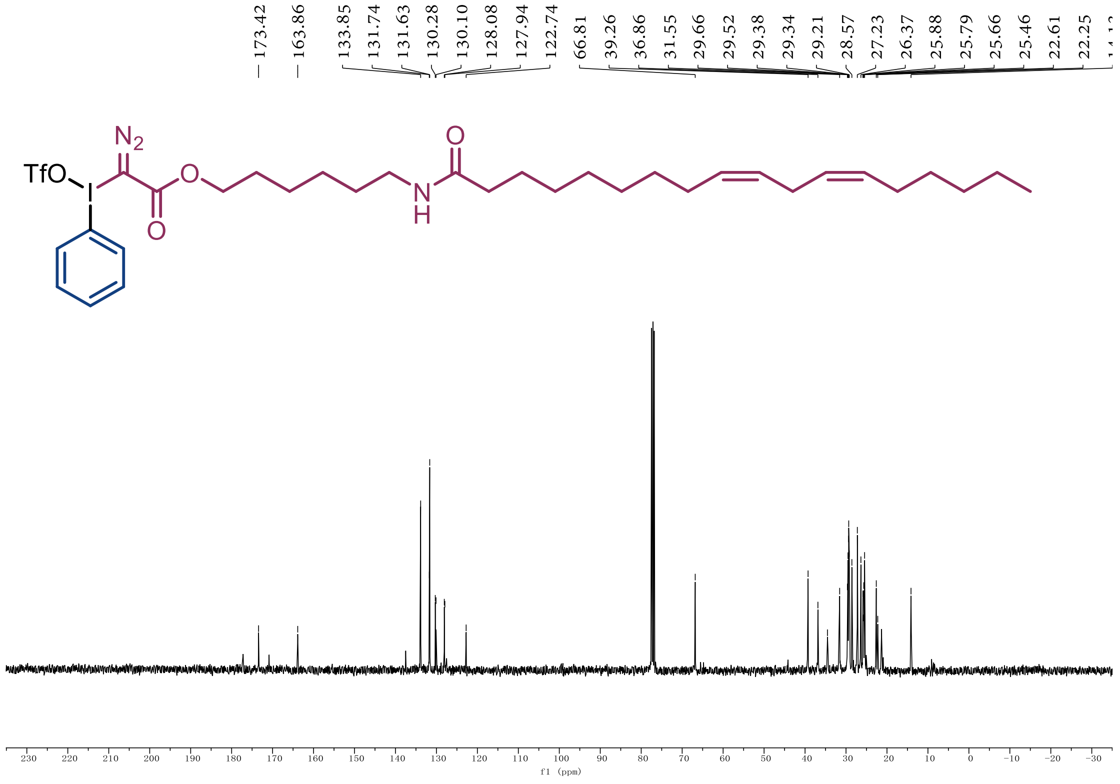

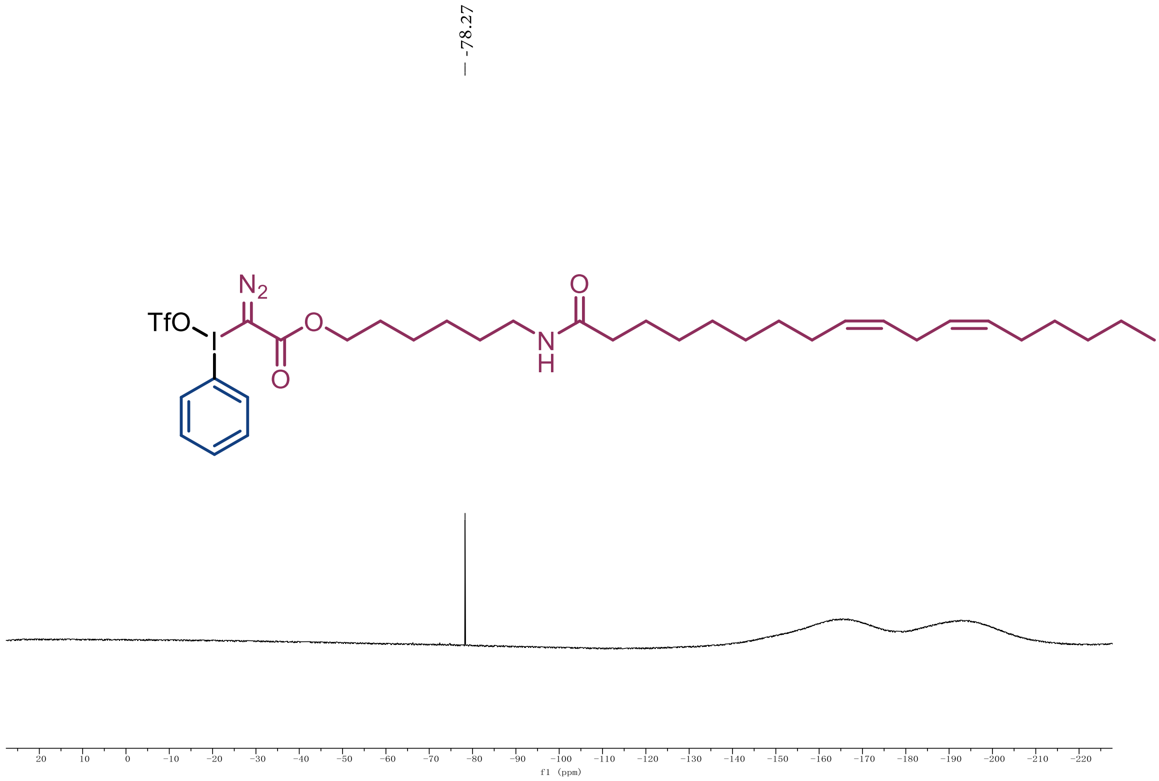
-78.27
TfO
N2
O
O
O
N
H
f1 (ppm)

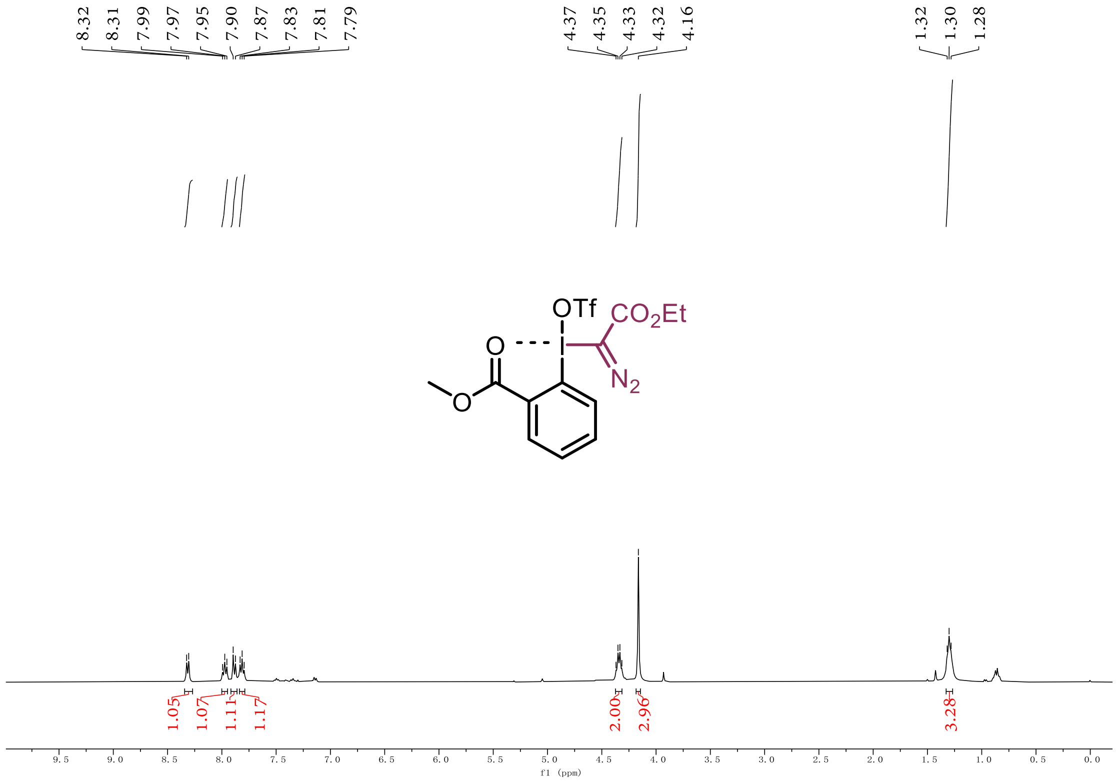
8.32
8.31
7.99
7.97
7.95
7.90
7.87
7.83
7.81
7.79
4.37
4.35
4.33
4.32
4.16
1.32
1.30
1.28
OTf
CO2Et
O
O
N2
1.05
1.07
1.11
1.17
2.00
2.96
3.28
f1 (ppm)

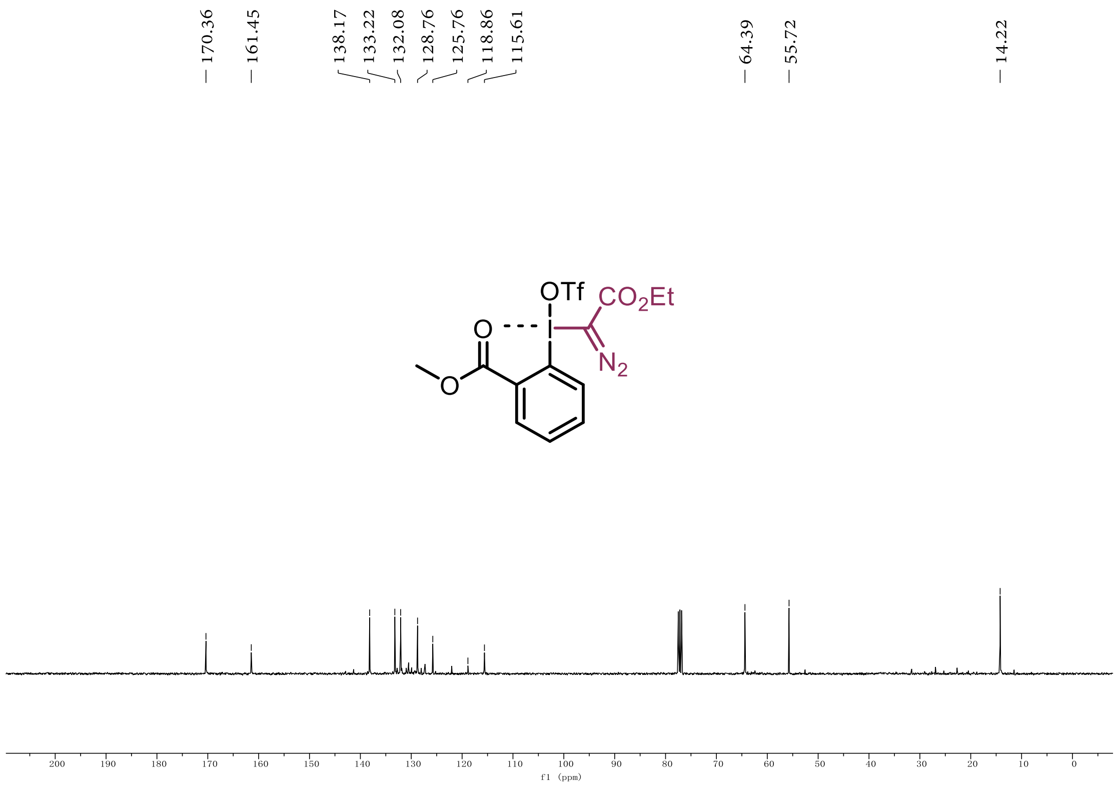
170.36
161.45
138.17
133.22
132.08
128.76
125.76
118.86
115.61
64.39
55.72
14.22
OTf
CO2Et
N2
O
O
I
f1 (ppm)

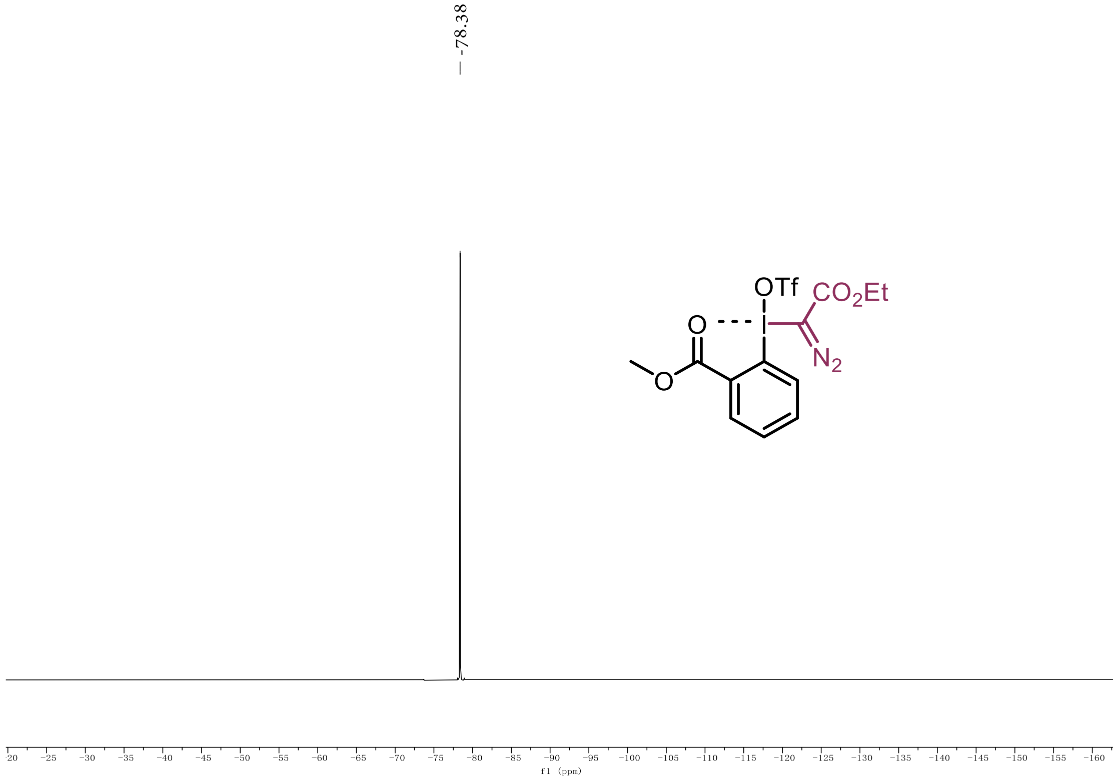
-78.38
OTf
CO2Et
N2
O
O
I
f1 (ppm)

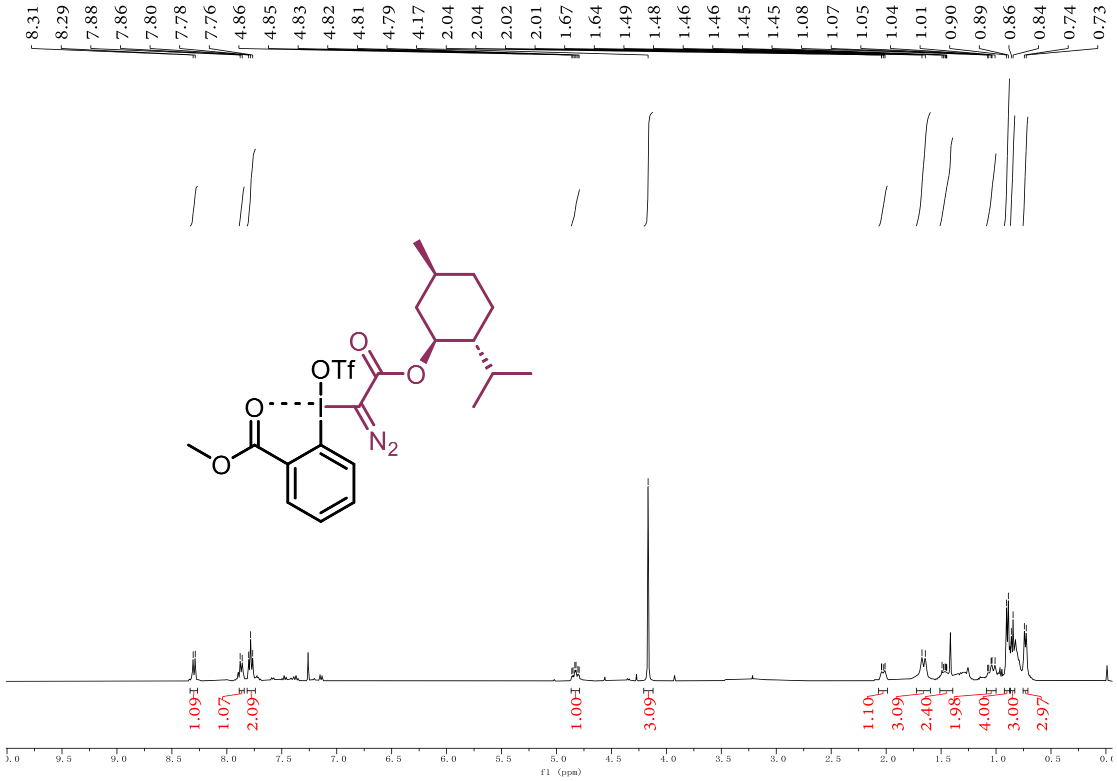
8.31
8.29
7.88
7.86
7.80
7.78
7.76
4.86
4.85
4.83
4.82
4.81
4.79
4.17
2.04
2.04
2.02
2.01
1.67
1.64
1.49
1.48
1.46
1.46
1.45
1.45
1.08
1.07
1.05
1.04
1.01
0.90
0.89
0.86
0.84
0.74
0.73
OTf
O
O
O
O
I
N2
1.09
1.07
2.09
1.00
3.09
1.10
3.09
2.40
1.98
4.00
3.00
2.97
f1 (ppm)

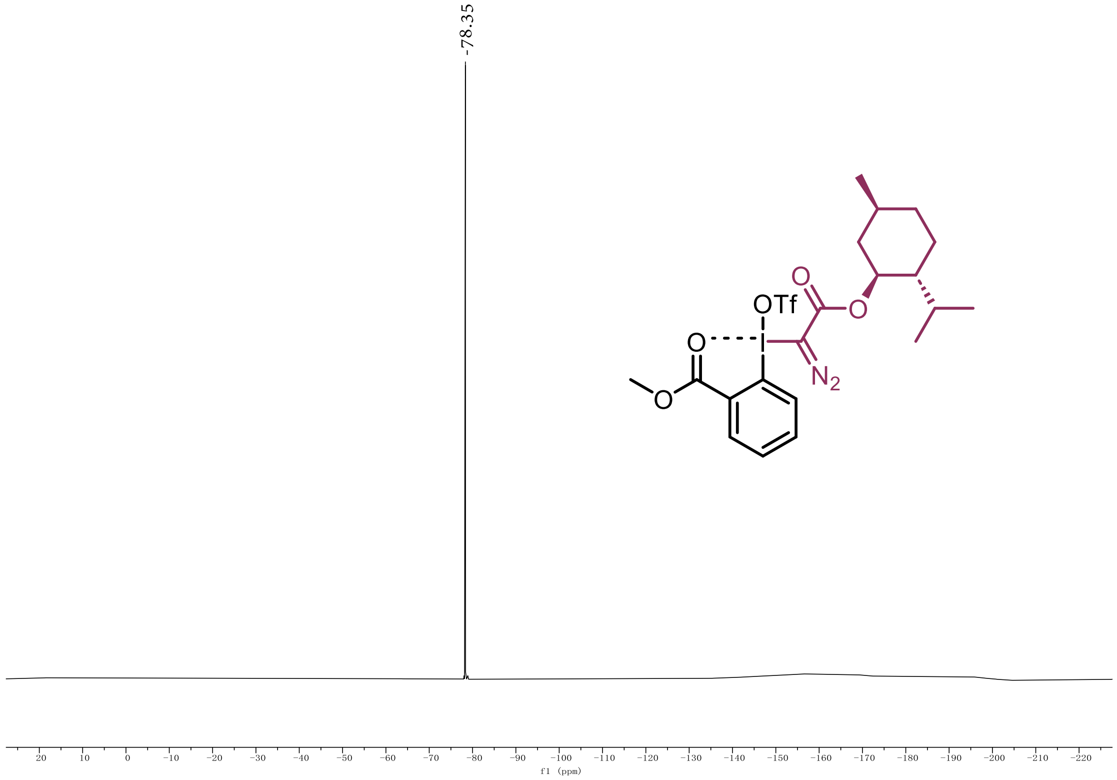
-78.35
OTf
O
O
O
O
I
N2
f1 (ppm)

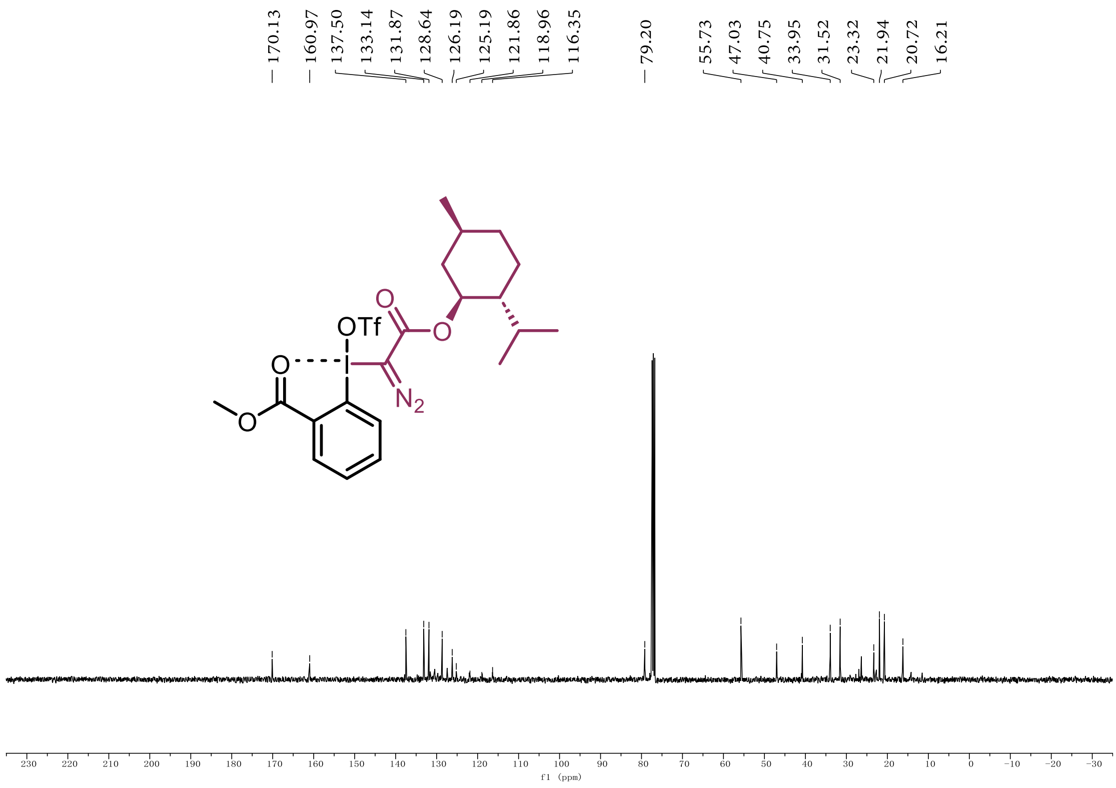

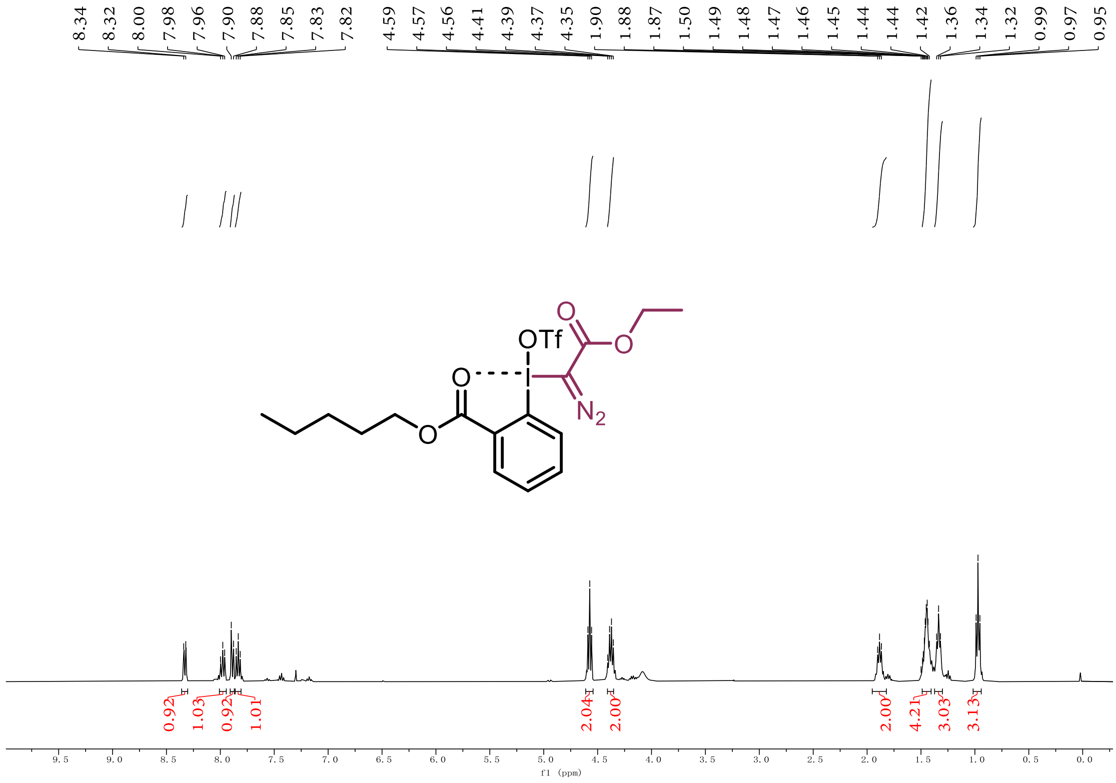

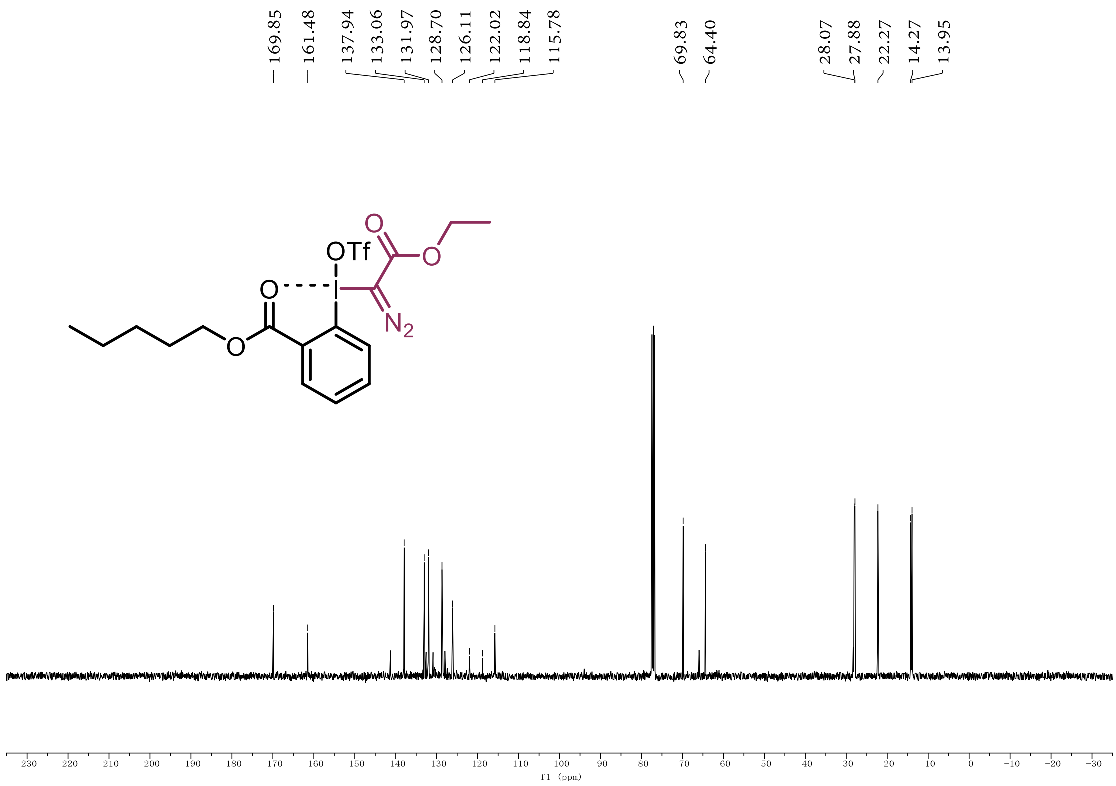
169.85
161.48
137.94
133.06
131.97
128.70
126.11
122.02
118.84
115.78
69.83
64.40
28.07
27.88
22.27
14.27
13.95
OTf
O
I
O
O
O
N2
230 220 210 200 190 180 170 160 150 140 130 120 110 100 90 80 70 60 50 40 30 20 10 0 -10 -20 -30
f1 (ppm)

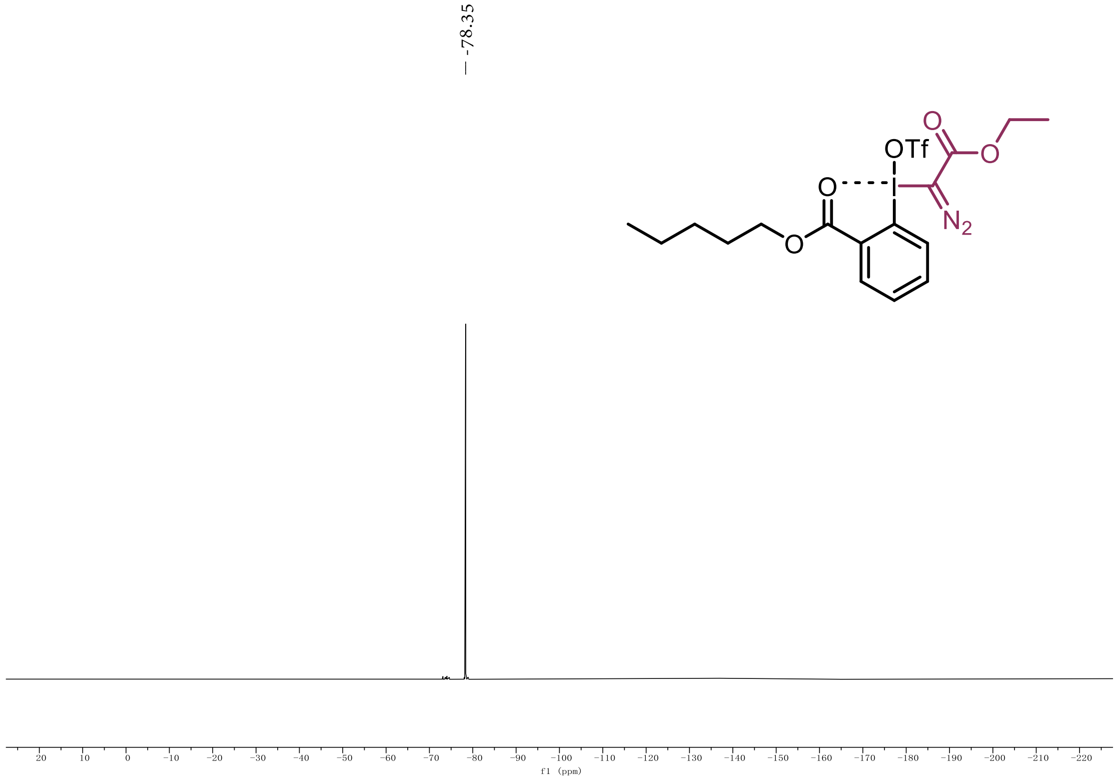
-78.35
OTf
O
I
O
O
O
N2
20 10 0 -10 -20 -30 -40 -50 -60 -70 -80 -90 -100 -110 -120 -130 -140 -150 -160 -170 -180 -190 -200 -210 -220
f1 (ppm)

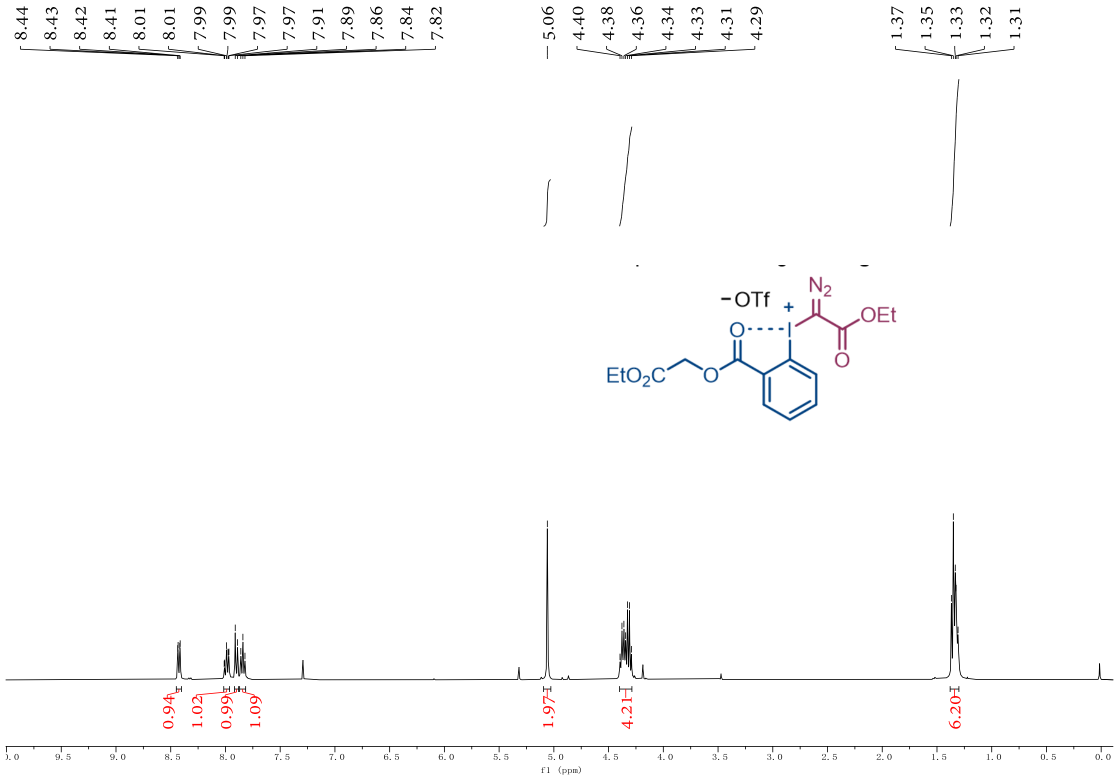
8.44 8.43 8.42 8.41 8.01 8.01 7.99 7.99 7.97 7.97 7.91 7.89 7.86 7.84 7.82
5.06
4.40 4.38 4.36 4.34 4.33 4.31 4.29
1.37 1.35 1.33 1.32 1.31
-OTf
+
N2
OEt
O
I
EtO2C
0.94
1.02
0.99
1.09
1.97
4.21
6.20
f1 (ppm)

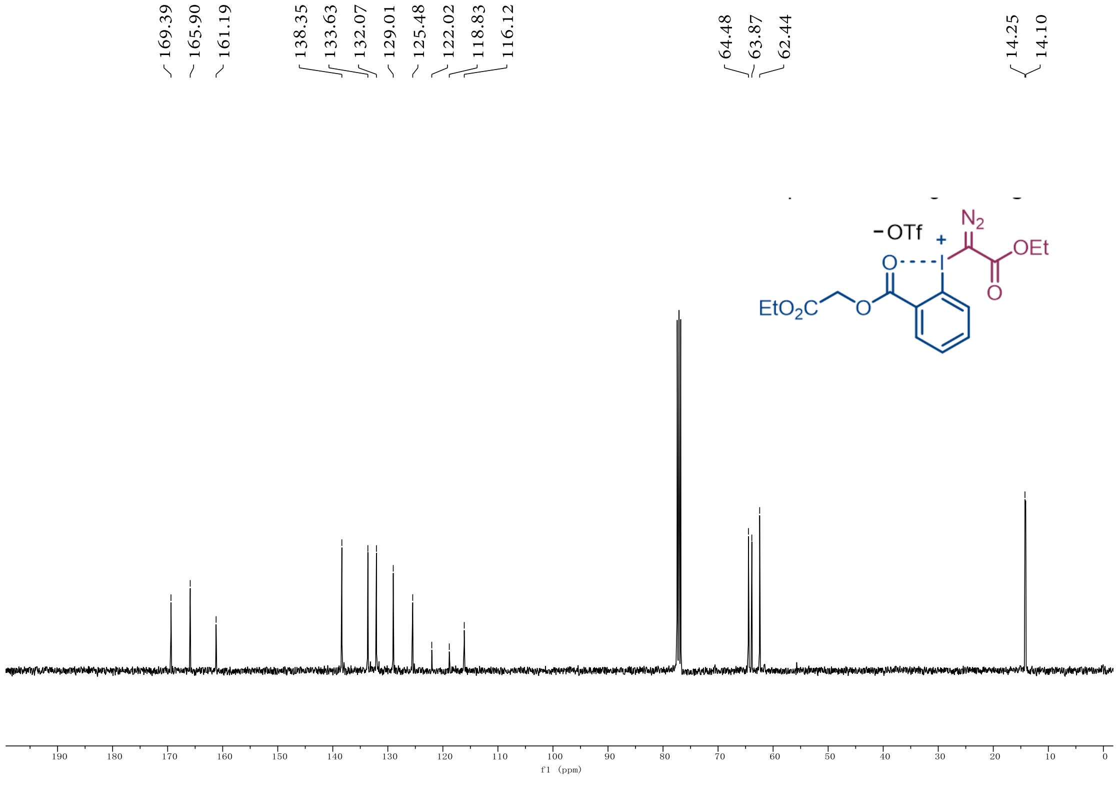
169.39 165.90 161.19
138.35 133.63 132.07 129.01 125.48 122.02 118.83 116.12
64.48 63.87 62.44
14.25 14.10
-OTf
+
N2
OEt
O
I
EtO2C
f1 (ppm)

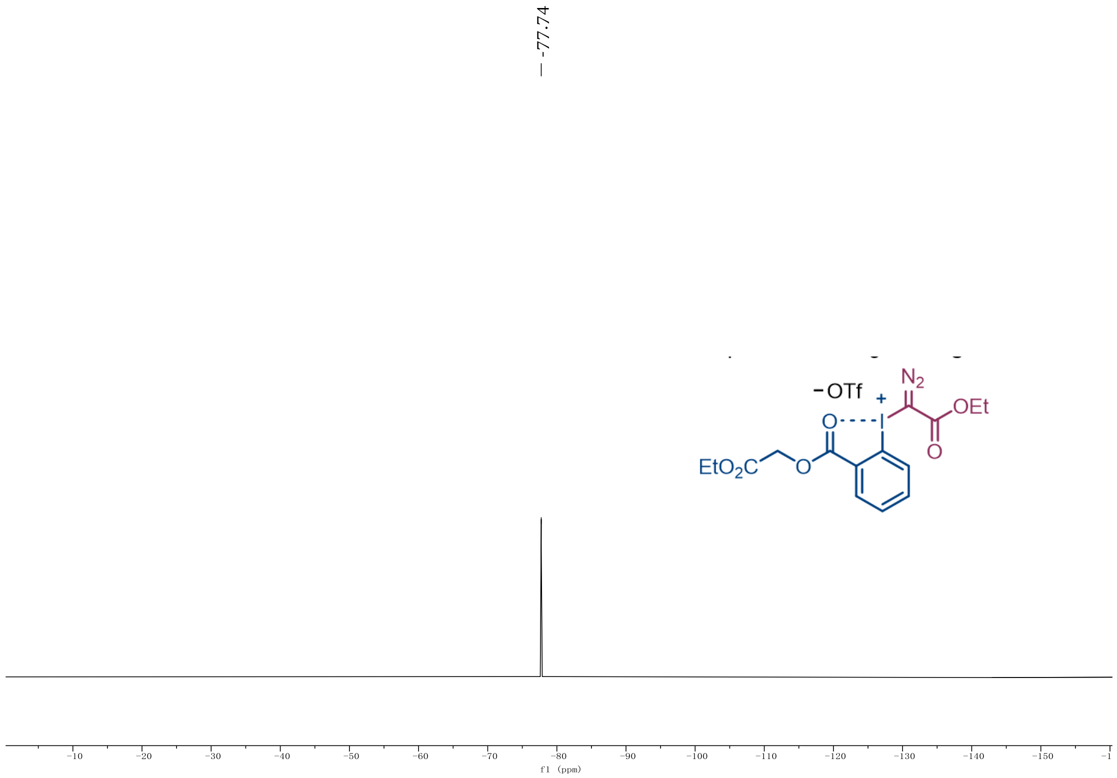
-77.74
-OTf
N2
OEt
O
I
+
EtO2C
O
O
O
-10
-20
-30
-40
-50
-60
-70
-80
-90
-100
-110
-120
-130
-140
-150
f1 (ppm)

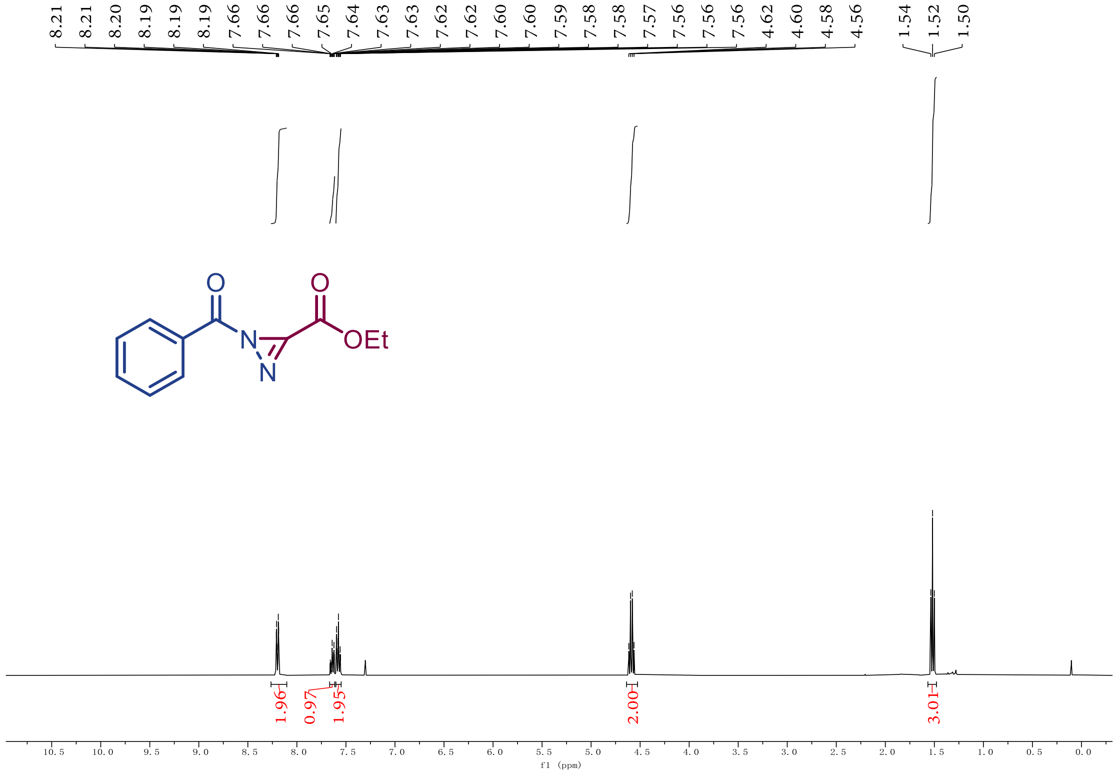
8.21
8.21
8.20
8.19
8.19
8.19
7.66
7.66
7.66
7.65
7.64
7.63
7.63
7.62
7.62
7.60
7.60
7.59
7.58
7.58
7.57
7.56
7.56
7.56
4.62
4.60
4.58
4.56
1.54
1.52
1.50
O
O
N
N
OEt
1.96
0.97
1.95
2.00
3.01
10.5
10.0
9.5
9.0
8.5
8.0
7.5
7.0
6.5
6.0
5.5
5.0
4.5
4.0
3.5
3.0
2.5
2.0
1.5
1.0
0.5
0.0
f1 (ppm)

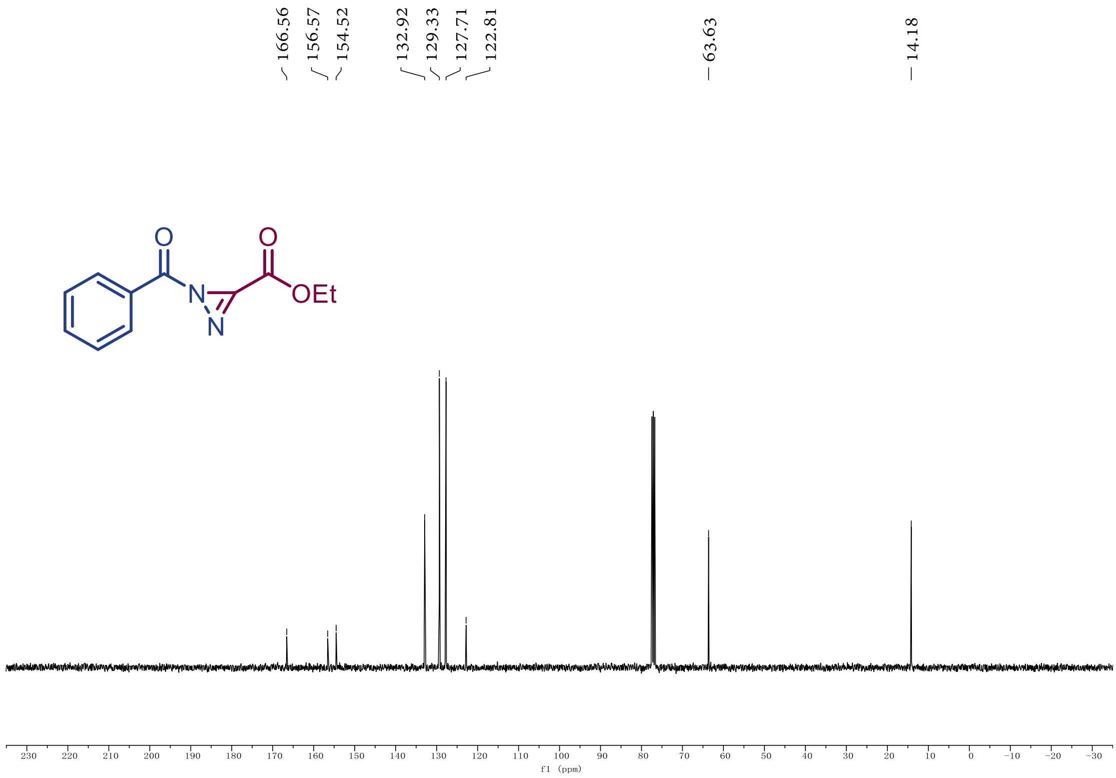
166.56
156.57
154.52
132.92
129.33
127.71
122.81
63.63
14.18
O
O
N
N
OEt
f1 (ppm)

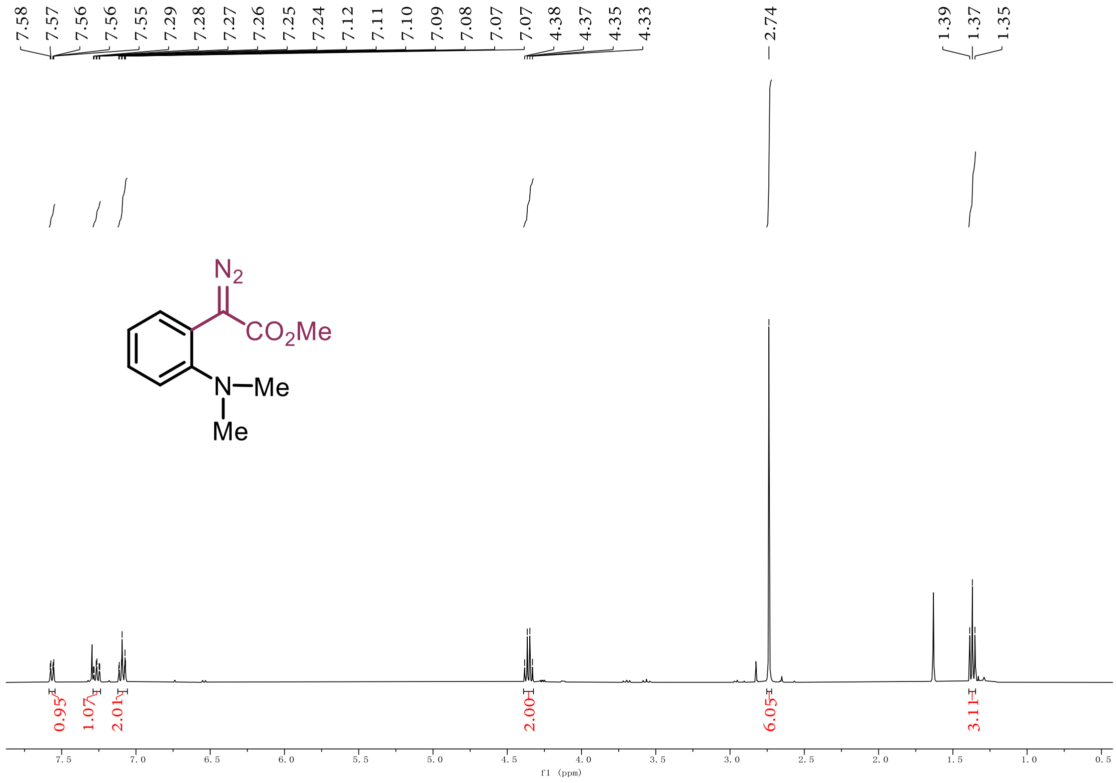
7.58
7.57
7.56
7.56
7.55
7.29
7.28
7.27
7.26
7.25
7.24
7.12
7.11
7.10
7.09
7.08
7.07
7.07
4.38
4.37
4.35
4.33
2.74
1.39
1.37
1.35
N2
CO2Me
N
Me
Me
0.95
1.07
2.01
2.00
6.05
3.11
f1 (ppm)

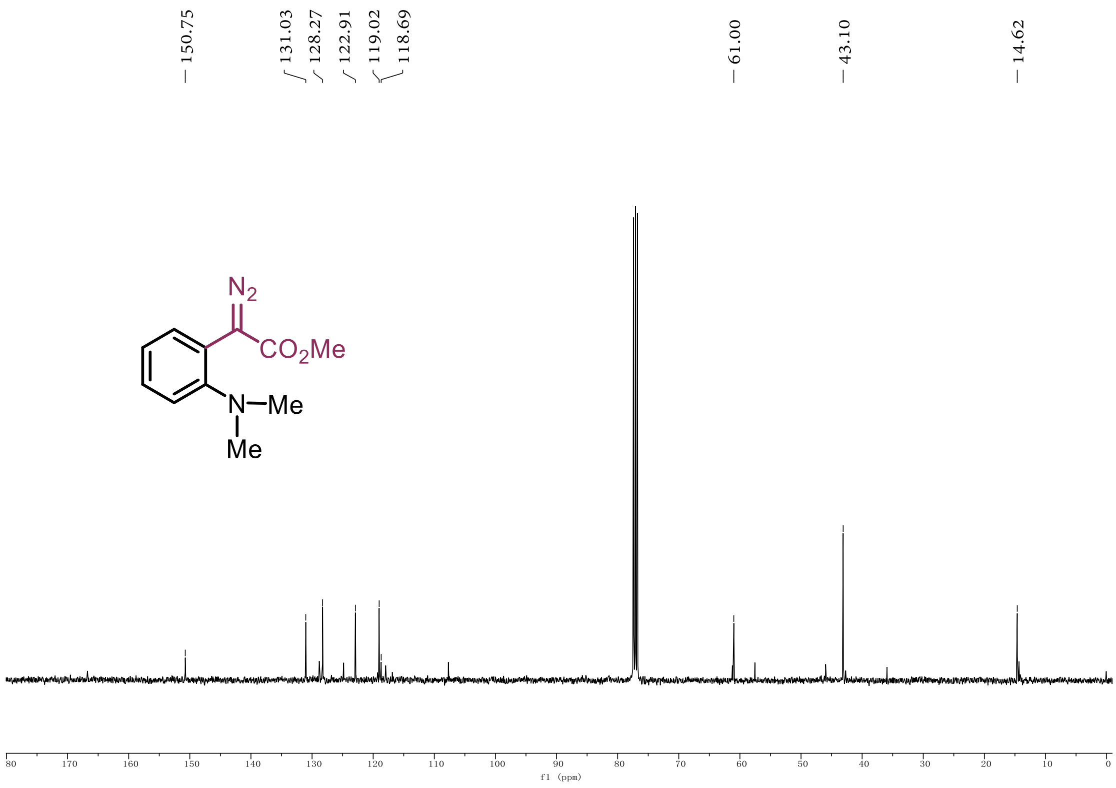

150.75
131.03
128.27
122.91
119.02
118.69
61.00
43.10
14.62
N2
CO2Me
N
Me
Me
f1 (ppm)

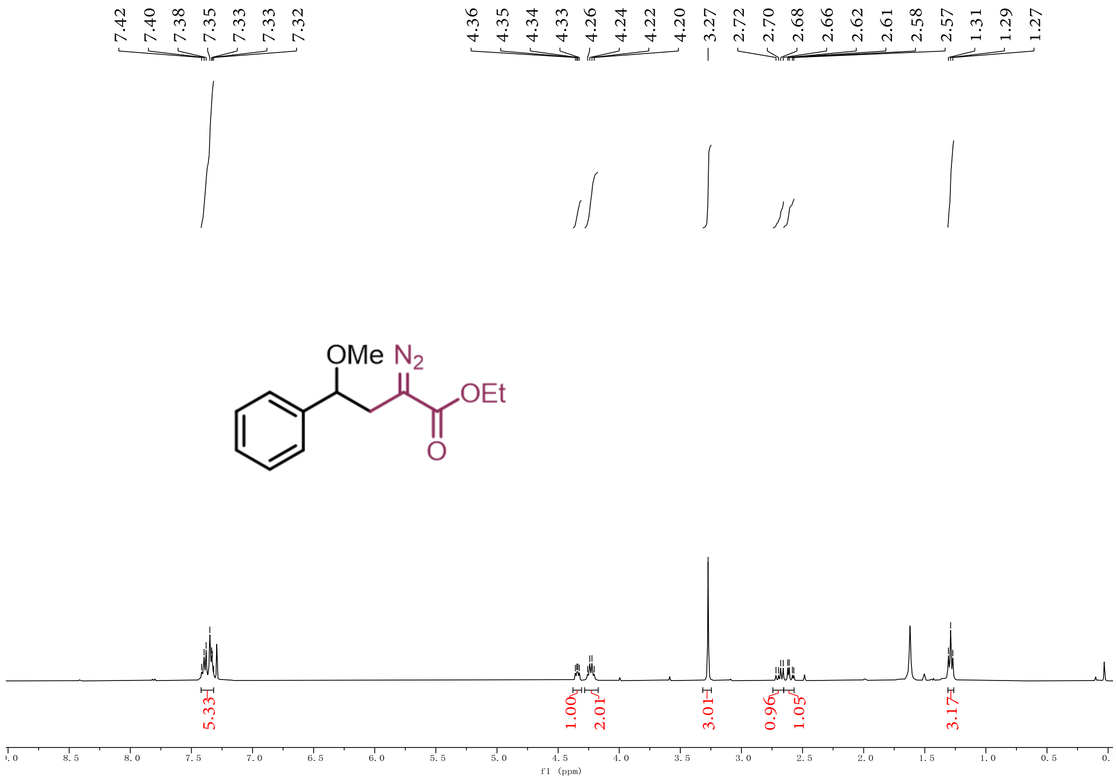

7.42
7.40
7.38
7.35
7.33
7.33
7.32
4.36
4.35
4.34
4.33
4.26
4.24
4.22
4.20
3.27
2.72
2.70
2.68
2.66
2.62
2.61
2.58
2.57
1.31
1.29
1.27
OMe
N2
OEt
O
5.33
1.00
2.01
3.01
0.96
1.05
3.17
f1 (ppm)

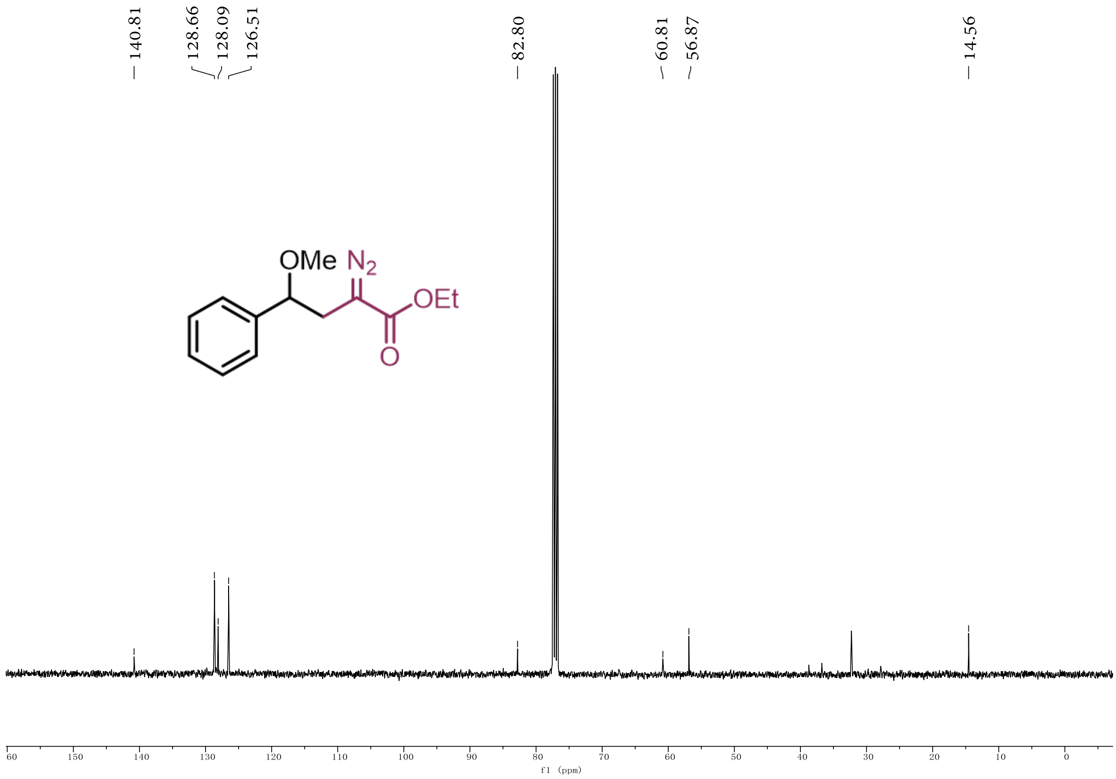

140.81
128.66
128.09
126.51
82.80
60.81
56.87
14.56
OMe
N2
OEt
O
f1 (ppm)

.

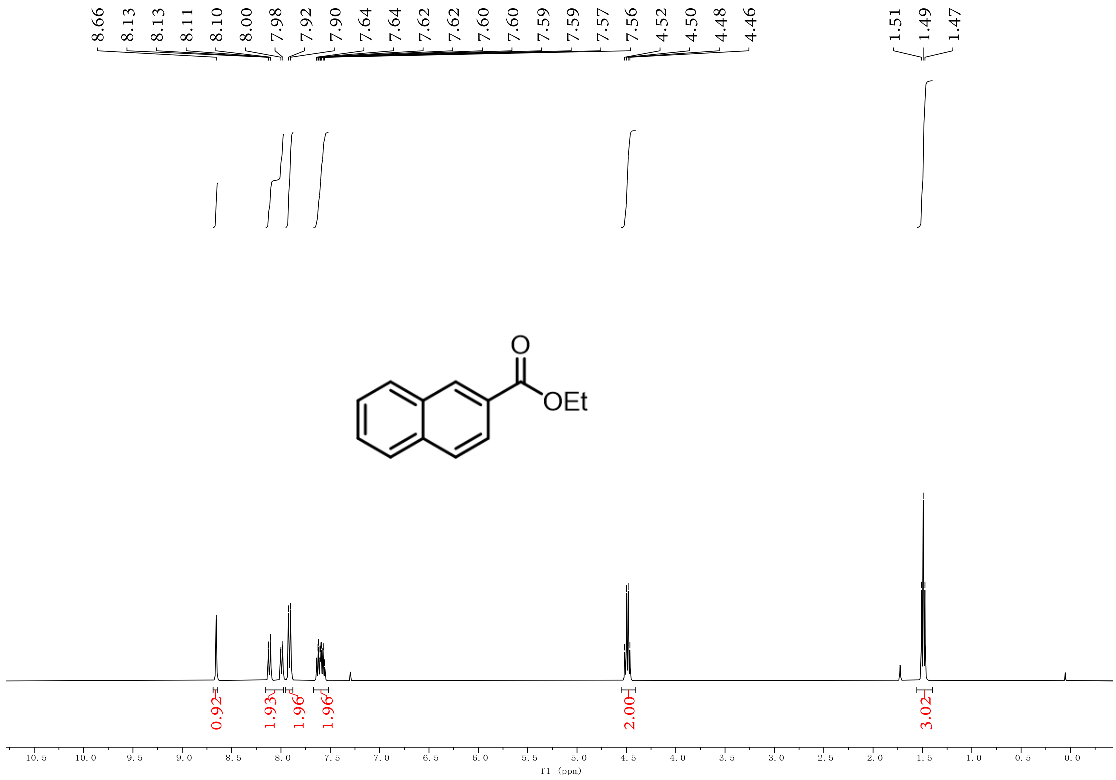

8.66
8.13
8.13
8.11
8.10
8.00
7.98
7.92
7.90
7.64
7.64
7.62
7.62
7.60
7.60
7.59
7.59
7.57
7.56
4.52
4.50
4.48
4.46
1.51
1.49
1.47
O
OEt
0.92
1.93
1.96
1.96
2.00
3.02
f1 (ppm)

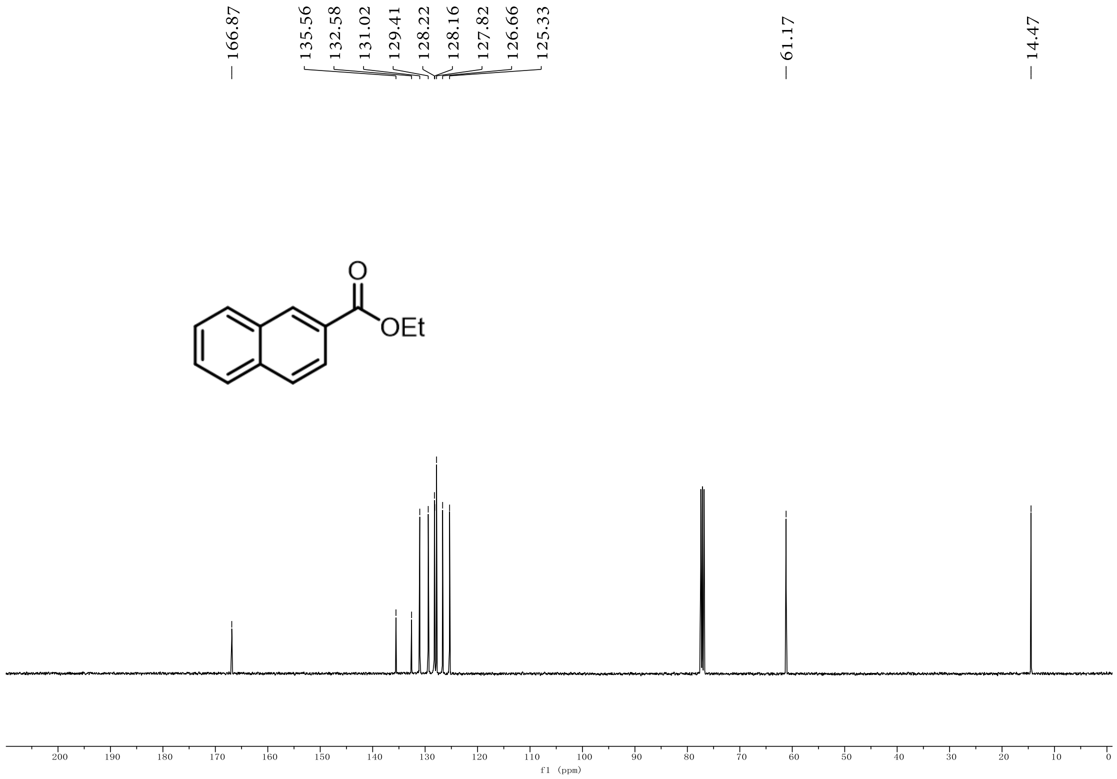

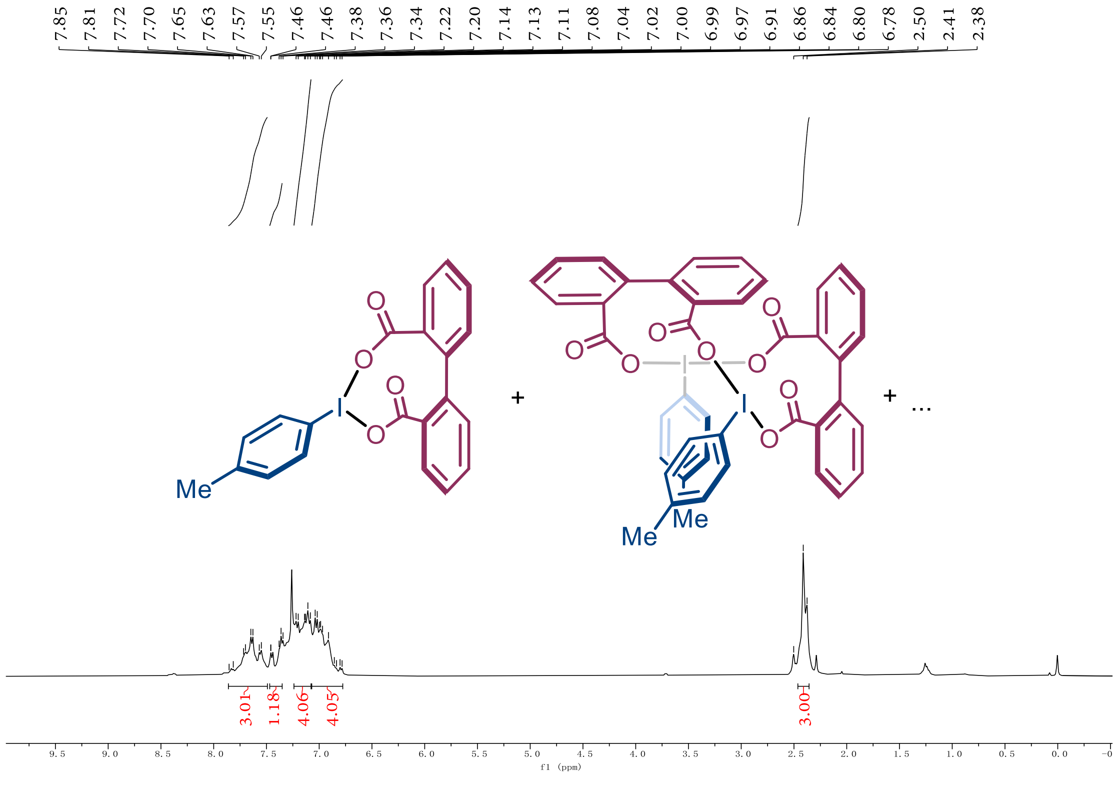

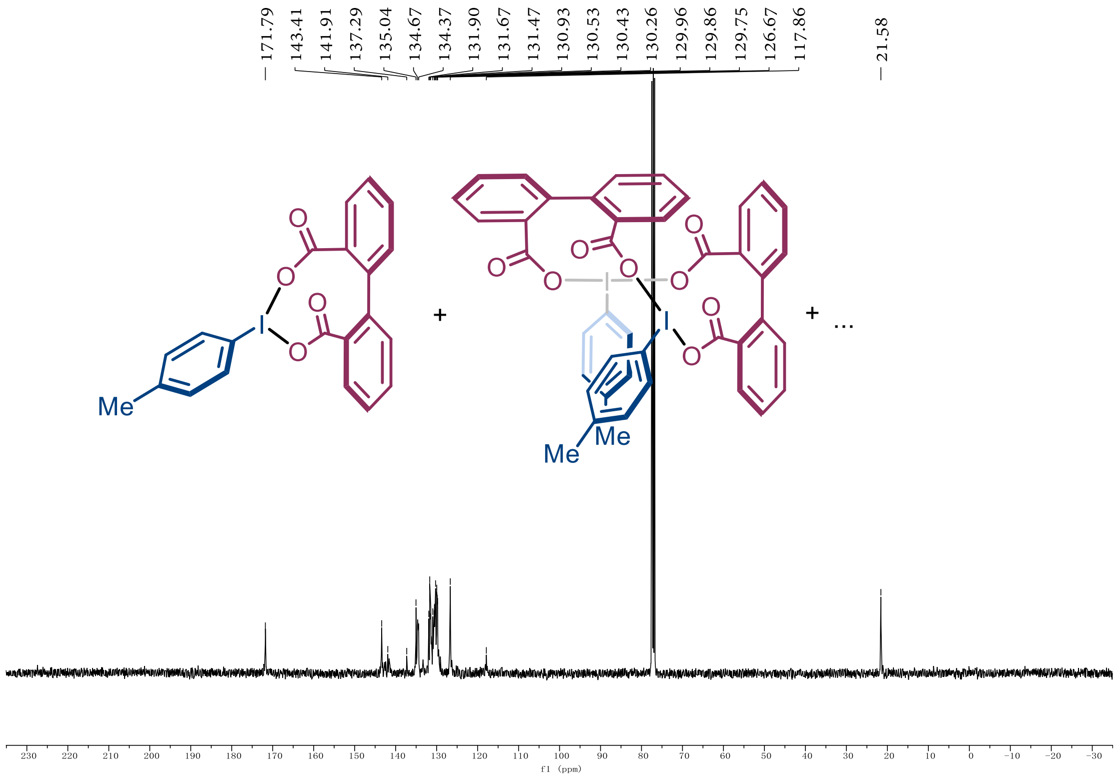
171.79
143.41
141.91
137.29
135.04
134.67
134.37
131.90
131.67
131.47
130.93
130.53
130.43
130.26
129.96
129.86
129.75
126.67
117.86
21.58
Me
Me
Me
f1 (ppm)

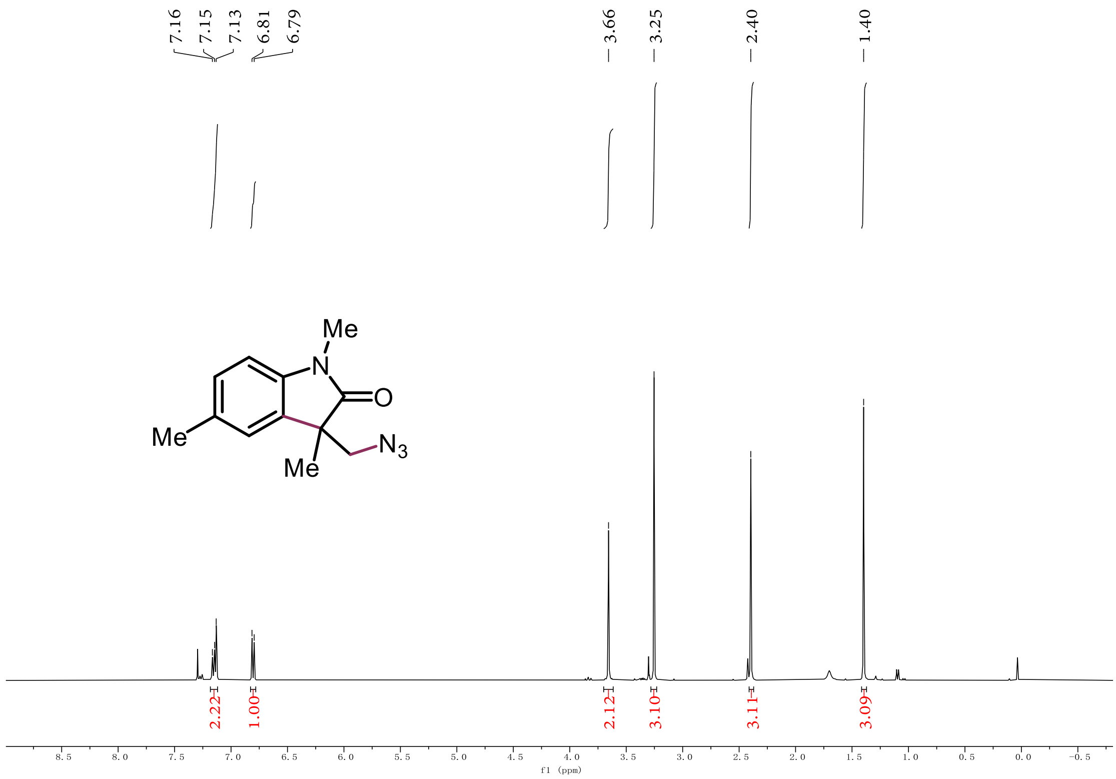
7.16
7.15
7.13
6.81
6.79
3.66
3.25
2.40
1.40
Me
N
O
Me
Me
N_3
2.22
1.00
2.12
3.10
3.11
3.09
f1 (ppm)

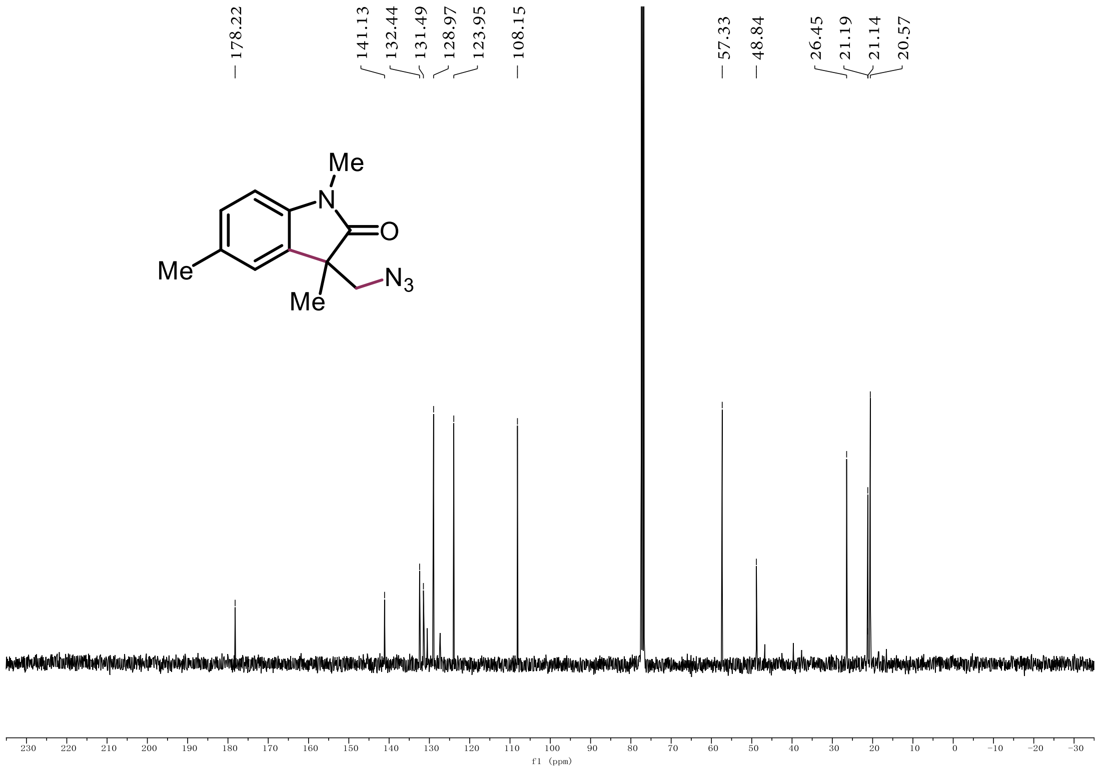
178.22
141.13
132.44
131.49
128.97
123.95
108.15
57.33
48.84
26.45
21.19
21.14
20.57
Me
N
O
Me
N3
Me
f1 (ppm)

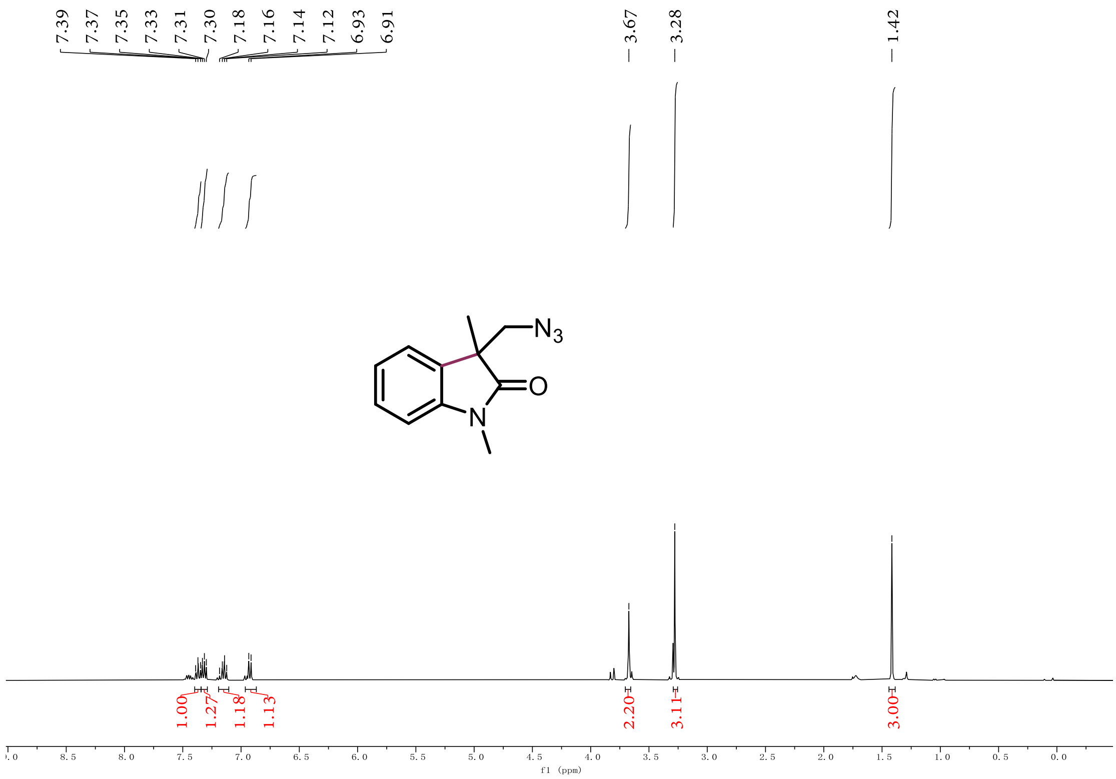
7.39
7.37
7.35
7.33
7.31
7.30
7.18
7.16
7.14
7.12
6.93
6.91
3.67
3.28
1.42
N3
O
N
1.00
1.27
1.18
1.13
2.20
3.11
3.00
f1 (ppm)

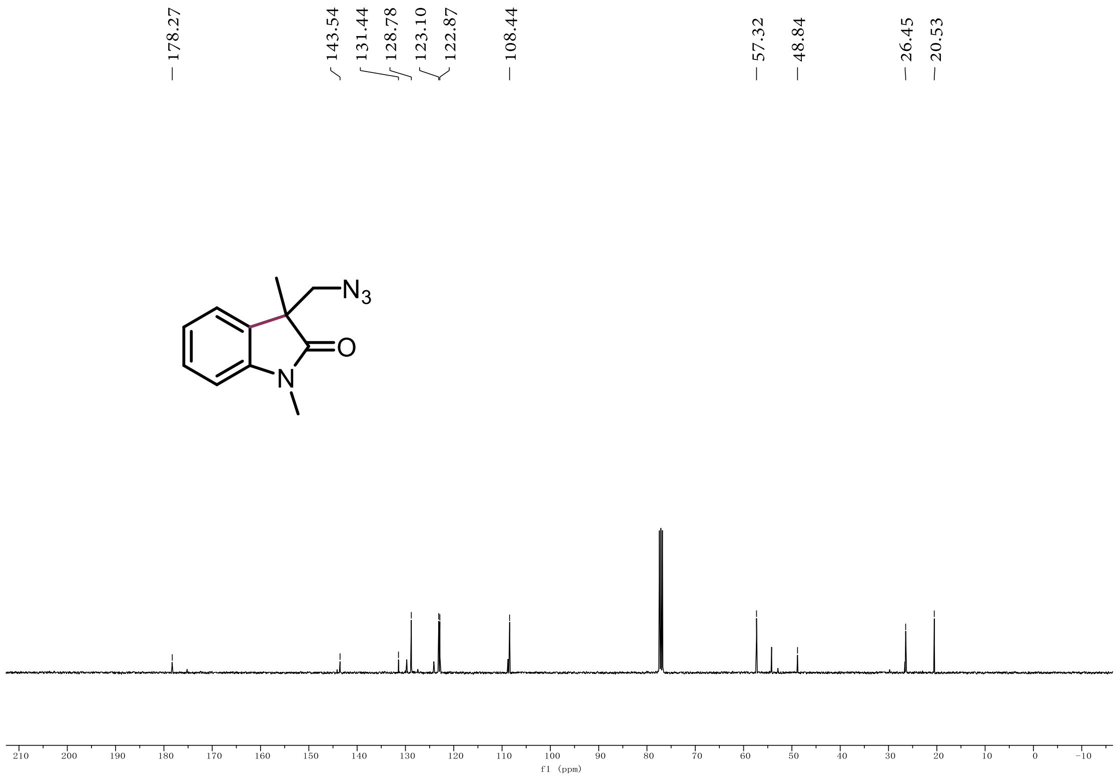

178.27
143.54
131.44
128.78
123.10
122.87
108.44
57.32
48.84
26.45
20.53
N3
O
N
f1 (ppm)

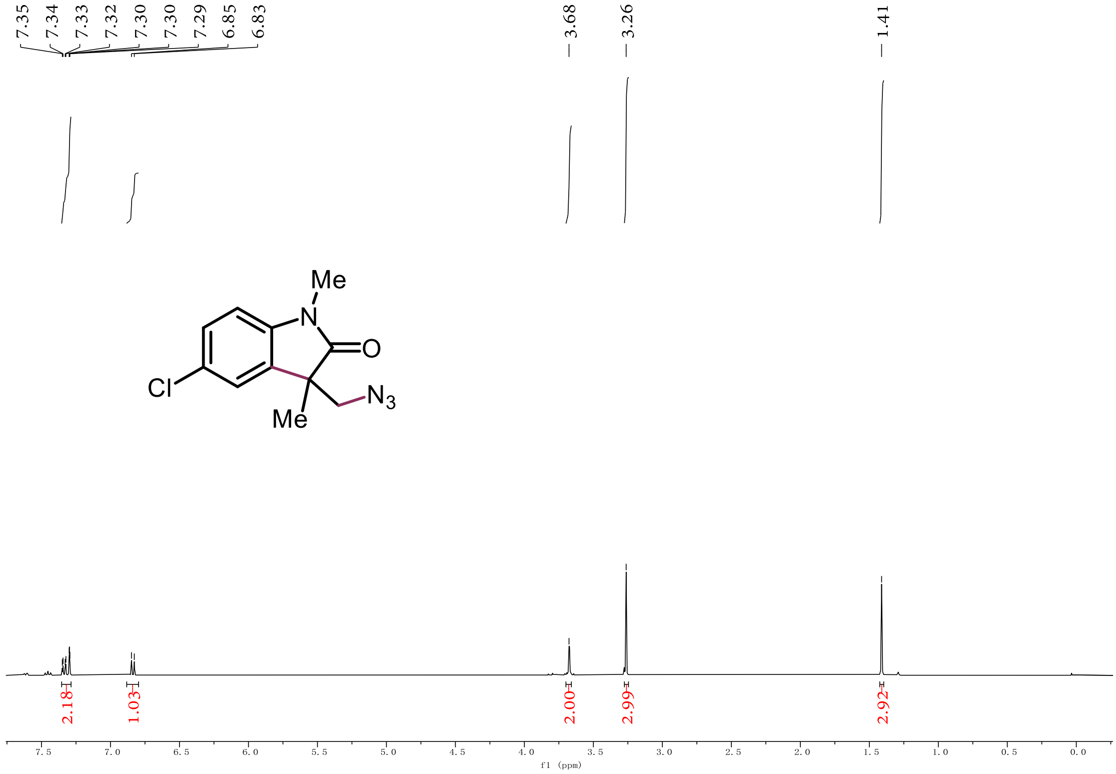

7.35
7.34
7.33
7.32
7.30
7.30
7.29
6.85
6.83
3.68
3.26
1.41
Me
N
O
Cl
Me
N3
2.18
1.03
2.00
2.99
2.92
f1 (ppm)

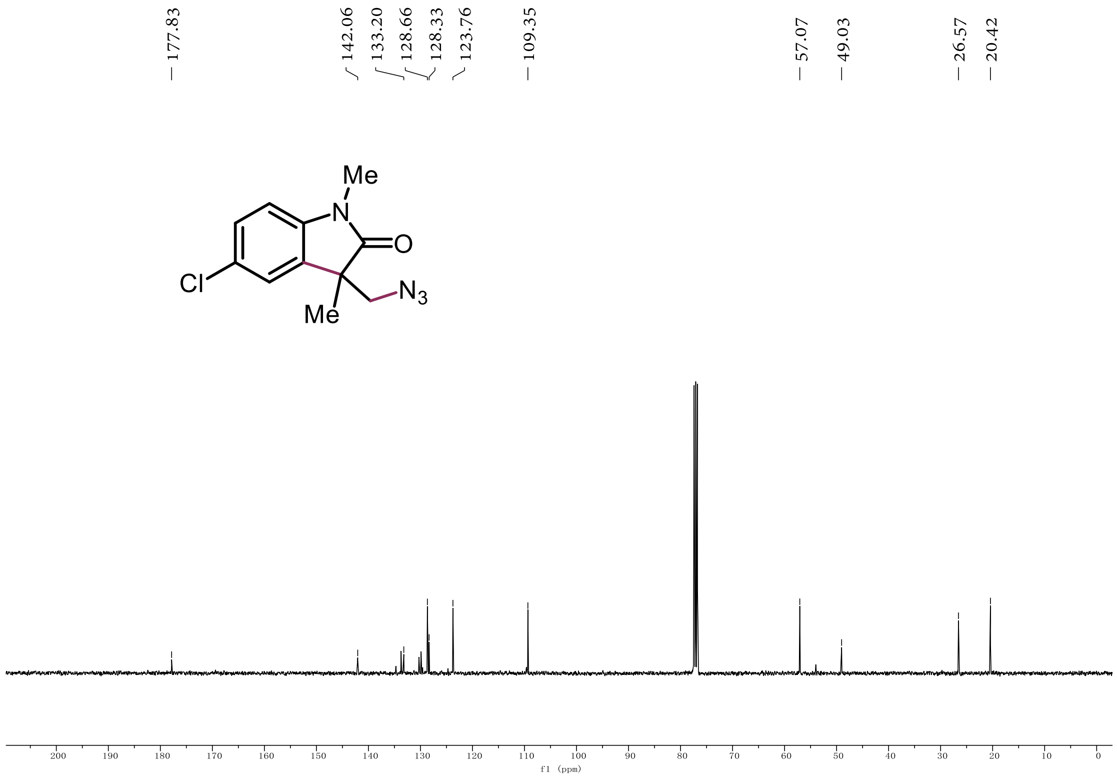
177.83
142.06
133.20
128.66
128.33
123.76
109.35
57.07
49.03
26.57
20.42
Me
N
O
Cl
N3
Me
200 190 180 170 160 150 140 130 120 110 100 90 80 70 60 50 40 30 20 10 0
f1 (ppm)

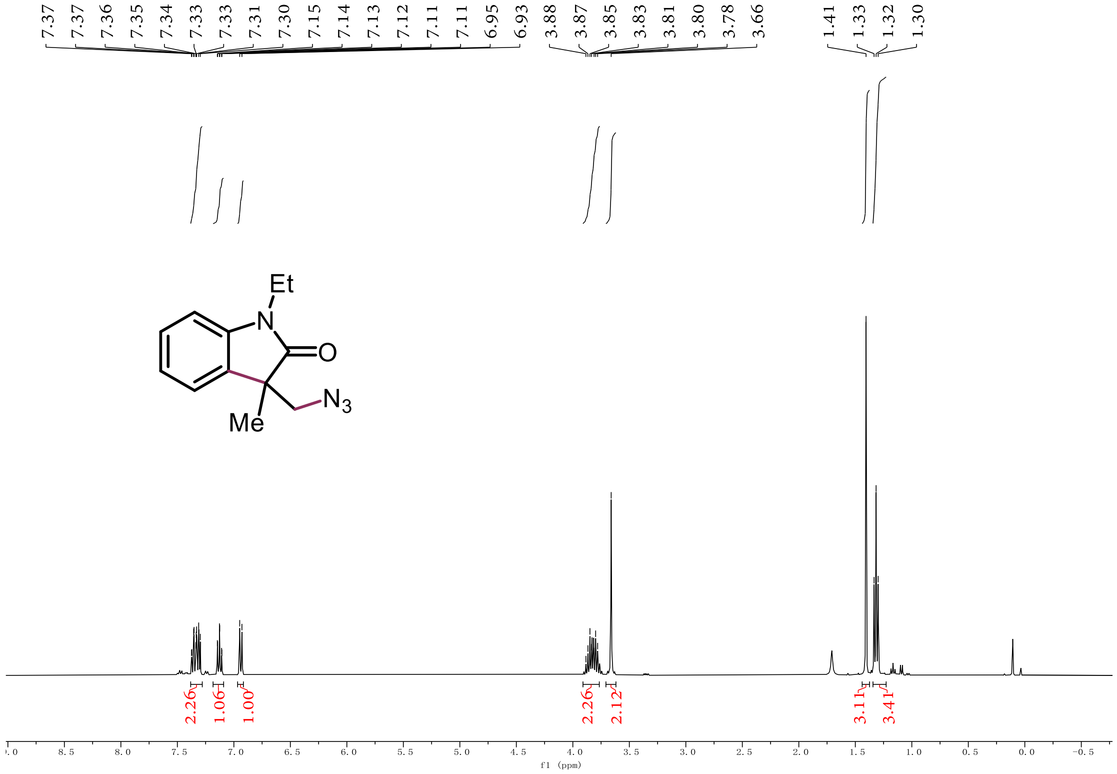
7.37 7.37 7.36 7.35 7.34 7.33 7.33 7.31 7.30 7.15 7.14 7.13 7.12 7.11 7.11 6.95 6.93 3.88 3.87 3.85 3.83 3.81 3.80 3.78 3.66 1.41 1.33 1.32 1.30
Et
N
O
N3
Me
2.26
1.06
1.00
2.26
2.12
3.11
3.41
8.5 8.0 7.5 7.0 6.5 6.0 5.5 5.0 4.5 4.0 3.5 3.0 2.5 2.0 1.5 1.0 0.5 0.0 -0.5
f1 (ppm)

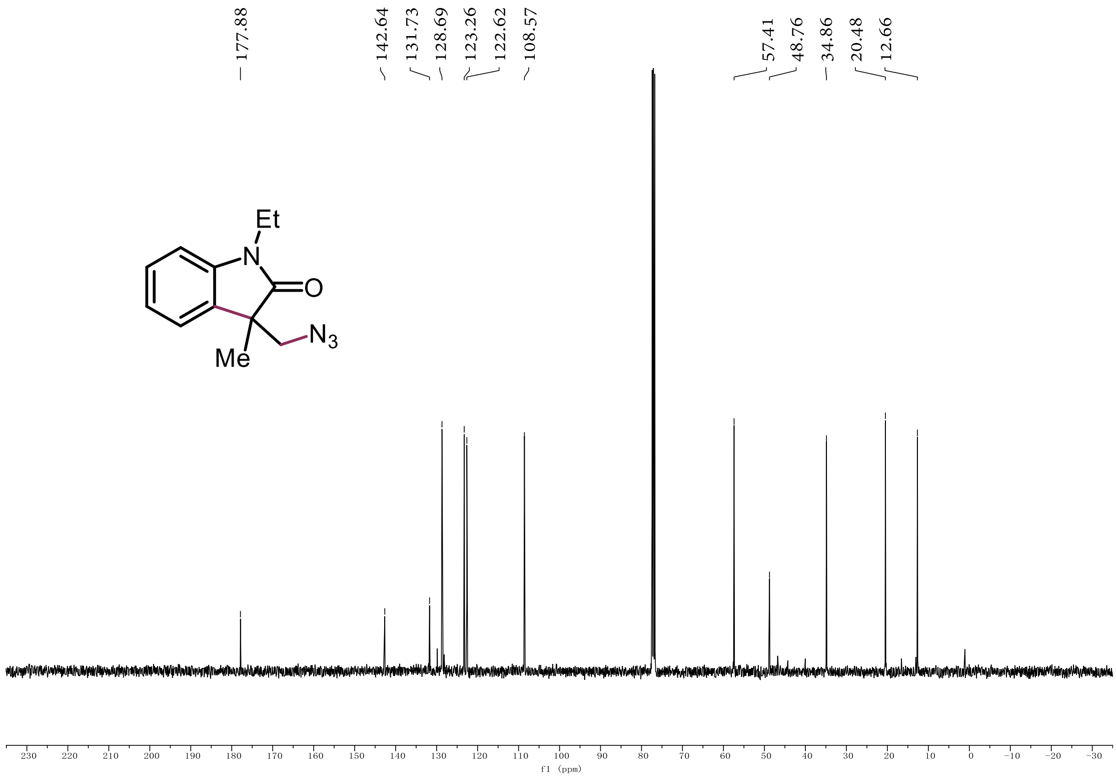
177.88
142.64
131.73
128.69
123.26
122.62
108.57
57.41
48.76
34.86
20.48
12.66
Et
N
O
Me
N_3
f1 (ppm)

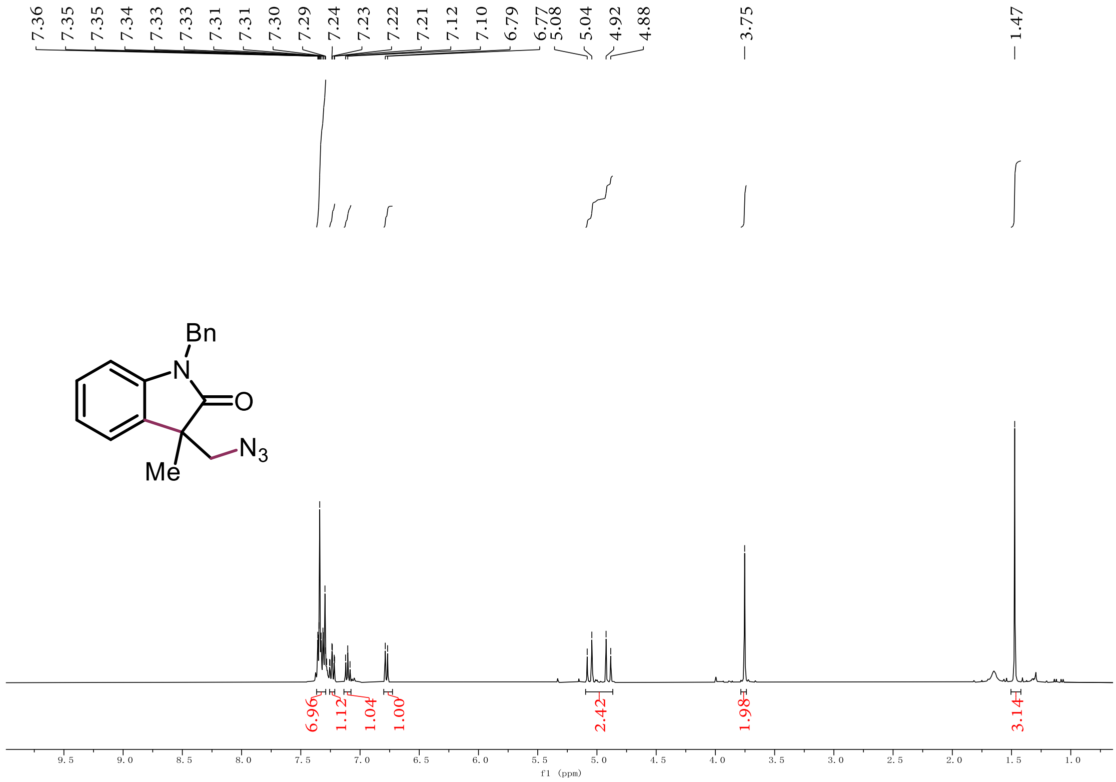
7.36
7.35
7.35
7.34
7.33
7.33
7.31
7.31
7.30
7.29
7.24
7.23
7.22
7.21
7.12
7.10
6.79
6.77
5.08
5.04
4.92
4.88
3.75
1.47
Bn
N
O
Me
N_3
6.96
1.12
1.04
1.00
2.42
1.98
3.14
f1 (ppm)

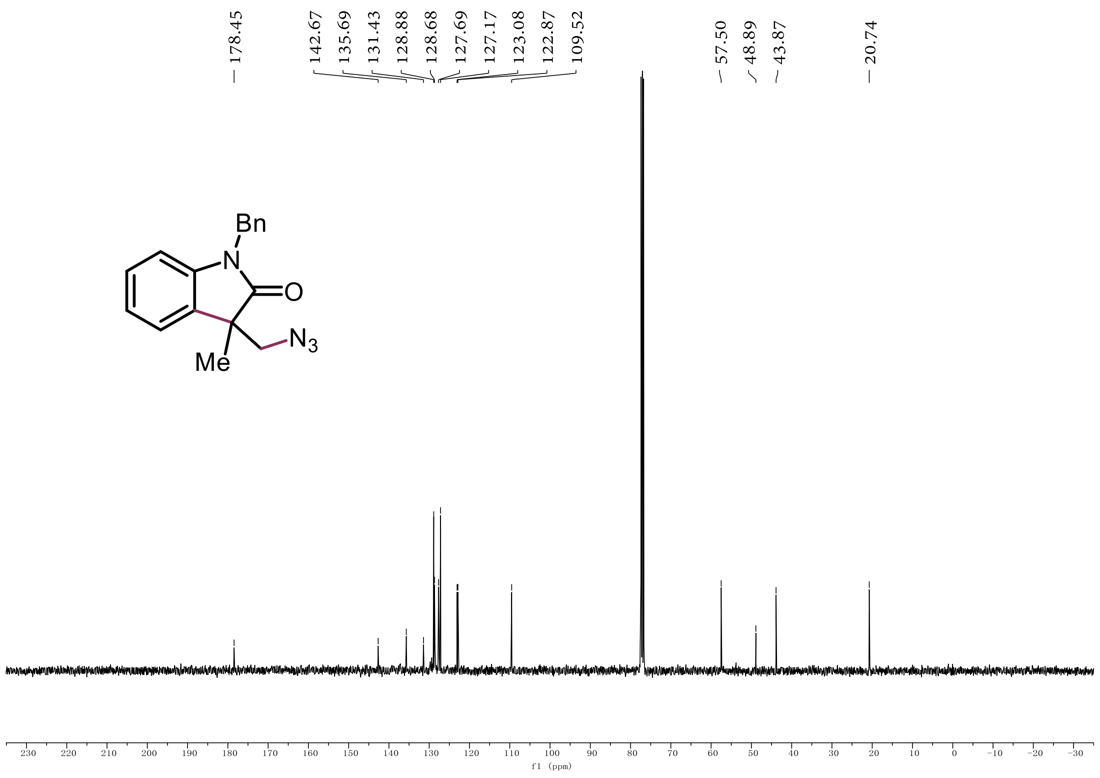
178.45
142.67
135.69
131.43
128.88
128.68
127.69
127.17
123.08
122.87
109.52
57.50
48.89
43.87
20.74
Bn
N
O
N3
Me
f1 (ppm)

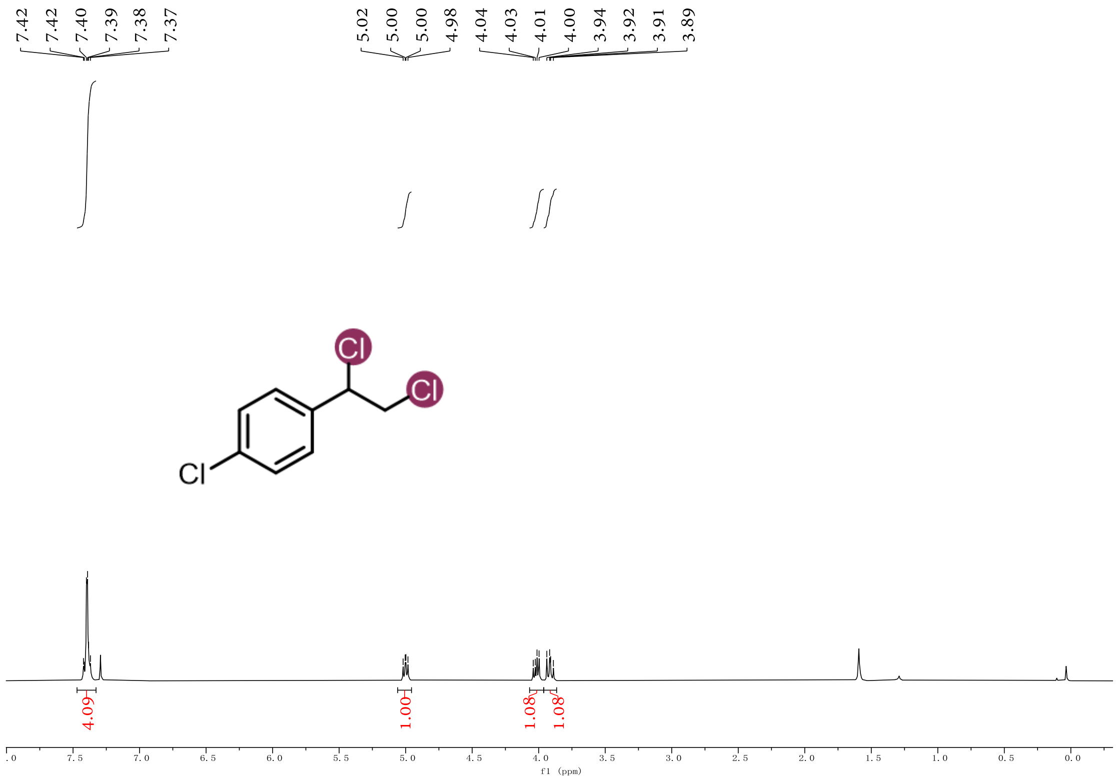
7.42
7.42
7.40
7.39
7.38
7.37
5.02
5.00
5.00
4.98
4.04
4.03
4.01
4.00
3.94
3.92
3.91
3.89
Cl
Cl
Cl
4.09
1.00
1.08
1.08
f1 (ppm)

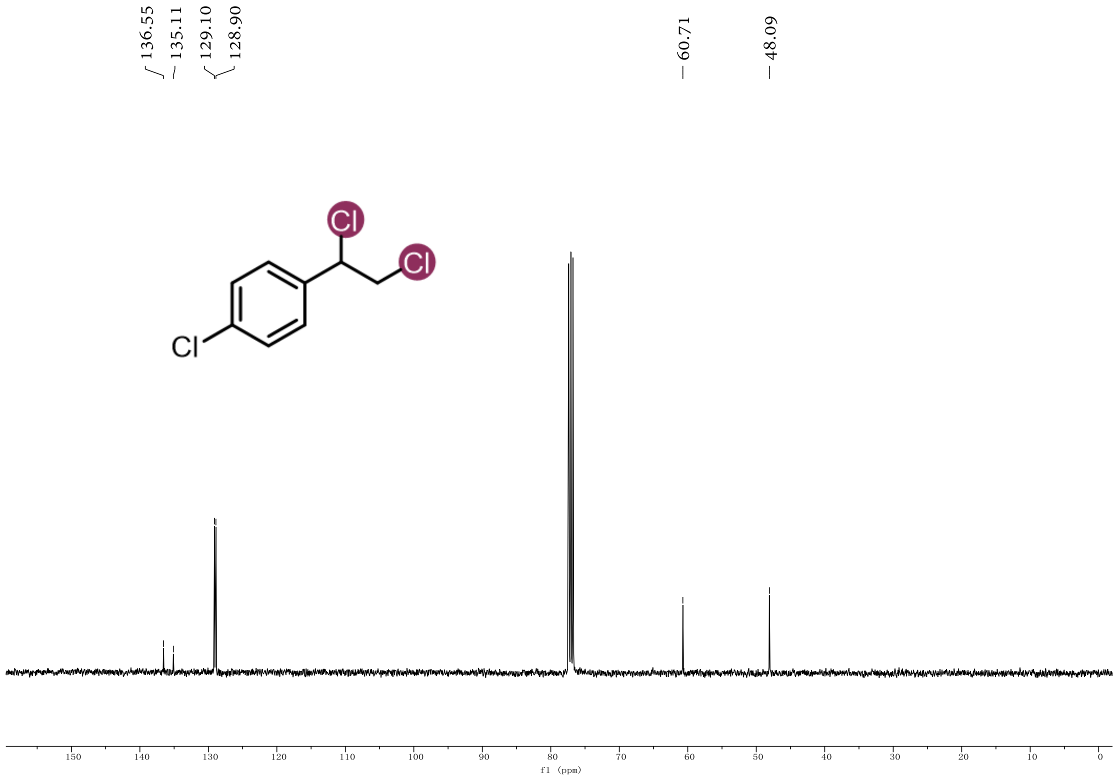
136.55
135.11
129.10
128.90
60.71
48.09
Cl
Cl
Cl
f1 (ppm)

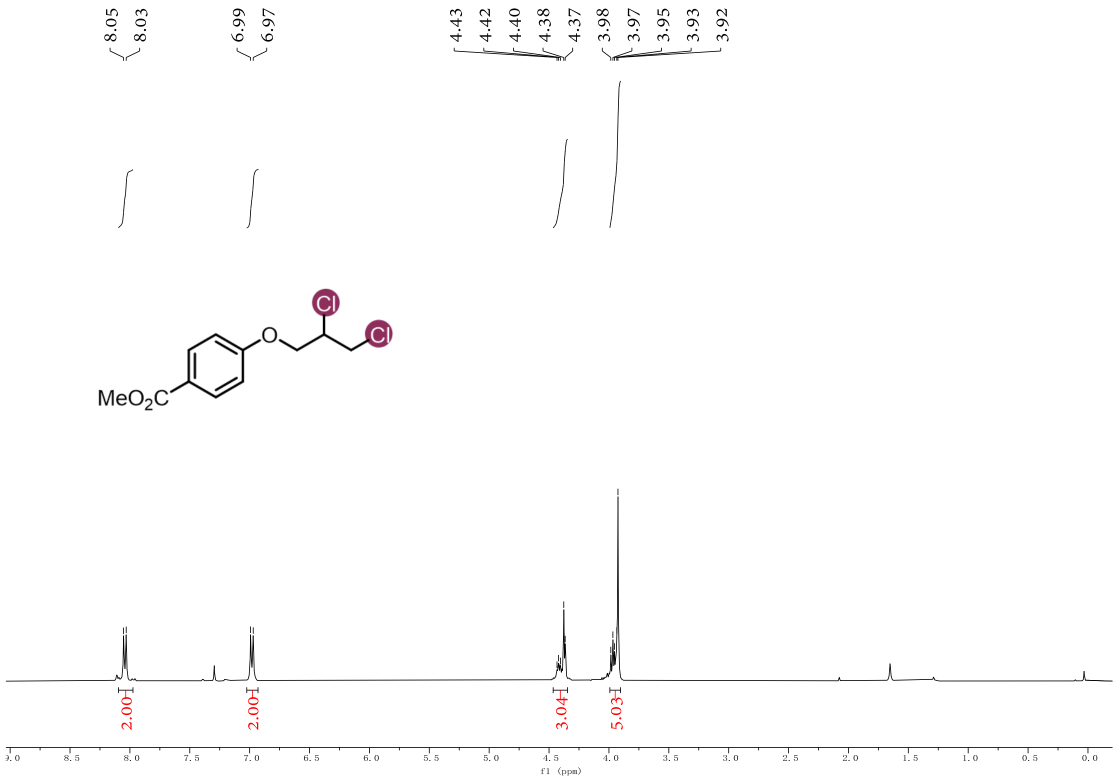
8.05
8.03
6.99
6.97
4.43
4.42
4.40
4.38
4.37
3.98
3.97
3.95
3.93
3.92
Cl
Cl
O
MeO2C
2.00
2.00
3.04
5.03
f1 (ppm)

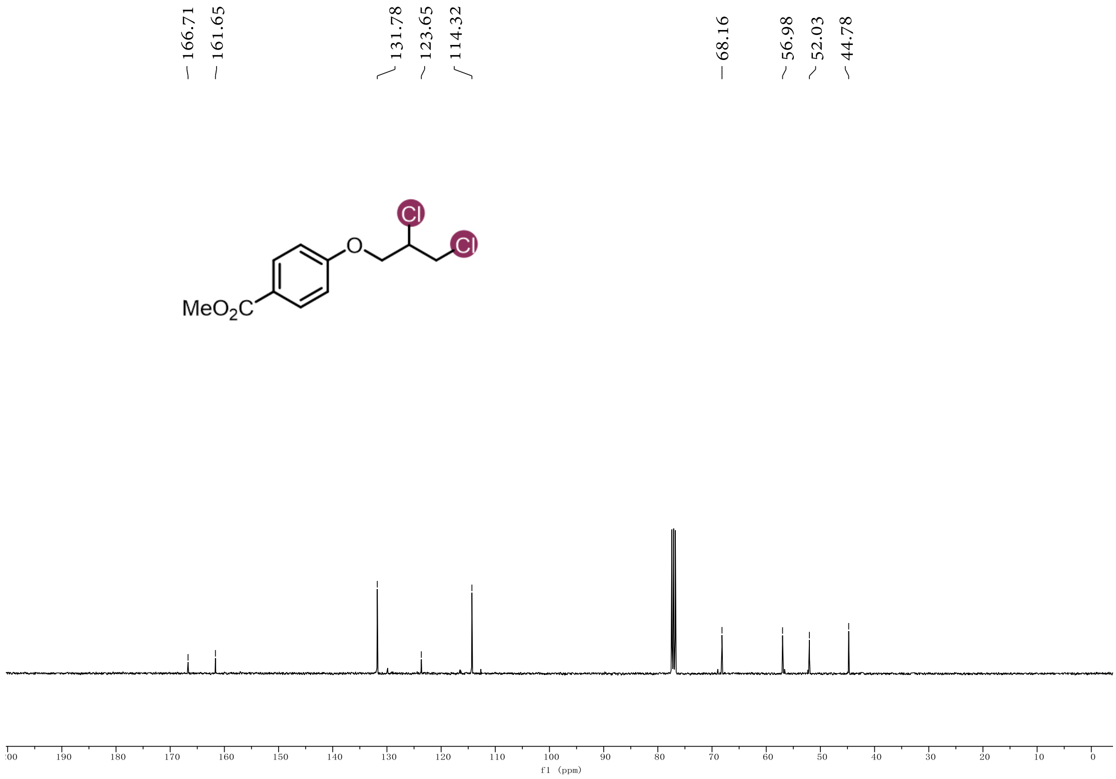
166.71
161.65
131.78
123.65
114.32
68.16
56.98
52.03
44.78
MeO2C
O
Cl
Cl
f1 (ppm)

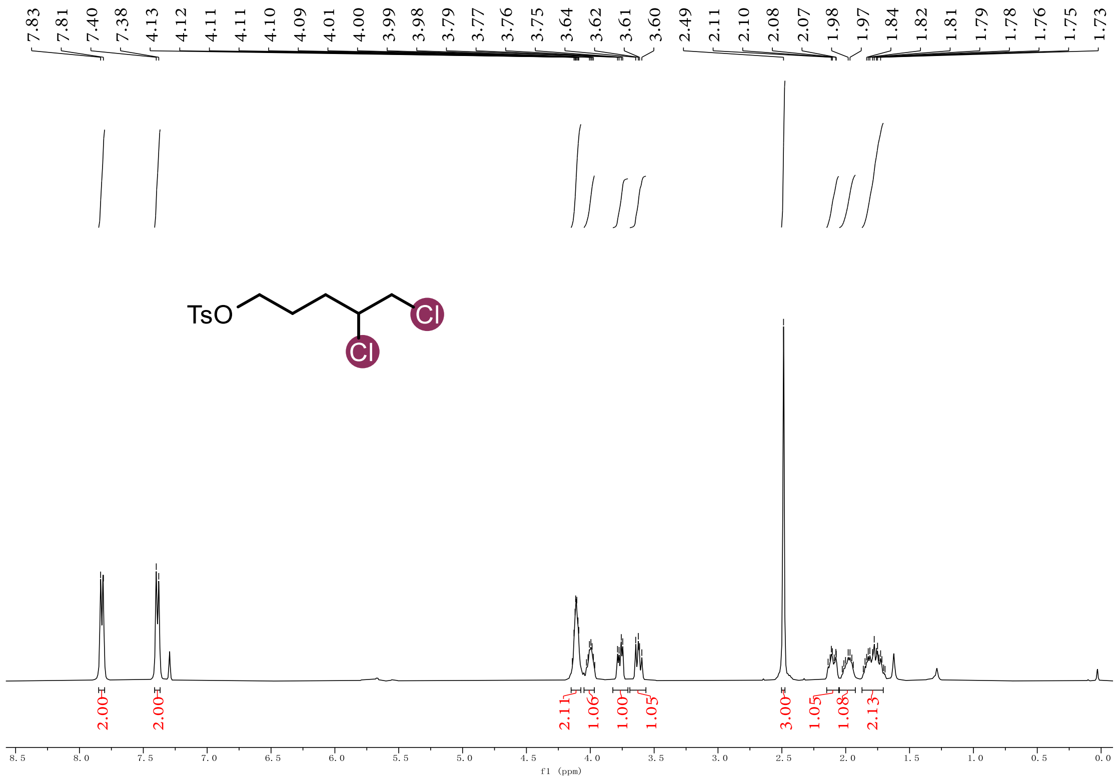
7.83
7.81
7.40
7.38
4.13
4.12
4.11
4.11
4.10
4.09
4.01
4.00
3.99
3.98
3.79
3.77
3.76
3.75
3.64
3.62
3.61
3.60
2.49
2.11
2.10
2.08
2.07
1.98
1.97
1.84
1.82
1.81
1.79
1.78
1.76
1.75
1.73
TsO
Cl
Cl
2.00
2.00
2.11
1.06
1.00
1.05
3.00
1.05
1.08
2.13
f1 (ppm)

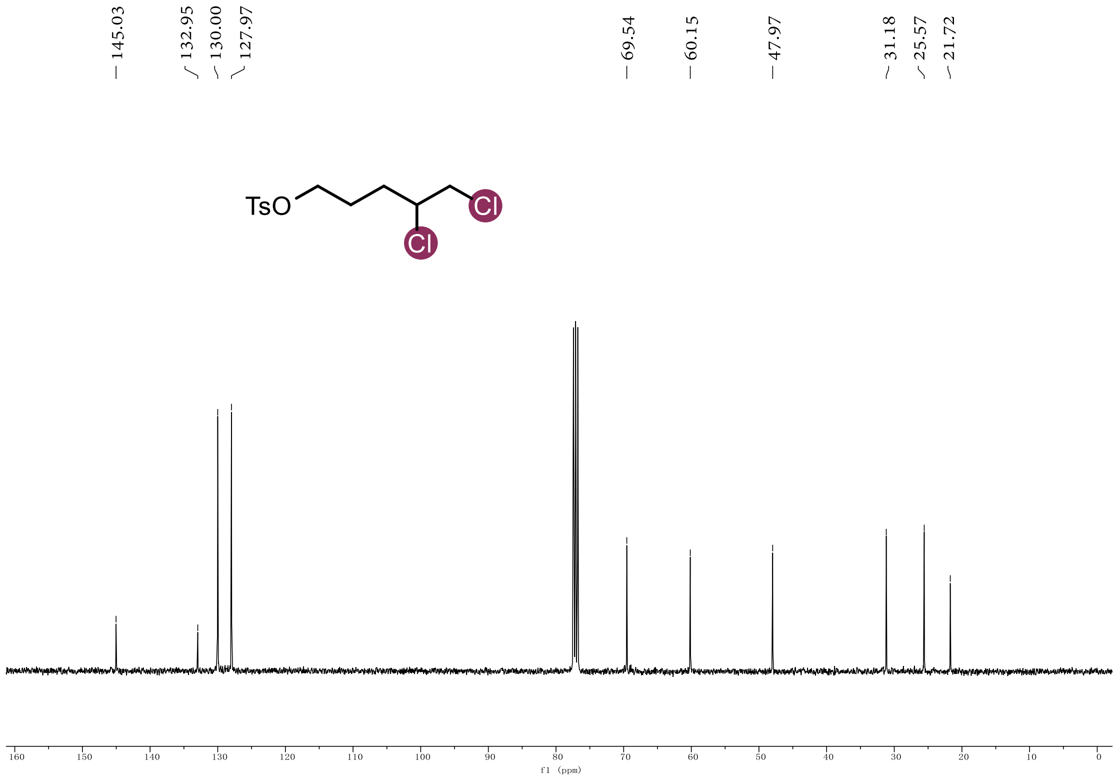
145.03
132.95
130.00
127.97
69.54
60.15
47.97
31.18
25.57
21.72
TsO
Cl
Cl
160 150 140 130 120 110 100 90 80 70 60 50 40 30 20 10 0
f1 (ppm)

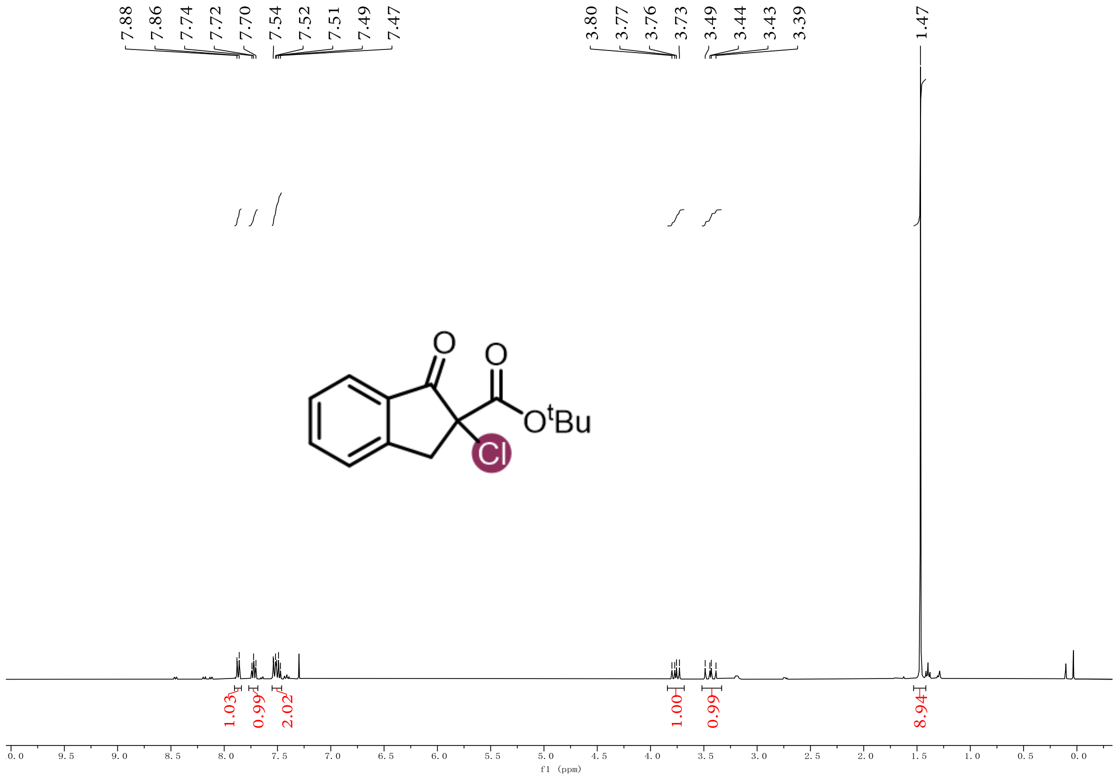
7.88
7.86
7.74
7.72
7.70
7.54
7.52
7.51
7.49
7.47
3.80
3.77
3.76
3.73
3.49
3.44
3.43
3.39
1.47
O
O
O^{t}Bu
Cl
1.03
0.99
2.02
1.00
0.99
8.94
10.0 9.5 9.0 8.5 8.0 7.5 7.0 6.5 6.0 5.5 5.0 4.5 4.0 3.5 3.0 2.5 2.0 1.5 1.0 0.5 0.0
f1 (ppm)

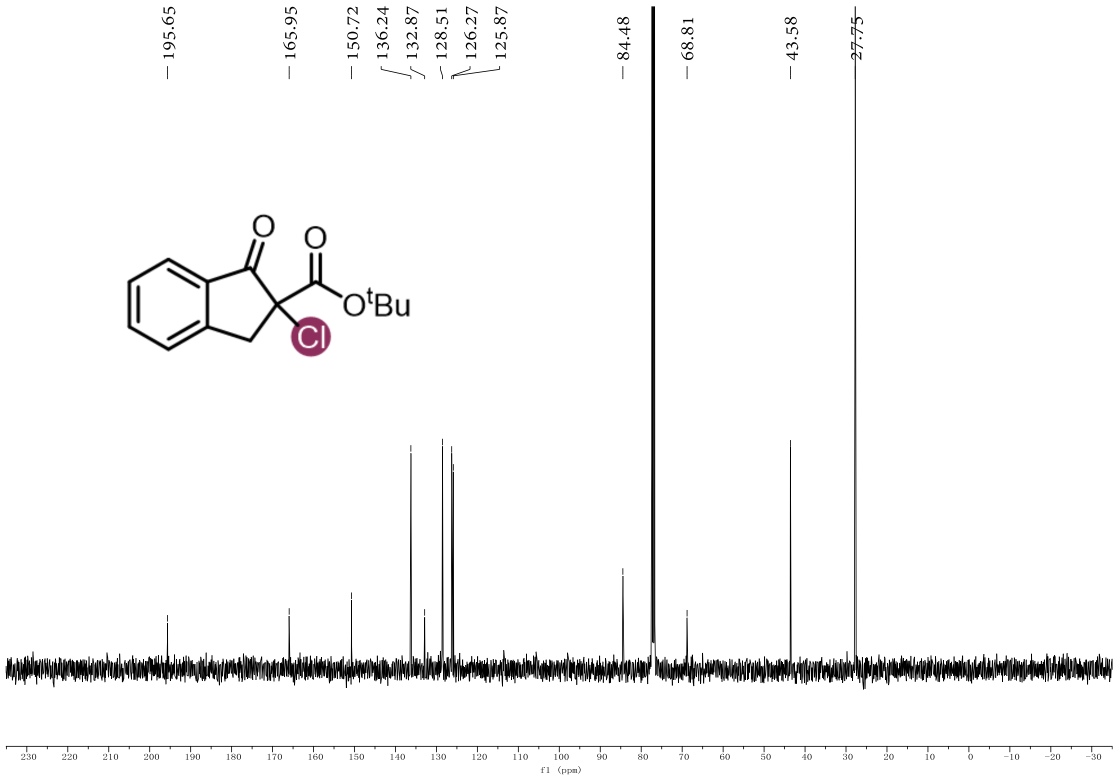
195.65
165.95
150.72
136.24
132.87
128.51
126.27
125.87
84.48
68.81
43.58
27.75
O
O
O^{t}Bu
Cl
f1 (ppm)

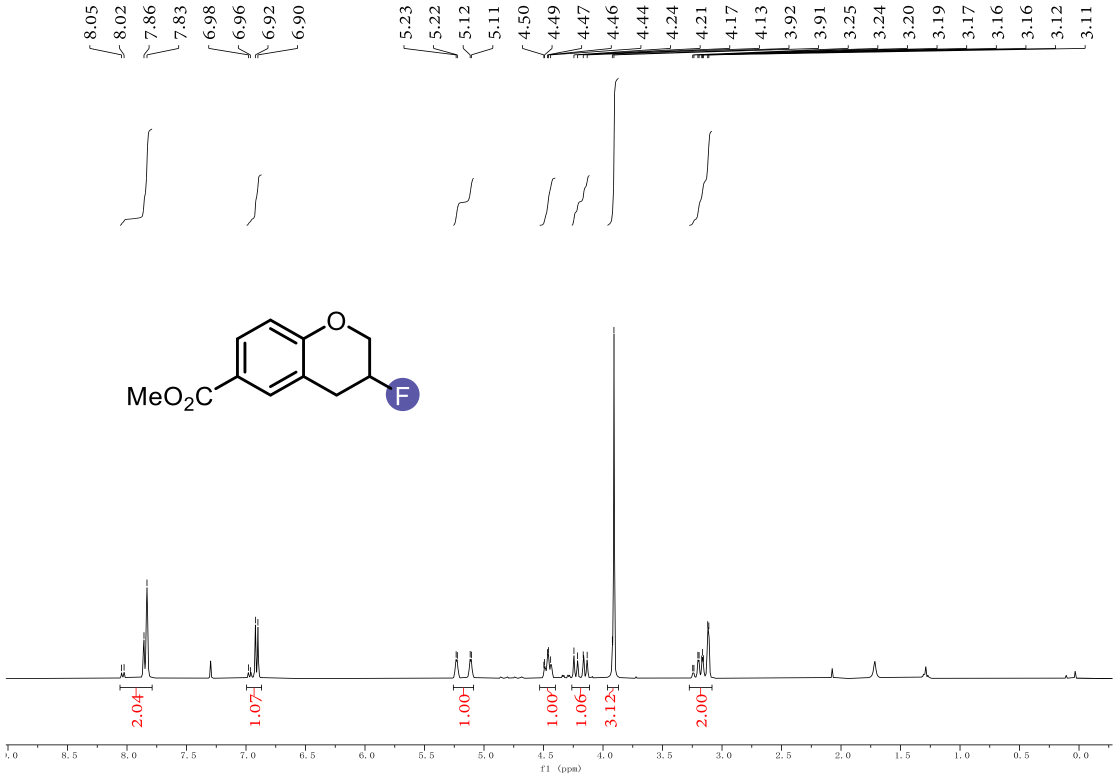
8.05
8.02
7.86
7.83
6.98
6.96
6.92
6.90
5.23
5.22
5.12
5.11
4.50
4.49
4.47
4.46
4.44
4.24
4.21
4.17
4.13
3.92
3.91
3.25
3.24
3.20
3.19
3.17
3.16
3.16
3.12
3.11
O
MeO_2C
F
2.04
1.07
1.00
1.00
1.06
3.12
2.00
f1 (ppm)

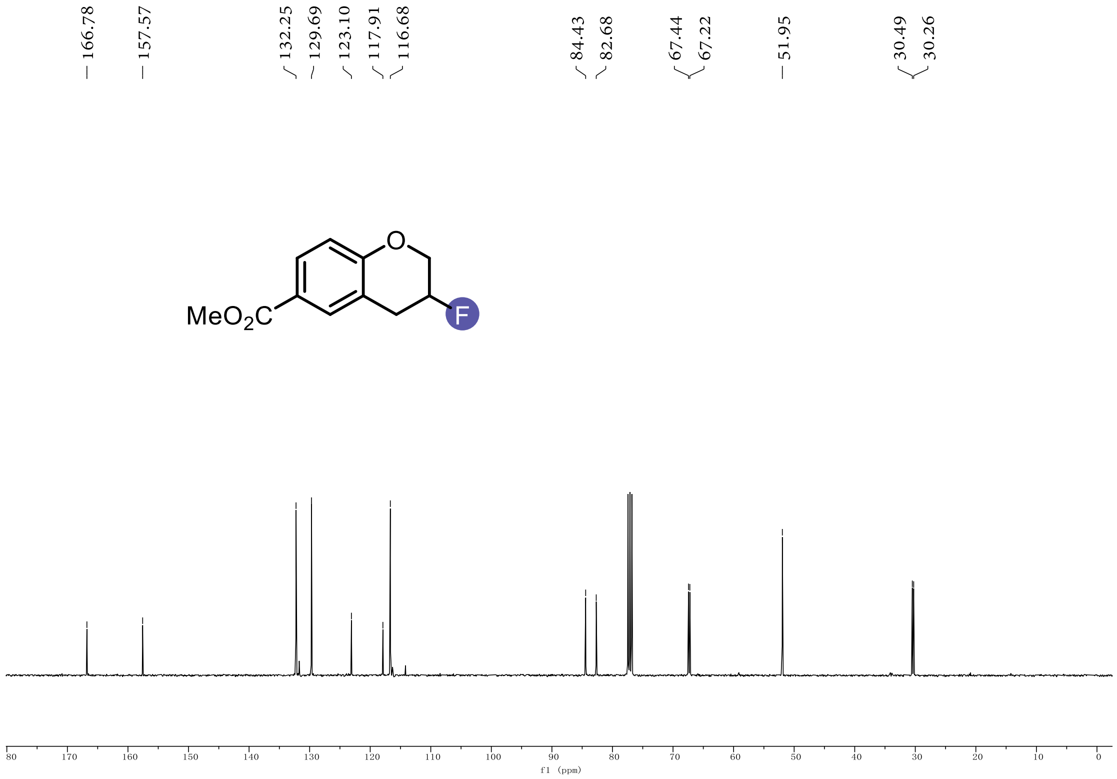
166.78
157.57
132.25
129.69
123.10
117.91
116.68
84.43
82.68
67.44
67.22
51.95
30.49
30.26
O
MeO2C
F
f1 (ppm)

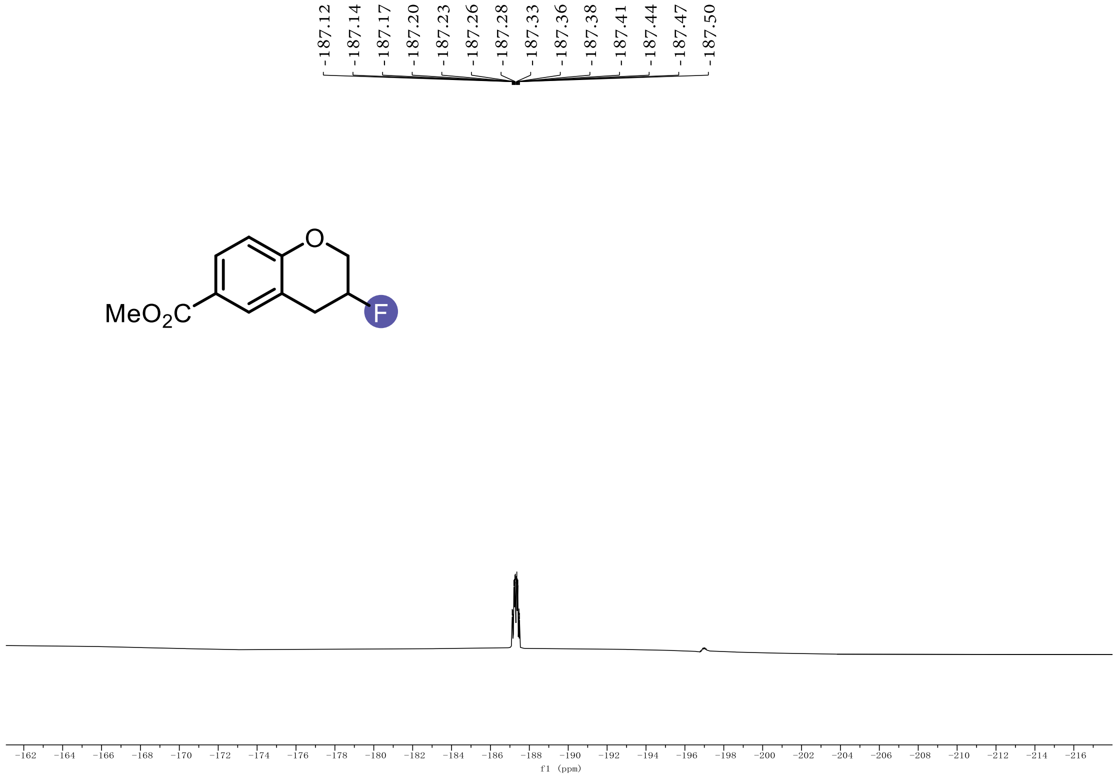
-187.12
-187.14
-187.17
-187.20
-187.23
-187.26
-187.28
-187.33
-187.36
-187.38
-187.41
-187.44
-187.47
-187.50
O
MeO2C
F
f1 (ppm)

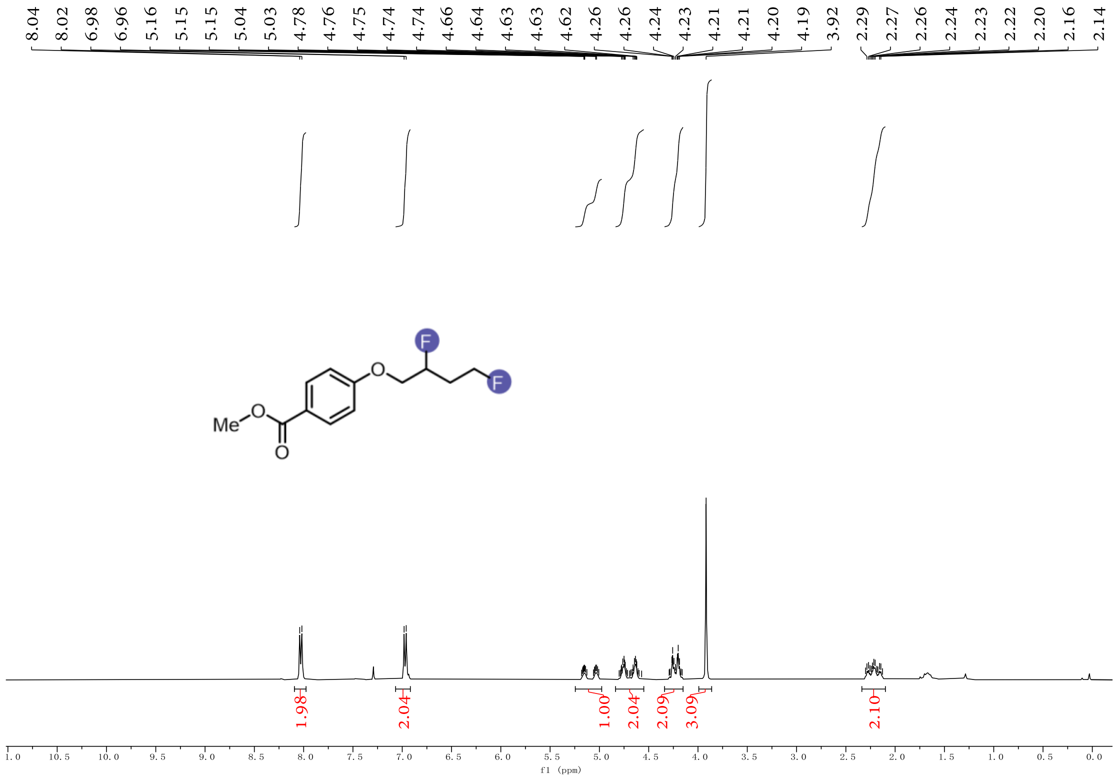
8.04 8.02 6.98 6.96 5.16 5.15 5.15 5.04 5.03 4.78 4.76 4.75 4.74 4.74 4.66 4.64 4.63 4.63 4.62 4.26 4.26 4.24 4.23 4.21 4.21 4.20 4.19 3.92 2.29 2.27 2.26 2.24 2.23 2.22 2.20 2.16 2.14
Me
O
O
O
F
F
1.98
2.04
1.00
2.04
2.09
3.09
2.10
f1 (ppm)

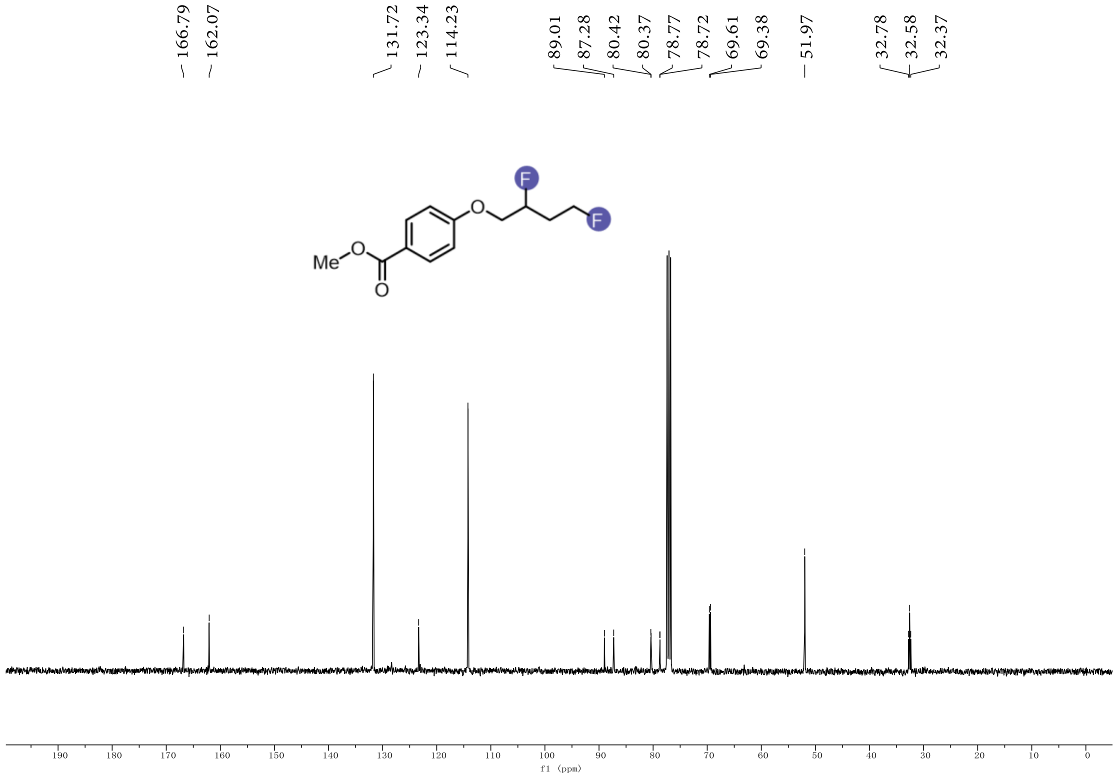
166.79 162.07 131.72 123.34 114.23 89.01 87.28 80.42 80.37 78.77 78.72 69.61 69.38 51.97 32.78 32.58 32.37
Me
O
O
O
F
F
f1 (ppm)

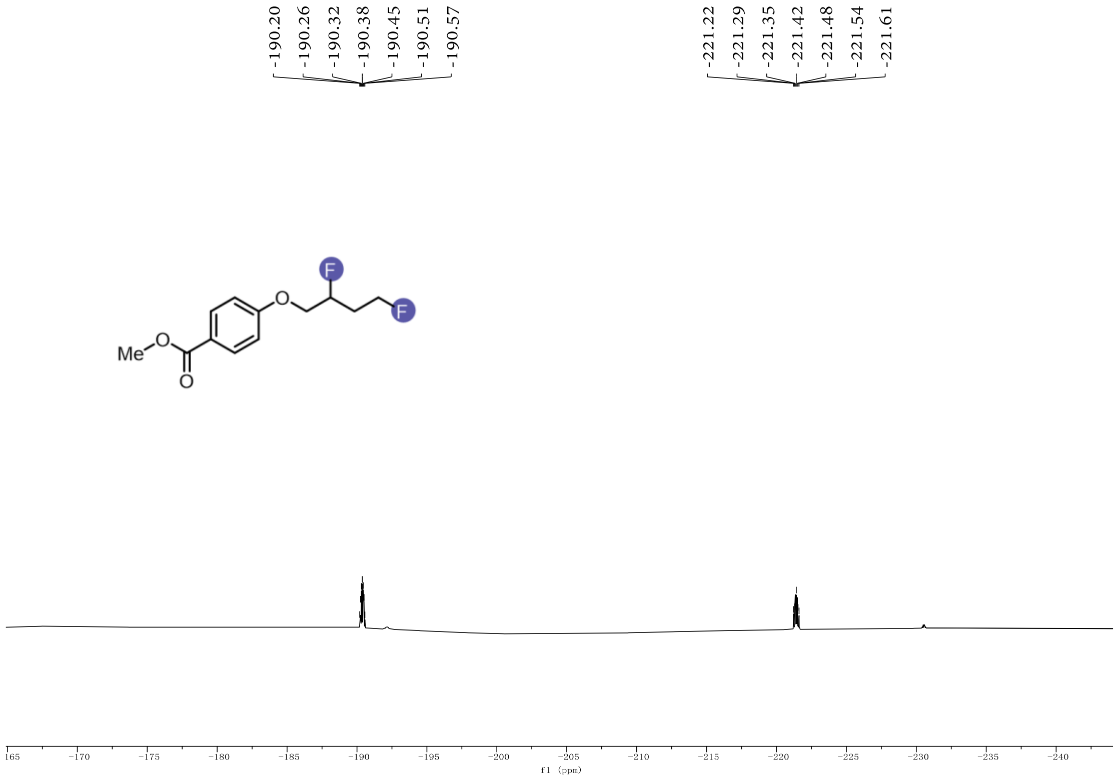
-190.20
-190.26
-190.32
-190.38
-190.45
-190.51
-190.57
-221.22
-221.29
-221.35
-221.42
-221.48
-221.54
-221.61
Me
O
O
O
F
F
-170 -175 -180 -185 -190 -195 -200 -205 -210 -215 -220 -225 -230 -235 -240
f1 (ppm)

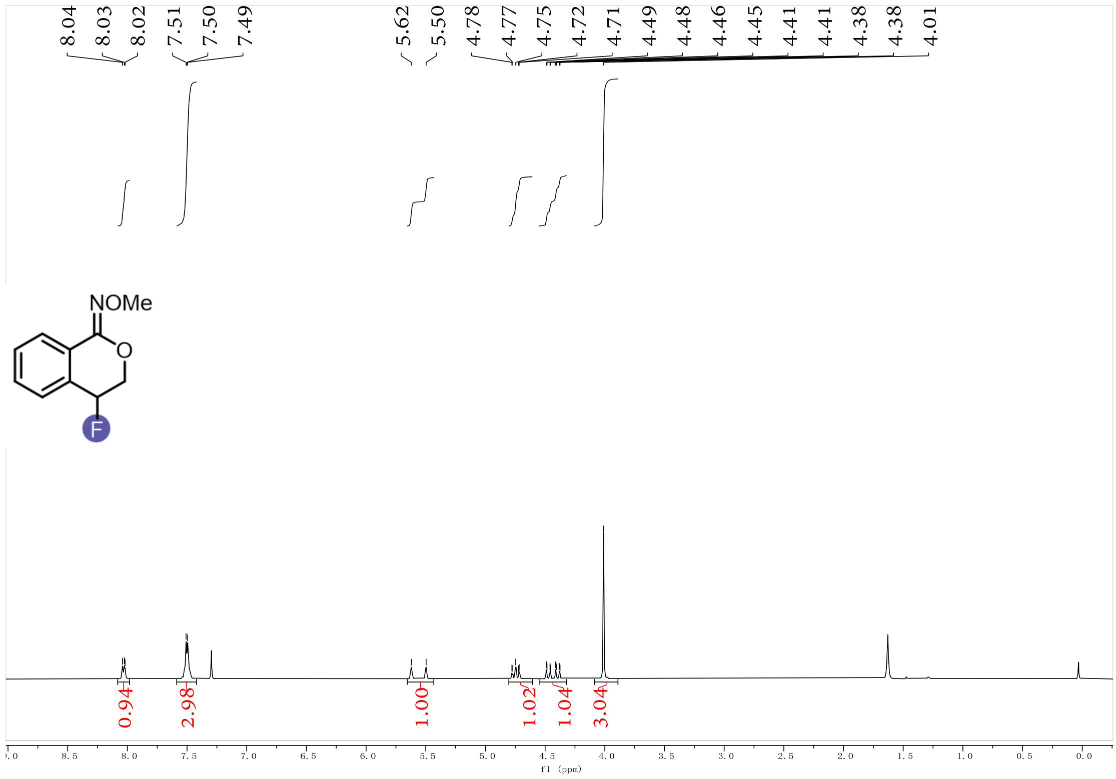
8.04
8.03
8.02
7.51
7.50
7.49
5.62
5.50
4.78
4.77
4.75
4.72
4.71
4.49
4.48
4.46
4.45
4.41
4.41
4.38
4.38
4.01
NOMe
O
F
0.94
2.98
1.00
1.02
1.04
3.04
8.5 8.0 7.5 7.0 6.5 6.0 5.5 5.0 4.5 4.0 3.5 3.0 2.5 2.0 1.5 1.0 0.5 0.0
f1 (ppm)

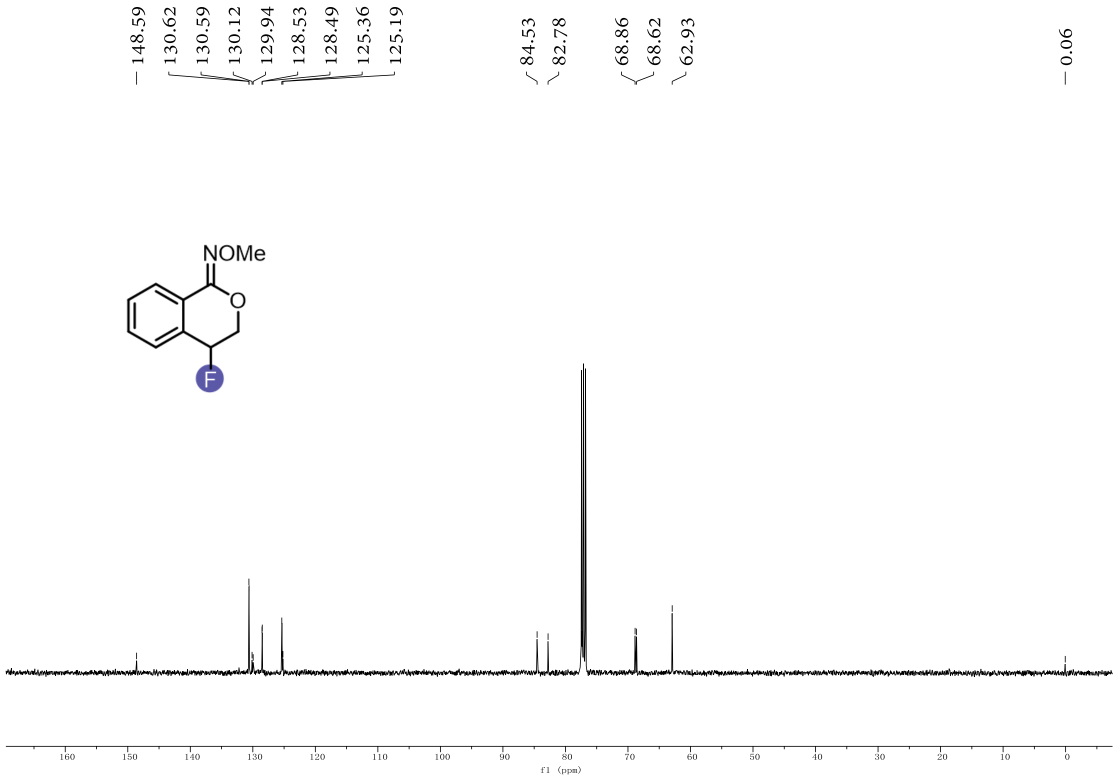
148.59
130.62
130.59
130.12
129.94
128.53
128.49
125.36
125.19
84.53
82.78
68.86
68.62
62.93
0.06
NOMe
O
F
160 150 140 130 120 110 100 90 80 70 60 50 40 30 20 10 0
f1 (ppm)

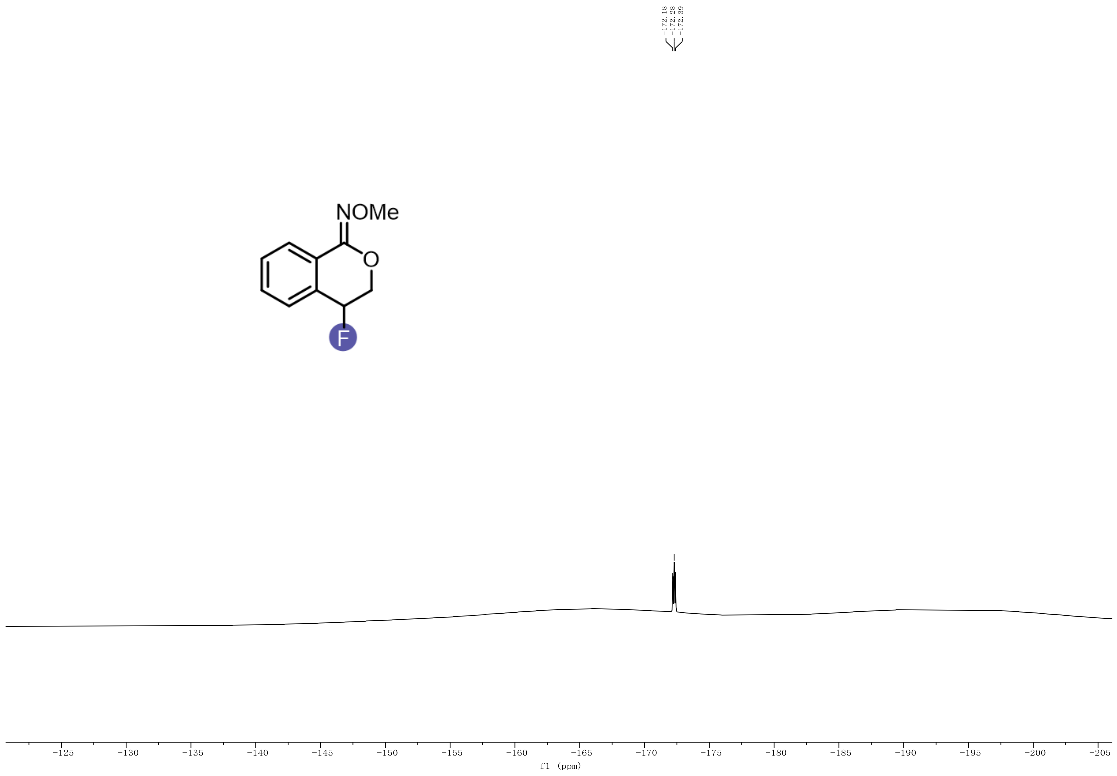
-172.18
-172.28
-172.39
NOMe
O
F
-125 -130 -135 -140 -145 -150 -155 -160 -165 -170 -175 -180 -185 -190 -195 -200 -205
f1 (ppm)

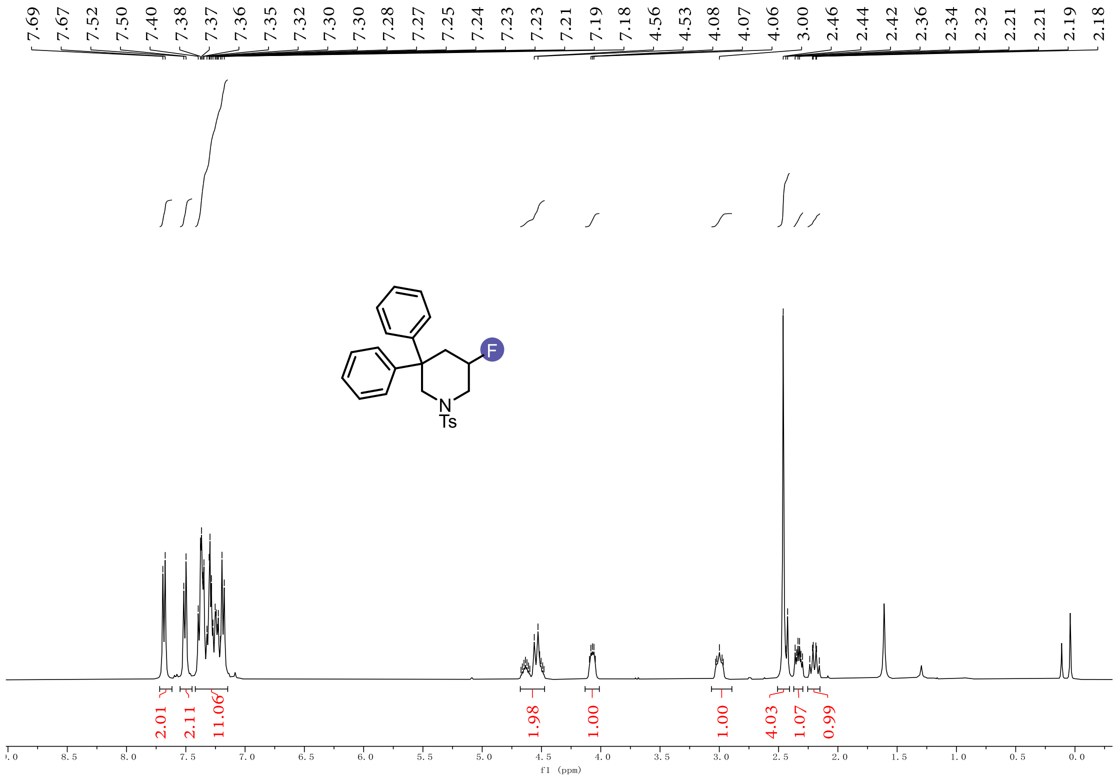
7.69
7.67
7.52
7.50
7.40
7.38
7.37
7.36
7.35
7.32
7.30
7.30
7.28
7.27
7.25
7.24
7.23
7.23
7.21
7.19
7.18
4.56
4.53
4.08
4.07
4.06
3.00
2.46
2.44
2.42
2.36
2.34
2.32
2.21
2.21
2.19
2.18
F
N
Ts
2.01
2.11
11.06
1.98
1.00
1.00
4.03
1.07
0.99
f1 (ppm)

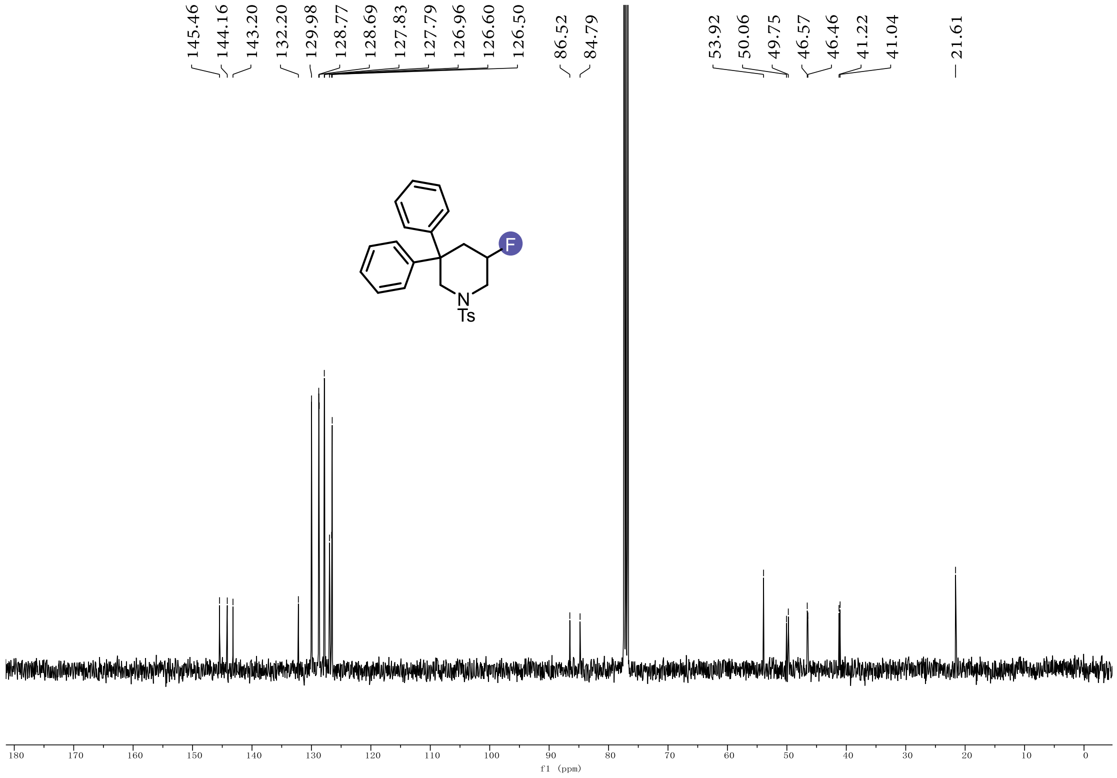
145.46
144.16
143.20
132.20
129.98
128.77
128.69
127.83
127.79
126.96
126.60
126.50
86.52
84.79
53.92
50.06
49.75
46.57
46.46
41.22
41.04
21.61
F
N
Ts
f1 (ppm)

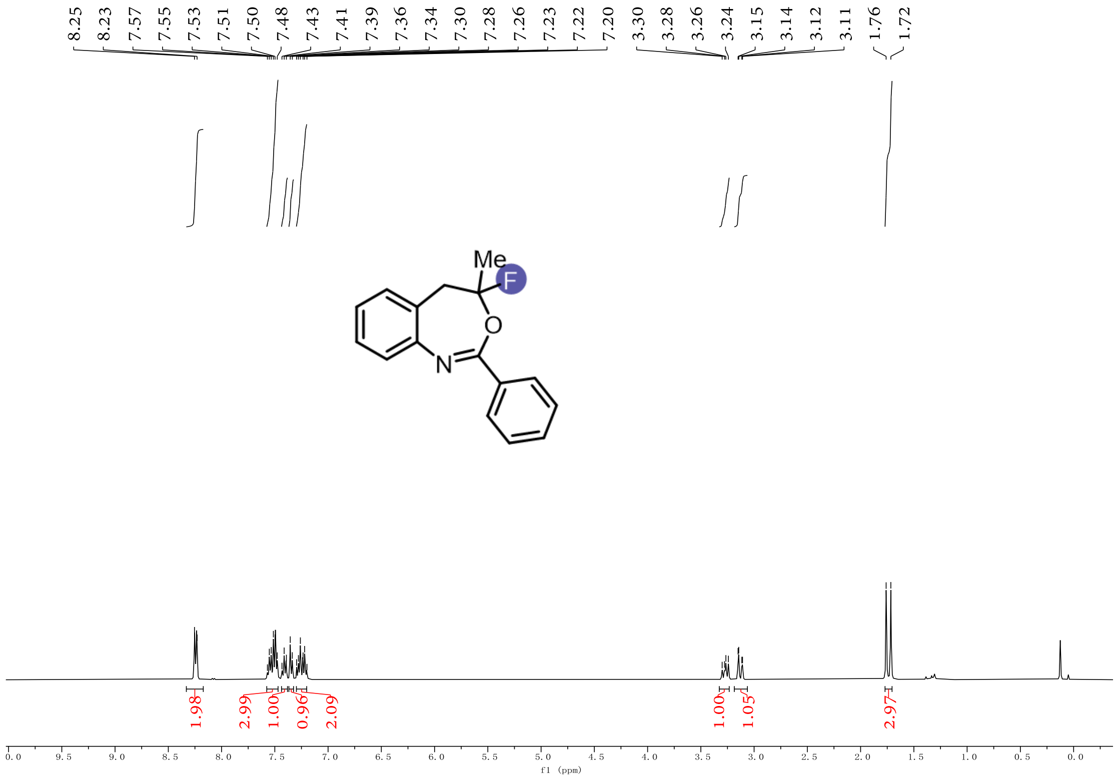
8.25 8.23 7.57 7.55 7.53 7.51 7.50 7.48 7.43 7.41 7.39 7.36 7.34 7.30 7.28 7.26 7.23 7.22 7.20 3.30 3.28 3.26 3.24 3.15 3.14 3.12 3.11 1.76 1.72
Me
F
O
N
1.98 2.99 1.00 0.96 2.09 1.00 1.05 2.97
10.0 9.5 9.0 8.5 8.0 7.5 7.0 6.5 6.0 5.5 5.0 4.5 4.0 3.5 3.0 2.5 2.0 1.5 1.0 0.5 0.0
f1 (ppm)

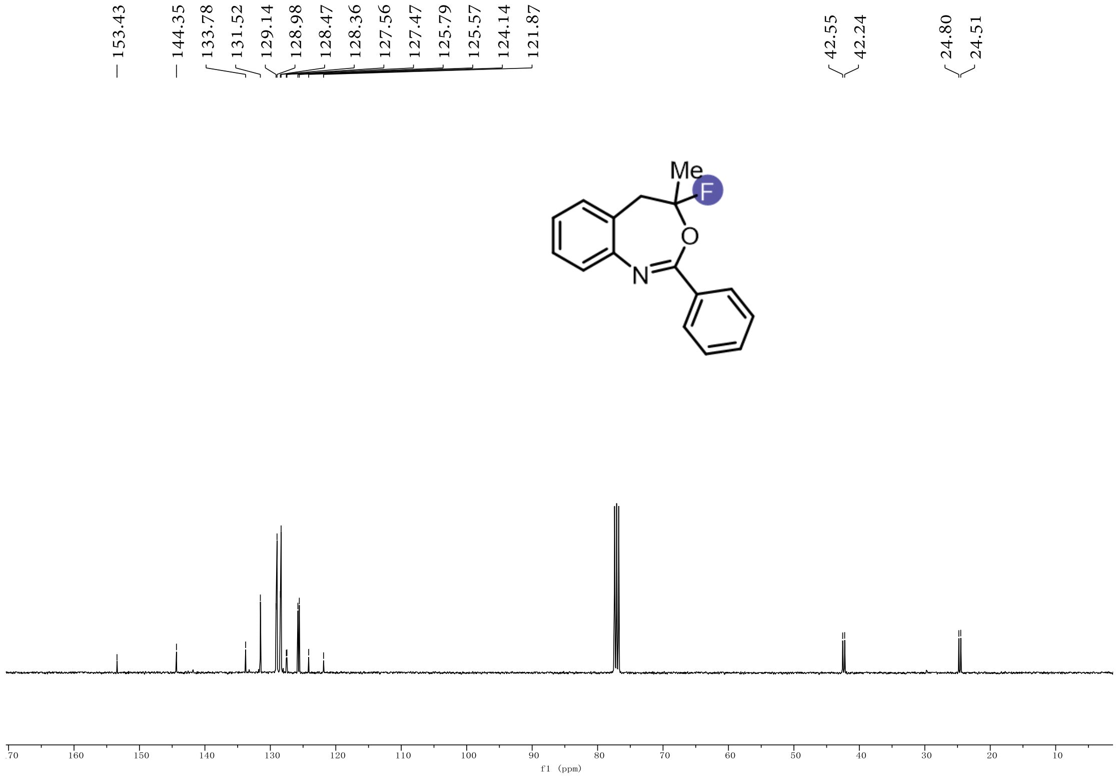
153.43 144.35 133.78 131.52 129.14 128.98 128.47 128.36 127.56 127.47 125.79 125.57 124.14 121.87 42.55 42.24 24.80 24.51
Me
F
O
N
170 160 150 140 130 120 110 100 90 80 70 60 50 40 30 20 10
f1 (ppm)

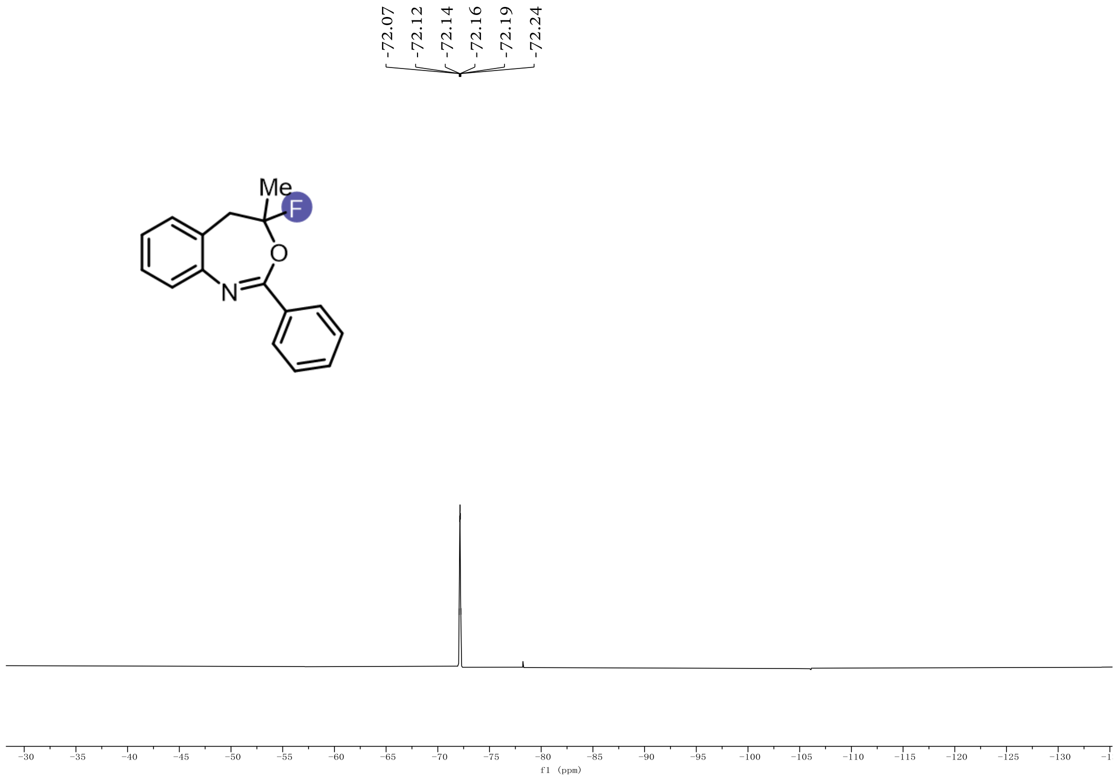
-72.07
-72.12
-72.14
-72.16
-72.19
-72.24
Me
F
O
N
f1 (ppm)

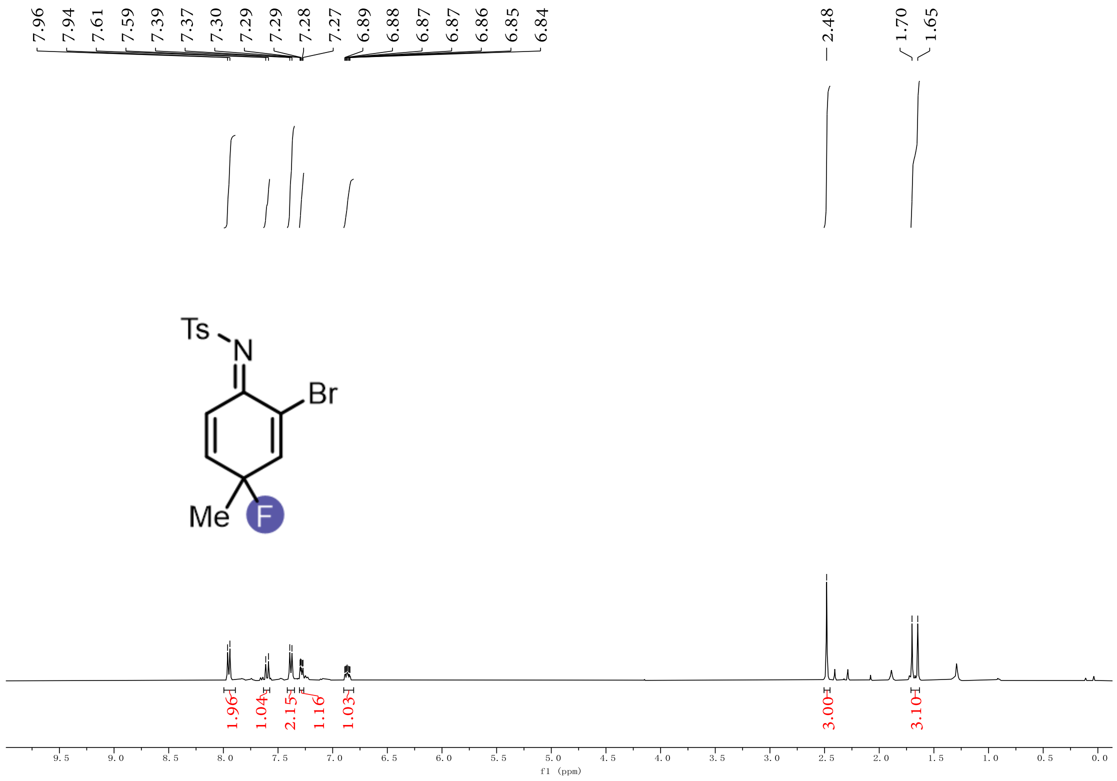
7.96
7.94
7.61
7.59
7.39
7.37
7.30
7.29
7.29
7.28
7.27
6.89
6.88
6.87
6.87
6.86
6.85
6.84
2.48
1.70
1.65
Ts
N
Br
Me
F
1.96
1.04
2.15
1.16
1.03
3.00
3.10
f1 (ppm)

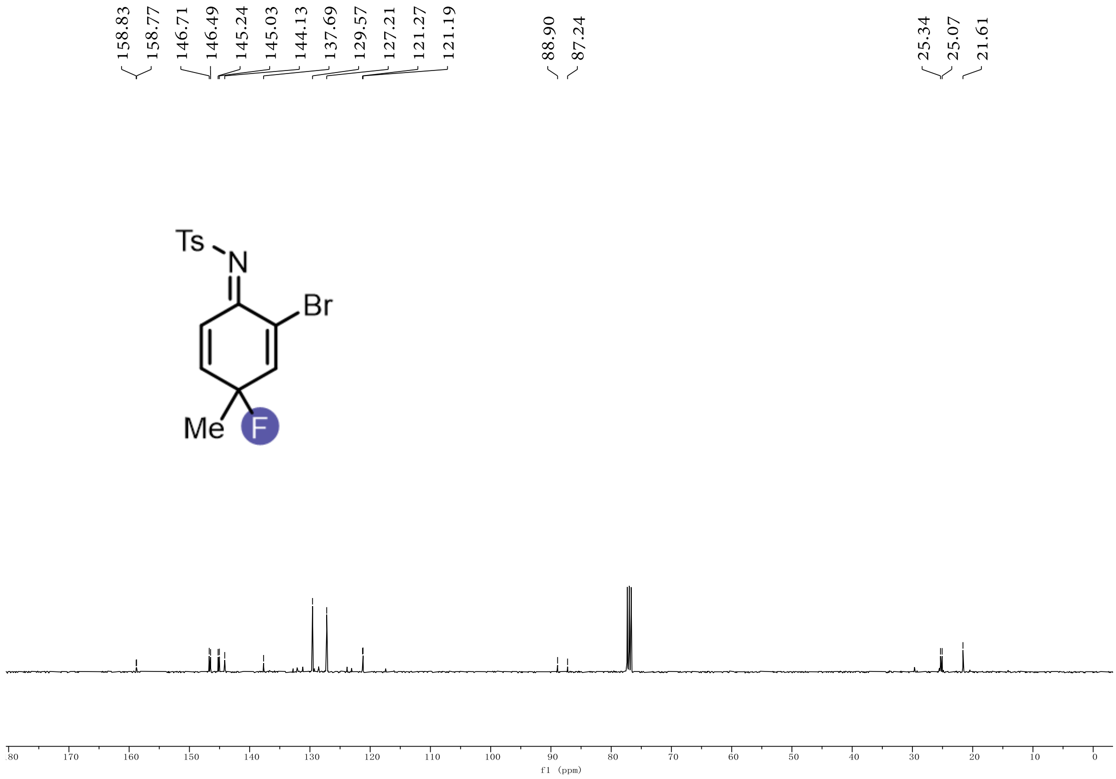
158.83
158.77
146.71
146.49
145.24
145.03
144.13
137.69
129.57
127.21
121.27
121.19
88.90
87.24
25.34
25.07
21.61
Ts
N
Br
Me
F
f1 (ppm)

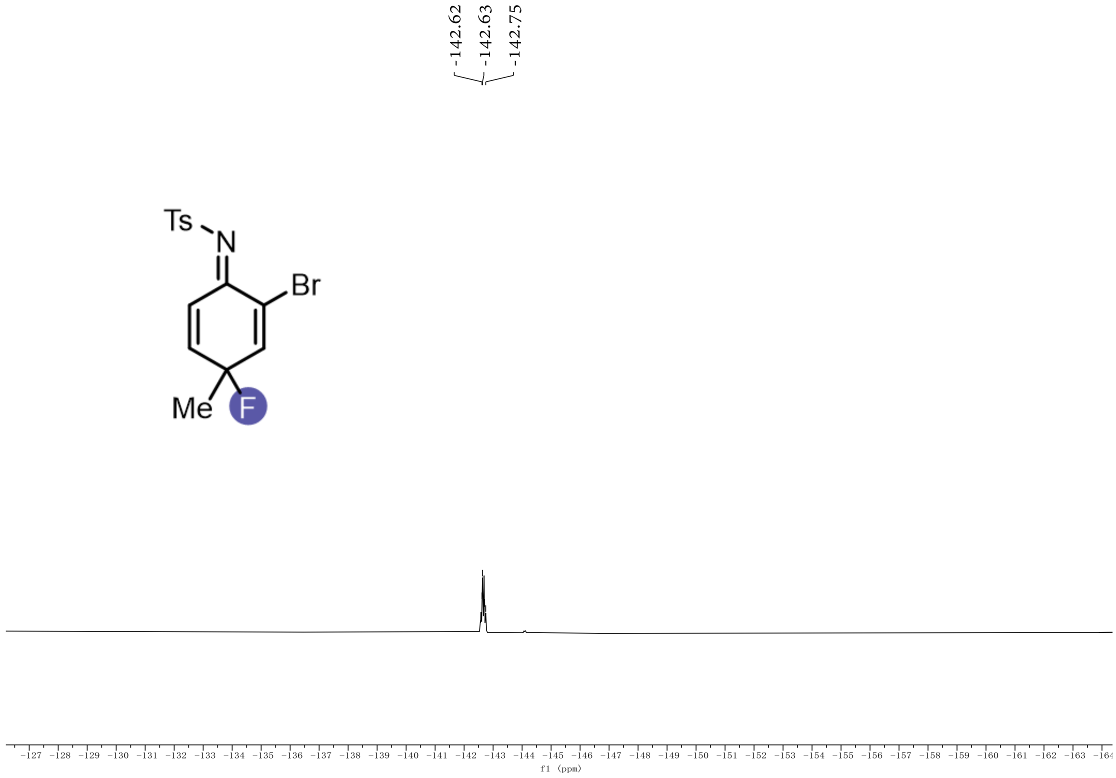
-142.62
-142.63
-142.75
Ts
N
Br
Me
F
f1 (ppm)

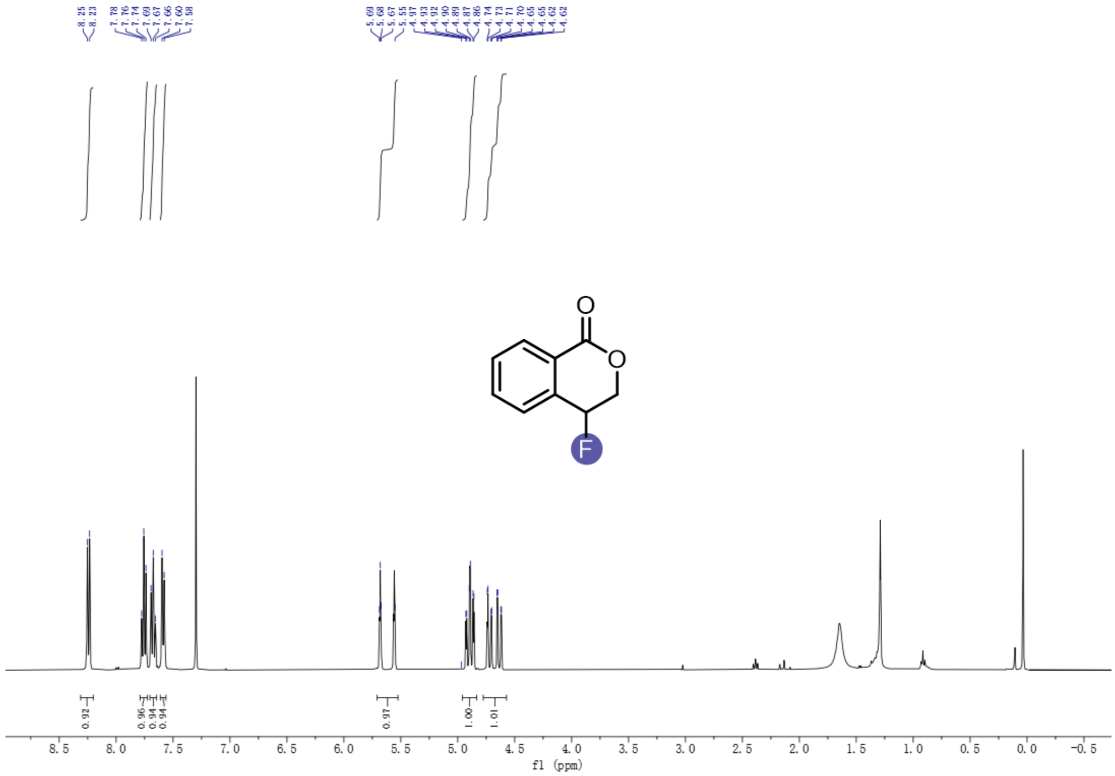

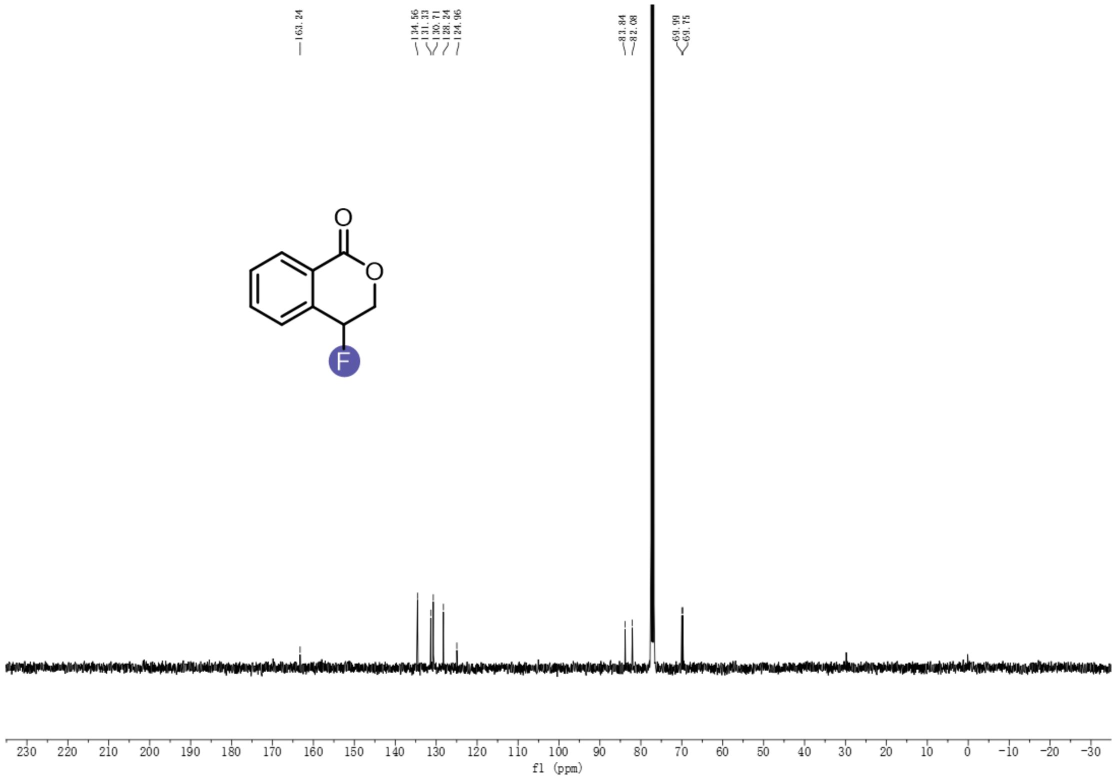

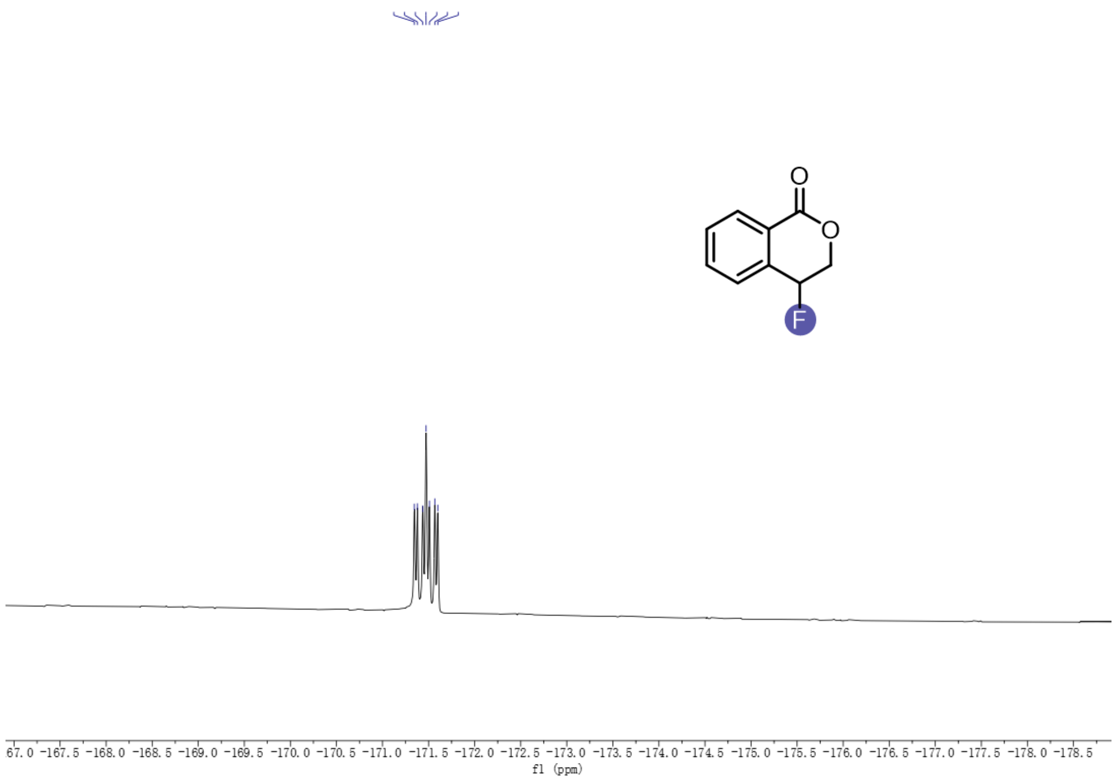

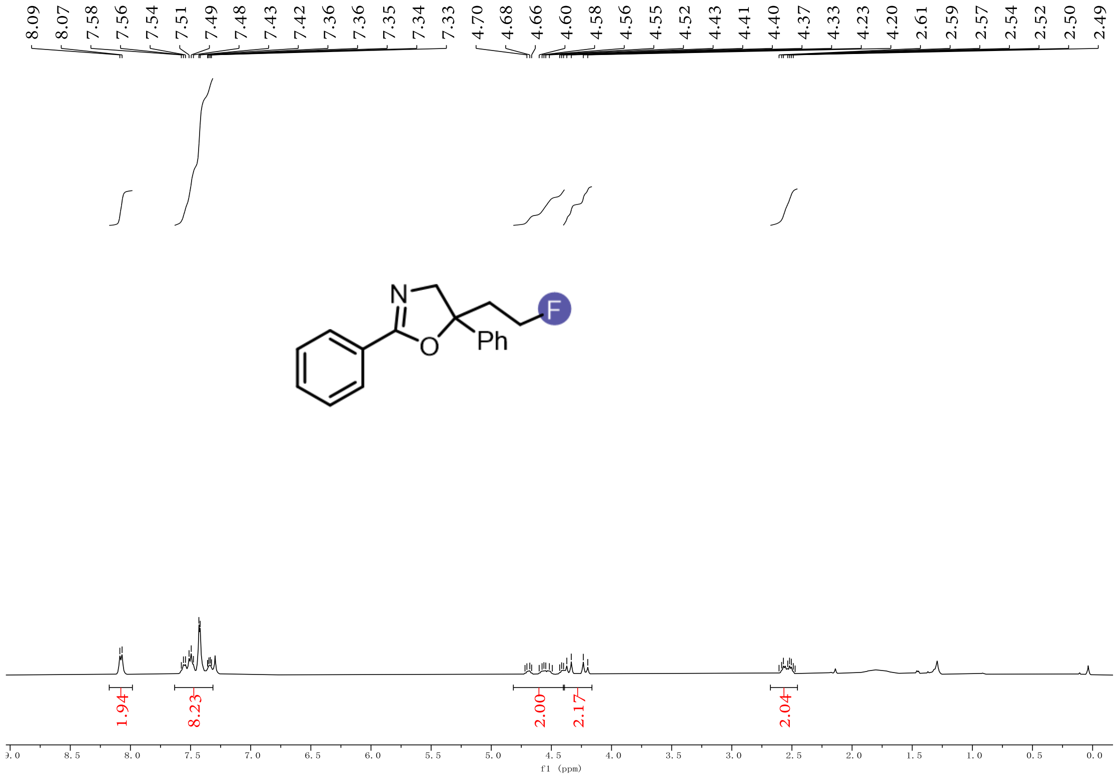

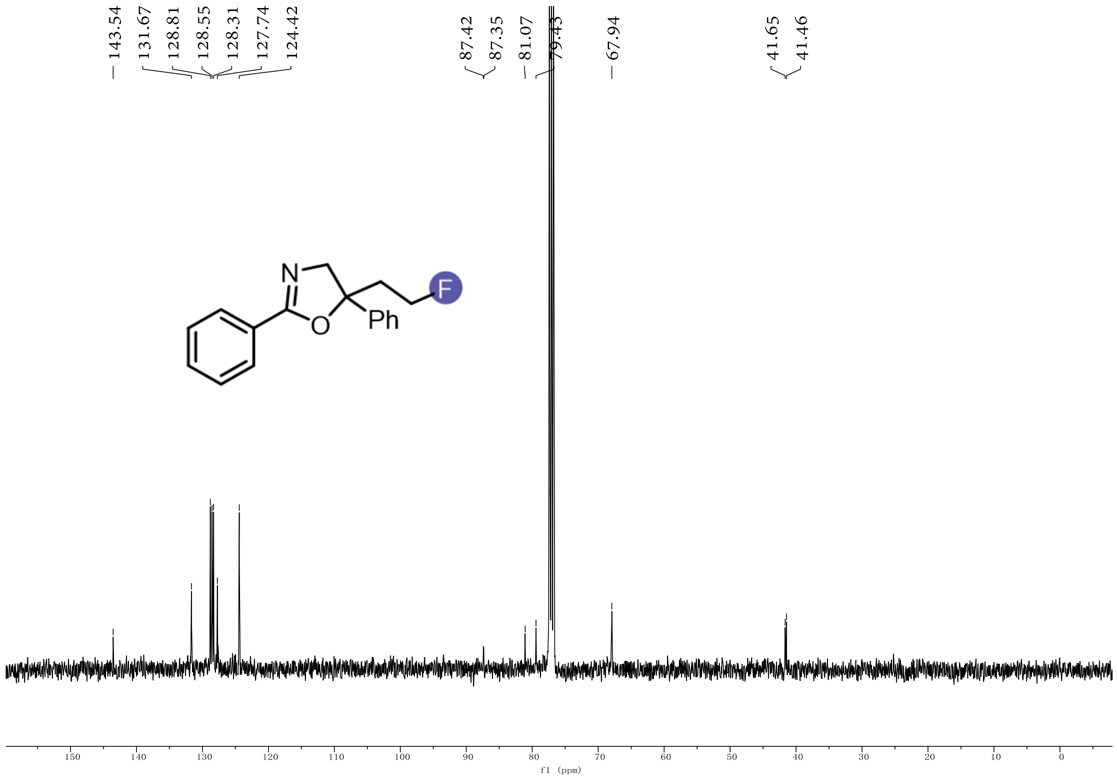
143.54
131.67
128.81
128.55
128.31
127.74
124.42
87.42
87.35
81.07
79.43
67.94
41.65
41.46
N
O
Ph
F
f1 (ppm)

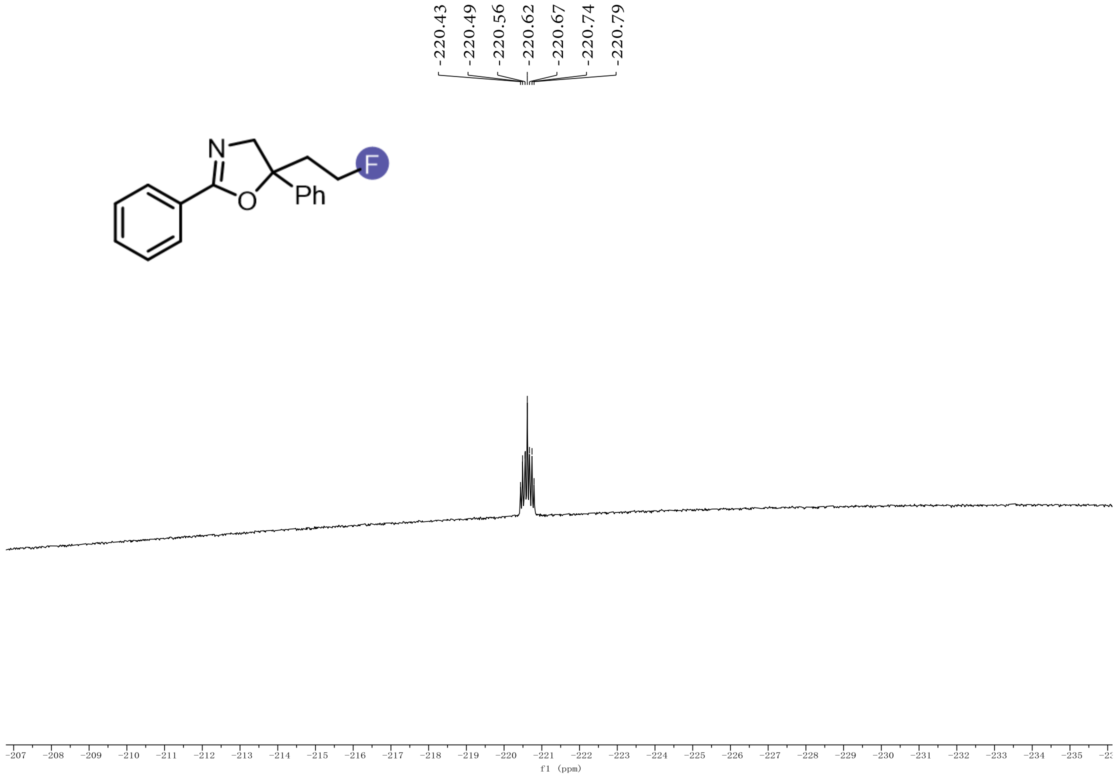
-220.43
-220.49
-220.56
-220.62
-220.67
-220.74
-220.79
N
O
Ph
F
f1 (ppm)

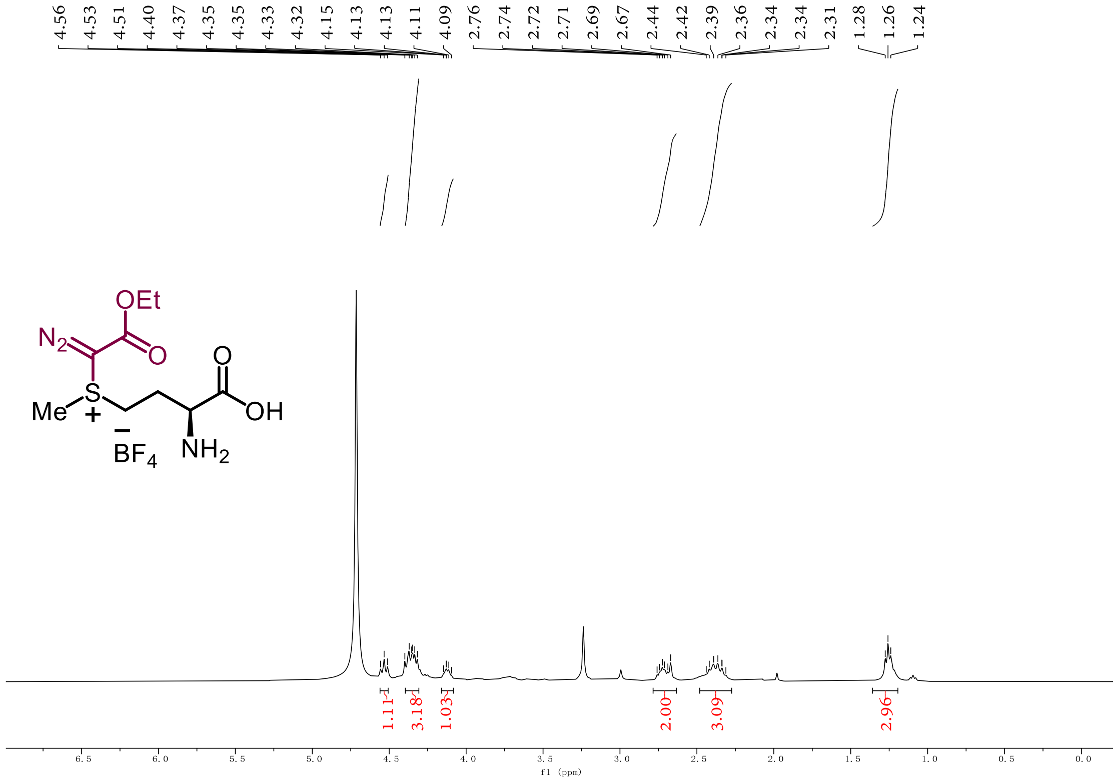
4.56 4.53 4.51 4.40 4.37 4.35 4.35 4.33 4.32 4.15 4.13 4.13 4.11 4.09 2.76 2.74 2.72 2.71 2.69 2.67 2.44 2.42 2.39 2.36 2.34 2.34 2.31 1.28 1.26 1.24
OEt
N2
O
O
Me
S
+
OH
BF4
NH2
1.11
3.18
1.03
2.00
3.09
2.96
f1 (ppm)

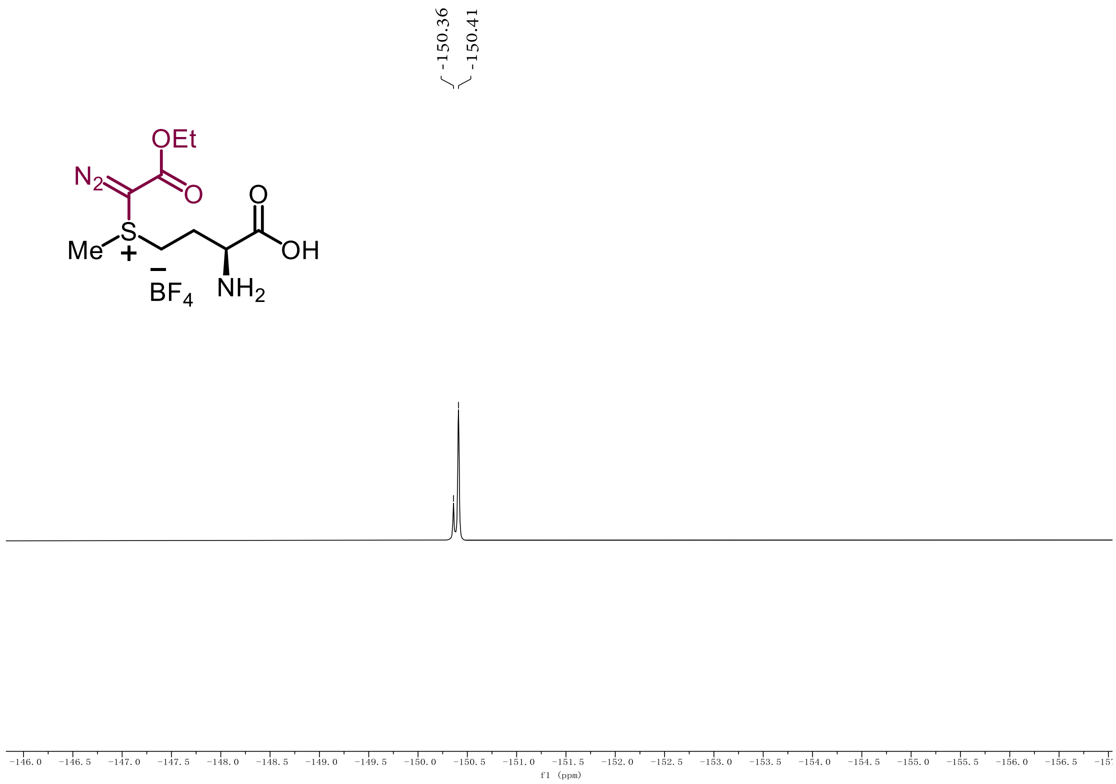
-150.36
-150.41
OEt
N2
O
O
Me
S
+
OH
BF4
NH2
f1 (ppm)

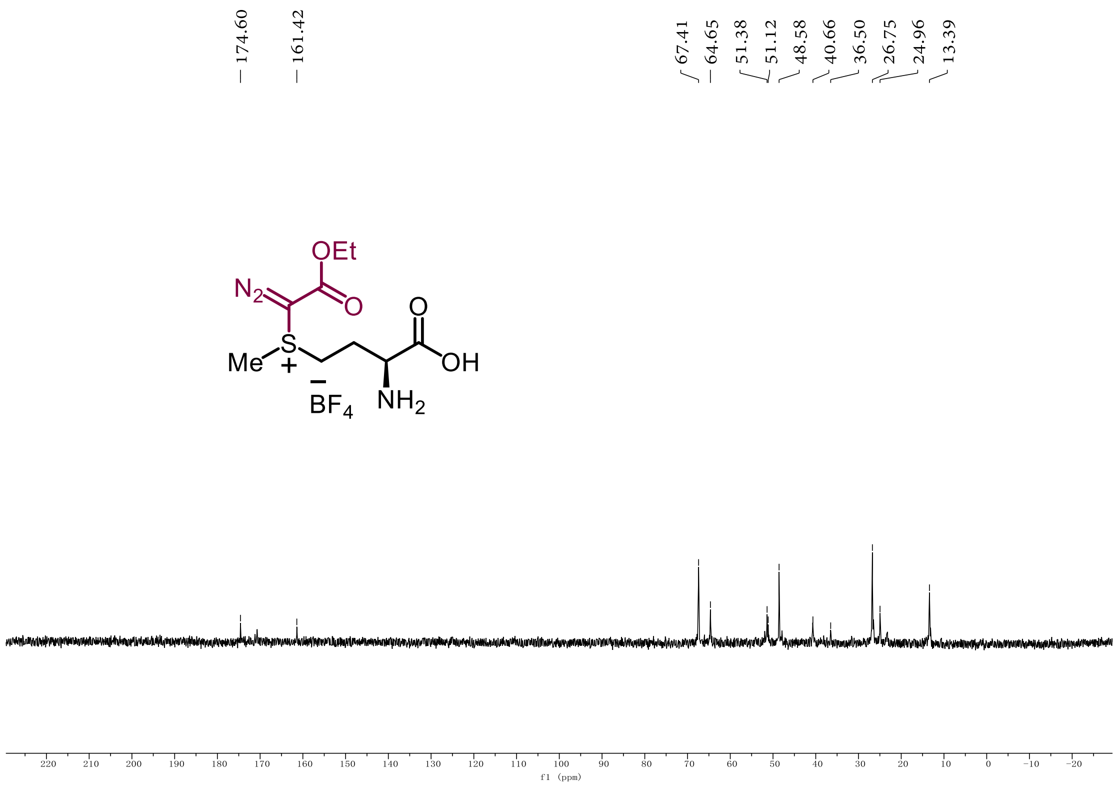
174.60
161.42
67.41
64.65
51.38
51.12
48.58
40.66
36.50
26.75
24.96
13.39
OEt
N2
O
S
Me
+
OH
BF4
NH2
f1 (ppm)

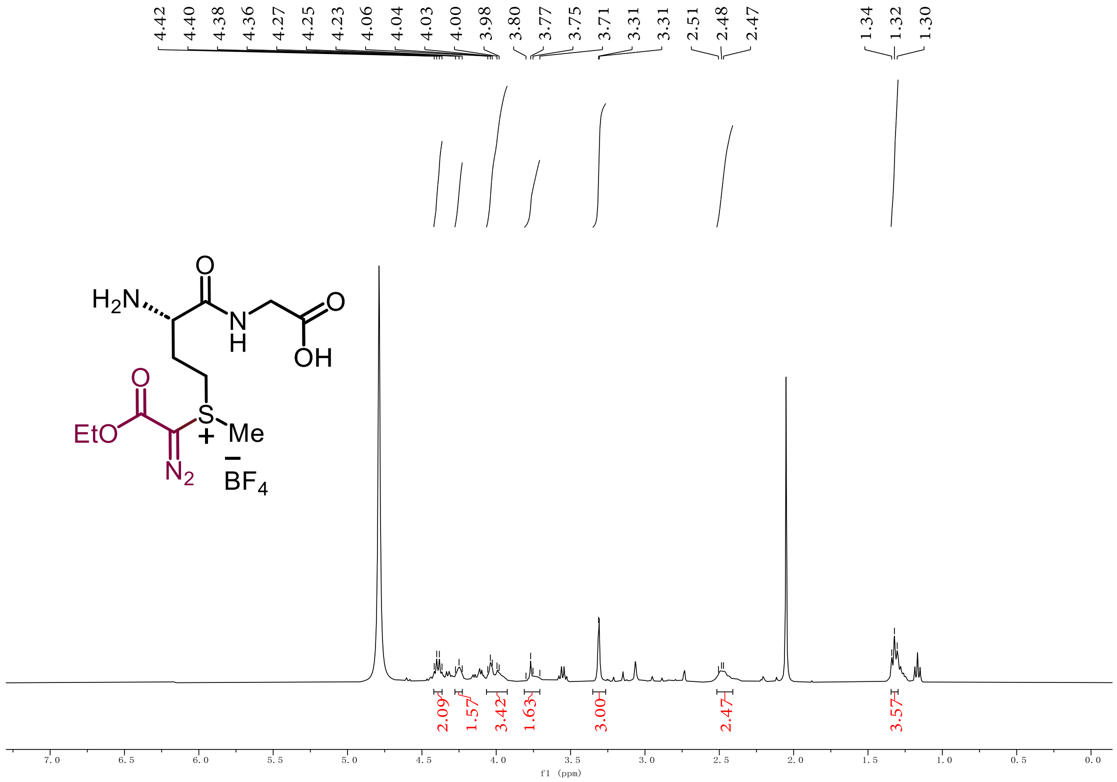
4.42
4.40
4.38
4.36
4.27
4.25
4.23
4.06
4.04
4.03
4.00
3.98
3.80
3.77
3.75
3.71
3.31
3.31
2.51
2.48
2.47
1.34
1.32
1.30
H2N
O
N
H
OH
EtO
S
Me
N2
BF4
2.09
1.57
3.42
1.63
3.00
2.47
3.57
f1 (ppm)

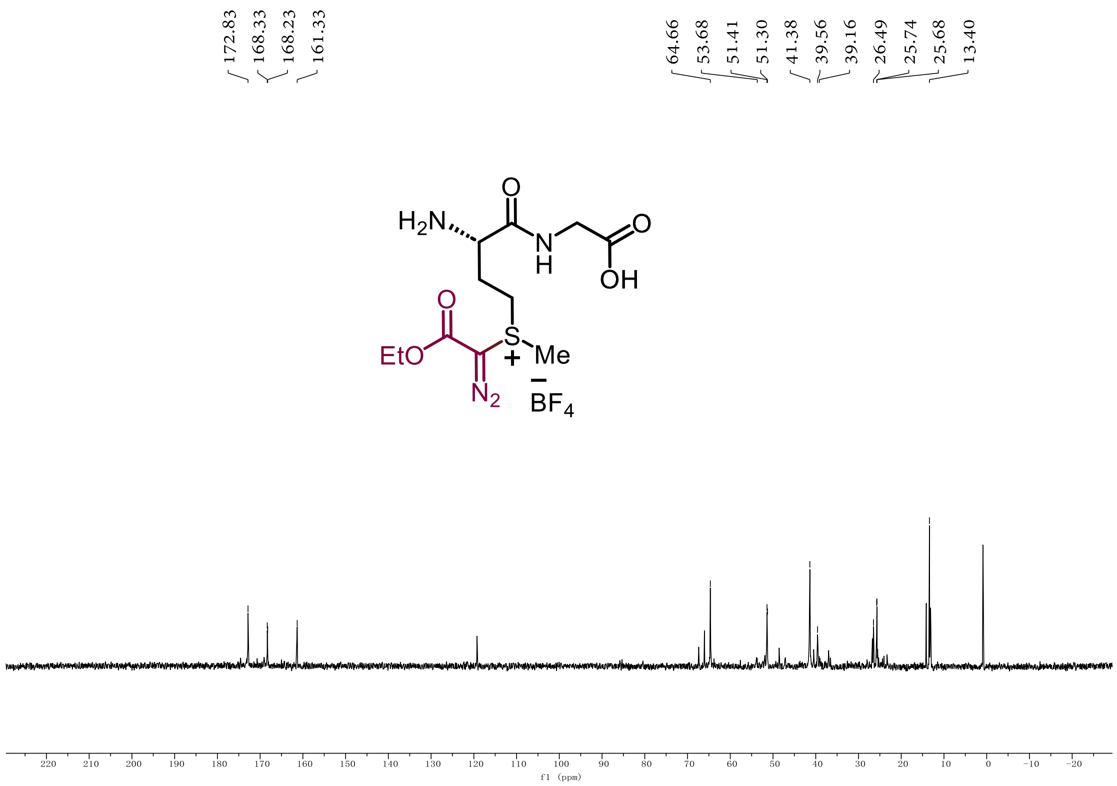
172.83
168.33
168.23
161.33
64.66
53.68
51.41
51.30
41.38
39.56
39.16
26.49
25.74
25.68
13.40
H2N
O
N
H
O
OH
O
EtO
S
Me
N2
BF4
f1 (ppm)

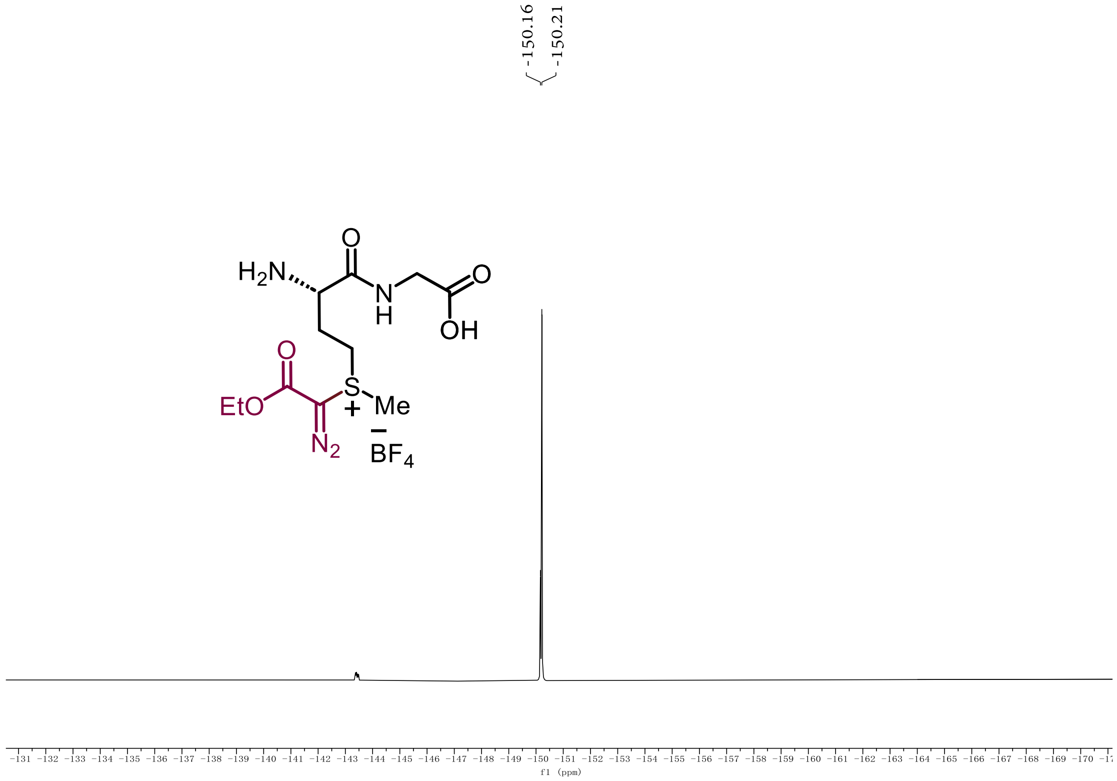
-150.16
-150.21
H2N
O
N
H
O
OH
O
EtO
S
Me
N2
BF4
f1 (ppm)

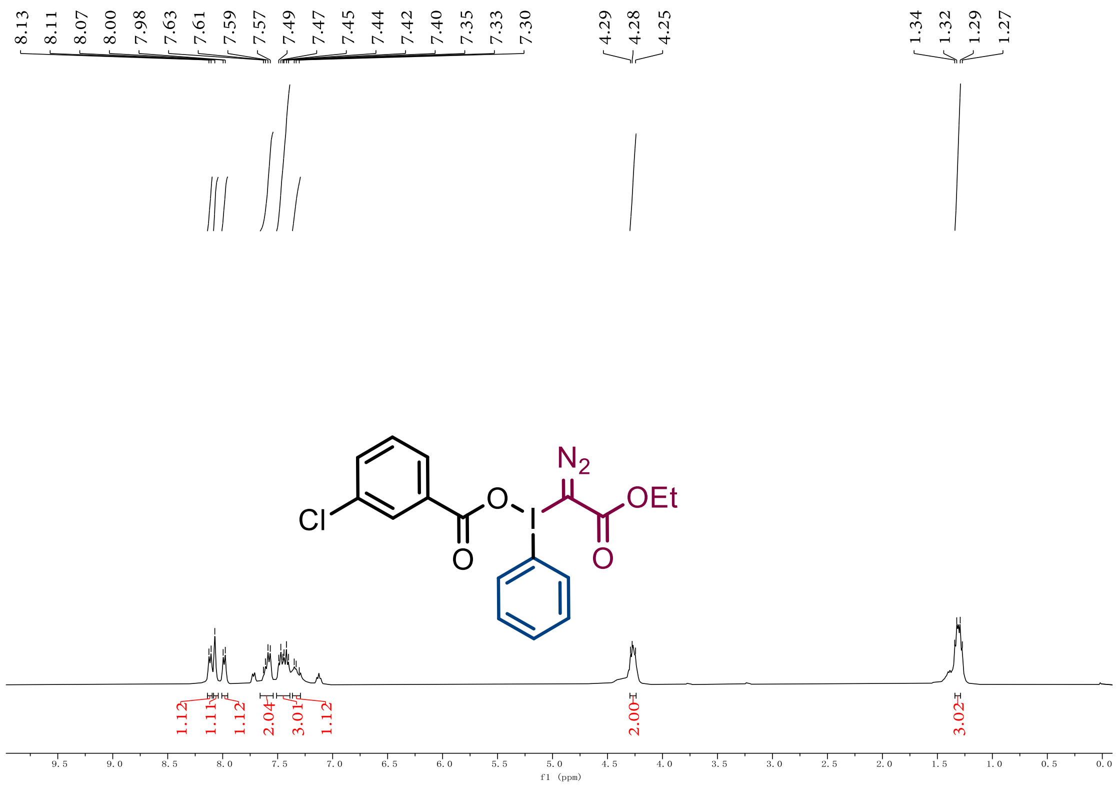
8.13 8.11 8.07 8.00 7.98 7.63 7.61 7.59 7.57 7.49 7.47 7.45 7.44 7.42 7.40 7.35 7.33 7.30
4.29 4.28 4.25
1.34 1.32 1.29 1.27
Cl
O
O
I
N2
OEt
O
1.12 1.11 1.12 2.04 3.01 1.12
2.00
3.02
f1 (ppm)

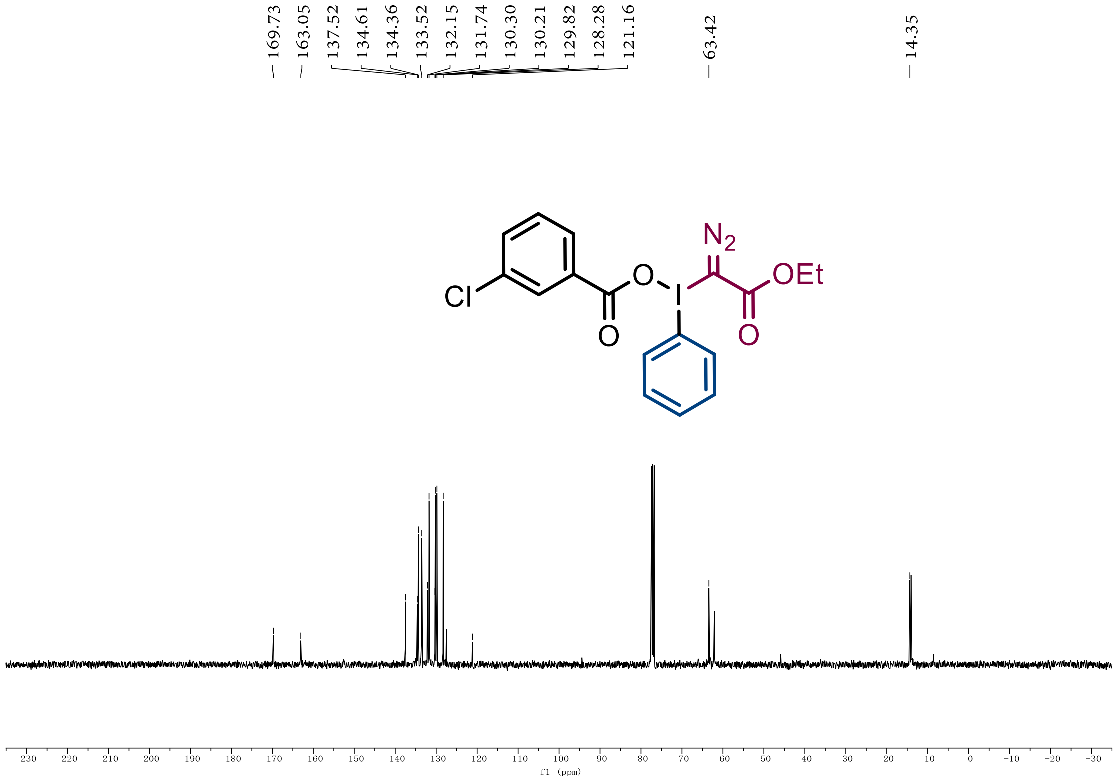
169.73 163.05 137.52 134.61 134.36 133.52 132.15 131.74 130.30 130.21 129.82 128.28 121.16
63.42
14.35
Cl
O
O
I
N2
OEt
O
f1 (ppm)

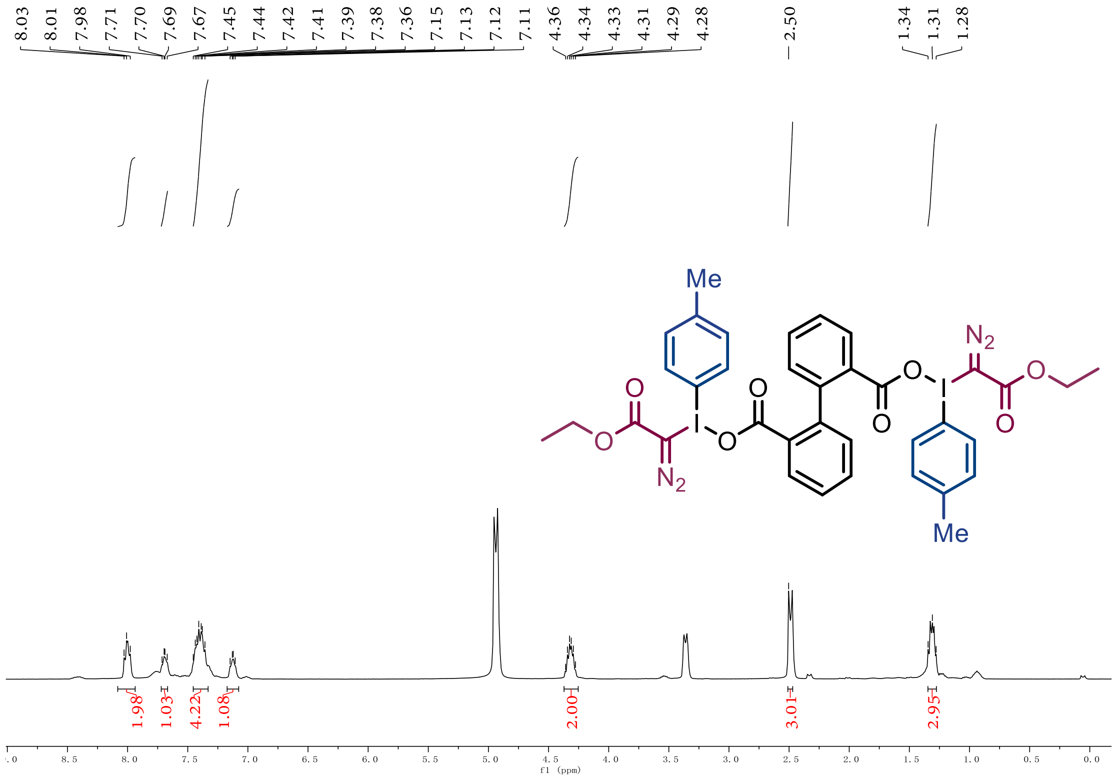
8.03
8.01
7.98
7.71
7.70
7.69
7.67
7.45
7.44
7.42
7.41
7.39
7.38
7.36
7.15
7.13
7.12
7.11
4.36
4.34
4.33
4.31
4.29
4.28
2.50
1.34
1.31
1.28
Me
N_2
O
1.98
1.03
4.22
1.08
2.00
3.01
2.95
f1 (ppm)

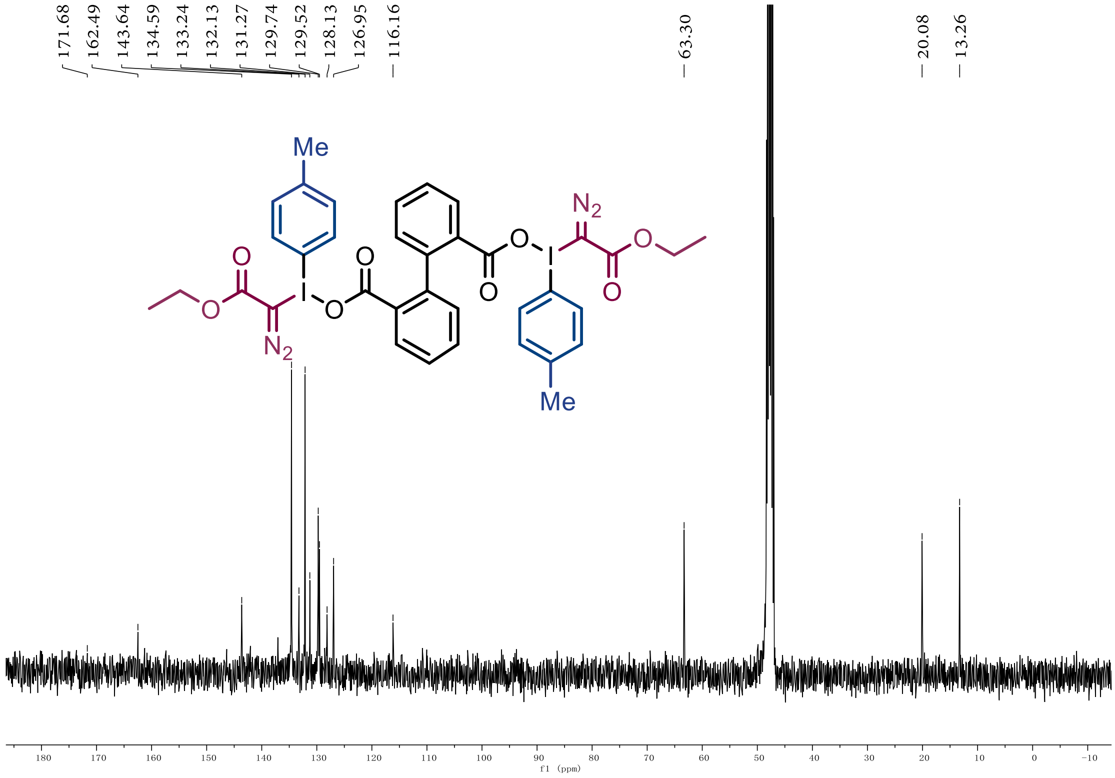
171.68
162.49
143.64
134.59
133.24
132.13
131.27
129.74
129.52
128.13
126.95
116.16
63.30
20.08
13.26
Me
N_2
O
f1 (ppm)
